# One‐Shot Pd(II)‐Catalyzed Multiple C–H Activation Enables Modular Construction of Fluorenylidene Oxindole‐Based Multi(Polycyclic) Aromatic Enes

**DOI:** 10.1002/chem.71221

**Published:** 2026-06-30

**Authors:** Haiyu Wang, Kouki Sakata, Takuma Sato, Masahiro Terada, Tienan Jin

**Affiliations:** ^1^ Department of Chemistry, Graduate School of Science Tohoku University Sendai Japan

**Keywords:** diarylation of alkynes, fluorenylidene oxindole, multi(polycyclic) aromatic enes, one‐shot multiple C─H bond activation, Palladium catalysis

## Abstract

Multi(polycyclic) aromatic enes (MPAEs) are attractive π‐architectures but their synthesis typically requires prefunctionalized, π‐extended building blocks. Herein we report a one‐shot Pd(II)‐catalyzed multiple C–H activation strategy that enables modular access to fluorenylidene/oxindole‐based MPAEs through intramolecular diarylation of *N*‐aryl amide‐tethered biarylalkynes. The synergistic PdCl_2_/PivOH/MnO_2_ system promotes sequential C–H activation/alkyne insertion/C–H activation under mild conditions, furnishing mono‐, di‐, and tri‐olefin‐bridged frameworks in good to high yields. Mechanistic investigations, including kinetic isotope effect measurements and DFT calculations, indicate that the *syn*‐alkyne insertion step is likely rate‐determining, whereas the C–H activation steps proceed through a pivalate‐assisted concerted metalation–deprotonation (CMD) pathway. The method allows rapid assembly of densely conjugated, nonplanar π‐systems from simple linear precursors, including multiple annulations that proceed in a single operation. The resulting 3D π‐architectures display broad and bathochromically shifted absorptions (*λ*_onset_ up to 820 nm), tunable frontier orbital energies, and donor–acceptor electronic structures modulated by π‐extension and heteroatom incorporation. This modular C–H activation platform provides efficient entry to structurally diverse, narrow optical gap MPAEs and expands the synthetic toolbox for advanced optoelectronic π‐materials.

## Graphical abstract

A one‐shot Pd(II)‐catalyzed C─H activation strategy enables the rapid conversion of simple linear propiolamides into structurally diverse mono‐, di‐, and tri‐olefin‐bridged MPAEs. This cascade process orchestrates up to six sequential C–H activations in a single operation, providing streamlined access to highly conjugated architectures.

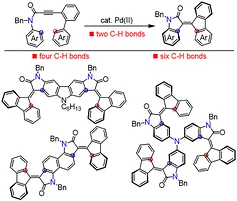

## 1 Introduction

Fulvalene and its heteromerous analogues, exemplified by 9,9′‐bifluorenylidene (99′BF), represent prototypical multi(polycyclic) aromatic enes (MPAEs) composed of two polyaromatic units linked through an ethylenic (C═C) bridge (Scheme 1a). Their inherently twisted alkene geometries, coupled with extended π‐surfaces, give rise to distinctive electronic structures and functions, including charge‐transport capability, dye characteristics, mechano,‐ and thermochromism, molecular switching, and oligoradicaloid behavior [1, 2, 3, 4, 5, 6, 7, 8, 9, 10, 11, 12, 13, 14, 15, 16, 17, 18, 19, 20, 21, 22, 23, 24, 25]. More architecturally sophisticated π‐extended MPAEs containing multiple C═C bridges can further amplify these properties by simultaneously enhancing conjugation and enforcing 3D distortion [16, 17, 18, 19, 20, 21, 22, 23, 24]. Conventional access to such frameworks typically relies on prefunctionalized carbonyl or thione precursors through Barton–Kellogg [13, 16, 22, 24] or Wittig–Horner [17, 18, 19] olefinations, or on Pd‐catalyzed Suzuki/Heck sequences [26, 27] (Scheme 1b). Although powerful, these approaches necessitate prior construction of π‐extended fragments and prefunctionalization of aromatic components, which diminishes step economy and limits modular diversification. Consequently, the development of more direct and convergent strategies for constructing complex MPAEs from simple arenes remains an important synthetic challenge.

**SCHEME 1:**
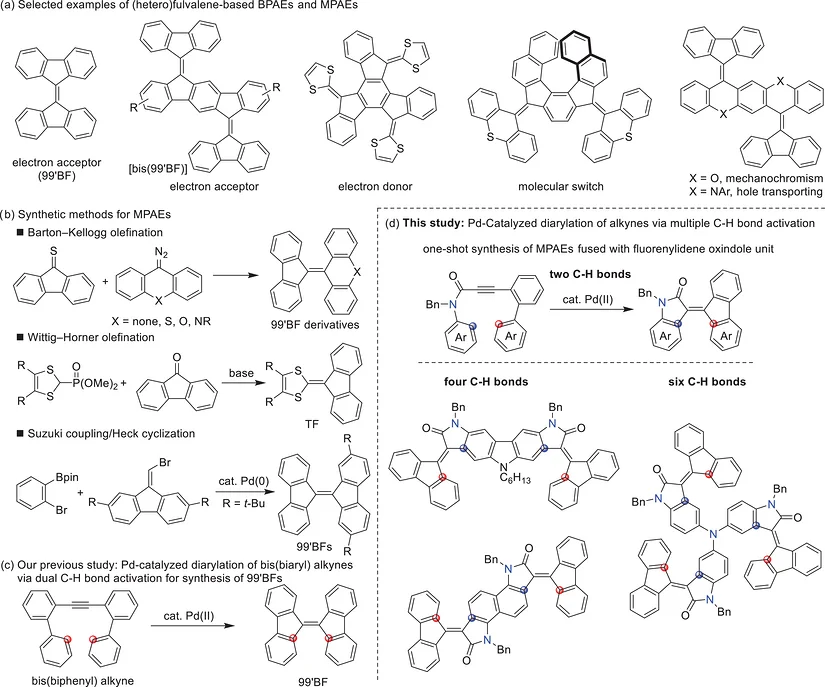
Synthetic methodologies of MPAEs.

Transition‐metal‐catalyzed multiple C–H activation offers a conceptually distinct solution by enabling direct transformation of unfunctionalized arenes into elaborate π‐architectures [28, 29, 30, 31, 32, 33, 34, 35, 36, 37, 38, 39]. Notably, Itami and coworkers demonstrated Pd‐catalyzed annulative π‐extension of polycyclic aromatic hydrocarbons with silicon‐bridged aromatics, providing streamlined access to nanographenes via multiple C–H activation events in a single operation [35]. In parallel, transition‐metal‐catalyzed diarylation of alkynes has emerged as a versatile route to multisubstituted alkenes. Intramolecular variants are particularly attractive, as they furnish structurally constrained π‐systems, including bis(polycyclic) enes, in a concise manner [37, 38, 39, 40, 41, 42, 43, 44, 45, 46, 47, 48, 49, 50, 51, 52]. In this context, we recently reported a Pd(II)‐catalyzed intramolecular diarylation of bis(biaryl) alkynes, enabling rapid construction of fulvalene‐ and azafulvalene‐based MPAEs through dual aromatic C–H activation (Scheme 1c) [37, 38]. However, this transformation was limited to alkyne substrates bearing two biaryl groups at the alkynyl termini. Building on this foundation and inspired by annulative π‐extension chemistry [53], we envisioned that biphenyl alkynes tethered to an *N*‐phenyl amide could undergo sequential dual *ortho*‐C–H activation at both the biphenyl and *N*‐phenyl units, with the latter proceeding through amide‐directed C–H activation (Scheme 1d). Such a process would provide a modular platform for the synthesis of a new class of MPAEs by simultaneously constructing fluorene and oxindole motifs in a one‐shot transformation. Importantly, these twisted π‐architectures, integrating electron‐accepting fluorene [1, 16] and donor–acceptor oxindole [54] subunits through a sterically constrained alkene bridge, were expected to afford structurally programmable π‐systems with electron‐deficient character, long‐wavelength absorption, and finely tunable optoelectronic properties.

Herein, we disclose a one‐shot Pd(II)‐catalyzed multiple C–H activation strategy that converts simple linear propiolamide precursors into mono‐, di‐, and tri‐olefin‐bridged MPAEs containing fluorenylidene‐oxindole untits (Scheme 1d). The transformation orchestrates up to six C–H activation events in a single operation. Mechanistic investigations, supported by experimental studies and theoretical calculations, are consistent with a sequential C–H activation/insertion/C–H activation/reductive elimination manifold. The resulting π‐architectures display broad absorption profiles, tunable redox properties, and promising potential for application in organic optoelectronic materials.

## 2 Results and Discussion

We commenced our investigation with optimization of the reaction conditions using 3‐(biphenyl‐2‐yl)‐*N*‐benzyl‐N‐phenylpropiolamide (**1a**) as the model substrate and our previously developed catalyst system [37], PdCl_2_/PivOH/MnO_2_. To our delight, **1a** was converted to the corresponding bis(polycyclic) aromatic enes (BPAEs) **2a** in 93% isolated yield (Table 1, entry 1). To gain insight into the role of each component, we systematically varied the reaction conditions. In the absence of PivOH, the combination of PdCl_2_ and MnO_2_ resulted in a dramatic decrease in yield (entry 2). Similarly, replacing PivOH with AcOH led to significantly diminished efficiency (entry 3). These results indicate that PivOH is essential, likely facilitating aromatic C─H bond activation via a pivalate‐assisted concerted metalation–deprotonation (CMD) pathway [55]. We next evaluated AgOPiv and Cu(OPiv)_2_ as alternative oxidants and pivalate sources, as they had proven effective in our previous aromatic C–H coupling reactions [36, 56]. However, both oxidants were completely inactive under the present conditions (entries 4 and 5). Subsequent examination of other palladium catalysts revealed that PdBr_2_ also promoted the transformation, whereas Pd(OAc)_2_ and Pd(OPiv)_2_ afforded **2a** in low yields (entries 6–8). The diminished reactivity observed with PdCl_2_ alone (entry 2) and with Pd(OPiv)_2_ (entry 8) underscores the importance of combining PdCl_2_ with PivOH. We attribute this synergy to the in situ formation of an active Cl–Pd–OPiv species that facilitates dual C–H activation [38]. Alternative oxidants were also evaluated. Replacing MnO_2_ with CuO, Ag_2_O, or 1,4‐benzoquinone (BQ) resulted in significantly lower yields or no detectable reaction (entries 9–11). It was noted that reducing the amount of MnO_2_ from 5 equiv to 3 equiv decreased the yield of **2a** to 80%, likely because the lower loading of the insoluble MnO_2_ oxidant reduced the efficiency of the heterogeneous oxidation process in the solvent system (entry 12). Collectively, these studies identify PdCl_2_/PivOH/MnO_2_ as a uniquely effective catalytic system for the present diarylation of alkynes via dual C–H activation events. Finally, the influence of the *N*‐substituent was investigated under the standard conditions. Substrates **1b** and **1c**, bearing either no substituent or an acyl substituent on nitrogen, underwent almost complete decomposition without formation of the desired products (entries 13 and 14); in the latter case, this outcome is likely attributable to *N*‐deacylation under the acidic conditions. In contrast, *N*‐methyl‐ and *N*‐phenyl‐substituted propiolamides **1d** and **1e** afforded the corresponding products **2d** and **2e** in 76% and 66% yields, respectively (entries 15 and 16), albeit with reduced efficiency compared to **1a**.

**TABLE 1:** Screening of reaction conditions and *N*‐substituent effect.^a^

		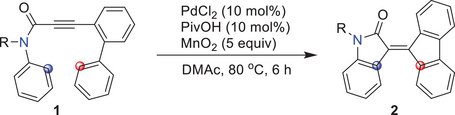			

Entries	R (1)	Deviation from standard conditions	2	2 [%]^b^	1 [%]^b^
1		none		95 and 93^c^	0
2	Bn (**1a**)	without PivOH	**2a**	68	9
3		AcOH instead of PivOH		40	42
4		AgOPiv (2 equiv) instead of PivOH and MnO_2_		0	89
5		Cu(OPiv)_2_ (2 equiv) instead of PivOH and MnO_2_		0	92
6		PdBr_2_ instead of PdCl_2_		84	0
7		Pd(OAc)_2_ instead of PdCl_2_		32	36
8		Pd(OPiv)_2_ instead of PdCl_2_		25	38
9		CuO instead of MnO_2_		5	75
10		Ag_2_O instead of MnO_2_		0	89
11		BQ instead of MnO_2_		trace	81
12		MnO_2_ (3 equiv)		80	0
13	H (**1b**)	none	**2b**	0	0
14	Ac (**1c**)	none	**2c**	trace	5
15	Me (**1d**)	none	**2d**	76^c^	0
16	Ph (**1e**)	none	**2e**	66^c^	0

^a^ Standard conditions: **1** (0.1 mmol), Pd (10 mol%), PivOH (10 mol%) and MnO_2_ (5 equiv) in DMAc (0.125 M, 0.8 mL) at 80°C for 6 h.

^b^ ^1^H NMR yield determined using anisole as an internal standard.

^c^ Isolated yields after silica gel chromatography are shown.

Next, the substrate scope of various *N*‐benzyl‐substituted propiolamides **1** was investigated for the synthesis of BPAEs **2** under the standard conditions (Table 2). Propiolamides bearing either an electron‐donating methyl or an electron‐withdrawing fluoro substituent at the *meta*‐position of the C─H activation site, afforded the corresponding BPAEs **2f** and **2** **g** in good yields, indicating that the electronic nature of the substituent has little influence on the reaction efficiency. Moreover, substrates bearing strong electron‐withdrawing functional groups such as ‐CO_2_Me and ‐NO_2_ at the R‐position were also compatiable with the reaction conditions, delivering the corresponding BPAEs **2** **h** and **2i** in 79% and 76% yields, respectively. Gratifyingly, substrates containing extended aromatic systems were also well tolerated. The C─H bonds of *N*‐(naphthalen‐1‐yl) and *N*‐(phenanthren‐9‐yl) substituents underwent smooth activation to furnish the π‐extended BPAEs **2j** and **2k** in 64% and 65% yields, respectively. Furthermore, propiolamides bearing electron‐rich *N*‐heteroaryl substituents, such as benzo[*b*]thiophene and 9*H*‐carbazole, furnished the corresponding π‐extended bis(polycyclic) heteroaromatic enes **2l**‐**2n** in comparable yields. Notably, product **2n** was obtained as a single regioisomer; no isomer resulting from C–H activation at the 4‐position of the carbazole moiety was detected. The methodology also enabled the construction of more complex π‐frameworks. A [5]helicene‐conjugated BPAE **2o** was synthesized in 77% yield via the C–H diarylation of the corresponding binaphthyl‐derived propiolamide. In addition, a propiolamide bearing an electron‐rich 2,2′‐bithienyl substituent proved compatible with the catalytic system, affording BPAE **2p** in 59% yield. The twisted molecular structure of **2p** was unambiguously established by single‐crystal X‐ray diffraction analysis [57].

**TABLE 2:** Substrate scope for synthesis of BPAEs with mono‐olefin bridge. ^a^ ^,^ ^b^

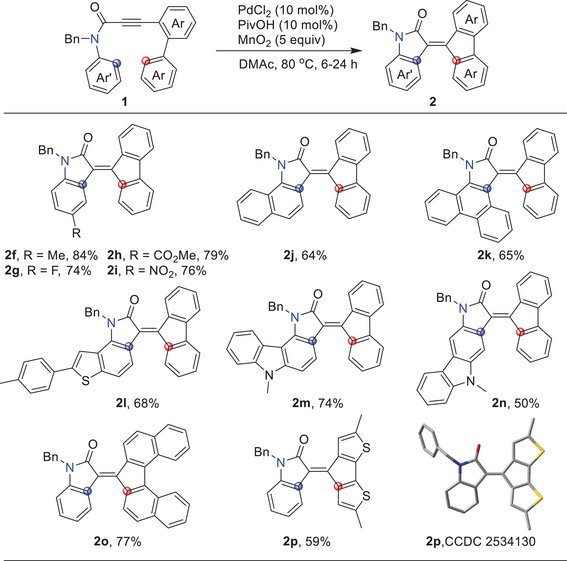

^a^ Standard conditions: 1 (0.1 mmol), PdCl_2_ (10 mol%), PivOH (10 mol%) and MnO_2_ (5 equiv) in DMAc (0.125 M, 0.8 mL) at 80°C for 6–24 h.

^b^ Isolated yields after silica gel chromatography are shown.

To gain mechanistic insight into the alkyne diarylation process, substrate **1q**, bearing a methyl substituent at the para position of the biphenyl moiety, was examined under mild conditions by lowering the reaction temperature (Scheme 2a). When **1q** was treated with PdCl_2_/PivOH (50 mol%) at room temperature for 0.5 h, a mixture of products was obtained in 24% yield with a 2:1 ratio of (*E*)‐**2q** to (*Z*)‐**2q**. After removal of the Pd catalyst and MnO_2_ oxidant by short silica gel chromatography, the isolated product mixture underwent partial post‐isomerization at room temperature over 24 h, affording an (*E*)‐**2q**/(*Z*)‐**2q** ratio of 1.3:1. In contrast, under the standard conditions (80°C), the reaction of **1q** delivered the corresponding products in 95% yield with an approximately 1.1:1 ratio of (*E*)‐**2q** and (*Z*)‐**2q**. These results indicate that (*E*)‐**2q**, which is expected to arise from *syn*‐insertion during the alkyne diarylation process, is formed preferentially at the early stage of the reaction. Subsequently, (*E*)‐**2q** undergoes partial isomerization to (*Z*)‐**2q**, leading to an erosion of selectivity over time. This behavior is consistent with our previous observations [37]. To probe the aromatic C–H palladation step, two intramolecular competitive kinetic isotope effect (KIE) experiments were conducted using deuterium‐labeled substrates **1a‐*d*** and **1a'‐*d*** bearing a deuterium atom at the *ortho*‐position of the phenyl ring (Scheme 2b). KIE values of 3.2 and 3.8 were obtained under the standard conditions. These moderate primary KIE values indicate that C─H bond activation is involved in the catalytic process. To further clarify the rate‐determining step in this dual C–H activation process, two intermolecular competitive KIE experiments were conducted using 1:1 mixtures of **1a‐*d*_5_**/**1a** and **1a'‐*d*_5_**/**1a** (Scheme 2c). In contrast to the intramolecular experiments, the intermolecular experiments exhibited only negligible KIE values (1.1), suggesting that C─H bond cleavage is not involved in the rate‐determining step of the catalytic cycle. The substantial KIEs observed in the intramolecular experiments reflect the intrinsic preference for C─H over C─D bond cleavage within the same molecule, whereas the near‐unity KIEs obtained from the intermolecular experiments represent the kinetics of the overall catalytic cycle. Collectively, these results suggest that although C–H activation is a kinetically relevant step, a subsequent elementary step is likely responsible for determining the overall reaction rate [58]. Collectively, these results suggest that although C–H activation is a kinetically relevant step, a subsequent elementary step is likely responsible for determining the overall reaction rate.

**SCHEME 2:**
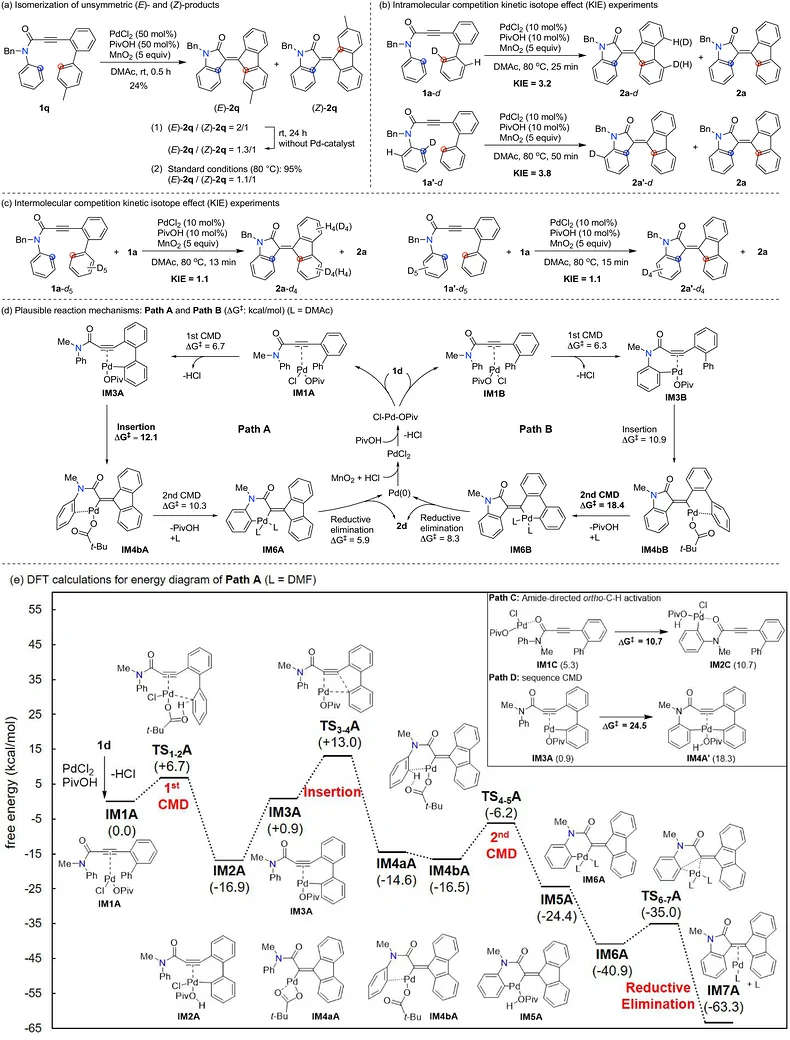
Mechanistic studies and plausible reaction mechanism with energy diagram. KIE values were determined based on the product yields measured by ^1^H NMR spectroscopy, using anisole as an internal standard. DFT computations were performed at the M06/def2‐SVP level.

To gain insight into the energetically favorable pathways of this alkyne diarylation, density functional theory (DFT) calculations were performed on key intermediates (IMs) and transition states (TSs) for the reaction of **1d**. The computed catalytic cycles and corresponding energy profiles are summarized in Scheme 2d,e (see also Figure S4). Initially, ligand exchange between PdCl_2_ and PivOH generates the active Cl–Pd–OPiv species in situ with concomitant elimination of HCl. Coordination of this Pd(II) complex to the alkyne moiety of **1d** affords two nearly isoenergetic π‐complexes, **IM1A** (path A) and **IM1B** (path B, Figure S4). In path A, C–H palladation at the 2′‐position of the biphenyl unit in **IM1A** proceeds via a CMD mechanism to form aryl–Pd intermediate **IM2A** through **TS_1–2_A** (ΔG^‡^ = 6.7 kcal mol^−1^) [55]. Similarly, in path B, C–H palladation at the *ortho*‐position of the *N*‐phenyl ring in **IM1B** occurs via **TS_1–2_B** with a comparable barrier (ΔG^‡^ = 6.4 kcal mol^−1^), yielding **IM2B**. These similar activation barriers indicate that CMD‐mediated C–H activation at either aryl ring is initially feasible. In contrast, a third possibility (path C), involving amide‐directed C–H palladation (**IM1C** to **IM2C**), is both kinetically and thermodynamically disfavored due to its higher barrier (ΔG^‡^ = 10.7 kcal mol^−1^) and the lower stability of **IM1C** and **IM2C** (Scheme 2e, inset). Subsequent elimination of HCl from **IM2A** and **IM2B** furnishes **IM3A** and **IM3B**, respectively. Intramolecular *syn*‐insertion of the alkyne into the aryl–Pd bond then produces vinyl–Pd intermediates **IM4aA** and **IM4aB** via **TS_3–4_A** (ΔG^‡^ = 12.1 kcal mol^−1^) and **TS_3–4_B** (ΔG^‡^ = 10.9 kcal mol^−1^), respectively. An alternative pathway (path D), involving direct second CMD‐mediated C–H palladation of **IM3A** to generate biaryl–Pd complex **IM4A′** as proposed in our previous study [37], was also examined. However, this pathway is strongly disfavored owing to its high activation barrier (ΔG^‡^ = 24.5 kcal mol^−1^) and the low stability of **IM4A′** (Scheme 2e, inset). The bidentate complexes **IM4aA** and **IM4aB** subsequently rearrange to the more stable monodentate species **IM4bA** and **IM4bB**, enabling a second CMD‐mediated C–H palladation [55]. This step proceeds via **TS_4–5_A** (ΔG^‡^ = 10.3 kcal mol^−1^) to give palladacycle **IM5A**, or via **TS_4–5_B** (ΔG^‡^ = 18.4 kcal mol^−1^) to give **IM5B**. Elimination of PivOH from **IM5A** and **IM5B** affords the more stable palladacycles **IM6A** and **IM6B**, which undergo reductive elimination through **TS_6–7_A** (ΔG^‡^ = 5.9 kcal mol^−1^) and **TS_6–7_B** (ΔG^‡^ = 18.4 kcal mol^−1^), respectively. Finally, decoordination of **IM7A** and **IM7B** releases the desired product **2d** and generates a Pd(0) species. Overall, for path A, the *syn*‐insertion step (ΔG^‡^ = 12.1 kcal mol^−1^) is identified as the rate‐determining step, consistent with the intermolecular KIE experiments (Scheme 2c). In contrast, for path B, the second CMD process (ΔG^‡^ = 18.4 kcal mol^−1^) constitutes the rate‐limiting step. Comparison of these activation barriers, together with the KIE results, suggest that *syn*‐insertion in path A is kinetically more favorable than the second CMD step in path B, supporting path A as the predominant pathway in this catalytic transformation. It should be noted, however, that the present DFT calculations were performed at the M06/def2‐SVP level and do not explicitly account for dispersion corrections or comprehensive solvation effects in the key transition states, which may influence the quantitative accuracy of the calculated energy barriers. The catalytic cycle is completed by oxidation of the Pd(0) species to PdCl_2_ by in situ‐generated Cl_2_ under acidic conditions [59]. Cl_2_ is formed via the well‐established redox reaction between MnO_2_ and HCl, highlighting the cooperative role of PdCl_2_, MnO_2_, and PivOH in enabling this transformation.

To demonstrate the synthetic power and modularity of this dual C–H activation strategy, we explored one‐shot twofold and threefold dual C–H activation reactions for the rapid construction of multi‐olefin‐bridged MPAEs (Scheme 3). We first examined twofold dual C–H activation of substrates **3a** and **3b**, bearing two 3‐(biphenyl‐2‐yl)propiolamide units at the 1,4‐positions of a phenylene core and the 1,5‐positions of a naphthalene core, respectively (Scheme 3a,b). Under PdCl_2_/PivOH (20 mol%) catalysis with MnO_2_ (10 equiv), both substrates underwent efficient multiple annulation in a single operation, affording the corresponding tri(polycyclic) aromatic enes (TPAEs) **4a** and **4b** in 37% and 30% yield, respectively. These products feature two oppositely oriented fluorene units conjugated to π‐extended central scaffolds, 5,7‐dihydropyrrolo[2,3‐*f*]indole‐2,6(1H,3H)‐dione and 1,3,6,8‐tetrahydroindolo[7,6‐*g*]indole‐2,7‐dione, through two olefin bridges, generating rigid and twisted multi‐ene architectures. The ability to assemble such densely conjugated frameworks in one step highlights the rapid increase in molecular complexity enabled by this strategy. The scope extends beyond simple benzenoid systems. Heteroaromatic cores proved particularly effective: dibenzo[b,d]thiophene‐ and 9*H*‐carbazole‐derived substrates **3c** and **3d**, each bearing two tethered propiolamide units, underwent smooth multiple annulation to furnish **4c** and **4d** in 82% and 47% yield, respectively (Scheme 3c). Notably, both annulations occurred selectively on the same side of the π‐extended core, delivering densely fused, side‐oriented bis(olefin‐bridged) structures with remarkable positional control. This inherent structural bias enables the programmable construction of architecturally defined, nonplanar π‐systems. Encouraged by these results, we next investigated threefold dual C–H activation (Scheme 3d). Triphenylamine‐centered triamide **5a** successfully underwent six sequential C–H activations under the standard conditions to afford propeller‐shaped MPAE **6a**, bearing three olefin bridges, in 48% yield. This transformation demonstrates the feasibility of orchestrating multiple C–H activations in a single operation and provides direct access to twisted, 3D π‐frameworks from simple polyamine precursors. Importantly, all poly(propiolamide) substrates were readily prepared from 3‐(biphenyl‐2‐yl)propioloyl chloride and the corresponding di‐ or triamines, underscoring the operational simplicity and modularity of the platform. Collectively, this one‐shot multiple dual C–H activation enables the concise and programmable assembly of structurally complex, multi‐olefin‐bridged π‐architectures from simple linear building blocks. The capacity to control multiple annulations in a single step offers an efficient entry to twisted and 3D conjugated frameworks that are otherwise difficult to access.

**SCHEME 3:**
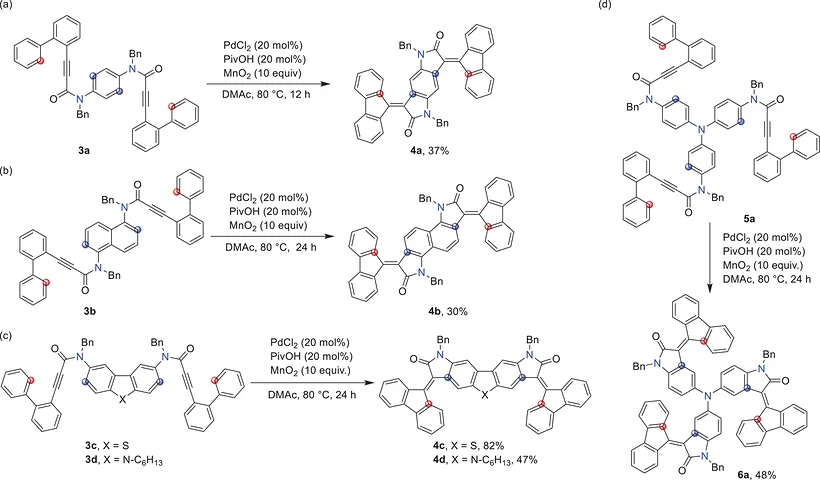
Synthesis of MPAEs with di‐ and tri‐olefin bridges via multiple C‐H activation method.

The UV–vis absorption spectra of the MPAEs measured in dilute CH_2_Cl_2_ are shown in Figure 1a, b, and the corresponding absorption maxima (*λ*_max_), absorption onsets (*λ*_onset_), and electrochemical data are summarized in Figure 1c (see also Figure S1). BPAE **2a** exhibits a broad and markedly red‐shifted absorption band (*λ*_max_ = 496 nm) compared with fulvalene‐based 99′BF (*λ*_max_ = 458 nm), which can be mainly attributed to stabilization of its lowest unoccupied molecular orbital (LUMO). Extension of the π‐conjugation on the oxindole moiety (**2j–2n**) further bathochromically shifts the absorption, affording broad bands with *λ*_max_ values in the range of 545–594 nm. These absorptions are significantly red‐shifted relative to those of **2a** and **2o** (*λ*_max_ = 493 nm), the latter incorporating a π‐extended [5]helicene unit in place of the fluorene moiety (Figures 1a and S1). In contrast, **2p** bearing an electron‐donating 2,6‐dimethyl‐4*H*‐cyclopenta[2,1‐b:3,4‐*b*′]dithiophene unit displays a substantially extended absorption profile, with *λ*_max_ and *λ*_onset_ at 614 and 802 nm, respectively. The weak low‐energy band is assigned to the HOMO–LUMO transition with a very small oscillator strength (*f* = 0.0149), as supported by time‐dependent DFT (TD‐DFT) calculations (Figure S5 and Table S9). The more π‐extended TPAEs **4a–4d**, featuring two olefin bridges, exhibit broad absorptions with *λ*_max_ values between 593 and 659 nm. Notably, **4a** and **4b** show remarkably long *λ*_onset_ values of 820 and 801 nm, respectively. These low‐energy absorptions correlate with their high‐lying HOMOs (−5.36 and −5.30 eV) and low‐lying LUMOs (−3.75 and −3.66 eV), consistent with DFT‐predicted donor–acceptor (D–A) electronic structures (Figure 1c,d). Interestingly, substitution with heteroatoms significantly modulates the frontier orbital energies. TPAE **4c**, incorporating an electron‐donating sulfur atom, shows a deeper HOMO (−5.48 eV) than **4a** and **4b**, whereas **4d** containing an electron‐donating nitrogen atom exhibits both a higher HOMO (−5.28 eV) and LUMO (−3.58 eV). Orbital contour analyses reveal that the HOMO of **4c** is delocalized from the π‐extended central core to the olefin bridges, while its LUMO is distributed over the entire framework with negligible contribution from the sulfur atom (Scheme 1d). By contrast, in **4d** the HOMO is localized on the electron‐donating polyaromatic core, whereas the LUMO is primarily delocalized from the two fluorene units to the olefin bridges, resulting in a spatially separated D–A electronic structure. These distinct frontier orbital distributions, together with the heteroatom effects in **4c** and **4d**, are consistent with the trends observed in the cyclic voltammetry (CV) measurements. Finally, the 3D MPAE **6a** exhibits a weak, broad absorption band spanning 520–811 nm, assigned to a HOMO–LUMO transition with a very small oscillator strength (*f* = 0.0496) (Table S8). Despite its weak absorption, **6a** possesses a high‐lying HOMO (−5.03 eV), arising from the strongly electron‐donating central triarylamine unit. DFT calculations disclose an unusual three‐directional D–A electronic structure, in which the HOMO is delocalized from the central nitrogen atom toward the oxindole moieties, whereas the LUMO is predominantly localized on the heterofulvalene segments. Overall, systematic π‐extension and heteroatom incorporation effectively modulate the frontier molecular orbitals and optical gaps of these MPAEs, leading to pronounced bathochromic shifts and tunable donor–acceptor electronic structures.

**FIGURE 1:**
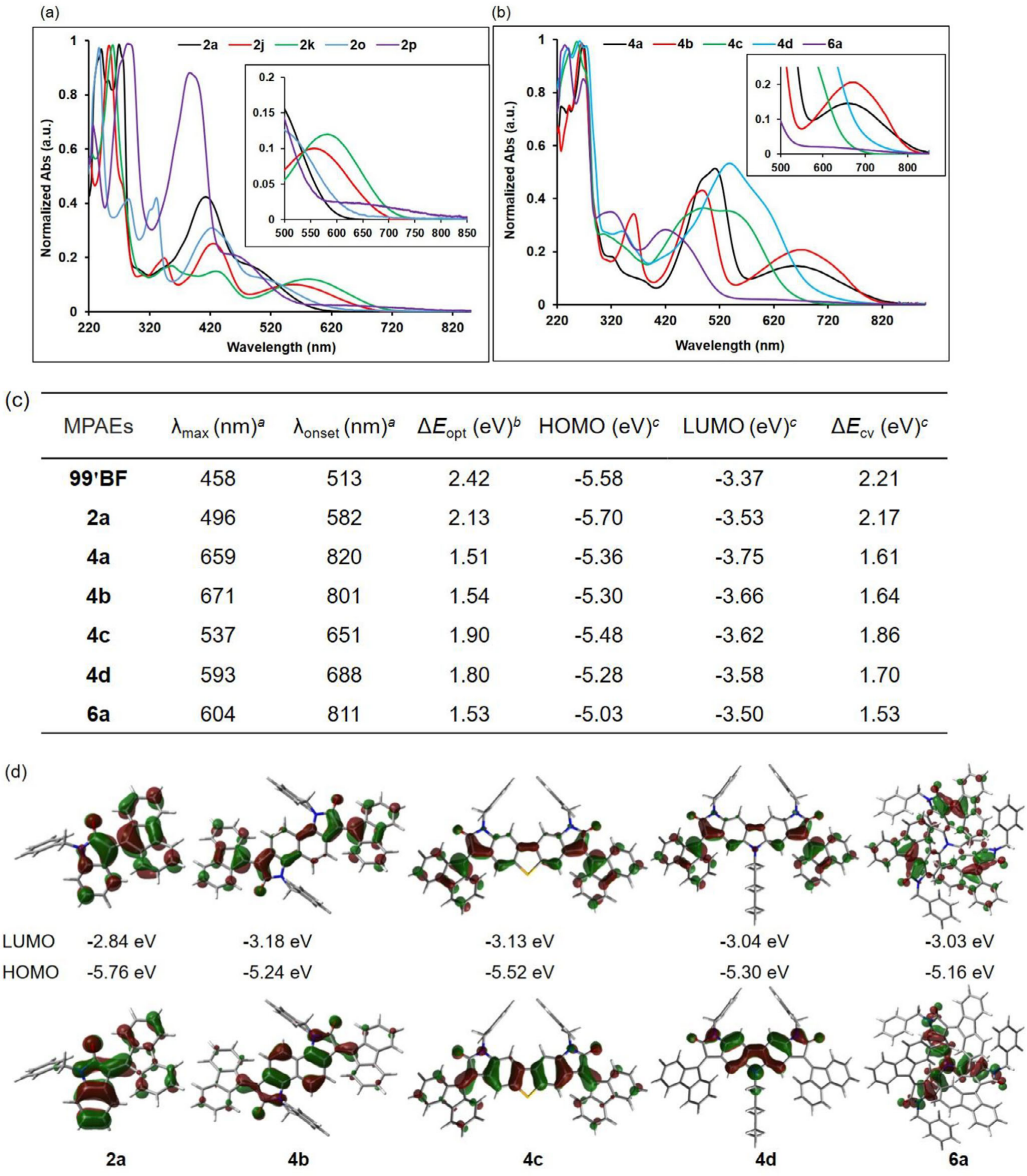
(a) UV‐Vis absorption spectra **2a**, **2j**, **2k**, **2o**, and **2p** in CH_2_Cl_2_ (10^−5^ M). (b) UV‐vis absorption spectra of **4a**, **4b**, **4c**, **4d**, and **6a** in CH_2_Cl_2_ (10^−5^ M). (c) Summary of optical and electrochemical properties. *^a^ λ*_max_ and *λ*_onset_ estimated from the maximum absorption at the longest wavelength and absorption onsets, respectively. *^b^* Optical energy gap (Δ*E*_opt_) was estimated from *λ*_onset_ according to the equation, Δ*E*_opt_ (eV) = 1240/*λ*_onset_. *^c^* HOMO and LUMO were calculated from the oxidation and reduction potentials by CV measurement and the Fc/Fc+ (−4.80 eV) was used as an external standard. *^d^* HOMO–LUMO energy gap (Δ*E*_cv_) was calculated from the HOMO and LUMO energy levels by CV measurement. (d) DFT calculations of HOMO and LUMO contours, and energy levels of MPAEs were carried out at the B3LYP/6‐311G(d,p) level. Geometry of compound **6a** was optimized at M06‐2X/6‐31G(d,p) level. The time‐dependent DFT (TD‐DFT) calculations were conducted at the CAM‐B3LYP/6‐311+G(2d,p) level for the excited state calculation using the ground‐state geometry.

Finally, we demonstrated that this protocol is applicable to a range of biphenyl alkynes bearing diverse heteroatom‐tethered aryl groups, enabling the synthesis of structurally diverse heteroatom‐containing fluorene‐based BPAEs. For example, the reaction of ester substrate **7a** under the standard Pd‐catalyzed conditions afforded the corresponding BPAE **8a**, featuring fluorene and benzofuranone motifs, in 50% yield (Scheme 4a). In contrast, thioester substrates, such as *S*‐phenyl 3‐(biphenyl‐2‐yl)prop‐2‐ynethioate, failed to produce the corresponding BPAE, likely because sulfur‐induced catalyst poisoning deactivated the Pd catalytic system. Moreover, unlike the *N*‐phenyl amide substrates **1**, propargyl amines, such as *N*‐(3‐(biphenyl‐2‐yl)prop‐2‐yn‐1‐yl)‐*N*‐benzylaniline, and ketones, such as 4‐(biphenyl‐2‐yl)‐1‐phenylbut‐3‐yn‐2‐one, gave complex mixtures without formation of the desired BPAE products under the standard conditions. Notably, sulfonamide substrate **7b**, *N*‐(biphenyl‐2‐ylethynyl)‐*N*‐ethylbenzenesulfonamide, and phosphonate substrate **7c**, diphenyl (biphenyl‐2‐ylethynyl)phosphonate, exhibited remarkably high reactivity, affording the corresponding BPAEs **8b** and **8c** incorporating 2,3‐dihydrobenzo[*d*]isothiazole 1,1‐dioxide and 3*H*‐benzo[*d*][1,2]oxaphosphole 2‐oxide motifs, respectively, in high yields (Scheme 4bc). The UV–vis absorption spectra showed that the *λ*_max_ values of the new BPAEs **8a–8c** were in the range of 400–446 nm, which are significantly blue‐shifted relative to that of **2a** (496 nm) (Figure S2 and Table S2). Compounds **8a** and **8c** exhibited low‐lying HOMO (−5.87 and −5.96 eV, respectively) and LUMO (−3.55 and −3.57 eV, respectively) energy levels, reflecting the electron‐deficient nature of the benzofuranone and 3*H*‐benzo[*d*][1,2]oxaphosphole 2‐oxide motifs (Figure S3 and Table S2). In contrast, compound **8b** showed relatively high‐lying HOMO (−5.44 eV) and LUMO (−2.96 eV) energy levels, probably due to the electron‐donating enamine‐like structure of the 2,3‐dihydrobenzo[*d*]isothiazole moiety.

**SCHEME 4:**
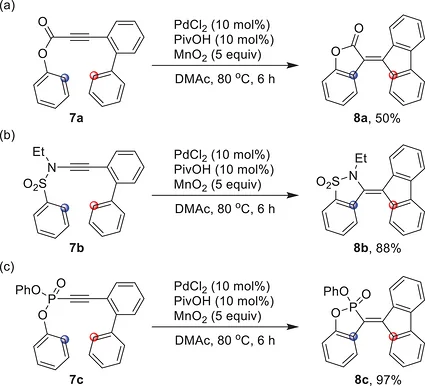
Synthesis of various heteroatom‐containing BPAEs via dual C‐H activation method.

## 3 Conclusion

In summary, we have developed a one‐shot Pd(II)‐catalyzed multiple aromatic C–H activation strategy that enables concise and modular construction of fluorenylidene oxindole‐based MPAEs. The cooperative PdCl_2_/PivOH/MnO_2_ catalytic system facilitates sequential dual C–H activation and alkyne insertion, allowing efficient formation of mono‐, di‐, and tri‐olefin‐bridged π‐frameworks from readily accessible propiolamide precursors. Mechanistic experiments and DFT studies support a pivalate‐assisted CMD pathway, with syn‐insertion identified as the kinetically preferred route, rationalizing the observed selectivity. Notably, this platform orchestrates up to six C–H activation events in a single operation, rapidly increasing molecular complexity and delivering rigid, twisted, and 3D π‐architectures that are otherwise challenging to access. Systematic π‐extension and heteroatom incorporation enable precise modulation of frontier molecular orbitals, leading to pronounced bathochromic shifts, narrow optical gaps, and tunable donor–acceptor electronic structures. By integrating step economy, structural diversity, and functional tunability, this work highlights the power of C–H activation in advanced π‐molecule construction and provides a versatile foundation for the rational design of next‐generation optoelectronic materials.

## 4 Experimental Section

### 4.1 General Procedure for the Synthesis of 2a

To an anhydrous DMAc (1.6 mL, 0.125 M) solution were added 3‐(biphenyl‐2‐yl)‐*N*‐benzyl‐*N*‐phenylpropiolamide (**1a**) (77.4 mg, 0.2 mmol), PdCl_2_ (3.5 mg, 0.02 mmol), PivOH (2.0 mg, 0.02 mmol) and MnO_2_ (86.9 mg, 1.0 mmol). The reaction vial was purged with Ar atmosphere and capped. The reaction mixture was stirred at room temperature for 5 min and then heated at 80°C for 6 h. After cooling to room temperature, the reaction mixture was passed through Celite using CH_2_Cl_2_ as an eluent. The organic layer was washed with water and brine, dried over anhydrous MgSO_4_, filtered, and concentrated under reduced pressure. The crude product was purified by silica gel column chromatography using hexane/CH_2_Cl_2_ (10:1) as eluent to afford the desired product **2a** in 93% yield (71.6 mg, 0.18 mmol) as dark red solid.

## Conflicts of Interest

The author declare no conflicts of interest.

## Supporting information




**Supporting file**: Additional supporting information can be found online in the Supporting Information section. The authors have cited additional references within the Supporting Information. Experimental procedures, characterization data (^1^H, ^13^C, ^19^F NMR, HRMS) for all compounds, and crystallographic data, as well as DFT calculation data are available in the Supporting Information.

## Data Availability

The data that supports the findings of this study are available in the supplementary material of this article.
