# Supplementary material for: One‐Shot Pd(II)‐Catalyzed Multiple C–H Activation Enables Modular Construction of Fluorenylidene Oxindole‐Based Multi(Polycyclic) Aromatic Enes
Source: Chemistry. 2026 Jun 30;32(38):e71221. doi: 10.1002/chem.71221 (PMC13660499; doi:10.1002/chem.71221)
Supplement: Supplementary file 1 [file CHEM-32-e71221-s001.pdf]

Supporting Information

One-Shot Pd(II)-Catalyzed Multiple C–H Activation Enables Modular Construction of Fluorenylidene Oxindole-Based Multi(polycyclic) Aromatic Enes

Haiyu Wang, Kouki Sakata, Takuma, Sato, Masahiro Terada, and Tienan Jin*

Department of Chemistry, Graduate School of Science, Tohoku University, 6-3 Aramaki Azaaoba Aoba-ku, Sendai 980-8578, Japan

E-mail: tetsuo.kin.a6@tohoku.ac.jp

Table of Contents

1. General Information S2
2. General procedures for the synthesis of **2a** S2
3. General procedures for the synthesis of substrates S3
4. Isomerization of unsymmetric (*E*)- and (*Z)*- products S20
5. Kinetic isotope effect (KIE) experiments S21
6. Summary of single crystal **2p** S22
7. UV-vis absorption spectra of compounds **2a**, **2l**, **2m**, **2n** and **8a**-**8c** S24
8. Cyclic voltammetry (CV) of products S26
9. DFT calculations S29
   9.1 DFT calculation data for the reaction mechanism: Path A S29
   9.2 DFT calculation data for the reaction mechanism: Path B S49
   9.3 Path C: Amide-directed *ortho*-C-H activation S69
   9.4 Path D: sequence CMD S74
   9.5 Cartesian coordinates and energies of the DFT-optimized products S77
   9.6 Summary of HOMO and LUMO energies S92
   9.7 TD-DFT excitation energies and oscillator strengths for **2a**, **4b**, **4c**, **4d**, **6a**, and **2p** S92
10. Analytical data of starting substrates and products S94
11. ^{1}H and ^{13}C NMR spectra of starting substrates and products S110
12. References S176

1. General Information

1.1 General Information

^{1}H NMR and ^{13}C NMR spectra were recorded on JEOL JNM AL 400 (400 MHz), JEOL JNM AL 600 (600 MHz), and Bruker AVANCE NEO 700 (700 MHz) spectrometers. ^{1}H NMR spectra are reported as follows: chemical shift in ppm (δ) relative to the chemical shift of $CDCl_3$ at 7.26 ppm, multiplicities (s = singlet, d = doublet, t = triplet, q = quartet, quint = quintet, m = multiplet, bs = broad singlet), and coupling constants (Hz). ^{13}C NMR spectra were recorded on JEOL JNM AL 400 (100 MHz) spectrometer with complete proton decoupling, and chemical shift reported in ppm (δ) relative to the central line for $CDCl_3$ at 77.16 ppm. ^{19}F NMR spectra were recorded on JEOL JNM ECA 400 (376 MHz) spectrometer. High-resolution mass spectra were obtained on a Bruker Daltonics SolariX spectrometer using dithranol (DIT) as an exact matrix and FT-ICR-MS analyzer. UV/Vis absorption spectra were recorded on a JASCO V-650DS spectrometer. Redox potential values are measured by cyclic voltammograms (CV), which are versus $Ag/AgNO_3$ reference electrode; Pt wire as a counter electrode and glassy carbon as a working electrode; 0.1 M $TBAPF_6$ as a supporting electrolyte in dichloromethane; scan rate is 50 mV s^{-1}, and the energy level of Fc/Fc^+ as -4.80 eV. Flash column chromatography was performed on silica gel 60N (spherical, neutral, 40-50 μm; Kanto Chemical Co., Inc.). Analytical thin-layer chromatography (TLC) was performed on 0.2 mm precoated plate Kieselgel 60 F254 (Merck).

1.2 Materials

Unless otherwise noted, materials were purchased from Wako Pure Chemical Industries, LTD., Tokyo Chemical Industry Co., LTD., Kanto Chemical Co., Inc., Aldrich Inc., and other commercial suppliers and were used without purification. Tetrahydrofuran and diethyl ether were supplied from Kanto Chemical Co., Inc. as "Dehydrated solvent system". MnO_2 (activated, ~85%, <10 μm, Cat. No. 217646) and DMAc were purchased from Aldrich Inc. Other solvents were purchased from commercial suppliers as dehydrated solvents, and used under argon or N_2 atmosphere. $CDCl_3$ was purchased from KANTO Chemical Co., Inc. All air- and moisture-sensitive manipulations were performed under argon atmosphere using oven-dried glassware, including standard Schlenk and glovebox techniques. The structure of **2n** was confirmed by means of X-ray crystallography. The structures of starting substrates and products were determined by ^{1}H, ^{13}C NMR, high resolution mass spectrometry (HRMS).

2. General procedure for the synthesis of 2a

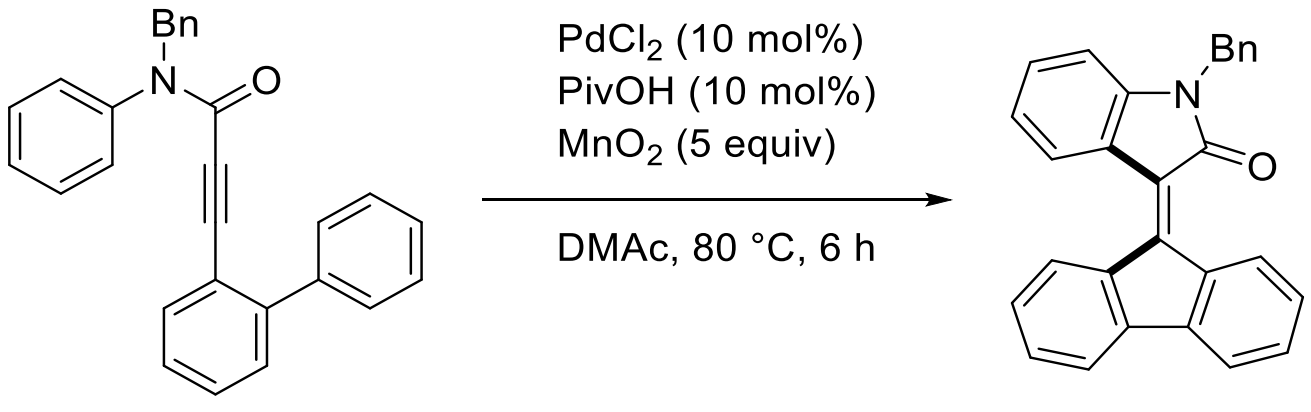

1a **2a**

To an anhydrous DMAc (1.6 mL, 0.125 M) solution were added 3-(biphenyl-2-yl)-*N*-benzyl-*N*-phenylpropiolamide (**1a**) (77.4 mg, 0.2 mmol), $PdCl_2$ (3.5 mg, 0.02 mmol), PivOH (2.0 mg, 0.02

mmol) and MnO_2 (86.9 mg, 1.0 mmol). The reaction vial was purged with Ar atmosphere and capped. The reaction mixture was stirred at room temperature for 5 min and then heated at 80 °C for 6 hours. After cooling to room temperature, the reaction mixture was passed through Celite using CH_2Cl_2 as an eluent. The organic layer was washed with water and brine, dried over anhydrous $MgSO_4$, filtered, and concentrated under reduced pressure. The crude product was purified by column chromatography using hexane/CH_2Cl_2 (10:1) as eluent to afford the desired product **2a** in 93% yield (71.6 mg, 0.18 mmol) as a dark red solid.

3 General procedures for synthesis of substrates

3.1 Synthetic procedure of 2-ethynyl-1,1'-biphenyl

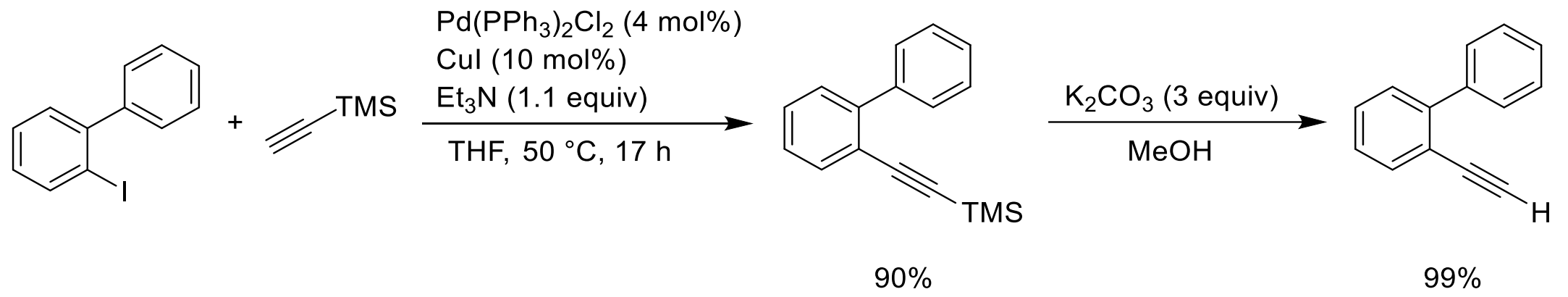

A Schlenk flask equipped with a magnetic stir bar was charged with $Pd(PPh_3)_2Cl_2$ (281 mg, 0.4 mmol) and CuI (190 mg, 1.0 mmol), evacuated and refilled with N_2 three times. Subsequently, 2-iodo-1,1′-biphenyl (2.80 g, 10 mmol), triethylamine (15 mL), dry THF (25 mL), and ethynyltrimethylsilane (1.08 g, 11 mmol) were added sequentially. The reaction mixture was stirred at 50 °C for 24 h. Upon completion, the solvent was removed under reduced pressure, and the crude residue was purified by silica gel column chromatography to afford (biphenyl-2-ylethynyl)trimethylsilane as a yellow oil (2.25 g, 90% yield).

The obtained (biphenyl-2-ylethynyl)trimethylsilane (1.77 g, 9 mmol) was dissolved in MeOH (30 mL), and K_2CO_3 (3.73 g, 27 mmol) was added. The reaction mixture was stirred at room temperature overnight. After completion, the mixture was poured into water (20 mL) and extracted with ethyl acetate (3 × 30 mL), then washed with brine. The combined organic layers were dried over anhydrous Na_2SO_4, filtered, and concentrated under reduced pressure. Purification by flash chromatography afforded 2-ethynyl-1,1′-biphenyl as a yellow oil (1.59 g, 99% yield).

3.2 Synthetic procedure of 3-(biphenyl-2-yl)propioloyl chloride

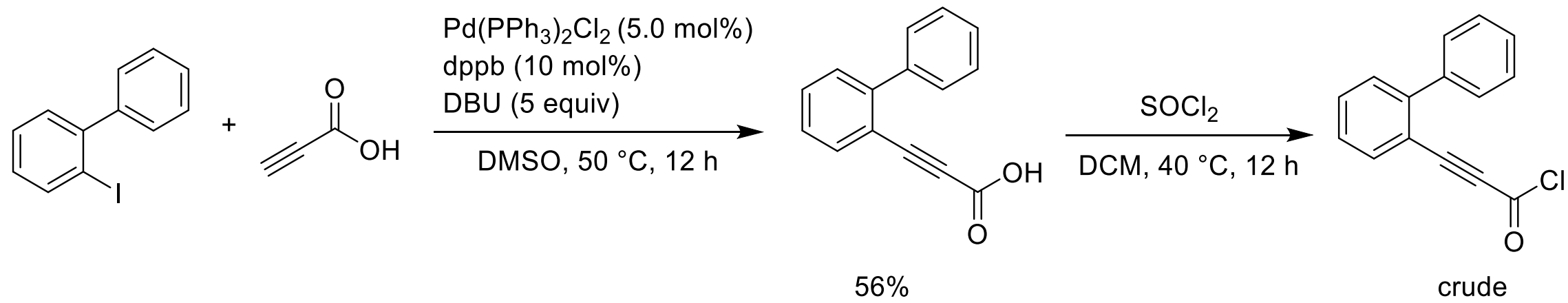

A Schlenk flask equipped with a magnetic stir bar was charged with $Pd(PPh_3)_2Cl_2$ (701.9 mg, 1.0 mmol), dppb (853.0 mg, 2.0 mmol), and 2-iodo-1,1′-biphenyl (5.60 g, 20 mmol), then evacuated and refilled with N_2 three times. DBU (15.2 g, 100 mmol) and dry DMSO (40 mL) were added, followed by addition of propiolic acid (1.4 g, 20 mmol). The reaction mixture was

stirred at 50 °C for 12 h. Upon completion, the mixture was poured into ethyl acetate and washed with saturated $NaHCO_3$ solution. The aqueous layer was acidified to pH 2 using 1 N HCl (aq) at 0 °C and extracted with CH_2Cl_2. The organic layer was dried over $MgSO_4$, filtered, and concentrated under reduced pressure. The crude residue was purified by silica gel chromatography to yield 3-(biphenyl-2-yl)propiolic acid as a yellow solid (2.49 g, 56%).

In a flame-dried two-necked Schlenk flask under N_2 atmosphere, 3-(biphenyl-2-yl)propiolic acid (0.67 g, 3 mmol) was dissolved in dry CH_2Cl_2 (7.5 mL), and $SOCl_2$ (1.43 g, 12 mmol) was added. The reaction mixture was refluxed for 12 h. After cooling, excess $SOCl_2$ was removed under reduced pressure, and the resulting crude 3-(biphenyl-2-yl)propioloyl chloride was used in the next step without further purification.

3.3 General procedure A for synthesis of starting substrates

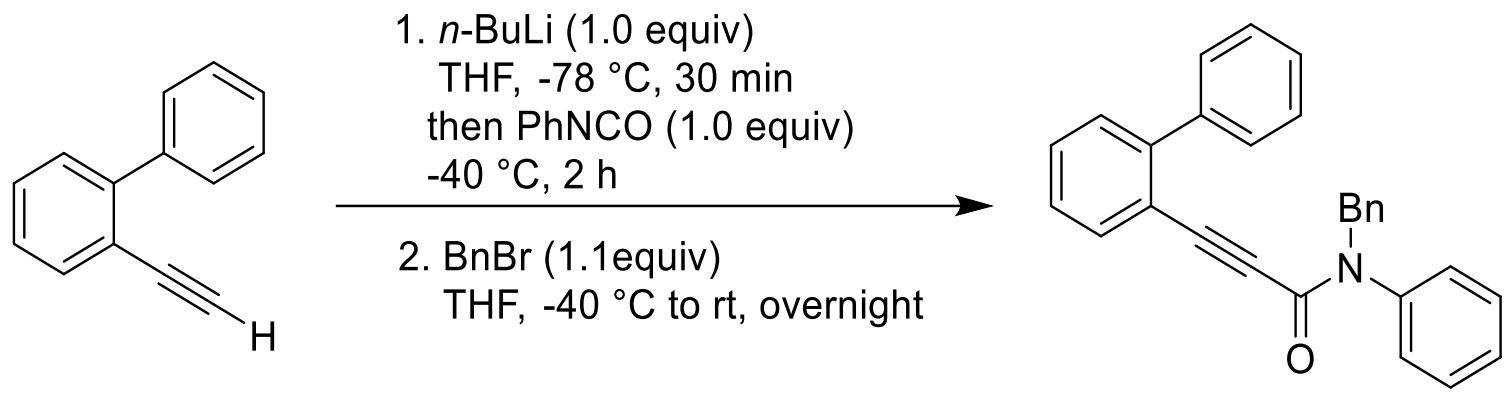

1a, 79%

Following a modified literature procedure,[1] in a flame-dried two-necked Schlenk flask under a N_2 atmosphere, 2-ethynyl-1,1′-biphenyl (0.53 g, 3 mmol) was dissolved in dry THF (6 mL) and cooled to –78 °C. To this solution, *n*-BuLi (1.6 M in hexane, 1.9 mL, 3 mmol) was added dropwise, and the mixture was stirred at –78 °C for 30 min. A solution of phenyl isocyanate (0.36 g, 3 mmol) in THF (1 mL) was then added slowly, and the reaction was stirred at –40 °C for 2 h. Subsequently, a solution of benzyl bromide (0.56 g, 3.3 mmol) in THF (1 mL) was added dropwise. The mixture was gradually warmed to room temperature and stirred overnight. The reaction was quenched with saturated aqueous NH_4Cl, extracted with ethyl acetate (3 × 20 mL) and washed with brine. The combined organic phases were dried over Na_2SO_4, filtered, and concentrated. The crude residue was purified by flash chromatography to afford compound **1a** as a pale solid (0.92 g, 79% yield). If necessary, the product was further purified by gel permeation chromatography (GPC).

3.4 General procedure B for synthesis of starting substrates

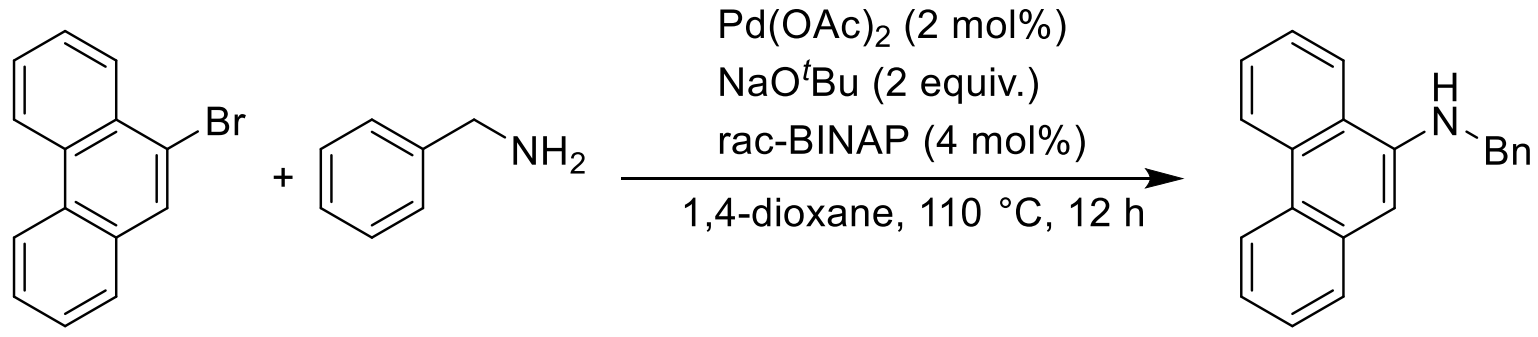

75%

Following a modified literature procedure,[2] a Schlenk flask was charged with $Pd(OAc)_2$ (45 mg, 0.2 mmol), rac-BINAP (249 mg, 0.4 mmol), sodium *tert*-butoxide (1.92 g, 20 mmol), phenylmethanamine (0.93 g, 10 mmol), and 9-bromophenanthrene (2.57 g, 10 mmol) in 1,4-dioxane (10 mL). The flask was evacuated and backfilled with N_2 three times. The reaction

mixture was stirred at 110 °C for 12 h. After cooling to room temperature, the mixture was diluted with CH_2Cl_2 and filtered through a pad of Celite. The filtrate was concentrated, and the residue was purified by silica gel chromatography to afford *N*-benzylphenanthren-9-amine as a white solid (2.12 g, 75%).

3.5 General procedure C for synthesis of starting substrates

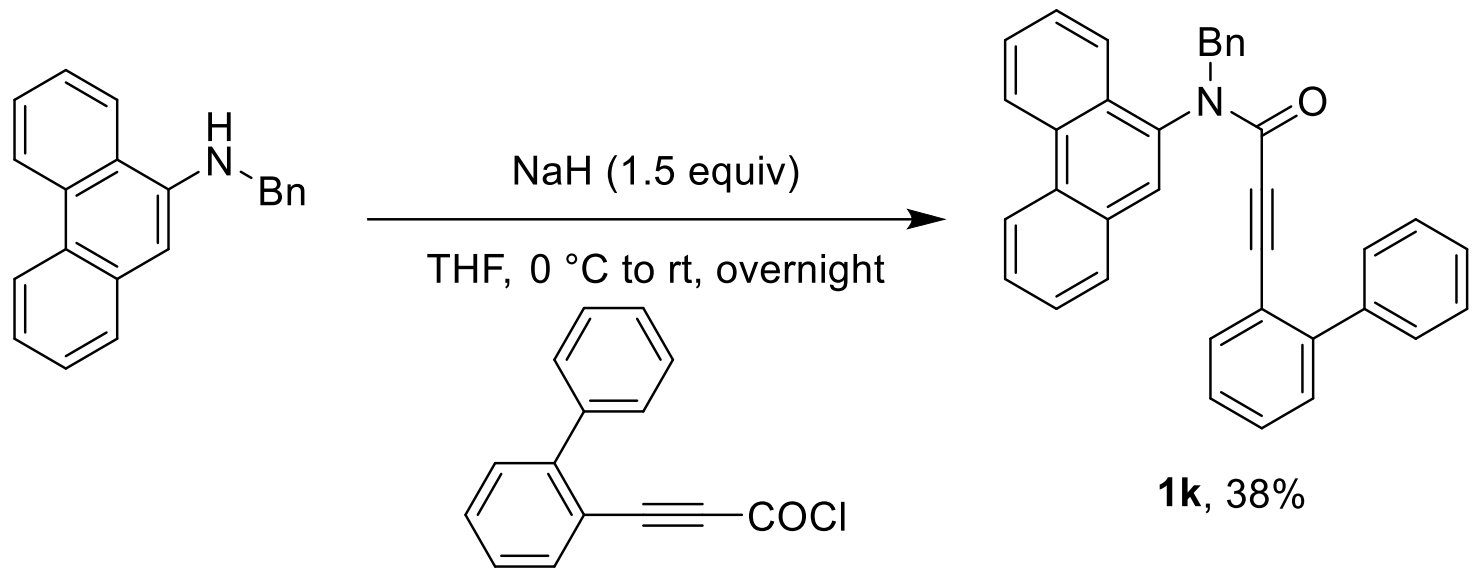

1k, 38%

In a flame-dried two-necked Schlenk flask under a N_2 atmosphere, *N*-benzylphenanthren-9-amine (0.85 g, 3 mmol) was dissolved in dry THF (6 mL) and cooled to 0 °C. NaH (60% dispersion in paraffin oil, 0.18 g, 4.5 mmol) was added portion-wise. After stirring for 30 min at 0 °C, the mixture was warmed to room temperature and stirred for an additional 4 h. A solution of 3-(biphenyl-2-yl)propioloyl chloride (0.72 g, 3 mmol) in THF (2 mL) was added dropwise. The reaction mixture was stirred at room temperature for 12 h. The mixture was then quenched with saturated aqueous NH_4Cl and extracted with ethyl acetate (3 × 10 mL) and washed with brine. The combined organic layers were dried over Na_2SO_4, filtered, and concentrated under reduced pressure. The crude residue was purified by flash chromatography to give compound **1i** as a yellow solid (0.56 g, 38% yield).

3.6 Synthetic procedure of substrate 1b

Compound **1b** was synthesized according to the General Procedure A using methanol instead of benzyl bromide. The final product was obtained as a yellow solid (0.83 g, 93% yield).

3.7 Synthetic procedure of substrate 1c

Compound **1c** was synthesized according to the General Procedure A using acetic anhydride instead of benzyl bromide. The final product was obtained as a yellow solid (0.77 g, 76% yield).

3.8 Synthetic procedure of substrate 1d

Compound **1d** was synthesized according to the General Procedure A using methyl iodide instead of benzyl bromide. The final product was obtained as a yellow liquid (0.61 g, 66% yield).

3.9 Synthetic procedure of substrate 1e

Compound **1e** was synthesized from diphenylamine and 3-(biphenyl-2-yl)propioloyl chloride according to the General Procedure C. The final product was obtained as a yellow solid (0.72 g, 64% yield).

3.10 Synthetic procedure of substrate 1f

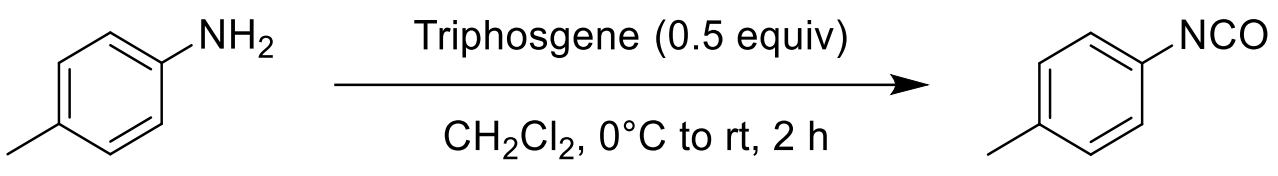

crude

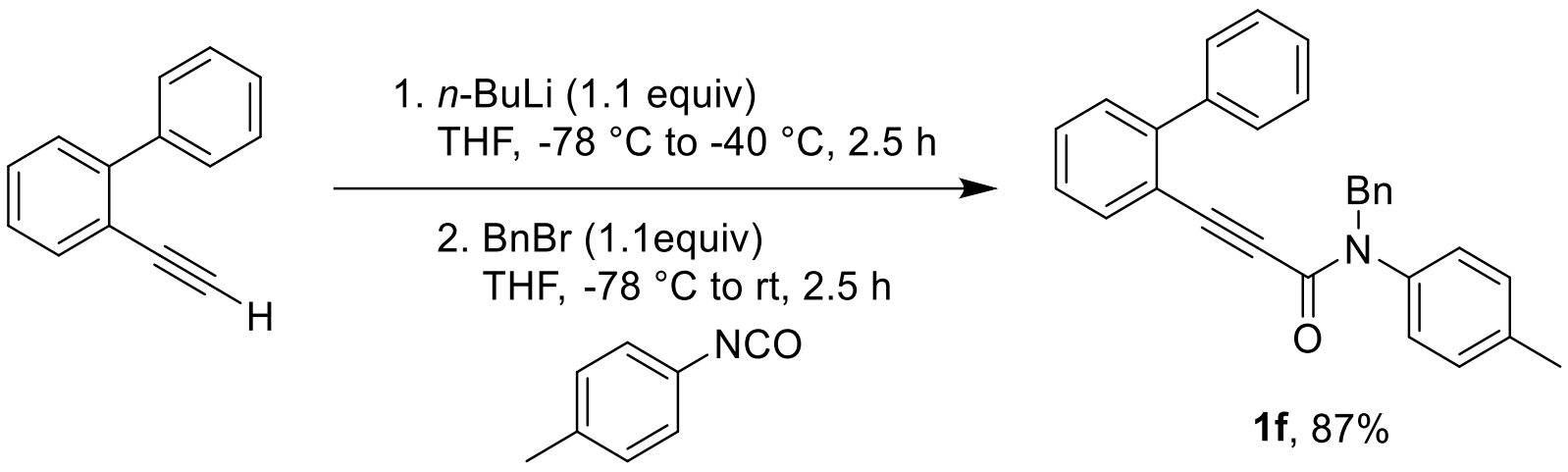

1f, 87%

In a flame-dried two-necked Schlenk flask under a N_2 atmosphere, *p*-toluidine (1.07 g, 10 mmol) was dissolved in dry CH_2Cl_2 (30 mL) and cooled to 0 °C. A solution of triphosgene (1.48 g, 5 mmol) in CH_2Cl_2 (10 mL) was added dropwise, and the mixture was stirred at 0 °C for 5 min. The mixture was then warmed to room temperature and stirred for 2 h. After completion, the reaction was quenched with hexane, and the resulting mixture was filtered through a pad of Celite. The filtrate was concentrated under reduced pressure, and the crude residue was purified by flash chromatography on Florisil using hexane as the eluent to afford 1-isocyanato-4-methylbenzene as a colorless liquid. The obtained isocyanate was used directly in the next step without further purification.

Compound **1f** was synthesized from 1-isocyanato-4-methylbenzene and 2-ethynyl-1,1′-biphenyl according to the General Procedure A. The final product was obtained as a yellow solid (1.05 g, 87% yield).

3.11 Synthetic procedure of substrate 1g

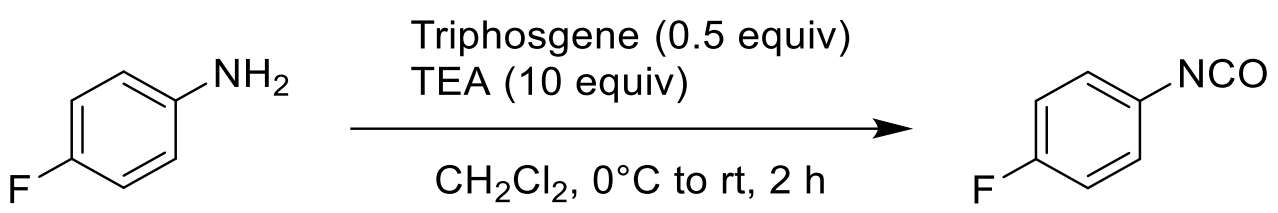

crude

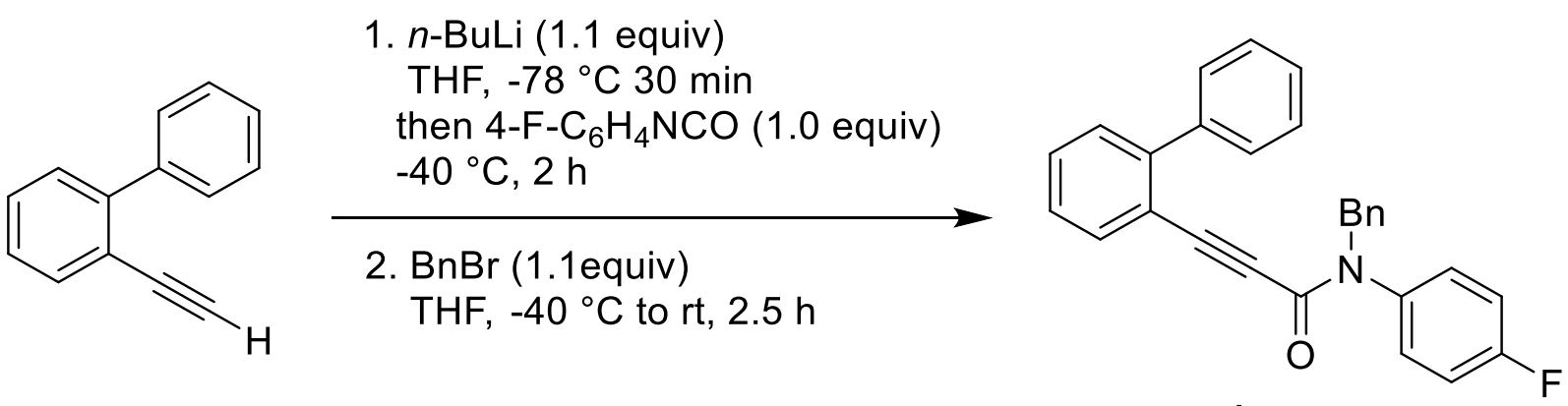

1g, 94%

In a flame-dried two-necked Schlenk flask under a N_2 atmosphere, 4-fluoroaniline (1.11 g, 10 mmol) and triethylamine (10.12 g, 100 mmol) were dissolved in dry CH_2Cl_2 (30 mL) and the solution was cooled to 0 °C. A solution of triphosgene (1.48 g, 5 mmol) in CH_2Cl_2 (10 mL) was added dropwise, and the reaction mixture was stirred at 0 °C for 5 min. The mixture was then allowed to warm to room temperature and stirred for an additional 2 h. After completion, the reaction was quenched with hexane, and the resulting mixture was filtered through a pad of Celite. The filtrate was concentrated under reduced pressure, and the crude residue was purified by flash chromatography on Florisil using hexane as the eluent to afford 1-isocyanato-

4-fluorobenzene as a yellow liquid. The obtained isocyanate was used directly in the next step without further purification.

Compound **1g** was synthesized from 1-isocyanato-4-fluorobenzene and 2-ethynyl-1,1′-biphenyl according to the General Procedure A. The final product was obtained as a yellow solid (1.14 g, 94% yield).

3.12 Synthetic procedure of substrate 1h

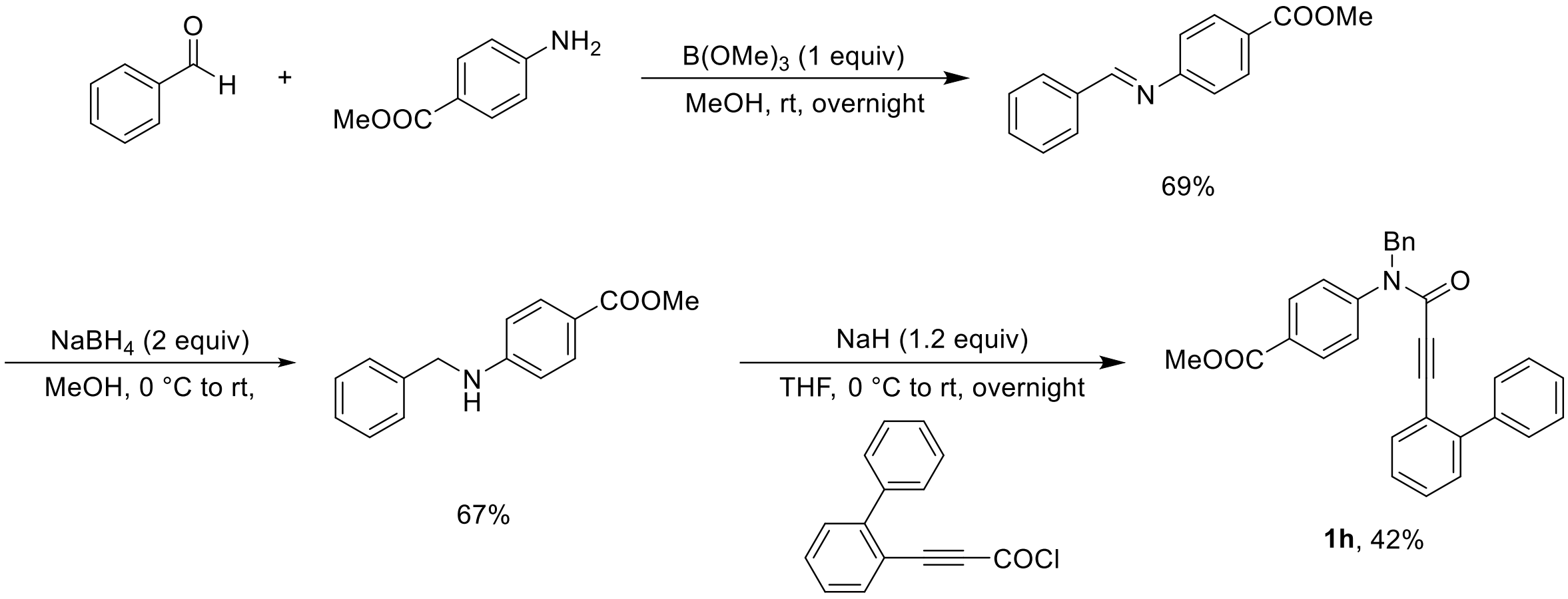

In a flame-dried two-necked Schlenk flask under N_2 atmosphere, methyl 4-aminobenzoate (0.83 g, 5.5 mmol) and benzaldehyde (0.53 g, 5 mmol) were dissolved in MeOH (3 mL), and $B(OMe)_3$ (0.52 g, 5 mmol) was added dropwise. The resulting mixture was stirred at room temperature overnight. Upon completion, the mixture was diluted with CH_2Cl_2 and filtered through a pad of Celite. The filtrate was concentrated under reduced pressure, and the crude residue was purified by recrystallization from toluene/hexane to afford methyl 4-(benzylideneamino)benzoate as a white solid (0.82 g, 69% yield).

The obtained methyl-4-(benzylideneamino)benzoate (0.72 g, 3 mmol) was dissolved in MeOH (7.5 mL), and cooled to 0 °C. $NaBH_4$ (0.23 g, 6 mmol) was carefully added in small portions. The resulting mixture was stirred at room temperature for 6 h. After all the starting materials were consumed, the reaction was quenched with water and extracted with ethyl acetate (3 × 10 mL). The organic layers were washed with brine, dried over anhydrous Na_2SO_4, filtered, and concentrated under reduced pressure. The resulting methyl 4-(benzylamino)benzoate was purified by chromatography on silica gel to afford a white solid (0.48 g, 67% yield).

In a flame-dried two-necked Schlenk flask under a N_2 atmosphere, methyl 4-(benzylamino)benzoate (0.43 g, 1.8 mmol) was dissolved in dry THF (5 mL) and cooled to 0 °C. NaH (60% dispersion in paraffin oil, 86.4 mg, 2.2 mmol) was added portion-wise. After stirring for 1 h at 0 °C, a solution of 3-(biphenyl-2-yl)propioloyl chloride (0.43g, 1.8 mmol) in THF (2 mL) was added dropwise. The reaction mixture was stirred at room temperature for 12 h. The mixture was then quenched with saturated aqueous NH_4Cl and extracted with ethyl acetate (3 × 10 mL) and washed with brine. The combined organic layers were dried over Na_2SO_4, filtered, and concentrated under reduced pressure. The crude residue was purified by flash chromatography to give compound **1h** as a white solid (0.34 g, 42% yield).

3.13 Synthetic procedure of substrate 1i

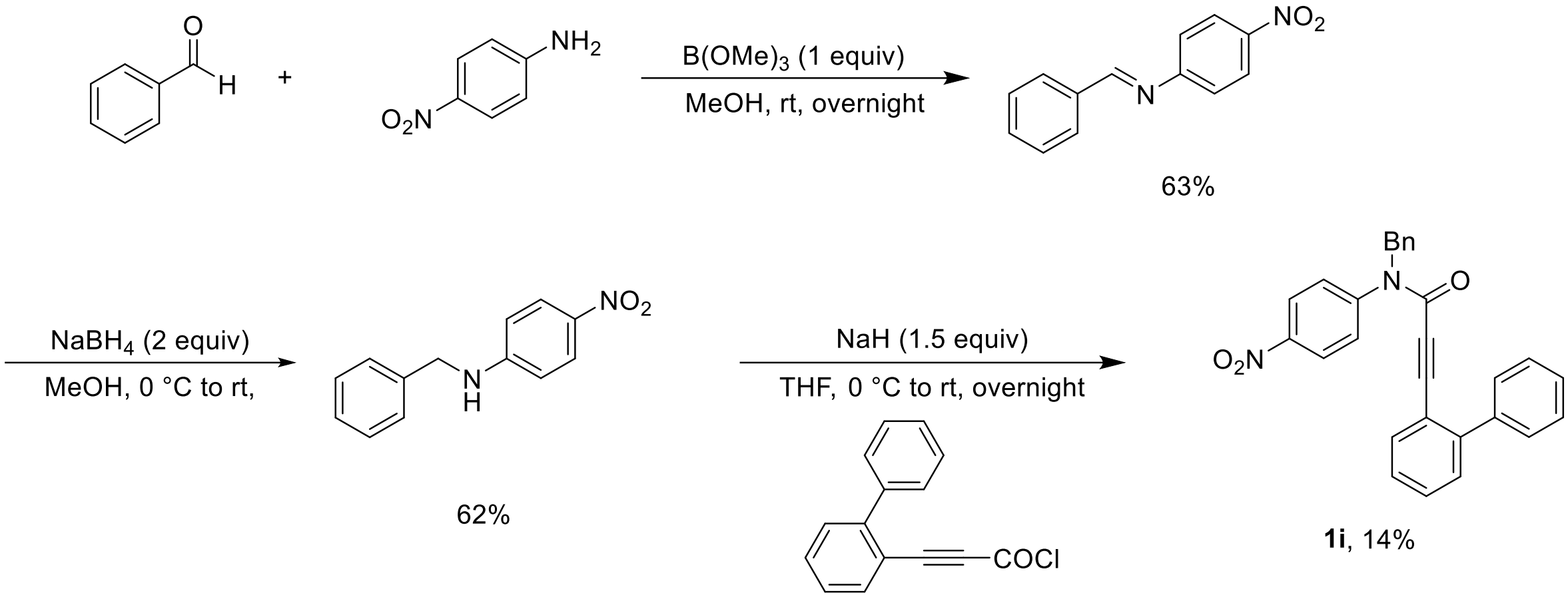

In a flame-dried two-necked Schlenk flask under N_2 atmosphere, 4-nitroaniline (0.76 g, 5.5 mmol) and benzaldehyde (0.53 g, 5 mmol) were dissolved in MeOH (3 mL), and $B(OMe)_3$ (0.52 g, 5 mmol) was added dropwise. The resulting mixture was stirred at room temperature overnight. Upon completion, the mixture was diluted with CH_2Cl_2 and filtered through a pad of Celite. The filtrate was concentrated under reduced pressure, and the crude residue was purified by recrystallization from EtOH to afford *N*-(4-nitrophenyl)-1-phenylmethanimine as a yellow solid (0.71 g, 63% yield).

The obtained N-(4-nitrophenyl)-1-phenylmethanimine (0.68 g, 3 mmol) was dissolved in MeOH (7.5 mL), and cooled to 0 °C. $NaBH_4$ (0.23 g, 6 mmol) was carefully added in small portions. The resulting mixture was stirred at room temperature for 6 h. After all the starting materials were consumed, the reaction was quenched with water and extracted with ethyl acetate (3 × 10 mL). The organic layers were washed with brine, dried over anhydrous Na_2SO_4, filtered, and concentrated under reduced pressure. The resulting N-benzyl-4-nitroaniline was purified by chromatography on silica gel to afford a yellow solid (0.42 g, 62% yield).

In a flame-dried two-necked Schlenk flask under a N_2 atmosphere, N-benzyl-4-nitroaniline (0.41 g, 1.8 mmol) was dissolved in dry THF (5 mL) and cooled to 0 °C. NaH (60% dispersion in paraffin oil, 0.11 g, 2.7 mmol) was added portion-wise. After stirring for 1 h at 0 °C, a solution of 3-(biphenyl-2-yl)propioloyl chloride (0.43g, 1.8 mmol) in THF (2 mL) was added dropwise. The reaction mixture was stirred at room temperature for 12 h. The mixture was then quenched with saturated aqueous NH_4Cl and extracted with ethyl acetate (3 × 10 mL) and washed with brine. The combined organic layers were dried over Na_2SO_4, filtered, and concentrated under reduced pressure. The crude residue was purified by flash chromatography on silica gel to give partially purified compound **1i**. Since compound **1i** showed almost the same Rf value as N-benzyl-4-nitroaniline on TLC, the material was further purified by gel permeation chromatography (GPC) to afford compound **1i** as a white solid (0.11 g, 14% yield).

3.14 Synthetic procedure of substrate 1j

Compound **1j** was synthesized from 1-isocyanatonaphthalene and 2-ethynyl-1,1′-biphenyl according to the General Procedure A. The final product was obtained as a yellow solid (0.90

g, 69% yield).

3.15 Synthetic procedure of substrate 1k

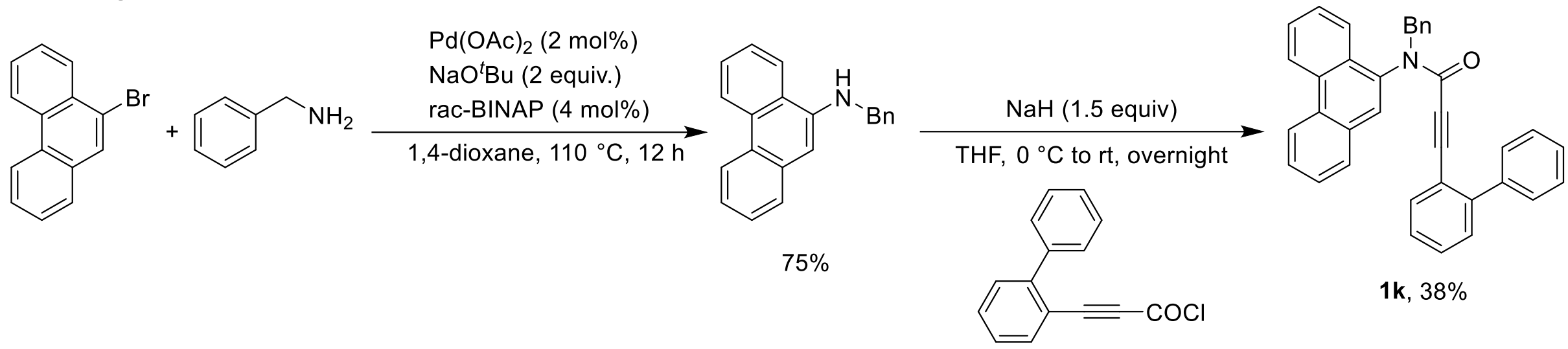

N-benzylphenanthren-9-amine was synthesized from phenylmethanamine and 9-bromophenanthrene according to the General Procedure B. The product was obtained as a white solid (2.12 g, 75%).

Compound **1k** was synthesized from *N*-benzylphenanthren-9-amine and 3-(biphenyl-2-yl)propioloyl chloride according to the General Procedure C. The final product was obtained as a yellow solid (0.56 g, 38% yield).

3.16 Synthetic procedure of starting substrate 1l

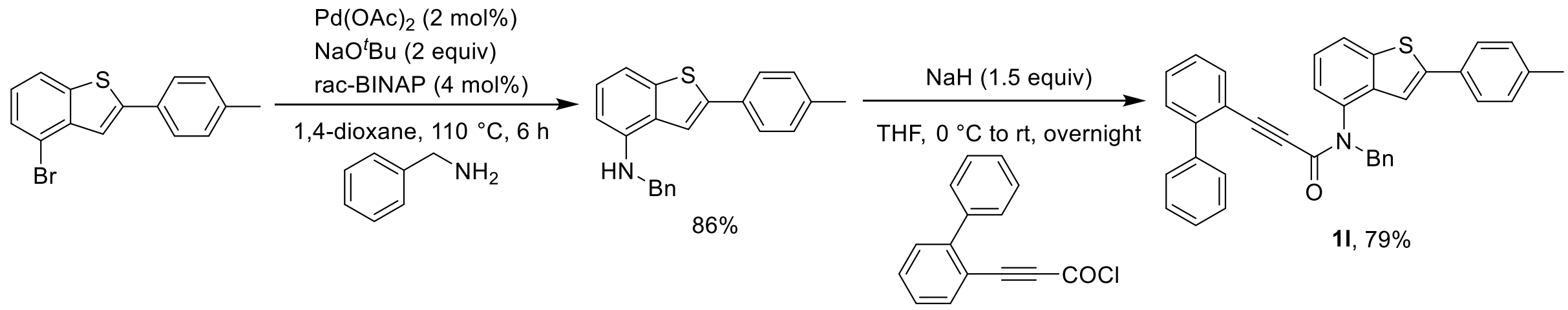

The known compound 4-bromo-2-(*p*-tolyl)benzo[*b*]thiophene was prepared from 4-bromobenzo[*b*]thiophene and 1-iodo-4-methylbenzene according to literature procedures.[3]
N-benzyl-2-(*p*-tolyl)benzo[*b*]thiophen-4-amine was synthesized from 4-bromo-2-(*p*-tolyl)benzo[b]thiophene and benzylamine according to the General Procedure B. The product was obtained as a white solid (2.83 g, 86%).

Compound **1l** was synthesized from *N*-benzyl-2-(*p*-tolyl)benzo[*b*]thiophen-4-amine and 3-(biphenyl-2-yl)propioloyl chloride according to the General Procedure C. The final product was obtained as a yellow solid (1.26 g, 79% yield).

3.17 Synthetic procedure of starting substrate 1m

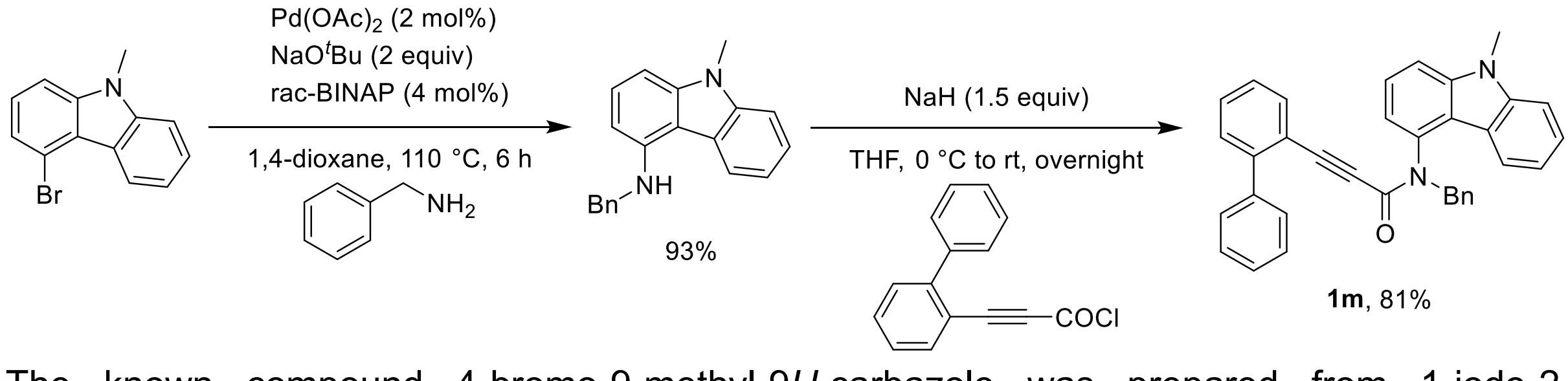

The known compound 4-bromo-9-methyl-9*H*-carbazole was prepared from 1-iodo-2-nitrobenzene and (2-bromophenyl) boronic acid according to literature procedures.[4]

N-benzyl-9-methyl-9H-carbazol-4-amine was synthesized from 4-bromo-9-methyl-9*H*-

carbazole and benzylamine according to the General Procedure B. The product was obtained as a white solid (2.66 g, 93%).

Compound **1m** was synthesized from *N*-benzyl-9-methyl-9*H*-carbazol-4-amine and 3-(biphenyl-2-yl)propioloyl chloride according to the General Procedure C. The final product was obtained as a yellow solid (1.19 g, 81% yield).

3.18 Synthetic procedure of starting substrate 1n

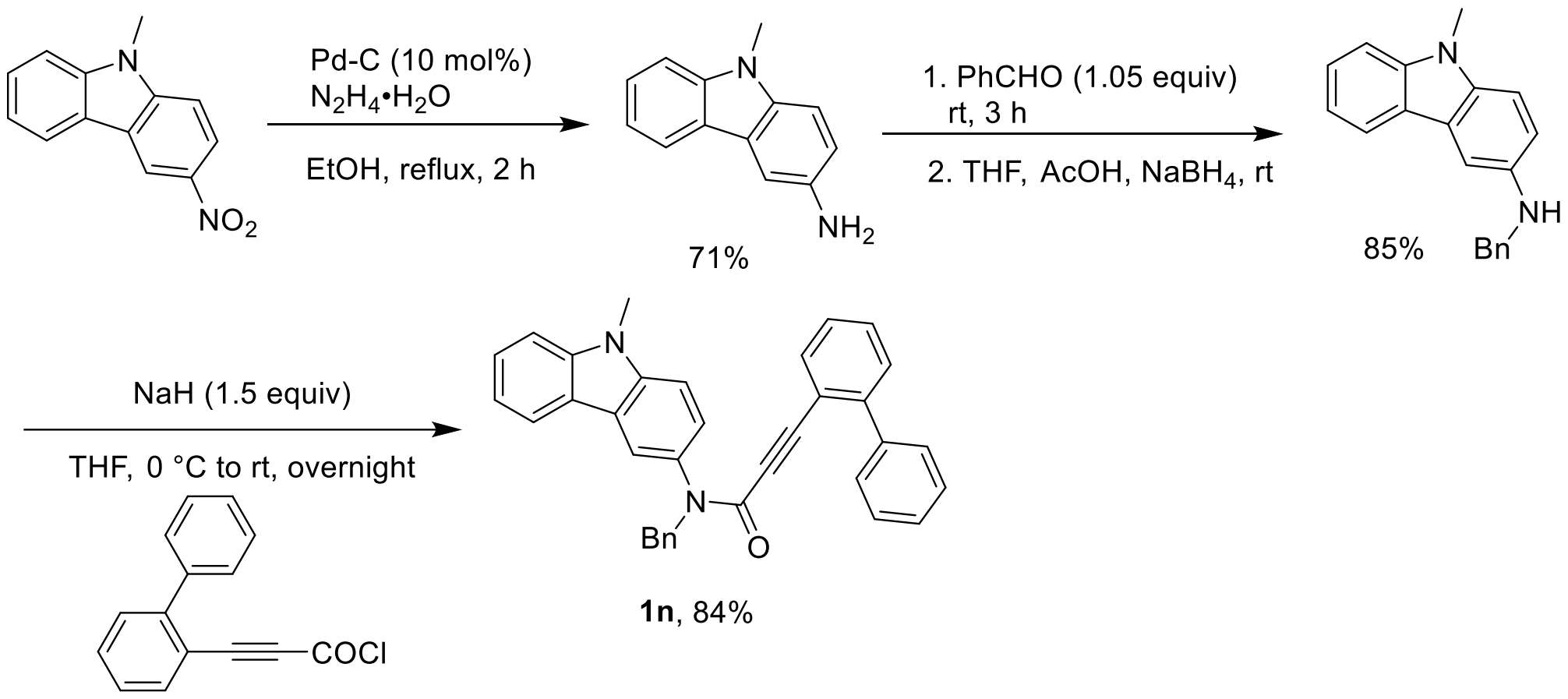

The known compound 9-methyl-3-nitro-9*H*-carbazole was prepared from carbazole according to literature procedures.[5]

In a flame-dried two-necked flask, 9-methyl-3-nitro-9H-carbazole (2.26 g, 10 mmol) and Pd/C (10%, 0.85 g, 0.8 mmol) were added, then evacuated and backfilled with N_2 three times. EtOH (40 mL) was added, and $N_2H_4 \cdot H_2O$ (3.6 mL) was added dropwise. The reaction mixture was refluxed for 2 h, then filtered through a pad of Celite and poured into water. The mixture was extracted with ethyl acetate (3 × 30 mL) and washed with brine. The combined organic layers were dried over anhydrous Na_2SO_4, filtered, and concentrated under reduced pressure. The crude product was purified by flash chromatography on silica gel to afford 9-methyl-9*H*-carbazol-3-amine as a yellow solid (1.39 g, 71%).

The obtained 9-methyl-9*H*-carbazol-3-amine (0.98 g, 5 mmol) and benzaldehyde (0.56 g, 5.25 mmol) were dissolved in MeOH (5 mL), and 3 drops of AcOH were added. The resulting mixture was stirred at room temperature for 3 h. After all the starting materials were consumed, 10 mL of dry THF was added, and $NaBH_4$ (0.21 g, 5.5 mmol) was carefully added in small portions. After gas evolution ceased, the reaction was quenched with water and extracted with ethyl acetate (3 × 20 mL). The organic layers were washed with brine, dried over anhydrous Na_2SO_4, filtered, and concentrated under reduced pressure. The resulting N-benzyl-9-methyl-9H-carbazol-3-amine was purified by flash chromatography on silica gel to afford a brown solid (1.22 g, 85% yield).

Compound **1n** was synthesized from *N*-benzyl-9-methyl-9*H*-carbazol-3-amine and 3-([1,1'-biphenyl]-2-yl)propioloyl chloride according to the General Procedure C. The final product was obtained as a yellow solid (1.23 g, 84% yield).

3.19 Synthetic procedure of starting substrate 1o

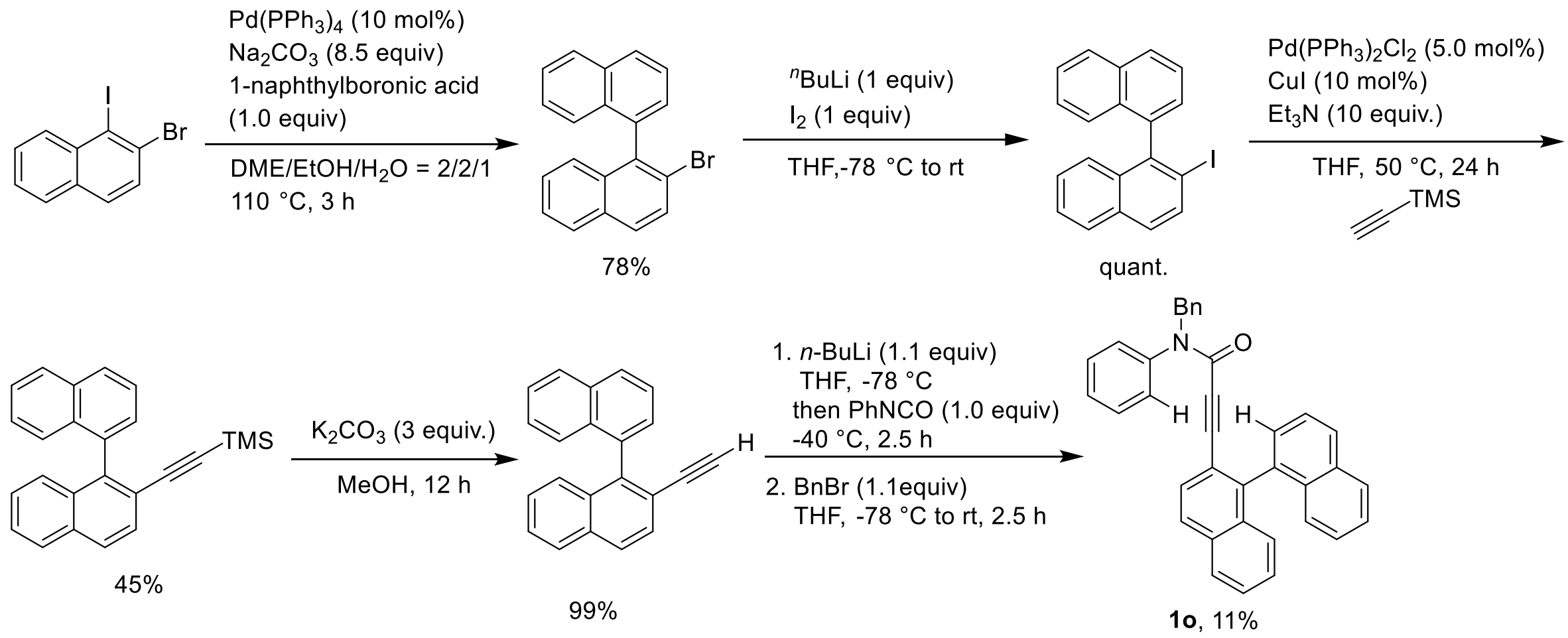

The known compound 2-bromo-1-iodonaphthalene was synthesized from naphthalen-1-amine following literature procedures.[6]

A Schlenk flask equipped with a magnetic stir bar was charged with 2-bromo-1-iodonaphthalene (3.32 g, 10 mmol), 1-naphthylboronic acid (1.72 g, 10 mmol), $Pd(PPh_3)_4$ (1.16 g, 1.0 mmol), and Na_2CO_3 (9.0 g, 85 mmol), then purged with N_2 three times. DME (100 mL), EtOH (50 mL), and H_2O (50 mL) were added sequentially. The reaction mixture was stirred at 110 °C for 3 h. Upon completion, the mixture was extracted with ethyl acetate (3 × 60 mL) and washed with brine. The combined organic layers were dried over anhydrous Na_2SO_4, filtered, and concentrated under reduced pressure. The crude product was purified by flash chromatography on silica gel to afford 2-bromo-1,1′-binaphthalene as a yellow solid (2.58 g, 78%).

In a flame-dried two-necked Schlenk flask under a N_2 atmosphere, 2-bromo-1,1′-binaphthalene (2.32 g, 7.0 mmol) was dissolved in dry THF (70 mL) and cooled to –78 °C. n-BuLi (1.6 M in hexane, 4.38 mL, 7.0 mmol) was added dropwise over 30 min. The reaction mixture was stirred at –78 °C for 1 h, followed by the slow addition of I_2 (1.78 g, 7.0 mmol) dissolved in THF (7.0 mL). The mixture was allowed to warm to room temperature and stirred for 2 h. The reaction was quenched with saturated $Na_2S_2O_3$ solution, extracted with ethyl acetate (3 × 40 mL), then washed with brine. The combined organic phases were dried over Na_2SO_4, filtered, and concentrated. The resulting 2-iodo-1,1′-binaphthalene was used directly in the next step without further purification.

A Schlenk flask equipped with a magnetic stir bar was charged with $Pd(PPh_3)_2Cl_2$ (245 mg, 0.35 mmol) and CuI (133 mg, 0.70 mmol), evacuated and refilled with N_2 three times. Subsequently, 2-iodo-1,1′-binaphthalene (2.88 g, 7.6 mmol), triethylamine (12 mL), dry THF (20 mL), and ethynyltrimethylsilane (0.82 g, 8.4 mmol) were added sequentially. The reaction mixture was stirred at 50 °C for 24 h. Upon completion, the solvent was removed under reduced pressure, and the crude residue was purified by silica gel column chromatography to afford ([1,1'-binaphthalen]-2-ylethynyl)trimethylsilane as a yellow oil (1.20 g, 45% yield).

The obtained ([1,1'-binaphthalen]-2-ylethynyl)trimethylsilane (1.20 g, 3.4 mmol) was dissolved in MeOH (12 mL), and K_2CO_3 (1.42 g, 10.2 mmol) was added. The reaction mixture was stirred at room temperature overnight. After completion, the mixture was poured into water

(20 mL) and extracted with ethyl acetate (3 × 20 mL), then washed with brine. The combined organic layers were dried over anhydrous Na_2SO_4, filtered, and concentrated under reduced pressure. Purification by flash chromatography afforded 2-ethynyl-1,1'-binaphthalene as a colorless oil (0.94 g, 99% yield).

Compound **1o** was synthesized from 2-ethynyl-1,1'-binaphthalene and phenyl isocyanate according to the General Procedure A. The final product was obtained as a yellow solid (0.18 g, 11% yield).

3.20 Synthetic procedure of starting substrate 1p

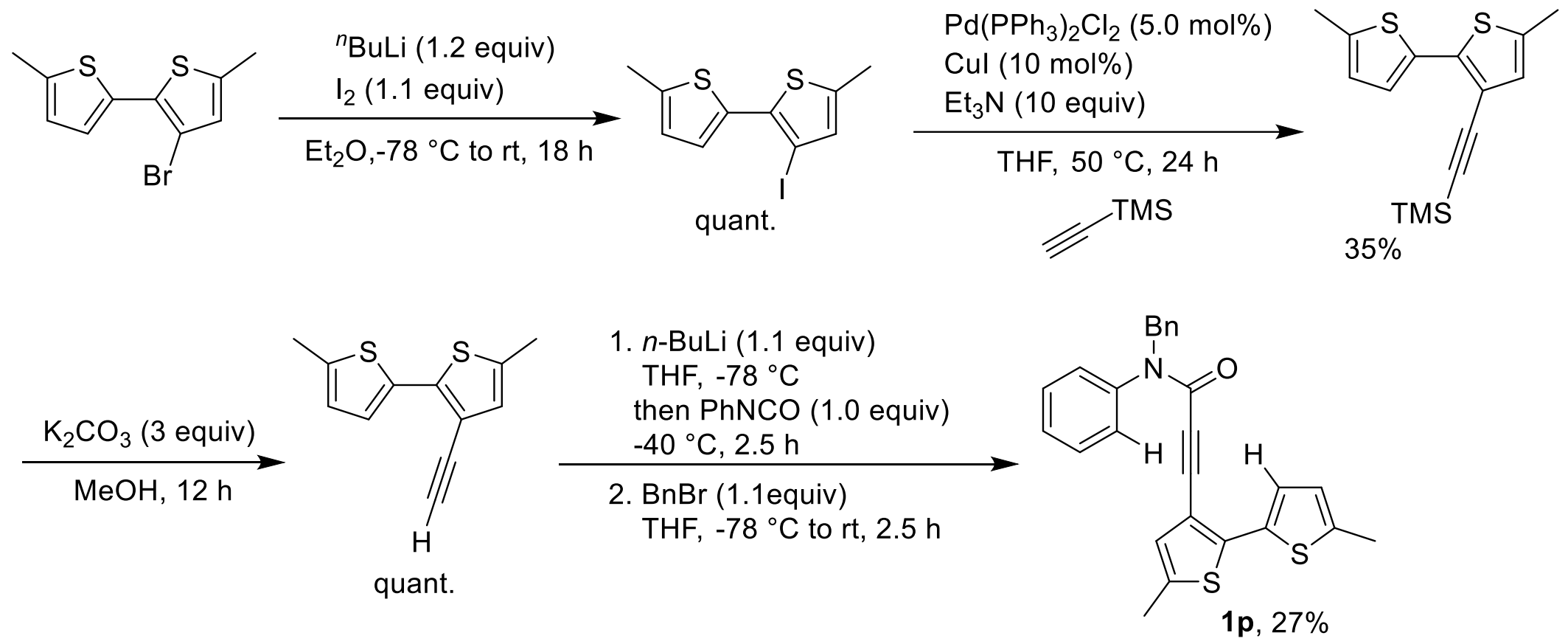

The known compound 3-bromo-5,5′-dimethyl-2,2′-bithiophene was prepared from 2-methylthiophene according to literature procedures.[7]

In a flame-dried two-necked Schlenk flask under N_2 atmosphere, 3-bromo-5,5′-dimethyl-2,2′-bithiophene (1.36 g, 5.0 mmol) was dissolved in dry Et_2O (33 mL) and cooled to –78 °C. n-BuLi (1.6 M in hexane, 3.75 mL, 6.0 mmol) was added dropwise over 5 min. After stirring at –78 °C for 30 min, a solution of I_2 (1.40 g, 5.5 mmol) in dry Et_2O (11 mL) was added quickly. The reaction mixture was stirred at –78 °C for 1 h, then allowed to warm to room temperature and stirred for an additional 18 h. The reaction was quenched with saturated aqueous NH_4Cl, followed by $Na_2S_2O_3$ solution, and extracted with ethyl acetate (3 × 20 mL). The organic layers were washed with brine, dried over anhydrous Na_2SO_4, filtered, and concentrated under reduced pressure. The resulting 3-iodo-5,5′-dimethyl-2,2′-bithiophene was used in the next step without further purification.

Compound **1p** was synthesized from 3-iodo-5,5′-dimethyl-2,2′-bithiophene and 2-ethynyl-1,1′-biphenyl according to the General Procedure A. The final product was obtained as a yellow solid (0.35 g, 27% yield).

3.21 Synthetic procedure of starting substrate 1q

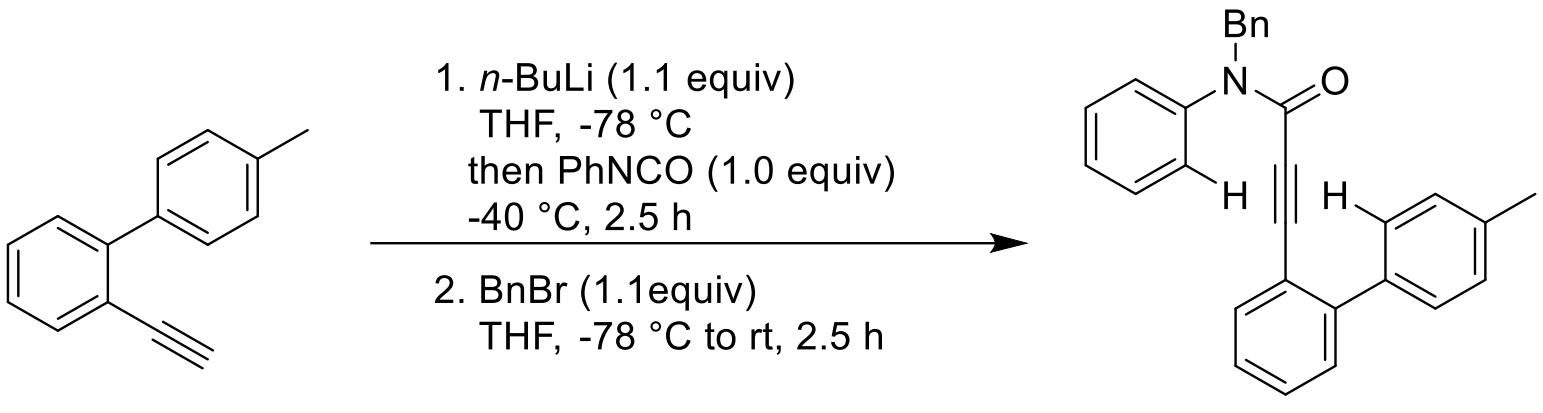

1q, 51%

The known compound 2-ethynyl-4'-methyl-1,1'-biphenyl was prepared from 1-bromo-2-iodobenzene according to a literature procedure.[8]

Compound **1q** was synthesized from phenyl isocyanate and 2-ethynyl-4'-methyl-1,1'-biphenyl according to the General Procedure A. The final product was obtained as a yellow solid (0.62 g, 51% yield).

3.22 Synthetic procedure of starting substrate 1a-*d*

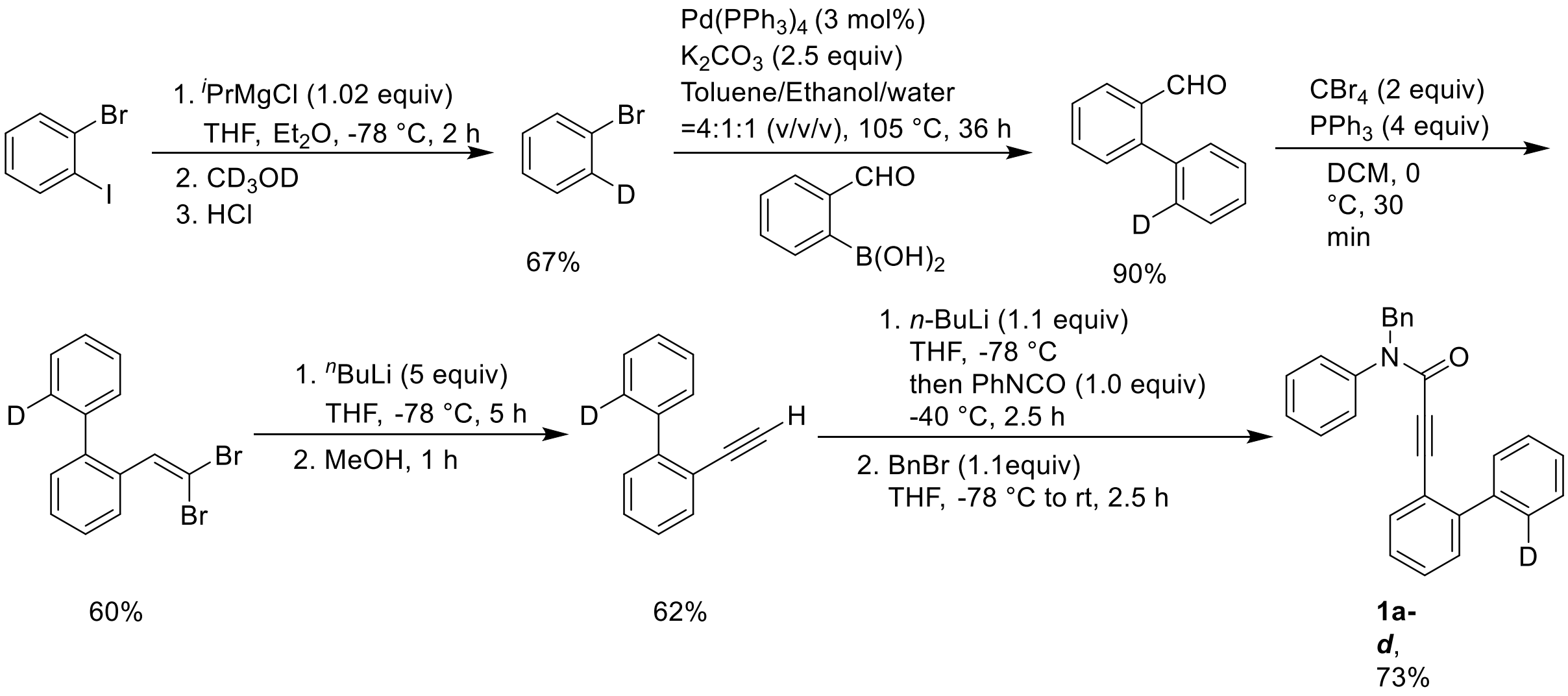

The known compound 1-bromobenzene-2-*d* was prepared from 1-bromo-2-iodobenzene according to a literature procedure.[9]

A Schlenk flask equipped with a magnetic stir bar was charged with (2-formylphenyl)boronic acid (2.10 g, 14 mmol), $Pd(PPh_3)_4$ (486 mg, 0.42 mmol), and K_2CO_3 (0.484 g, 35 mmol), then purged with N_2 three times. Toluene (40 mL), EtOH (10 mL), H_2O (10 mL), and 1-bromobenzene-2-*d* (2.10 g, 13.4 mmol) were added sequentially. The reaction mixture was stirred at 105 °C for 36 h. Upon completion, the mixture was extracted with ethyl acetate (3 × 60 mL) and washed with brine. The combined organic layers were dried over anhydrous Na_2SO_4, filtered, and concentrated under reduced pressure. The crude product was purified by flash chromatography on silica gel to afford [1,1'-biphenyl]-2'-*d*-2-carbaldehyde as a yellow solid (2.20 g, 90% yield).

PPh_3 (10.88 g, 41.5 mmol) was dissolved in CH_2Cl_2 (120 mL) and cooled to 0 °C, followed by the addition of CBr_4 (6.89 g, 20 mmol). The resulting mixture was stirred at this temperature for 10 min. A solution of [1,1'-biphenyl]-2'-*d*-2-carbaldehyde (1.52 g, 8.3 mmol) in CH_2Cl_2 (80 mL) was then added dropwise. The reaction was stirred at 0 °C for 1 h and subsequently

quenched with brine. The mixture was extracted with ethyl acetate (3 × 20 mL) and washed with brine. The combined organic layers were dried over Na_2SO_4, filtered, and concentrated under reduced pressure. The crude product was purified by flash chromatography on silica gel to afford 2-(2,2-dibromovinyl)-1,1'-biphenyl-2'-*d* as a yellow solid (1.68 g, 60% yield).

In a flame-dried two-necked Schlenk flask under N_2 atmosphere, 2-(2,2-dibromovinyl)-1,1'-biphenyl-2'-*d* (1.35 g, 4 mmol) was dissolved in dry THF (10 mL) and cooled to –78 °C. *n*-BuLi (1.6 M in hexane, 12.5 mL, 20 mmol) was added dropwise. The reaction mixture was stirred at –78 °C for 5 h, followed by the addition of MeOH (4 mL). The mixture was stirred for an additional 1 h, allowed to warm to 0 °C, and then quenched with saturated NH_4Cl solution. The mixture was extracted with ethyl acetate (3 × 20 mL) and washed with brine. The combined organic layers were dried over Na_2SO_4, filtered, and concentrated under reduced pressure. The crude product was purified by flash chromatography on silica gel to afford 2-ethynyl-1,1'-biphenyl-2'-*d* as a yellow oil (0.44 g, 62% yield).

Compound **1a-*d*** was synthesized from phenyl isocyanate and 2-ethynyl-1,1'-biphenyl-2'-*d* according to the General Procedure A. The final product was obtained as a yellow solid (0.85 g, 73% yield).

3.23 Synthetic procedure of starting substrate 1a'-*d*

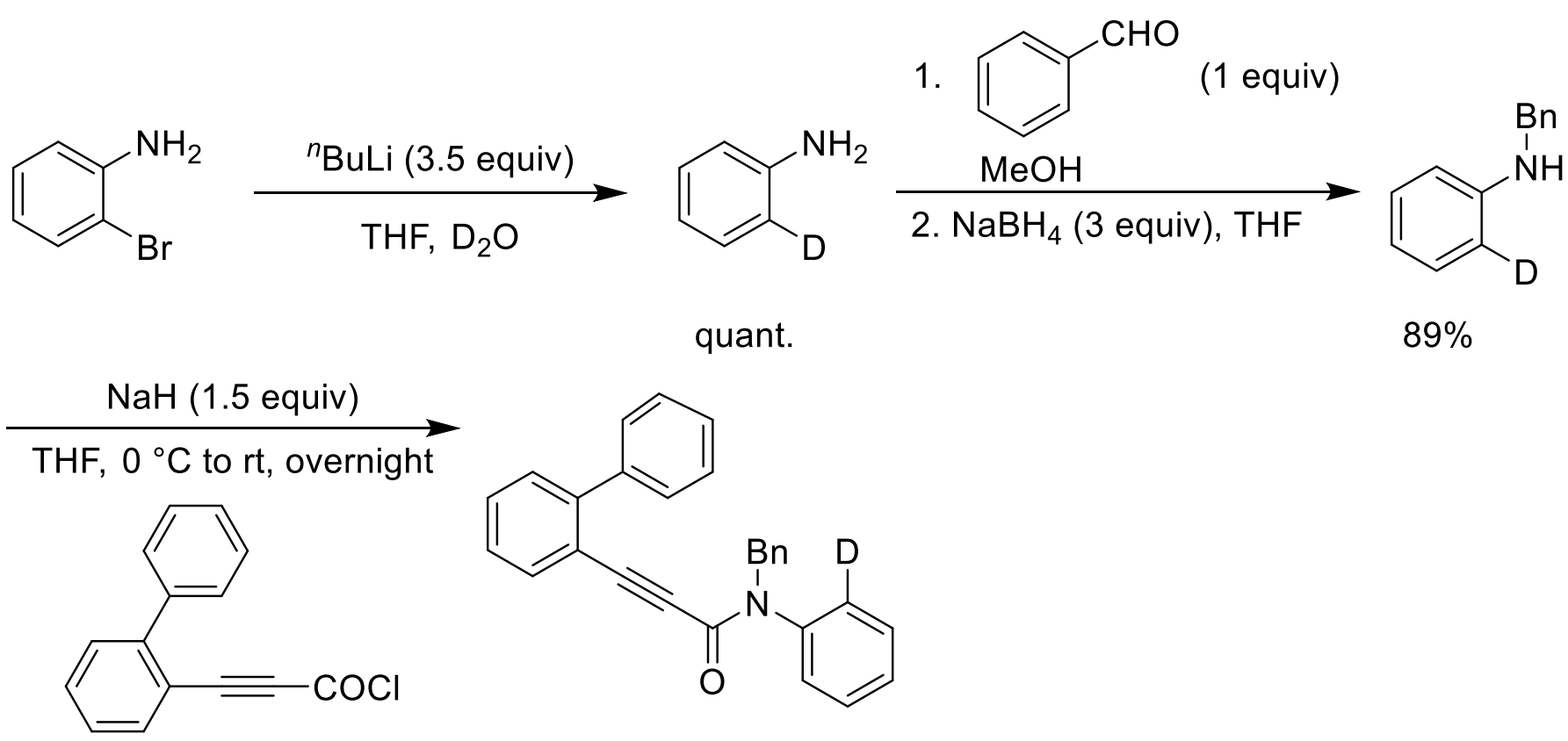

1a'-*d*, 79%

The known compound benzen-2-*d*-amine was prepared from 2-bromoaniline according to literature procedure. [10]

The obtained benzen-2-*d*-amine (0.32 g, 3.5 mmol) was dissolved in MeOH (20 mL), and benzaldehyde (0.37 g, 3.5 mmol) was added dropwise. The reaction mixture was stirred at room temperature for 12 h. The resulting mixture was then cooled to 0 °C, and $NaBH_4$ (0.26 g, 7 mmol) was carefully added in small portions. The reaction mixture was stirred at room temperature for an additional 12 h. The reaction was quenched with water and extracted with ethyl acetate (3 × 20 mL). The organic layers were washed with brine, dried over anhydrous Na_2SO_4, filtered, and concentrated under reduced pressure. The resulting *N*-benzylbenzen-2-d-amine was purified by flash chromatography on silica gel to afford a yellow solid (0.82 g, 89% yield).

Compound **1a'-*d*** was synthesized from *N*-benzylbenzen-2-*d*-amine and 3-(biphenyl-2-yl)propioloyl chloride according to the General Procedure C. The final product was obtained

as a yellow solid (0.92 g, 79% yield).

3.24 Synthetic procedure of starting substrate 1a-d_5

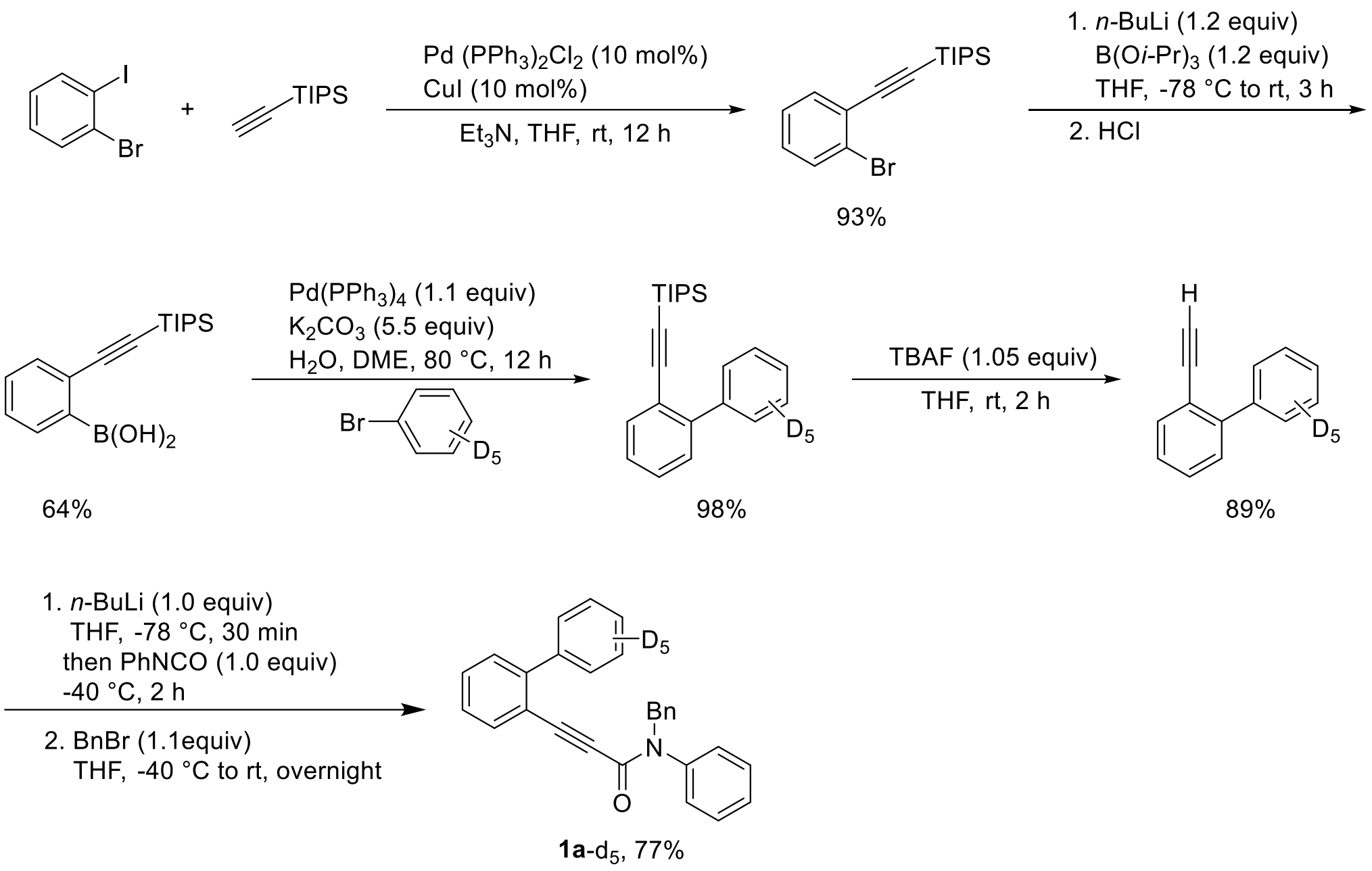

The known compound (2-((triisopropylsilyl)ethynyl)phenyl)boronic acid was prepared from 1-bromo-2-iodobenzene according to literature procedures.[11]

A Schlenk flask equipped with a magnetic stir bar was charged with (2-((triisopropylsilyl)ethynyl)phenyl)boronic acid (1.00 g, 3.3 mmol), $Pd(PPh_3)_4$ (105 mg, 0.15 mmol), and K_2CO_3 (1.04 g, 7.5 mmol), then purged with N_2 three times. DME (7.5 mL), H_2O (7.5 mL), and bromobenzene-d_5 (0.49 g, 3 mmol) were added sequentially. The reaction mixture was stirred at 80 °C for 12 h. Upon completion, the mixture was extracted with ethyl acetate (3 × 10 mL) and washed with brine. The combined organic layers were dried over anhydrous Na_2SO_4, filtered, and concentrated under reduced pressure. The crude product was purified by flash chromatography on silica gel to afford (([1,1'-biphenyl]-2-yl-2',3',4',5',6'-d_5)ethynyl)triisopropylsilane as a colorless oil (1.00 g, 98% yield).

In a flame-dried two-necked Schlenk flask under N_2 atmosphere, ((biphenyl-2-yl-2',3',4',5',6'-d_5)ethynyl)triisopropylsilane (1.00 g, 3 mmol) was dissolved in dry THF (30 mL) and cooled to 0 °C. TBAF (1.0 M in THF, 3.2 mL, 3.2 mmol) was added dropwise and the reaction mixture was warmed to room temperature and stirred for 2 h. The reaction was quenched with water and extracted with CH_2Cl_2 (3 × 10 mL), then washed with brine. The combined organic layers were dried over Na_2SO_4, filtered, and concentrated under reduced pressure. The crude product was purified by chromatography on silica gel to afford 2-ethynyl-biphenyl-2',3',4',5',6'-d_5 as a colorless oil (0.48 g, 89% yield).

Compound **1a**-d_5 was synthesized from -ethynyl-biphenyl-2',3',4',5',6'-d_5 and phenyl isocyanate according to the General Procedure A. The final product was obtained as a yellow solid (0.78 g, 77% yield).

3.25 Synthetic procedure of starting substrate 1a'-d_5

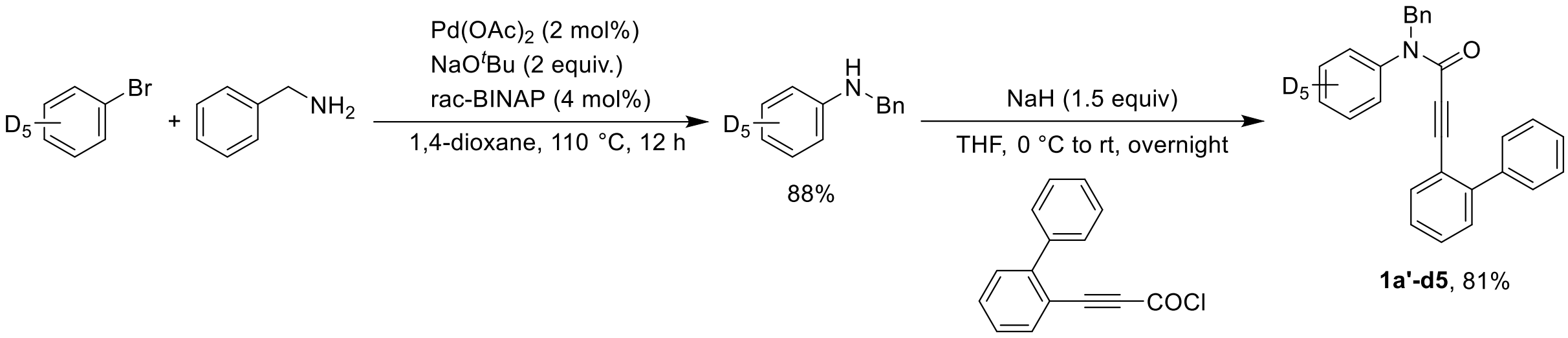

N-benzylbenzen-d5-amine was synthesized from phenylmethanamine and bromobenzene-d_5 according to the General Procedure B. The product was obtained as a white solid (0.48 g, 85%).

Compound **1a'**-d_5 was synthesized from N-benzylbenzen-d_5-amine and 3-(biphenyl-2-yl)propioloyl chloride according to the General Procedure C. The final product was obtained as a yellow solid (0.81 g, 81% yield).

3.26 Synthetic procedure of starting substrate 3a

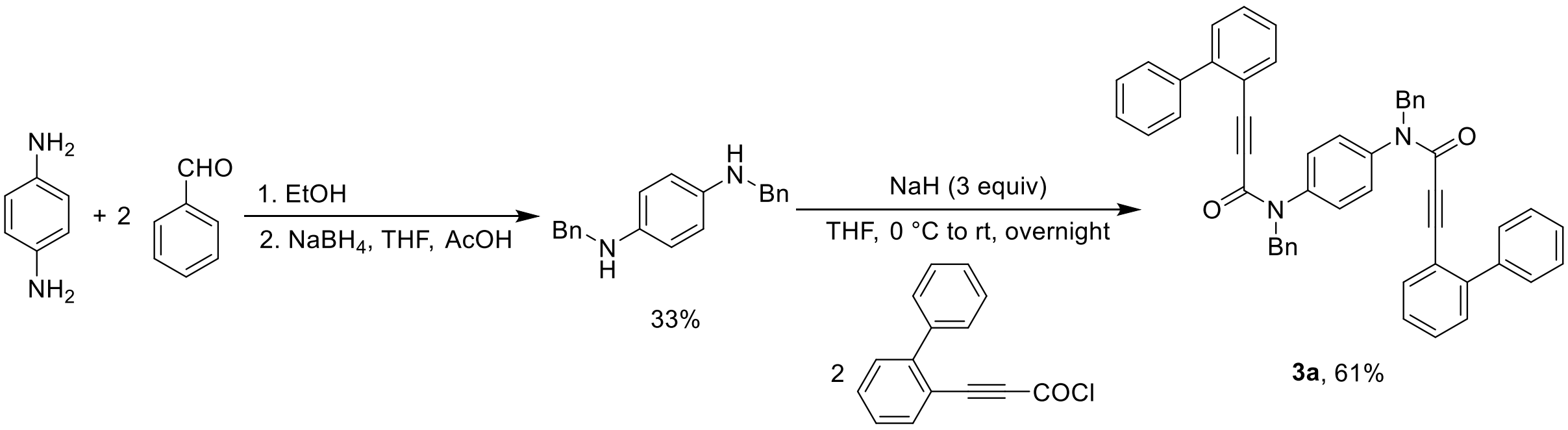

The known compound N^1, N^4-dibenzylbenzene-1,4-diamine was prepared from benzene-1,4-diamine and benzaldehyde according to literature procedures.[12]

Compound **3a** was synthesized from N^1, N^4-dibenzylbenzene-1,4-diamine and 3-(biphenyl-2-yl)propioloyl chloride according to the General Procedure C. The final product was obtained as a yellow solid (1.27 g, 61% yield).

3.27 Synthetic procedure of starting substrate 3b

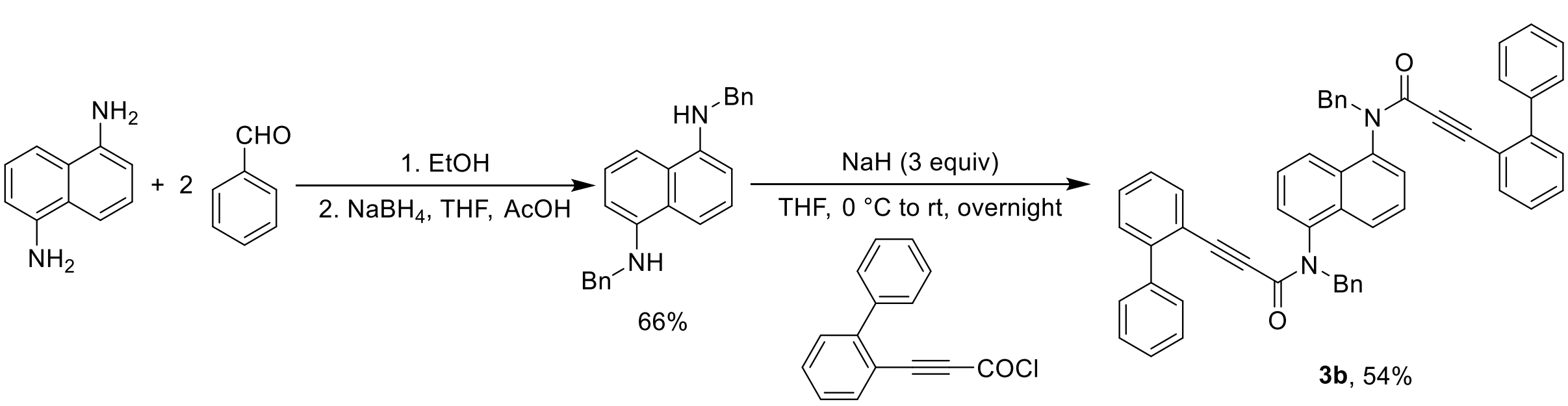

The known compound N^1, N^5-dibenzylnaphthalene-1,5-diamine was prepared from naphthalene-1,5-diamine and benzaldehyde according to literature procedures.[12]

Compound **3b** was synthesized from N^1, N^5-dibenzylnaphthalene-1,5-diamine and 3-(biphenyl-2-yl)propioloyl chloride according to the General Procedure C. The final product was obtained as a yellow solid (1.21 g, 54% yield).

3.28 Synthetic procedure of starting substrate 3c

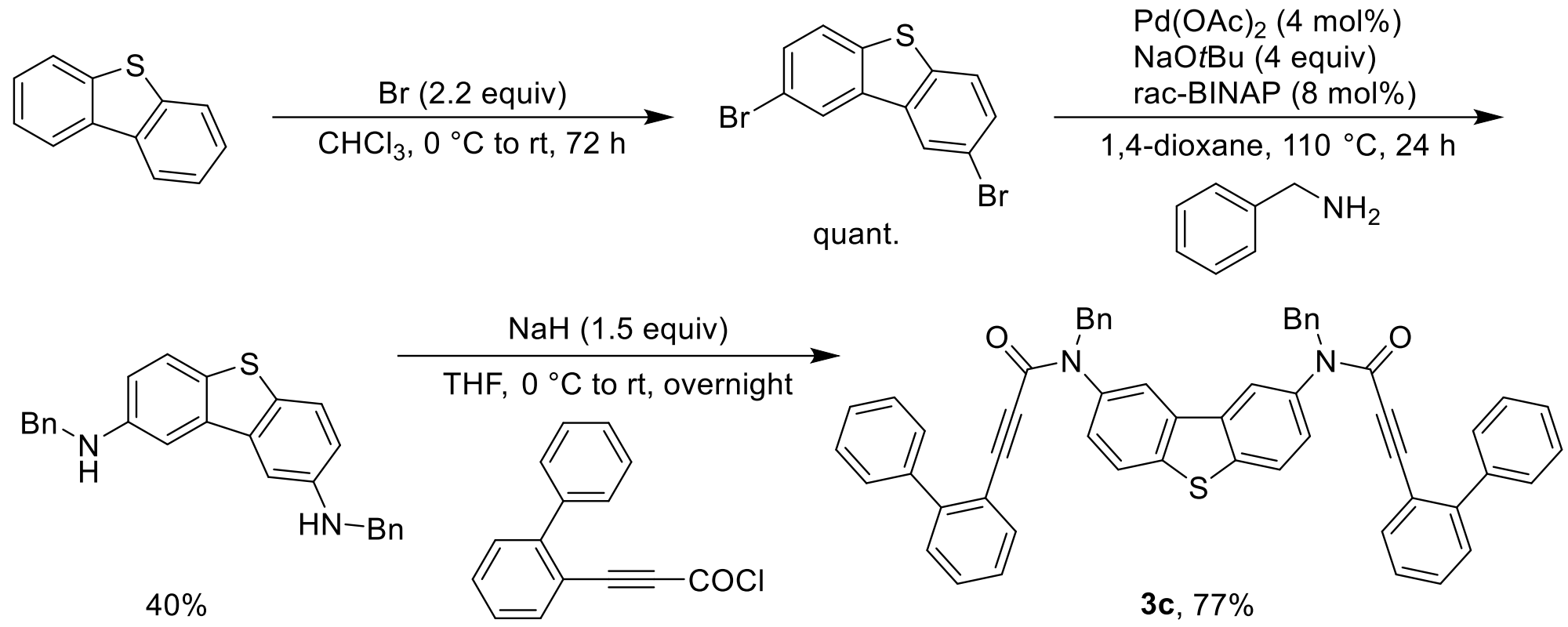

The known compound 2,8-dibromodibenzo[*b*,*d*]thiophene was prepared from dibenzo[*b*,*d*]thiophene according to literature procedures.[13]

N^2,*N*^{8}-dibenzyldibenzo[*b*,*d*]thiophene-2,8-diamine was synthesized from 2,8-dibromodibenzo[*b*,*d*]thiophene and benzylamine according to the General Procedure B. The product was obtained as a white solid (1.58 g, 40%).

Compound **3c** was synthesized from *N*^{2},*N*^{8}-dibenzyldibenzo[*b*,*d*]thiophene-2,8-diamine and 3-(biphenyl-2-yl)propioloyl chloride according to the General Procedure C. The final product was obtained as a yellow solid (1.85 g, 77% yield).

3.29 Synthetic procedure of starting substrate 3d

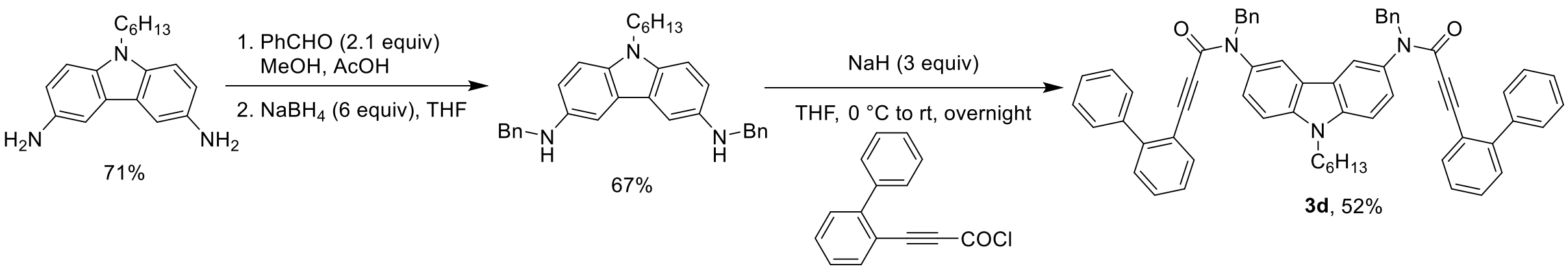

The known compound 9-hexyl-9*H*-carbazole-3,6-diamine was prepared from 9-hexyl-9*H*-carbazole according to literature procedures.[14]

The obtained 9-hexyl-9*H*-carbazole-3,6-diamine (1.41 g, 5 mmol) and benzaldehyde (1.11 g, 10.5 mmol) were dissolved in MeOH (5 mL), and 3 drops of AcOH were added. The resulting mixture was stirred at room temperature for 3 h. After all the starting materials were consumed, 10 mL of dry THF was added, and $NaBH_4$ (0.42 g, 11 mmol) was carefully added in small portions. After gas evolution ceased, the reaction was quenched with water and extracted with ethyl acetate (3 × 20 mL). The organic layers were washed with brine, dried over anhydrous Na_2SO_4, filtered, and concentrated under reduced pressure. The resulting *N*^{3},*N*^{6}-dibenzyl-9-hexyl-9*H*-carbazole-3,6-diamine was purified by chromatography on neutral Al_2O_3 to afford a brown oil (1.55 g, 67% yield).

Compound **3d** was synthesized from *N*^{3},*N*^{6}-dibenzyl-9-hexyl-9*H*-carbazole-3,6-diamine and 3-(biphenyl-2-yl)propioloyl chloride according to the General Procedure C. The final product was obtained as a yellow solid (1.36 g, 52% yield).

3.30 Synthetic procedure of starting substrate 5a

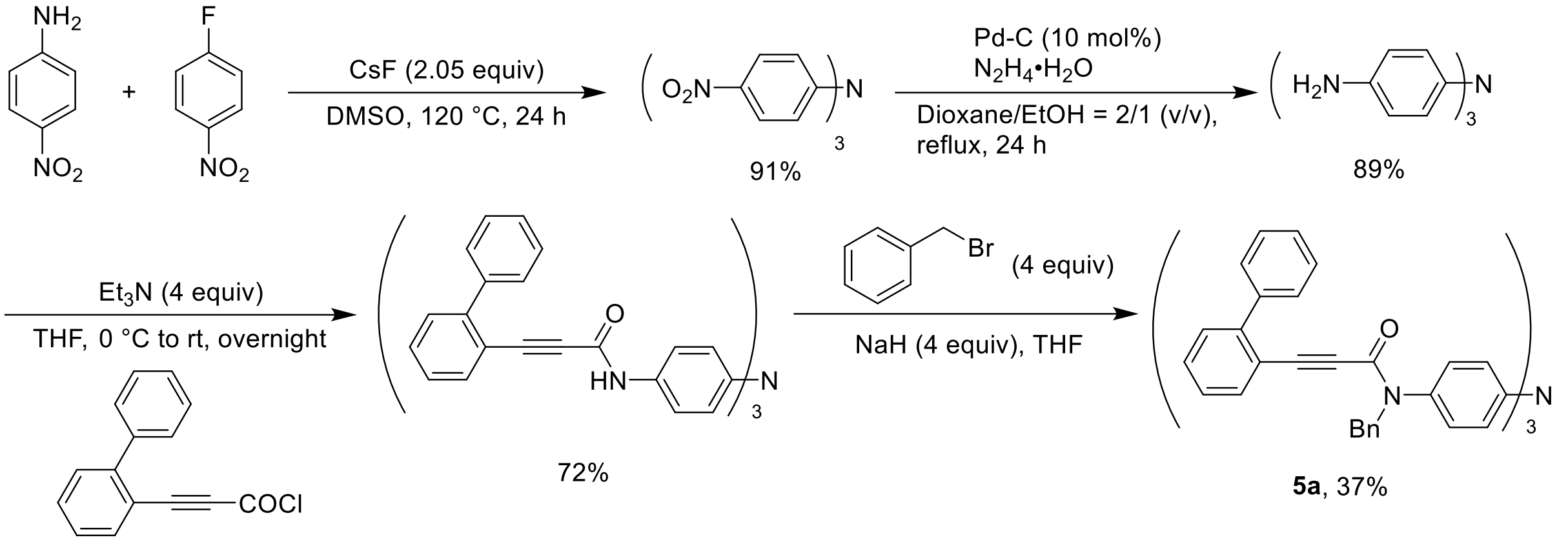

The known compound tris(4-nitrophenyl)amine was prepared from 4-nitroaniline and 1-fluoro-4-nitrobenzene according to literature procedures.[15]

The known compound tris(4-aminophenyl)amine was prepared from tris(4-nitrophenyl)amine according to literature procedures.[16]

Following a modified literature procedure [17], the obtained tris(4-aminophenyl)amine (0.39 g, 1.33 mmol) and Et_3N (0.73 mL, 5.32 mmol) were dissolved in THF (40 mL) and cooled to 0 °C. A solution of 3-(biphenyl-2-yl)propioloyl chloride (prepared above) in THF (20 mL) was added dropwise. The reaction mixture was stirred at room temperature for 12 h. Subsequently, the insoluble material was removed by filtration, and the solvent was evaporated under reduced pressure. After washing the residue with diethyl ether, the crude resulting product, tris[4-(3-(biphenyl-2-yl)propiolamido)phenyl]amine, was obtained as a yellow solid (1.11 g, 72% yield).

Compound **5a** was synthesized from tris[4-(3-(biphenyl-2-yl)propiolamido)phenyl]amine and (bromomethyl)benzene according to the General Procedure C. The final product was obtained as a yellow solid (1.30 g, 37% yield).

3.31 Synthetic procedure of substrate 7a

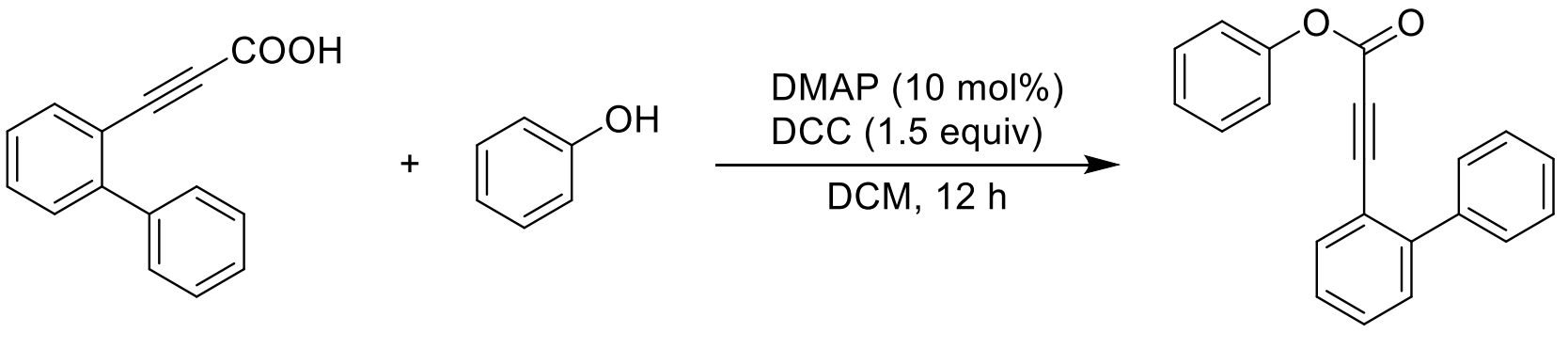

7a, 84%

Following a modified literature procedure[18], phenol (0.28 g, 3.0 mmol) and aryl alkynyl carboxylic acid (0.73 g, 3.3 mmol) were dissolved in dry CH_2Cl_2 (6 mL) and cooled to 0 °C. A solution of DCC (0.93 g, 4.5 mmol) and DMAP (36.7 mg, 0.30 mmol) in CH_2Cl_2 (3 mL) was added dropwise. The resulting reaction mixture was stirred at room temperature for 12 h. Upon completion, the mixture was diluted with CH_2Cl_2 and filtered through a pad of Celite. The filtrate was concentrated, and the residue was purified by silica gel chromatography to afford **7a** as a white solid (0.75 g, 84% yield).

3.32 Synthetic procedure of substrate 7b

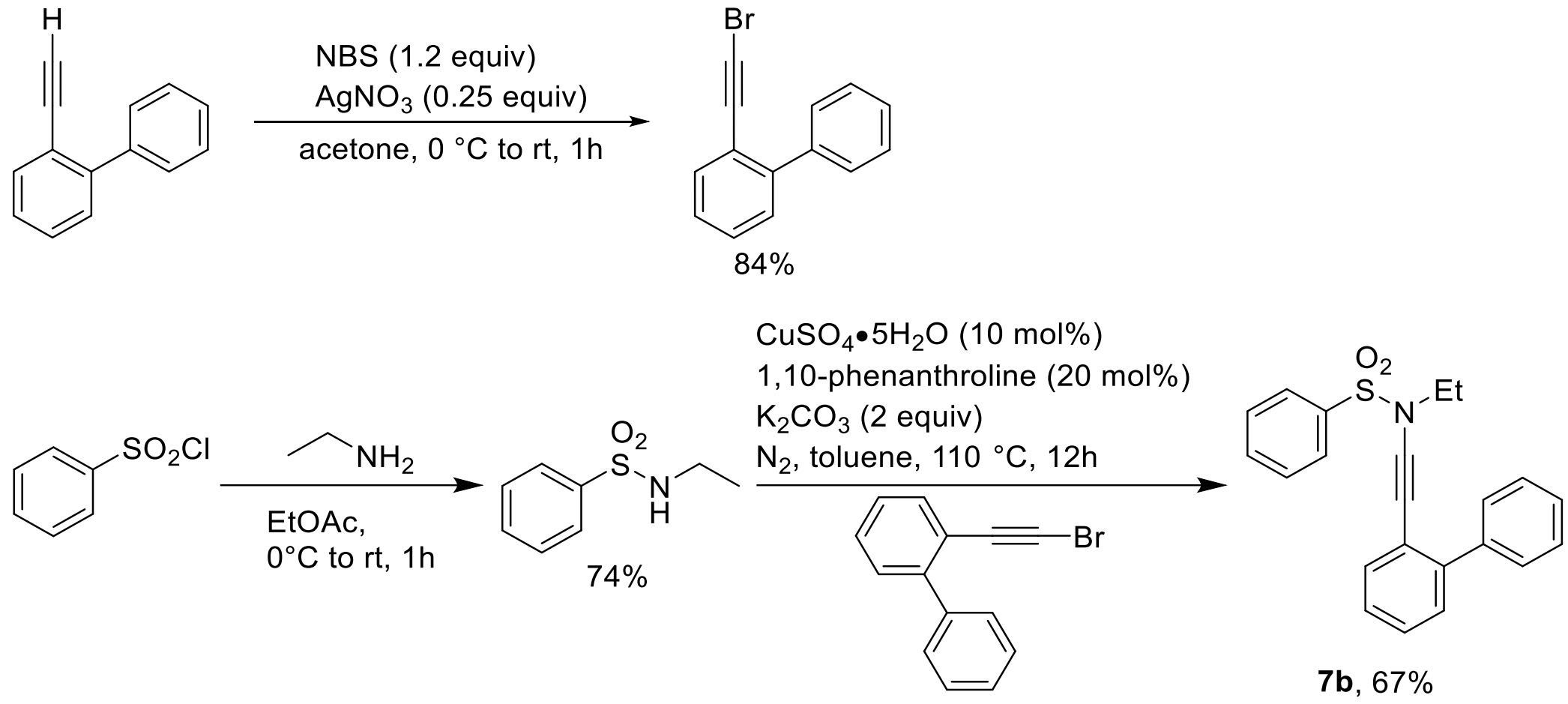

7b, 67%

The known compound 2-(bromoethynyl)-1,1'-biphenyl was prepared from 2-ethynyl-1,1'-biphenyl according to literature procedures[19].

The known compound N-ethylbenzenesulfonamide was prepared from benzenesulfonyl chloride and ethanamine according to literature procedures[20].

A Schlenk flask equipped with a magnetic stir bar was charged with *N*-ethylbenzenesulfonamide (0.67 g, 3.6 mmol), $CuSO_4 \cdot 5H_2O$ (150 mg, 0.6 mmol), 1,10-phenanthroline (0.22 g, 1.2 mmol) and K_2CO_3 (0.83 g, 6 mmol), then purged with N_2 three times. Toluene (6 mL) and 2-(bromoethynyl)-1,1'-biphenyl (0.77 g, 3 mmol) were added sequentially. The reaction mixture was stirred at 110 °C for 12 h. After cooling to room temperature, the mixture was diluted with CH_2Cl_2 and filtered through a pad of Celite. The filtrate was concentrated, and the residue was purified by silica gel chromatography to afford **7b** as a white solid (0.74 g, 67%).

3.33 Synthetic procedure of substrate 7c

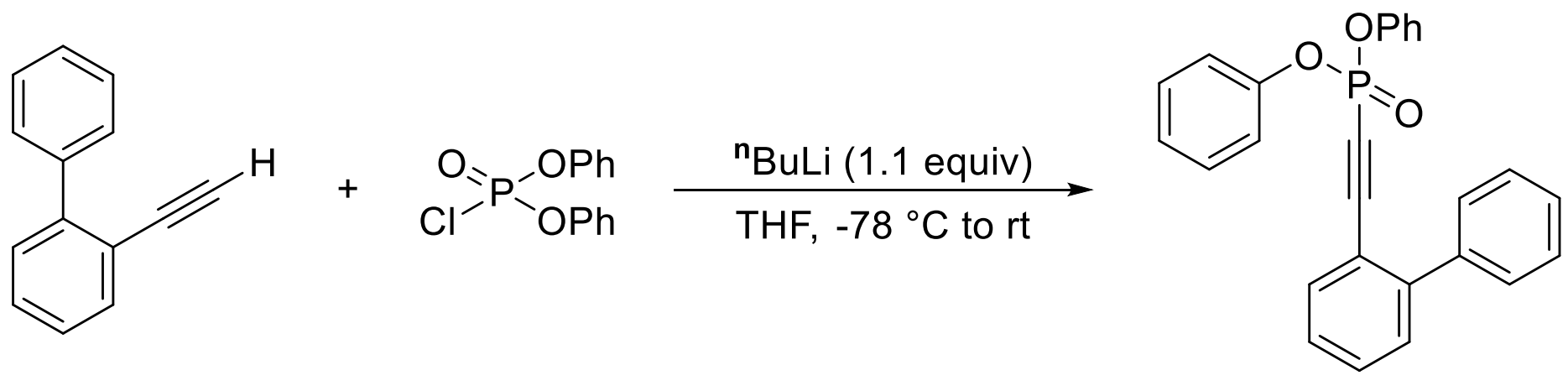

7c, 22%

Following a modified literature procedure,[21] in a flame-dried two-necked Schlenk flask under a N_2 atmosphere, 2-ethynyl-biphenyl (0.36 g, 2 mmol) was dissolved in dry THF (7 mL) and cooled to –78 °C. To this solution, *n*-BuLi (1.6 M in hexane, 1.4 mL, 2.2 mmol) was added dropwise, and the mixture was stirred at –78 °C for 1 h. A solution of diphenyl phosphorochloridate (0.81 g, 3 mmol) in THF (1 mL) was then added slowly, and the reaction mixture was stirred at –78 °C for 2 h. Subsequently, the mixture was gradually warmed to room temperature and stirred overnight. The reaction was quenched with saturated aqueous NH_4Cl, extracted with ethyl acetate (3 × 10 mL) and washed with brine. The combined organic phases were dried over Na_2SO_4, filtered, and concentrated. The crude residue was purified by silica

gel chromatography to afford compound **7c** as a colorless oil (0.18 g, 22% yield).

4. Isomerization of unsymmetric (*E*)- and (*Z*)- products

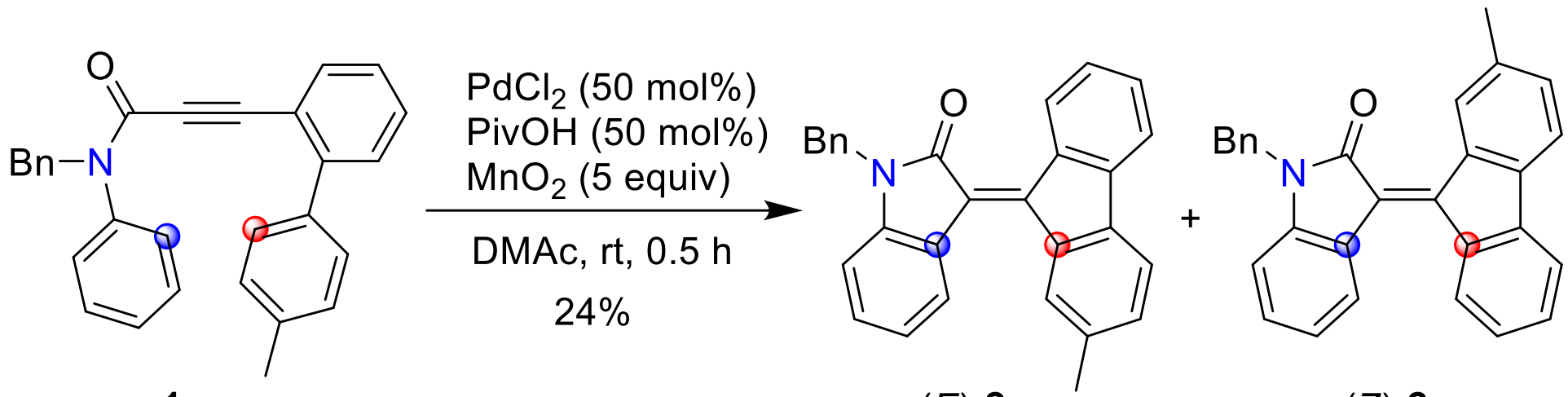

1q (*E*)-**2q** (*Z*)-**2q**

(1) (*E*)-**2q** / (*Z*)-**2q** = 2/1 → rt, 24 h without Pd-catalyst → (*E*)-**2q** / (*Z*)-**2q** = 1.3/1

(2) Standard conditions (80 °C): 95%
(*E*)-**2q** / (*Z*)-**2q** = 1.1/1

To an anhydrous DMAc solution (1.6 mL, 0.125 M) were added *N*-benzyl-3-(4′-methyl-biphenyl-2-yl)-N-phenylpropiolamide (**1q**) (80.2 mg, 0.2 mmol), $PdCl_2$ (17.7 mg, 0.10 mmol), PivOH (10.2 mg, 0.10 mmol), and MnO_2 (86.9 mg, 1.0 mmol). The reaction vial was purged with Ar atmosphere and capped. The reaction mixture was stirred at room temperature for 0.5 h. The mixture was filtered through a pad of Celite and washed with CH_2Cl_2. The filtrate was washed with water, and then passed through a short silica gel chromatography to remove residual Pd catalyst and MnO_2. The solvent was carefully removed under reduced pressure at room temperature without heating to avoid rapid isomerization of the products. The crude residue was dissolved in $CDCl_3$, and anisole was added as an internal standard. The yield and product ratio were determined by ^{1}H NMR analysis, giving a 2:1 ratio of (*E*)-**2q** to (*Z*)-**2q**. The combined ^{1}H NMR yield of (*E*)-**2q** and (*Z*)-**2q** was 24%, with 61% of starting material **1o** remaining. The same NMR sample was kept in $CDCl_3$ at room temperature, and the ^{1}H NMR spectrum was recorded again after 24 h, which showed that the (*E*)-**2q**/(*Z*)-**2q** ratio changed to 1.3:1. Under the standard reaction conditions ($PdCl_2$ (10 mol%), PivOH (10 mol%), MnO_2 (5 equiv), DMAc, 80 °C, 6 h), the reaction of **1q** (0.2 mmol) afforded a mixture of (*E*)-**2q** and (*Z*)-**2q** in 95% NMR yield, and the product mixture was isolated in 87% yield after column chromatography. The signal assignments of (*E*)-**2q** and (*Z*)-**2q** in the ^{1}H NMR spectra were established by COSY, TOCSY, and NOE experiments.

5. Kinetic isotope effect (KIE) experiments

5.1 Intramolecular competition kinetic isotope effect (KIE) experiments

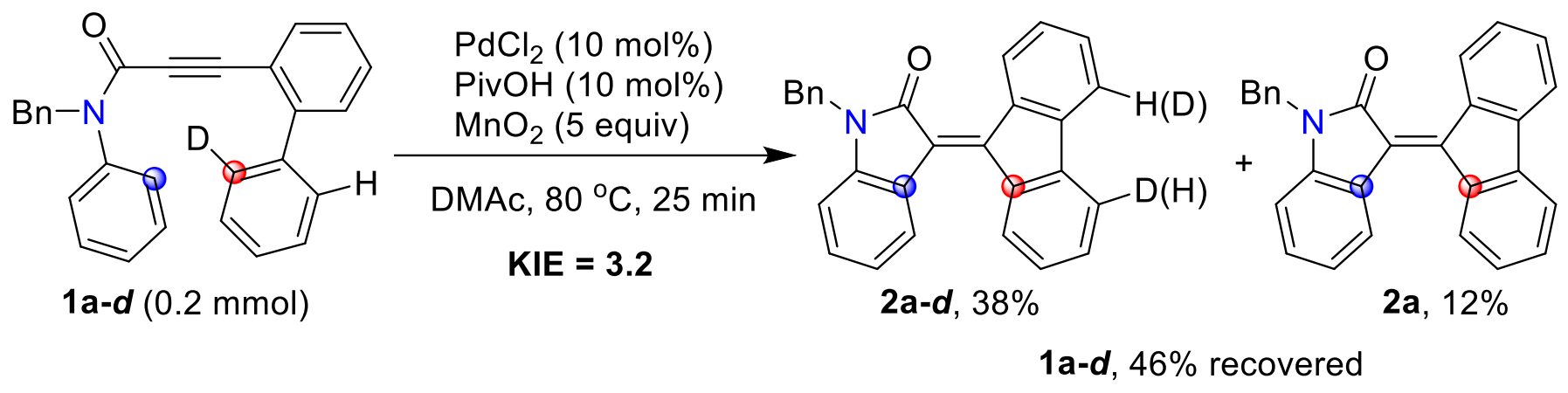

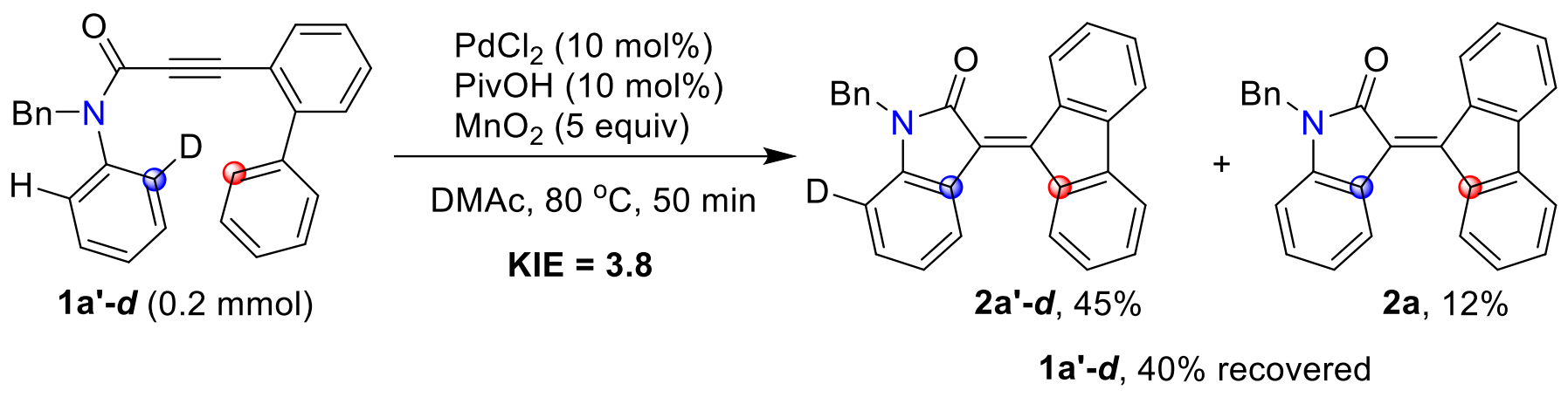

Intramolecular competition KIE experiments were carried out under the standard reaction conditions using the corresponding deuterium-labeled substrates. The KIE values were determined based on the relative product yields measured by ^{1}H NMR spectroscopy using anisole as an internal standard.

5.2 Intermolecular competition kinetic isotope effect (KIE) experiments

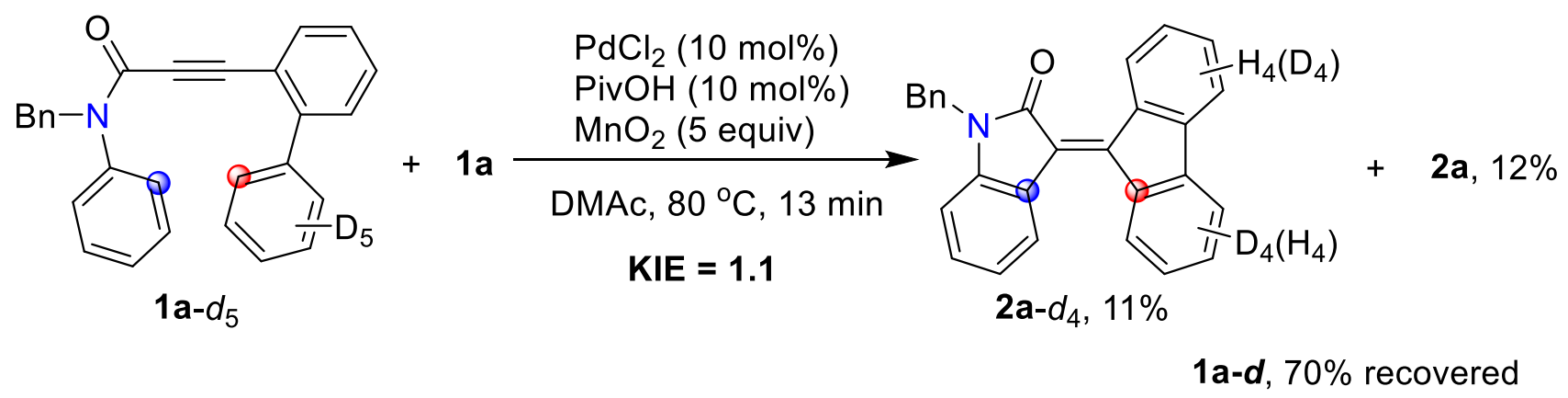

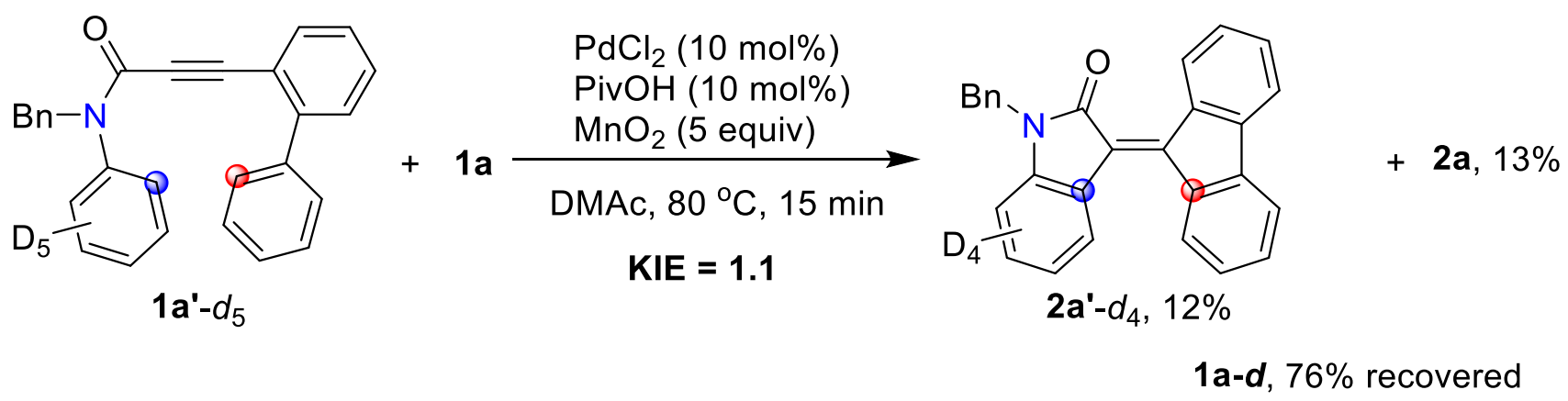

To an anhydrous DMAc solution (1.6 mL, 0.125 M) were added the deuterium-labeled substrate **1a-d$_5$** or **1a′-d$_5$** (39.2 mg, 0.10 mmol), **1a** (38.7 mg, 0.10 mmol), $PdCl_2$ (3.55 mg, 0.020 mmol), PivOH (2.04 mg, 0.020 mmol), and MnO_2 (86.9 mg, 1.0 mmol). The reaction vial was purged with Ar and capped. The reaction mixture was stirred at 80 °C for 13 min when **1a-d$_5$** was used and for 15 min when 1a′-d$_5$ was used. The mixture was filtered through a pad of Celite and washed with CH_2Cl_2. The filtrate was washed with water and passed through a short pad of silica gel to remove residual Pd catalyst and MnO_2. The crude residue was dissolved in $CDCl_3$, and anisole was added as an internal standard. The product yields and ratios were determined by ^{1}H NMR analysis.

6. Summary of single crystal 2n

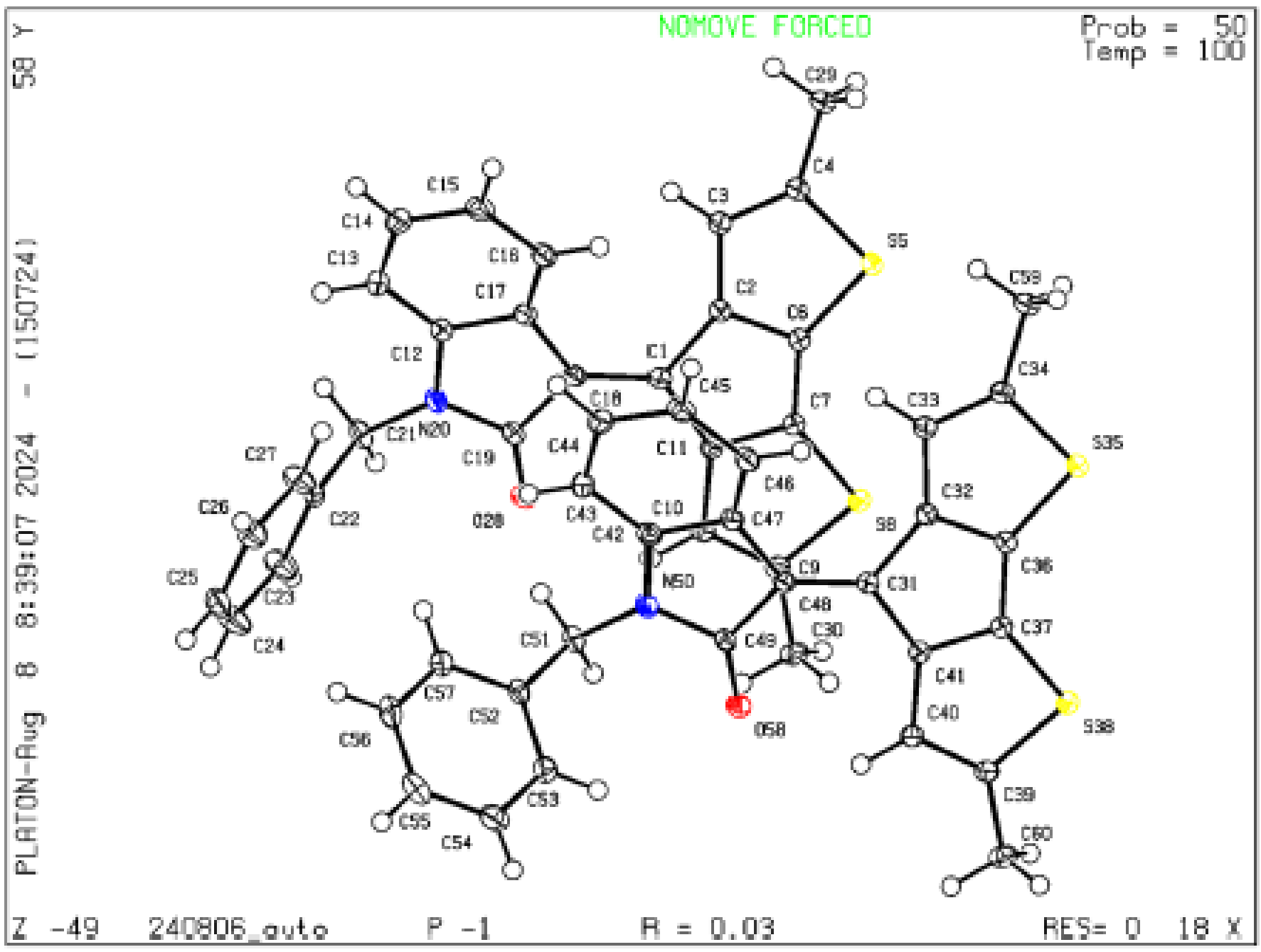

Table S1 Crystal data and structure refinement for 240806_auto.

Identification code	240806_auto
Empirical formula	$C_{52}H_{38}N_2O_2S_4$
Formula weight	851.08
Temperature/K	99.95(10)
Crystal system	triclinic
Space group	P-1
a/Å	9.34770(10)
b/Å	10.19310(10)
c/Å	22.6116(2)
α/°	98.9060(10)
β/°	90.9480(10)
γ/°	109.7770(10)
Volume/Å^3	1997.44(4)
Z	2
ρ_{calc}g/cm^3	1.415
μ/mm^{-1}	0.286
F(000)	888.0
Crystal size/mm^3	0.2 × 0.2 × 0.1
Radiation	Mo Kα (λ = 0.71073)
2Θ range for data collection/°	4.644 to 61.794

Index ranges	$-13 \le h \le 13$, $-14 \le k \le 13$, $-32 \le l \le 32$
Reflections collected	108817
Independent reflections	11211 [R_{int} = 0.0228, R_{sigma} = 0.0129]
Data/restraints/parameters	11211/0/545
Goodness-of-fit on F^2	1.049
Final R indexes [I>=2σ (I)]	R_1 = 0.0313, wR_2 = 0.0908
Final R indexes [all data]	R_1 = 0.0362, wR_2 = 0.0945
Largest diff. peak/hole / e $Å^{-3}$	0.46/-0.28

7. UV-vis absorption spectra

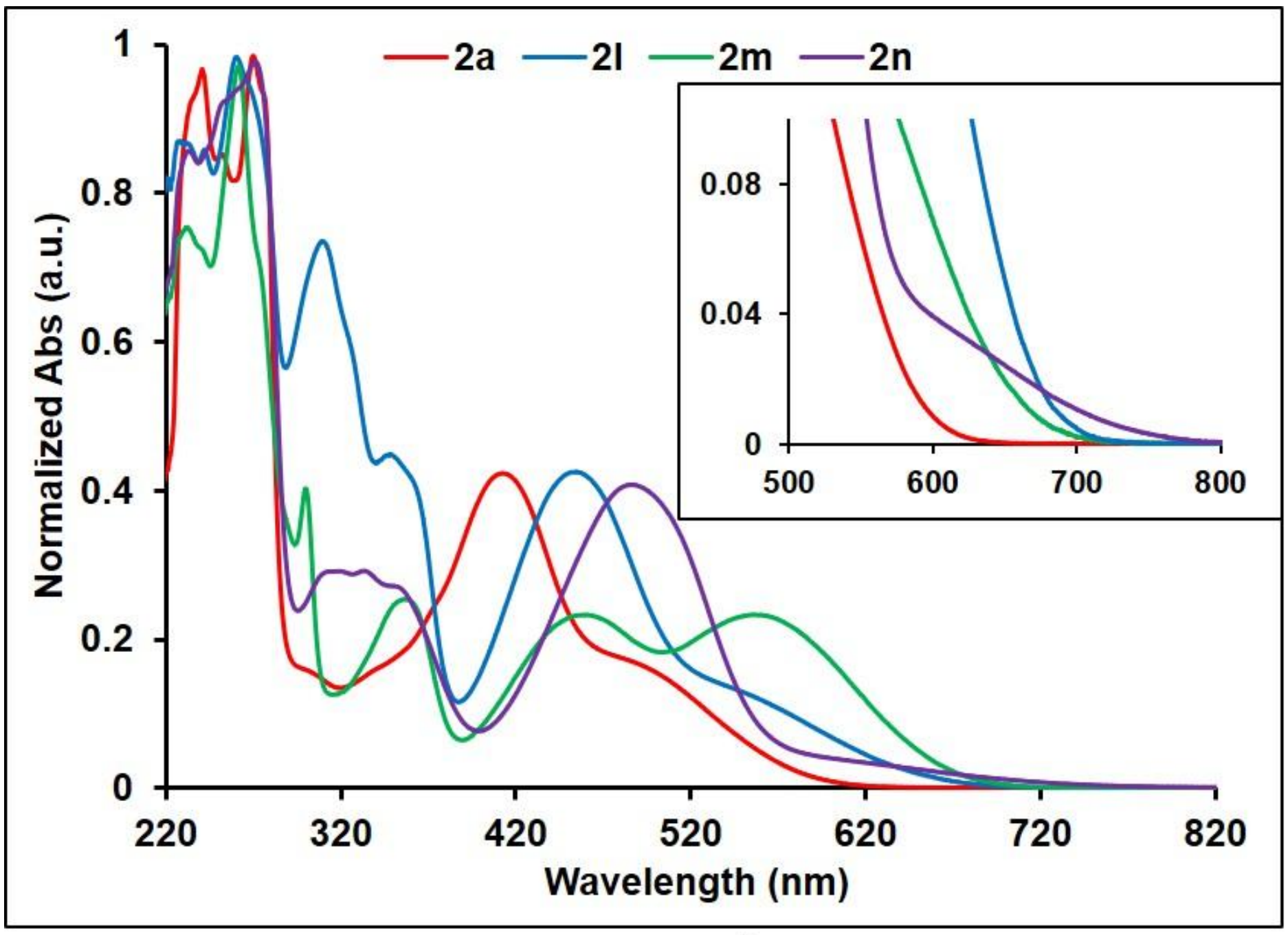

2a
λ_{max} = 496 nm
λ_{onset} = 582 nm

2l
λ_{max} = 545 nm
λ_{onset} = 656 nm

2m
λ_{max} = 559 nm
λ_{onset} = 666 nm

2n
λ_{max} = 594 nm
λ_{onset} = 733 nm

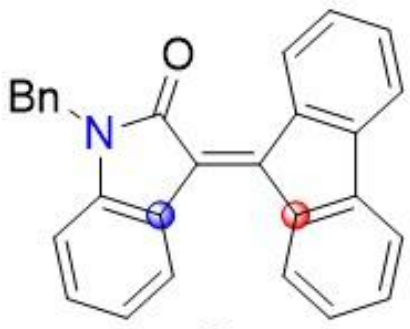

2a

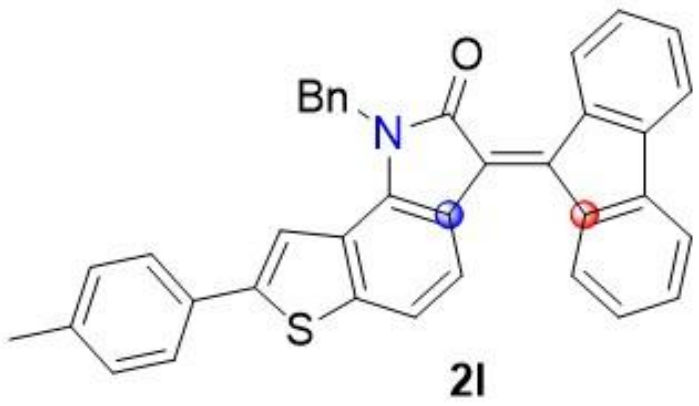

2l

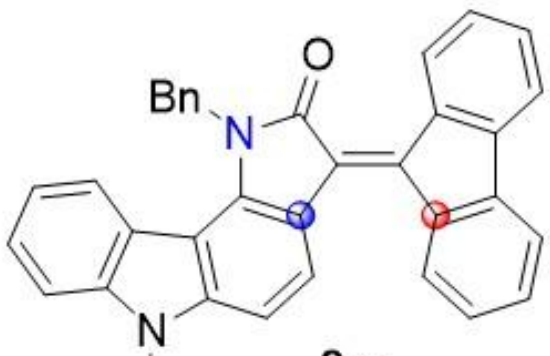

2m

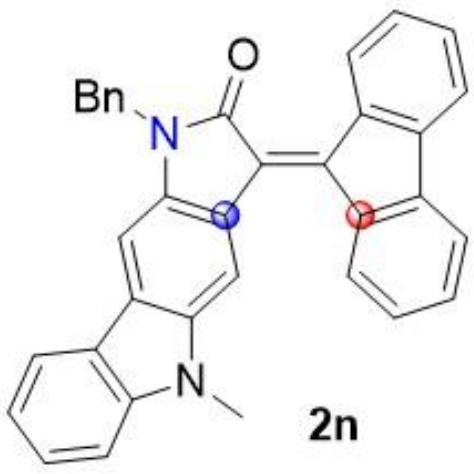

2n

Figure S1. UV-Vis absorption spectra **2a**, **2l**, **2m**, and **2n** in CH_2Cl_2 (10^{-5} M).

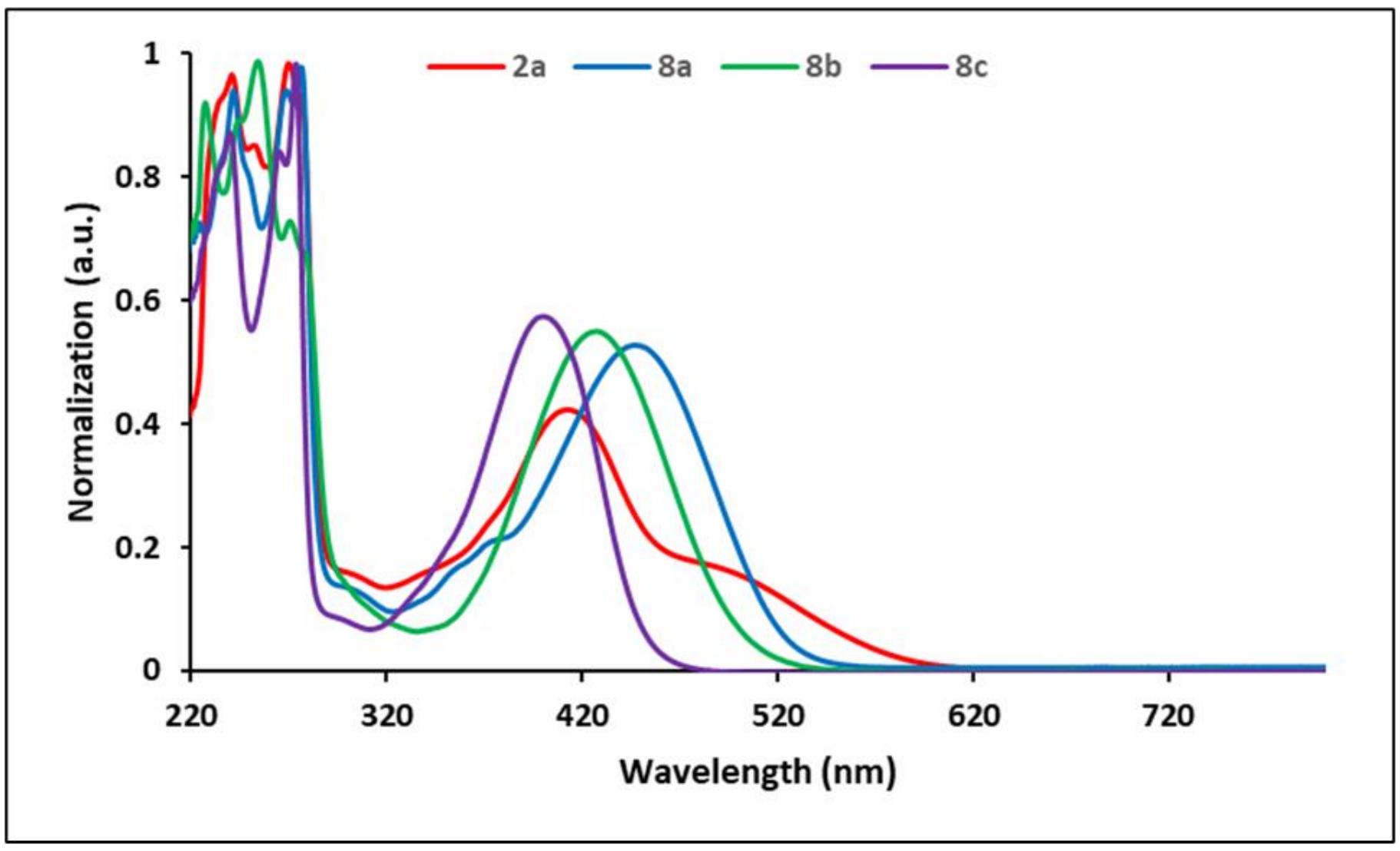

2a
λ_{max} = 496 nm
λ_{onset} = 582 nm

8a
λ_{max} = 496 nm
λ_{onset} = 582 nm

8b
λ_{max} = 426 nm
λ_{onset} = 501 nm

8c
λ_{max} = 400 nm
λ_{onset} = 450 nm

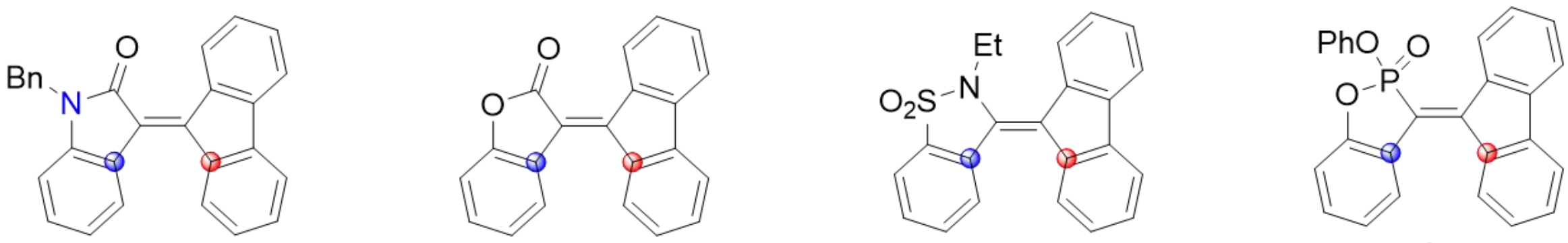

2a **8a** **8b** **8c**

Figure S2. UV-Vis absorption spectra **2a**, **8a**, **8b**, and **8c** in CH_2Cl_2 (10^{-5} M).

8. Cyclic voltammetry (CV) of products

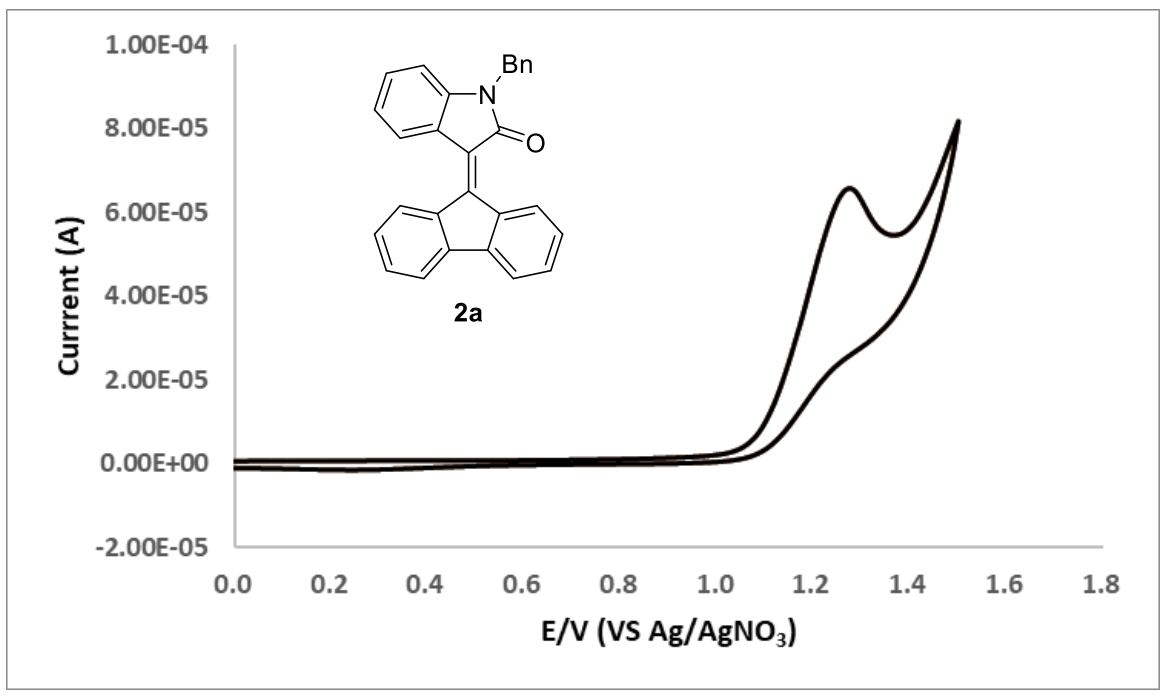

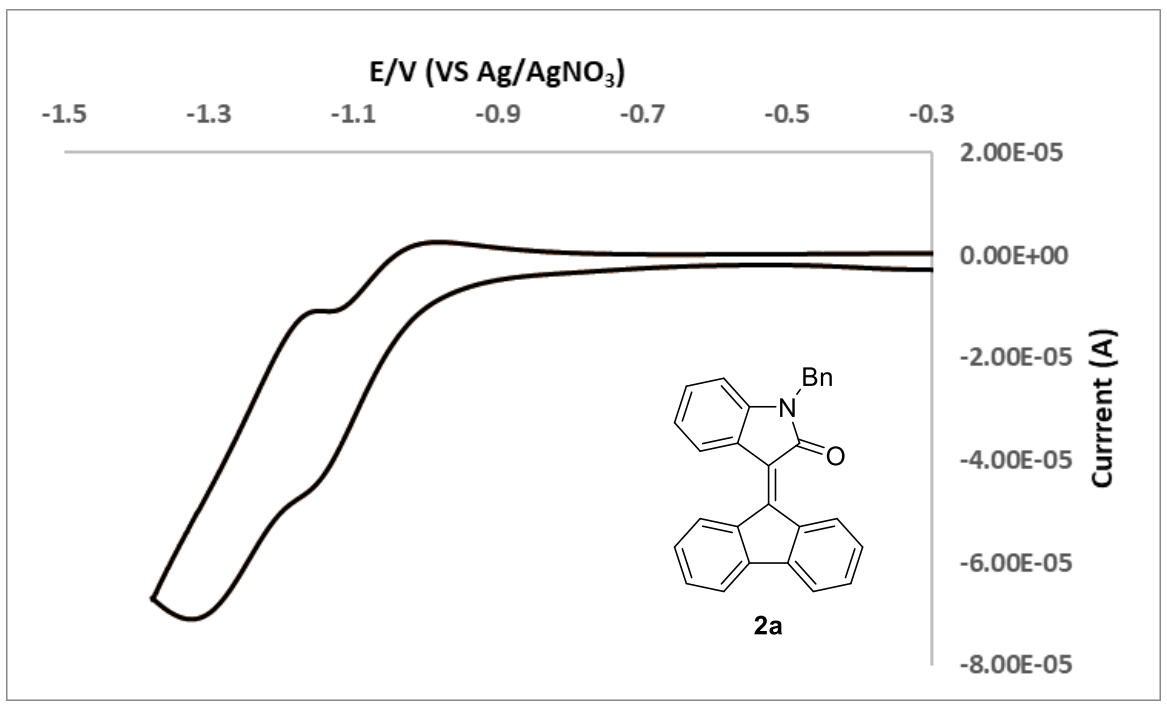

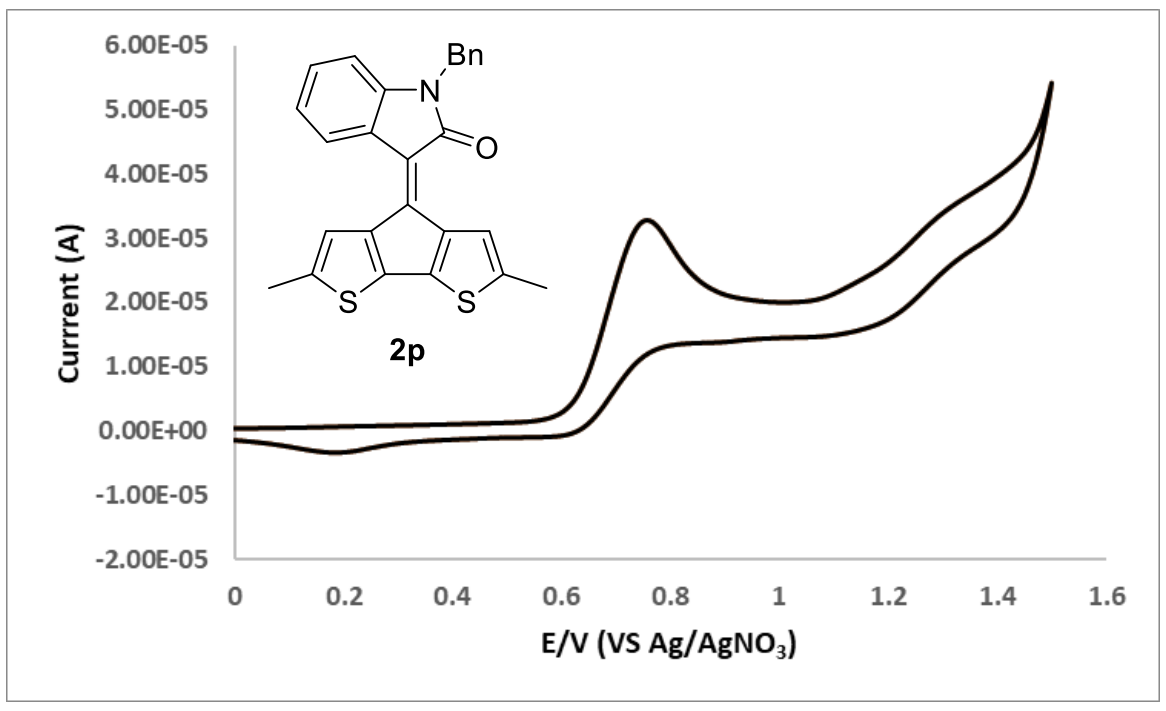

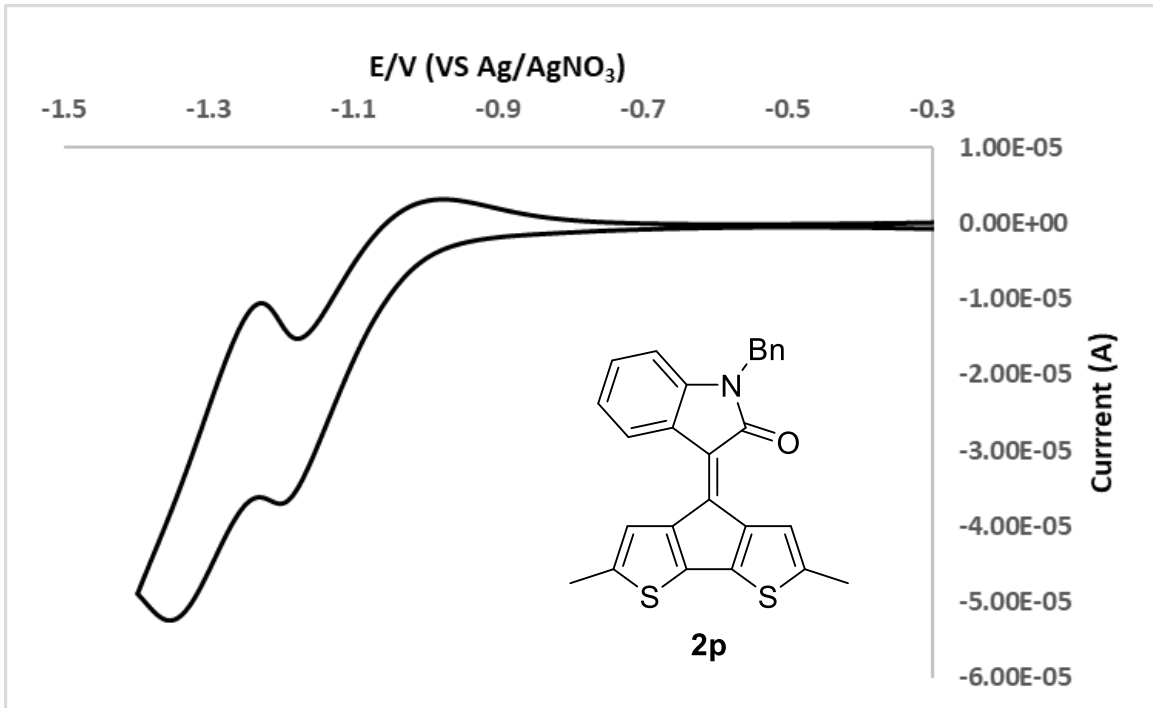

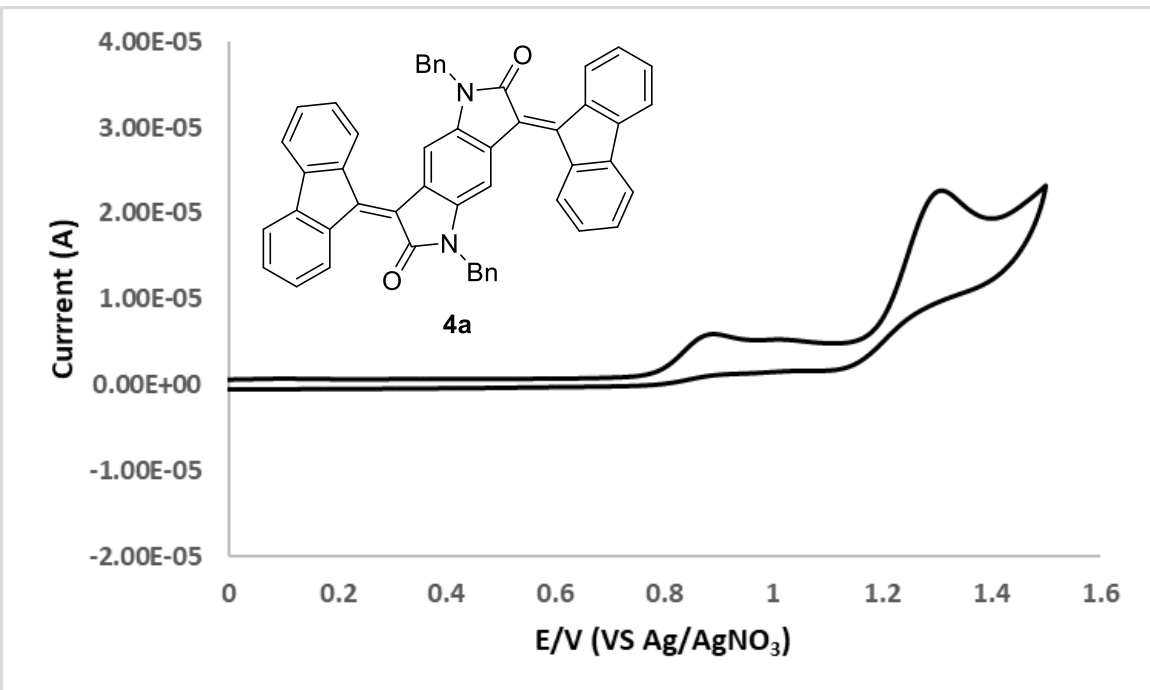

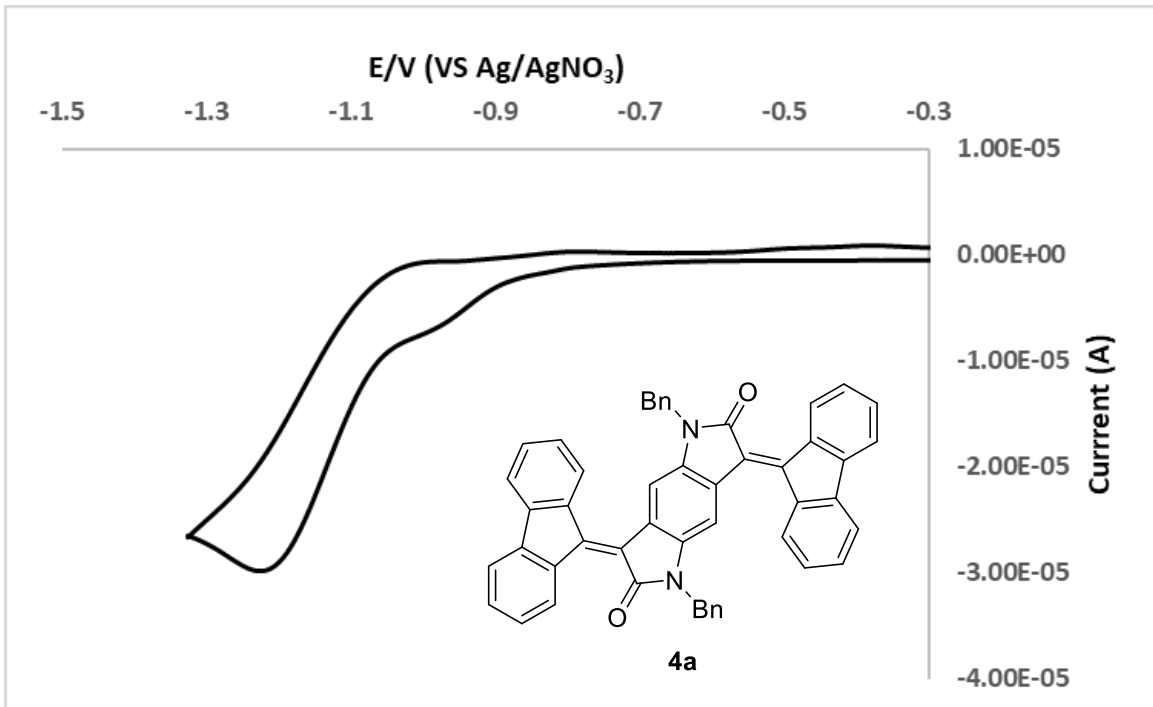

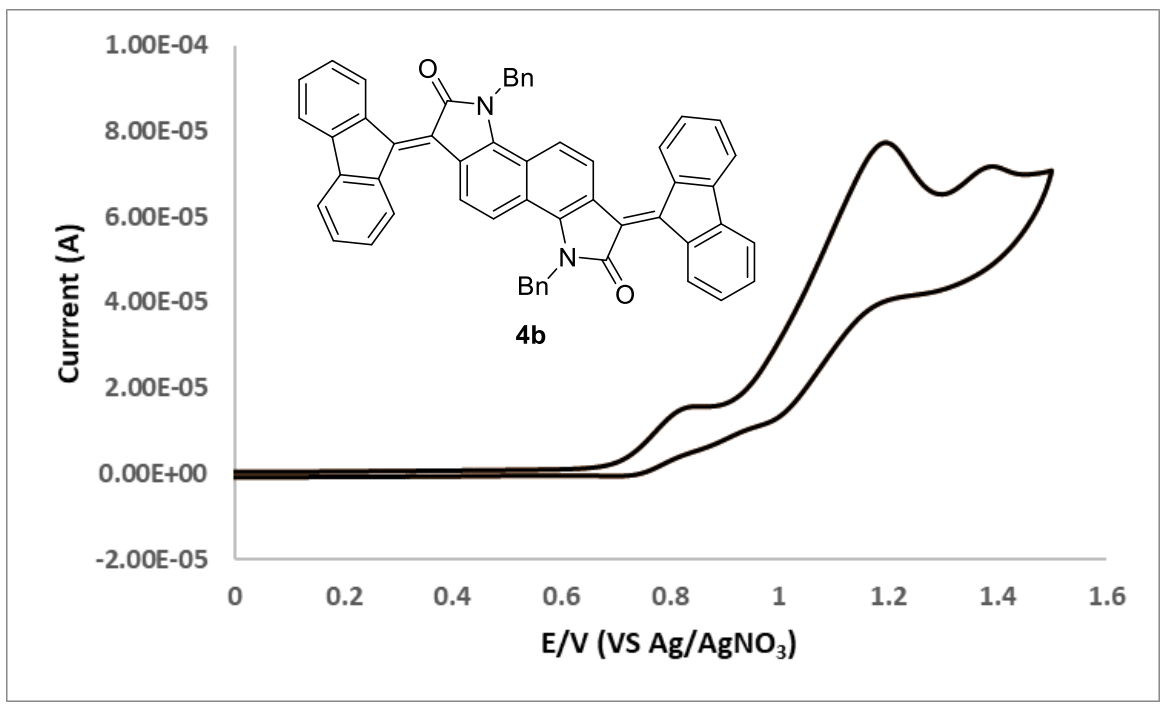

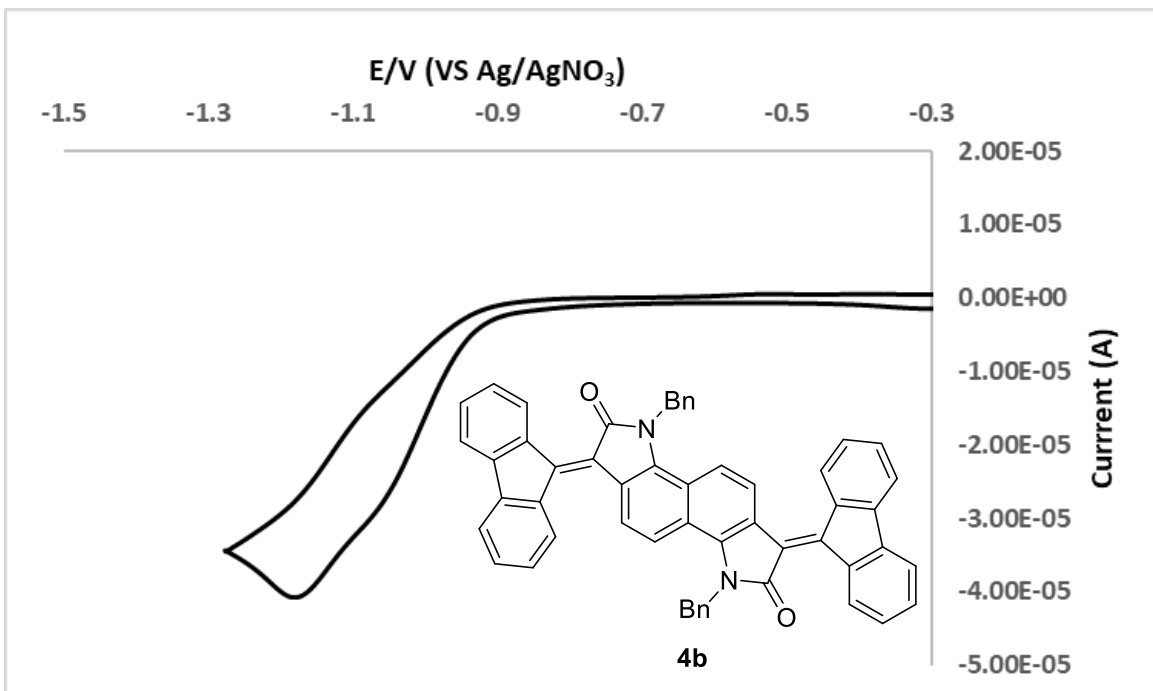

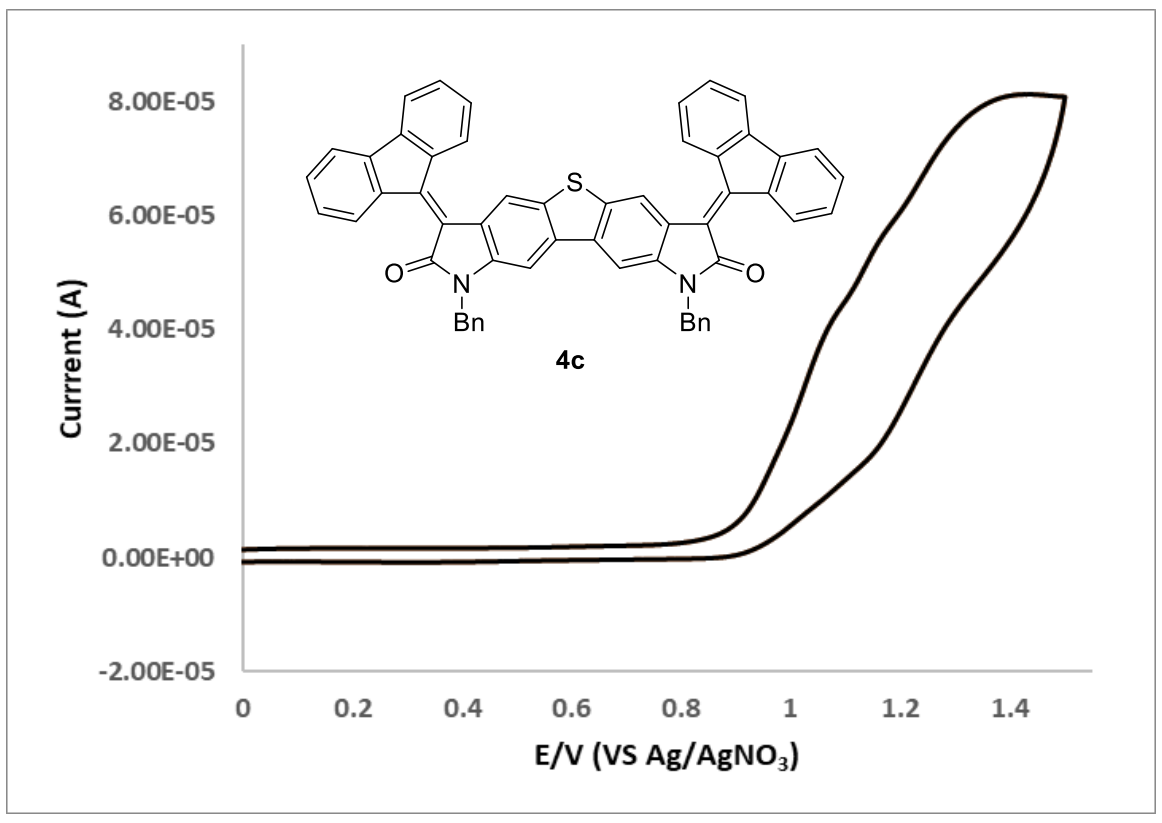
8.00E-05
6.00E-05
4.00E-05
2.00E-05
0.00E+00
-2.00E-05
Current (A)
0
0.2
0.4
0.6
0.8
1
1.2
1.4
E/V (VS Ag/AgNO3)
Bn
Bn
4c

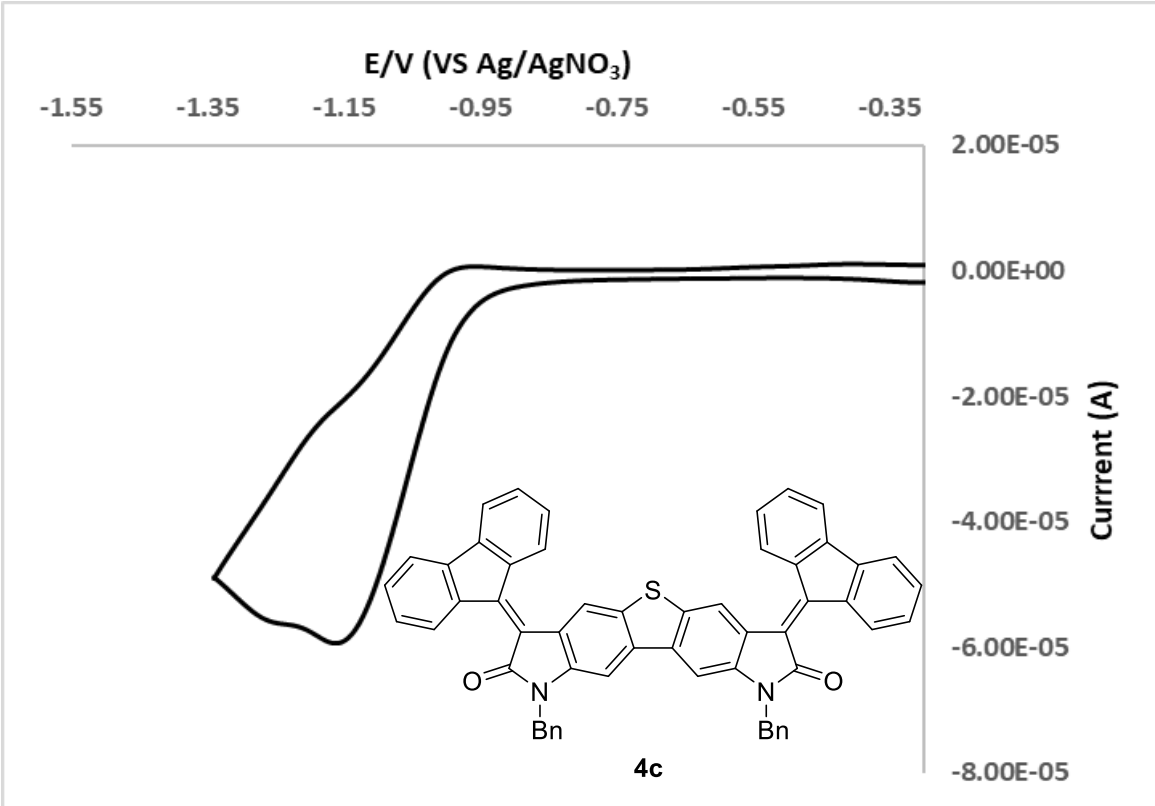
E/V (VS Ag/AgNO3)
-1.55
-1.35
-1.15
-0.95
-0.75
-0.55
-0.35
2.00E-05
0.00E+00
-2.00E-05
-4.00E-05
-6.00E-05
-8.00E-05
Current (A)
Bn
Bn
4c

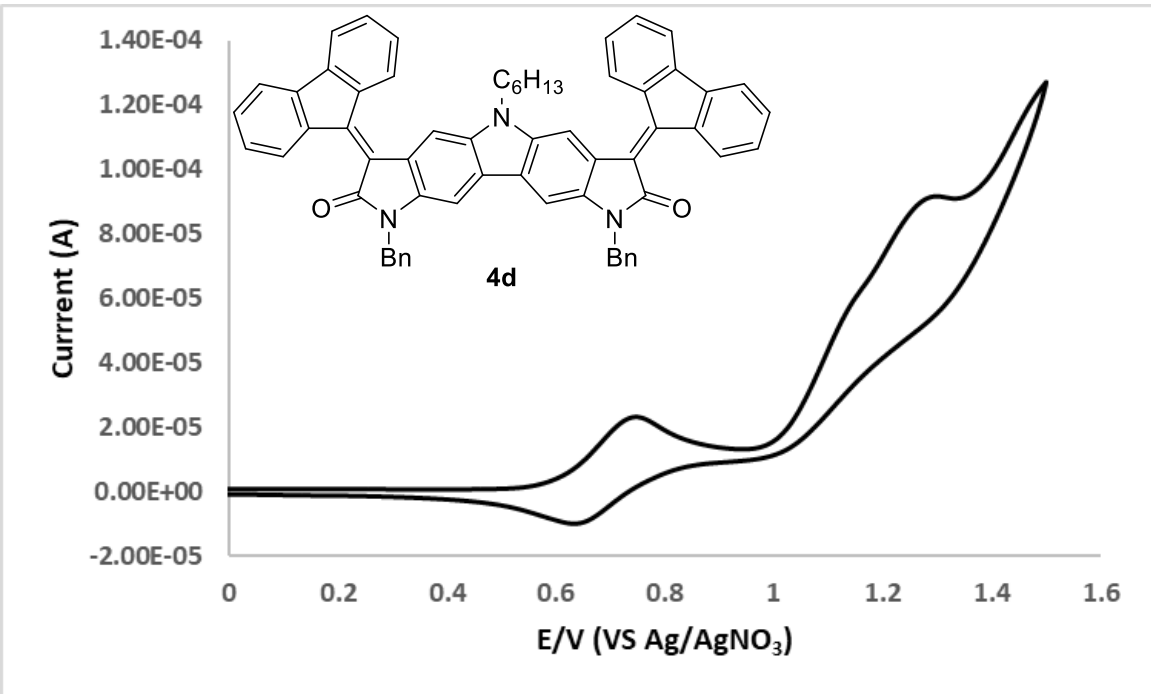
1.40E-04
1.20E-04
1.00E-04
8.00E-05
6.00E-05
4.00E-05
2.00E-05
0.00E+00
-2.00E-05
Current (A)
0
0.2
0.4
0.6
0.8
1
1.2
1.4
1.6
E/V (VS Ag/AgNO3)
C6H13
Bn
Bn
4d

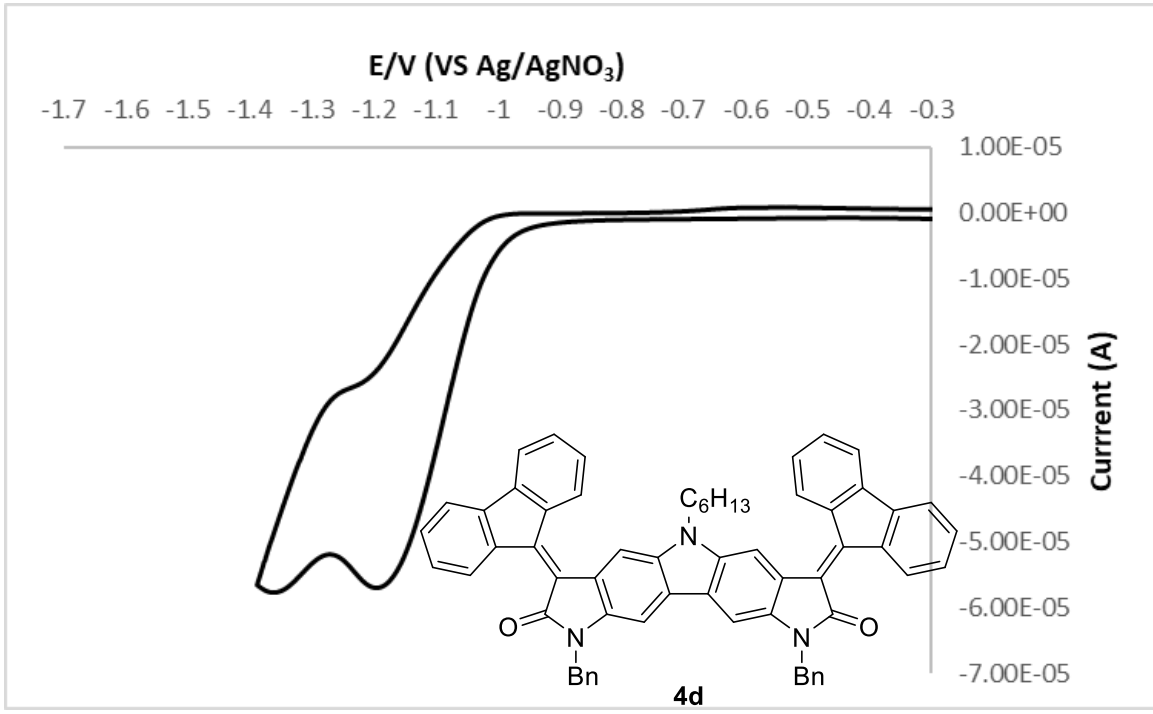
E/V (VS Ag/AgNO3)
-1.7
-1.6
-1.5
-1.4
-1.3
-1.2
-1.1
-1
-0.9
-0.8
-0.7
-0.6
-0.5
-0.4
-0.3
1.00E-05
0.00E+00
-1.00E-05
-2.00E-05
-3.00E-05
-4.00E-05
-5.00E-05
-6.00E-05
-7.00E-05
Current (A)
C6H13
Bn
Bn
4d

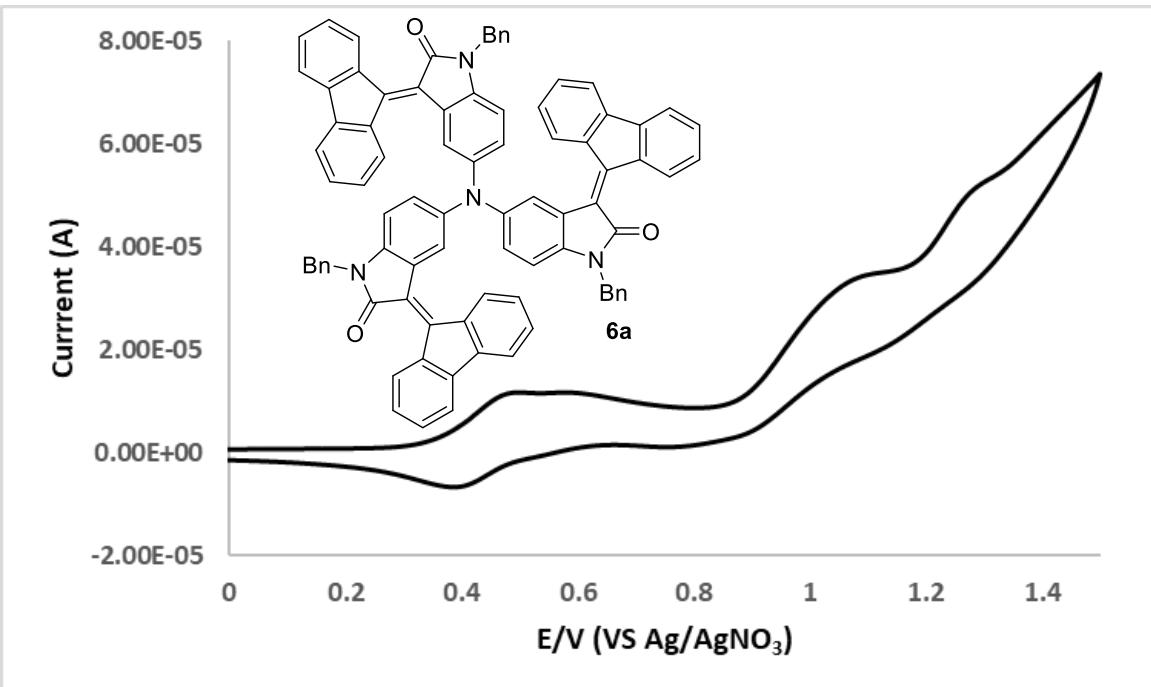
8.00E-05
6.00E-05
4.00E-05
2.00E-05
0.00E+00
-2.00E-05
Current (A)
0
0.2
0.4
0.6
0.8
1
1.2
1.4
E/V (VS Ag/AgNO3)
Bn
Bn
Bn
6a

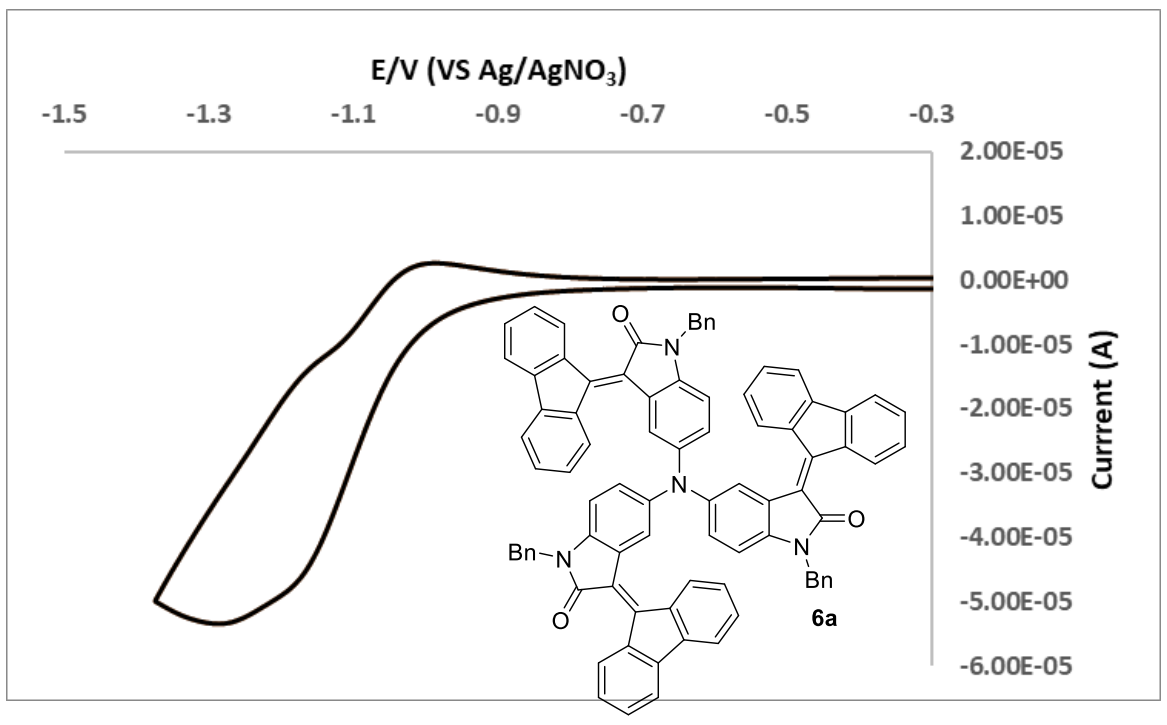
E/V (VS Ag/AgNO3)
-1.5
-1.3
-1.1
-0.9
-0.7
-0.5
-0.3
2.00E-05
1.00E-05
0.00E+00
-1.00E-05
-2.00E-05
-3.00E-05
-4.00E-05
-5.00E-05
-6.00E-05
Current (A)
Bn
Bn
Bn
6a

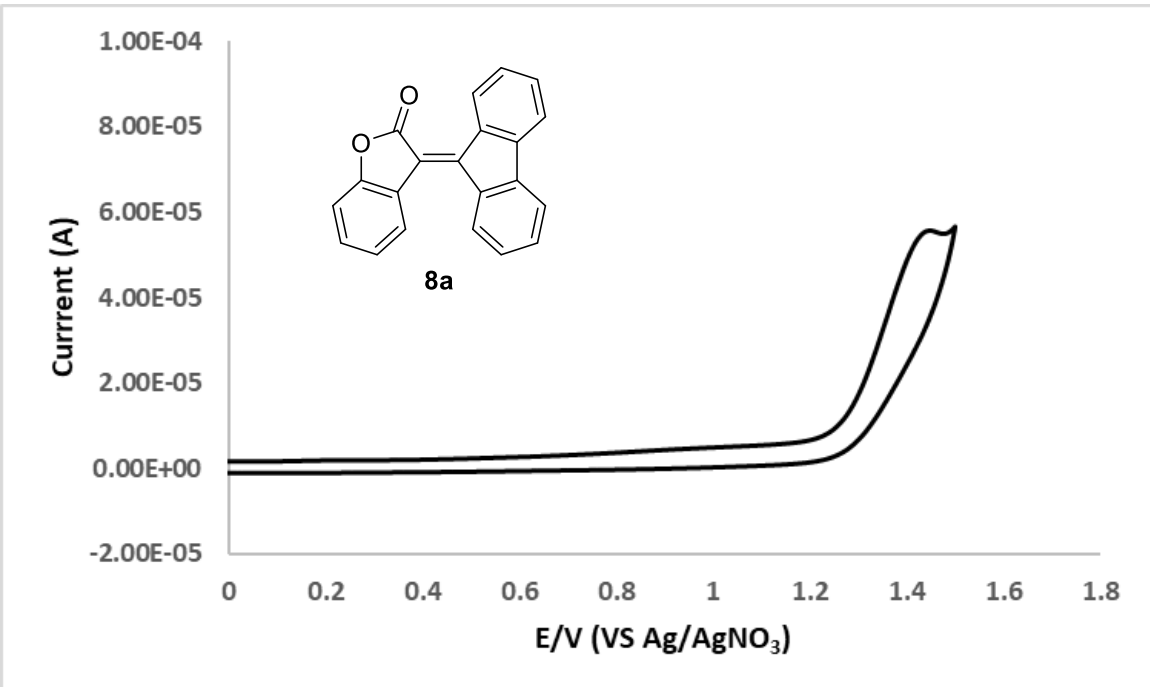
1.00E-04
8.00E-05
6.00E-05
4.00E-05
2.00E-05
0.00E+00
-2.00E-05
Current (A)
0
0.2
0.4
0.6
0.8
1
1.2
1.4
1.6
1.8
E/V (VS Ag/AgNO3)
8a

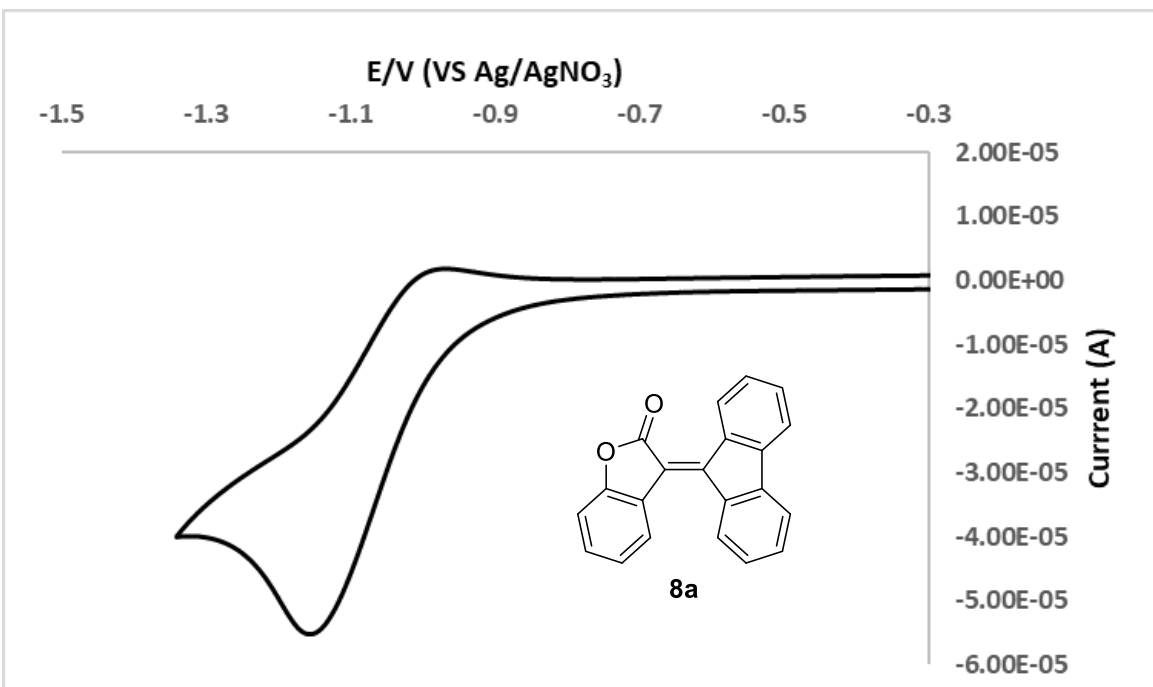
E/V (VS Ag/AgNO3)
-1.5
-1.3
-1.1
-0.9
-0.7
-0.5
-0.3
2.00E-05
1.00E-05
0.00E+00
-1.00E-05
-2.00E-05
-3.00E-05
-4.00E-05
-5.00E-05
-6.00E-05
Current (A)
8a

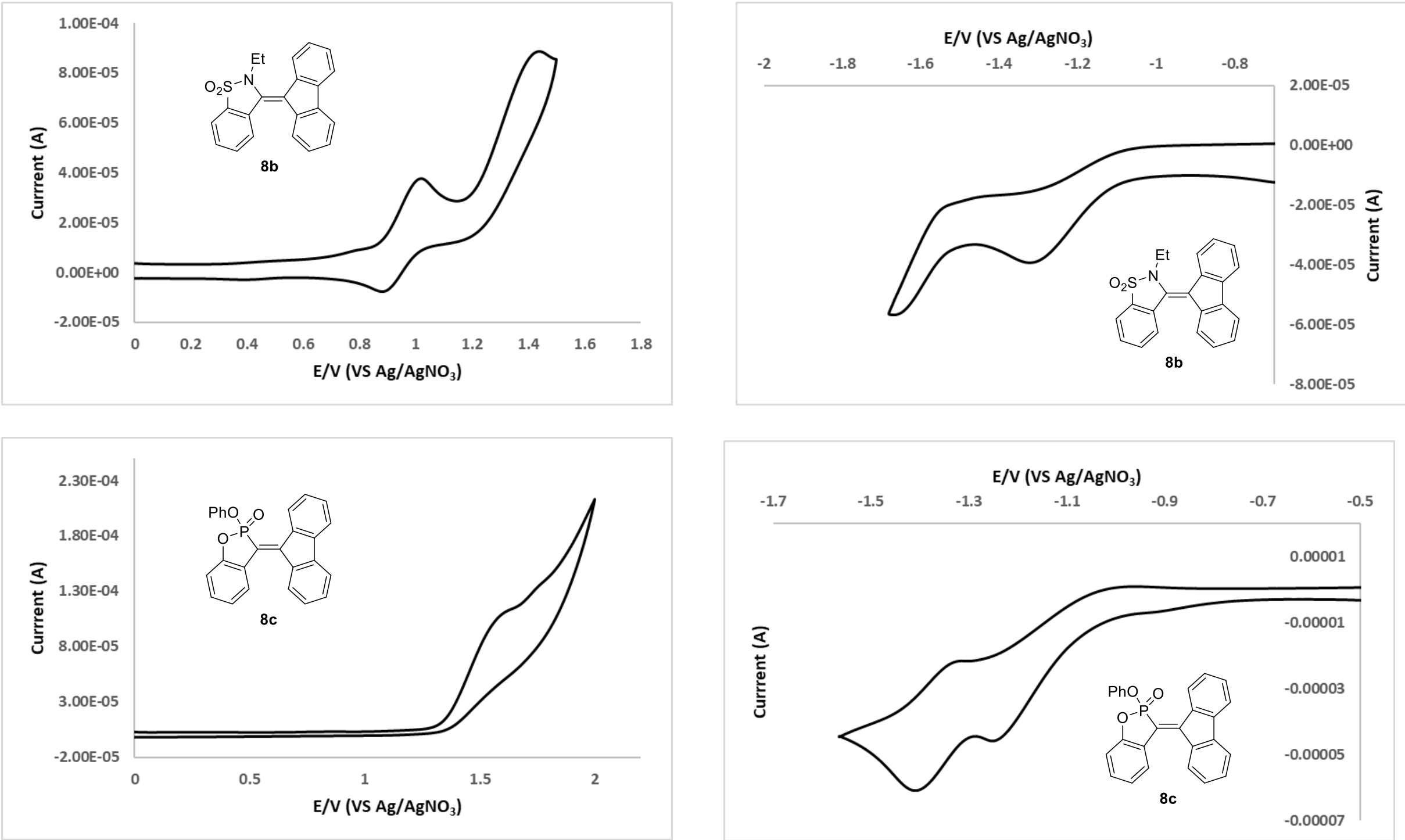

Figure S3. Cyclic voltammograms (CV) of products. First oxidation potentials measured by CV using Ag/$AgNO_3$ as a reference electrode, Pt wire as a counter electrode, glassy carbon as a working electrode, and Bu_4NPF_6 (0.1 M) as a supporting electrolyte in dichloromethane. The scan rate is 50 mV s^{-1} and the Fc/Fc^+ (-4.80 eV) was used as an external standard.

Table S2. Summary of photophysical and electrochemical of **8a-8c**.

Compound	λ_{max} (nm)[a]	λ_{onset} (nm)[a]	ΔE_{opt} (eV)[b]	$HOMO_{cv}$ (eV)[c]	$LUMO_{cv}$ (eV)[c]	ΔE_{cv} (eV)[d]
**2a**	496	582	2.03	-5.70	-3.53	2.17
**8a**	446	523	2.37	-5.87	-3.55	2.32
**8b**	426	504	2.48	-5.44	-2.96[e]	2.48[b]
**8c**	400	450	2.76	-5.96	-3.57	2.39

[a] UV-Vis absorption was measured in CH_2Cl_2 solution. λ_{max} was maximum absorption of the long wavelength feature.

[b] Optical energy gap (ΔE_{opt}) was estimated from λ_{onset} according to the equation, ΔE_{opt} (eV)=1240/λ_{onset}.

[c] HOMO and LUMO were calculated from the oxidation and reduction potentials by CV measurement and the Fc/Fc+ (−4.80 eV) was used as an external standard.

[d] HOMO–LUMO energy gap (ΔE_{cv}) was calculated from the HOMO and LUMO energy levels by CV measurement.

[e] Estimated by optical energy gap (ΔE_{opt}) and HOMO energy level.

9. DFT calculations

9.1 DFT calculation data for the reaction mechanism: Path A

Density functional theory (DFT) calculations were performed using the Gaussian16[a] program package unless otherwise noted. Geometry optimizations for intermediates and transition states were carried out at the M06/def2-SVP level of theory. For all transition states, intrinsic reaction coordinate (IRC) calculations were performed to confirm the connection between the reactant and product.

[a] Frisch, M. J.; Trucks, G. W.; Schlegel, H. B.; Scuseria, G. E.; Robb, M. A.; Cheeseman, J. R.; Scalmani, G.; Barone, V.; Petersson, G. A.; Nakatsuji, H.; Li, X.; Caricato, M.; Marenich, A. V.; Bloino, J.; Janesko, B. G.; Gomperts, R.; Mennucci, B.; Hratchian, H. P.; Ortiz, J. V.; Izmaylov, A. F.; Sonnenberg, J. L.; Williams-Young, D.; Ding, F.; Lipparini, F.; Egidi, F.; Goings, J.; Peng, B.; Petrone, A.; Henderson, T.; Ranasinghe, D.; Zakrzewski, V. G.; Gao, J.; Rega, N.; Zheng, G.; Liang, W.; Hada, M.; Ehara, M.; Toyota, K.; Fukuda, R.; Hasegawa, J.; Ishida, M.; Nakajima, T.; Honda, Y.; Kitao, O.; Nakai, H.; Vreven, T.; Throssell, K.; Montgomery, J. A., Jr.; Peralta, J. E.; Ogliaro, F.; Bearpark, M. J.; Heyd, J. J.; Brothers, E. N.; Kudin, K. N.; Staroverov, V. N.; Keith, T. A.; Kobayashi, R.; Normand, J.; Raghavachari, K.; Rendell, A. P.; Burant, J. C.; Iyengar, S. S.; Tomasi, J.; Cossi, M.; Millam, J. M.; Klene, M.; Adamo, C.; Cammi, R.; Ochterski, J. W.; Martin, R. L.; Morokuma, K.; Farkas, O.; Foresman, J. B.; Fox, D. J. *Gaussian 16*, Revision C.02; Gaussian, Inc.: Wallingford, CT, 2019.

Cartesian coordinates

****

Structure: IM1A

Charge = 0, Multiplicity = 1

Final SCF Energy = -1911.02567460 Hartree

Gibbs Free Energy = -1910.629410 Hartree

Thermal Correction = 0.396265 Hartree

Imaginary Frequencies = none

Cartesian Coordinates (Angstrom):

C	-1.521515	2.622343	-1.415915
O	-2.235561	2.967108	-2.331493
N	-0.706887	3.454476	-0.704182
C	-1.526451	1.200744	-1.006226
C	-1.976714	0.046046	-0.898350
C	-2.752755	-1.149039	-0.784903
C	-2.771271	-1.853912	0.439323
C	-3.575376	-1.552803	-1.847630
C	-3.655783	-2.924303	0.584602
C	-4.425831	-2.638211	-1.690095
H	-3.541722	-0.991499	-2.784866
C	-4.471373	-3.318087	-0.472285

H	-3.677632	-3.468956	1.533760
H	-5.064228	-2.953333	-2.519432
H	-5.143459	-4.171637	-0.348478
C	-1.877506	-1.434101	1.534289
C	-0.474152	-1.419008	1.343074
C	-2.397569	-1.042600	2.767866
C	0.369176	-0.986842	2.378741
H	-0.039119	-1.996639	0.498408
C	-1.546169	-0.623850	3.790941
H	-3.481611	-1.050196	2.919674
C	-0.167070	-0.585056	3.597635
H	1.450320	-0.978631	2.209436
H	-1.971151	-0.313100	4.750191
H	0.492241	-0.248500	4.402704
C	0.042359	3.044444	0.438566
C	1.415702	2.814091	0.325432
C	-0.588007	2.940532	1.681560
C	2.156435	2.483807	1.456817
H	1.882751	2.867150	-0.662738
C	0.159617	2.615689	2.809947
H	-1.664897	3.126981	1.749409
C	1.531450	2.392277	2.698823
H	3.226934	2.283674	1.360406
H	-0.332878	2.540784	3.784089
H	2.117748	2.139381	3.587798
C	-0.591175	4.842476	-1.105247
H	-1.238913	5.010665	-1.975095
H	-0.899896	5.511012	-0.284219
H	0.450665	5.082517	-1.376072
Pd	0.188141	-0.025770	-0.511239
Cl	0.949055	0.777900	-2.545402
O	2.098458	-0.558959	-0.117989
C	2.402453	-1.767927	-0.482652
O	1.584019	-2.623073	-0.789653
C	3.914746	-2.029475	-0.484051
C	4.182380	-3.480092	-0.855749
H	5.268805	-3.674112	-0.860186
H	3.707959	-4.171635	-0.141406
H	3.781302	-3.715540	-1.853599
C	4.472784	-1.732289	0.906905
H	5.560221	-1.920325	0.928718
H	4.298093	-0.682663	1.192687

H	4.007968	-2.381168	1.670271
C	4.548151	-1.093650	-1.513408
H	4.117436	-1.257407	-2.515638
H	4.383319	-0.037578	-1.248031
H	5.634993	-1.277291	-1.574694

****

Structure: $TS_{1\text{-}2}A$
Charge = 0, Multiplicity = 1
Final SCF Energy = -1911.01315114 Hartree
Gibbs Free Energy = -1910.618671 Hartree
Thermal Correction = 0.394480 Hartree
Imaginary Frequencies = -1580.97 cm^-1
Cartesian Coordinates (Angstrom):

C	-3.251865	0.436666	-1.048497
O	-4.095534	0.020673	-1.811686
N	-3.313652	1.634445	-0.397319
C	-2.071734	-0.410124	-0.767186
C	-1.432949	-1.476918	-0.702448
C	-0.924359	-2.804717	-0.573302
C	-0.162885	-3.141492	0.567892
C	-1.236507	-3.775564	-1.536372
C	0.249796	-4.468329	0.722695
C	-0.801622	-5.082663	-1.366239
H	-1.830111	-3.485140	-2.407014
C	-0.060069	-5.427874	-0.235375
H	0.851232	-4.739233	1.595872
H	-1.042280	-5.838552	-2.118236
H	0.289462	-6.455693	-0.104569
C	0.160774	-2.107455	1.564488
C	0.650511	-0.832748	1.169921
C	0.000775	-2.390444	2.925624
C	0.945134	0.110766	2.171233
H	1.611541	-0.886137	0.303424
C	0.293733	-1.432766	3.892974
H	-0.388438	-3.367904	3.228098
C	0.768642	-0.177067	3.518884
H	1.344077	1.087273	1.872826
H	0.147748	-1.671771	4.950525
H	1.005218	0.574560	4.278220
C	-2.369747	2.066734	0.581382
C	-1.350236	2.955486	0.227759

C	-2.522142	1.664103	1.910033
C	-0.495373	3.449068	1.210238
H	-1.225389	3.226681	-0.825471
C	-1.665466	2.162552	2.887696
H	-3.326324	0.966082	2.164648
C	-0.658540	3.061737	2.539513
H	0.307711	4.136971	0.930316
H	-1.787299	1.848516	3.928551
H	0.010301	3.457544	3.310490
C	-4.399567	2.545248	-0.700546
H	-5.049369	2.078113	-1.451523
H	-4.987889	2.765350	0.206204
H	-4.006417	3.495640	-1.099193
Pd	0.015334	0.150942	-0.696824
Cl	-0.592193	1.224309	-2.686991
O	1.812433	1.185894	-0.743844
C	2.859940	0.490158	-0.605776
O	2.851676	-0.718320	-0.267694
C	4.195199	1.213877	-0.765702
C	5.315728	0.214205	-1.019082
H	6.276648	0.747931	-1.109754
H	5.396837	-0.516434	-0.200464
H	5.147888	-0.347356	-1.952331
C	4.424554	1.928887	0.570024
H	5.398264	2.447226	0.557413
H	3.637455	2.678129	0.758168
H	4.432266	1.209832	1.407564
C	4.118426	2.230614	-1.898300
H	3.904066	1.741263	-2.862390
H	3.327830	2.974044	-1.719737
H	5.082953	2.757729	-1.991048

****

Structure: IM2A

Charge = 0, Multiplicity = 1

Final SCF Energy = -1911.05281529 Hartree

Gibbs Free Energy = -1910.656396 Hartree

Thermal Correction = 0.396420 Hartree

Imaginary Frequencies = none

Cartesian Coordinates (Angstrom):

C	3.329804	0.256899	-0.943399
O	4.039864	0.847210	-1.729160

N	3.649841	-0.929463	-0.346861
C	2.022805	0.846726	-0.593088
C	1.180662	1.765252	-0.519081
C	0.439080	2.984380	-0.494278
C	-0.603683	3.157044	0.442910
C	0.757646	3.988107	-1.419733
C	-1.323320	4.355673	0.390605
C	0.033315	5.172611	-1.438010
H	1.577759	3.814510	-2.121878
C	-1.013759	5.348268	-0.534644
H	-2.161241	4.508882	1.076178
H	0.278058	5.953435	-2.162645
H	-1.603818	6.268936	-0.553645
C	-0.878536	2.110180	1.448092
C	-0.568331	0.750569	1.248433
C	-1.450602	2.477847	2.680958
C	-0.826594	-0.183031	2.251479
H	-1.224204	-1.493334	-2.608356
C	-1.711057	1.545220	3.675785
H	-1.664534	3.533428	2.874807
C	-1.397744	0.205212	3.461999
H	-0.596374	-1.239943	2.081680
H	-2.148383	1.869094	4.624391
H	-1.596786	-0.543239	4.235591
C	2.874247	-1.536217	0.683549
C	2.124302	-2.683243	0.407373
C	2.926362	-1.029342	1.983858
C	1.440205	-3.324740	1.436421
H	2.069997	-3.042897	-0.624668
C	2.234531	-1.671375	3.007941
H	3.518690	-0.130028	2.180577
C	1.501000	-2.824798	2.737120
H	0.851549	-4.220575	1.218892
H	2.272332	-1.269396	4.024373
H	0.965709	-3.333711	3.544375
C	4.864075	-1.612159	-0.747080
H	5.371308	-1.004331	-1.507053
H	5.534852	-1.751113	0.117189
H	4.632350	-2.603339	-1.171827
Pd	0.107038	-0.099449	-0.458805
Cl	0.753820	-1.247988	-2.535282
O	-1.642552	-1.328049	-0.306786

C	-2.461134	-1.643651	-1.173826
O	-2.204274	-1.674513	-2.443254
C	-3.887302	-1.957037	-0.766343
C	-4.661500	-2.642652	-1.883217
H	-5.692184	-2.842683	-1.547359
H	-4.710211	-2.018632	-2.788298
H	-4.201928	-3.604204	-2.163484
C	-4.517046	-0.596075	-0.437697
H	-5.559789	-0.741510	-0.110164
H	-3.963905	-0.088879	0.370541
H	-4.527194	0.063686	-1.321524
C	-3.867425	-2.825934	0.489138
H	-3.386342	-3.799687	0.298567
H	-3.326358	-2.329141	1.308943
H	-4.901242	-3.018218	0.819652

****

Structure: IM3A

Charge = 0, Multiplicity = 1

Final SCF Energy = -1450.36563441 Hartree

Gibbs Free Energy = -1449.977706 Hartree

Thermal Correction = 0.387929 Hartree

Imaginary Frequencies = none

Cartesian Coordinates (Angstrom):

C	0.292074	2.997661	-0.021311
O	0.027304	4.009508	-0.636894
N	1.384502	2.843963	0.787126
C	-0.606612	1.836964	-0.157015
C	-1.650865	1.221770	-0.458124
C	-2.976603	0.847226	-0.829550
C	-3.524961	-0.375513	-0.385653
C	-3.709093	1.715857	-1.651466
C	-4.809490	-0.703294	-0.834140
C	-4.987970	1.370720	-2.066306
H	-3.248592	2.658612	-1.960433
C	-5.530626	0.152012	-1.661664
H	-5.250804	-1.661252	-0.546783
H	-5.556050	2.044703	-2.712563
H	-6.528681	-0.141575	-1.999030
C	-2.767591	-1.242307	0.539703
C	-1.362876	-1.231926	0.639271
C	-3.471006	-2.119990	1.385247

C	-0.708514	-2.084913	1.527933
C	-2.821296	-2.960579	2.279166
H	-4.564784	-2.120786	1.359052
C	-1.431534	-2.949564	2.348075
H	0.385393	-2.090492	1.570578
H	-3.404509	-3.622518	2.925341
H	-0.902168	-3.614058	3.037698
C	1.574004	1.718543	1.637834
C	2.690306	0.895660	1.466851
C	0.669361	1.461206	2.670852
C	2.885966	-0.190281	2.314337
H	3.378087	1.098986	0.640414
C	0.865996	0.369077	3.511735
H	-0.193156	2.121888	2.803130
C	1.975092	-0.455523	3.337292
H	3.753552	-0.841532	2.168479
H	0.147268	0.162797	4.309753
H	2.130327	-1.311890	3.999928
C	2.359422	3.914851	0.856599
H	2.030586	4.727610	0.196797
H	2.444586	4.295110	1.887989
H	3.352502	3.565592	0.529754
Pd	-0.025980	-0.193064	-0.432849
O	1.356663	-1.767683	-0.796137
C	2.175632	-0.985073	-1.374837
O	1.895855	0.238366	-1.484876
C	3.466446	-1.530561	-1.959483
C	4.608562	-0.572022	-1.632999
H	5.544446	-0.926751	-2.096800
H	4.396246	0.441722	-2.006282
H	4.780093	-0.508917	-0.543716
C	3.263906	-1.593653	-3.476199
H	4.175237	-1.980392	-3.963241
H	2.427764	-2.264194	-3.736090
H	3.046513	-0.594994	-3.887278
C	3.763722	-2.919039	-1.410458
H	3.878156	-2.900245	-0.313841
H	2.953494	-3.626002	-1.644869
H	4.700774	-3.303595	-1.847703

****

Structure: $TS_{3\text{-}4}A$

Charge = 0, Multiplicity = 1
Final SCF Energy = -1450.34725653 Hartree
Gibbs Free Energy = -1449.958295 Hartree
Thermal Correction = 0.388962 Hartree
Imaginary Frequencies = -263.25 cm^-1
Cartesian Coordinates (Angstrom):

C	-0.197319	2.858336	-0.711617
O	-0.830348	3.554647	-1.482617
N	1.014251	3.195801	-0.174615
C	-0.722696	1.533387	-0.338403
C	-1.763372	0.794596	-0.199039
C	-3.092971	0.489264	-0.656313
C	-3.634152	-0.720661	-0.192922
C	-3.772010	1.289099	-1.579388
C	-4.876146	-1.135530	-0.672283
C	-5.018321	0.871323	-2.033394
H	-3.308874	2.218931	-1.924743
C	-5.560379	-0.337995	-1.587273
H	-5.309265	-2.083739	-0.339571
H	-5.571561	1.486202	-2.748361
H	-6.533607	-0.665331	-1.964093
C	-2.805177	-1.347314	0.834831
C	-1.499571	-0.837773	1.003387
C	-3.304614	-2.254859	1.773687
C	-0.786443	-1.128917	2.172068
C	-2.551432	-2.620299	2.884207
H	-4.323653	-2.636876	1.654883
C	-1.304397	-2.038062	3.095739
H	0.201017	-0.690032	2.353329
H	-2.957048	-3.331806	3.608610
H	-0.718786	-2.293759	3.983378
C	1.620393	2.431424	0.863080
C	2.860167	1.824230	0.652717
C	0.983745	2.309665	2.101833
C	3.446293	1.079614	1.670850
H	3.335097	1.902027	-0.329164
C	1.573052	1.558763	3.116241
H	0.020819	2.806196	2.259416
C	2.803665	0.940909	2.901276
H	4.411106	0.593247	1.499124
H	1.068284	1.461982	4.081948
H	3.268580	0.353389	3.698012

C	1.657263	4.427493	-0.584611
H	1.034603	4.904699	-1.352281
H	1.768927	5.114166	0.270968
H	2.657174	4.227688	-1.003451
Pd	0.057433	-0.329478	-0.295851
O	1.250275	-2.166228	-0.462176
C	2.154323	-1.511676	-1.050020
O	1.987653	-0.274439	-1.273659
C	3.423253	-2.214402	-1.501256
C	4.463381	-1.204424	-1.964941
H	5.378609	-1.727514	-2.290659
H	4.090575	-0.599700	-2.806075
H	4.737407	-0.509288	-1.153580
C	3.038294	-3.143387	-2.655084
H	3.926269	-3.694031	-3.009932
H	2.279508	-3.874673	-2.335102
H	2.630068	-2.572508	-3.506000
C	3.963902	-3.040023	-0.335486
H	4.228459	-2.397528	0.522007
H	3.218342	-3.772428	0.009034
H	4.873228	-3.582440	-0.645926

****

Structure: IM4aA

Charge = 0, Multiplicity = 1

Final SCF Energy = -1450.39297324 Hartree

Gibbs Free Energy = -1450.002276 Hartree

Thermal Correction = 0.390697 Hartree

Imaginary Frequencies = none

Cartesian Coordinates (Angstrom):

C	-0.332362	2.085435	-0.564628
O	-0.879542	2.646076	-1.497581
N	0.757042	2.624604	0.082806
C	-0.802299	0.754385	-0.088090
C	-2.077478	0.305885	-0.020478
C	-3.410360	0.740910	-0.476732
C	-4.295429	-0.366341	-0.358328
C	-3.860218	1.939709	-1.023980
C	-5.621302	-0.258154	-0.762986
C	-5.196471	2.039312	-1.413645
H	-3.167901	2.773519	-1.159412
C	-6.068480	0.955800	-1.284894

H	-6.300579	-1.112320	-0.676572
H	-5.563674	2.979155	-1.835823
H	-7.109521	1.057608	-1.604889
C	-3.565860	-1.502015	0.217003
C	-2.261979	-1.052566	0.477155
C	-3.903766	-2.810957	0.546611
C	-1.316314	-1.845371	1.124084
C	-2.932180	-3.641592	1.122471
H	-4.913096	-3.194366	0.366047
C	-1.650648	-3.173048	1.419580
H	-0.423922	-1.380592	1.634328
H	-3.193404	-4.672774	1.376186
H	-0.928376	-3.825970	1.916220
C	1.340646	2.042094	1.234453
C	2.713571	1.774720	1.267809
C	0.558516	1.752073	2.359528
C	3.281765	1.173948	2.385982
H	3.324321	2.002454	0.390290
C	1.131984	1.142197	3.472888
H	-0.504265	2.010920	2.352679
C	2.492806	0.843410	3.487784
H	4.353658	0.952881	2.393422
H	0.509416	0.914436	4.343032
H	2.942437	0.366770	4.363061
C	1.425146	3.748655	-0.544037
H	0.689826	4.313100	-1.131912
H	1.862229	4.400458	0.226146
H	2.224402	3.413248	-1.229591
Pd	0.517494	-0.713797	0.079659
O	2.345608	-1.943808	-0.261117
C	2.775824	-1.003504	-0.969928
O	2.048080	0.034638	-1.145069
C	4.120211	-1.076717	-1.669229
C	4.819463	0.277600	-1.592060
H	5.781892	0.234756	-2.129246
H	4.206370	1.072965	-2.044504
H	5.032812	0.558647	-0.545904
C	3.829410	-1.427688	-3.131846
H	4.774853	-1.506808	-3.694855
H	3.304372	-2.394348	-3.208971
H	3.204697	-0.655766	-3.608990
C	4.981786	-2.157850	-1.031274

H	5.177041	-1.939123	0.031588
H	4.492166	-3.142016	-1.081584
H	5.951162	-2.221621	-1.553811

****

Structure: IM4bB
Charge = 0, Multiplicity = 1
Final SCF Energy = -1450.41484067 Hartree
Gibbs Free Energy = -1450.027282 Hartree
Thermal Correction = 0.387559 Hartree
Imaginary Frequencies = none
Cartesian Coordinates (Angstrom):

C	2.285090	-2.085925	-0.680012
O	3.315231	-1.735321	-1.210761
N	2.003356	-3.391872	-0.299763
C	1.072830	-1.272589	-0.295493
C	0.983723	0.055676	-0.522241
C	2.013907	0.926352	-1.088281
C	2.592086	1.884009	-0.224216
C	2.503651	0.797631	-2.395102
C	3.702905	2.618301	-0.640457
C	3.576315	1.571663	-2.815012
H	2.050109	0.059324	-3.061555
C	4.188659	2.467389	-1.936007
H	4.159565	3.337975	0.046955
H	3.957633	1.461357	-3.833823
H	5.042511	3.063660	-2.269583
C	1.959938	2.052400	1.096794
C	0.622364	2.521202	1.141832
C	2.582715	1.670773	2.285246
C	-0.084928	2.537190	2.360118
C	1.870773	1.693488	3.483448
H	3.612716	1.302167	2.255493
C	0.534374	2.099476	3.523919
H	-1.116126	2.904146	2.372213
H	2.359893	1.359806	4.403424
H	-0.014593	2.086401	4.468792
C	0.760674	-3.480863	0.317739
C	0.150841	-2.209542	0.355197
C	0.157831	-4.608909	0.856113
C	-1.083558	-2.076304	0.982554
C	-1.092370	-4.453461	1.462410

H	0.647888	-5.585654	0.811748
C	-1.703301	-3.202797	1.530666
H	-1.569275	-1.097701	1.051622
H	-1.590656	-5.325837	1.895327
H	-2.676109	-3.097425	2.019166
C	2.909677	-4.485705	-0.491901
H	3.807401	-4.094577	-0.990297
H	3.203147	-4.938948	0.470984
H	2.461911	-5.271950	-1.123786
Pd	-0.623639	1.044687	-0.005826
O	-2.749000	1.721109	0.139630
C	-3.050930	0.786540	-0.648706
O	-2.138169	0.040979	-1.128735
C	-4.497719	0.462673	-0.975808
C	-4.629810	0.098554	-2.451385
H	-5.673482	-0.178098	-2.678323
H	-4.356111	0.947449	-3.099925
H	-3.978971	-0.749634	-2.711600
C	-5.399673	1.638759	-0.629515
H	-6.450439	1.383490	-0.848622
H	-5.320451	1.905705	0.435276
H	-5.135081	2.533585	-1.216210
C	-4.855401	-0.750096	-0.109440
H	-4.207198	-1.609646	-0.347082
H	-4.742435	-0.515631	0.963504
H	-5.904101	-1.045514	-0.284039
H	0.247691	3.103613	0.280853

****

Structure: $TS_{4\text{-}5}A$

Charge = 0, Multiplicity = 1

Final SCF Energy = -1450.37247926 Hartree

Gibbs Free Energy = -1449.988975 Hartree

Thermal Correction = 0.383504 Hartree

Imaginary Frequencies = -1130.55 cm^-1

Cartesian Coordinates (Angstrom):

C	0.822361	-1.875672	-1.077770
O	1.282857	-2.312647	-2.112112
N	0.140151	-2.709038	-0.180273
C	0.939748	-0.466932	-0.641372
C	2.091163	0.209840	-0.417522
C	3.495889	-0.247091	-0.570949

C	4.354448	0.780733	-0.116171
C	4.041932	-1.432542	-1.063002
C	5.735719	0.619433	-0.125131
C	5.428703	-1.589182	-1.065742
H	3.403451	-2.218527	-1.469415
C	6.270090	-0.577644	-0.599412
H	6.391986	1.419891	0.231115
H	5.861194	-2.516904	-1.451542
H	7.354368	-0.721531	-0.615486
C	3.541571	1.919700	0.303724
C	2.183540	1.588860	0.114666
C	3.911633	3.162683	0.808140
C	1.198587	2.519907	0.437924
C	2.916239	4.085631	1.126503
H	4.967350	3.413509	0.951777
C	1.568956	3.766268	0.942314
H	0.127781	2.292717	0.310676
H	3.190676	5.068035	1.521440
H	0.796440	4.498656	1.192581
C	-0.424318	-2.207345	0.987393
C	-1.208345	-1.019577	0.942008
C	-0.256297	-2.879372	2.206124
C	-1.660007	-0.464987	2.166661
C	-0.756122	-2.328909	3.377374
H	0.317462	-3.809291	2.238190
C	-1.444225	-1.107957	3.370623
H	-2.255693	0.454404	2.130147
H	-0.591490	-2.852402	4.323829
H	-1.828093	-0.686612	4.303025
C	0.209234	-4.139105	-0.407771
H	0.319321	-4.304205	-1.487580
H	1.077001	-4.601391	0.095195
H	-0.713120	-4.619518	-0.051486
Pd	-0.824456	0.392377	-0.548897
O	-2.793616	1.222430	-0.607480
C	-3.730843	0.373827	-0.554896
O	-3.561934	-0.850759	-0.335499
C	-5.164514	0.894357	-0.674276
C	-5.584079	1.245413	0.757058
H	-6.624501	1.612801	0.769244
H	-5.525577	0.359969	1.412556
H	-4.938013	2.035270	1.176150

C	-6.080768	-0.188945	-1.229154
H	-7.117549	0.184877	-1.279422
H	-5.777816	-0.487151	-2.246523
H	-6.062360	-1.090195	-0.598769
C	-5.220348	2.138274	-1.551337
H	-4.583045	2.941194	-1.152197
H	-4.881496	1.920427	-2.577793
H	-6.257224	2.510910	-1.609497
H	-2.205257	-1.041184	0.159660

****

Structure: IM5A

Charge = 0, Multiplicity = 1

Final SCF Energy = -1450.40673539 Hartree

Gibbs Free Energy = -1450.018045 Hartree

Thermal Correction = 0.388690 Hartree

Imaginary Frequencies = none

Cartesian Coordinates (Angstrom):

C	-0.951284	1.898435	-1.079857
O	-1.411756	2.346902	-2.113796
N	-0.303467	2.715571	-0.154831
C	-1.001073	0.469241	-0.697987
C	-2.127180	-0.240059	-0.442937
C	-3.543325	0.191944	-0.563932
C	-4.374953	-0.850808	-0.094381
C	-4.122775	1.365914	-1.047117
C	-5.759366	-0.717188	-0.078678
C	-5.512071	1.495719	-1.024297
H	-3.507182	2.163703	-1.465588
C	-6.325976	0.468726	-0.542856
H	-6.392473	-1.530987	0.289558
H	-5.968930	2.415442	-1.401465
H	-7.413052	0.591818	-0.538981
C	-3.533881	-1.971903	0.314487
C	-2.184407	-1.618564	0.098930
C	-3.876114	-3.214559	0.838925
C	-1.179659	-2.525686	0.428725
C	-2.861253	-4.116347	1.155808
H	-4.925806	-3.479330	1.001691
C	-1.522994	-3.772226	0.952423
H	-0.121916	-2.266690	0.282809
H	-3.113183	-5.097901	1.567972

H	-0.734393	-4.486564	1.206547
C	0.274470	2.178488	1.004732
C	0.753488	0.852099	1.009633
C	0.398432	2.947918	2.169583
C	1.233995	0.294978	2.196876
C	0.936349	2.396611	3.329598
H	0.041484	3.981201	2.178491
C	1.344333	1.066893	3.355891
H	1.564032	-0.751327	2.210605
H	1.019223	3.014880	4.227973
H	1.754317	0.627739	4.269617
C	-0.300475	4.142241	-0.381292
H	-0.570471	4.316427	-1.430530
H	-1.034594	4.668376	0.254346
H	0.699082	4.560925	-0.182604
Pd	0.830449	-0.257182	-0.658968
O	2.904761	-1.021007	-0.784919
C	3.904002	-0.426210	-0.400247
O	3.847364	0.698416	0.276648
C	5.313197	-0.921584	-0.650178
C	5.963296	-1.205207	0.707610
H	6.986594	-1.583400	0.549794
H	6.022855	-0.297081	1.326597
H	5.404458	-1.972817	1.268462
C	6.094825	0.173627	-1.380463
H	7.119763	-0.180729	-1.577651
H	5.632339	0.420307	-2.350475
H	6.157856	1.095351	-0.782671
C	5.272068	-2.190179	-1.489390
H	4.710601	-2.991475	-0.984517
H	4.794928	-2.013494	-2.466053
H	6.299084	-2.547504	-1.667524
H	2.906005	0.936116	0.448805

****

Structure: IM6A
Charge = 0, Multiplicity = 1
Final SCF Energy = -1600.22424946 Hartree
Gibbs Free Energy = -1599.780805 Hartree
Thermal Correction = 0.443444 Hartree
Imaginary Frequencies = none
Cartesian Coordinates (Angstrom):

C	0.821107	1.628671	1.253597
O	1.338888	1.486122	2.359033
N	0.281546	2.829643	0.849269
C	0.696596	0.519495	0.285545
C	1.754828	-0.109449	-0.282877
C	3.205815	0.132206	-0.071337
C	3.938194	-0.826330	-0.811042
C	3.898232	1.077562	0.688252
C	5.330487	-0.844820	-0.793344
C	5.293045	1.059011	0.696032
H	3.367272	1.818865	1.287933
C	6.007878	0.106509	-0.034300
H	5.882864	-1.592476	-1.372221
H	5.834056	1.802748	1.288874
H	7.101684	0.109436	-0.010361
C	2.996782	-1.679745	-1.528274
C	1.686747	-1.239505	-1.235379
C	3.219077	-2.750654	-2.390624
C	0.600841	-1.869959	-1.842674
C	2.124391	-3.384367	-2.975933
H	4.238211	-3.085183	-2.610810
C	0.826225	-2.942276	-2.705757
H	-0.421083	-1.518559	-1.650591
H	2.281469	-4.225377	-3.657972
H	-0.024629	-3.437127	-3.183701
C	-0.468611	2.937280	-0.347235
C	-1.172012	1.826415	-0.852335
C	-0.547135	4.167241	-1.010631
C	-1.890184	1.966563	-2.039678
C	-1.298104	4.296552	-2.176504
H	0.000484	5.031326	-0.623639
C	-1.968836	3.195745	-2.698219
H	-2.427748	1.102188	-2.445253
H	-1.346643	5.265762	-2.681512
H	-2.559194	3.289838	-3.614863
C	0.430392	3.981512	1.708699
H	0.769949	3.632327	2.691901
H	1.175840	4.697746	1.320092
H	-0.532593	4.506428	1.814825
Pd	-1.217932	0.064263	0.068291
C	-3.991617	-1.255298	0.270549
O	-3.389202	-0.311746	-0.232228

N	-5.307429	-1.478945	0.107456
H	-3.472988	-2.003803	0.907190
C	-5.960348	-2.612938	0.711504
H	-6.764554	-2.288390	1.394451
H	-6.409867	-3.265545	-0.057009
H	-5.233770	-3.204731	1.286207
C	-6.122486	-0.591360	-0.690403
H	-6.946154	-0.176577	-0.084977
H	-5.499275	0.231227	-1.063018
H	-6.561377	-1.131444	-1.546694
C	-0.067563	-2.164405	1.562149
O	-1.232125	-1.955856	1.199096
N	0.471312	-1.753078	2.710488
H	0.645790	-2.729994	0.919300
C	1.900992	-1.814910	2.928935
H	2.377755	-2.408601	2.134513
H	2.315264	-0.791465	2.897654
H	2.125445	-2.274168	3.905814
C	-0.293104	-0.966403	3.656057
H	0.104139	0.061893	3.684761
H	-1.343862	-0.944150	3.340124
H	-0.219867	-1.413881	4.661292

****

Structure: $TS_{6\text{-}7}A$

Charge = 0, Multiplicity = 1

Final SCF Energy = -1600.21396713 Hartree

Gibbs Free Energy = -1599.771461 Hartree

Thermal Correction = 0.442506 Hartree

Imaginary Frequencies = -245.92 cm^-1

Cartesian Coordinates (Angstrom):

C	0.780696	1.580990	1.363920
O	1.202236	1.340187	2.486861
N	0.299257	2.813017	0.985794
C	0.664531	0.577700	0.267597
C	1.730842	-0.132235	-0.216105
C	3.168320	0.006720	0.105212
C	3.902168	-0.892873	-0.707944
C	3.859536	0.824499	1.004147
C	5.289836	-0.971640	-0.630504
C	5.250019	0.743433	1.072641
H	3.327402	1.509171	1.667089

C	5.964599	-0.145437	0.265371
H	5.841877	-1.670090	-1.268157
H	5.788108	1.385550	1.776553
H	7.055126	-0.192916	0.338674
C	2.964701	-1.626862	-1.549691
C	1.658048	-1.172252	-1.254229
C	3.180261	-2.620029	-2.500640
C	0.568878	-1.735302	-1.922436
C	2.084530	-3.167229	-3.167574
H	4.194773	-2.967310	-2.722415
C	0.789909	-2.727454	-2.877953
H	-0.453068	-1.409280	-1.684694
H	2.238089	-3.947695	-3.918813
H	-0.062238	-3.168282	-3.404022
C	-0.175469	2.883358	-0.331806
C	-0.503516	1.645280	-0.927151
C	-0.239291	4.072684	-1.053516
C	-0.773165	1.629052	-2.301272
C	-0.587381	4.036595	-2.404218
H	0.011069	5.022480	-0.571710
C	-0.830557	2.817622	-3.031716
H	-0.989335	0.679923	-2.802958
H	-0.644373	4.969629	-2.972249
H	-1.085309	2.788220	-4.095244
C	0.334413	3.943577	1.871977
H	0.619985	3.581601	2.868651
H	1.073716	4.697687	1.548516
H	-0.654745	4.427855	1.927000
Pd	-1.256100	0.055347	0.124476
C	-4.219630	-0.950481	0.133104
O	-3.472630	-0.101416	-0.336797
N	-5.537453	-1.032316	-0.138177
H	-3.848893	-1.730903	0.832037
C	-6.364206	-2.058962	0.442282
H	-7.178004	-1.619578	1.045569
H	-6.824132	-2.687949	-0.340094
H	-5.759539	-2.705375	1.094465
C	-6.174922	-0.090409	-1.028096
H	-6.981984	0.451762	-0.505994
H	-5.426572	0.630105	-1.381648
H	-6.615393	-0.611103	-1.895812
C	-0.504246	-2.149271	1.623838

O	-1.687903	-1.880346	1.388713
N	0.149817	-1.846181	2.750415
H	0.128820	-2.693221	0.884062
C	1.577940	-2.036529	2.869442
H	1.960474	-2.588019	1.997219
H	2.078802	-1.053886	2.908332
H	1.819362	-2.604270	3.783958
C	-0.493052	-1.082522	3.798284
H	-0.033481	-0.081944	3.860854
H	-1.560479	-0.981863	3.563884
H	-0.377185	-1.597415	4.766831

****

Structure: IM7A
Charge = 0, Multiplicity = 1
Final SCF Energy = -1600.26443750 Hartree
Gibbs Free Energy = -1599.816595 Hartree
Thermal Correction = 0.447842 Hartree
Imaginary Frequencies = none
Cartesian Coordinates (Angstrom):

C	-2.131149	1.570732	-0.207862
O	-1.453017	2.572501	-0.379780
N	-3.422370	1.623250	0.308157
C	-1.824353	0.112738	-0.406156
C	-0.588784	-0.402288	-0.917208
C	0.342524	0.250316	-1.875880
C	1.296994	-0.700648	-2.307443
C	0.345654	1.521197	-2.457477
C	2.272885	-0.378314	-3.246668
C	1.309043	1.830225	-3.417474
H	-0.392311	2.264399	-2.153910
C	2.277370	0.898535	-3.801927
H	3.005935	-1.127433	-3.562957
H	1.302792	2.823616	-3.878226
H	3.026905	1.166417	-4.552503
C	1.022067	-1.970873	-1.655402
C	-0.135146	-1.826911	-0.856960
C	1.730661	-3.172164	-1.700941
C	-0.540485	-2.907373	-0.061868
C	1.292499	-4.246253	-0.933086
H	2.623892	-3.261265	-2.328340
C	0.166939	-4.105751	-0.112733

H	-1.385501	-2.810931	0.624422
H	1.833295	-5.196682	-0.958808
H	-0.159153	-4.944914	0.508466
C	-4.019450	0.372999	0.301711
C	-3.094857	-0.589653	-0.160053
C	-5.331995	0.057299	0.623784
C	-3.558439	-1.882834	-0.398730
C	-5.752107	-1.261785	0.434864
H	-6.019939	0.823395	0.992725
C	-4.880958	-2.213936	-0.091307
H	-2.905821	-2.635958	-0.844924
H	-6.781321	-1.540046	0.679248
H	-5.234862	-3.232346	-0.274133
C	-4.071289	2.842759	0.689712
H	-3.354850	3.663161	0.547542
H	-4.962845	3.036134	0.067720
H	-4.384734	2.816334	1.747379
Pd	-0.351947	0.008821	1.154562
C	2.326217	-1.012100	1.743027
O	1.434450	-0.436029	2.372923
N	3.605927	-1.082283	2.121975
H	2.116120	-1.553859	0.793844
C	4.600727	-1.570310	1.197283
H	5.136936	-0.723137	0.735217
H	5.321531	-2.225945	1.712755
H	4.113928	-2.143007	0.393753
C	4.080480	-0.276398	3.221075
H	4.521585	0.660893	2.835609
H	3.240878	-0.028328	3.882896
H	4.845627	-0.826066	3.791896
C	2.877732	2.098768	-0.251847
O	3.320630	1.121449	0.323560
N	2.004149	2.995766	0.258125
H	3.172877	2.358537	-1.299170
C	1.504943	4.104581	-0.515316
H	1.962734	4.101110	-1.515836
H	0.409485	4.029349	-0.624317
H	1.746892	5.065505	-0.025796
C	1.445043	2.832165	1.578279
H	0.362845	2.606476	1.507609
H	1.947622	2.000951	2.090751
H	1.571988	3.759033	2.165410

9.2 DFT calculation data for the reaction mechanism: Path B

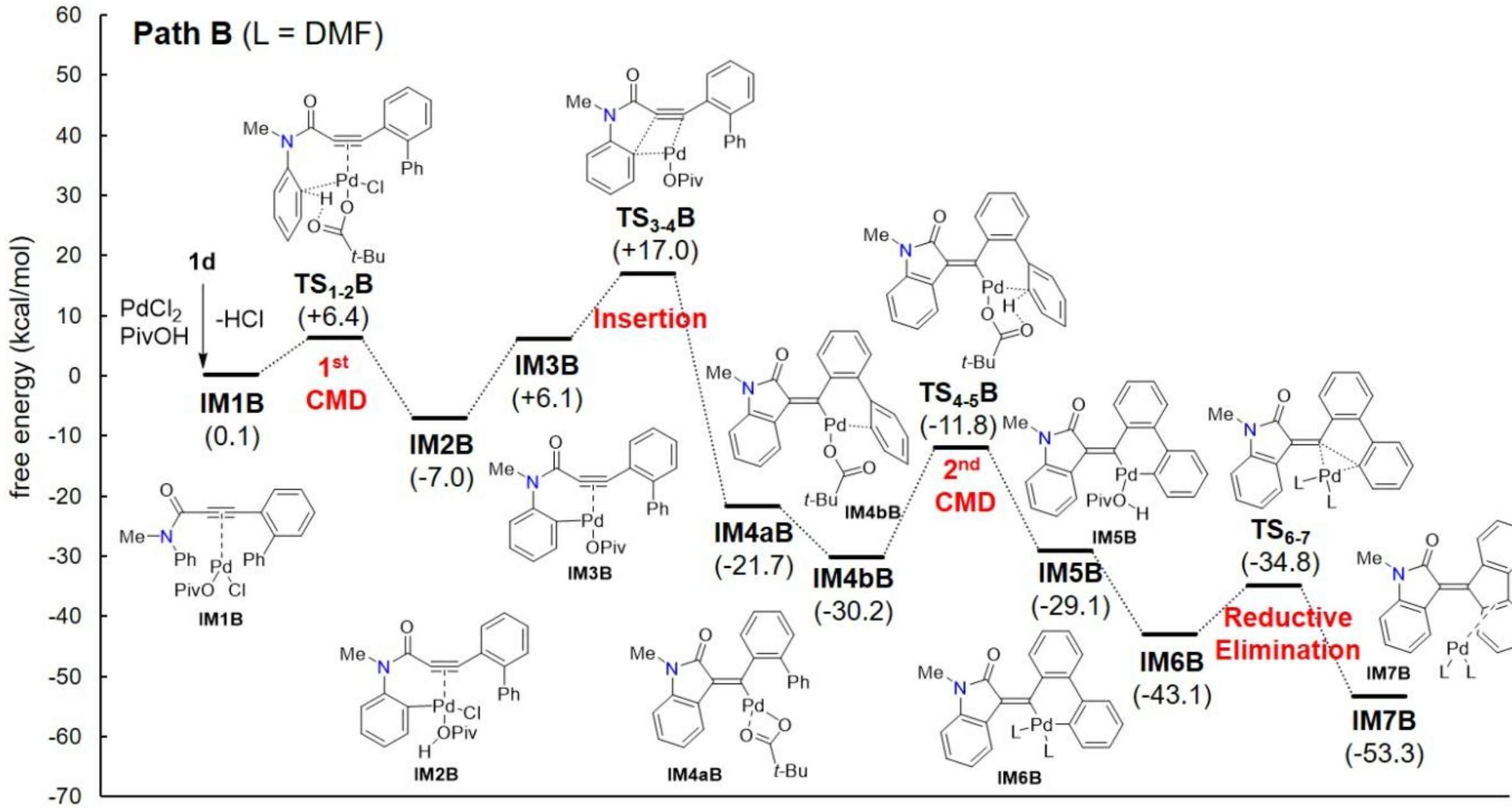

Figure S4. Alternative reaction mechanism Path B with energy diagram.

Cartesian coordinates

****

Structure: IM1B

Charge = 0, Multiplicity = 1

Final SCF Energy = -1911.02457577 Hartree

Gibbs Free Energy = -1910.629223 Hartree

Thermal Correction = 0.395353 Hartree

Imaginary Frequencies = none

Cartesian Coordinates (Angstrom):

C	-0.714376	3.055608	-1.082733
O	-0.968107	3.721607	-2.057675
N	-0.146498	3.538698	0.074139
C	-1.044250	1.618746	-1.012666
C	-1.741005	0.586559	-0.977682
C	-2.785125	-0.375666	-1.147826
C	-3.083610	-1.380666	-0.200449
C	-3.594000	-0.217023	-2.288235
C	-4.223296	-2.162569	-0.410035
C	-4.708890	-1.019135	-2.480101
H	-3.328217	0.558079	-3.012269
C	-5.029181	-1.988348	-1.531144
H	-4.461293	-2.941668	0.321104
H	-5.329710	-0.886917	-3.369894

H	-5.907437	-2.624780	-1.671246
C	-2.238068	-1.665753	0.984514
C	-0.952729	-2.204624	0.828842
C	-2.755983	-1.505138	2.276231
C	-0.200358	-2.565681	1.945337
H	-0.557821	-2.381623	-0.177359
C	-2.004484	-1.872906	3.389256
H	-3.764104	-1.094919	2.400097
C	-0.725953	-2.405512	3.225000
H	0.801494	-2.980709	1.801570
H	-2.423330	-1.748614	4.392719
H	-0.139866	-2.703602	4.100148
C	0.017888	2.679750	1.191310
C	1.044683	1.711806	1.177872
C	-0.876761	2.741041	2.253311
C	1.120394	0.785152	2.232740
H	1.867820	1.788743	0.443124
C	-0.769725	1.827936	3.303627
H	-1.677954	3.485975	2.238205
C	0.215480	0.844907	3.290308
H	1.904578	0.022828	2.211517
H	-1.485934	1.869906	4.129570
H	0.279006	0.119674	4.105011
C	0.137343	4.952706	0.205561
H	-0.000115	5.420008	-0.778491
H	-0.542055	5.439319	0.926746
H	1.175210	5.104804	0.540651
Pd	0.422381	0.150319	-0.509478
Cl	0.316319	-1.026646	-2.495997
O	2.067504	-0.953938	-0.087007
C	3.187717	-0.404949	-0.446413
O	3.315642	0.770981	-0.758037
C	4.362052	-1.390076	-0.508350
C	5.672621	-0.629856	-0.361835
H	6.525355	-1.321799	-0.470336
H	5.759009	0.159358	-1.123075
H	5.748994	-0.147383	0.627249
C	4.274478	-2.029408	-1.896926
H	5.084809	-2.767637	-2.027308
H	3.306901	-2.538652	-2.037219
H	4.371735	-1.265106	-2.685660
C	4.249451	-2.465559	0.566285

H	4.220048	-2.024692	1.578386
H	3.341461	-3.072939	0.433640
H	5.123764	-3.137479	0.519017

****

Structure: $TS_{1\text{-}2}B$

Charge = 0, Multiplicity = 1

Final SCF Energy = -1911.01357604 Hartree

Gibbs Free Energy = -1910.619229 Hartree

Thermal Correction = 0.394347 Hartree

Imaginary Frequencies = -1509.51 cm^-1

Cartesian Coordinates (Angstrom):

C	-0.555760	3.034492	-0.984869
O	-0.901456	3.801361	-1.853578
N	0.324380	3.378500	0.025048
C	-1.091418	1.674469	-0.897787
C	-1.909073	0.744900	-0.746061
C	-3.100493	-0.043173	-0.681279
C	-3.290086	-1.084444	0.253808
C	-4.141050	0.323127	-1.553296
C	-4.543831	-1.698423	0.307827
C	-5.372090	-0.312580	-1.487684
H	-3.958875	1.121235	-2.278055
C	-5.575353	-1.321202	-0.547960
H	-4.697848	-2.504107	1.032691
H	-6.174009	-0.020900	-2.170756
H	-6.542729	-1.827693	-0.486883
C	-2.218303	-1.565830	1.162899
C	-1.171302	-2.356678	0.668530
C	-2.286738	-1.305235	2.536237
C	-0.214123	-2.877112	1.537541
H	-1.120225	-2.573979	-0.403817
C	-1.325162	-1.823339	3.401122
H	-3.107760	-0.693055	2.924752
C	-0.289793	-2.613561	2.903808
H	0.596863	-3.491015	1.133786
H	-1.389058	-1.612235	4.472959
H	0.459715	-3.027499	3.585880
C	0.605119	2.523812	1.107559
C	0.983408	1.174242	0.890662
C	0.538060	3.031881	2.409135
C	1.218239	0.365298	2.021262

H	1.925660	1.024185	0.032719
C	0.786806	2.204283	3.498542
H	0.257858	4.076345	2.570165
C	1.117783	0.861573	3.312155
H	1.513664	-0.677820	1.860987
H	0.711213	2.614909	4.509722
H	1.309893	0.211904	4.170575
C	0.756949	4.763832	0.061551
H	0.855766	5.126505	-0.969890
H	0.028959	5.416198	0.574877
H	1.728040	4.829833	0.570724
Pd	0.201430	0.025452	-0.787850
Cl	-0.597669	-1.175966	-2.630272
O	1.879839	-1.193730	-0.761463
C	2.998709	-0.603704	-0.720194
O	3.130358	0.630081	-0.537035
C	4.246197	-1.482347	-0.794843
C	5.442900	-0.663723	-1.261705
H	6.343070	-1.300563	-1.285158
H	5.282503	-0.263131	-2.275853
H	5.633375	0.188349	-0.592331
C	4.015075	-2.669544	-1.721031
H	4.920273	-3.299140	-1.751959
H	3.170030	-3.286961	-1.382333
H	3.794044	-2.339214	-2.748966
C	4.474825	-1.969784	0.639869
H	4.595306	-1.119263	1.332761
H	3.630261	-2.588522	0.987553
H	5.391044	-2.582319	0.688048

****

Structure: IM2B

Charge = 0, Multiplicity = 1

Final SCF Energy = -1911.04017922 Hartree

Gibbs Free Energy = -1910.640508 Hartree

Thermal Correction = 0.399671 Hartree

Imaginary Frequencies = none

Cartesian Coordinates (Angstrom):

C	-0.709262	3.192608	-0.615602
O	-1.248564	4.042530	-1.291159
N	0.421611	3.422760	0.141466
C	-1.232107	1.830161	-0.571015

C	-2.033109	0.875048	-0.471405
C	-3.227043	0.090328	-0.395797
C	-3.334588	-1.077462	0.390612
C	-4.345691	0.580502	-1.091977
C	-4.582087	-1.700868	0.472833
C	-5.570296	-0.064911	-1.002037
H	-4.228001	1.480613	-1.701444
C	-5.688955	-1.206461	-0.212213
H	-4.672801	-2.604875	1.083460
H	-6.433141	0.323239	-1.549461
H	-6.650015	-1.722529	-0.133575
C	-2.192267	-1.678401	1.129637
C	-1.234999	-2.451670	0.457488
C	-2.113612	-1.553293	2.521454
C	-0.228390	-3.096978	1.174546
H	-1.290177	-2.547438	-0.632786
C	-1.100007	-2.191813	3.233052
H	-2.862123	-0.950997	3.047356
C	-0.160120	-2.972025	2.561245
H	0.504265	-3.707790	0.637883
H	-1.049321	-2.084701	4.320586
H	0.627412	-3.485359	3.121919
C	0.921928	2.489816	1.084696
C	0.818815	1.096185	0.892773
C	1.554540	2.976804	2.240599
C	1.303172	0.243876	1.889257
H	2.494715	1.096005	-0.599145
C	2.056375	2.108072	3.202635
H	1.637439	4.054261	2.401980
C	1.925425	0.732984	3.036127
H	1.200599	-0.839661	1.758812
H	2.541569	2.516361	4.093758
H	2.305599	0.038966	3.791877
C	0.954922	4.769280	0.103079
H	0.647255	5.236326	-0.840444
H	0.572048	5.396847	0.926919
H	2.053023	4.738400	0.161155
Pd	0.011816	0.164448	-0.730785
Cl	-0.866933	-0.918434	-2.671716
O	1.763179	-1.062013	-0.905177
C	2.931143	-0.693605	-0.933551
O	3.288738	0.564793	-0.830175

C	4.074375	-1.680495	-0.995001
C	5.289861	-1.080776	-1.693813
H	6.100909	-1.826555	-1.715965
H	5.059757	-0.801830	-2.734893
H	5.661757	-0.185165	-1.174407
C	3.620020	-2.952608	-1.699381
H	4.443719	-3.684562	-1.703572
H	2.752917	-3.405107	-1.195939
H	3.330596	-2.753083	-2.743443
C	4.402141	-1.979265	0.475466
H	4.705599	-1.066212	1.014020
H	3.530224	-2.414423	0.993020
H	5.231300	-2.703950	0.527207

****

Structure: IM3B
Charge = 0, Multiplicity = 1
Final SCF Energy = -1450.35813324 Hartree
Gibbs Free Energy = -1449.969293 Hartree
Thermal Correction = 0.388840 Hartree
Imaginary Frequencies = none
Cartesian Coordinates (Angstrom):

C	-0.193466	2.740709	-1.574758
O	-0.578962	3.190039	-2.633702
N	1.049665	2.969134	-1.046383
C	-1.065291	1.857707	-0.791058
C	-2.015680	1.220420	-0.305291
C	-3.249129	0.638197	0.139453
C	-3.381421	-0.767618	0.224641
C	-4.315419	1.472625	0.501771
C	-4.589537	-1.289819	0.691369
C	-5.507965	0.927445	0.961550
H	-4.186407	2.555981	0.430702
C	-5.642466	-0.455437	1.059523
H	-4.708830	-2.376243	0.746567
H	-6.333147	1.584951	1.247069
H	-6.578391	-0.891418	1.419401
C	-2.283649	-1.679237	-0.187367
C	-1.630597	-1.513384	-1.429992
C	-1.928322	-2.777506	0.612658
C	-0.653007	-2.423813	-1.847036
H	-1.962845	-0.731060	-2.120173

C	-0.955166	-3.674670	0.189395
H	-2.414619	-2.909537	1.584237
C	-0.314534	-3.500442	-1.037755
H	-0.154450	-2.274135	-2.807841
H	-0.679167	-4.514719	0.833206
H	0.458806	-4.204400	-1.359670
C	1.421630	2.587116	0.267995
C	0.975814	1.405667	0.880345
C	2.283674	3.425408	0.990952
C	1.363567	1.096029	2.180225
C	2.681294	3.105428	2.283985
H	2.637658	4.353631	0.534978
C	2.215942	1.942874	2.888674
H	1.029547	0.157075	2.631417
H	3.356106	3.777963	2.820743
H	2.524687	1.680501	3.904616
C	1.962106	3.759260	-1.849072
H	1.677494	3.655106	-2.903353
H	1.923657	4.833014	-1.595387
H	2.987402	3.389789	-1.705714
Pd	-0.178584	0.057255	-0.040071
O	1.245285	-1.299736	0.497902
C	2.201917	-1.379264	-0.372108
O	2.215258	-0.788087	-1.443338
C	3.397450	-2.228222	0.083067
C	4.145399	-2.748388	-1.136466
H	5.038774	-3.317456	-0.825955
H	3.506827	-3.417966	-1.738452
H	4.459464	-1.920790	-1.789407
C	2.969033	-3.388526	0.973102
H	3.853540	-3.964194	1.297764
H	2.435743	-3.034057	1.868207
H	2.298432	-4.081456	0.435716
C	4.289663	-1.270421	0.877717
H	4.580760	-0.404000	0.259640
H	3.763562	-0.890726	1.769846
H	5.208935	-1.784089	1.209799

****

Structure: $TS_{3\text{-}4}B$

Charge = 0, Multiplicity = 1

Final SCF Energy = -1450.34087228 Hartree

Gibbs Free Energy = -1449.951985 Hartree
Thermal Correction = 0.388888 Hartree
Imaginary Frequencies = -289.77 cm^-1
Cartesian Coordinates (Angstrom):

C	0.099265	-2.790604	-1.194766
O	0.658013	-3.326593	-2.123875
N	-1.256744	-2.780816	-0.977936
C	0.815629	-1.972682	-0.184673
C	1.861889	-1.249037	-0.034616
C	3.216094	-0.805850	0.059372
C	3.463715	0.586339	0.097710
C	4.280838	-1.717346	0.139182
C	4.778887	1.027555	0.234222
C	5.583164	-1.255010	0.275849
H	4.062787	-2.788263	0.108092
C	5.830691	0.116204	0.328460
H	4.977993	2.103726	0.258773
H	6.410131	-1.966436	0.345952
H	6.854999	0.482517	0.439966
C	2.355471	1.576656	-0.048492
C	1.626118	1.658128	-1.260178
C	2.144205	2.572713	0.926296
C	0.730379	2.710542	-1.481023
H	1.834738	0.943658	-2.064170
C	1.246498	3.607215	0.696473
H	2.707521	2.523252	1.863918
C	0.540369	3.680009	-0.506633
H	0.171757	2.746867	-2.420029
H	1.089849	4.366390	1.467485
H	-0.167910	4.495392	-0.676873
C	-1.689374	-2.290995	0.253290
C	-0.814088	-1.443883	0.954256
C	-2.869626	-2.722085	0.861788
C	-1.048298	-1.166137	2.302647
C	-3.140024	-2.361356	2.179779
H	-3.560850	-3.367637	0.313664
C	-2.220154	-1.613784	2.912296
H	-0.351191	-0.537863	2.866161
H	-4.069665	-2.697199	2.647709
H	-2.421654	-1.355365	3.955371
C	-2.159040	-3.352265	-1.944478
H	-1.593176	-3.542966	-2.865362

H	-2.589166	-4.307836	-1.597834
H	-2.971090	-2.637228	-2.152167
Pd	0.320241	0.085859	0.166244
O	-1.303694	1.366874	0.402723
C	-2.186679	1.195675	-0.518283
O	-2.039874	0.480169	-1.507431
C	-3.526481	1.912482	-0.291460
C	-3.944721	2.590886	-1.591294
H	-4.941851	3.053257	-1.486251
H	-3.232004	3.387996	-1.869601
H	-3.974281	1.864099	-2.416992
C	-3.455786	2.930899	0.837027
H	-4.433211	3.430051	0.961503
H	-3.184560	2.452698	1.791098
H	-2.698810	3.706651	0.632681
C	-4.525144	0.810793	0.069309
H	-4.601535	0.072465	-0.745660
H	-4.204041	0.276977	0.982461
H	-5.527008	1.237016	0.254727

****

Structure: IM4aB

Charge = 0, Multiplicity = 1

Final SCF Energy = -1450.40083189 Hartree

Gibbs Free Energy = -1450.013616 Hartree

Thermal Correction = 0.387216 Hartree

Imaginary Frequencies = none

Cartesian Coordinates (Angstrom):

C	-2.995564	2.159249	-0.327110
O	-4.122096	1.930568	-0.712827
N	-2.361889	3.404144	-0.462492
C	-1.967537	1.263043	0.285048
C	-1.760963	-0.084791	0.240902
C	-2.648204	-1.207771	0.000512
C	-2.047019	-2.480707	0.031070
C	-4.043012	-1.101775	-0.134627
C	-2.836144	-3.628120	-0.024353
C	-4.817235	-2.254140	-0.205175
H	-4.499130	-0.107990	-0.173361
C	-4.220502	-3.513746	-0.137901
H	-2.359355	-4.613593	0.002752
H	-5.903256	-2.169341	-0.301621

H	-4.837808	-4.415548	-0.182634
C	-0.563347	-2.588398	0.045434
C	0.166109	-2.171854	-1.108754
C	0.107700	-3.396133	1.004329
C	1.489683	-2.610433	-1.309927
H	-0.367559	-1.671751	-1.924984
C	1.418004	-3.780276	0.804749
H	-0.446192	-3.725647	1.889158
C	2.106090	-3.401673	-0.362831
H	2.018952	-2.300057	-2.214681
H	1.922792	-4.394028	1.555586
H	3.137011	-3.734482	-0.514973
C	-1.088761	3.395356	0.098454
C	-0.855914	2.116901	0.635396
C	-0.129045	4.386479	0.206670
C	0.298278	1.835927	1.361880
C	1.077844	4.065587	0.858822
H	-0.291668	5.387878	-0.201975
C	1.298515	2.819223	1.433522
H	0.352877	0.938948	2.017973
H	1.851013	4.834906	0.940081
H	2.227671	2.613106	1.971963
C	-2.992014	4.546709	-1.059580
H	-3.994726	4.243208	-1.390700
H	-3.089698	5.376913	-0.338696
H	-2.421080	4.905851	-1.932630
Pd	0.192156	-0.432373	0.420202
O	2.304595	-0.523977	0.631961
C	2.766114	0.158559	-0.352693
O	2.044792	0.661409	-1.219601
C	4.290415	0.299357	-0.450998
C	4.733648	-0.562423	-1.634268
H	5.824935	-0.485445	-1.782440
H	4.493841	-1.626868	-1.460997
H	4.231661	-0.240994	-2.561222
C	4.987949	-0.166924	0.818458
H	6.081569	-0.052898	0.716846
H	4.662447	0.418376	1.694446
H	4.766134	-1.224493	1.032817
C	4.623218	1.761154	-0.735597
H	4.101563	2.109064	-1.639991
H	4.313436	2.411521	0.100830

H	5.710441	1.889251	-0.878247

****

Structure: IM4bB
Charge = 0, Multiplicity = 1
Final SCF Energy = -1450.41484067 Hartree
Gibbs Free Energy = -1450.027282 Hartree
Thermal Correction = 0.387559 Hartree
Imaginary Frequencies = none
Cartesian Coordinates (Angstrom):

C	2.285090	-2.085925	-0.680012
O	3.315231	-1.735321	-1.210761
N	2.003356	-3.391872	-0.299763
C	1.072830	-1.272589	-0.295493
C	0.983723	0.055676	-0.522241
C	2.013907	0.926352	-1.088281
C	2.592086	1.884009	-0.224216
C	2.503651	0.797631	-2.395102
C	3.702905	2.618301	-0.640457
C	3.576315	1.571663	-2.815012
H	2.050109	0.059324	-3.061555
C	4.188659	2.467389	-1.936007
H	4.159565	3.337975	0.046955
H	3.957633	1.461357	-3.833823
H	5.042511	3.063660	-2.269583
C	1.959938	2.052400	1.096794
C	0.622364	2.521202	1.141832
C	2.582715	1.670773	2.285246
C	-0.084928	2.537190	2.360118
C	1.870773	1.693488	3.483448
H	3.612716	1.302167	2.255493
C	0.534374	2.099476	3.523919
H	-1.116126	2.904146	2.372213
H	2.359893	1.359806	4.403424
H	-0.014593	2.086401	4.468792
C	0.760674	-3.480863	0.317739
C	0.150841	-2.209542	0.355197
C	0.157831	-4.608909	0.856113
C	-1.083558	-2.076304	0.982554
C	-1.092370	-4.453461	1.462410
H	0.647888	-5.585654	0.811748
C	-1.703301	-3.202797	1.530666

H	-1.569275	-1.097701	1.051622
H	-1.590656	-5.325837	1.895327
H	-2.676109	-3.097425	2.019166
C	2.909677	-4.485705	-0.491901
H	3.807401	-4.094577	-0.990297
H	3.203147	-4.938948	0.470984
H	2.461911	-5.271950	-1.123786
Pd	-0.623639	1.044687	-0.005826
O	-2.749000	1.721109	0.139630
C	-3.050930	0.786540	-0.648706
O	-2.138169	0.040979	-1.128735
C	-4.497719	0.462673	-0.975808
C	-4.629810	0.098554	-2.451385
H	-5.673482	-0.178098	-2.678323
H	-4.356111	0.947449	-3.099925
H	-3.978971	-0.749634	-2.711600
C	-5.399673	1.638759	-0.629515
H	-6.450439	1.383490	-0.848622
H	-5.320451	1.905705	0.435276
H	-5.135081	2.533585	-1.216210
C	-4.855401	-0.750096	-0.109440
H	-4.207198	-1.609646	-0.347082
H	-4.742435	-0.515631	0.963504
H	-5.904101	-1.045514	-0.284039
H	0.247691	3.103613	0.280853

****

Structure: $TS_{4\text{-}5}B$

Charge = 0, Multiplicity = 1

Final SCF Energy = -1450.38150167 Hartree

Gibbs Free Energy = -1449.997952 Hartree

Thermal Correction = 0.383549 Hartree

Imaginary Frequencies = -1537.85 cm^-1

Cartesian Coordinates (Angstrom):

C	3.683655	0.348990	-0.261455
O	4.064011	1.482646	-0.445688
N	4.524626	-0.730582	0.006651
C	2.286454	-0.207841	-0.243222
C	1.149601	0.511911	-0.401488
C	1.009681	1.904308	-0.797110
C	0.188725	2.773475	-0.039733
C	1.670777	2.406295	-1.932500

C	0.107540	4.126004	-0.393497
C	1.547257	3.738764	-2.289937
H	2.298563	1.731992	-2.520155
C	0.771091	4.604899	-1.514430
H	-0.525370	4.794909	0.198099
H	2.069426	4.114077	-3.174217
H	0.672745	5.656371	-1.798167
C	-0.535222	2.242791	1.119606
C	-1.227915	1.004301	1.041751
C	-0.531458	2.943662	2.333458
C	-1.796432	0.465719	2.219640
C	-1.126799	2.405141	3.467748
H	0.001830	3.897139	2.398892
C	-1.752040	1.155417	3.420196
H	-2.330755	-0.489125	2.153987
H	-1.087091	2.958496	4.410693
H	-2.215858	0.735988	4.317075
C	3.801145	-1.905959	0.170907
C	2.424874	-1.635024	0.034174
C	4.274206	-3.185887	0.424341
C	1.512562	-2.678025	0.147552
C	3.337390	-4.218781	0.538687
H	5.344882	-3.382212	0.530208
C	1.971728	-3.973643	0.400604
H	0.425168	-2.505876	0.053403
H	3.685270	-5.236465	0.738034
H	1.256692	-4.795248	0.490995
C	5.951676	-0.618941	0.091200
H	6.216285	0.433031	-0.084253
H	6.320429	-0.919171	1.087145
H	6.450395	-1.245320	-0.668576
Pd	-0.538253	-0.483511	-0.302359
O	-2.424305	-1.515043	-0.423125
C	-3.418216	-0.748322	-0.565430
O	-3.358113	0.505634	-0.484094
C	-4.794725	-1.387921	-0.750052
C	-5.345009	-1.562966	0.669433
H	-6.354640	-2.006858	0.634350
H	-4.698885	-2.231020	1.264139
H	-5.414470	-0.591216	1.186811
C	-4.678480	-2.744115	-1.432770
H	-5.677943	-3.199841	-1.536446

H	-4.243780	-2.648947	-2.441681
H	-4.040003	-3.430716	-0.857309
C	-5.709325	-0.466886	-1.547939
H	-5.812047	0.514174	-1.061257
H	-5.317611	-0.296098	-2.564457
H	-6.710633	-0.920494	-1.642078
H	-2.114556	0.847900	0.111574

****

Structure: IM5B
Charge = 0, Multiplicity = 1
Final SCF Energy = -1450.41373775 Hartree
Gibbs Free Energy = -1450.025415 Hartree
Thermal Correction = 0.388323 Hartree
Imaginary Frequencies = none
Cartesian Coordinates (Angstrom):

C	3.751008	0.287864	-0.194177
O	4.159681	1.418271	-0.338430
N	4.570281	-0.802760	0.097121
C	2.348032	-0.253434	-0.257546
C	1.232101	0.497119	-0.448401
C	1.175897	1.932306	-0.726281
C	0.324837	2.757192	0.047157
C	1.901308	2.499374	-1.783716
C	0.252257	4.122936	-0.246570
C	1.799059	3.853117	-2.074193
H	2.557981	1.858749	-2.377317
C	0.976583	4.670234	-1.299723
H	-0.413635	4.764103	0.339180
H	2.369097	4.274982	-2.906668
H	0.888082	5.736765	-1.525518
C	-0.457732	2.156702	1.144200
C	-0.858966	0.807919	1.095617
C	-0.812069	2.906837	2.278462
C	-1.550727	0.232564	2.165983
C	-1.528195	2.342469	3.327769
H	-0.482757	3.948096	2.352932
C	-1.899352	1.000548	3.276649
H	-1.840186	-0.824571	2.127136
H	-1.782373	2.951012	4.200318
H	-2.456836	0.546935	4.101750
C	3.831027	-1.971582	0.212952

C	2.463686	-1.690056	0.008926
C	4.290047	-3.252855	0.485645
C	1.547166	-2.732662	0.087389
C	3.348034	-4.284032	0.557762
H	5.354906	-3.448582	0.640892
C	1.991759	-4.029532	0.362000
H	0.473474	-2.542815	-0.052430
H	3.683627	-5.303123	0.771337
H	1.269254	-4.847906	0.423711
C	5.990345	-0.702705	0.259602
H	6.269353	0.350599	0.116657
H	6.303816	-1.022432	1.268616
H	6.526558	-1.319652	-0.482509
Pd	-0.540056	-0.370303	-0.500491
O	-2.540860	-1.328779	-0.705367
C	-3.589960	-0.702267	-0.611903
O	-3.629936	0.592337	-0.396428
C	-4.948747	-1.366643	-0.653662
C	-5.359437	-1.535709	0.816119
H	-6.343196	-2.030569	0.868222
H	-4.633588	-2.162105	1.361450
H	-5.433881	-0.562645	1.327842
C	-4.839040	-2.728704	-1.324735
H	-5.824496	-3.221811	-1.321921
H	-4.506518	-2.636437	-2.371336
H	-4.123644	-3.380650	-0.801605
C	-5.963402	-0.488627	-1.381320
H	-6.085900	0.487949	-0.890226
H	-5.666458	-0.310597	-2.427940
H	-6.942676	-0.994307	-1.390967
H	-2.713576	0.917384	-0.231103

****

Structure: IM6B

Charge = 0, Multiplicity = 1

Final SCF Energy = -1600.22594727 Hartree

Gibbs Free Energy = -1599.784288 Hartree

Thermal Correction = 0.441659 Hartree

Imaginary Frequencies = none

Cartesian Coordinates (Angstrom):

C	-2.979104	1.506392	-0.275870
O	-3.105242	2.629315	0.163272

N	-4.047817	0.765180	-0.795651
C	-1.776586	0.615443	-0.365147
C	-0.528152	0.898245	0.103769
C	-0.094041	2.141695	0.751024
C	1.089734	2.773999	0.294186
C	-0.769644	2.677921	1.855909
C	1.527159	3.933762	0.941517
C	-0.300217	3.813638	2.505076
H	-1.690559	2.198899	2.199472
C	0.848270	4.450575	2.040618
H	2.445916	4.420331	0.599283
H	-0.840477	4.208372	3.370372
H	1.225297	5.346064	2.543376
C	1.833484	2.198864	-0.846397
C	1.808873	0.808791	-1.086677
C	2.564874	3.024001	-1.715003
C	2.478710	0.295964	-2.203366
C	3.254567	2.498568	-2.803258
H	2.563355	4.107513	-1.555306
C	3.209609	1.128916	-3.052140
H	2.438520	-0.780078	-2.413421
H	3.809569	3.164747	-3.470562
H	3.735741	0.706524	-3.914607
C	-3.625035	-0.473184	-1.254549
C	-2.237194	-0.608396	-1.031385
C	-4.381648	-1.469004	-1.858657
C	-1.598280	-1.761153	-1.475426
C	-3.718737	-2.628453	-2.279498
H	-5.456482	-1.341850	-2.019061
C	-2.343799	-2.768613	-2.098336
H	-0.515562	-1.872636	-1.337353
H	-4.287751	-3.424458	-2.769413
H	-1.839140	-3.673103	-2.450315
C	-5.387524	1.262820	-0.877141
H	-5.391671	2.278924	-0.457722
H	-5.742354	1.305239	-1.921893
H	-6.089479	0.632774	-0.300723
Pd	0.907937	-0.454751	0.168223
C	3.763449	-1.499475	0.347758
O	2.601163	-1.890344	0.331777
N	4.824148	-2.283515	0.608436
H	4.028993	-0.439847	0.134693

C	6.168360	-1.763063	0.598777
H	6.648840	-1.892589	1.584058
H	6.787234	-2.282681	-0.153160
H	6.156692	-0.691016	0.355918
C	4.657531	-3.687300	0.906910
H	3.588118	-3.932292	0.893879
H	5.181811	-4.307764	0.160061
H	5.071321	-3.921786	1.902415
C	-1.264557	-1.365084	2.007949
O	-0.234184	-1.852652	1.549638
N	-2.463774	-1.966355	2.023585
H	-1.284866	-0.346206	2.457308
C	-2.649708	-3.283554	1.456050
H	-1.672310	-3.689549	1.166246
H	-3.122687	-3.951776	2.195546
H	-3.291181	-3.231507	0.558599
C	-3.650212	-1.253906	2.429134
H	-4.325199	-1.111414	1.564144
H	-4.198064	-1.806858	3.210977
H	-3.381641	-0.261648	2.821879

****

Structure: $TS_{6\text{-}7}B$

Charge = 0, Multiplicity = 1

Final SCF Energy = -1600.21136705 Hartree

Gibbs Free Energy = -1599.771129 Hartree

Thermal Correction = 0.440238 Hartree

Imaginary Frequencies = -258.95 cm^-1

Cartesian Coordinates (Angstrom):

C	2.921334	1.406702	0.509513
O	3.089322	2.554563	0.153305
N	3.930920	0.627915	1.092604
C	1.716459	0.530253	0.439671
C	0.478446	0.899126	-0.027189
C	0.141423	2.194787	-0.649827
C	-1.048160	2.785989	-0.172999
C	0.833486	2.794305	-1.702002
C	-1.517946	3.968893	-0.735657
C	0.344087	3.966477	-2.276629
H	1.769564	2.355970	-2.058847
C	-0.824297	4.554781	-1.795214
H	-2.438378	4.427912	-0.359736

H	0.890111	4.433308	-3.101469
H	-1.199036	5.477338	-2.248065
C	-1.625301	2.043525	0.955306
C	-1.154950	0.714577	1.085148
C	-2.451408	2.591467	1.937383
C	-1.428520	0.012645	2.267991
C	-2.785776	1.848852	3.070477
H	-2.799258	3.625514	1.838796
C	-2.253704	0.571612	3.246500
H	-1.025774	-0.995585	2.414694
H	-3.431321	2.285912	3.838169
H	-2.484702	0.001144	4.151759
C	3.468890	-0.643137	1.395309
C	2.111346	-0.751004	1.014214
C	4.164837	-1.702279	1.962326
C	1.452034	-1.960977	1.216841
C	3.475059	-2.902819	2.171781
H	5.216560	-1.598424	2.245447
C	2.135160	-3.030156	1.807100
H	0.410854	-2.075492	0.884884
H	3.998474	-3.749792	2.625498
H	1.614939	-3.978248	1.972089
C	5.256261	1.110099	1.339407
H	5.299013	2.154221	0.998199
H	5.506981	1.074555	2.414034
H	6.012726	0.521844	0.789239
Pd	-0.872219	-0.463965	-0.577235
C	-3.760133	-1.256910	-0.124939
O	-2.799506	-1.687613	-0.749037
N	-4.995012	-1.794915	-0.145800
H	-3.681992	-0.359783	0.532879
C	-6.070713	-1.235832	0.632806
H	-6.900374	-0.903428	-0.015483
H	-6.471702	-1.979380	1.343703
H	-5.708574	-0.369907	1.206555
C	-5.285522	-2.958933	-0.949309
H	-4.376369	-3.256982	-1.487009
H	-5.622863	-3.796549	-0.314683
H	-6.082467	-2.737209	-1.679703
C	1.078284	-1.161936	-2.484171
O	-0.022033	-1.688572	-2.336282
N	2.263830	-1.772622	-2.312490

H	1.185614	-0.093104	-2.787314
C	2.339202	-3.151462	-1.886565
H	1.320826	-3.544126	-1.768165
H	2.880574	-3.757336	-2.633561
H	2.866925	-3.223602	-0.919205
C	3.507841	-1.051393	-2.414115
H	4.022479	-1.029472	-1.435098
H	4.181176	-1.526247	-3.148446
H	3.321331	-0.013830	-2.729377

****

Structure: IM7B

Charge = 0, Multiplicity = 1

Final SCF Energy = -1600.24140913 Hartree

Gibbs Free Energy = -1599.800597 Hartree

Thermal Correction = 0.440812 Hartree

Imaginary Frequencies = none

Cartesian Coordinates (Angstrom):

C	3.270801	1.319525	0.023490
O	3.475576	2.398030	-0.508212
N	4.309629	0.418007	0.282557
C	2.035440	0.696239	0.556346
C	0.780716	1.295832	0.630089
C	0.225317	2.421262	-0.173744
C	-1.158197	2.553331	0.125660
C	0.782439	3.229165	-1.164809
C	-1.953488	3.482694	-0.529143
C	-0.024985	4.164901	-1.820607
H	1.844606	3.150005	-1.396117
C	-1.374837	4.296248	-1.506995
H	-3.017327	3.571338	-0.286725
H	0.419650	4.808594	-2.585158
H	-1.986699	5.039393	-2.027265
C	-1.513732	1.577129	1.167097
C	-0.304659	0.882739	1.533368
C	-2.651371	1.539367	2.014119
C	-0.260359	0.225714	2.794110
C	-2.585322	0.837665	3.200157
H	-3.551874	2.101252	1.741626
C	-1.383338	0.203438	3.595077
H	0.672039	-0.218089	3.151101
H	-3.454769	0.803496	3.863992

H	-1.336951	-0.295808	4.567456
C	3.824708	-0.755281	0.828354
C	2.426732	-0.636282	1.018794
C	4.527702	-1.921086	1.103355
C	1.727571	-1.774624	1.429215
C	3.806684	-3.026603	1.566037
H	5.611150	-1.970249	0.957754
C	2.421179	-2.955928	1.707674
H	0.637493	-1.757513	1.512148
H	4.334396	-3.955327	1.802893
H	1.863698	-3.836466	2.039870
C	5.678194	0.680424	-0.044443
H	5.731200	1.692928	-0.468763
H	6.323450	0.630474	0.849554
H	6.067737	-0.039779	-0.788113
Pd	-1.303363	-0.156549	-0.121364
C	-4.217121	-1.089861	-0.370095
O	-3.236039	-0.983463	-1.089126
N	-5.327884	-1.790335	-0.681988
H	-4.272643	-0.599399	0.631695
C	-6.450831	-1.869902	0.215739
H	-7.364007	-1.461140	-0.251673
H	-6.656179	-2.916152	0.502939
H	-6.243809	-1.295872	1.130596
C	-5.427110	-2.497906	-1.936829
H	-4.498252	-2.350879	-2.502561
H	-5.578944	-3.577195	-1.763520
H	-6.277508	-2.120829	-2.530776
C	0.970797	-1.047548	-1.894085
O	-0.017744	-1.600535	-1.428254
N	2.123412	-1.662681	-2.217562
H	1.002014	0.050630	-2.090153
C	2.303831	-3.076464	-1.982483
H	1.361988	-3.497667	-1.608209
H	2.590623	-3.590095	-2.916288
H	3.093345	-3.241810	-1.227409
C	3.260473	-0.923977	-2.704601
H	4.118787	-1.048279	-2.019331
H	3.565281	-1.272441	-3.707067
H	3.022396	0.149655	-2.761125

9.3. Path C: Amide-directed *ortho*-C-H activation

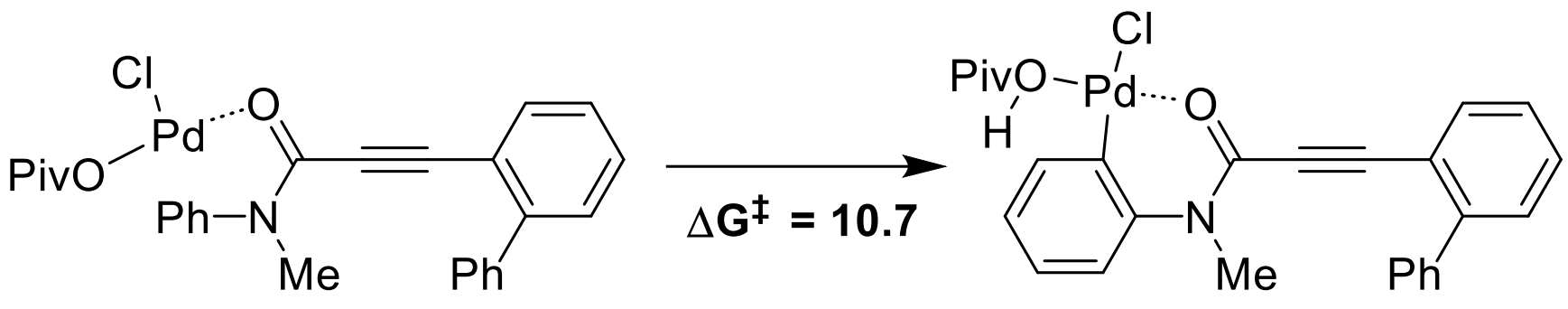

IM1C (5.3) **IM2C** (10.7)

Cartesian coordinates

****

Structure: IM1C

Charge = 0, Multiplicity = 1

Final SCF Energy = -1911.01774604 Hartree

Gibbs Free Energy = -1910.620995 Hartree

Thermal Correction = 0.396751 Hartree

Imaginary Frequencies = none

Cartesian Coordinates (Angstrom):

C	-0.900362	-0.446067	0.130339
O	-0.028339	-1.323190	0.006240
N	-0.588452	0.867079	0.240490
C	-2.282453	-0.825972	0.155149
C	-3.446066	-1.179366	0.167517
C	-4.809168	-1.586314	0.228994
C	-5.854512	-0.643726	0.082030
C	-5.106810	-2.931045	0.508059
C	-7.170001	-1.081243	0.255881
C	-6.423067	-3.343385	0.660317
H	-4.281433	-3.639876	0.616954
C	-7.455202	-2.413749	0.541155
H	-7.983595	-0.358068	0.140355
H	-6.645343	-4.391213	0.877618
H	-8.494661	-2.731084	0.662303
C	-5.578752	0.771790	-0.251446
C	-4.847714	1.108853	-1.399045
C	-6.067323	1.800607	0.563620
C	-4.612516	2.441956	-1.721855
H	-4.478771	0.312712	-2.054051
C	-5.818981	3.134215	0.247602
H	-6.635106	1.545113	1.464527
C	-5.092229	3.458255	-0.896326
H	-4.055655	2.689582	-2.630452
H	-6.198662	3.926395	0.899176
H	-4.906341	4.505895	-1.149844

C	0.793240	1.268257	0.351365
C	1.526323	0.944332	1.519175
C	1.286542	2.254324	-0.533541
C	2.722137	1.629704	1.799616
C	2.477239	2.893030	-0.249840
H	0.714300	2.494619	-1.434124
C	3.187824	2.592445	0.924117
H	3.281474	1.373725	2.701820
H	2.868244	3.639887	-0.945746
H	4.118389	3.123468	1.144102
C	-1.566867	1.925934	0.433981
H	-2.582758	1.508606	0.466794
H	-1.364273	2.453073	1.380724
H	-1.522282	2.657537	-0.388046
Pd	1.946849	-0.674579	-0.192336
O	3.788801	-0.036618	-0.532113
C	4.664235	-0.350352	0.377987
O	4.404008	-0.841364	1.462309
Cl	2.535695	-2.598243	-1.277383
C	6.111402	-0.079149	-0.059511
C	6.935084	0.286904	1.168021
H	6.864150	-0.496918	1.936519
H	7.995217	0.421050	0.891757
H	6.581440	1.230402	1.620779
C	6.204595	1.021620	-1.108646
H	7.260601	1.190682	-1.383068
H	5.646174	0.757679	-2.019072
H	5.797576	1.976272	-0.732085
C	6.603880	-1.402233	-0.651141
H	6.555140	-2.207045	0.100233
H	5.982065	-1.702756	-1.510702
H	7.649454	-1.303421	-0.992019
H	1.093316	0.266517	2.262172

****

Structure: $TS_{1-2}C$

Charge = 0, Multiplicity = 1

Final SCF Energy = -1910.99657834 Hartree

Gibbs Free Energy = -1910.603965 Hartree

Thermal Correction = 0.392613 Hartree

Imaginary Frequencies = -1634.22 cm^-1

Cartesian Coordinates (Angstrom):

C	-0.694867	-0.150644	-0.527783
O	0.123354	-0.737741	-1.254704
N	-0.470483	1.051636	0.068616
C	-1.990069	-0.733125	-0.322556
C	-3.099223	-1.208387	-0.170334
C	-4.394504	-1.751497	0.062580
C	-5.483718	-0.903213	0.375614
C	-4.568193	-3.145630	0.050408
C	-6.708548	-1.490456	0.702325
C	-5.799604	-3.703725	0.364330
H	-3.711262	-3.779116	-0.194381
C	-6.868678	-2.873525	0.698181
H	-7.555784	-0.840123	0.942416
H	-5.925351	-4.789417	0.354208
H	-7.840870	-3.306857	0.949166
C	-5.345722	0.570813	0.356415
C	-4.904861	1.234348	-0.796803
C	-5.676469	1.329728	1.485844
C	-4.797116	2.621733	-0.818099
H	-4.660747	0.651809	-1.691157
C	-5.555734	2.717422	1.468722
H	-6.016971	0.818280	2.392421
C	-5.117480	3.367105	0.316565
H	-4.468421	3.126016	-1.731719
H	-5.809065	3.295617	2.361922
H	-5.033016	4.457522	0.299754
C	0.633712	1.858216	-0.341407
C	1.948132	1.356827	-0.363712
C	0.362598	3.169655	-0.743880
C	2.961994	2.206889	-0.843004
C	1.391344	3.989107	-1.194987
H	-0.666153	3.541372	-0.733226
C	2.697975	3.506376	-1.254495
H	3.989331	1.826110	-0.870514
H	1.165166	5.009538	-1.516933
H	3.506799	4.145824	-1.617988
C	-1.473747	1.643173	0.949048
H	-1.869012	0.880114	1.634052
H	-0.991619	2.426306	1.548254
H	-2.324008	2.078102	0.394963
Pd	2.150780	-0.728788	-0.828016
O	4.080319	-0.799558	-0.182089

C	4.249580	-0.319298	0.977498
O	3.368561	0.318581	1.605385
Cl	2.321344	-2.885621	-1.616775
C	5.641945	-0.447985	1.588584
C	5.532027	-0.568908	3.104392
H	4.982857	-1.479871	3.394026
H	6.540837	-0.626526	3.546524
H	5.006881	0.294462	3.539241
C	6.364958	0.851753	1.220457
H	7.379322	0.853343	1.654114
H	6.463959	0.952258	0.126087
H	5.822978	1.730825	1.607702
C	6.385967	-1.643410	1.009387
H	5.850961	-2.585079	1.211832
H	6.500079	-1.558349	-0.081201
H	7.388727	-1.712466	1.463828
H	2.440973	0.658348	0.641747

****

Structure: IM2C
Charge = 0, Multiplicity = 1
Final SCF Energy = -1911.04163340 Hartree
Gibbs Free Energy = -1910.646556 Hartree
Thermal Correction = 0.395077 Hartree
Imaginary Frequencies = none
Cartesian Coordinates (Angstrom):

C	-0.848249	-0.080987	-0.313409
O	0.049088	-0.722650	-0.888989
N	-0.701928	1.162634	0.203596
C	-2.144997	-0.689589	-0.216627
C	-3.241603	-1.213181	-0.168879
C	-4.527787	-1.813757	-0.056628
C	-5.676757	-1.019215	0.171655
C	-4.638705	-3.213578	-0.104528
C	-6.898958	-1.664435	0.377373
C	-5.867449	-3.829831	0.087518
H	-3.735922	-3.805010	-0.280086
C	-6.997825	-3.052566	0.335942
H	-7.791758	-1.055301	0.551515
H	-5.943633	-4.919555	0.049304
H	-7.968780	-3.531397	0.490742
C	-5.602563	0.459424	0.194002

C	-5.090856	1.169178	-0.900884
C	-6.066187	1.175966	1.304278
C	-5.045352	2.559888	-0.884755
H	-4.741404	0.619651	-1.780970
C	-6.006778	2.567525	1.326338
H	-6.462484	0.628598	2.166133
C	-5.498165	3.263073	0.231374
H	-4.658722	3.099796	-1.754183
H	-6.363781	3.112430	2.204984
H	-5.461254	4.356212	0.244285
C	0.430038	1.979754	-0.117729
C	1.692287	1.448712	-0.434532
C	0.239199	3.371944	-0.122269
C	2.722252	2.334579	-0.766230
C	1.280993	4.232571	-0.441759
H	-0.742636	3.795464	0.101221
C	2.530211	3.713257	-0.767581
H	3.706497	1.928095	-1.014419
H	1.106752	5.312140	-0.443961
H	3.358275	4.380779	-1.024322
C	-1.775105	1.747750	0.997616
H	-2.358562	0.954769	1.481521
H	-1.334836	2.379341	1.781136
H	-2.467997	2.354905	0.390039
Pd	2.033885	-0.502446	-0.502616
O	4.051601	-0.147632	-0.039855
C	4.953419	-0.872115	0.395617
O	4.864477	-2.148976	0.557306
Cl	2.329981	-2.921594	-0.616837
C	6.288446	-0.274436	0.808888
C	7.405703	-0.992987	0.050522
H	7.298194	-0.865821	-1.039536
H	8.380793	-0.570405	0.344521
H	7.414227	-2.070889	0.271256
C	6.450540	-0.508730	2.313980
H	7.411285	-0.085082	2.650661
H	5.645615	-0.016696	2.885087
H	6.441101	-1.582275	2.556667
C	6.319842	1.216207	0.506266
H	6.206879	1.410245	-0.572804
H	5.512938	1.753818	1.028992
H	7.285271	1.638674	0.829804

H	3.974182	-2.513743	0.202749

9.4 Path D: sequence CMD

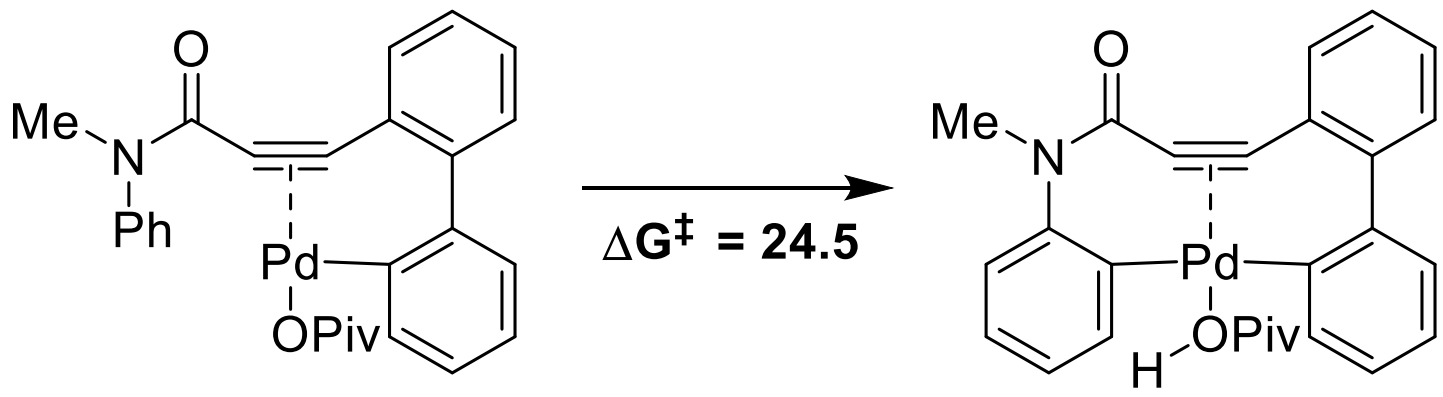

IM3A (0.9) **IM4A'** (18.3)

Cartesian coordinates

****

Structure: $TS_{3\text{-}4}D$

Charge = 0, Multiplicity = 1

Final SCF Energy = -1450.32231602 Hartree

Gibbs Free Energy = -1449.937149 Hartree

Thermal Correction = 0.385167 Hartree

Imaginary Frequencies = -1550.30 cm^-1

Cartesian Coordinates (Angstrom):

C	-0.208096	3.130277	0.537728
O	0.407079	3.974672	1.162721
N	-1.557482	3.210555	0.301241
C	0.516526	1.965515	0.037791
C	1.586045	1.296037	0.057787
C	2.957485	1.088475	0.373941
C	3.615732	-0.126575	0.099999
C	3.604014	2.136283	1.054622
C	4.926798	-0.263998	0.578154
C	4.907165	1.976751	1.496217
H	3.042216	3.057755	1.240323
C	5.561021	0.764717	1.263753
H	5.462461	-1.204435	0.428515
H	5.409885	2.785728	2.032444
H	6.582177	0.616767	1.626815
C	2.943216	-1.165041	-0.699993
C	1.552317	-1.204241	-0.909099
C	3.732689	-2.135964	-1.349430
C	1.004567	-2.169556	-1.760183
C	3.183315	-3.099192	-2.179935
H	4.819024	-2.107064	-1.229005
C	1.808283	-3.112812	-2.394288
H	-0.075155	-2.207032	-1.932055

H	3.831719	-3.827924	-2.674214
H	1.353012	-3.855899	-3.055754
C	-2.244664	2.327855	-0.561336
C	-2.043174	0.931871	-0.502538
C	-3.152834	2.881380	-1.475161
C	-2.762471	0.143646	-1.428421
C	-3.851406	2.068837	-2.357294
H	-3.292273	3.964964	-1.513026
C	-3.653744	0.688905	-2.342074
H	-2.633198	-0.944548	-1.401148
H	-4.548623	2.520104	-3.069222
H	-4.202024	0.041986	-3.032284
C	-2.225979	4.396330	0.803912
H	-1.750073	4.694513	1.747159
H	-2.145008	5.250666	0.109114
H	-3.286926	4.171452	0.978901
Pd	-0.019615	-0.028526	-0.278008
O	-1.061370	-1.850985	0.180257
C	-1.888471	-1.734683	1.126414
O	-2.273917	-0.624927	1.585912
C	-2.533783	-3.006001	1.669132
C	-3.763148	-3.236603	0.783088
H	-4.319905	-4.122229	1.133362
H	-4.442960	-2.368344	0.815334
H	-3.468875	-3.411083	-0.265627
C	-2.969617	-2.805611	3.115477
H	-3.460811	-3.720571	3.487124
H	-2.107138	-2.593781	3.768598
H	-3.674643	-1.966520	3.209711
C	-1.580344	-4.188445	1.556969
H	-1.263283	-4.354332	0.516636
H	-0.671957	-4.029486	2.161020
H	-2.075582	-5.103671	1.922665
H	-1.980392	0.272280	0.671760

****

Structure: IM4A'

Charge = 0, Multiplicity = 1

Final SCF Energy = -1450.33793191 Hartree

Gibbs Free Energy = -1449.948614 Hartree

Thermal Correction = 0.389318 Hartree

Imaginary Frequencies = none

Cartesian Coordinates (Angstrom):

C	-0.126802	3.209284	-0.317341
O	-0.880986	4.044708	-0.790695
N	1.234858	3.383115	-0.243087
C	-0.675605	1.943217	0.135349
C	-1.699421	1.187549	0.091245
C	-3.059512	0.959012	-0.257740
C	-3.659519	-0.313807	-0.161169
C	-3.754139	2.060891	-0.793614
C	-4.963773	-0.438906	-0.665088
C	-5.047750	1.906479	-1.262034
H	-3.232835	3.022864	-0.847025
C	-5.644739	0.644823	-1.204059
H	-5.459203	-1.412387	-0.653248
H	-5.587132	2.758705	-1.683542
H	-6.658447	0.501854	-1.589826
C	-2.940237	-1.429688	0.482372
C	-1.553418	-1.398047	0.738818
C	-3.682669	-2.546444	0.917225
C	-0.982975	-2.461741	1.453663
C	-3.096162	-3.600144	1.599316
H	-4.762747	-2.574463	0.750919
C	-1.733928	-3.553597	1.880160
H	0.085401	-2.457664	1.690635
H	-3.706725	-4.445138	1.930183
H	-1.251097	-4.363036	2.436613
C	2.089250	2.504253	0.463458
C	1.732695	1.166017	0.727267
C	3.322468	3.008259	0.915207
C	2.618553	0.413946	1.518820
C	4.190542	2.219383	1.657178
H	3.601516	4.043256	0.708606
C	3.834952	0.913958	1.977171
H	2.361207	-0.619155	1.781988
H	5.141093	2.638893	1.999000
H	4.497010	0.284001	2.578531
C	1.754469	4.622058	-0.783936
H	0.995359	5.049918	-1.449046
H	1.969075	5.362813	0.005775
H	2.679095	4.430788	-1.349219
Pd	-0.032928	0.015871	0.418894
O	1.299648	-1.715260	-0.084980

C	2.102377	-1.678841	-1.010356
O	2.393502	-0.576619	-1.657831
C	2.890492	-2.896058	-1.441222
C	4.264759	-2.748920	-0.774191
H	4.892048	-3.619199	-1.028433
H	4.782184	-1.837335	-1.113672
H	4.170745	-2.705974	0.324169
C	3.046111	-2.928355	-2.959473
H	3.631472	-3.816762	-3.247483
H	2.068047	-2.991446	-3.463986
H	3.568436	-2.036166	-3.335477
C	2.198661	-4.158683	-0.944800
H	2.102948	-4.163017	0.151705
H	1.184837	-4.255560	-1.365979
H	2.783234	-5.043011	-1.245915
H	1.969782	0.177358	-1.178085

9.5. Cartesian coordinates and energies of the DFT-optimized products

Density functional theory (DFT) calculations were performed using the Gaussian16 program package. The ground-state geometry of products was optimized at the B3LYP/6-311G(d,p) level, and the time-dependent DFT (TD-DFT) calculations were conducted at the CAM-B3LYP/6-311+G(2d,p) level for the excited state calculation using the ground-state geometry. Geometry of compound **6a** was optimized at M06-2X/6-31G(d,p) to capture the dispersion-driven folded conformation. For consistent orbital energies and isosurface plots across the series, single-point calculations were performed at B3LYP/6-311G(d,p) on the optimized geometries.

****

Structure: **2a**

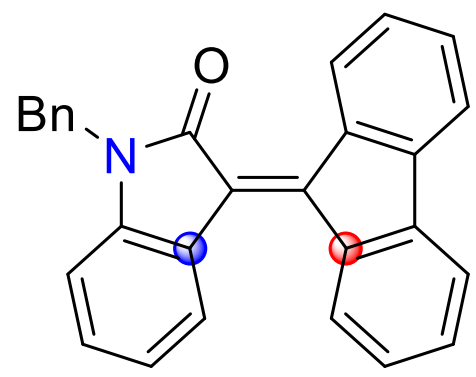

2a

Charge = 0, Multiplicity = 1

Final SCF Energy = -1208.70858398 Hartree

Gibbs Free Energy = -1208.374529 Hartree

Thermal Correction = 0.334055 Hartree

Imaginary Frequencies = none

Cartesian Coordinates (Angstrom):

C	5.391826	-2.109522	0.110009
C	4.230244	-2.819203	0.417060
C	2.987683	-2.184385	0.427566
C	2.905378	-0.828204	0.107621
C	4.096270	-0.105154	-0.129497
C	5.332512	-0.739546	-0.147491
H	6.348487	-2.619606	0.100105

H	4.291643	-3.873870	0.659620
H	2.107267	-2.747383	0.702608
H	6.241573	-0.178083	-0.331739
C	1.761654	0.119934	0.153579
C	2.368959	1.470137	0.016270
C	1.814382	2.752056	-0.030941
C	2.639556	3.844832	-0.302268
C	4.003680	3.679492	-0.537046
C	4.571060	2.405003	-0.504900
C	3.757406	1.314885	-0.228054
H	0.767007	2.902120	0.175232
H	2.207491	4.838854	-0.324522
H	4.625811	4.542578	-0.746242
H	5.629457	2.270925	-0.698935
C	0.432708	-0.223558	0.233203
C	-0.647444	0.647342	0.827960
C	-0.237185	-1.481264	-0.113640
C	0.136847	-2.589111	-0.878742
C	-1.587129	-1.379106	0.296191
C	-0.787628	-3.601543	-1.133439
H	1.135001	-2.665193	-1.286478
C	-2.516709	-2.381137	0.054401
C	-2.094044	-3.508082	-0.653132
H	-0.488008	-4.463220	-1.718078
H	-3.546014	-2.278786	0.372222
H	-2.800951	-4.304985	-0.853891
O	-0.594253	1.780885	1.272088
C	-3.024898	0.321669	1.521350
H	-3.344830	-0.426079	2.253615
H	-2.747373	1.226484	2.067778
C	-4.162543	0.622583	0.560618
C	-3.950720	1.401851	-0.580685
C	-5.452019	0.160299	0.830978
C	-5.007029	1.707341	-1.433375
H	-2.955644	1.773284	-0.798382
C	-6.512951	0.469005	-0.019263
H	-5.630090	-0.445236	1.714579
C	-6.291989	1.242185	-1.155389
H	-4.828192	2.312215	-2.315337
H	-7.508164	0.101223	0.204452
H	-7.113753	1.480843	-1.820988
N	-1.797710	-0.135137	0.887716

****

Structure: **4b**

Charge = 0, Multiplicity = 1

Final SCF Energy = -2338.76981823 Hartree

Gibbs Free Energy = -2338.129325 Hartree

Thermal Correction = 0.640493 Hartree

Imaginary Frequencies = none

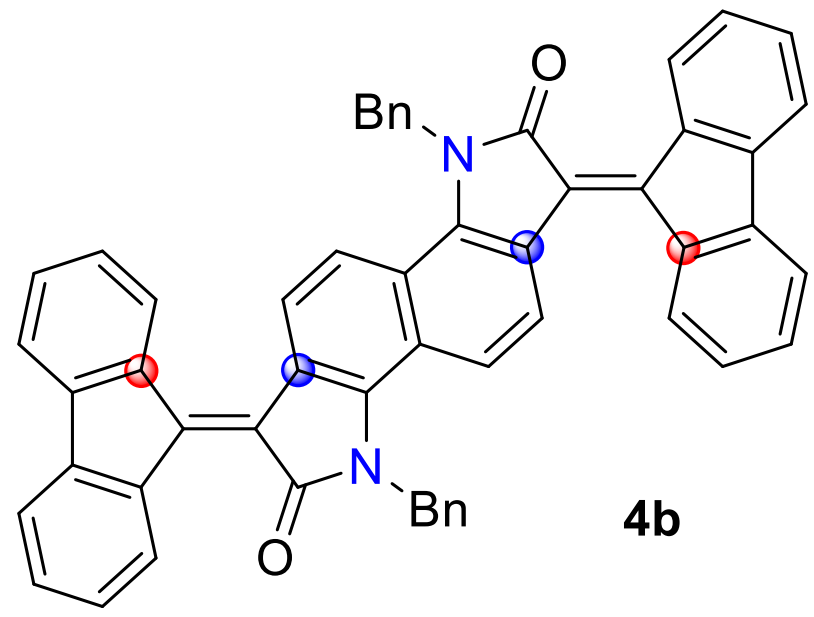

4b

Cartesian Coordinates (Angstrom):

C	-2.205403	-1.105845	-0.651358
C	-0.890291	-1.436489	-0.835221
C	0.163314	-0.620852	-0.336606
C	-0.163314	0.620852	0.336606
C	-1.542973	0.934406	0.485523
C	-2.555239	0.057662	0.064198
H	-2.975386	-1.736646	-1.071384
H	-0.666654	-2.340316	-1.373498
C	1.542973	-0.934406	-0.485523
C	0.890291	1.436489	0.835221
C	2.205403	1.105845	0.651358
C	2.555239	-0.057662	-0.064198
H	0.666654	2.340316	1.373498
H	2.975386	1.736646	1.071384
N	-2.146534	2.105730	0.979764
C	-3.539481	2.037695	0.832140
C	-1.576210	3.278280	1.629964
C	-3.850694	0.675340	0.295756
O	-4.283035	2.933781	1.188628
H	-0.917092	2.964613	2.442495
H	-2.433921	3.776799	2.089351
C	-0.872256	4.270976	0.718622
C	-5.117256	0.143143	0.183077
C	-1.139425	4.341548	-0.648862
C	0.029511	5.182089	1.277382
C	-6.397538	0.846807	-0.074534
C	-5.464310	-1.298532	0.235581
C	-0.515895	5.302229	-1.442812
H	-1.834313	3.641662	-1.097419
C	0.650619	6.144952	0.486734
H	0.246906	5.139311	2.340685
C	-6.708192	2.198170	-0.250197
C	-7.409921	-0.124576	-0.284963
C	-4.733341	-2.404613	0.673977

C	-6.840372	-1.452615	-0.052089
C	0.379922	6.207192	-0.879046
H	-0.732689	5.342757	-2.504459
H	1.346960	6.843992	0.936496
C	-8.007051	2.558891	-0.612194
H	-5.964068	2.958414	-0.074592
C	-8.697713	0.240851	-0.651344
C	-5.349196	-3.655400	0.731841
H	-3.702851	-2.307566	0.985142
C	-7.450857	-2.699223	0.001849
H	0.863298	6.954677	-1.497826
C	-8.995068	1.595389	-0.810667
H	-8.248310	3.608579	-0.734964
H	-9.461025	-0.511052	-0.818114
C	-6.690672	-3.807451	0.379220
H	-4.776992	-4.514366	1.062611
H	-8.506287	-2.810474	-0.220188
H	-9.997614	1.898368	-1.091373
H	-7.152397	-4.787330	0.425729
N	2.146534	-2.105730	-0.979764
C	3.539481	-2.037695	-0.832140
C	1.576210	-3.278280	-1.629964
C	3.850694	-0.675340	-0.295756
O	4.283035	-2.933781	-1.188628
H	0.917092	-2.964613	-2.442495
H	2.433921	-3.776799	-2.089351
C	0.872256	-4.270976	-0.718622
C	5.117256	-0.143143	-0.183077
C	-0.029511	-5.182089	-1.277382
C	1.139425	-4.341548	0.648862
C	6.397538	-0.846807	0.074534
C	5.464310	1.298532	-0.235581
C	-0.650619	-6.144952	-0.486734
H	-0.246906	-5.139311	-2.340685
C	0.515895	-5.302229	1.442812
H	1.834313	-3.641662	1.097419
C	6.708192	-2.198170	0.250197
C	7.409921	0.124576	0.284963
C	4.733341	2.404613	-0.673977
C	6.840372	1.452615	0.052089
C	-0.379922	-6.207192	0.879046
H	-1.346960	-6.843992	-0.936496

H	0.732689	-5.342757	2.504459
C	8.007051	-2.558891	0.612194
H	5.964068	-2.958414	0.074592
C	8.697713	-0.240851	0.651344
C	5.349196	3.655400	-0.731841
H	3.702851	2.307566	-0.985142
C	7.450857	2.699223	-0.001849
H	-0.863298	-6.954677	1.497826
C	8.995068	-1.595389	0.810667
H	8.248310	-3.608579	0.734964
H	9.461025	0.511052	0.818114
C	6.690672	3.807451	-0.379220
H	4.776992	4.514366	-1.062611
H	8.506287	2.810474	0.220188
H	9.997614	-1.898368	1.091373
H	7.152397	4.787330	-0.425729

****

Structure: **4c**

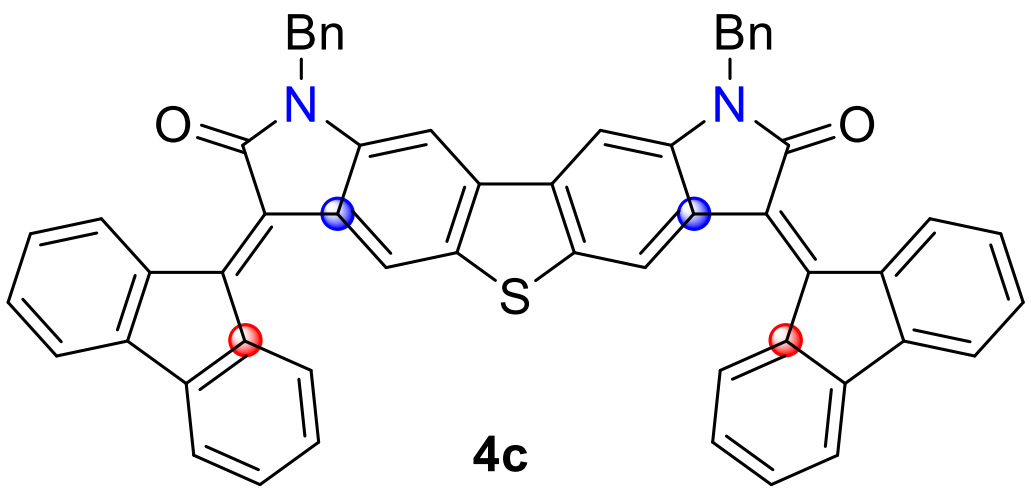

4c

Charge = 0, Multiplicity = 1
Final SCF Energy = -2813.23823525 Hartree
Gibbs Free Energy = -2812.589072 Hartree
Thermal Correction = 0.649164 Hartree
Imaginary Frequencies = none
Cartesian Coordinates (Angstrom):

C	-0.135019	-2.948014	-0.954400
C	-0.239745	-3.489951	0.358322
C	-0.155958	-2.624773	1.448218
C	-0.074943	-1.255352	1.206374
C	-0.040601	-0.720689	-0.101913
C	-0.050609	-1.594424	-1.205202
H	-0.147199	-3.001218	2.461067
H	0.045348	-1.215509	-2.214021
C	0.074943	1.255352	1.206374
C	0.155958	2.624773	1.448218
C	0.239745	3.489951	0.358322
C	0.135019	2.948014	-0.954400
C	0.050609	1.594424	-1.205202
C	0.040601	0.720689	-0.101913
H	0.147199	3.001218	2.461067
H	-0.045348	1.215509	-2.214021
S	-0.000000	0.000000	2.448096

C	-0.230210	-4.948206	0.244078
C	-0.056926	-5.229815	-1.228389
C	-0.428274	-5.910130	1.208553
O	-0.000000	-6.289145	-1.826681
N	-0.068365	-3.995412	-1.872405
C	0.065274	-7.311006	1.237993
C	-1.134320	-5.716926	2.501249
C	-0.073691	-3.867753	-3.320423
C	0.811376	-8.065636	0.328358
C	-0.216339	-7.859194	2.515588
C	-1.998538	-4.709897	2.935328
C	-0.995936	-6.886864	3.282430
H	-0.937462	-3.265806	-3.620833
H	-0.227168	-4.881989	-3.697860
C	1.194553	-3.278200	-3.914058
C	1.246525	-9.340850	0.692667
H	1.019261	-7.683916	-0.658159
C	0.226176	-9.124960	2.874299
C	-2.635286	-4.835005	4.170497
H	-2.200144	-3.843279	2.321691
C	-1.624201	-7.007250	4.515923
C	2.456280	-3.684374	-3.471055
C	1.112287	-2.350090	-4.954637
C	0.959735	-9.870209	1.950091
H	1.813161	-9.928364	-0.020722
H	0.012131	-9.528533	3.857698
C	-2.433042	-5.963300	4.966361
H	-3.300667	-4.049104	4.508633
H	-1.511930	-7.907121	5.110195
C	3.609918	-3.170363	-4.055897
H	2.534675	-4.406212	-2.665948
C	2.266386	-1.838589	-5.546076
H	0.137780	-2.025641	-5.307073
H	1.308283	-10.862989	2.211936
H	-2.930096	-6.041499	5.926672
C	3.519170	-2.246523	-5.096145
H	4.582097	-3.493366	-3.700743
H	2.185709	-1.120755	-6.354888
H	4.418751	-1.847655	-5.550928
C	0.230210	4.948206	0.244078
C	0.056926	5.229815	-1.228389
C	0.428274	5.910130	1.208553

O	0.000000	6.289145	-1.826681
N	0.068365	3.995412	-1.872405
C	-0.065274	7.311006	1.237993
C	1.134320	5.716926	2.501249
C	0.073691	3.867753	-3.320423
C	-0.811376	8.065636	0.328358
C	0.216339	7.859194	2.515588
C	1.998538	4.709897	2.935328
C	0.995936	6.886864	3.282430
H	0.937462	3.265806	-3.620833
H	0.227168	4.881989	-3.697860
C	-1.194553	3.278200	-3.914058
C	-1.246525	9.340850	0.692667
H	-1.019261	7.683916	-0.658159
C	-0.226176	9.124960	2.874299
C	2.635286	4.835005	4.170497
H	2.200144	3.843279	2.321691
C	1.624201	7.007250	4.515923
C	-2.456280	3.684374	-3.471055
C	-1.112287	2.350090	-4.954637
C	-0.959735	9.870209	1.950091
H	-1.813161	9.928364	-0.020722
H	-0.012131	9.528533	3.857698
C	2.433042	5.963300	4.966361
H	3.300667	4.049104	4.508633
H	1.511930	7.907121	5.110195
C	-3.609918	3.170363	-4.055897
H	-2.534675	4.406212	-2.665948
C	-2.266386	1.838589	-5.546076
H	-0.137780	2.025641	-5.307073
H	-1.308283	10.862989	2.211936
H	2.930096	6.041499	5.926672
C	-3.519170	2.246523	-5.096145
H	-4.582097	3.493366	-3.700743
H	-2.185709	1.120755	-6.354888
H	-4.418751	1.847655	-5.550928

****

Structure: **4d**

Charge = 0, Multiplicity = 1

Final SCF Energy = -2706.33610264 Hartree

Gibbs Free Energy = -2705.514850 Hartree

Thermal Correction = 0.821253 Hartree

Imaginary Frequencies = none

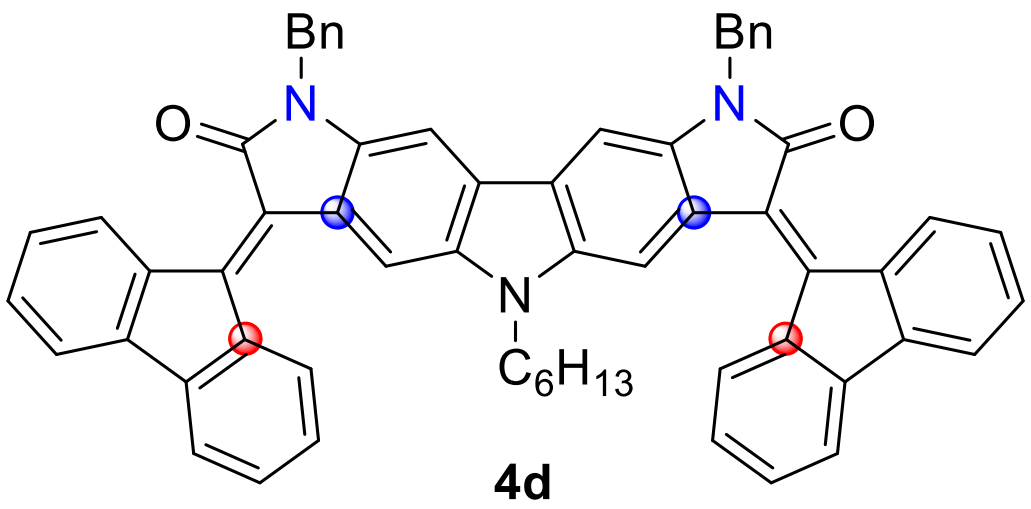

4d

Cartesian Coordinates (Angstrom):

C	2.949359	1.762203	-0.190760
C	3.382633	0.437670	-0.492732
C	2.445612	-0.596573	-0.533976
C	1.101485	-0.265162	-0.370381
C	0.676055	1.072290	-0.133091
C	1.624056	2.103940	-0.021549
H	2.754461	-1.621800	-0.675371
H	1.326879	3.114040	0.227978
C	-1.144210	-0.322480	-0.177912
C	-2.478847	-0.720170	-0.115300
C	-3.441574	0.260704	0.131269
C	-3.042358	1.626775	0.219277
C	-1.726164	2.034192	0.164388
C	-0.752563	1.036625	-0.015784
H	-2.761166	-1.752504	-0.260446
H	-1.454088	3.080263	0.215169
C	4.844433	0.438787	-0.512063
C	5.248429	1.848706	-0.146570
C	5.723512	-0.559208	-0.867257
O	6.356639	2.345537	-0.042995
N	4.073872	2.579481	-0.031396
C	7.123391	-0.768272	-0.416385
C	5.417967	-1.720838	-1.741211
C	4.062918	4.019316	0.162487
C	7.959804	-0.034650	0.430049
C	7.562351	-2.033424	-0.884728
C	4.370972	-1.946318	-2.637411
C	6.522030	-2.603850	-1.740229
H	3.454907	4.478927	-0.623590
H	5.097652	4.338574	0.013638
C	3.570333	4.476386	1.525385
C	9.206036	-0.556081	0.780091
H	7.662650	0.940726	0.780290
C	8.800566	-2.549582	-0.526254

C	4.388960	-3.083816	-3.445084
H	3.556696	-1.241474	-2.730757
C	6.534468	-3.742080	-2.538407
C	4.002365	3.847103	2.696259
C	2.710514	5.572147	1.627594
C	9.627835	-1.799355	0.309926
H	9.857123	0.022664	1.425428
H	9.119878	-3.522011	-0.884540
C	5.450213	-3.988659	-3.380520
H	3.574645	-3.257792	-4.139247
H	7.384450	-4.415230	-2.530658
C	3.580542	4.304149	3.941244
H	4.672634	2.997470	2.631030
C	2.290740	6.035117	2.873733
H	2.367053	6.069337	0.725360
H	10.600410	-2.184717	0.595061
H	5.445840	-4.870742	-4.010940
C	2.723828	5.400309	4.034526
H	3.922978	3.805463	4.841132
H	1.623497	6.887685	2.935895
H	2.396197	5.755076	5.005131
C	-4.902796	0.220470	0.143573
C	-5.342777	1.665908	0.186864
C	-5.756850	-0.857515	0.201986
O	-6.463340	2.144423	0.219179
N	-4.187234	2.427827	0.291529
C	-7.151677	-0.965708	-0.296787
C	-5.423504	-2.207614	0.723537
C	-4.212966	3.863807	0.511122
C	-8.004774	-0.046377	-0.914273
C	-7.561266	-2.320255	-0.196761
C	-4.371006	-2.646820	1.529343
C	-6.507627	-3.080460	0.475080
H	-3.612972	4.098440	1.396406
H	-5.254603	4.102235	0.741125
C	-3.738323	4.698366	-0.666725
C	-9.238780	-0.478948	-1.401578
H	-7.730123	0.994012	-0.981343
C	-8.787450	-2.745301	-0.690707
C	-4.363851	-3.961646	1.996187
H	-3.572735	-1.976503	1.815923
C	-6.494318	-4.393452	0.931831

C	-4.161888	4.414436	-1.968113
C	-2.905145	5.798923	-0.454643
C	-9.631861	-1.812616	-1.293538
H	-9.903095	0.240223	-1.867186
H	-9.084217	-3.785604	-0.615595
C	-5.404760	-4.837132	1.681198
H	-3.546649	-4.300468	2.622746
H	-7.329195	-5.056947	0.735850
C	-3.757751	5.213884	-3.033156
H	-4.811903	3.565187	-2.145961
C	-2.503219	6.604361	-1.519152
H	-2.568599	6.029795	0.551653
H	-10.595370	-2.126186	-1.679574
H	-5.380810	-5.857367	2.047329
C	-2.927603	6.312226	-2.812417
H	-4.093293	4.980961	-4.037596
H	-1.856556	7.455713	-1.337549
H	-2.613714	6.933980	-3.643185
N	-0.011691	-1.096682	-0.404261
C	0.012856	-2.538442	-0.593420
H	-0.908982	-2.828540	-1.105321
H	0.830882	-2.774468	-1.280245
C	0.170218	-3.337732	0.708007
H	1.091425	-3.023809	1.210625
H	-0.654384	-3.084379	1.383010
C	0.198033	-4.850186	0.463449
H	1.018144	-5.092457	-0.224698
H	-0.726532	-5.153960	-0.044113
C	0.360517	-5.670913	1.747841
H	1.282857	-5.366057	2.258369
H	-0.460521	-5.432368	2.436219
C	0.393205	-7.184493	1.507330
H	1.214192	-7.422030	0.820159
H	-0.528327	-7.488399	0.996202
C	0.554835	-7.997502	2.794814
H	1.485380	-7.740899	3.310500
H	-0.269938	-7.808099	3.488967
H	0.574928	-9.071049	2.589043

****

Structure: **6a**

Charge = 0, Multiplicity = 1

Final SCF Energy = -3676.84776771 Hartree

Gibbs Free Energy = -3675.795655 Hartree

Thermal Correction = 1.052112 Hartree

Imaginary Frequencies = none

Cartesian Coordinates (Angstrom):

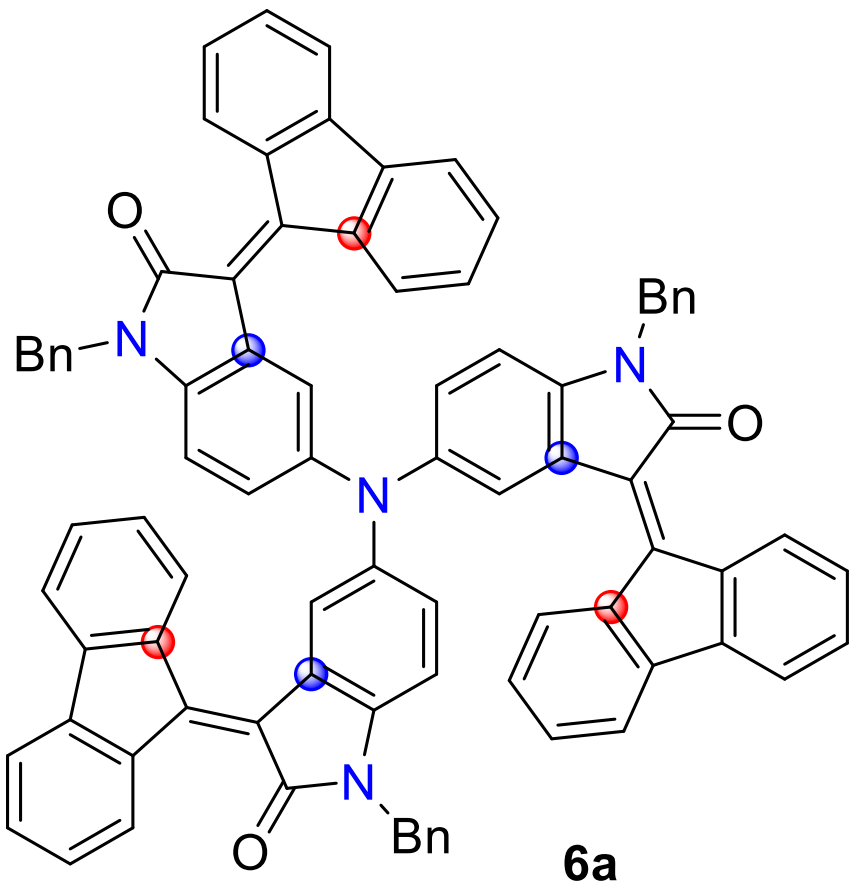

6a

C	-0.435322	1.311597	-2.652973
C	0.183523	1.994395	-1.598542
C	-1.593102	1.826491	-3.239904
C	-0.412171	3.134592	-1.071205
H	1.120508	1.629160	-1.204928
C	-2.183080	2.992356	-2.757934
H	-2.050106	1.276445	-4.056238
C	-1.592127	3.618100	-1.669714
C	0.000000	4.107044	-0.049526
H	-3.088644	3.387258	-3.204918
N	-1.992829	4.790537	-1.036440
C	-1.079256	5.168539	-0.067415
C	1.083685	4.123435	0.783014
C	-3.150358	5.583583	-1.381005
O	-1.230371	6.133586	0.657562
C	1.757883	5.315395	1.378223
C	1.895860	2.951669	1.224069
H	-3.146219	6.423800	-0.679744
H	-3.022474	5.993408	-2.391781
C	-4.466858	4.834541	-1.307326
C	1.518892	6.685618	1.264678
C	2.933715	4.882115	2.027724
C	1.656522	1.576809	1.178913
C	2.992109	3.418949	1.979323
C	-5.549814	5.313216	-2.047351
C	-4.633609	3.701547	-0.510921
C	2.430004	7.583001	1.822333
H	0.631994	7.056725	0.773810
C	3.843080	5.775936	2.573841
C	2.545724	0.701051	1.806309
H	0.781869	1.171263	0.682240
C	3.884780	2.551377	2.591256
C	-6.787341	4.681512	-1.982027
H	-5.421475	6.189614	-2.678378

C	-5.871597	3.065037	-0.450099
H	-3.793576	3.308388	0.055768
C	3.579149	7.140605	2.474022
H	2.234097	8.647293	1.743875
H	4.746110	5.418618	3.059909
C	3.663663	1.178052	2.487326
H	2.357772	-0.366352	1.775697
H	4.729141	2.933967	3.156621
C	-6.950296	3.553470	-1.181863
H	-7.620949	5.065665	-2.561295
H	-5.990723	2.181103	0.169254
H	4.272930	7.859169	2.898297
H	4.349823	0.476854	2.951514
H	-7.911637	3.052435	-1.133714
C	1.353537	-0.278798	-2.652973
C	1.635435	-1.156134	-1.598542
C	2.378338	0.466421	-3.239904
C	2.920721	-1.210345	-1.071205
H	0.850640	-1.784969	-1.204928
C	3.682997	0.394425	-2.757934
H	2.130487	1.137222	-4.056238
C	3.929430	-0.430227	-1.669714
C	3.556804	-2.053522	-0.049526
H	4.477774	0.981215	-3.204918
N	5.145142	-0.669428	-1.036440
C	5.015715	-1.649606	-0.067415
C	3.029157	-3.000216	0.783014
C	6.410703	-0.063501	-1.381005
O	5.927027	-2.001261	0.657562
C	3.724326	-4.180069	1.378223
C	1.608290	-3.117697	1.224069
H	7.136283	-0.487194	-0.679744
H	6.701681	-0.379164	-2.391781
C	6.420264	1.451142	-1.307326
C	5.030469	-4.658208	1.264678
C	2.761178	-4.981729	2.027724
C	0.537295	-2.222994	1.178913
C	1.464842	-4.300717	1.979323
C	7.376287	2.149672	-2.047351
C	5.522439	2.162050	-0.510921
C	5.352069	-5.895946	1.822333
H	5.795305	-4.075685	0.773810

C	3.080567	-6.216173	2.573841
C	-0.665734	-2.555187	1.806309
H	0.623409	-1.262750	0.682240
C	0.267167	-4.640007	2.591256
C	7.447979	3.537254	-1.982027
H	8.071100	1.600328	-2.678378
C	5.590199	3.552434	-0.450099
H	4.761936	1.631139	0.055768
C	4.394371	-6.669936	2.474022
H	6.371727	-6.258432	1.743875
H	2.319605	-6.819561	3.059909
C	-0.811609	-3.761852	2.487326
H	-1.496156	-1.858715	1.775697
H	0.176319	-5.562540	3.156621
C	6.552543	4.242398	-1.181863
H	8.197469	4.067103	-2.561295
H	4.884252	4.097567	0.169254
H	4.669775	-7.630050	2.898297
H	-1.761944	-4.005484	2.951514
H	6.599305	5.325461	-1.133714
C	-0.918215	-1.032799	-2.652973
C	-1.818959	-0.838262	-1.598542
C	-0.785236	-2.292912	-3.239904
C	-2.508551	-1.924246	-1.071205
H	-1.971148	0.155809	-1.204928
C	-1.499916	-3.386781	-2.757934
H	-0.080381	-2.413666	-4.056238
C	-2.337303	-3.187872	-1.669714
C	-3.556804	-2.053522	-0.049526
H	-1.389129	-4.368473	-3.204918
N	-3.152313	-4.121109	-1.036440
C	-3.936458	-3.518933	-0.067415
C	-4.112842	-1.123219	0.783014
C	-3.260345	-5.520081	-1.381005
O	-4.696656	-4.132325	0.657562
C	-5.482209	-1.135326	1.378223
C	-3.504150	0.166028	1.224069
H	-3.990064	-5.936606	-0.679744
H	-3.679206	-5.614244	-2.391781
C	-1.953406	-6.285683	-1.307326
C	-6.549361	-2.027410	1.264678
C	-5.694893	0.099614	2.027724

C	-2.193817	0.646186	1.178913
C	-4.456951	0.881768	1.979323
C	-1.826473	-7.462888	-2.047351
C	-0.888829	-5.863597	-0.510921
C	-7.782073	-1.687055	1.822333
H	-6.427300	-2.981039	0.773810
C	-6.923647	0.440237	2.573841
C	-1.879990	1.854136	1.806309
H	-1.405278	0.091487	0.682240
C	-4.151947	2.088630	2.591256
C	-0.660637	-8.218766	-1.982027
H	-2.649625	-7.789942	-2.678378
C	0.281399	-6.617471	-0.450099
H	-0.968360	-4.939527	0.055768
C	-7.973520	-0.470669	2.474022
H	-8.605824	-2.388861	1.743875
H	-7.065716	1.400943	3.059909
C	-2.852055	2.583800	2.487326
H	-0.861616	2.225067	1.775697
H	-4.905460	2.628573	3.156621
C	0.397753	-7.795868	-1.181863
H	-0.576520	-9.132768	-2.561295
H	1.106471	-6.278669	0.169254
H	-8.942704	-0.229119	2.898297
H	-2.587879	3.528630	2.951514
H	1.312333	-8.377896	-1.133714
N	0.000000	0.000000	-3.043183

****

Structure: **2p**

Charge = 0, Multiplicity = 1

Final SCF Energy = -1928.87712578 Hartree

Gibbs Free Energy = -1928.559489 Hartree

Thermal Correction = 0.317636 Hartree

Imaginary Frequencies = none

Cartesian Coordinates (Angstrom):

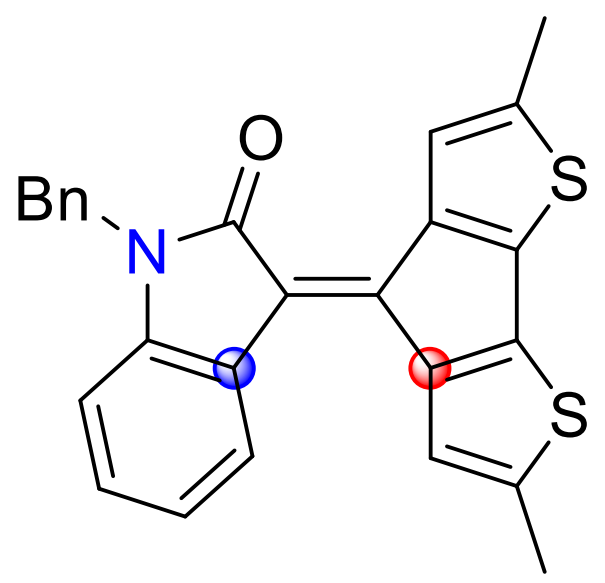

2p

C	-3.283178	1.253814	0.123760
C	-1.917101	1.380733	-0.090588
C	-1.321650	0.020570	-0.118594
C	-2.477799	-0.901498	0.023503
C	-3.629676	-0.144159	0.178287
C	-2.777375	-2.283610	-0.173799

C	-4.115402	-2.564957	-0.118391
C	-1.500373	2.745676	-0.070897
H	-0.486091	3.066027	-0.246386
C	-2.530853	3.620512	0.138590
H	-2.049188	-3.056581	-0.369707
S	-5.060940	-1.098079	0.142081
S	-4.064228	2.769083	0.342574
C	-4.793204	-3.896045	-0.225731
H	-5.290813	-4.179809	0.707391
H	-5.546964	-3.910314	-1.018885
H	-4.053277	-4.665676	-0.455010
C	-2.487413	5.117154	0.170529
H	-1.455111	5.451919	0.052064
H	-3.077626	5.562617	-0.636811
H	-2.864236	5.519759	1.116051
C	-0.000274	-0.323551	-0.212394
C	0.673838	-1.589690	0.075025
C	1.068703	0.579641	-0.777260
C	2.014067	-1.471538	-0.362501
C	0.302912	-2.736385	0.778158
C	2.938144	-2.493548	-0.201688
C	1.223318	-3.771538	0.949294
H	-0.681780	-2.818611	1.215059
C	2.519039	-3.658018	0.447142
H	3.961310	-2.383066	-0.536064
H	0.930595	-4.663726	1.490017
H	3.223724	-4.469991	0.585853
O	1.006777	1.740910	-1.137677
N	2.217529	-0.198082	-0.895027
C	3.441908	0.295875	-1.507009
H	3.157478	1.219792	-2.016465
H	3.769614	-0.418614	-2.268239
C	4.573453	0.567712	-0.530759
C	4.359652	1.345966	0.611263
C	5.856603	0.078979	-0.782845
C	5.407951	1.623041	1.483074
H	3.369896	1.740620	0.812130
C	6.909783	0.359089	0.086979
H	6.036399	-0.524275	-1.667664
C	6.686748	1.130347	1.223837
H	5.228201	2.227671	2.365013
H	7.900357	-0.029018	-0.122495

H 7.502351 1.347083 1.904354

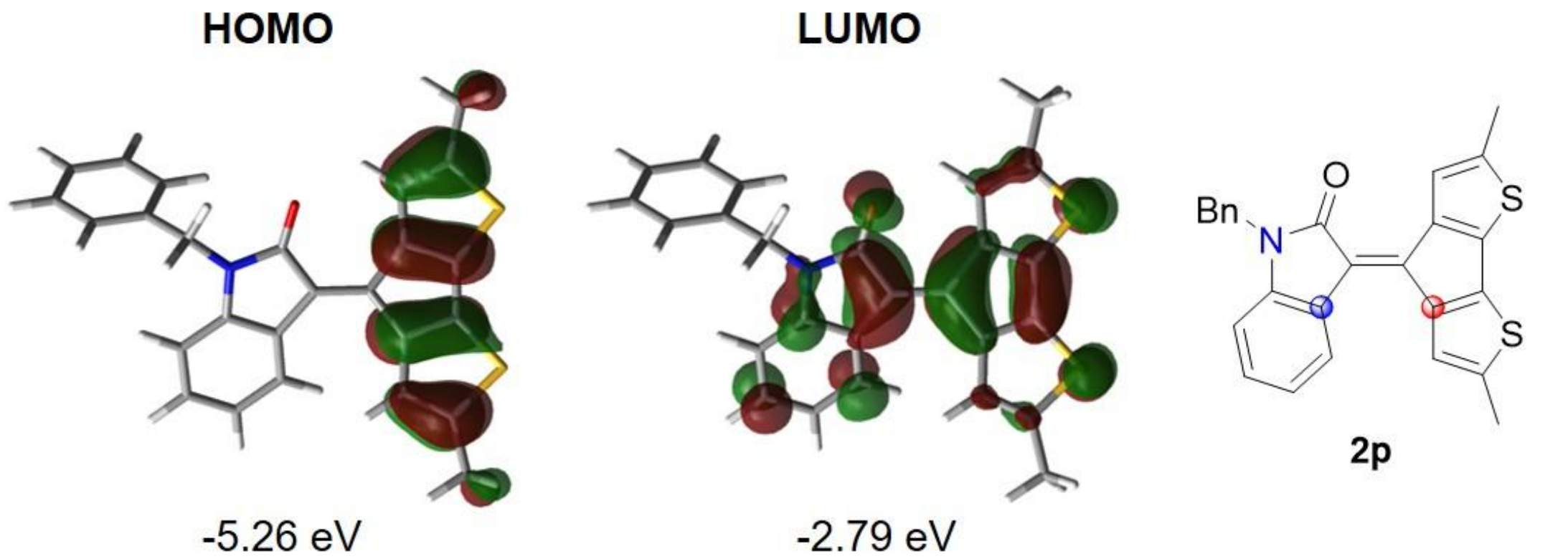

Figure S5. DFT calculations of HOMO and LUMO contours, and energy levels of **2p**.

9.6 Summary of HOMO and LUMO energies (calculated at B3LYP/6-311(d,p) level)

Table S3

compound	HOMO (eV)	LUMO (eV)
**2a**	-5.76	-2.84
**4b**	-5.24	-3.18
**4c**	-5.52	-3.13
**4d**	-5.30	-3.04
**6a**	-5.16	-3.03
**2p**	-5.26	-2.79

9.7 TD-DFT excitation energies and oscillator strengths for 2a, 4b, 4c, 4d, 6a, and 2n using the ground-state geometry at the CAM-B3LYP/6-311+G(2d,p) level

Table S4. Compound 2a

	E (eV)	l (nm)	*f*	dominant excitations
S1	2.8494	435.12	0.3048	H→L (0.68947)
S2	2.9916	414.44	0.0020	H-1→L (0.69249)
S3	3.4516	359.21	0.1921	H-2→L (0.68171)
S4	4.0080	309.34	0.0551	H-4→L (0.46627), H-7→L (0.42337)
S5	4.1378	299.64	0.0495	H-4→L (0.43045), H-7→L (-0.42852)

Table S5. Compound 4b

	E (eV)	λ (nm)	*f*	dominant excitations
S1	2.1830	567.95	0.6572	H→L (0.67579)
S2	2.6789	462.82	0.0000	H→L+1 (0.62509)
S3	2.8844	429.84	0.0224	H-3→L (0.51881), H-1→L (0.34495)

S4	2.8886	429.21	0.0000	H-2→L (0.46100), H-3→L+1 (0.39808)
S5	3.0051	412.58	0.6567	H-1→L (0.61378)

Table S6. Compound 4c

	E (eV)	λ (nm)	*f*	dominant excitations
S1	2.4941	497.12	0.9709	H→L (0.64254)
S2	2.7195	455.91	0.0883	H-1→L (0.59195), H→L+1 (0.30959)
S3	2.9176	424.95	0.0161	H-2→L (0.48956), H-3→L+1 (0.41727)
S4	2.9248	423.90	0.0006	H-3→L (0.53468), H-2→L+1 (0.37145)
S5	3.0102	411.87	0.1462	H→L+1 (0.47308), H-4→L (-0.37380)

Table S7. Compound 4d

	E (eV)	λ (nm)	*f*	dominant excitations
S1	2.3628	524.73	0.7814	H→L (0.64146)
S2	2.4596	504.08	0.0413	H-1→L (0.65189)
S3	2.8686	432.22	0.1953	H→L+1 (0.56495), H-2→L (0.33960)
S4	2.9212	424.43	0.3317	H-1→L+1 (0.55811), H-5→L (0.26718)
S5	2.9880	414.95	0.0080	H-4→L (0.50613), H-3→L+1 (0.42261)

Table S8. Compound 6a

	E (eV)	λ (nm)	*f*	dominant excitations
S1	2.4401	508.12	0.0496	H→L (0.64638)
S2	2.6049	475.96	0.1033	H→L+1 (0.46231), H→L+2 (-0.38049)
S3	2.6049	475.96	0.1033	H→L+2 (0.46231), H→L+1 (-0.38049)
S4	3.0380	408.11	0.0096	H-4→L+2 (0.33689), H-6→L+1 (0.33603)
S5	3.0380	408.11	0.0096	H-5→L (0.33689), H-6→L+2 (0.33603)

Table S9. Compound 2p

	E (eV)	λ (nm)	*f*	dominant excitations
S1	2.0654	600.30	0.0149	H→L (0.69776)
S2	2.9942	414.08	0.3121	H-1→L (0.67943)
S3	3.5963	344.76	0.3595	H-2→L (0.66930)
S4	4.0700	304.63	0.0629	H-3→L (0.54676), H-8→L (-0.35644)
S5	4.1729	297.12	0.0352	H-8→L (0.46544), H-3→L (0.39629)

10. Analytical data of starting substrates and products

3-(biphenyl-2-yl)-*N*-benzyl-*N*-phenylpropiolamide (1a)

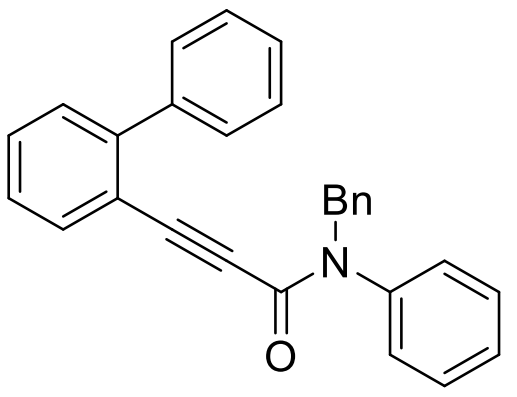

Purified by column chromatography (silica gel), [Hexane/EtOAc = 20:1] to give **1a** as a yellow solid (0.92 g, 79% yield, *rotamer ratio = 4:1*); ^{1}H NMR (400 MHz, $CDCl_3$, *major rotamer*) δ 7.78–7.43 (m, 1H), 7.43–7.15 (m, 16H), 7.10–6.87 (m, 2H), 4.94 (s, 2H); ^{13}C NMR (100 MHz, $CDCl_3$, *major rotamer*) δ 154.3, 144.5, 141.4, 139.7, 136.8, 134.0, 130.0, 129.7, 129.1(0), 129.0(8), 128.8, 128.6, 128.4, 128.3, 128.0, 127.9, 127.7, 127.0, 119.0, 91.4, 85.1, 52.5 cm^{-1}; HRMS (ESI_positive) m/z: $[M+Na]^+$ Calc for $C_{28}H_{21}NNaO^+$ 410.1515; Found 410.1515. mp. 79.7-81.6 °C.

3-(biphenyl-2-yl)-*N*-phenylpropiolamide (1b)

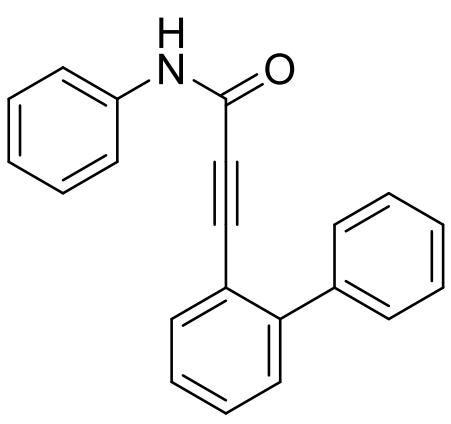

Purified by column chromatography (silica gel), [Hexane/EtOAc = 20:1] to give **1b** as a yellow solid (0.83 g, 93% yield); ^{1}H NMR (400 MHz, $CDCl_3$) δ 7.69 (s, 1H), 7.64–7.54 (m, 3H), 7.53–7.38 (m, 7H), 7.32–7.25 (m, 3H), 7.13–7.06 (m, 1H). ^{13}C NMR (100 MHz, $CDCl_3$) δ 151.2, 145.6, 140.0, 137.7, 134.1, 130.7, 129.7, 129.4, 129.1, 128.3, 128.2, 127.4, 124.8, 119.9, 118.5, 86.4, 85.9. HRMS (FD$^+$ (eiFi)) m/z: [M] Calc for $C_{21}H_{15}NO$ 297.1153; Found 297.1152. mp. 134.6-137.3 °C.

3-(biphenyl-2-yl)-*N*-acetyl-*N*-phenylpropiolamide (1c)

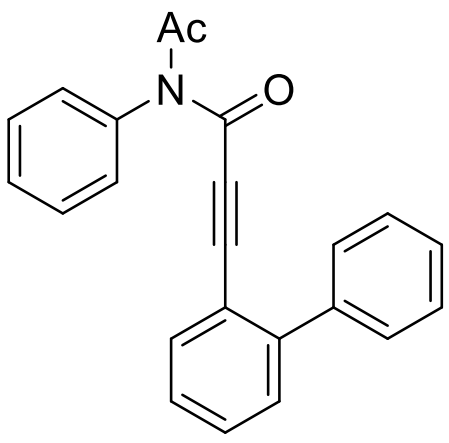

Purified by column chromatography (silica gel), [Hexane/EtOAc = 20:1] to give **1c** as a yellow solid (0.77 g, 76% yield); ^{1}H NMR (400 MHz, $CDCl_3$) δ 7.45–7.31 (m, 10H), 7.23–7.19 (m, 1H), 7.15–7.10 (m, 2H), 7.06–7.03 (m, 1H), 2.59 (s, 3H); ^{13}C NMR (100 MHz, $CDCl_3$) δ 172.5, 154.6, 145.2, 138.1, 134.4, 130.9, 129.8, 129.5, 129.4, 129.1, 129.1, 128.4, 128.2, 127.1, 118.1, 96.4, 85.2, 27.5. HRMS (FD$^+$ (eiFi)) m/z: [M] Calc for $C_{23}H_{17}NO_2$ 339.1259; Found 339.1258. mp. 77.2-79.9 °C.

3-(biphenyl-2-yl)-*N*-methyl-*N*-phenylpropiolamide (1d)

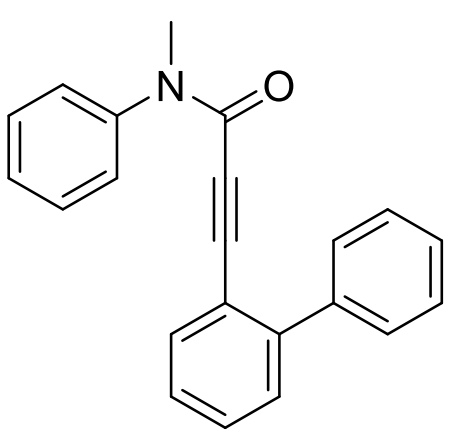

Purified by column chromatography (silica gel), [Hexane/EtOAc = 20:1] to give **1d** as a yellow solid (0.61 g, 66% yield, *rotamer ratio = 5:1*); ^{1}H NMR (400 MHz, $CDCl_3$, *major rotamer*) δ 7.78–7.26 (m, 9H), 7.26–7.08 (m, 5H), 3.30 (s, 3H); ^{13}C NMR (100 MHz, $CDCl_3$, *major rotamer*) δ 154.1, 144.4, 142.9, 139.7, 134.0, 130.0, 129.7, 129.2, 129.1, 128.3, 127.9, 127.7, 127.0(4), 127.0(0), 119.0, 90.7, 85.1, 36.5. HRMS (FD$^+$ (eiFi)) m/z: [M] Calc for $C_{22}H_{15}NO$ 309.1153; Found 309.1152.

3-(biphenyl-2-yl)-*N*,*N*-diphenylpropiolamide (1e)

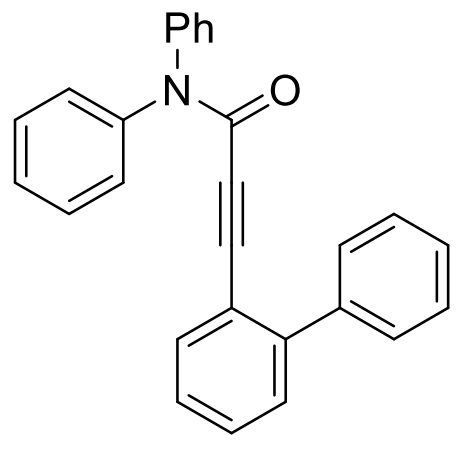

Purified by column chromatography (silica gel), [Hexane/EtOAc = 20:1] to give **1e** as a yellow solid (0.72 g, 64% yield); ^{1}H NMR (400 MHz, $CDCl_3$) δ 7.44–7.35 (m, 6H), 7.35–7.15 (m, 13H); ^{13}C NMR (100 MHz, $CDCl_3$) δ 153.6, 144.6, 139.7, 134.1, 130.1, 129.7, 129.1, 128.9, 128.3, 127.9, 127.0, 126.6, 126.1, 118.9, 92.3, 85.6, one aromatic sp^2 peak is not shown due to superimposition. HRMS (FD$^+$ (eiFi)) m/z: [M] Calc for $C_{27}H_{19}NO$ 373.1466; Found 373.1465. mp. 106.1-108.5 °C.

3-(biphenyl-2-yl)-*N*-benzyl-*N*-(*p*-tolyl)propiolamide (1f)

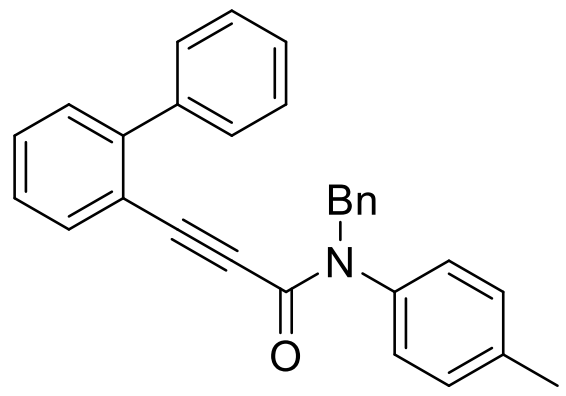

Purified by column chromatography (silica gel), [Hexane/EtOAc = 20:1] to give **1f** as a yellow liquid (1.05 g, 87% yield, *rotamer ratio = 4:1*); ^{1}H NMR (400 MHz, $CDCl_3$, *major rotamer*) δ 7.78–7.42 (m, 1H), 7.42–7.16 (m, 13H), 7.09–6.78 (m, 4H), 4.90 (s, 2H), 2.28 (s, 3H); ^{13}C NMR (100 MHz, $CDCl_3$, *major rotamer*) δ 154.4, 144.4, 139.7, 138.8, 137.7, 136.9, 134.1, 129.9, 129.7, 129.1, 128.8, 128.5, 128.2, 128.0, 127.9, 127.8, 127.6, 126.9, 119.1, 91.3, 85.2, 52.5, 21.2. HRMS (FD$^+$ (eiFi)) m/z: [M] Calc for $C_{29}H_{23}NO$ 401.1779; Found 401.1778.

3-(biphenyl-2-yl)-*N*-benzyl-*N*-(4-fluorophenyl)propiolamide (1g)

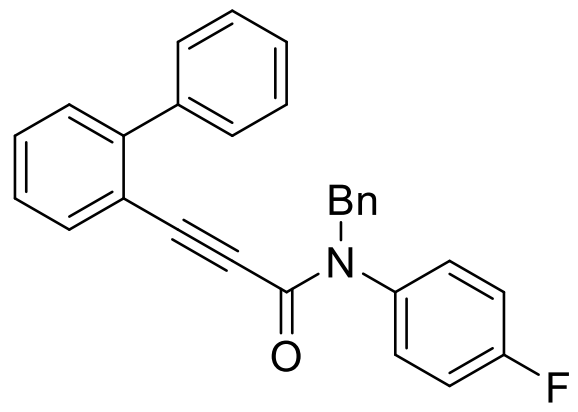

Purified by column chromatography (silica gel), [Hexane/EtOAc = 20:1] to give **1g** as a yellow liquid (1.14 g, 94% yield, *rotamer ratio = 4:1*); ^{1}H NMR (400 MHz, $CDCl_3$, *major rotamer*) δ 7.80–7.44 (m, 1H), 7.41–7.15 (m, 13H), 7.06–6.74 (m, 4H), 4.90 (s, 2H); ^{13}C NMR (100 MHz, $CDCl_3$, *major rotamer*) δ 161.8 (d, J_F = 247.7 Hz), 154.3, 144.6, 139.7, 137.2 (d, J_F = 3.2 Hz), 136.5, 134.1, 130.2, 130.1, 129.8, 129.1, 128.9, 128.7, 128.3, 127.8(9), 127.8(7) (d, J_F = 247.7 Hz), 127.1, 118.8, 116.0 (d, J_F = 22.8 Hz), 91.9, 84.8, 52.5. ^{19}F NMR (376 MHz, $CDCl_3$) δ -112.87. HRMS (FD$^+$ (eiFi)) m/z: [M] Calc for $C_{28}H_{20}FNO$ 405.1528; Found 405.1528.

methyl 4-(3-(biphenyl-2-yl)-*N*-benzylpropiolamido)benzoate (1h)

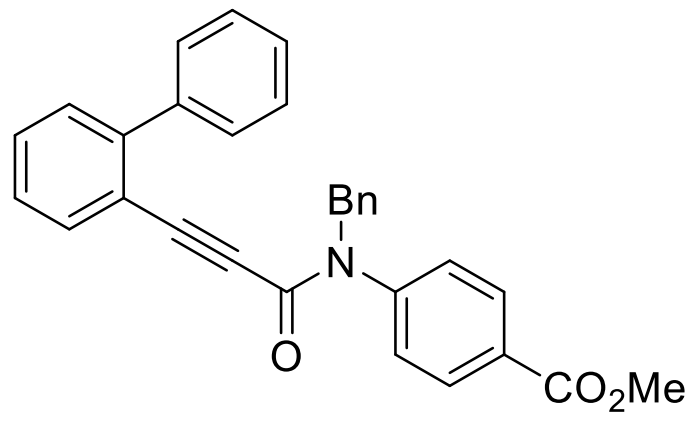

Purified by column chromatography (silica gel), [Hexane/EtOAc = 10:1] to give **1h** as a white solid (0.34 g, 42% yield, *rotamer ratio = 3:1*); ^{1}H NMR (400 MHz, $CDCl_3$, *major rotamer*) δ 8.04–7.64 (m, 2H), 7.58–7.08 (m, 14H), 7.08–6.89 (m, 2H), 4.94 (s, 2H), 3.89 (s, 3H); ^{13}C NMR (100 MHz, $CDCl_3$, *major rotamer*) δ 166.3, 153.8, 145.3, 144.6, 139.6, 136.4, 134.1, 130.5, 130.3, 129.8, 129.3, 129.0, 128.7, 128.6, 128.3, 127.9, 127.8, 127.5, 127.1, 126.3, 118.6, 91.8, 84.7, 52.3. HRMS (ESI_positive) m/z: [M+Na]$^+$ Calc for $C_{30}H_{23}NNaO_3^+$ 468.1570; Found 468.1570. mp. 92.4-93.6 °C.

3-(biphenyl-2-yl)-*N*-benzyl-*N*-(4-nitrophenyl)propiolamide (1i)

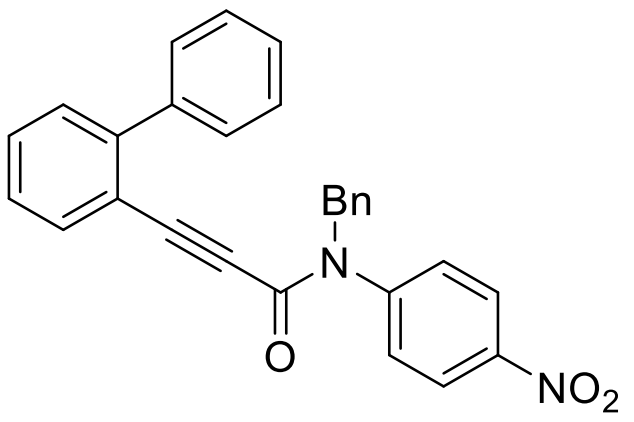

Purified by GPC to give **1i** as a white solid (0.11 g, 14% yield, *rotamer mixture*); ^{1}H NMR (400 MHz, $CDCl_3$, *both rotamers*) δ 8.20–7.67 (m, 2H), 7.67–6.84 (m, 16H), 5.07–4.74 (m, 2H); ^{13}C NMR (100 MHz, $CDCl_3$, *major rotamer*) δ 153.6, 146.6, 146.4, 144.8, 139.7, 135.9, 134.4, 130.6, 129.9, 129.0, 128.9, 128.4, 128.3, 128.1, 127.9, 127.3, 124.4, 118.3, 92.5, 84.2, 52.1, one aromatic sp^2 peak is not shown due to superimposition. HRMS (ESI_positive) m/z: $[M+Na]^+$ Calc for $C_{28}H_{20}N_2NaO_3^+$ 455.1366; Found 455.1366. mp. 94.9-96.3 °C.

3-(biphenyl-2-yl)-*N*-benzyl-*N*-(naphthalen-1-yl)propiolamide (1j)

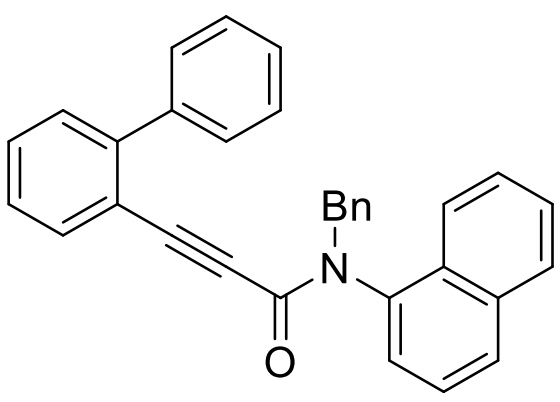

Purified by column chromatography (silica gel), [Hexane/EtOAc = 20:1] to give **1j** as a yellow solid (0.90 g, 69% yield, *rotamer ratio = 5:1*); ^{1}H NMR (400 MHz, $CDCl_3$, *major rotamer*) δ 7.92–7.60 (m, 3H), 7.58–7.38 (m, 3H), 7.30–7.21 (m, 6H), 7.21–7.12 (m, 6H), 7.08–6.75 (m, 3H), 5.66 (d, *J* = 14.0 Hz, 1H), 4.33 (d, *J* = 14.1 Hz, 1H); ^{13}C NMR (100 MHz, $CDCl_3$, *major rotamer*) δ 155.2, 144.3, 139.4, 137.3, 136.9, 134.6, 134.0, 130.7, 129.9, 129.5, 129.3, 129.0, 128.9, 128.6, 128.5, 128.1, 127.9, 127.8, 127.8, 127.3, 126.8, 126.6, 125.3, 122.8, 118.8, 90.9, 85.2, 52.2. HRMS (FD$^+$ (eiFi)) m/z: [M] Calc for $C_{32}H_{23}NO$ 437.1779; Found 437.1779. mp. 134.4-136.3 °C.

3-(biphenyl-2-yl)-*N*-benzyl-*N*-(phenanthren-9-yl)propiolamide (1k)

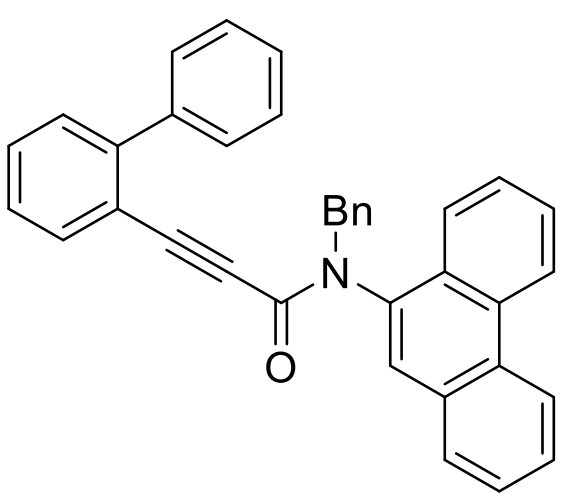

Purified by column chromatography (silica gel), [Hexane/EtOAc = 20:1] to give **1k** as a yellow solid (0.56 g, 38% yield, *rotamer ratio = 5:1*); ^{1}H NMR (400 MHz, $CDCl_3$, *major rotamer*) δ 8.79–8.58 (m, 2H), 7.94–7.83 (m, 1H), 7.76–7.25 (m, 9H), 7.24–6.92 (m, 10H), 6.80–6.72 (m, 1H), 5.72 (d, *J* = 14.1 Hz, 1H), 4.35 (d, *J* = 14.1 Hz, 1H); ^{13}C NMR (100 MHz, $CDCl_3$, *major rotamer*) δ 155.3, 144.3, 139.2, 136.9, 135.7, 134.1, 131.7, 131.1, 130.4, 129.8, 129.6, 129.4, 129.3, 129.2, 128.7, 128.6, 128.5, 128.4, 127.8, 127.6(4), 127.6(0), 127.5, 127.3, 127.0, 126.7, 123.6, 123.4, 122.7, 118.7, 90.9, 85.3, 52.1. HRMS (APCI_positive) m/z: $[M+H]^+$ Calc for $C_{36}H_{26}NO^+$ 488.2008; Found 488.2009. mp. 136.1-137.9 °C.

3-(biphenyl-2-yl)-*N*-benzyl-*N*-(2-(*p*-tolyl)benzo[*b*]thiophen-4-yl)propiolamide (1l)

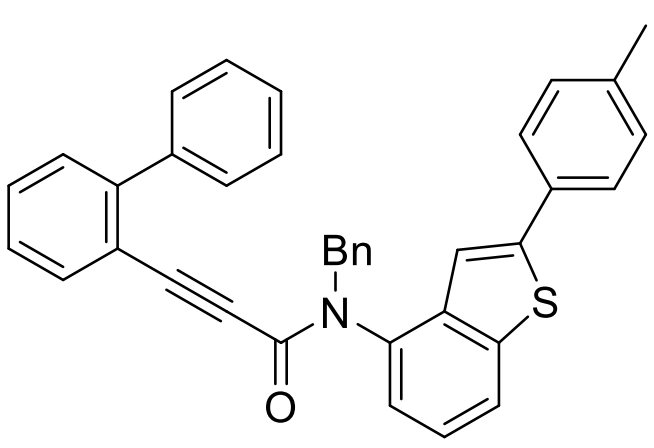

Purified by column chromatography (silica gel), [Hexane/EtOAc = 20:1] to give **1l** as a yellow solid (1.26 g, 79% yield); ^{1}H NMR (400 MHz, $CDCl_3$) δ 7.88–7.38 (m, 4H), 7.33–7.17 (m, 13H), 7.17–6.99 (m, 3H), 6.99–6.78 (m, 2H), 5.35 (d, *J* = 14.0 Hz, 1H), 4.70 (d, *J* = 14.1 Hz, 1H), 2.40 (s, 3H); ^{13}C NMR (100 MHz, $CDCl_3$) δ 154.7, 145.8, 144.4, 140.5, 139.4, 139.3, 138.9, 136.9, 135.6, 134.1, 131.0, 129.9, 129.8, 129.5, 129.4, 128.9, 128.5, 128.1, 127.9, 127.8, 126.9, 126.6, 125.7, 124.3, 122.6, 118.8, 115.9, 91.2, 85.1, 52.2, 21.4. HRMS (MALDI_positive_DIT) m/z: $[M+H]^+$ Calc for $C_{37}H_{28}NOS^+$ 534.1886; Found 534.1886. mp. 64.9-66.3 °C.

3-(biphenyl-2-yl)-*N*-benzyl-*N*-(9-methyl-9*H*-carbazol-4-yl)propiolamide (1m)

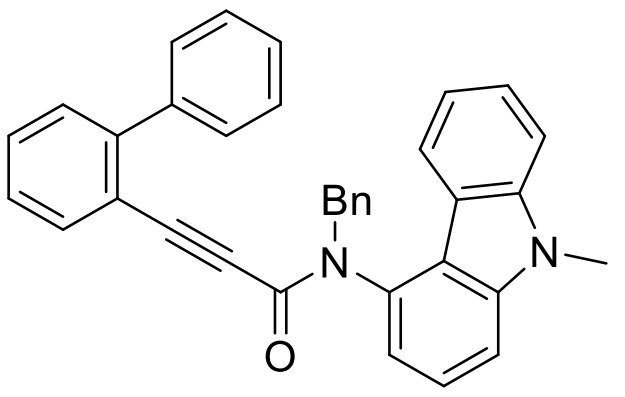

Purified by column chromatography (silica gel), [Hexane/EtOAc = 20:1] to give **1m** as a yellow solid (1.23 g, 84% yield, *rotamer ratio = 6:1*); ^{1}H NMR (400 MHz, $CDCl_3$, *major rotamer*) δ 8.02–7.82 (m, 1H), 7.63–7.31 (m, 3H), 7.31–7.09 (m, 12H), 7.09–6.91 (m, 4H), 6.61–6.54 (m, 1H), 5.92 (d, *J* = 14.1 Hz, 1H), 4.31 (d, *J* = 14.1 Hz, 1H), 3.87 (s, 3H); ^{13}C NMR (100 MHz, $CDCl_3$, *major rotamer*) δ 154.7, 144.3, 142.1, 141.1, 139.3, 136.9, 134.9, 134.0, 129.7, 129.4, 129.3, 128.8, 128.4, 127.8, 127.6, 127.5, 126.7, 126.4, 125.4, 122.3, 121.1, 120.6, 119.9, 119.6, 118.9, 108.7, 90.4, 85.2, 51.0, 29.4, one aromatic sp^2 peak is not shown due to superimposition. HRMS (MALDI_positive_DIT) m/z: $[M+H]^+$ Calc for $C_{35}H_{27}N_2O^+$ 491.2117; Found 494.2118. mp. 183.6-186.0 °C.

3-(biphenyl-2-yl)-*N*-benzyl-*N*-(9-methyl-9*H*-carbazol-2-yl)propiolamide (1n)

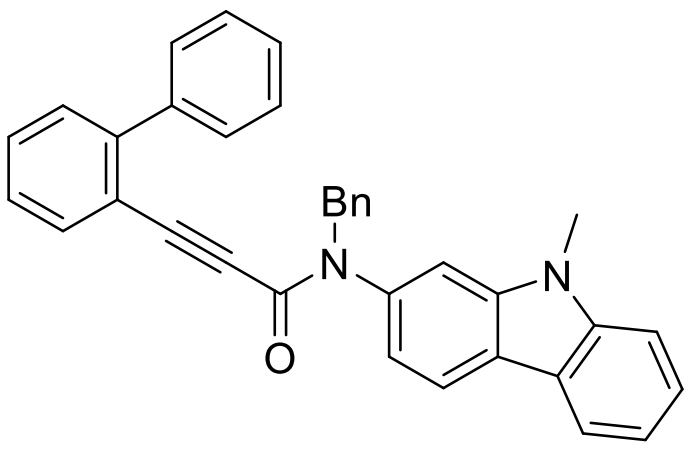

Purified by column chromatography (silica gel), [Hexane/EtOAc = 20:1] to give **1n** as a yellow solid (1.19 g, 81% yield, *rotamer ratio = 5:1*); ^{1}H NMR (400 MHz, $CDCl_3$, *major rotamer*) δ 7.99–7.70 (m, 2H), 7.53–7.32 (m, 3H), 7.29–7.16 (m, 10H), 7.14–6.97 (m, 6H), 5.01 (s, 2H), 3.80 (s, 3H); ^{13}C NMR (100 MHz, $CDCl_3$, *major rotamer*) δ 155.0, 144.2, 141.6, 140.1, 139.5, 137.0, 134.2, 132.9, 129.8, 129.6, 129.1, 128.9, 128.5, 127.9, 127.6, 127.4, 126.9, 126.5, 126.3, 122.9, 122.6, 120.6, 119.9, 119.3, 119.1, 108.8, 108.4, 91.6, 85.5, 53.2, 29.3. HRMS (MALDI_positive_DIT) m/z: $[M+H]^+$ Calc for $C_{35}H_{27}N_2O^+$ 491.2117; Found 494.2118. mp. 69.7-71.9 °C.

3-(binaphthalen-2-yl)-*N*-benzyl-*N*-phenylpropiolamide (1o)

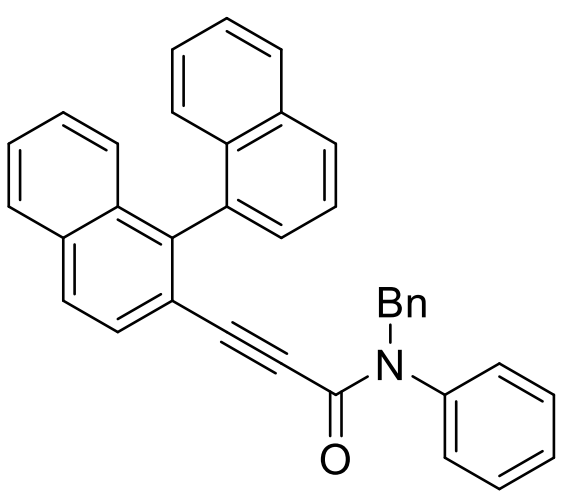

Purified by GPC to give **1o** as a yellow solid (0.16 g, 11% yield, *rotamer ratio = 1.4:1*); ^{1}H NMR (400 MHz, $CDCl_3$, *both rotamers*) δ 8.00–7.87 (m, 4.3H), 7.86–7.77 (m, 2.0H), 7.72–7.66 (m, 1.5H), 7.58–7.51 (m, 1.6H), 7.51–7.45 (m, 1.6H), 7.45–7.40 (m, 1.6H), 7.40–7.36 (m, 1.3H), 7.35–7.30 (m, 1.5H), 7.30–7.25 (m, 2.2H), 7.24–7.18 (m, 7.1H), 7.18–7.14 (m, 2.1H), 7.14–7.06 (m, 5.4H), 7.05–6.99 (m, 2.1H), 6.97–6.89 (m, 2.1H), 6.87–6.82 (m, 1.5H), 6.72–6.66 (m, 3.5H), 4.81 (s, 2H), 3.98–3.85 (m, 1.5H). ^{13}C NMR (100 MHz, $CDCl_3$, *major rotamer*) δ 154.3, 154.1, 143.5, 142.2, 141.1, 139.5, 136.7, 136.3, 136.1, 135.6, 134.0, 133.6, 133.5, 133.4, 132.8, 132.7, 132.5, 132.4, 129.2, 129.0, 128.8, 128.7, 128.6, 128.5, 128.4, 128.4, 128.3, 128.2, 128.1, 128.0, 127.9(4), 127.9(1), 127.7, 127.5, 127.4, 127.2, 127.1, 127.0, 126.9, 126.6, 126.3, 126.2, 126.1, 125.9, 125.6, 125.4, 118.8, 91.6, 90.8, 85.9, 85.6, 55.2, 52.4. HRMS (APCI_positive) m/z: $[M+H]^+$ Calc for $C_{36}H_{26}NO^+$ 488.2008; Found 488.2009. mp. 185.3-187.8 °C.

***N*-benzyl-3-(5,5'-dimethyl-[2,2'-bithiophen]-3-yl)-*N*-phenylpropiolamide (1p)**

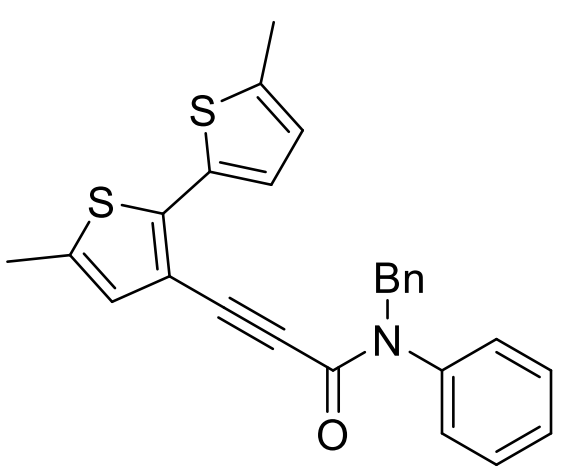

Purified by GPC to give **1p** as a yellow liquid (0.35 g, 27% yield, *rotamer ratio = 6:1*); ^{1}H NMR (400 MHz, $CDCl_3$, *major rotamer*) δ 7.36–7.20 (m, 8H), 7.19–7.09 (m, 2H), 6.91–6.71 (m, 1H), 6.62–6.49 (m, 1H), 6.31 (s, 1H), 4.99 (s, 2H), 2.43 (s, 3H), 2.30 (s, 3H); ^{13}C NMR (100 MHz, $CDCl_3$, *major rotamer*) δ 154.5, 141.7, 141.6, 140.6, 136.9, 136.7, 133.1, 129.3, 129.2, 128.8, 128.7, 128.6, 128.2, 127.7, 126.1, 125.6, 113.1, 88.2, 86.2, 52.6, 15.5, 15.2. HRMS (APCI_positive) m/z: $[M+H]^+$ Calc for $C_{26}H_{22}NOS_2^+$ 428.1137; Found 428.1137.

_N_-benzyl-3-(4'-methyl-biphenyl-2-yl)-_N_-phenylpropiolamide (1q)

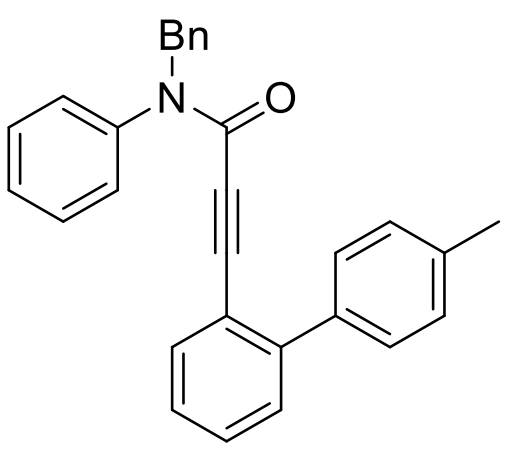

Purified by column chromatography (silica gel), [Hexane/EtOAc = 20:1] to give **1q** as a yellow liquid (0.62 g, 51% yield, *rotamer ratio = 3:1*); ^{1}H NMR (400 MHz, $CDCl_3$, *major rotamer*) δ 7.78–7.32 (m, 2H), 7.32–7.14 (m, 13H), 7.09–6.98 (m, 3H), 4.95 (s, 2H), 2.42 (s, 3H); ^{13}C NMR (100 MHz, $CDCl_3$, *major rotamer*) δ 154.4, 144.5, 141.5, 137.5, 136.8, 136.8, 134.1, 130.0, 129.6, 129.1, 129.0, 128.9, 128.8, 128.6, 128.4, 128.0, 127.7, 126.7, 118.9, 91.7, 85.0, 52.5, 21.4. HRMS (APCI_positive) m/z: $[M+H]^+$ Calc for $C_{29}H_{24}NO^+$ 402.1852; Found 402.1852.

3-(biphenyl-2-yl-2'-_d_)-_N_-benzyl-_N_-phenylpropiolamide (1a-_d_)

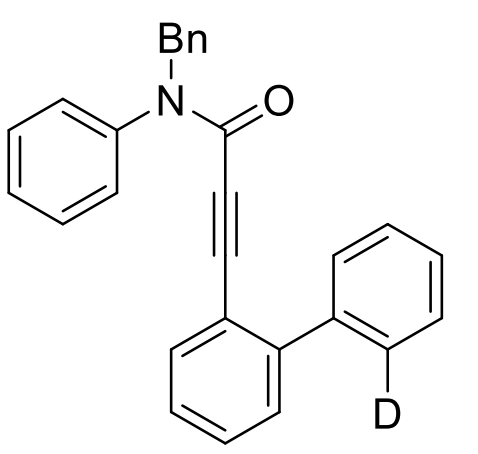

Purified by column chromatography (silica gel), [Hexane/EtOAc = 20:1] to give **1a-_d_** as a yellow solid (0.85 g, 73% yield, *rotamer ratio = 4:1*); ^{1}H NMR (400 MHz, $CDCl_3$, *major rotamer*) δ 7.78–7.32 (m, 6H), 7.32–7.15 (m, 10H), 7.09–6.94 (m, 2H), 4.94 (s, 2H); ^{13}C NMR (100 MHz, $CDCl_3$, *major rotamer*) δ 154.3, 144.4, 141.4, 139.6, 136.8, 134.0, 130.0, 129.7, 129.1, 128.8, 128.6, 128.4, 128.3, 128.1, 128.0, 127.8, 127.6, 127.0, 119.0, 91.4, 85.1, 52.5, sp^2 carbon adjacent to D cannot be identified due to their weak intensity and superimposition. HRMS (APCI_positive) m/z: $[M+H]^+$ Calc for $C_{28}H_{21}DNO^+$ 389.1758; Found 389.1759. mp. 81.6-84.3 °C.

3-(biphenyl-2-yl)-_N_-benzyl-_N_-(phenyl-2-_d_)propiolamide (1a'-_d_)

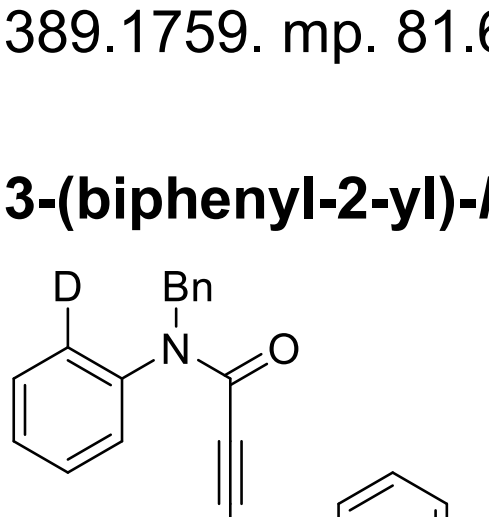

Purified by column chromatography (silica gel), [Hexane/EtOAc = 20:1] to give **1a'-_d_** as a yellow solid (0.92 g, 79% yield, *rotamer ratio = 4:1*); ^{1}H NMR (400 MHz, $CDCl_3$, *major rotamer*) δ 7.78–7.43 (m, 1H), 7.41–7.14 (m, 16H), 7.09–6.96 (m, 1H), 4.93 (s, 2H); ^{13}C NMR (100 MHz, $CDCl_3$, *major rotamer*) δ 154.3, 144.5, 141.4, 139.7, 136.8, 134.0, 130.0, 129.7, 129.09, 129.06, 129.0, 128.8, 128.6, 128.3, 128.3, 128.0, 127.8, 127.6, 127.0, 119.0, 91.4, 85.1, 52.5, sp^2 carbon adjacent to D cannot be identified due to their weak intensity and superimposition. HRMS (APCI_positive) m/z: $[M+H]^+$ Calc for $C_{28}H_{21}DNO^+$ 389.1758; Found 389.1759. mp. 81.1-82.9 °C.

3-(biphenyl-2-yl-2',3',4',5',6'-d_5)-_N_-benzyl-_N_-phenylpropiolamide (1a-d_5)

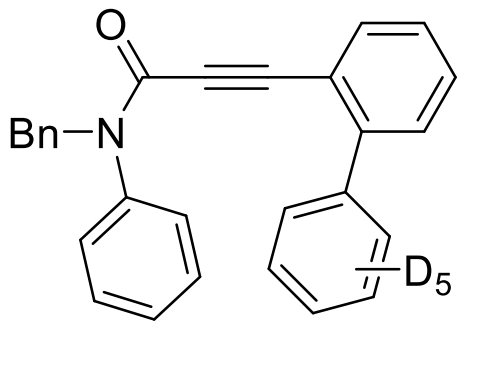

Purified by column chromatography (silica gel), [Hexane/EtOAc = 20:1] to give **1a-*d*₅** as a colorless oil (0.78 g, 77% yield, *rotamer ratio = 4:1*); ^{1}H NMR (400 MHz, $CDCl_3$, *major rotamer*) δ 7.80–7.43 (m, 1H), 7.43–7.12 (m, 11H), 7.09–6.95 (m, 2H), 4.93 (s, 2H); ^{13}C NMR (100 MHz, $CDCl_3$, *major rotamer*) δ 154.3, 144.4, 141.4, 139.5, 136.8, 134.1, 130.0, 129.7, 129.1, 129.1, 128.8, 128.6, 128.4, 128.0, 127.8, 127.7, 127.0, 126.9, 119.0, 91.4, 85.1, 52.5, C–D coupling was not resolved due to rotamer-induced signal overlap. HRMS (ESI_positive) m/z: $[M+Na]^+$ Calc for $C_{28}H_{16}D_5NNaO^+$ 415.1829; Found 415.1829.

3-(biphenyl-2-yl)-*N*-benzyl-*N*-(phenyl-*d*₅)propiolamide (1a'-*d*₅)

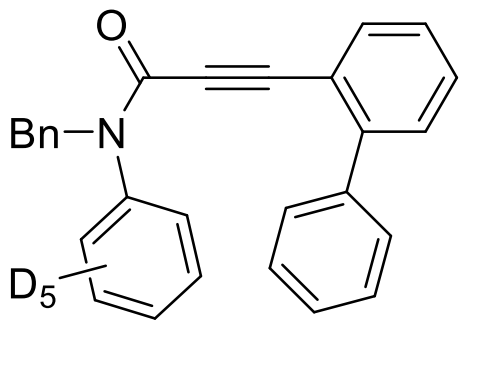

Purified by column chromatography (silica gel), [Hexane/EtOAc = 20:1] to give **1a'-*d*₅** as a yellow oil (0.81 g, 81% yield, *rotamer ratio = 4:1*); ^{1}H NMR (400 MHz, $CDCl_3$, *major rotamer*) δ 7.80–7.44 (m, 1H), 7.44–6.99 (m, 13H), 7.09–6.95 (m, 2H); ^{13}C NMR (100 MHz, $CDCl_3$, *major rotamer*) δ 154.3, 144.5, 141.3, 139.7, 136.8, 134.1, 130.0, 129.7, 129.1, 128.8, 128.6, 128.3, 127.9, 127.7, 127.0, 119.0, 91.4, 85.1, 52.5, three sp^2 carbons adjacent to D cannot be identified due to their weak intensity and superimposition. HRMS (ESI_positive) m/z: $[M+Na]^+$ Calc for $C_{28}H_{16}D_5NNaO^+$ 415.1829; Found 415.1829.

***N*,*N'*-(1,4-phenylene)bis(3-(biphenyl-2-yl)-*N*-benzylpropiolamide) (3a)**

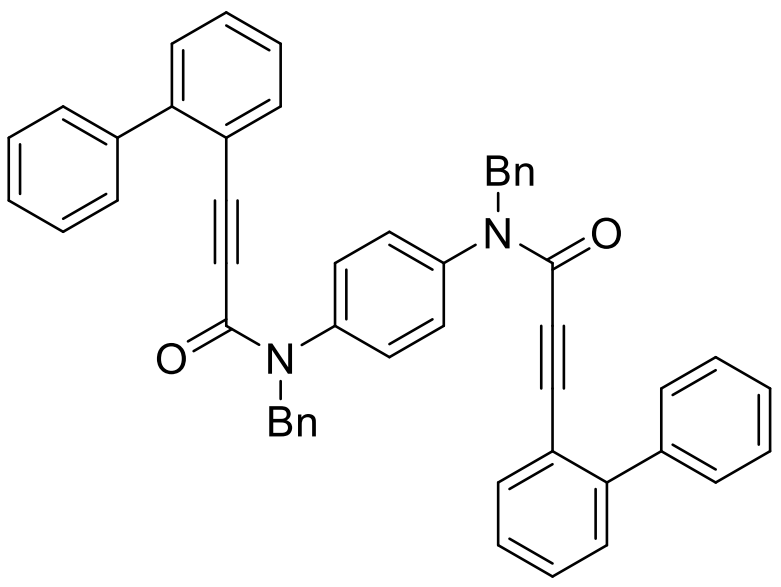

Purified by column chromatography (silica gel), [Hexane/EtOAc = 20:1] to give **3a** as a yellow solid (1.27 g, 61% yield, *rotamer mixture*); ^{1}H NMR (400 MHz, $CDCl_3$, *all rotamers*) δ 7.79–6.80 (m, 30H), 6.71–6.60 (m, 2H), 4.92–4.61 (m, 4H); ^{13}C NMR (100 MHz, $CDCl_3$, *major rotamer*) δ 153.9, 144.3, 140.6, 139.6, 136.3, 133.6, 130.1, 129.7, 129.2, 128.8, 128.7, 128.5, 128.3, 127.9, 127.7, 127.1, 127.0, 118.6, 91.7, 84.8, 52.2. HRMS (MALDI_positive_DIT) m/z: $[M+H]^+$ Calc for $C_{50}H_{37}N_2O_2^+$ 697.2849; Found 697.2849. mp. 63.2-65.6 °C.

***N*,*N'*-(naphthalene-2,6-diyl)bis(3-(biphenyl-2-yl)-*N*-benzylpropiolamide) (3b)**

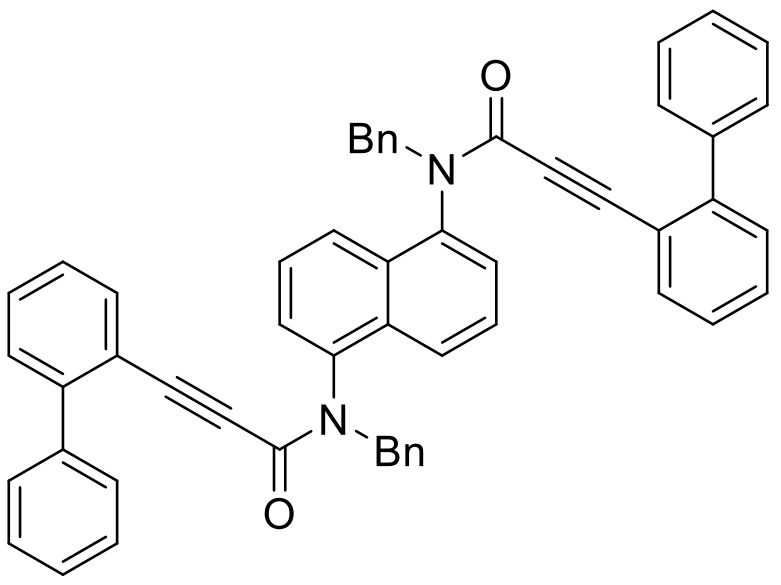

Purified by column chromatography (silica gel), [Hexane/EtOAc = 20:1] to give **3b** as a yellow solid (1.21 g, 54% yield, *rotamer mixture*); ^{1}H NMR (400 MHz, $CDCl_3$, *all rotamers*) δ 7.90–7.09 (m, 24H), 7.09–6.33 (m, 10H), 5.74–5.05 (m, 2H), 4.45–4.08 (m, 2H); ^{13}C NMR (100 MHz, $CDCl_3$, *major rotamer*) δ 154.9, 144.3, 139.4, 137.8, 136.4, 133.6, 131.9, 130.1, 129.9, 129.7, 129.4, 129.2, 128.9, 128.5, 128.2, 127.9, 126.8, 126.6, 123.6, 118.6, 91.0, 85.1, 52.3. HRMS (MALDI_positive_DIT) m/z: $[M+H]^+$ Calc for $C_{54}H_{39}N_2O_2^+$ 747.3006; Found 747.3006. mp. 81.7-83.5 °C.

***N*,*N*'-(dibenzo[*b*,*d*]thiophene-2,8-diyl)bis(3-(biphenyl-2-yl)-*N*-benzylpropiolamide) (3c)**

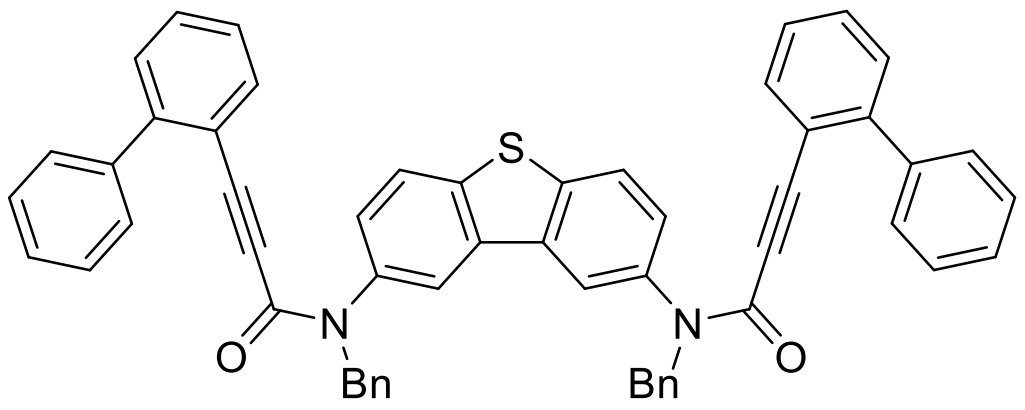

Purified by column chromatography (silica gel), [Hexane/EtOAc = 20:1] to give **3c** as a yellow solid (1.85 g, 77% yield, *rotamer mixture*); ^{1}H NMR (400 MHz, $CDCl_3$, *all rotamers*) δ 7.85–7.35 (m, 8H), 7.35–6.83 (m, 26H), 5.10–4.75 (m, 4H); ^{13}C NMR (100 MHz, $CDCl_3$, *major rotamer*) δ 154.4, 144.5, 139.5, 139.4, 138.4, 136.6, 135.7, 134.1, 130.1, 129.7, 129.1, 128.8, 128.8, 128.7, 128.4, 127.9, 127.5, 127.0, 123.1, 120.9, 118.7, 92.0, 85.1, 52.9. HRMS (MALDI_positive_DIT) m/z: $[M+H]^+$ Calc for $C_{56}H_{39}N_2O_2S^+$ 803.2726; Found 803.2727. mp. 84.4-86.9 °C.

***N*,*N*'-(9-hexyl-9*H*-carbazole-3,6-diyl)bis(3-(biphenyl-2-yl)-*N*-benzylpropiolamide) (3d)**

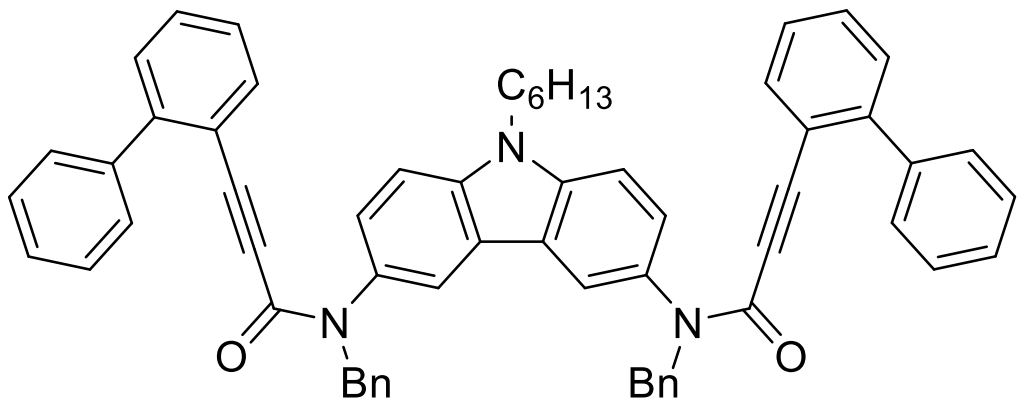

Purified by column chromatography (silica gel), [Hexane/EtOAc = 20:1] to give **3d** as a yellow solid (1.36 g, 52% yield, *rotamer mixture*); ^{1}H NMR (400 MHz, $CDCl_3$, *all rotamers*) δ 7.83–7.35 (m, 5H), 7.31–7.14 (m, 18H), 7.13–6.93 (m, 11H), 5.08–4.79 (m, 4H), 4.22–4.09 (m, 2H), 1.86–1.66 (m, 2H), 1.41–1.17 (m, 6H), 0.91–0.80 (m, 3H); ^{13}C NMR (100 MHz, $CDCl_3$ *major rotamer*) δ 154.9, 144.2, 140.1, 139.4, 137.0, 134.1, 133.2, 129.8, 129.6, 128.9, 128.8, 128.6, 127.9, 127.7, 127.5, 127.1, 126.8, 122.7, 119.9, 119.0, 108.8, 91.7, 85.5, 53.2, 43.5, 31.6, 29.0, 27.0, 22.6, 14.1. HRMS (MALDI_positive_DCTB) m/z: [M] Calc for $C_{62}H_{51}N_3O_2$ 869.3975; Found 869.3976. mp. 73.4-75.3 °C.

***N*,*N*',*N*''-(nitrilotris(benzene-4,1-diyl))tris(3-(biphenyl-2-yl)-*N*-benzylpropiolamide) (5a)**

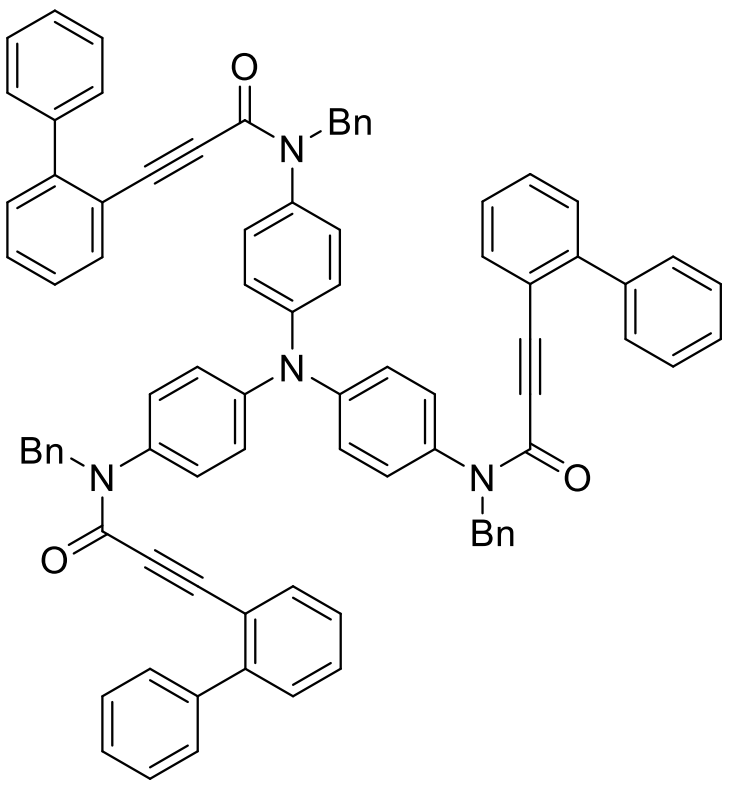

Purified by GPC to give **5a** as a yellow solid (1.30 g, 37% yield, *rotamer mixture*); ^{1}H NMR (400 MHz, $CDCl_3$, *all rotamers*) δ 7.77–6.99 (m, 42H), 6.91–6.48 (m, 12H), 5.02–4.60 (m, 6H); ^{13}C NMR (100 MHz, $CDCl_3$, *major rotamer*) δ 154.3, 146.5, 146.4, 144.3, 139.5, 136.8, 136.5, 133.7, 130.1, 129.8, 129.3, 129.1, 128.7, 128.6, 128.2, 127.9, 127.7, 127.0, 124.4, 124.1, 118.9, 91.5, 85.2, 52.3. HRMS (MALDI_positive_DCTB) m/z: [M] Calc for $C_{84}H_{60}N_4O_3$ 1172.4659; Found 1172.4660. mp. 67.7-71.0 °C.

phenyl 3-(biphenyl-2-yl)propiolate (7a)

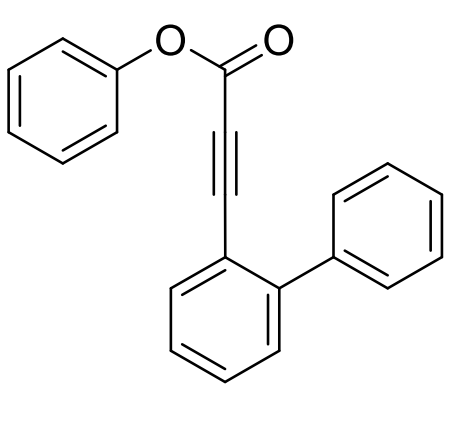

Purified by column chromatography (silica gel), [Hexane/EtOAc = 20:1] to give **7a** as a yellow solid (0.75 g, 84% yield); ^{1}H NMR (400 MHz, $CDCl_3$) δ 7.77–7.70 (m, 1H), 7.65–7.58 (m, 2H), 7.57–7.45 (m, 4H), 7.45–7.35 (m, 4H), 7.28–7.22 (m, 1H), 7.17–7.10 (m, 2H); ^{13}C NMR (100 MHz, $CDCl_3$) δ 152.3, 150.3, 146.0, 139.4, 134.8, 131.3, 130.0, 129.6, 129.3, 128.4, 128.3, 127.4, 126.4, 121.6, 117.8, 88.8, 82.8. HRMS (ESI_positive) m/z: $[M+Na]^+$ Calc for

$C_{21}H_{14}NaO_2^+$ 321.08860; Found 321.08860. mp. 73.3-74.7 °C.

_N_-(biphenyl-2-ylethynyl)-_N_-ethylbenzenesulfonamide (7b)

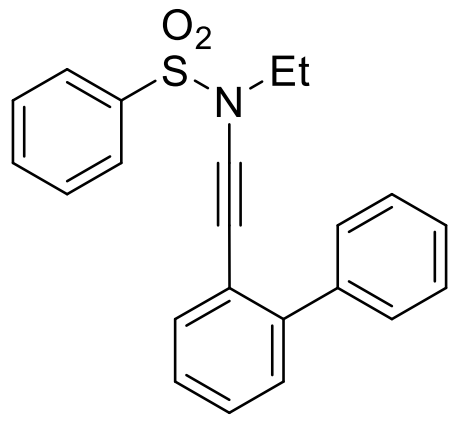

Purified by column chromatography (silica gel), [Hexane/EtOAc = 20:1] to give **7b** as a white solid (0.74 g, 67% yield); ^{1}H NMR (400 MHz, $CDCl_3$) δ 7.70–7.64 (m, 2H), 7.60–7.47 (m, 4H), 7.46–7.38 (m, 4H), 7.38–7.24 (m, 4H), δ 3.35 (q, J = 7.2 Hz, 2H), 1.06 (t, J = 7.2 Hz, 3H); ^{13}C NMR (100 MHz, $CDCl_3$) δ 143.1, 140.8, 137.7, 133.5, 132.3, 129.6, 129.3, 129.2, 128.2, 127.8, 127.5, 127.5, 127.1, 121.5, 84.6, 70.8, 46.9, 13.2. HRMS (APCI_positive) m/z: [M]$^+$ Calc for $C_{22}H_{19}NO_2S^+$ 361.1131; Found 361.1131. mp. 53.5-55.7 °C.

diphenyl (biphenyl-2-ylethynyl)phosphonate (7c)

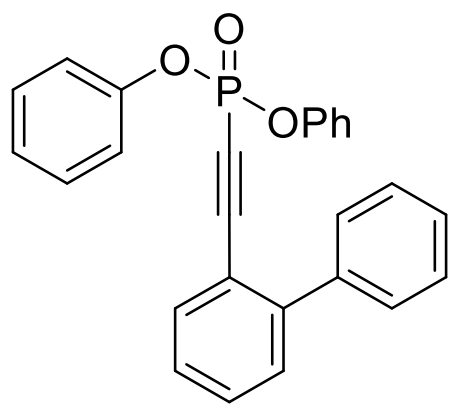

Purified by column chromatography (silica gel), [Hexane/EtOAc = 5:1] to give **7c** as a colorless oil (0.18 g, 22% yield); ^{1}H NMR (400 MHz, $CDCl_3$) δ 7.59–7.52 (m, 1H), 7.52–7.45 (m, 1H), 7.44–7.23 (m, 11H), 7.22–7.14 (m, 2H), 7.14–7.05 (m, 4H); ^{13}C NMR (100 MHz, $CDCl_3$) δ 150.0 (d, J = 7.6 Hz), 145.8, 139.3, 134.6 (d, J = 2.8 Hz), 131.3, 129.9, 129.1, 128.4, 128.2, 127.3, 125.6, 120.9 (d, J = 4.8 Hz), 117.5 (d, J = 6.0 Hz), 102.1 (d, J = 55.2 Hz), 79.7 (d, J = 317.9 Hz), one aromatic sp^2 peak is not shown due to superimposition; ^{31}P NMR (162 MHz, $CDCl_3$)δ 14.2. HRMS (ESI_positive) m/z: [M+Na]$^+$ Calc for $C_{26}H_{19}NaO_3P^+$ 433.0964; Found 433.0964.

1-benzyl-3-(9_H_-fluoren-9-ylidene)indolin-2-one (2a)

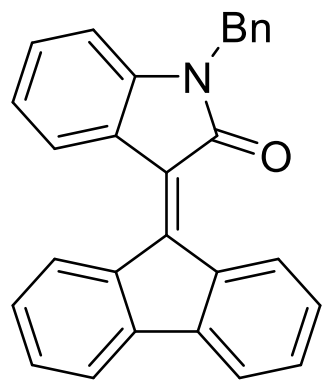

Purified by column chromatography (silica gel), [Hexane/CH_2Cl_2 = 10:1] to give **2a** as a red solid (71.6 mg, 93% yield); ^{1}H NMR (400 MHz, $CDCl_3$) δ 9.14–9.09 (m, 1H), 8.42–8.38 (m, 1H), 8.16 – 8.12 (m, 1H), 7.61–7.56 (m, 2H), 7.41–7.30 (m, 6H), 7.30–7.23 (m, 2H), 7.20–7.11 (m, 2H), 6.96–6.90 (m, 1H), 6.77–6.73 (m, 1H), 5.03 (s, 2H); ^{13}C NMR (100 MHz, $CDCl_3$) δ 167.8, 149.4, 143.3(5), 143.2(8), 141.7, 138.0, 137.6, 136.3, 131.5, 131.4, 130.3, 129.4, 128.9, 128.3, 127.7, 127.6, 127.3, 127.1, 126.7, 125.4, 123.1, 121.5, 120.1, 119.5, 109.3, 43.8. HRMS (FD$^+$ (eiFi)) m/z: [M] Calc for $C_{28}H_{19}NO$ 385.1466; Found 385.1466. mp. 77.7-79.4 °C.

3-(9_H_-fluoren-9-ylidene)-1-methylindolin-2-one (2d)

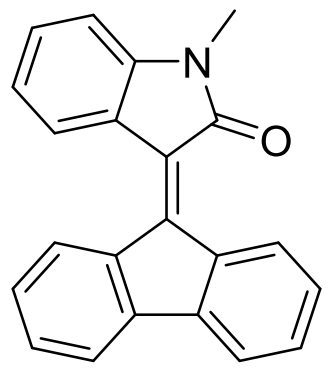

Purified by column chromatography (silica gel), [Hexane/CH_2Cl_2 = 10:1] to give **2d** as a red solid (47.0 mg, 76% yield); ^{1}H NMR (400 MHz, $CDCl_3$) δ 9.11–9.16 (m, 1H), 8.41–8.35 (m, 1H), 8.15–8.10 (m, 1H), 7.61–7.54 (m, 2H), 7.37–7.31 (m, 2H), 7.30–7.25 (m, 1H), 7.24–7.21 (m, 1H), 7.19–7.10 (m, 1H), 6.99–6.92 (m, 1H), 6.85–6.80 (m, 1H), 3.32 (s, 3H); ^{13}C NMR (100 MHz, $CDCl_3$) δ 167.8, 149.0, 144.2, 143.2, 141.6, 138.0, 137.6, 131.4, 131.3, 130.4, 129.3, 128.2, 127.6, 127.1, 126.7, 125.2, 123.0, 121.4, 120.1, 119.4, 108.4, 26.2. HRMS (FD$^+$ (eiFi)) m/z: [M] Calc for $C_{22}H_{15}NO$ 309.1153; Found 309.1152. mp. 149.4-152.7 °C.

3-(9*H*-fluoren-9-ylidene)-1-phenylindolin-2-one (2e)

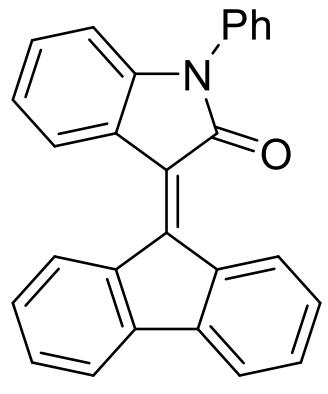

Purified by column chromatography (silica gel), [Hexane/CH_2Cl_2 = 10:1] to give **2e** as a red solid (49.0 mg, 66% yield); ^1H NMR (400 MHz, $CDCl_3$) δ 9.10–9.06 (m, 1H), 8.48–8.44 (m, 1H), 8.22–8.18 (m, 1H), 7.61–7.58 (m, 1H), 7.58–7.54 (m, 3H), 7.52–7.48 (m, 2H), 7.45–7.41 (m, 1H), 7.38–7.32 (m, 2H), 7.23–7.15 (m, 3H), 7.02–6.97 (m, 1H), 6.84–6.81 (m, 1H); ^{13}C NMR (100 MHz, $CDCl_3$) δ 167.1, 149.8, 144.0, 143.3, 141.8, 138.0, 137.6, 134.8, 131.6, 131.5, 130.3, 129.7, 129.6, 128.3, 128.1, 127.1(9), 127.1(5), 127.0(7), 126.7, 125.5, 123.1, 121.9, 120.2, 119.5, 109.8. HRMS (FD^+ (eiFi)) m/z: [M] Calc for $C_{27}H_{17}NO$ 371.1310; Found 371.1309. mp. 143.7-145.3 °C.

1-benzyl-3-(9*H*-fluoren-9-ylidene)-5-methylindolin-2-one (2f)

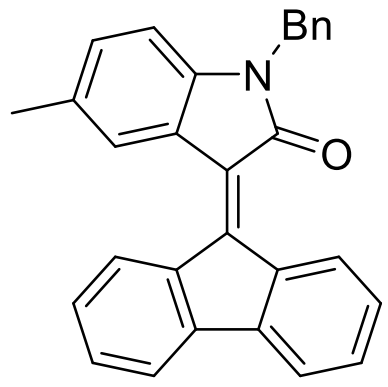

Purified by column chromatography (silica gel), [Hexane/CH_2Cl_2 = 10:1] to give **2f** as a red solid (67.1 mg, 84% yield); ^1H NMR (400 MHz, $CDCl_3$) δ 9.14–9.09 (m, 1H), 8.45–8.41 (m, 1H), 7.97 (s, 1H), 7.65–7.52 (m, 2H), 7.40–7.29 (m, 6H), 7.29–7.23 (m, 2H), 7.18–7.12 (m, 1H), 7.00–6.95 (m, 1H), 6.66–6.61 (m, 1H), 5.01 (s, 2H), 2.26 (s, 3H). ^{13}C NMR (100 MHz, $CDCl_3$) δ 167.8, 149.0, 143.3, 141.7, 141.3, 138.0, 137.7, 136.4, 131.4, 131.3, 130.9, 130.8, 129.4, 128.9, 128.2, 127.8, 127.6, 127.5, 127.0, 126.6, 125.9, 123.1, 120.1, 119.5, 109.0, 43.8, 21.3. HRMS (FD^+ (eiFi)) m/z: [M] Calc for $C_{29}H_{21}NO$ 399.1623; Found 399.1622. mp. 109.5-112.2 °C.

1-benzyl-3-(9*H*-fluoren-9-ylidene)-5-fluoroindolin-2-one (2g)

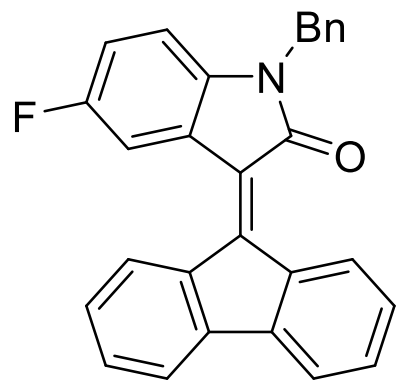

Purified by column chromatography (silica gel), [Hexane/CH_2Cl_2 = 10:1] to give **2g** as a red solid (59.7 mg, 74% yield); ^1H NMR (400 MHz, $CDCl_3$) δ 9.04–8.97 (m, 1H), 8.22–8.15 (m, 1H), 7.74–7.66 (m, 1H), 7.46–7.38 (m, 2H), 7.29–7.09 (m, 8H), 7.06–6.98 (m, 1H), 6.77–6.69 (m, 1H), 6.54–6.46 (m, 1H), 4.88 (s, 2H); ^{13}C NMR (100 MHz, $CDCl_3$) δ 167.4, 158.1 (d, *J* = 238.2 Hz), 150.8, 143.4, 141.8, 139.2, 137.8, 137.0, 136.0, 131.8 (d, *J* = 8.6 Hz), 129.5, 128.9, 128.3, 127.7, 127.4, 127.2, 126.7, 123.9, 123.8, 120.2, 119.5, 116.54 (d, *J* = 24.0 Hz), 112.1 (d, *J* = 25.9 Hz), 109.49 (d, *J* = 8.6 Hz), 43.8. ^{19}F NMR (376 MHz, $CDCl_3$) δ -121.34. HRMS (FD^+ (eiFi)) m/z: [M] Calc for $C_{28}H_{18}FNO$ 403.1372; Found 403.1372. mp. 71.3-72.3 °C.

1-benzyl-3-(9*H*-fluoren-9-ylidene)-5-fluoroindolin-2-one (2h)

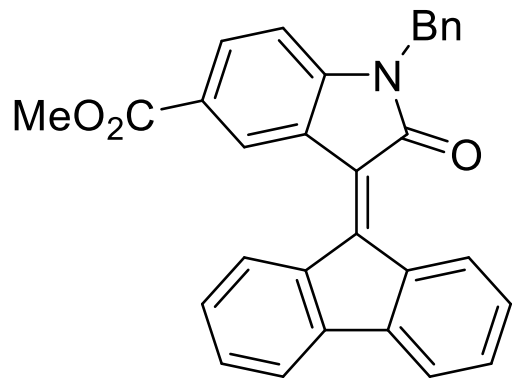

Purified by column chromatography (silica gel), [Hexane/CH_2Cl_2 = 5:1] to give **2g** as a red solid (70.0 mg, 79% yield); ^1H NMR (400 MHz, $CDCl_3$) δ 9.11–9.05 (m, 1H), 8.82 (s, 1H), 8.34–8.28 (m, 1H), 7.91–7.85 (m, 1H), 7.60–7.52 (m, 2H), 7.41–7.30 (m, 6H), 7.30–7.22 (m, 2H), 7.20–7.13 (m, 1H), 6.82–6.75 (m, 1H), 5.04 (s, 2H), 3.84 (s, 3H); ^{13}C NMR (100 MHz, $CDCl_3$) δ 167.8, 166.8, 151.1, 146.5, 143.5, 142.0, 137.8, 137.3, 135.8, 132.0, 131.9, 129.6, 129.0, 128.4, 127.9, 127.5, 127.4, 127.0, 126.6, 125.8, 123.5, 122.8, 120.3, 119.6, 108.7, 52.2, 44.0, one aromatic sp^2 peaks are not shown due to superimposition.-HRMS (ESI_positive) m/z: $[M+Na]^+$ Calc for $C_{30}H_{21}NNaO_3^+$ 466.1413; Found 466.1413. mp. 253.5-254.4 °C.

1-benzyl-3-(9*H*-fluoren-9-ylidene)-5-fluoroindolin-2-one (2i)

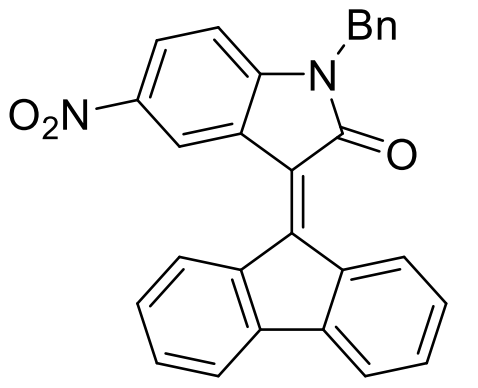

Purified by column chromatography (silica gel), [Hexane/CH_2Cl_2 = 5:1] to give **2i** as a red solid (65.4 mg, 74% yield); ^{1}H NMR (400 MHz, $CDCl_3$) δ 9.09–9.04 (m, 1H), 9.03 (s, 1H), 8.27–8.21 (m, 1H), 8.12–8.07 (m, 1H), 7.60–7.51 (m, 2H), 7.42–7.32 (m, 6H), 7.32–7.14 (m, 3H), 6.85–6.78 (m, 1H), 5.08 (s, 2H); ^{13}C NMR (100 MHz, $CDCl_3$) δ 167.5, 153.4, 147.4, 143.9, 142.6, 142.3, 137.7, 136.9, 135.3, 132.8, 132.6, 129.9, 129.1, 128.6, 128.1, 127.8, 127.5, 127.1, 125.8, 124.2, 123.2, 120.6, 120.4, 119.8, 108.6, 44.2. HRMS (ESI_positive) m/z: $[M+Na]^+$ Calc for $C_{28}H_{18}N_2NaO_3^+$ 453.1209; Found 453.1209. mp. 96.7-98.4 °C.

1-benzyl-3-(9*H*-fluoren-9-ylidene)-1,3-dihydro-2H-benzo[*g*]indol-2-one (2j)

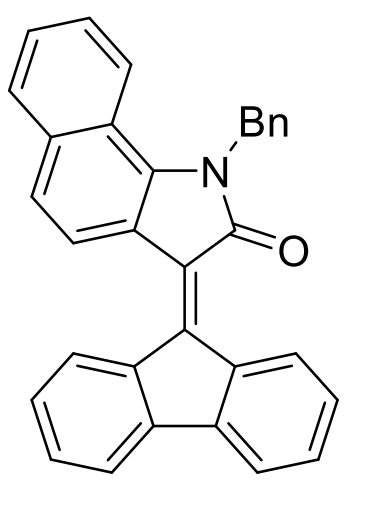

Purified by column chromatography (silica gel), [Hexane/CH_2Cl_2 = 10:1] to give **2j** as a black solid (55.7 mg, 64% yield); ^{1}H NMR (400 MHz, $CDCl_3$) δ 9.17–9.10 (m, 1H), 8.38–8.32 (m, 1H), 8.19–8.12 (m, 1H), 8.04–7.98 (m, 1H), 7.76–7.71 (m, 1H), 7.62–7.56 (m, 2H), 7.41–7.31 (m, 8H), 7.28–7.21 (m, 3H), 7.18–7.12 (m, 1H), 5.57 (s, 2H); ^{13}C NMR (100 MHz, $CDCl_3$) δ 169.3, 148.8, 143.2, 141.8, 140.5, 138.0, 137.6, 137.5, 135.8, 131.5, 131.4, 129.3, 129.1, 129.0(6), 128.3, 127.4, 127.2, 127.1, 126.9, 126.8, 126.2, 126.1, 123.2, 122.7, 121.6, 120.8, 120.2, 119.6, 118.7, 46.3. HRMS (FD^+ (eiFi)) m/z: [M] Calc for $C_{32}H_{21}NO$ 435.1623; Found 435.1622. mp. 229.8-232.1 °C.

1-benzyl-3-(9*H*-fluoren-9-ylidene)-1,3-dihydro-2*H*-dibenzo[*e*,*g*]indol-2-one (2k)

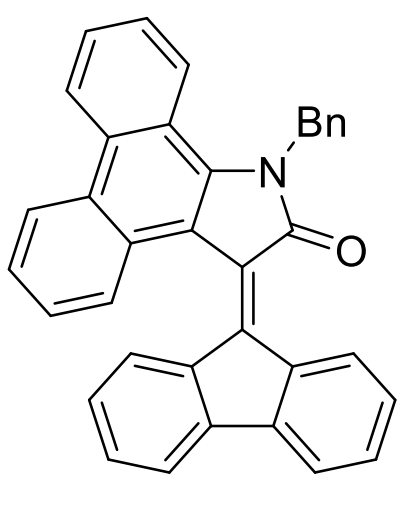

Purified by recrystallization (CH_2Cl_2/hexane) to give **2k** as a black solid (63.1 mg, 65% yield); ^{1}H NMR (700 MHz, $CDCl_3$) δ 9.09–9.06 (m, 1H), 8.73–8.70 (m, 1H), 8.62–8.59 (m, 1H), 8.17–8.14 (m, 1H), 7.75–7.72 (m, 1H), 7.66–7.61 (m, 2H), 7.61–7.58 (m, 1H), 7.47–7.44 (m, 1H), 7.43–7.38 (m, 4H), 7.35–7.30 (m, 4H), 7.26–7.22 (m, 2H), 7.00–6.97 (m, 1H), 6.82–6.78 (m, 1H), 5.64 (d, J = 17.8 Hz, 1H), 5.50 (d, J = 17.0 Hz, 1H); ^{13}C NMR (175 MHz, $CDCl_3$) δ 170.6, 148.1, 142.6, 142.3, 142.3, 138.0, 137.7, 137.5, 133.5, 131.4, 130.8, 130.5, 129.1, 129.0, 128.1, 127.9, 127.4(3), 127.4(0), 127.3, 126.9(4), 126.9(0), 126.8, 126.2, 125.9, 124.5, 124.2, 123.7, 121.3, 119.7, 119.6, 114.4, 46.7, two aromatic sp^2 peaks are not shown due to superimposition. HRMS (APCI_positive) m/z: $[M]^+$ Calc for $C_{36}H_{23}NO^+$ 485.1774; Found 485.1774. mp. 339.3-340.3 °C.

1-benzyl-3-(9*H*-fluoren-9-ylidene)-7-(*p*-tolyl)-1,3-dihydro-2*H*-thieno[2,3-*g*]indol-2-one (2l)

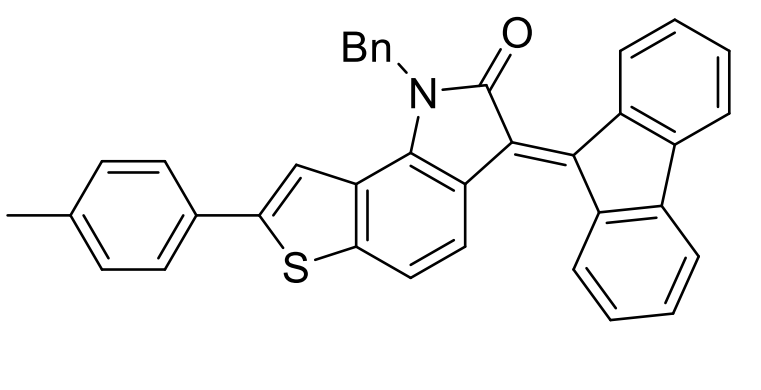

Purified by column chromatography (silica gel), [Hexane/CH_2Cl_2 = 10:1] to give **2l** a as black solid (72.2 mg, 68% yield); ^{1}H NMR (400 MHz, $CDCl_3$) δ 9.13–9.07 (m, 1H), 8.47–8.41 (m, 1H), 8.09–8.03 (m, 1H), 7.62–7.55 (m, 2H), 7.42–7.30 (m, 10H), 7.29–7.21 (m, 2H), 7.20–7.11 (m, 3H), 5.42 (s, 2H), 2.36 (s, 3H); ^{13}C NMR (100 MHz, $CDCl_3$) δ 168.5, 148.3, 144.5, 143.3, 143.1, 141.6, 138.7, 138.5, 138.0, 137.7, 137.2, 131.4, 131.3, 131.0, 129.8, 129.1, 128.2, 127.6, 127.4, 127.1, 126.5, 126.3,

126.2, 125.1, 121.5, 120.1, 119.5, 118.4, 115.7, 115.2, 45.1, 21.3, one aromatic sp[2] peak is not shown due to superimposition. HRMS (MALDI_positive_DIT) m/z: $[M+H]^+$ Calc for $C_{37}H_{26}NOS^+$ 532.1729; Found 532.1729. mp. 293.0-293.7 °C.

1-benzyl-3-(9*H*-fluoren-9-ylidene)-6-methyl-3,6-dihydropyrrolo[3,2-*c*]carbazol-2(1*H*)-one (2m)

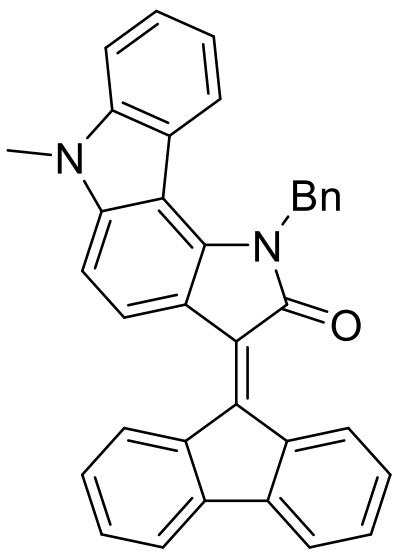

Purified by column chromatography (silica gel), [Hexane/CH_2Cl_2 = 10:1] to give **2m** as a black solid (72.3 mg, 74% yield); ^{1}H NMR (400 MHz, $CDCl_3$) δ 9.11–9.00 (m 1H), 8.57–8.44 (m, 1H), 8.30–8.20 (m, 1H), 7.87–7.76 (m, 1H), 7.64–7.52 (m, 2H), 7.49–7.08 (m, 11H), 7.02–6.87 (m, 2H), 5.61 (s, 2H), 3.78 (s, 3H); ^{13}C NMR (100 MHz, $CDCl_3$) δ 168.5, 148.3, 144.5, 143.3, 143.1, 141.6, 138.7, 138.5, 138.0, 137.7, 137.2, 131.4, 131.3, 131.0, 129.8, 129.1(4), 129.1(1), 128.2, 127.6, 127.4, 127.1, 126.5, 126.3, 126.2, 125.0, 121.5, 120.1, 119.5, 118.4, 115.7, 115.2, 45.1, 21.3. HRMS (MALDI_positive_DIT) m/z: $[M+H]^+$ Calc for $C_{35}H_{25}N_2O^+$ 489.1961; Found 489.1961. mp. 293.0-293.7 °C.

1-benzyl-3-(9*H*-fluoren-9-ylidene)-5-methyl-3,5-dihydropyrrolo[3,2-*b*]carbazol-2(1*H*)-one (2n)

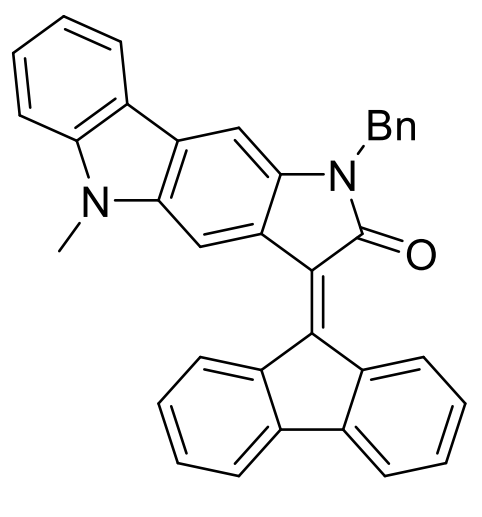

Purified by column chromatography (silica gel), [Hexane/CH_2Cl_2 = 10:1] to give **2n** as a black solid (48.8 mg, 50% yield); ^{1}H NMR (400 MHz, $CDCl_3$) δ 9.18–9.10 (m, 1H), 8.75–8.68 (m, 1H), 8.25 (s, 1H), 7.95–7.88 (m, 1H), 7.65–7.56 (m, 2H), 7.49–7.22 (m, 11H), 7.19–7.10 (m, 2H), 5.11 (s, 2H), 3.72 (s, 3H); ^{13}C NMR (100 MHz, $CDCl_3$) δ 168.0, 147.6, 143.2, 142.2, 141.3, 138.1, 137.8, 137.2, 136.9, 136.5, 131.3, 131.0, 129.2, 129.1, 128.9, 128.2, 127.6, 127.4, 126.8, 126.7, 125.7, 124.5, 122.6, 122.1, 120.6, 120.2, 119.4, 119.0, 108.8, 106.5, 100.0, 44.0, 29.3. HRMS (MALDI_positive_DIT) m/z: $[M+H]^+$ Calc for $C_{35}H_{25}N_2O^+$ 489.1961; Found 489.1961. mp. 285.7-286.4 °C.

1-benzyl-3-(7*H*-dibenzo[*c*,*g*]fluoren-7-ylidene)indolin-2-one (2o)

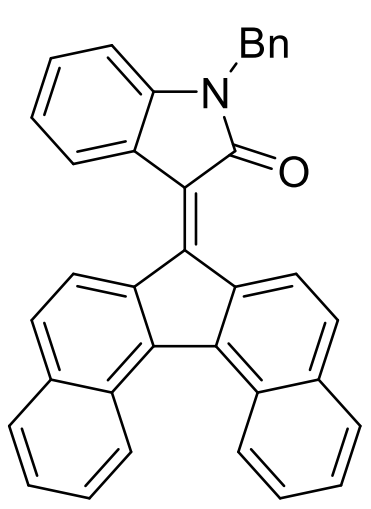

Purified by column chromatography (silica gel), [Hexane/CH_2Cl_2 = 10:1] to give **2o** as a black solid (74.7 mg, 77% yield); ^{1}H NMR (400 MHz, $CDCl_3$) δ 9.15–9.08 (m, 1H), 8.46–8.40 (m, 1H), 8.39–8.31 (m, 2H), 8.19–8.13 (m, 1H), 7.89–7.79 (m, 2H), 7.76–7.70 (m, 1H), 7.65–7.58 (m, 1H), 7.53–7.45 (m, 4H), 7.43–7.38 (m, 2H), 7.37–7.30 (m, 2H), 7.30–7.24 (m, 1H), 7.22–7.15 (m, 1H), 7.00–6.93 (m, 1H), 6.79–6.72 (m, 1H), 5.05 (s, 2H); ^{13}C NMR (100 MHz, $CDCl_3$) δ 167.6, 150.4, 143.6, 143.3, 142.0, 139.7, 137.8, 136.8(1), 136.7(7), 136.3, 130.7, 128.9, 128.8(2), 128.7(8), 128.7, 128.4, 128.3, 128.2, 128.1, 127.9, 127.7, 127.6, 127.5, 127.0, 126.7, 126.5, 125.7, 125.3, 123.3(2), 123.2(7), 121.7, 109.4, 43.9, one aromatic sp[2] peak is not shown due to superimposition. HRMS (APCI_positive) m/z: $[M+H]^+$ Calc for $C_{36}H_{24}NO^+$ 486.1852; Found 486.1852. mp. 185.3-187.8 °C.

1-benzyl-3-(2,6-dimethyl-4*H*-cyclopenta[2,1-*b*:3,4-*b*']dithiophen-4-ylidene)indolin-2-one (2p)

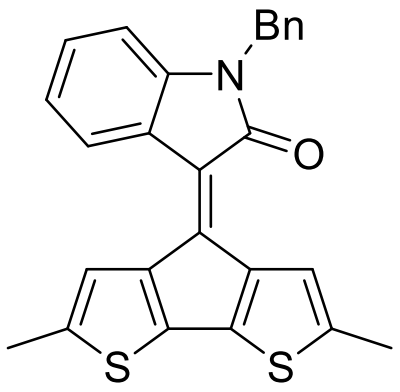

Purified by GPC to give **2p** as a black solid (50.2 mg, 59% yield); ^{1}H NMR (400 MHz, $CDCl_3$) δ 8.02–7.96 (m, 1H), 7.83–7.78 (m, 1H), 7.35–7.27 (m, 4H), 7.27–7.22 (m, 1H), 7.21–7.14 (m, 2H), 7.03–6.96 (m, 1H), 6.73–6.68 (m, 1H), 4.97 (s, 2H), 2.44 (s, 3H), 2.43 (s, 3H); ^{13}C NMR (100 MHz, $CDCl_3$) δ 168.0, 143.5, 143.3, 142.0, 141.5, 139.2, 139.0, 138.2, 136.2, 130.2, 128.9, 127.7, 127.4, 126.4, 125.6, 123.2, 122.8, 122.5, 121.7, 109.1, 43.6, 16.1, 16.0, one aromatic sp^2 peak is not shown due to superimposition. HRMS (APCI_positive) m/z: $[M+H]^+$ Calc for $C_{26}H_{20}NOS_2^+$ 426.0980; Found 426.0981. mp. 225.5-227.4 °C.

A mixture of (*E*)-1-benzyl-3-(2-methyl-9*H*-fluoren-9-ylidene)indolin-2-one ((*E*)-2q) and (*Z*)-1-benzyl-3-(2-methyl-9*H*-fluoren-9-ylidene)indolin-2-one ((*Z*)-2q)

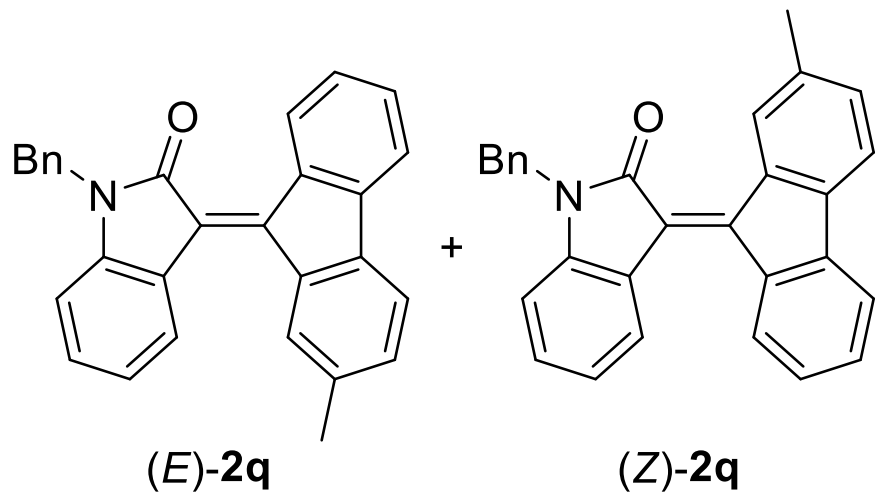

(*E*)-**2q** (*Z*)-**2q**

Purified by column chromatography (silica gel), [Hexane/CH_2Cl_2 = 10:1] to give a mixture of *(E)*-**2q** and *(Z)*-**2q** as a red solid (75.8 mg, 95% yield); ^{1}H NMR (400 MHz, $CDCl_3$) δ 9.14–9.10 (m, 0.52H), 8.99 (s, 0.42H), 8.38–8.34 (m, 0.47H), 8.22 (s, 0.55H), 8.17–8.13 (m, 0.55H), 8.13–8.10 (m, 0.45H), 7.64–7.06 (m, 12H), 6.96–6.89 (m, 0.99H), 6.77–6.74 (m,0.99H), 5.04 (s, 0.93H), 5.03 (s, 1.07H), 2.43 (s, 1.36H), 2.31 (s, 1.58H); ^{13}C NMR (100 MHz, $CDCl_3$) δ 167.8, 149.9, 149.8, 143.5, 143.3, 143.2, 142.0, 140.8, 139.3, 138.3, 138.1, 138.0, 137.8, 137.6, 136.9, 136.4, 136.3(9), 132.3(5), 132.2(6), 131.5, 131.4, 130.2, 130.1, 130.0, 129.4, 128.9(2), 128.9(1), 127.8, 127.7, 127.6, 127.6, 127.5, 127.3, 127.1, 127.0, 126.8, 126.6, 125.4, 125.2, 123.2, 123.1, 121.4, 121.3, 119.9, 119.8, 119.3, 119.2, 109.3, 109.2, 43.7(8), 43.7(6), 22.0, 21.7. HRMS (APCI_positive) m/z: $[M]^+$ Calc for $C_{29}H_{21}NO^+$ 399.1617; Found 399.1618.

A mixture of 1-benzyl-3-(9*H*-fluoren-9-ylidene-4-*d*)indolin-2-one (2a-*d*$_1$) and 1-benzyl-3-(9*H*-fluoren-9-ylidene)indolin-2-one (2a)

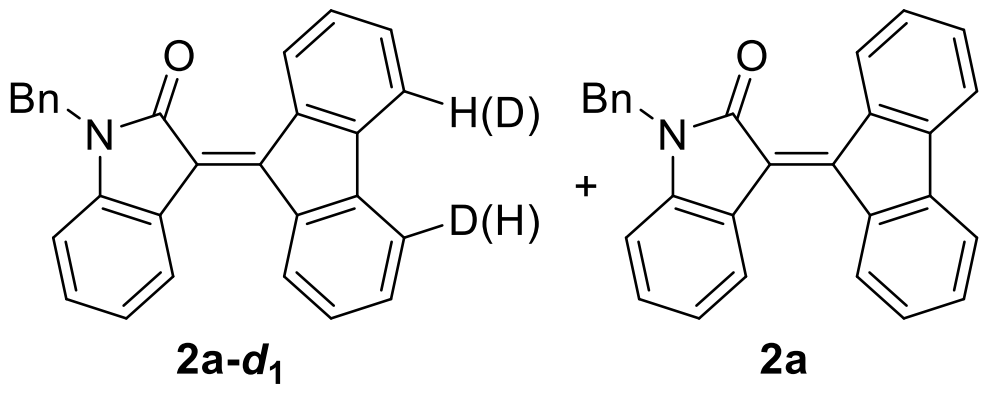

2a-*d*$_1$ **2a**

Purified by column chromatography (silica gel), [Hexane/CH_2Cl_2 = 10:1] to give a mixture of **2a**-*d*$_1$ (45% NMR yield) and **2a** (12% NMR yield) as a red solid; ^{1}H NMR (400 MHz, $CDCl_3$) δ 9.15–9.10 (m, 1H), 8.42–8.37 (m, 1H), 8.16–8.11 (m, 1H), 7.62–7.55 (m, 1.24H), 7.41–7.29 (m, 6H), 7.29–7.23 (m, 2H), 7.19–7.11 (m, 2H), 6.96–6.89 (m, 1H), 6.77–6.73 (m, 1H), 5.03 (s, 2H); ^{13}C NMR (100 MHz, $CDCl_3$) δ 167.8, 149.4, 143.4, 143.3, 141.7, 138.0, 137.6, 136.3, 131.5, 131.4, 131.4, 131.3, 130.3, 129.4, 128.9, 128.3, 127.7, 127.6, 127.3, 127.1, 126.7, 125.4, 123.1, 121.5, 120.1, 119.5, 109.3, 43.8. HRMS (APCI_positive) m/z: $[M]^+$ Calc for $C_{28}H_{18}DNO^+$ 386.1523; Found 386.1523.

A mixture of 1-benzyl-3-(9*H*-fluoren-9-ylidene)indolin-2-one-7-*d* (2a'-*d*$_1$) and 1-benzyl-3-(9*H*-fluoren-9-ylidene)indolin-2-one (2a)

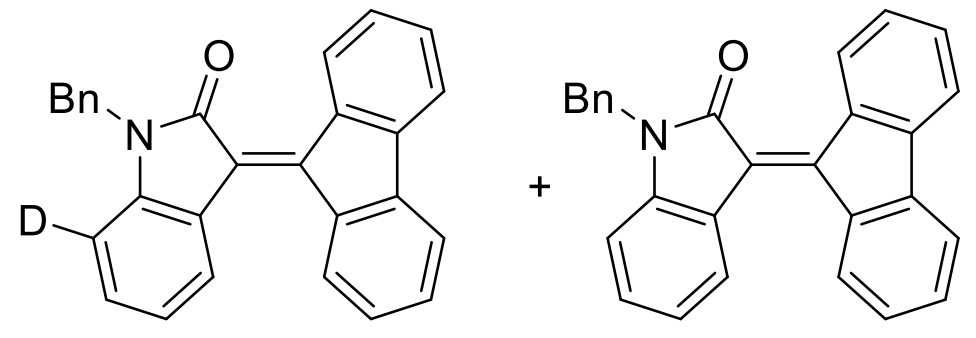

2a'-d_1 2a

Purified by column chromatography (silica gel), [Hexane/CH_2Cl_2 = 10:1] to give a mixture of **2a'**-d_1 (38% NMR yield) and **2a** (12% NMR yield) as a red solid; ^{1}H NMR (400 MHz, $CDCl_3$) δ 9.15–9.09 (m, 1H), 8.42–8.37 (m, 1H), 8.16–8.11 (m, 1H), 7.62–7.55 (m, 2H), 7.41–7.29 (m, 6H), 7.29–7.23 (m, 2H), 7.19–7.11 (m, 2H), 6.96–6.89 (m, 1H), 6.77–6.73 (m, 0.21H), 5.03 (s, 2H); ^{13}C NMR (100 MHz, $CDCl_3$) δ 167.8, 149.4, 143.4, 143.3, 141.7, 138.0, 137.6, 136.3, 131.5, 131.4, 131.4, 131.3, 130.3, 129.4, 128.9, 128.3, 127.7, 127.6, 127.3, 127.1, 126.7, 125.4, 123.1, 121.5, 120.1, 119.5, 109.3, 43.8. HRMS (APCI_positive) m/z: $[M]^+$ Calc for $C_{28}H_{18}DNO^+$ 386.1523; Found 386.1523.

1-benzyl-3-(9*H*-fluoren-9-ylidene-1,2,3,4-*d*$_4$)indolin-2-one (2a-*d*$_4$)

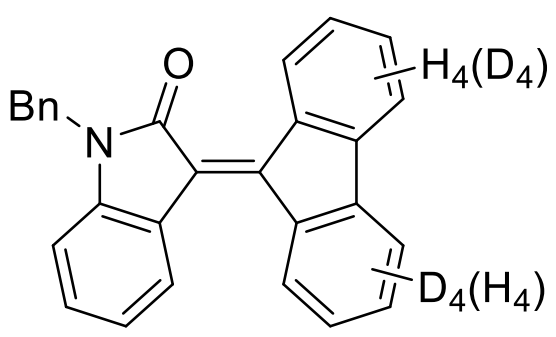

Purified by column chromatography (silica gel), [Hexane/CH_2Cl_2 = 10:1] to give a mixture of **2a**-d_4 as a red solid (71.8 mg, 92% yield); ^{1}H NMR (400 MHz, $CDCl_3$) δ 9.18–9.11 (m, 0.5H), 8.42–8.36 (m, 0.5H), 8.17–8.09 (m, 1H), 7.61–7.54 (m, 2H), 7.43–7.23 (m, 5H), 7.20–7.10 (m, 1.5H), 6.96–6.88 (m, 1.5H), 6.78–6.72 (m, 1H), 5.03 (s, 2H); ^{13}C NMR (100 MHz, $CDCl_3$) δ 167.8, 149.4, 143.3, 143.3, 143.2, 141.8, 141.7, 138.0, 137.9, 137.6, 137.5, 136.4, 131.5, 131.4, 130.3, 129.4, 128.9, 128.3, 127.7, 127.6, 127.3, 127.1, 126.7, 125.4, 125.3, 123.1, 121.5, 120.2, 119.5, 109.3, 43.8, sp^2 carbons adjacent to D cannot be identified due to their weak intensity and superimposition. HRMS (ESI_positive) m/z: $[M+Na]^+$ Calc for $C_{28}H_{15}D_4NNaO^+$ 412.1609; Found 412.1609. mp. 75.4-77.4 °C.

1-benzyl-3-(9*H*-fluoren-9-ylidene)indolin-2-one-4,5,6,7-*d*$_4$ (2a'-*d*$_4$)

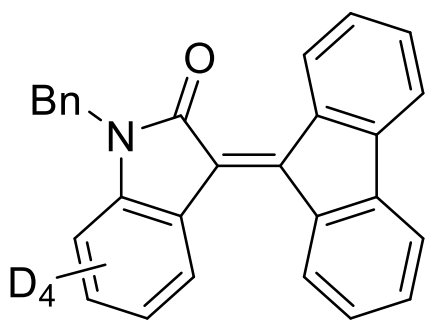

Purified by column chromatography (silica gel), [Hexane/CH_2Cl_2 = 10:1] to give a mixture of **2a'**-d_4 as a red solid (70.2 mg, 90% yield); ^{1}H NMR (400 MHz, $CDCl_3$) δ 9.17–9.12 (m, 1H), 8.42–8.37 (m, 1H), 7.61–7.54 (m, 2H), 7.42–7.30 (m, 6H), 7.30–7.23 (m, 2H), 7.16–7.11 (m, 1H), 5.03 (s, 2H); ^{13}C NMR (100 MHz, $CDCl_3$) δ 167.8, 149.4, 143.3, 141.7, 138.0, 137.6, 136.4, 131.5, 131.4, 129.8 (t, J = 24.4 Hz), 129.4, 128.9, 128.3, 127.7, 127.6, 127.3, 127.1, 126.7, 125.0 (t, J = 23.5 Hz), 123.0, 121.0, 120.1 (t, J = 23.9 Hz), 119.5, 108.9 (t, J = 24.3 Hz), 43.8, one aromatic sp^2 peak is not shown due to superimposition. HRMS (ESI_positive) m/z: $[M+Na]^+$ Calc for $C_{28}H_{15}D_4NNaO^+$ 412.1609; Found 412.1609. mp. 73.3-74.9 °C.

1,5-dibenzyl-3,7-di(9*H*-fluoren-9-ylidene)-5,7-dihydropyrrolo[2,3-*f*]indole-2,6(1*H*,3*H*)-dione (4a)

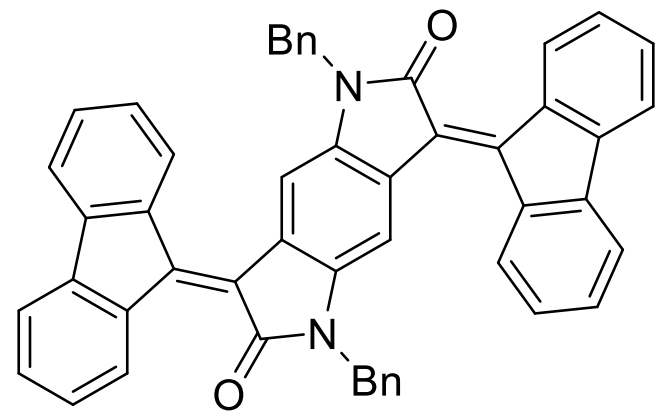

Purified by recrystallization (CH_2Cl_2/hexane) to give **4a** as a black solid (51.2 mg, 37% yield); ^{1}H NMR (700 MHz, $CDCl_3$) δ 9.07–9.03 (m, 2H), 8.08–8.04 (m, 2H), 7.58–7.54 (m, 4H), 7.49 (s, 2H), 7.36–7.28 (m, 14H), 7.25–7.22 (m, 2H), 6.81–6.77 (m, 2H), 4.93 (s, 4H); ^{13}C NMR (175 MHz, $CDCl_3$) δ 167.4, 149.9, 143.4, 141.9, 138.7, 137.9, 137.4, 136.2, 131.7, 131.5, 129.7, 129.0, 128.3, 127.7,

127.4, 127.3(4), 127.2(9), 126.5, 125.0, 120.2, 119.5, 106.0, 43.7. HRMS (MALDI_positive_DIT) m/z: $[M+H]^+$ Calc for $C_{50}H_{33}N_2O_2^+$ 693.2536; Found 693.2536. mp. 339.3-340.9 °C.

3,8-dibenzyl-1,6-di(9*H*-fluoren-9-ylidene)-1,3,6,8-tetrahydroindolo[7,6-*g*]indole-2,7-dione (4b)

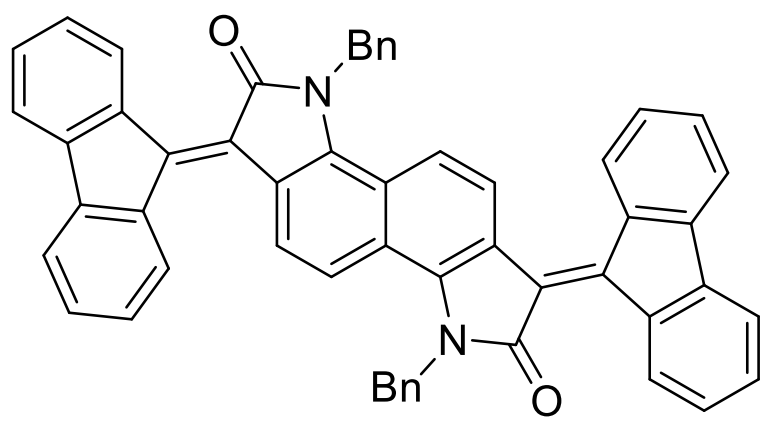

Purified by recrystallization (CH_2Cl_2/hexane) to give **4b** as a deep green solid (44.5 mg, 30% yield); ^1H NMR (400 MHz, $CDCl_3$) δ 9.08–9.03 (m, 2H), 8.23–8.18 (m, 2H), 7.93–7.88 (m, 2H), 7.65–7.60 (m, 2H), 7.60–7.54 (m, 4H), 7.39–7.30 (m, 12H), 7.24–7.20 (m, 4H), 7.18–7.11 (m, 2H), 5.57 (s, 4H); ^{13}C NMR (100 MHz, $CDCl_3$) δ 169.2, 149.6, 143.3, 141.9, 141.3, 137.9, 137.5, 137.3, 131.8, 131.7, 129.3, 129.1, 128.4, 127.5, 127.2, 126.9, 126.3, 126.2, 122.5, 122.1, 120.3, 120.1, 119.7, 116.7, 46.7. HRMS (MALDI_positive_DIT) m/z: $[M+H]^+$ Calc for $C_{54}H_{35}N_2O_2^+$ 743.2693; Found 743.2692. mp. 341.7-343.5 °C.

1,9-dibenzyl-3,7-di(9*H*-fluoren-9-ylidene)-1,3,7,9-tetrahydrothieno[2,3-*f*:5,4-*f'*]diindole-2,8-dione (4c)

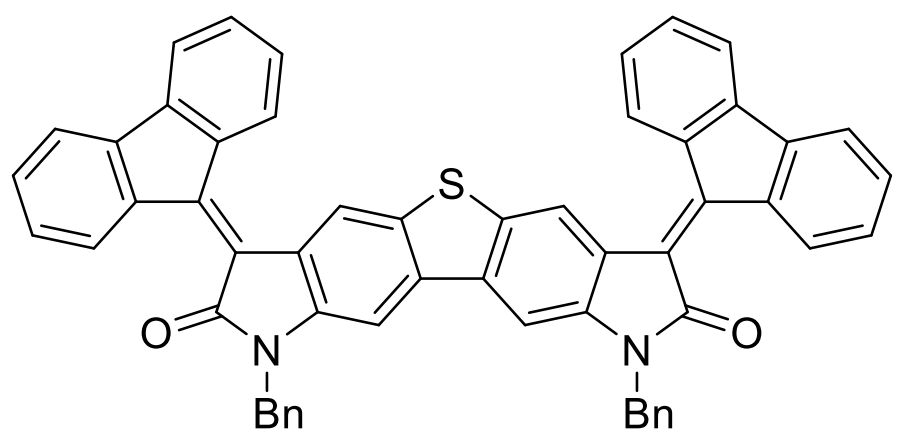

Purified by column chromatography (silica gel), [Hexane/CH_2Cl_2 = 10:1] to give **4c** as a black solid (130.9 mg, 82% yield); ^1H NMR (400 MHz, $CDCl_3$) δ 9.13–9.05 (m, 2H), 8.43 (s, 2H), 8.41–8.35 (m, 2H), 7.60–7.51 (m, 4H), 7.45–7.28 (m, 14H), 7.28–7.22 (m, 2H), 7.18–7.11 (m, 2H), 7.09 (s, 2H), 5.09 (s, 4H); ^{13}C NMR (100 MHz, $CDCl_3$) δ 167.6, 149.8, 143.4, 141.7, 140.9, 138.0, 137.5, 136.2, 136.1, 134.2, 131.8, 131.7, 129.6, 129.0, 128.3, 127.9, 127.5, 127.4, 126.7, 126.3, 124.1, 120.4, 119.6, 119.5, 101.1, 44.0. HRMS (MALDI_positive_DIT) m/z: $[M+H]^+$ Calc for $C_{56}H_{35}N_2O_2S^+$ 799.2413; Found 799.2414. mp. 294.4-297.7 °C.

1,9-dibenzyl-3,7-di(9*H*-fluoren-9-ylidene)-5-hexyl-1,5,7,9-tetrahydro-2*H*-dipyrrolo[3,2-*b*:2',3'-*h*]carbazole-2,8(3*H*)-dione (4d)

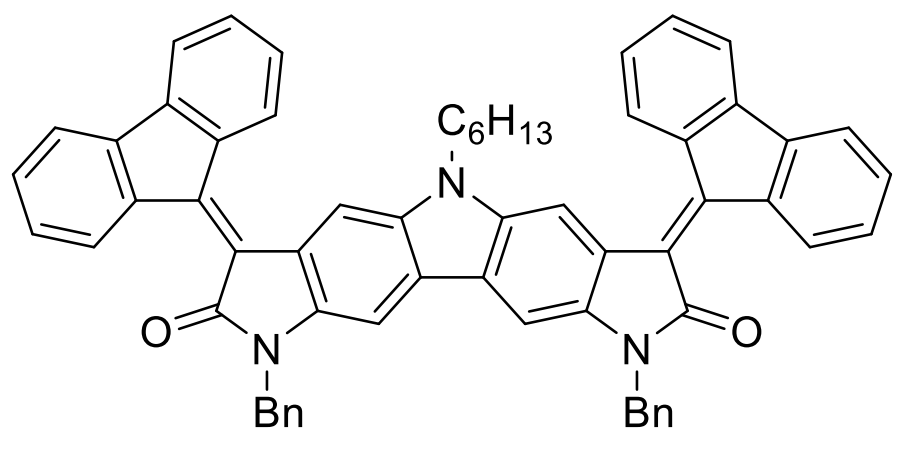

Purified by column chromatography (silica gel), [Hexane/CH_2Cl_2 = 10:1] to give **4d** as black solid (81.3 mg, 47% yield); ^1H NMR (400 MHz, $CDCl_3$) δ 9.17–9.05 (m, 2H), 8.66–8.54 (m, 2H), 8.11 (s, 2H), 7.63–7.50 (m, 4H), 7.50–7.41 (m, 2H), 7.41–7.19 (m, 12H), 7.12–7.01 (m, 4H), 5.08 (s, 4H), 3.96 (t, J = 7.2 Hz, 2H), 1.82–1.67 (m, 2H), 1.37–1.18 (m, 6H), 0.92–0.76 (m, 3H); ^{13}C NMR (100 MHz, $CDCl_3$) δ 167.8, 147.8, 143.3, 141.3, 138.0, 137.7, 137.5, 137.0, 136.5, 131.4, 131.1, 129.4, 128.9, 128.8, 128.2, 127.6, 127.4, 126.7, 125.7, 124.0, 123.1, 120.3, 119.5, 106.9, 99.5, 43.9, 43.2, 31.9, 29.8, 27.0, 22.7, 14.2. HRMS (MALDI_positive_DCTB) m/z: $[M]^+$ Calc for $C_{62}H_{47}N_3O_2^+$ 865.3662; Found 865.3663. mp. 313.8-315.9 °C.

5,5',5''-methanetriyltris(1-benzyl-3-(9*H*-fluoren-9-ylidene)indolin-2-one) (6a)

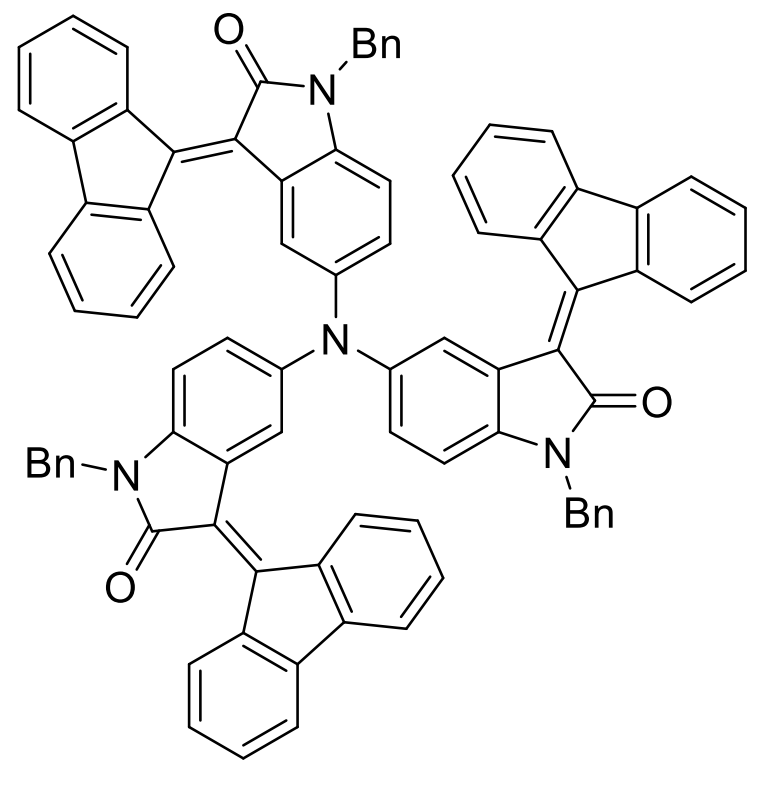

Purified by GPC to give **6a** as a deep green solid (112.0 mg, 48% yield); ^{1}H NMR (400 MHz, $CDCl_3$) δ 9.14–9.06 (m, 3H), 7.86–7.80 (m, 3H), 7.77–7.72 (m, 3H), 7.49–7.44 (m, 3H), 7.43–7.37 (m, 9H), 7.36–7.27 (m, 12H), 7.25–7.20 (m, 3H), 6.99–6.88 (m, 6H), 6.70–6.63 (m, 3H), 6.38–6.30 (m, 3H), 5.00 (s, 6H); ^{13}C NMR (100 MHz, $CDCl_3$) δ 167.6, 150.0, 143.2, 142.9, 141.7, 138.8, 137.8, 137.1, 136.4, 131.6, 131.5, 129.5, 129.0, 128.3, 127.8, 127.7, 126.9, 126.6, 126.4, 125.9, 124.1, 122.1, 120.0, 119.5, 109.7, 44.0. HRMS (MALDI_positive_DCTB) m/z: $[M]^+$ Calc for $C_{84}H_{54}N_4O_3^+$ 1166.4190; Found 1166.4191. mp. 301.5-303.6 °C.

3-(9*H*-fluoren-9-ylidene)benzofuran-2(3*H*)-one (8a)

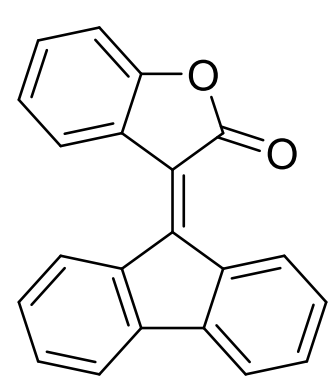

Purified by column chromatography (silica gel), [Hexane/CH_2Cl_2 = 10:1] to give **8a** as orange solid (29.6 mg, 50% yield); ^{1}H NMR (400 MHz, $CDCl_3$) δ 8.92–8.80 (m, 1H), 8.40–8.29 (m, 1H), 8.18–8.07 (m, 1H), 7.62–7.50 (m, 2H), 7.42–7.30 (m, 3H), 7.29–7.20 (m, 1H), 7.20–7.04 (m, 3H); ^{13}C NMR (100 MHz, $CDCl_3$) δ 167.2, 154.4, 152.0, 143.2, 142.0, 137.5, 137.0, 132.4, 132.1, 131.3, 129.3, 128.6, 127.5, 126.2, 124.6, 124.5, 123.3, 121.0, 120.4, 119.8, 111.3. HRMS (APCI_positive) m/z: $[M+H]^+$ Calc for $C_{21}H_{13}O_2^+$ 297.0910; Found 297.0910. Mp. 148.9-151.3 °C.

2-ethyl-3-(9*H*-fluoren-9-ylidene)-2,3-dihydrobenzo[*d*]isothiazole 1,1-dioxide (8b)

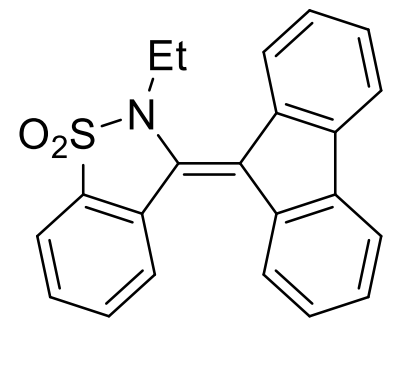

Purified by column chromatography (silica gel), [Hexane/ EtOAc = 20:1] to give **8b** as a orange solid (63.2 mg, 88% yield); ^{1}H NMR (400 MHz, $CDCl_3$) δ 8.65–8.54 (m, 1H), 8.18–8.10 (m, 1H), 7.93–7.85 (m, 1H), 7.82–7.69 (m, 3H), 7.63–7.54 (m, 2H), 7.40–7.30 (m, 3H), 7.28–7.20 (m, 1H), 3.99 (q, *J* = 7.0 Hz, 2H), 1.28 (t, *J* = 7.0 Hz, 3H); ^{13}C NMR (100 MHz, $CDCl_3$) δ 140.4, 139.3, 137.1, 136.9, 136.8, 133.2, 132.7, 131.5, 131.3, 128.1, 127.8, 127.5, 126.8, 125.4, 123.9, 122.0, 120.4, 120.1, 44.3, 13.0. HRMS (APCI_positive) m/z: $[M]^+$ Calc for $C_{22}H_{17}NO_2S^+$ 359.0974; Found 359.0974. Mp. 178.0-179.3 °C.

3-(9*H*-fluoren-9-ylidene)-2-phenoxy-3H-benzo[*d*][1,2]oxaphosphole 2-oxide (8c)

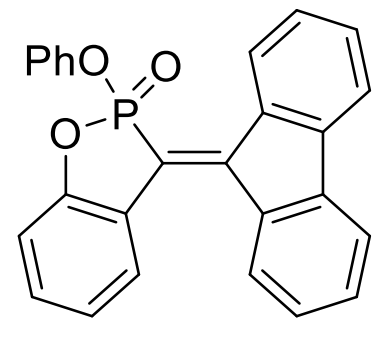

Purified by column chromatography (silica gel), [Hexane/ EtOAc = 5:1] to give **8C** as a yellow solid (79.2 mg, 97% yield); ^{1}H NMR (400 MHz, $CDCl_3$) δ 8.70–8.60 (m, 1H), 8.30–8.24 (m, 1H), 8.05–7.99 (m, 1H), 7.64–7.57 (m, 2H), 7.43–7.26 (m, 4H), 7.22–7.15 (m, 2H), 7.14–7.01 (m, 6H); ^{13}C NMR (100 MHz, $CDCl_3$) δ 153.6 (d, *J* = 7.9 Hz), 150.3 (d, *J* = 9.8 Hz), 147.6 (d, *J* = 9.0 Hz), 142.7, 141.3, 137.5 (d, *J* = 8.3 Hz), 136.0 (d, *J* = 22.1 Hz), 132.0 (d, *J* = 2.4 Hz), 131.4 (d, *J* = 65.3 Hz), 129.8, 128.3, 127.5, 127.4 (d, *J* = 16.2 Hz), 126.4, 125.7, 125.4 (d, *J* = 23.0 Hz), 124.8, 123.3, 120.7 (d, *J* = 4.3 Hz), 120.3, 120.0, 119.7, 118.0, 114.4 (d, *J* = 8.6 Hz); ^{31}P NMR

(162 MHz, $CDCl_3$) δ 29.1. HRMS (ESI_positive) m/z: $[M+Na]^+$ Calc for $C_{26}H_{17}NaO_3P^+$ 431.0807; Found 431.0807. Mp. 132.7-133.9 °C.

11. ^{1}H and ^{13}C NMR spectra of starting substrates and products

^{1}H NMR (400 MHz, $CDCl_3$)

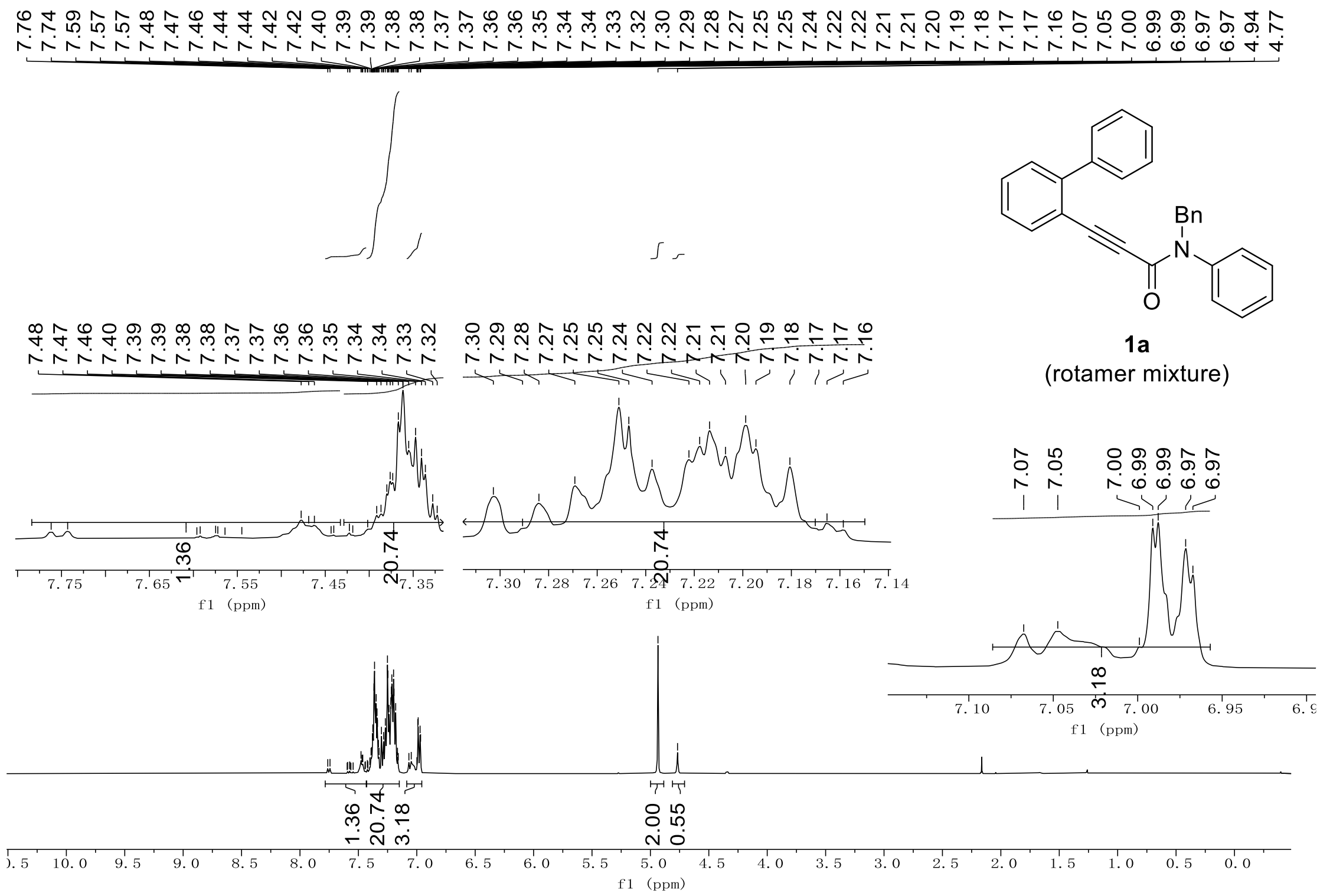

1a
(rotamer mixture)

^{13}C NMR (100 MHz, $CDCl_3$) (Only signals corresponding to the major rotamer are reported)

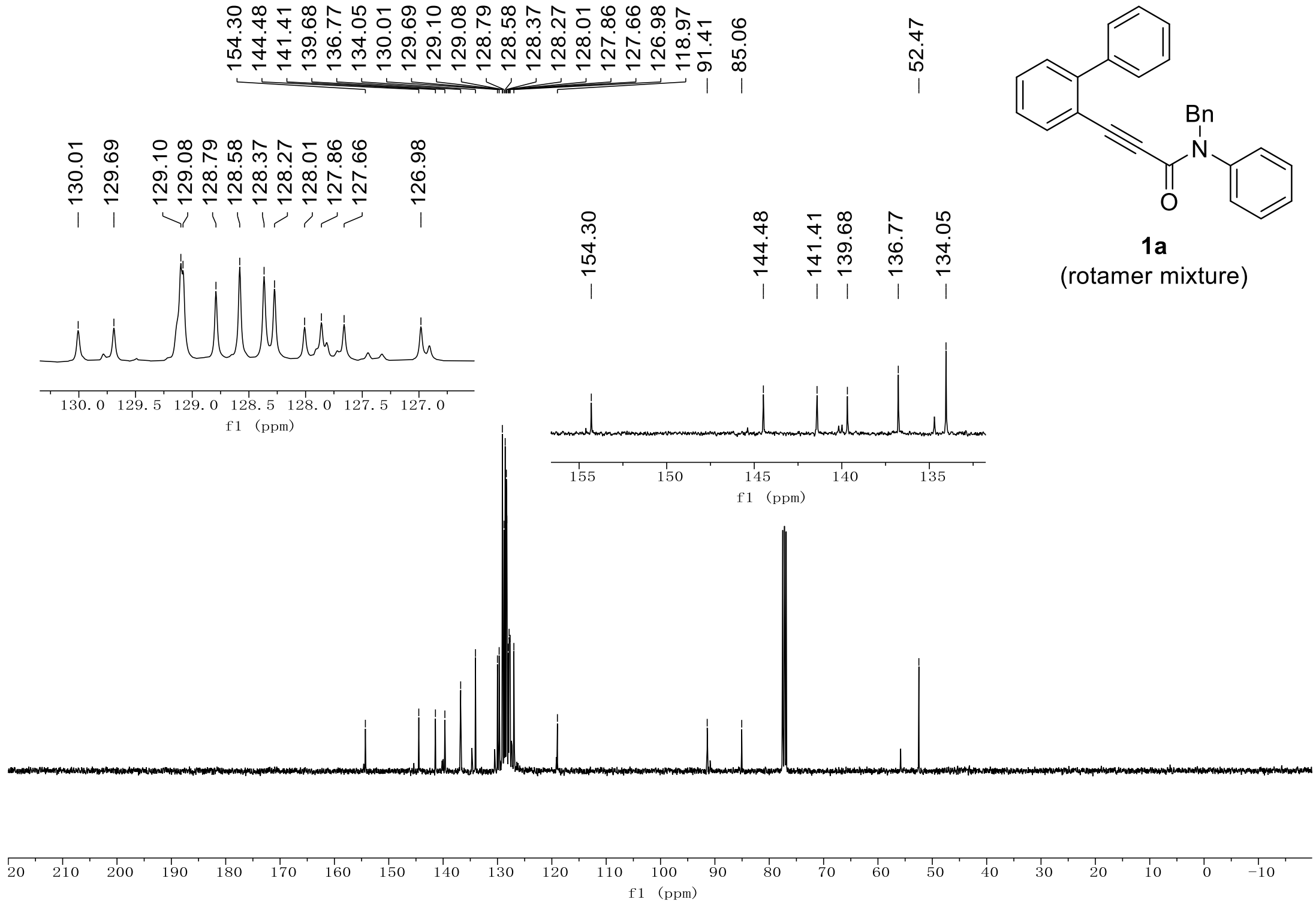

1a
(rotamer mixture)

^{1}H NMR (400 MHz, $CDCl_3$)

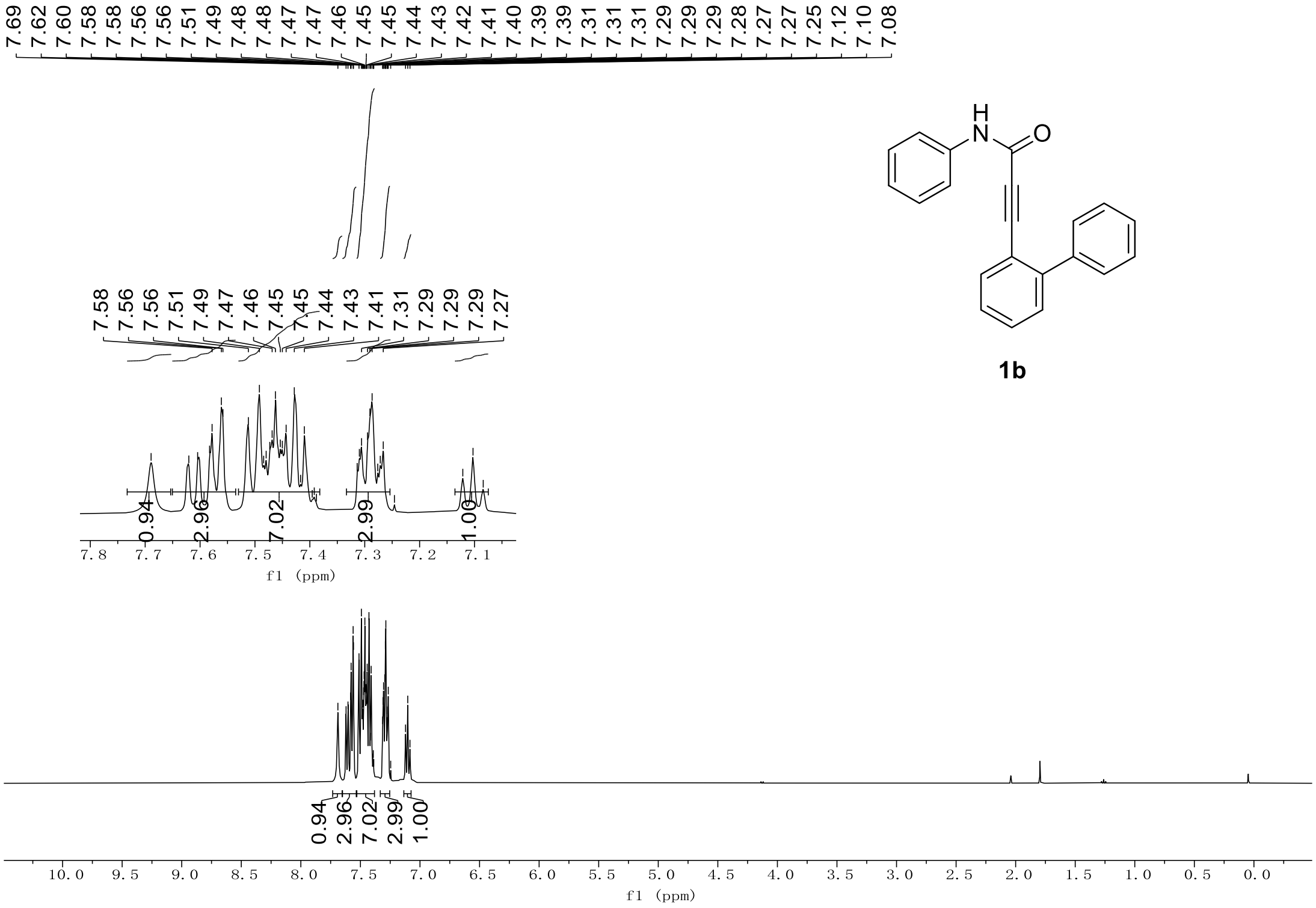

1b

^{13}C NMR (100 MHz, $CDCl_3$)

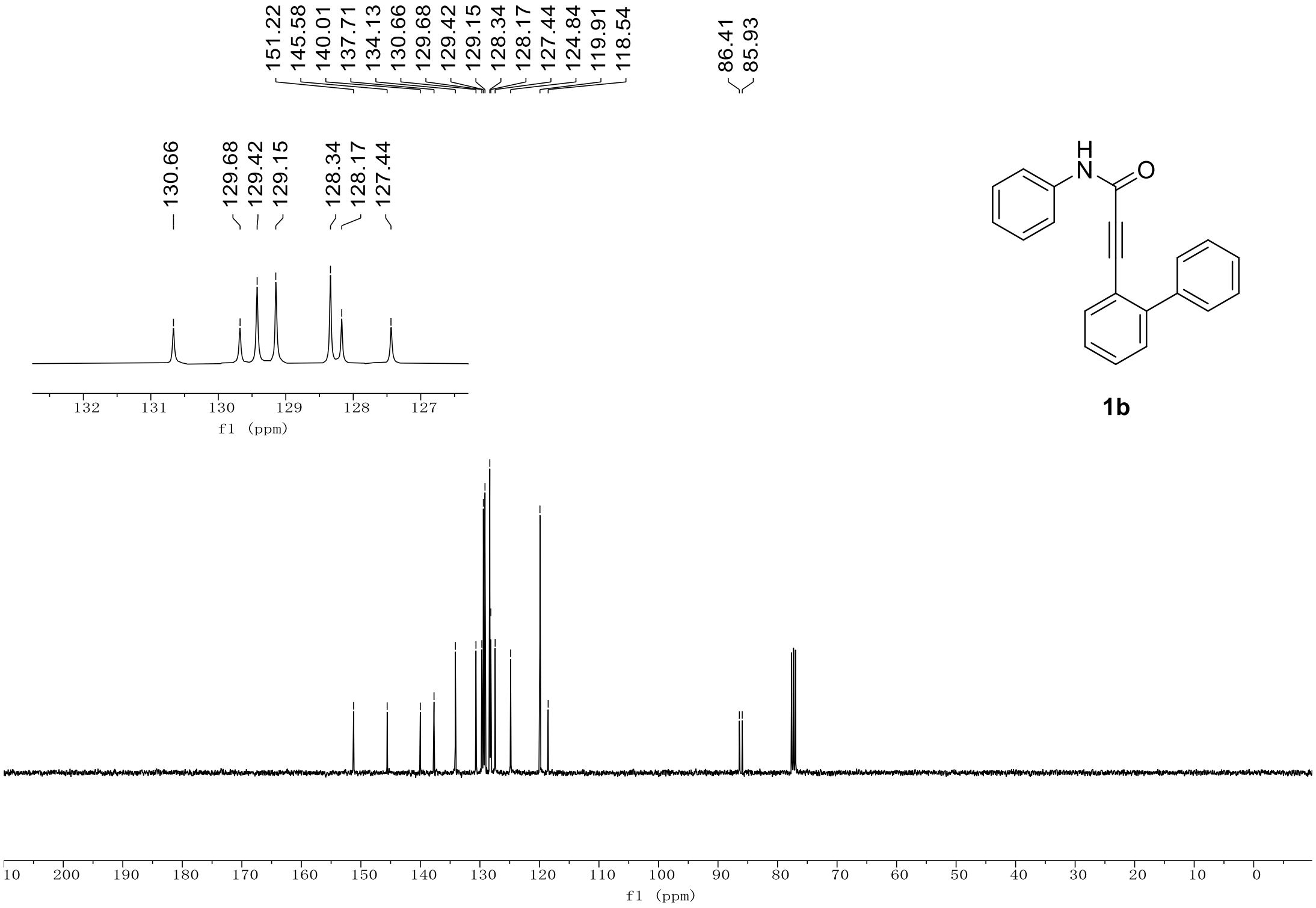

1b

^1H NMR (400 MHz, $CDCl_3$)

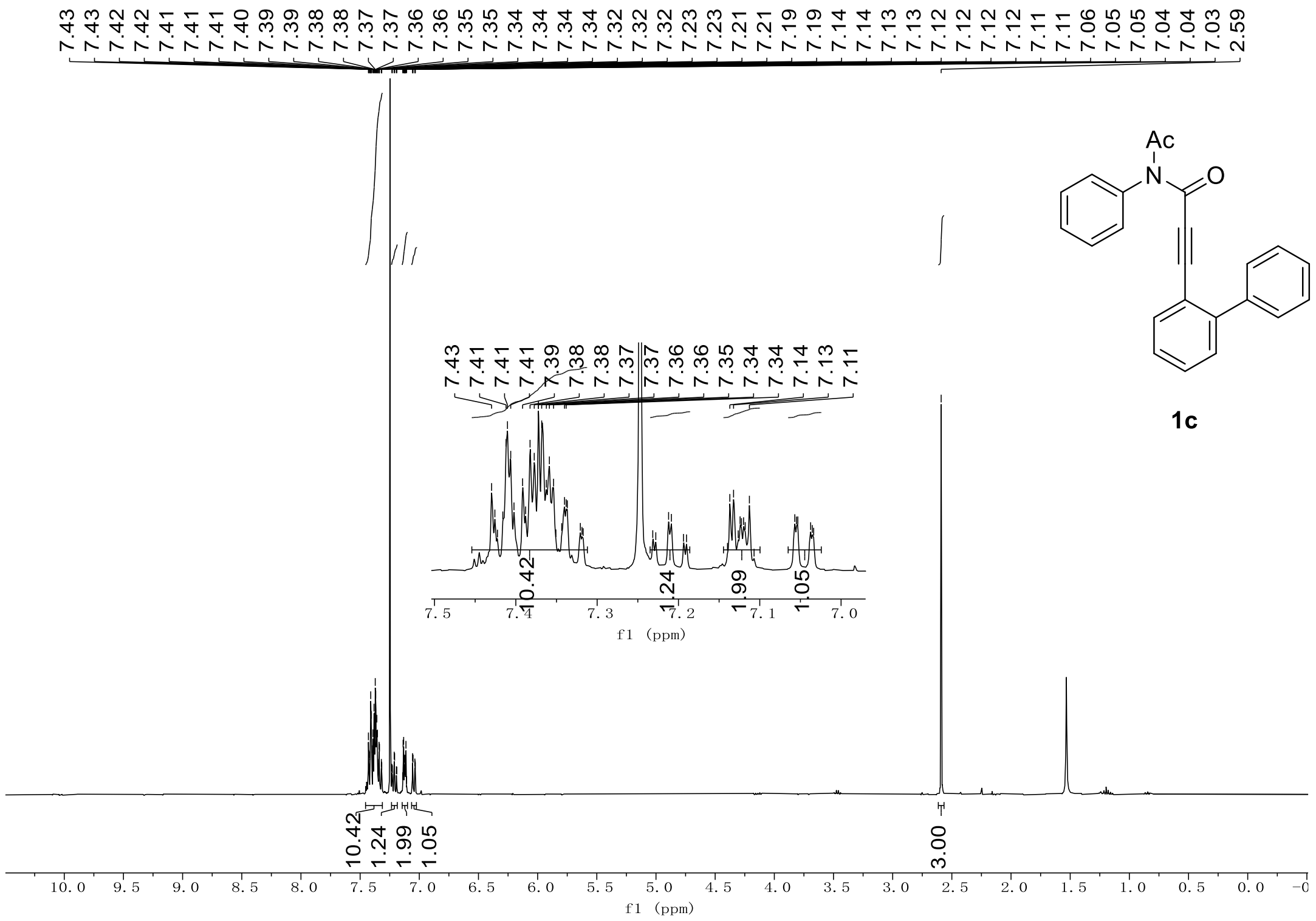

^{13}C NMR (100 MHz, $CDCl_3$)

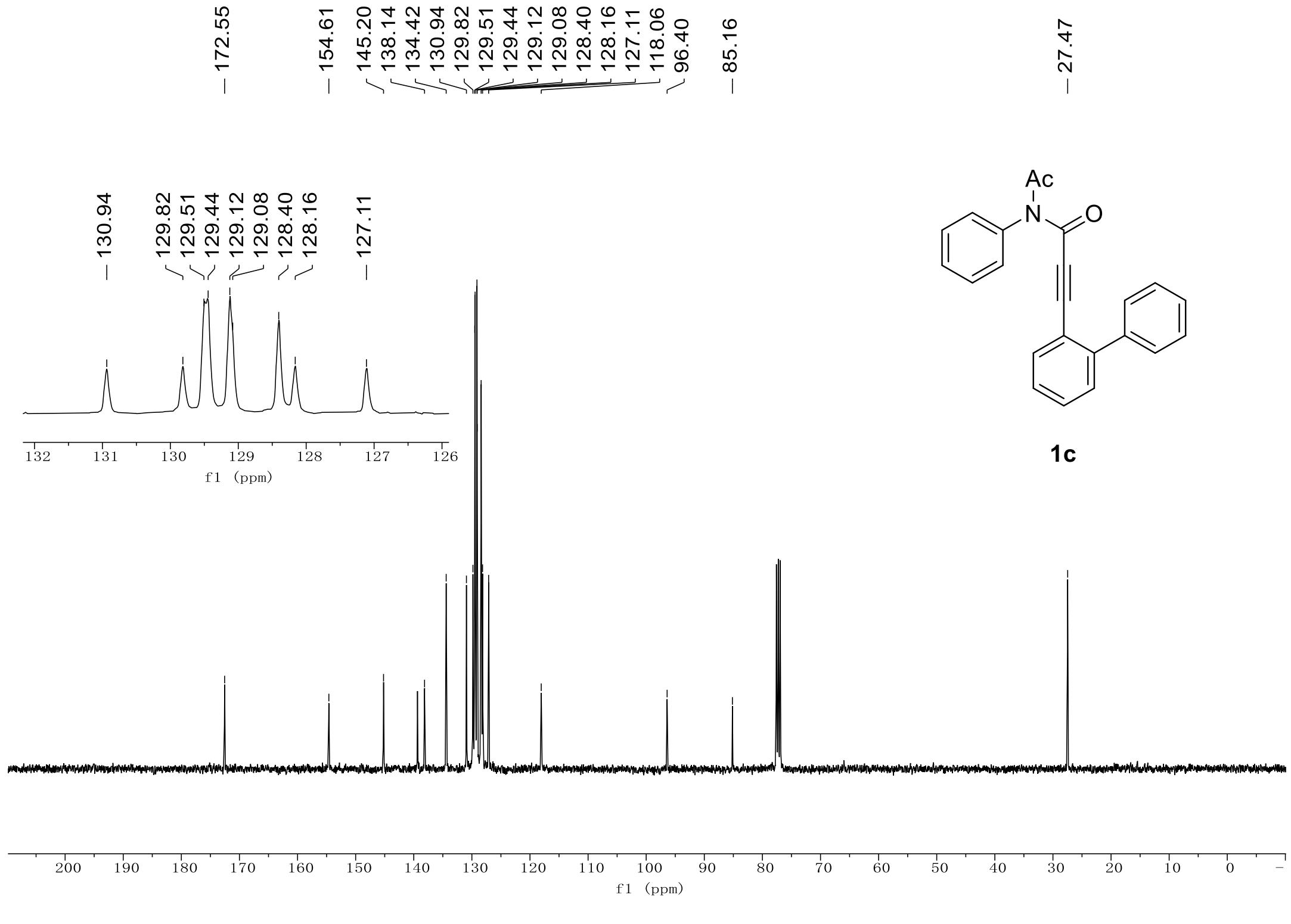

^1H NMR (400 MHz, $CDCl_3$)

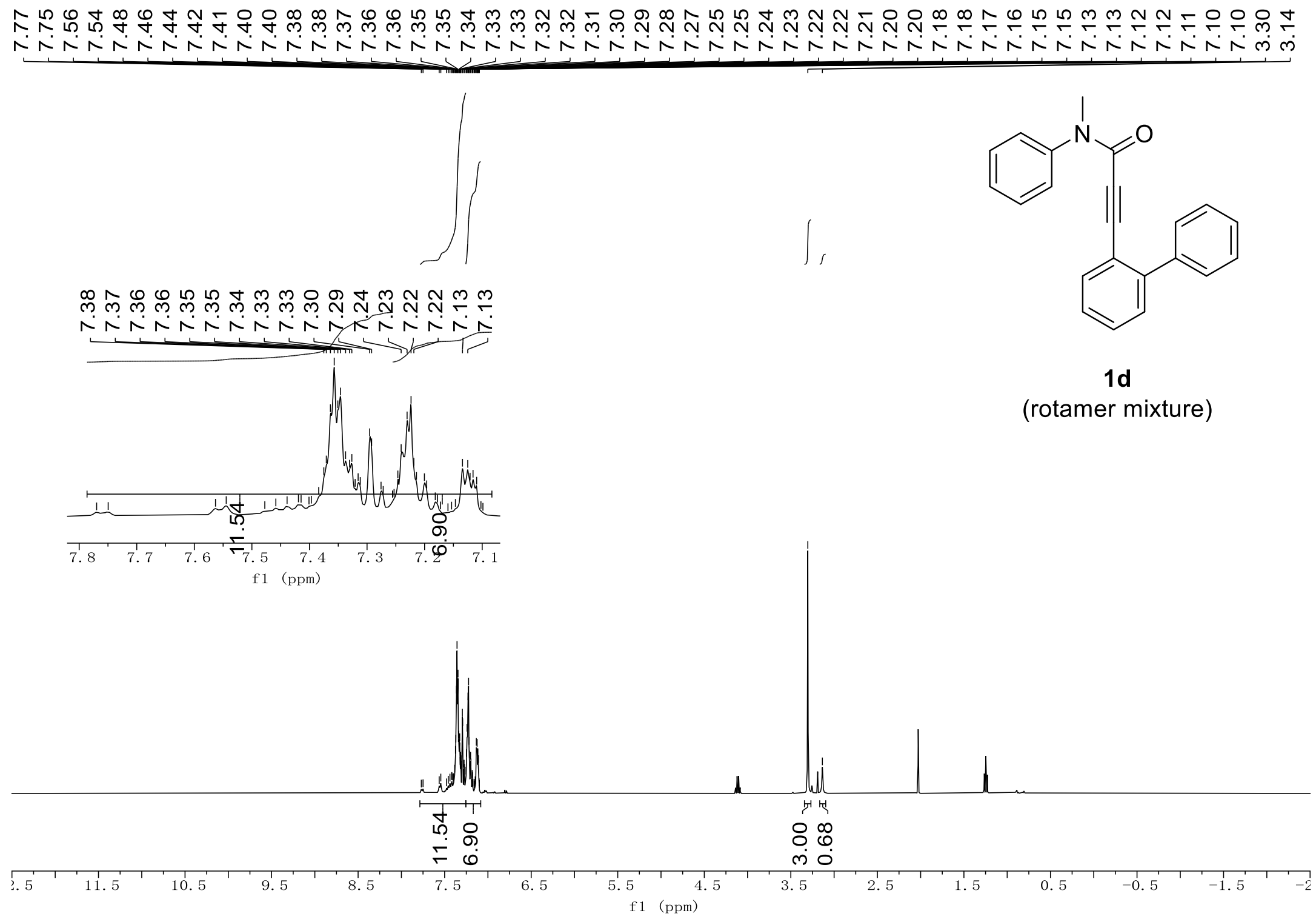

^{13}C NMR (100 MHz, $CDCl_3$) (Only signals corresponding to the major rotamer are reported)

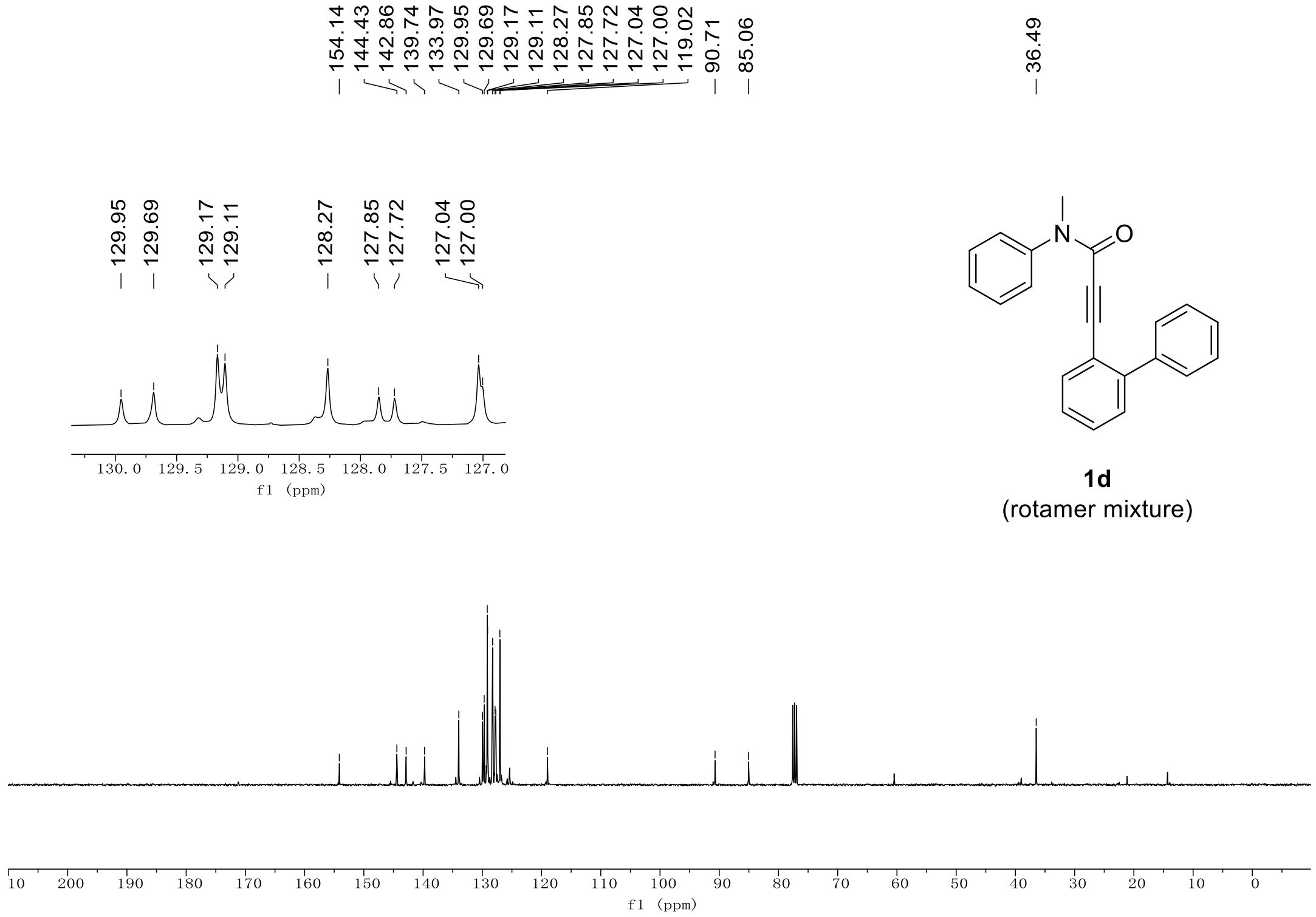

^{1}H NMR (400 MHz, $CDCl_3$)

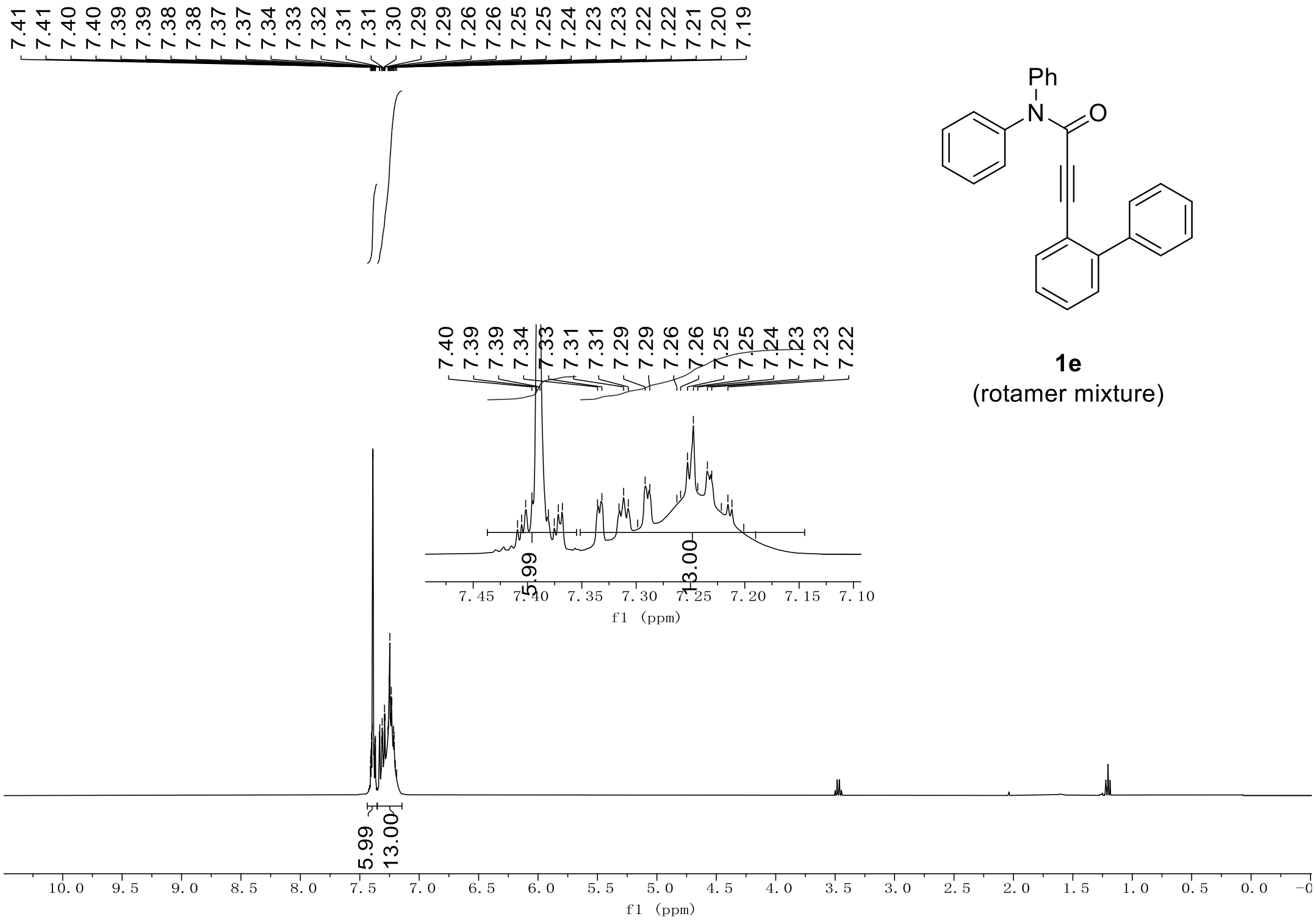

1e
(rotamer mixture)

^{13}C NMR (100 MHz, $CDCl_3$) (Only signals corresponding to the major rotamer are reported)

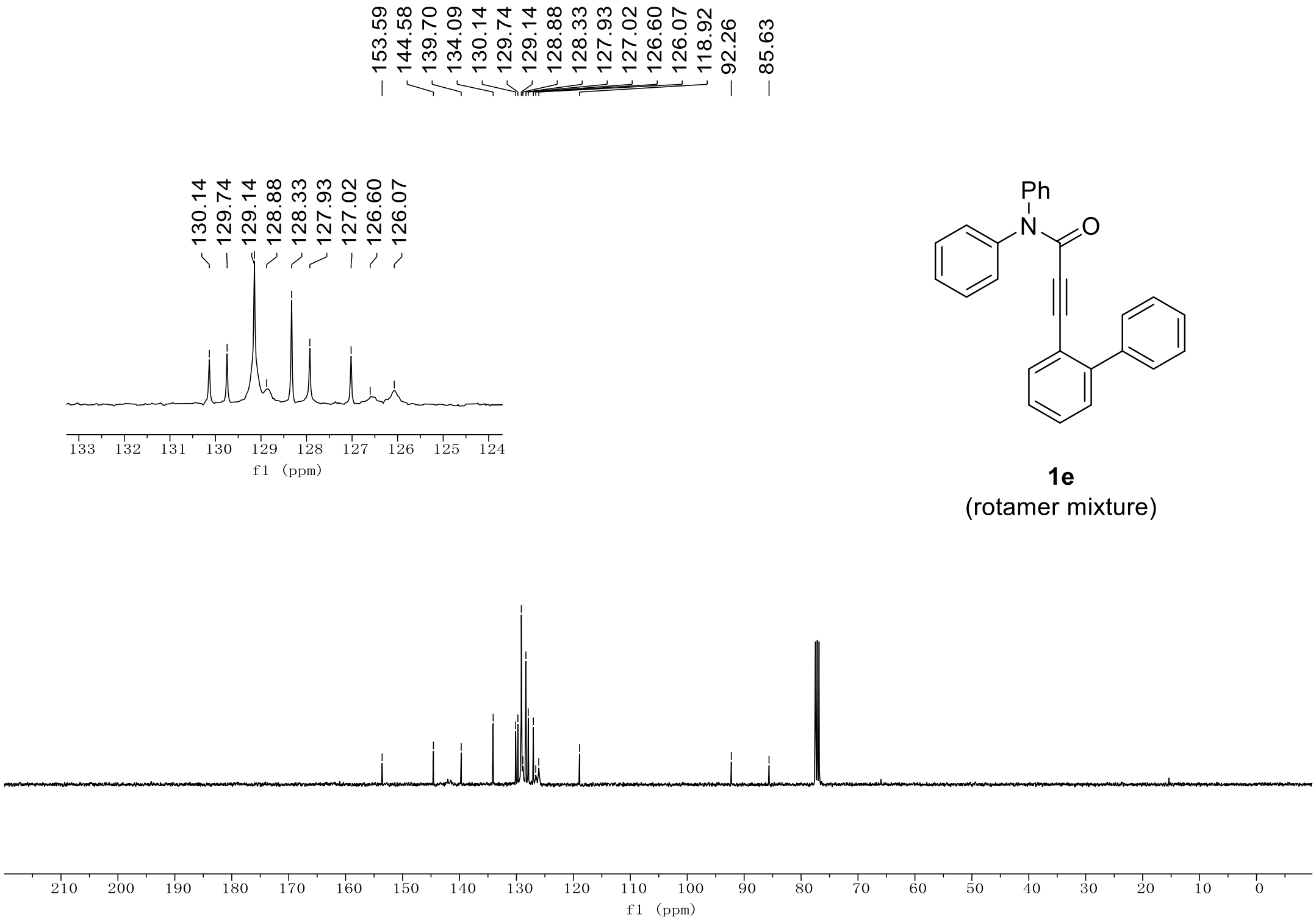

1e
(rotamer mixture)

^{1}H NMR (400 MHz, $CDCl_3$)

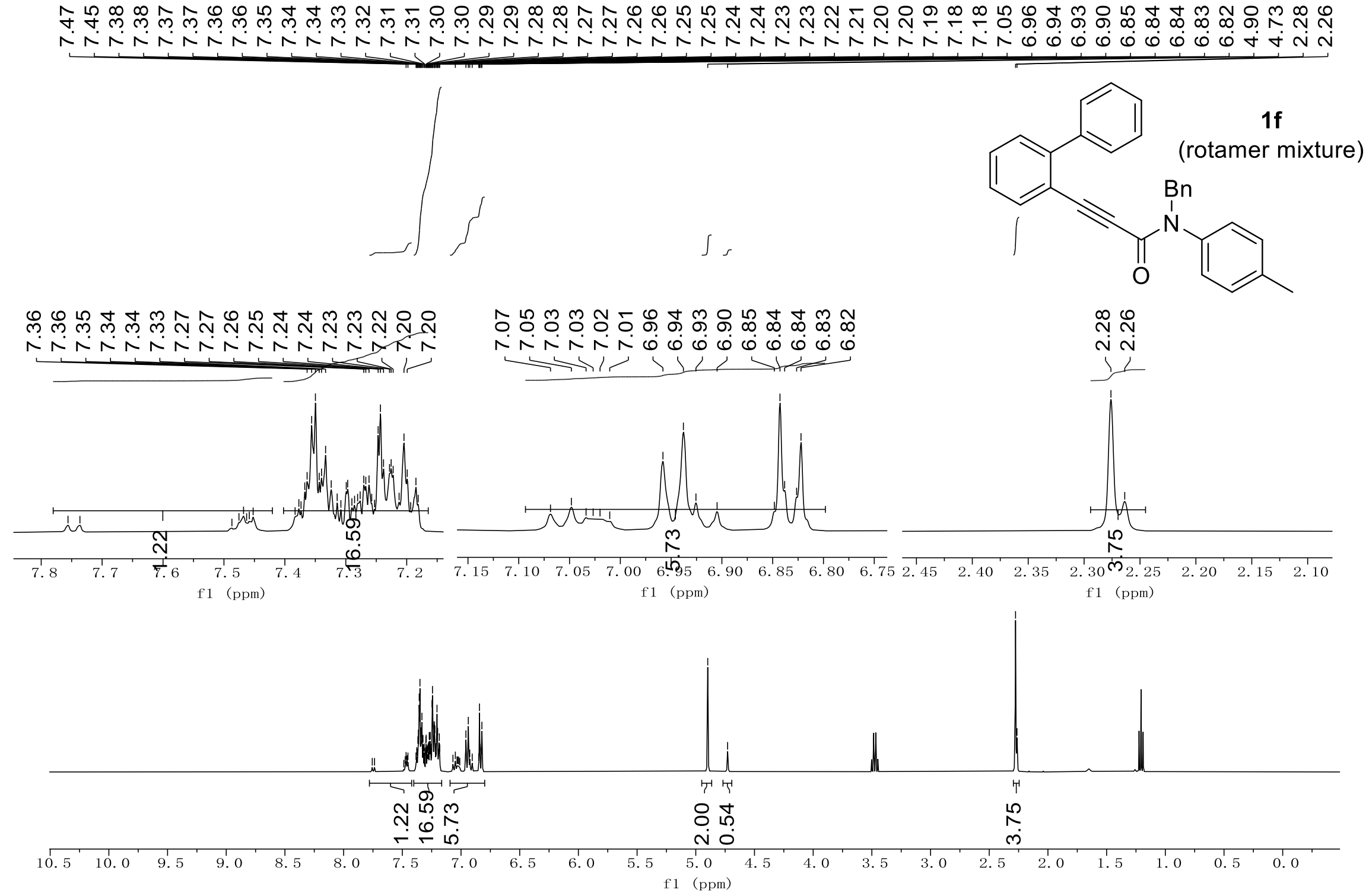

^{13}C NMR (100 MHz, $CDCl_3$) (Only signals corresponding to the major rotamer are reported)

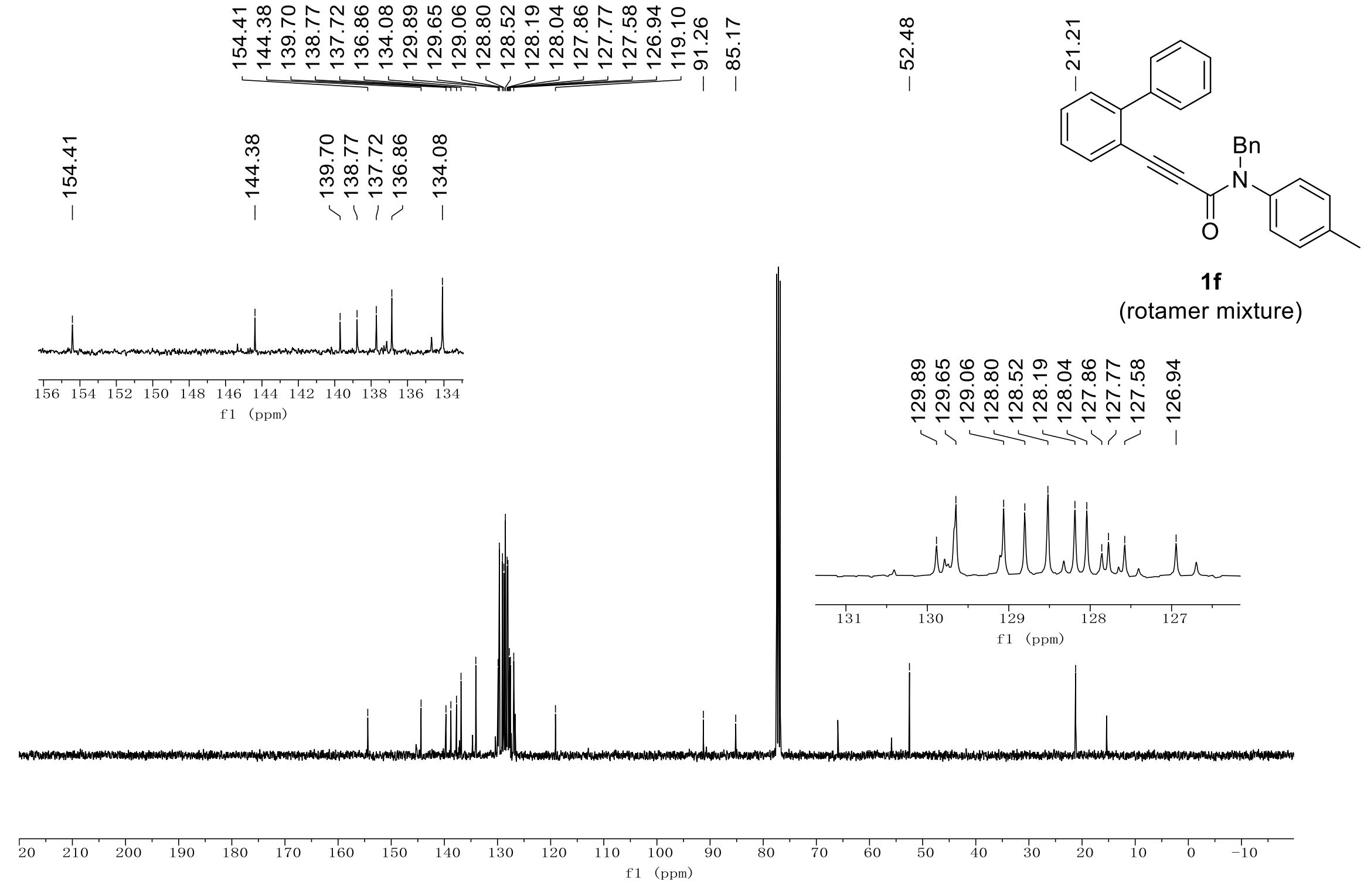

^{1}H NMR (400 MHz, $CDCl_3$)

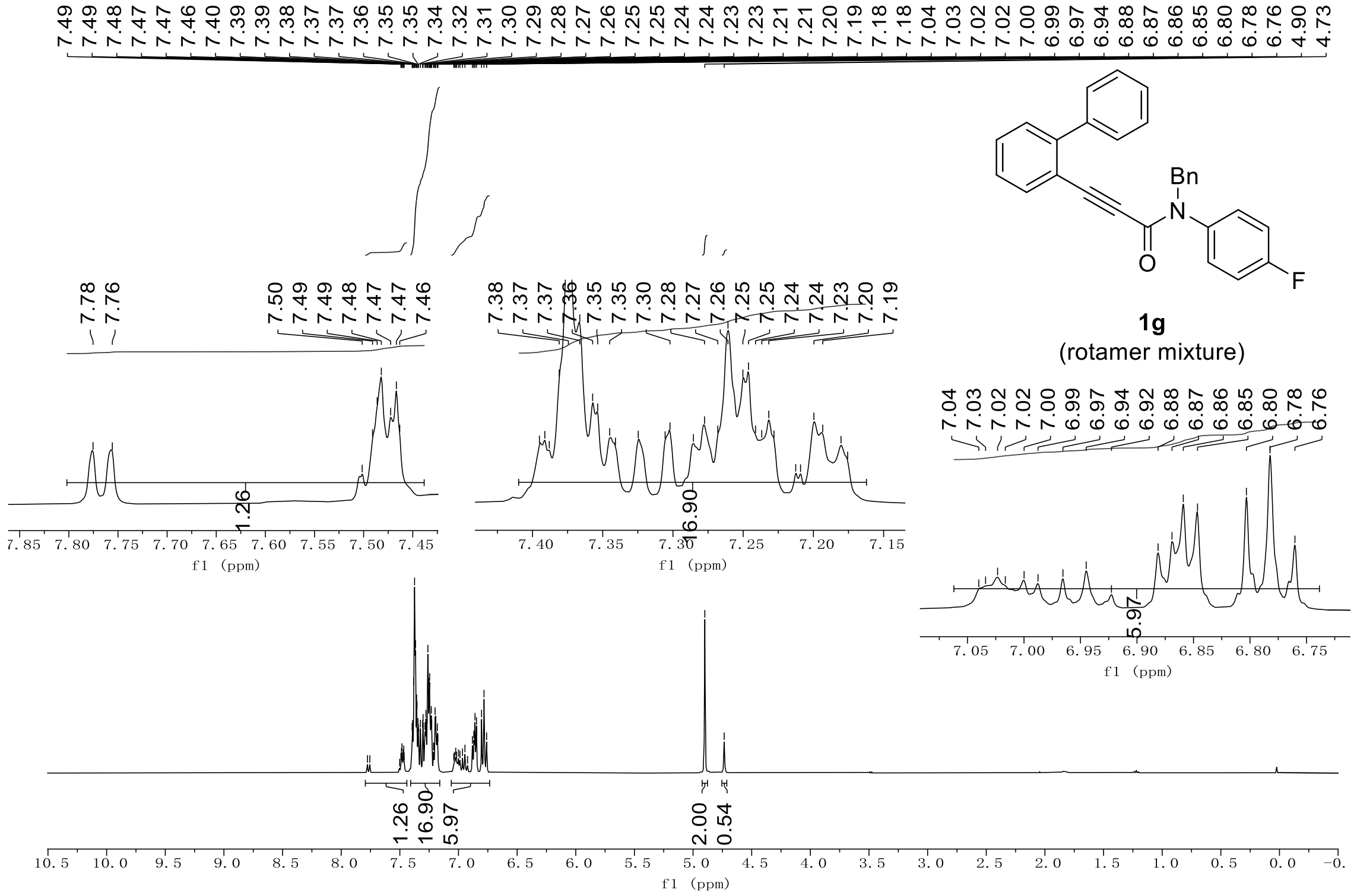

1g
(rotamer mixture)

^{13}C NMR (100 MHz, $CDCl_3$) (Only signals corresponding to the major rotamer are reported)

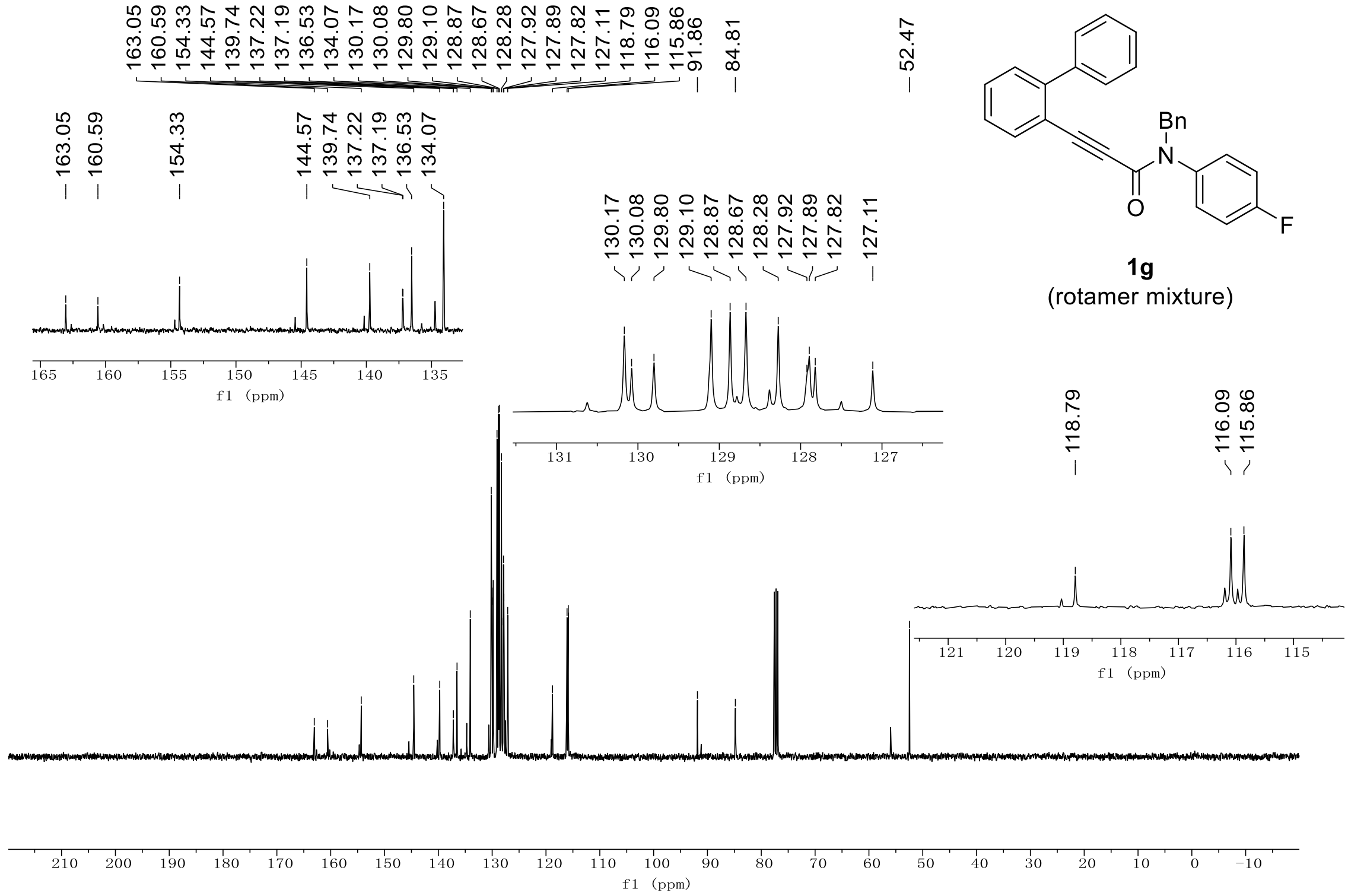

1g
(rotamer mixture)

^{19}F NMR (376 MHz, $CDCl_3$) (Only signals corresponding to the major rotamer are reported)

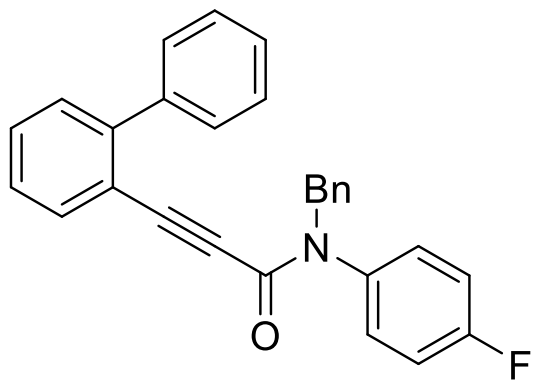

1g
(rotamer mixture)

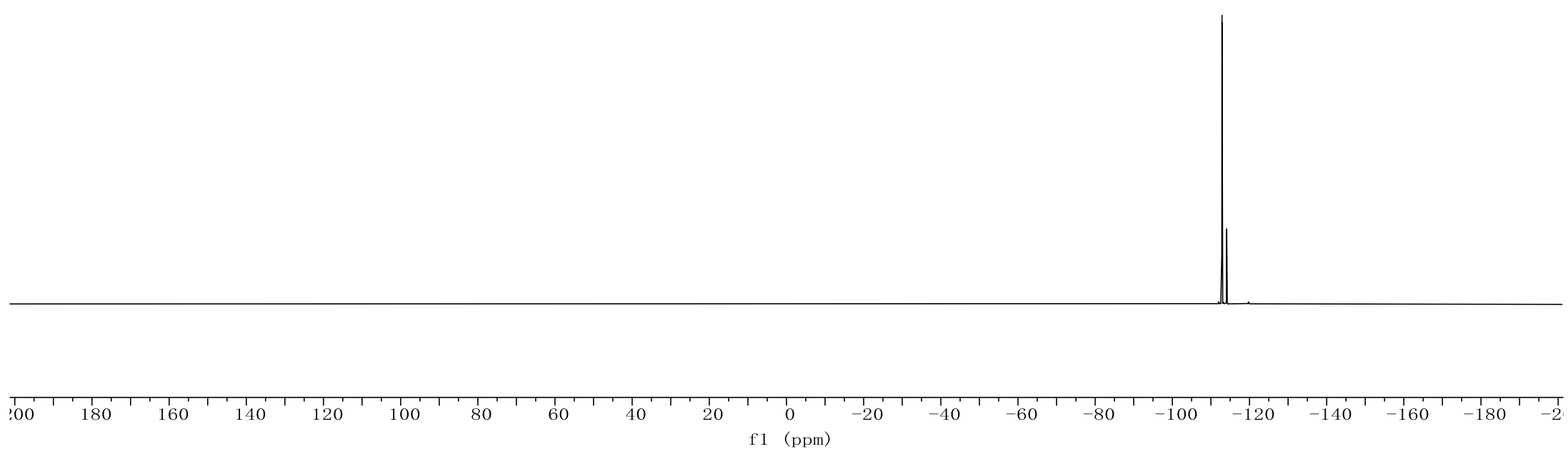

^{1}H NMR (400 MHz, $CDCl_3$)

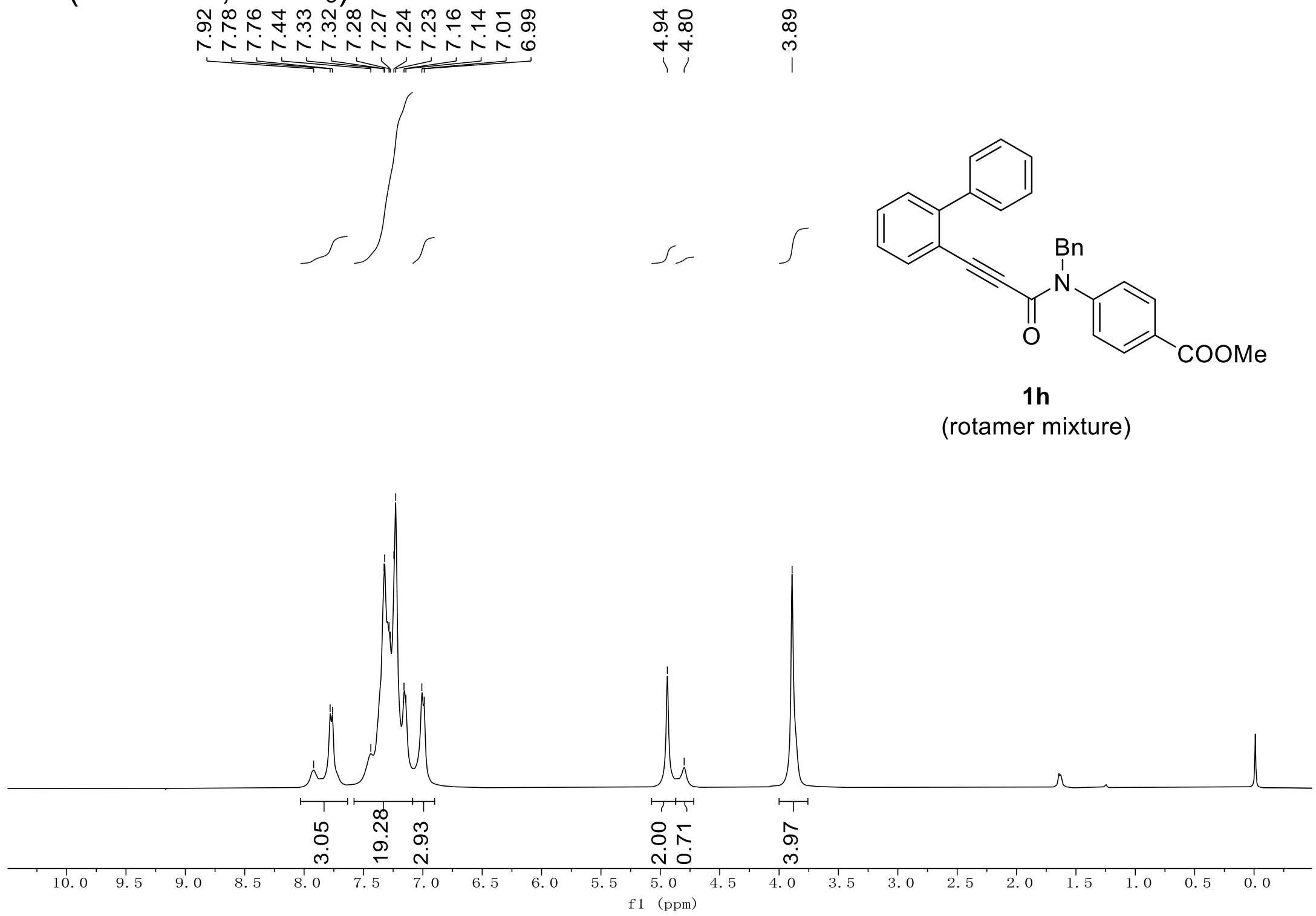

^{13}C NMR (100 MHz, $CDCl_3$) (Only signals corresponding to the major rotamer are reported)

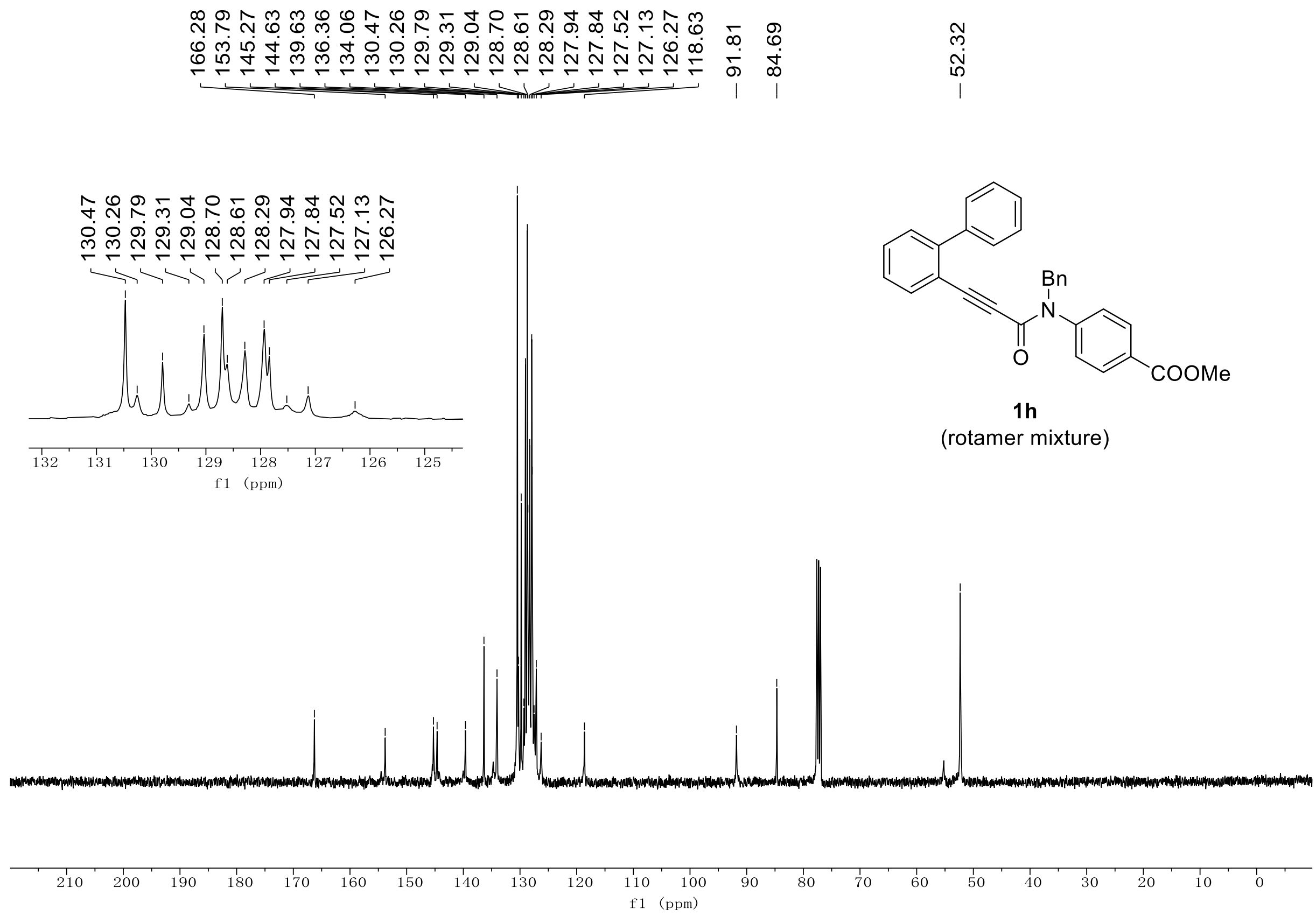

^{1}H NMR (400 MHz, $CDCl_3$)

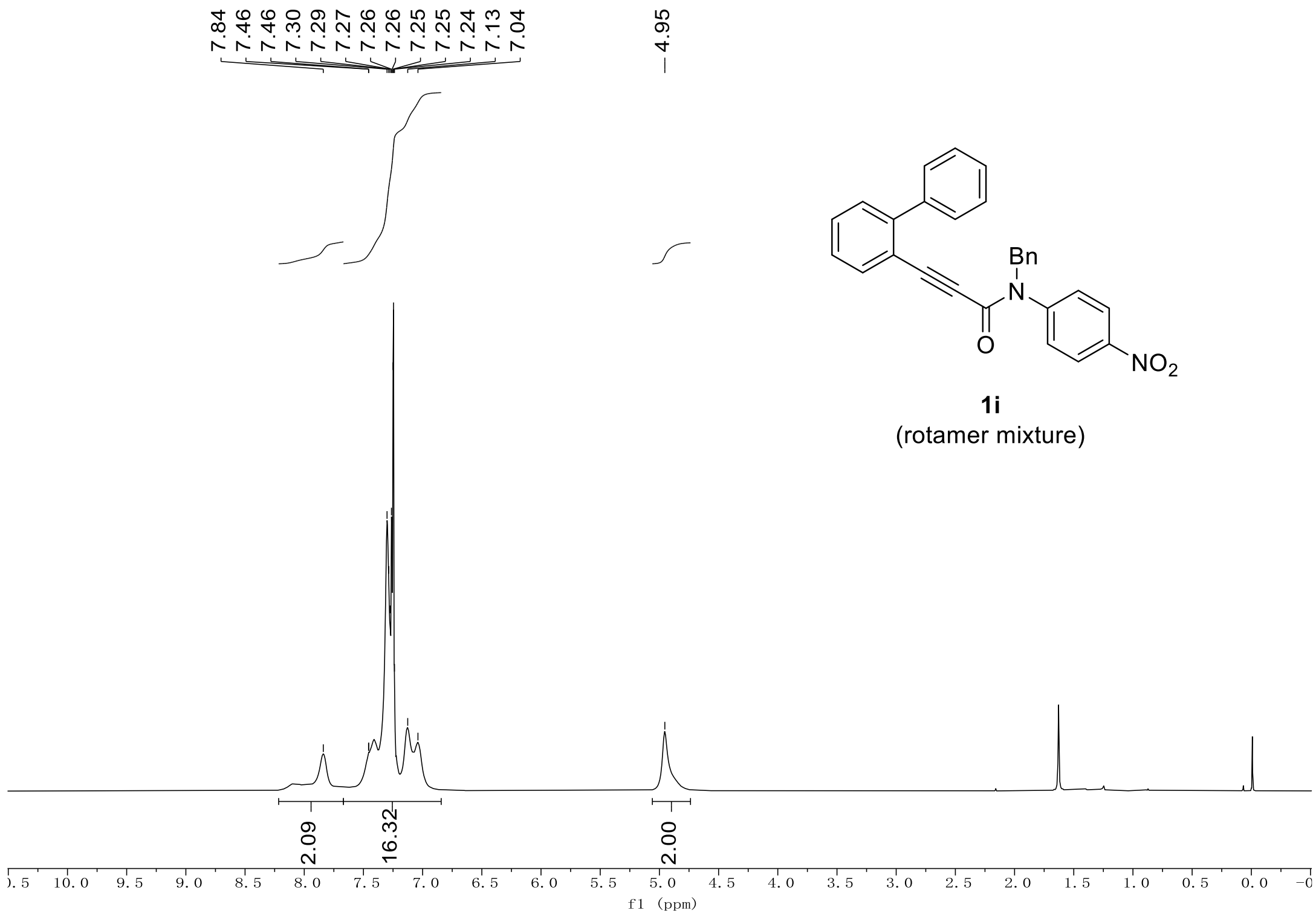

^{13}C NMR (100 MHz, $CDCl_3$) (Only signals corresponding to the major rotamer are reported)

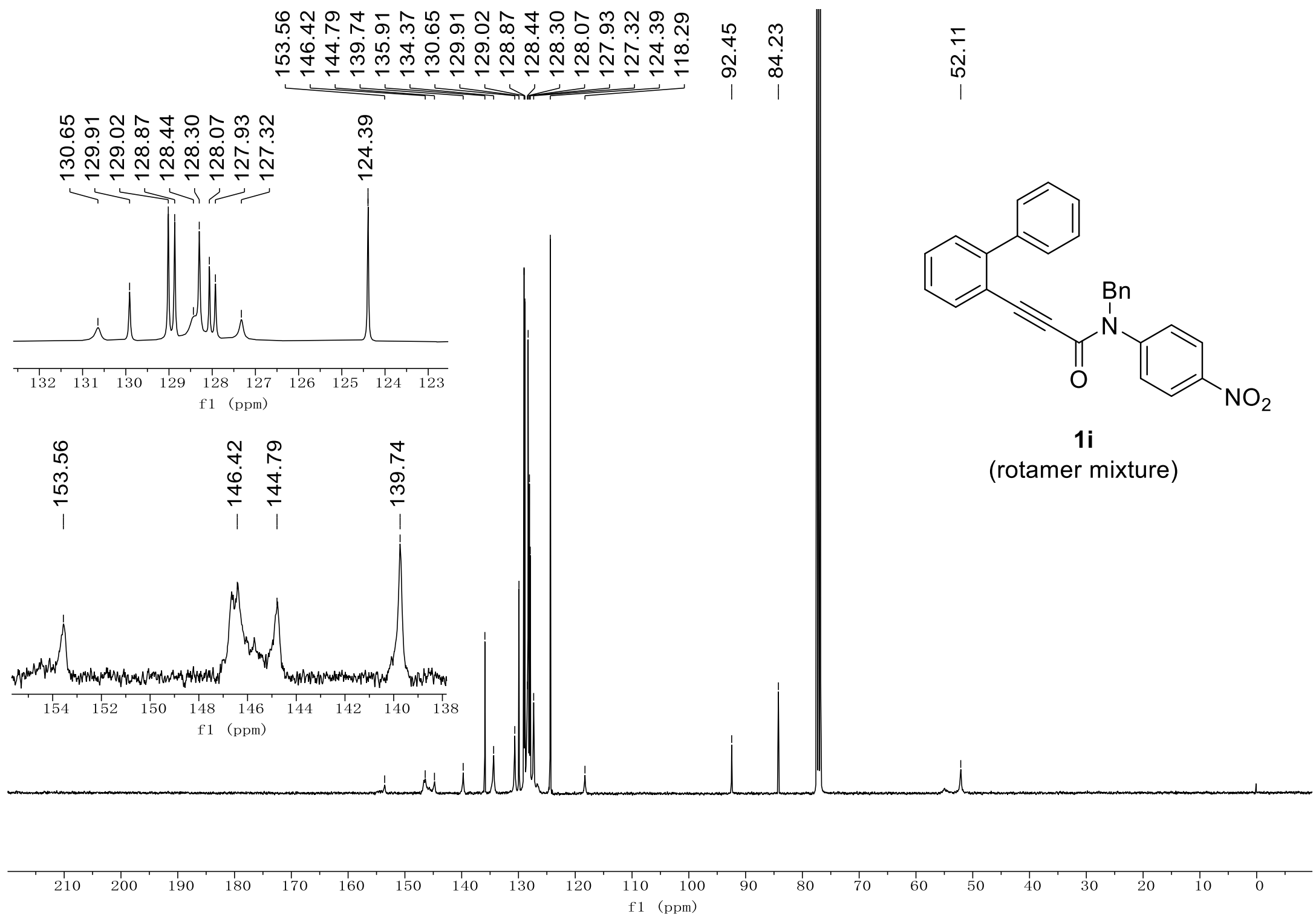

^{1}H NMR (400 MHz, $CDCl_3$)

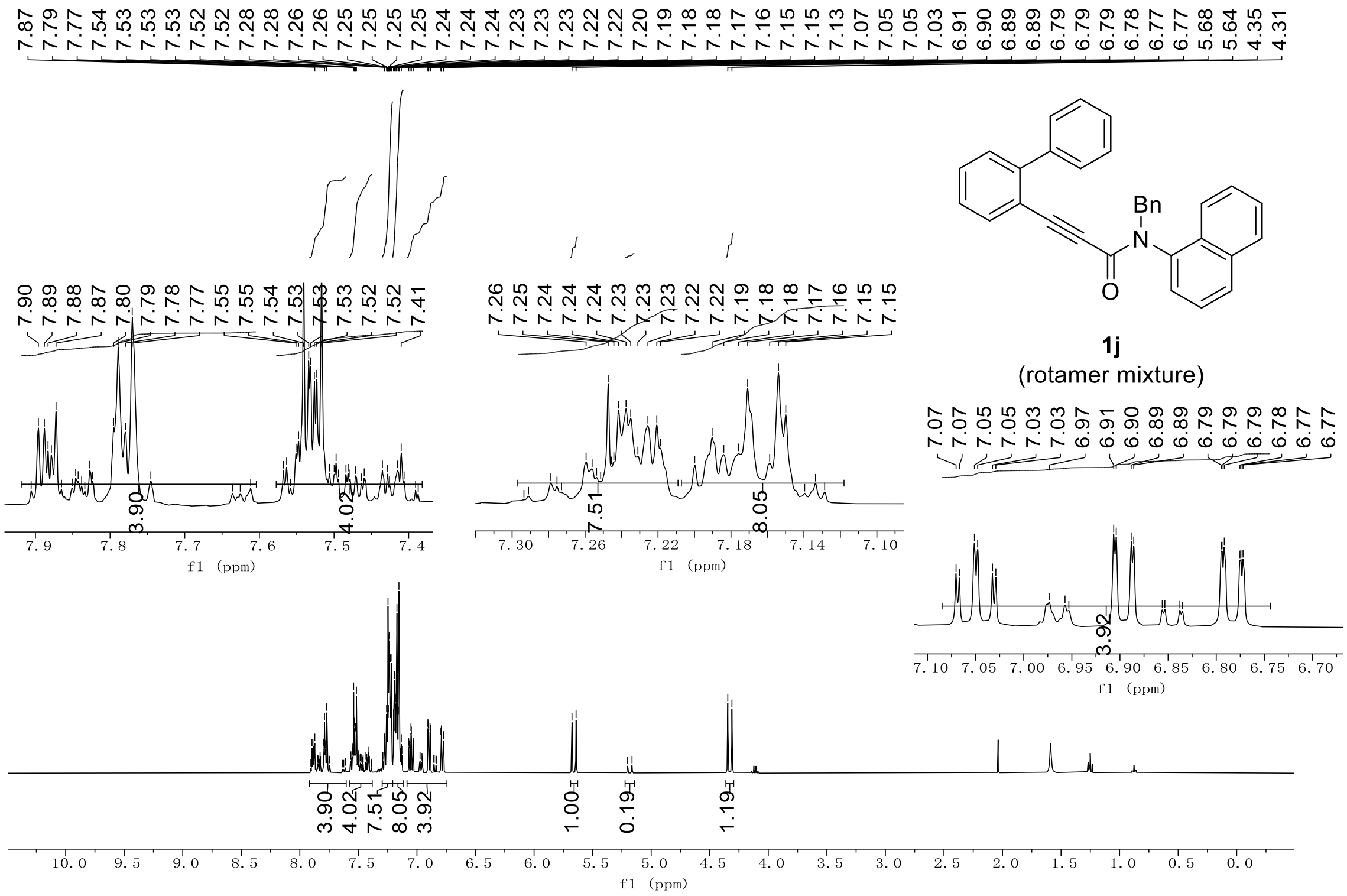

^{13}C NMR (100 MHz, $CDCl_3$) (Only signals corresponding to the major rotamer are reported)

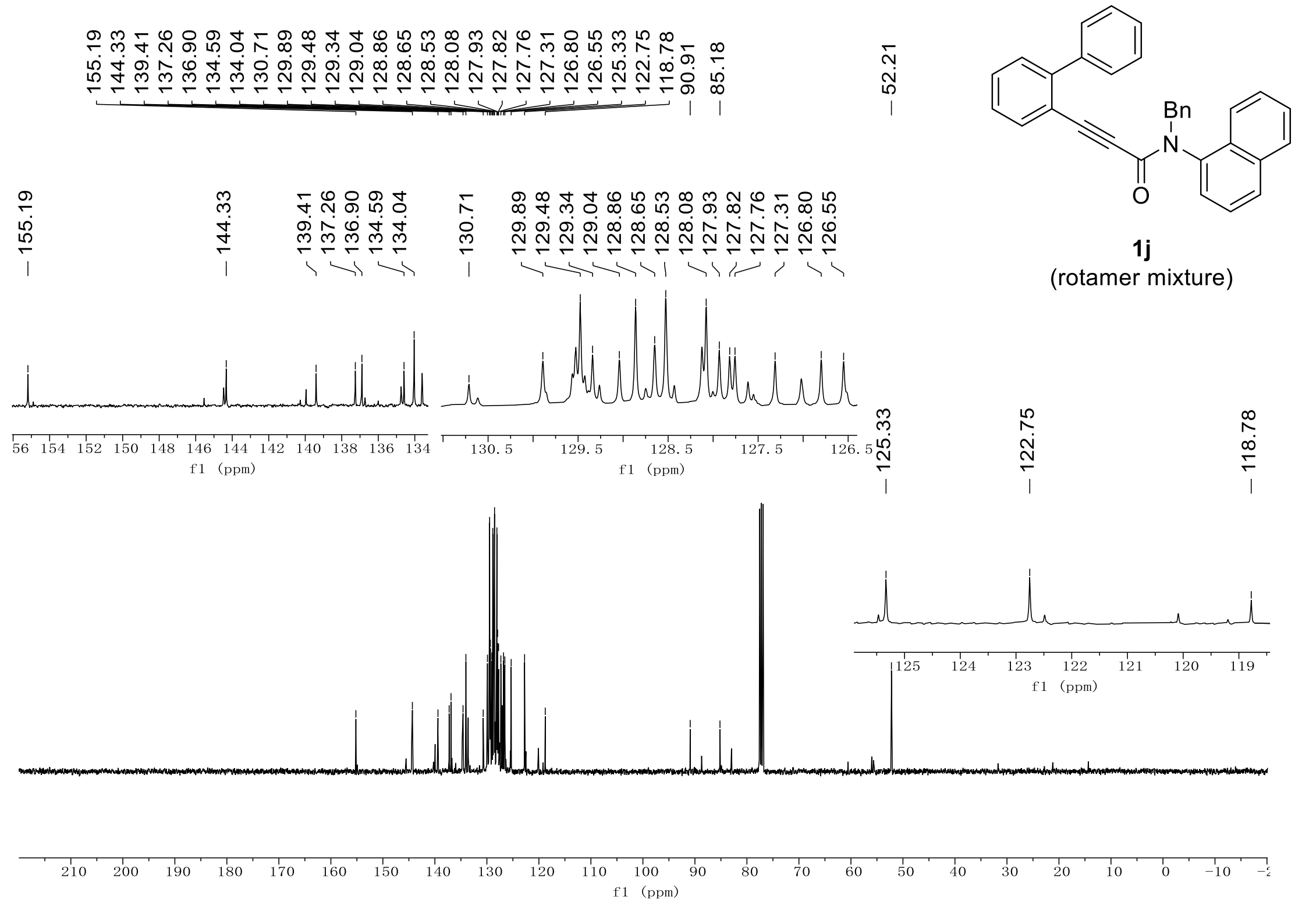

^{1}H NMR (400 MHz, $CDCl_3$)

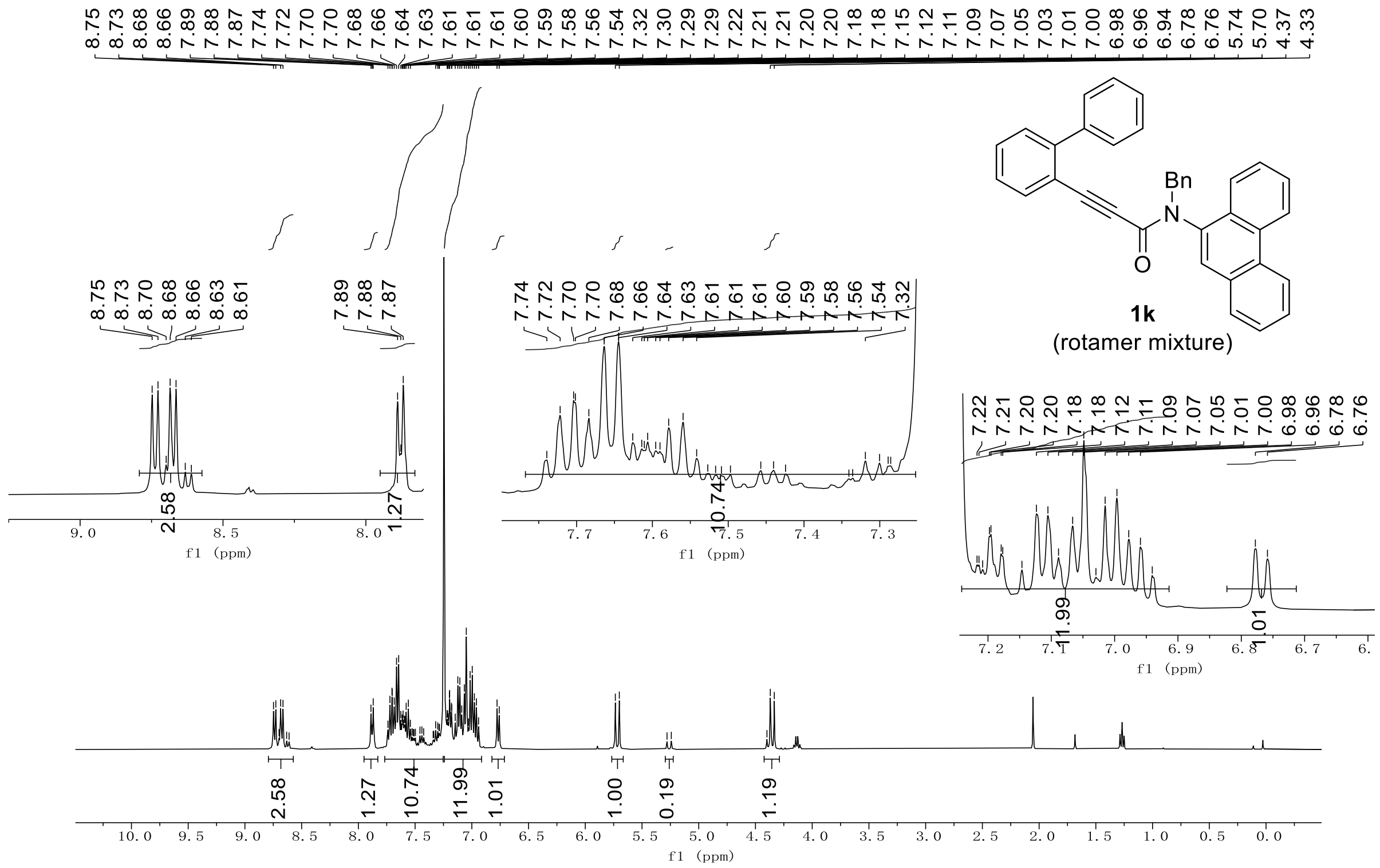

^{13}C NMR (100 MHz, $CDCl_3$) (Only signals corresponding to the major rotamer are reported)

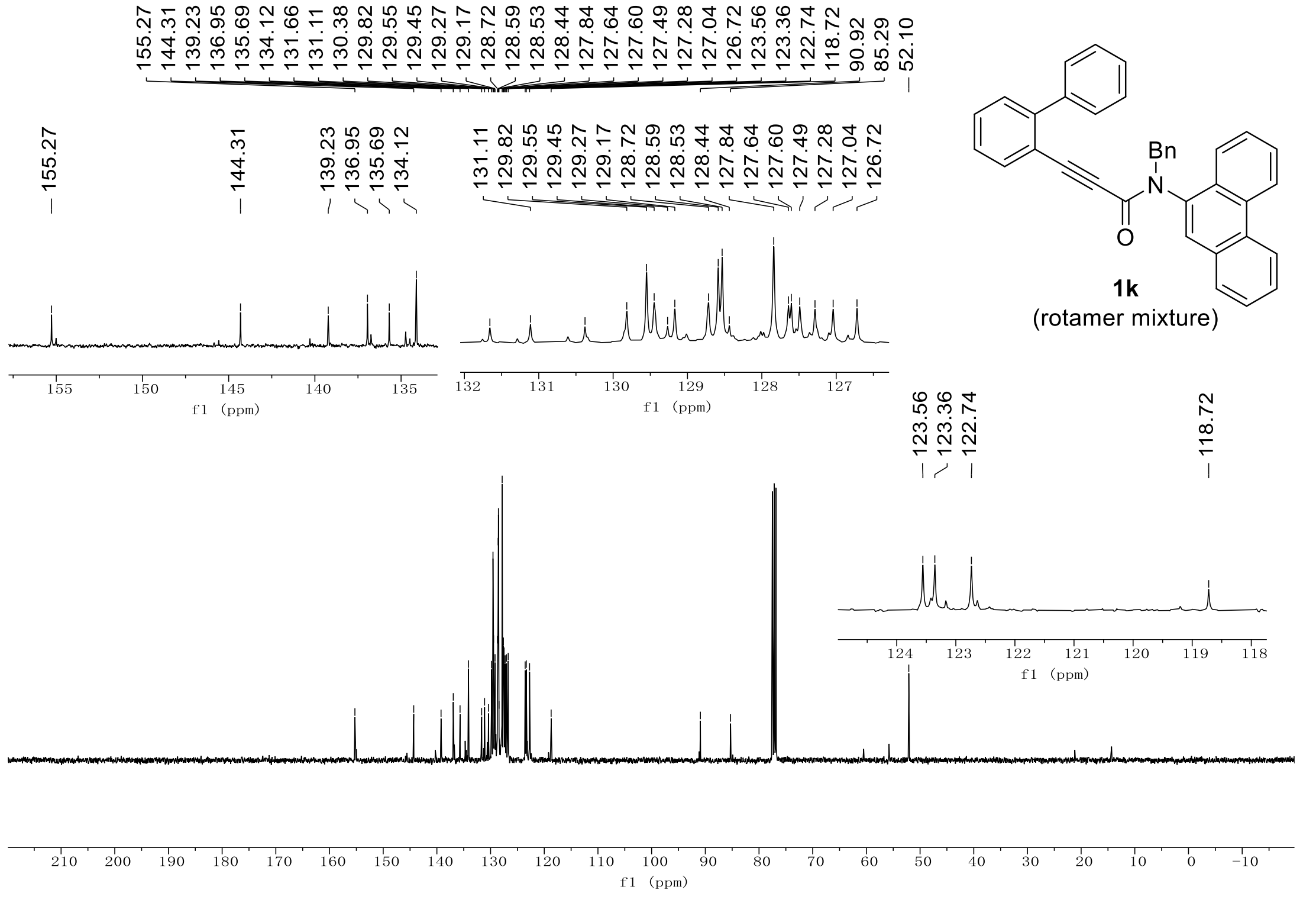

^{1}H NMR (400 MHz, $CDCl_3$)

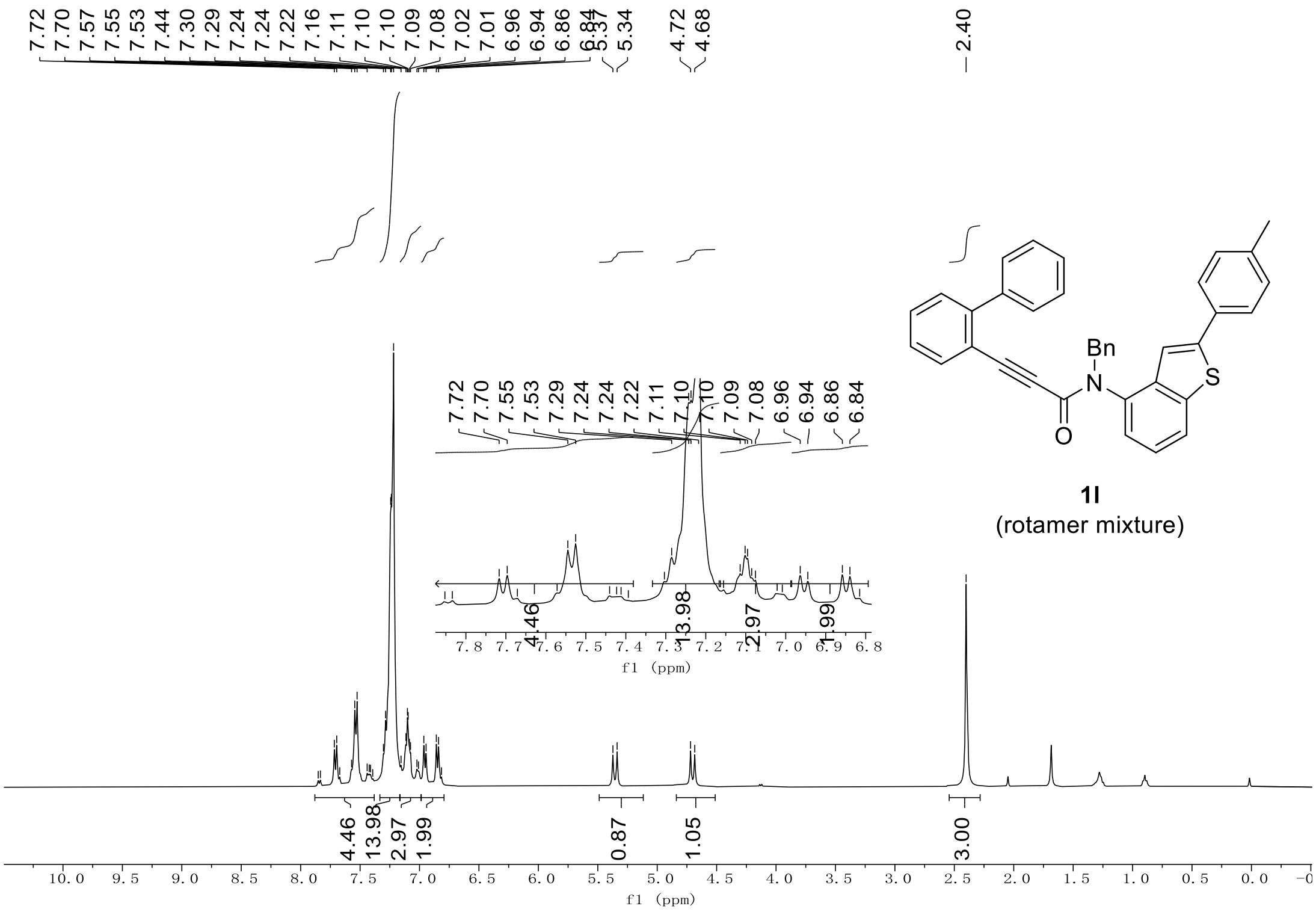

1l
(rotamer mixture)

^{13}C NMR (100 MHz, $CDCl_3$) (Only signals corresponding to the major rotamer are reported)

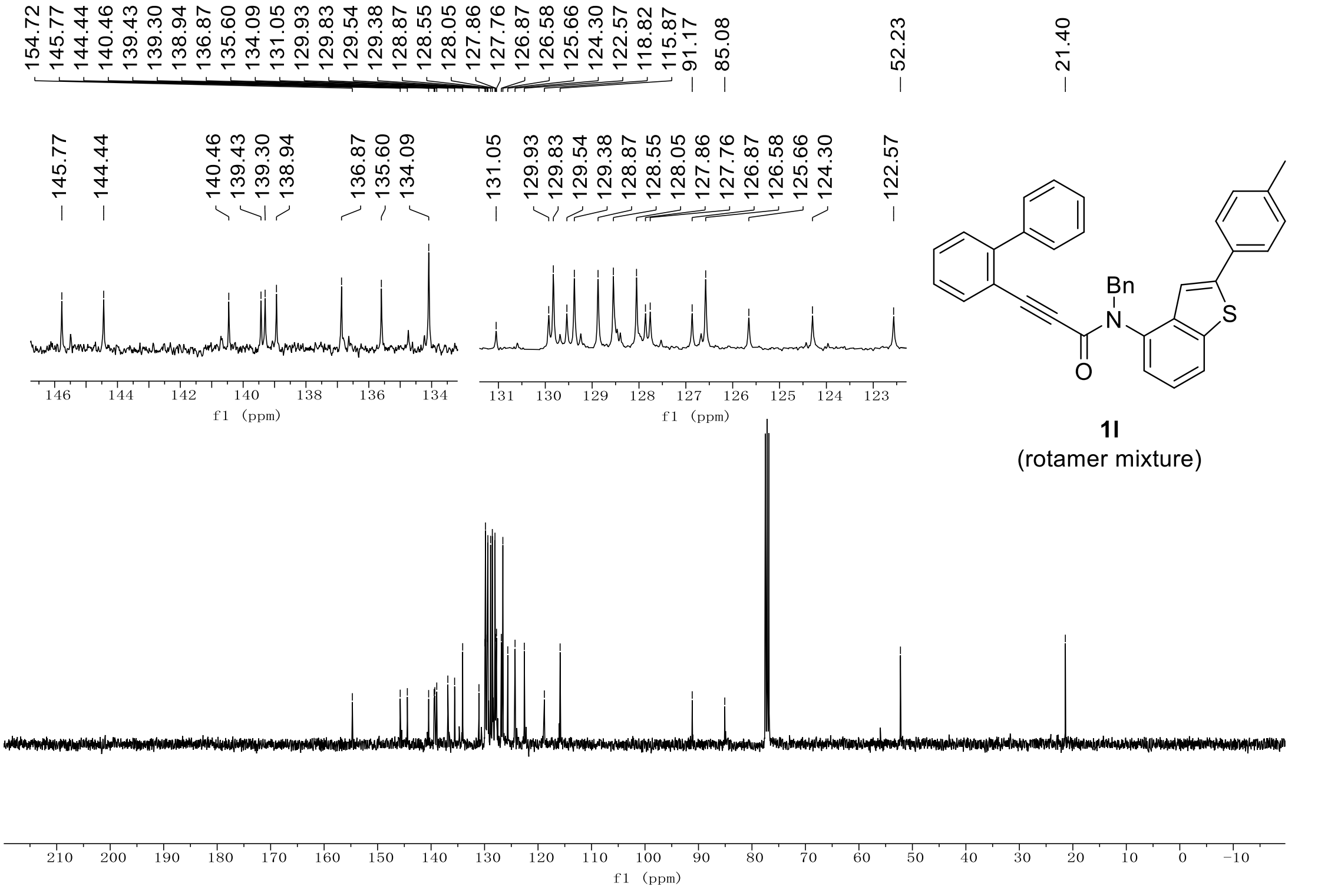

1l
(rotamer mixture)

^{1}H NMR (400 MHz, $CDCl_3$)

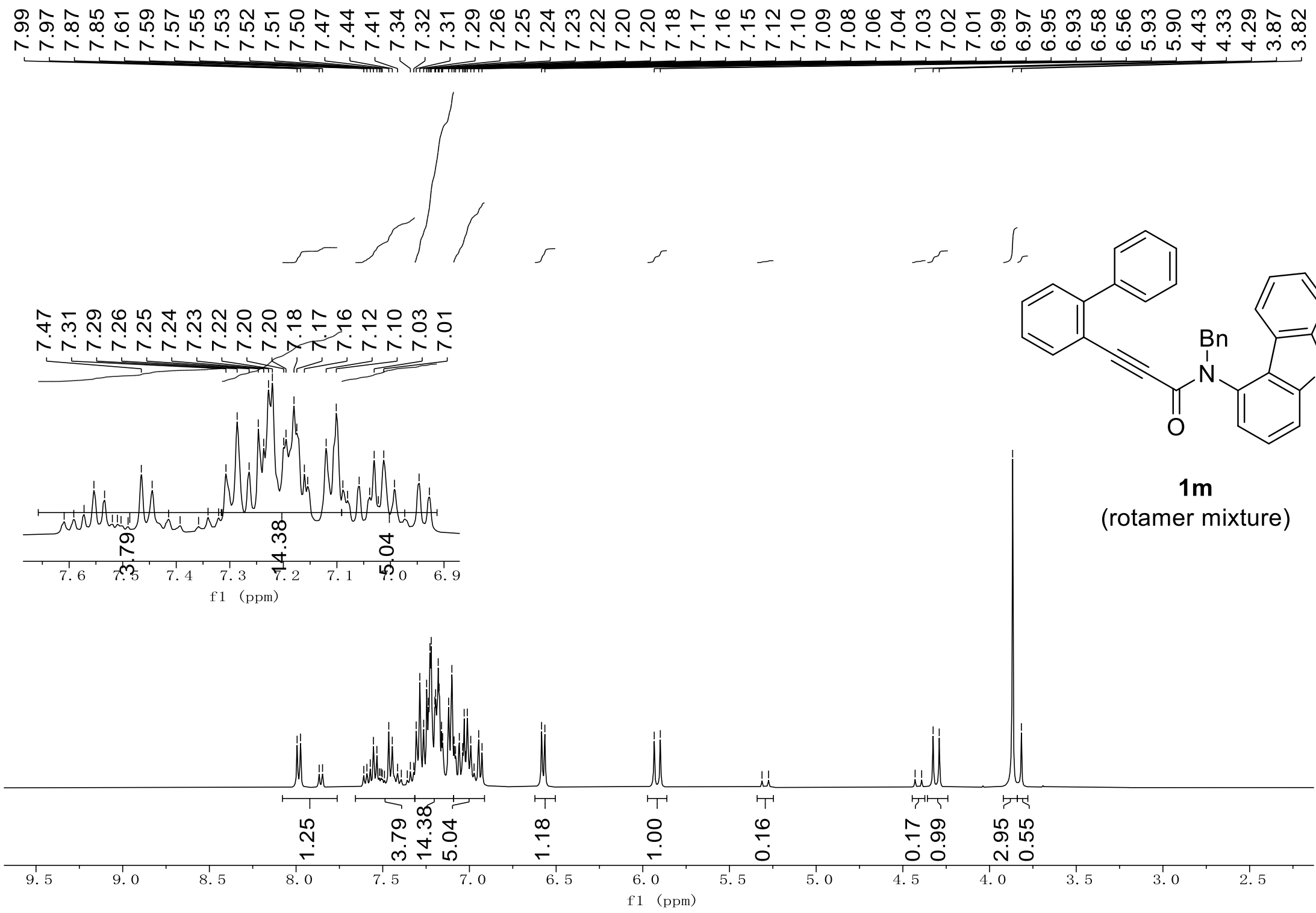

1m
(rotamer mixture)

^{13}C NMR (100 MHz, $CDCl_3$) (Only signals corresponding to the major rotamer are reported)

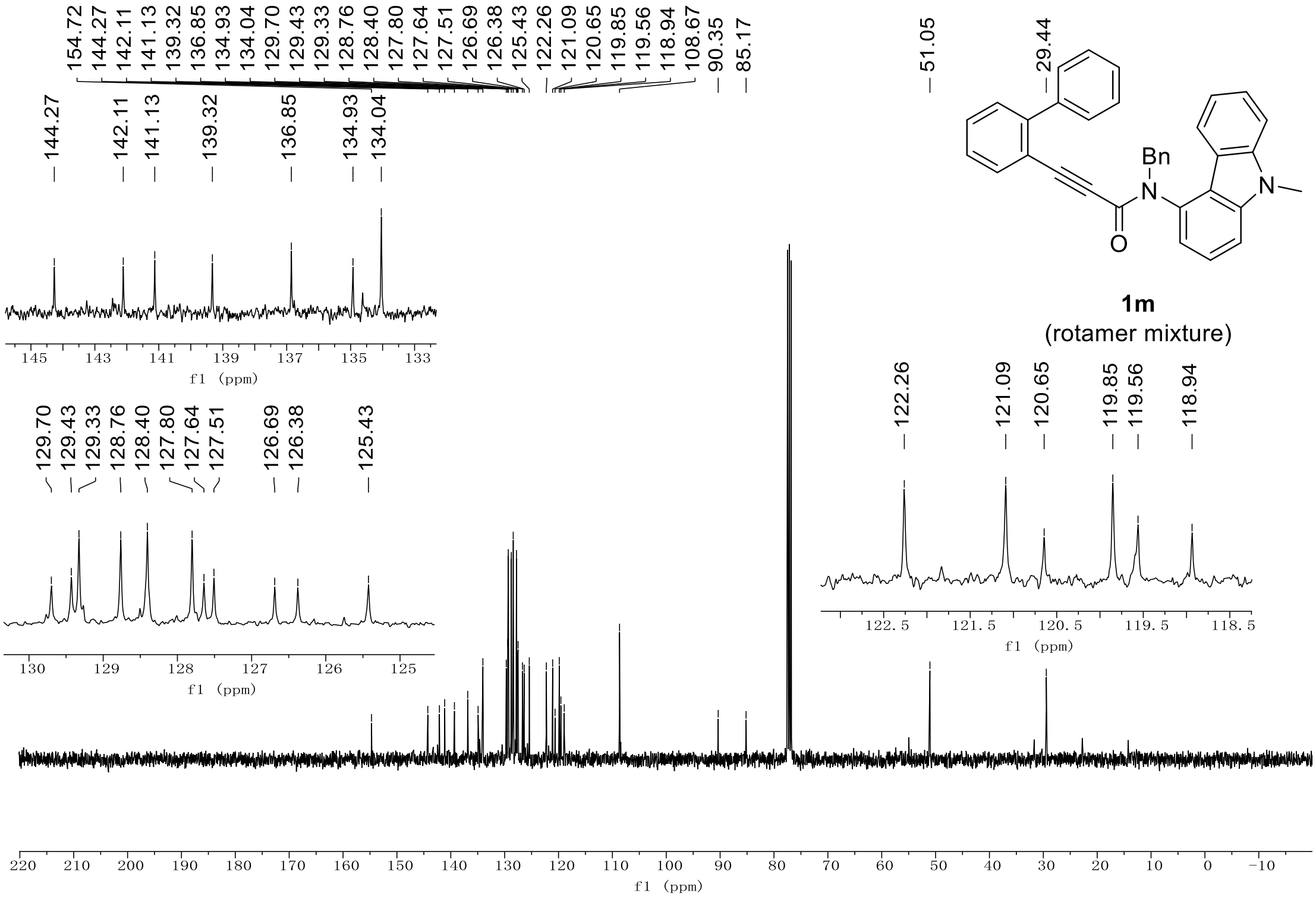

1m
(rotamer mixture)

^{1}H NMR (400 MHz, $CDCl_3$)

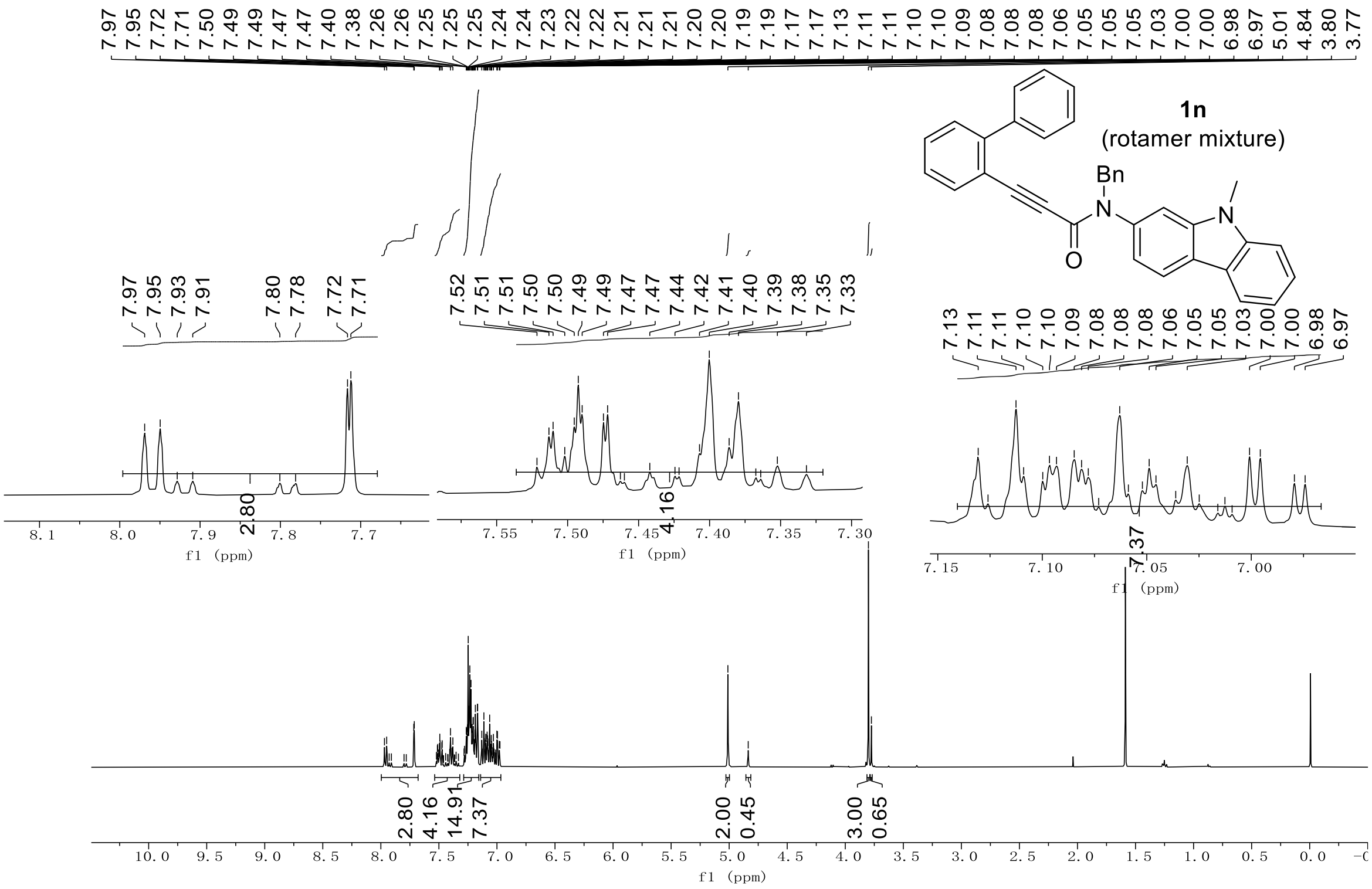

^{13}C NMR (100 MHz, $CDCl_3$) (Only signals corresponding to the major rotamer are reported)

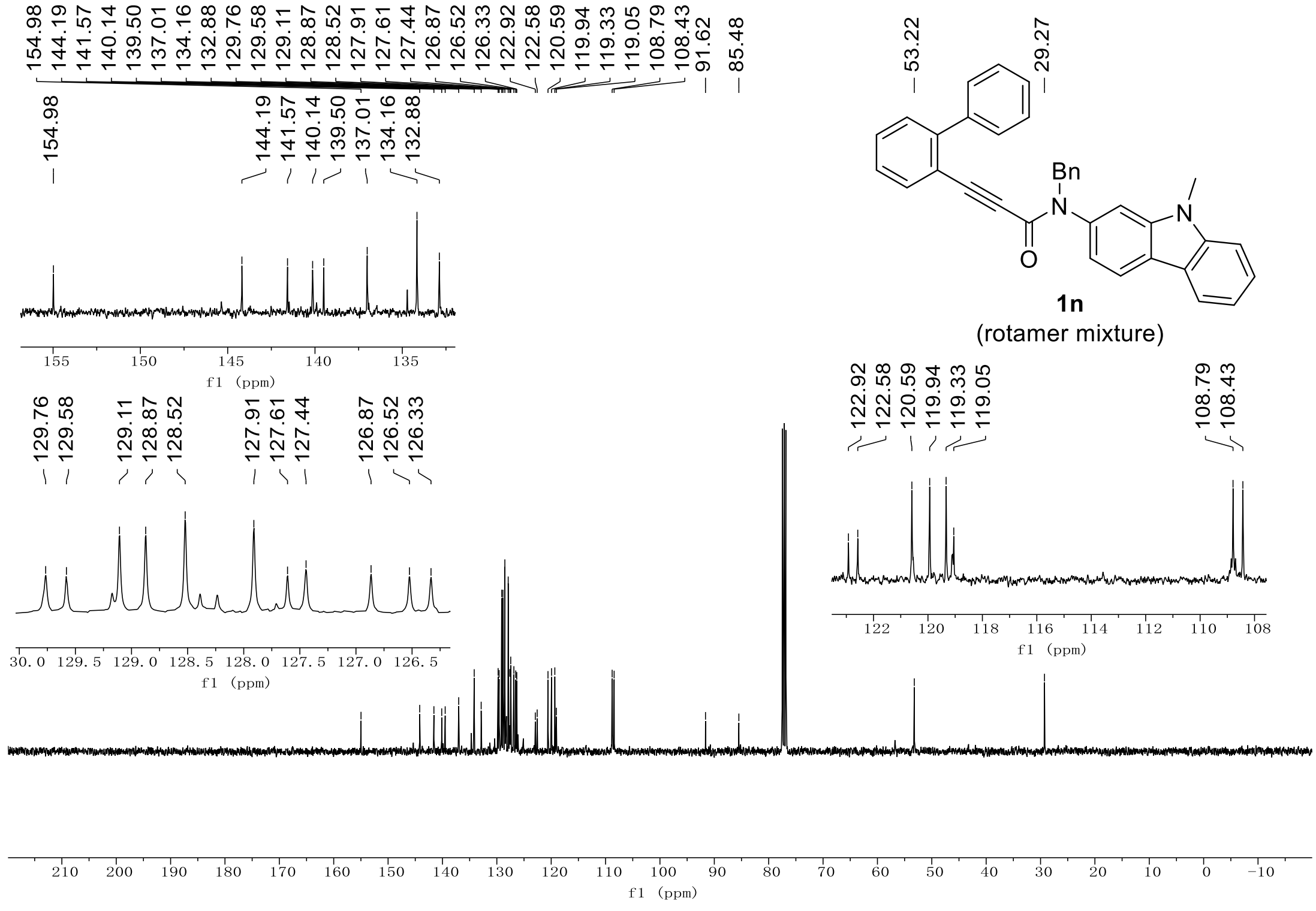

^{1}H NMR (400 MHz, $CDCl_3$)

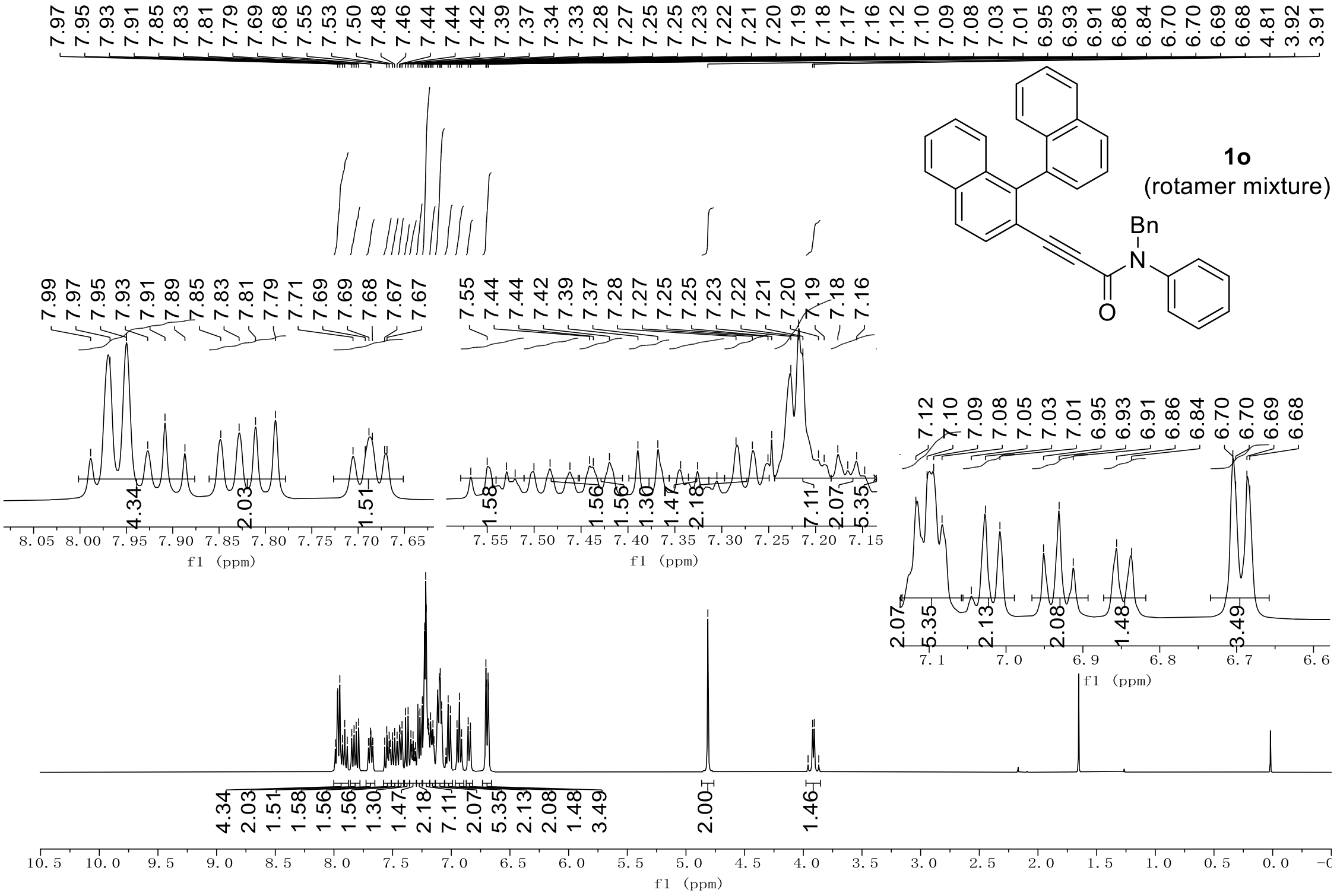

^{13}C NMR (100 MHz, $CDCl_3$) (Only signals corresponding to the major rotamer are reported)

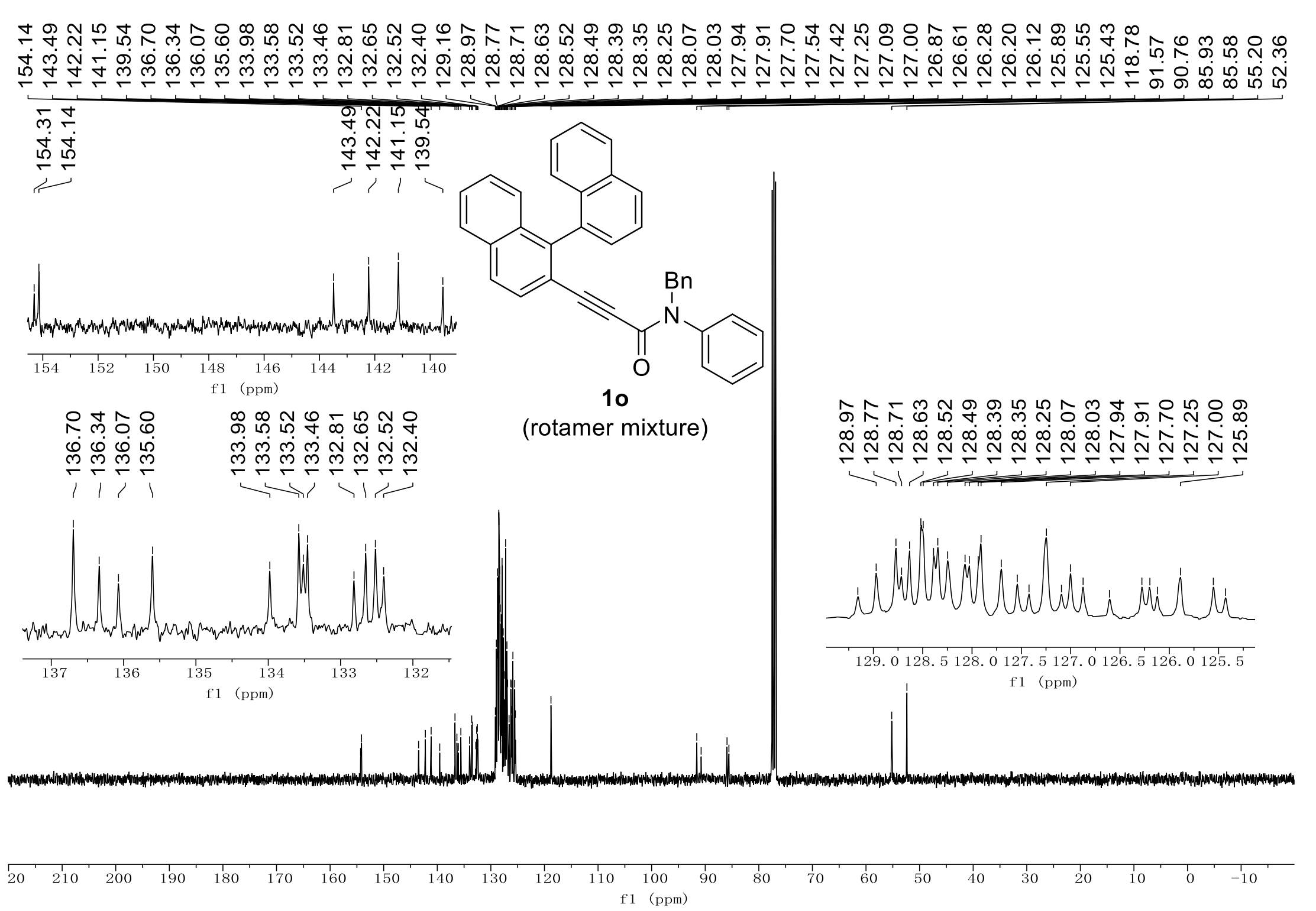

^{1}H NMR (400 MHz, $CDCl_3$)

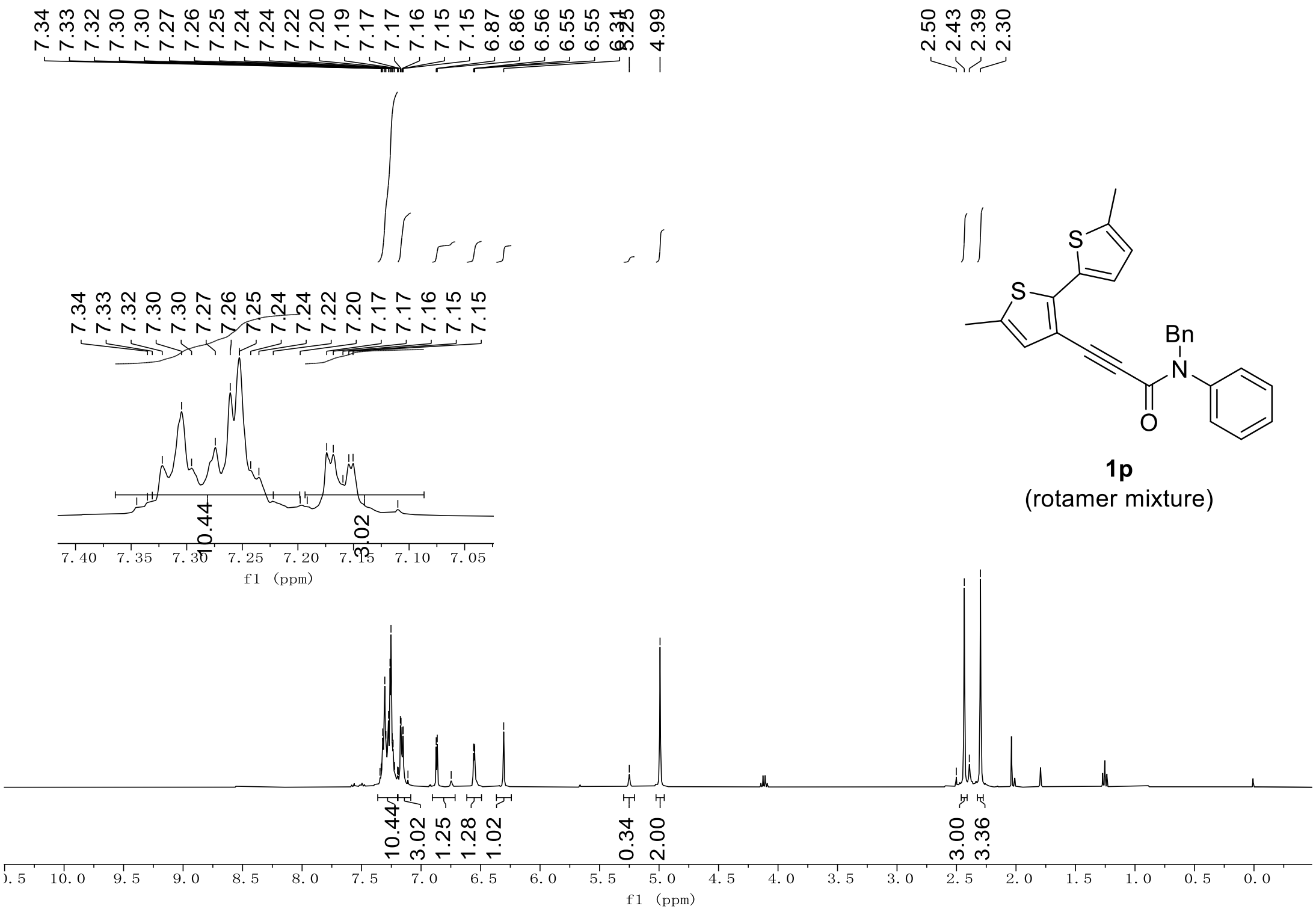

1p
(rotamer mixture)

^{13}C NMR (100 MHz, $CDCl_3$) (Only signals corresponding to the major rotamer are reported)

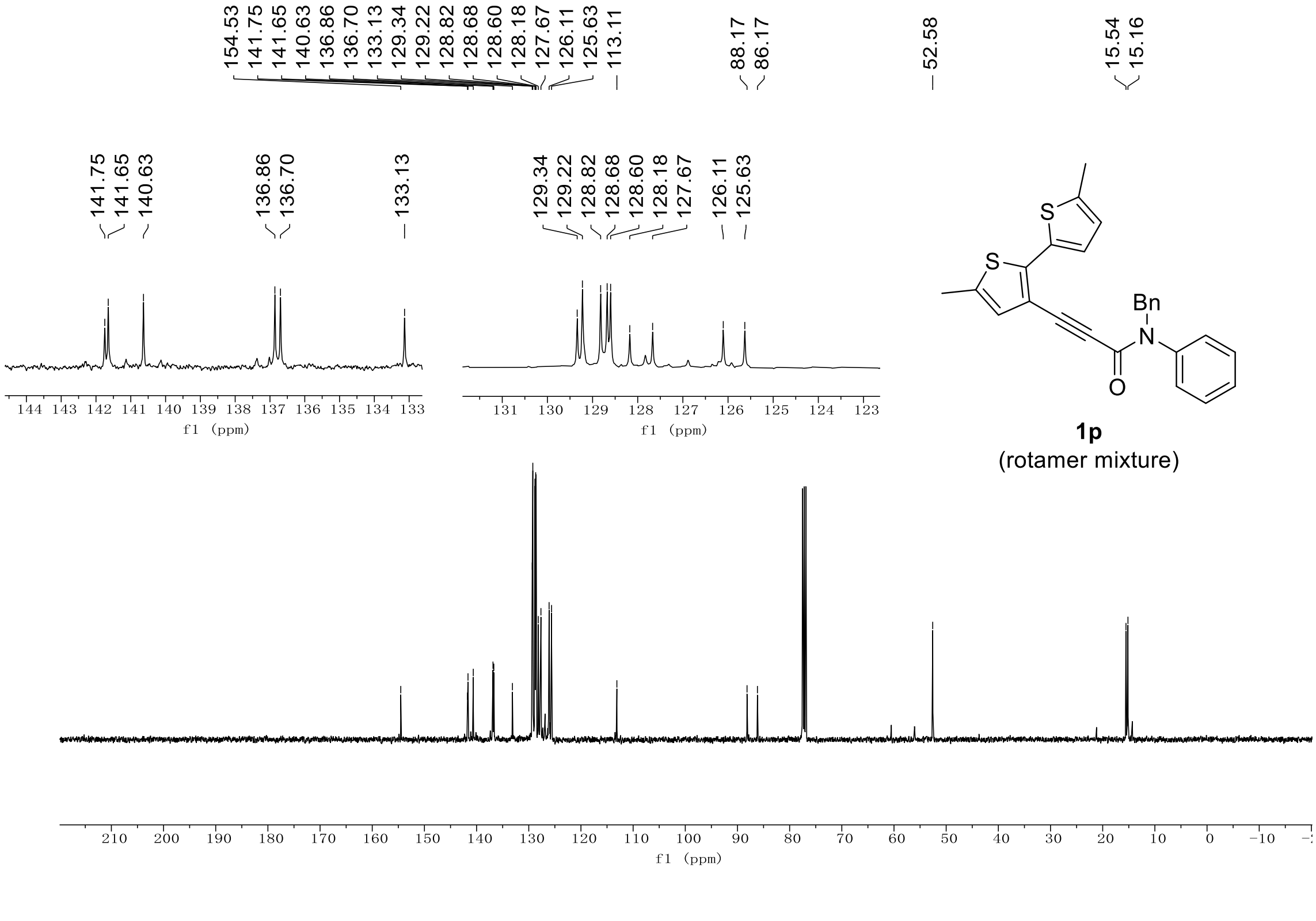

1p
(rotamer mixture)

^{1}H NMR (400 MHz, $CDCl_3$)

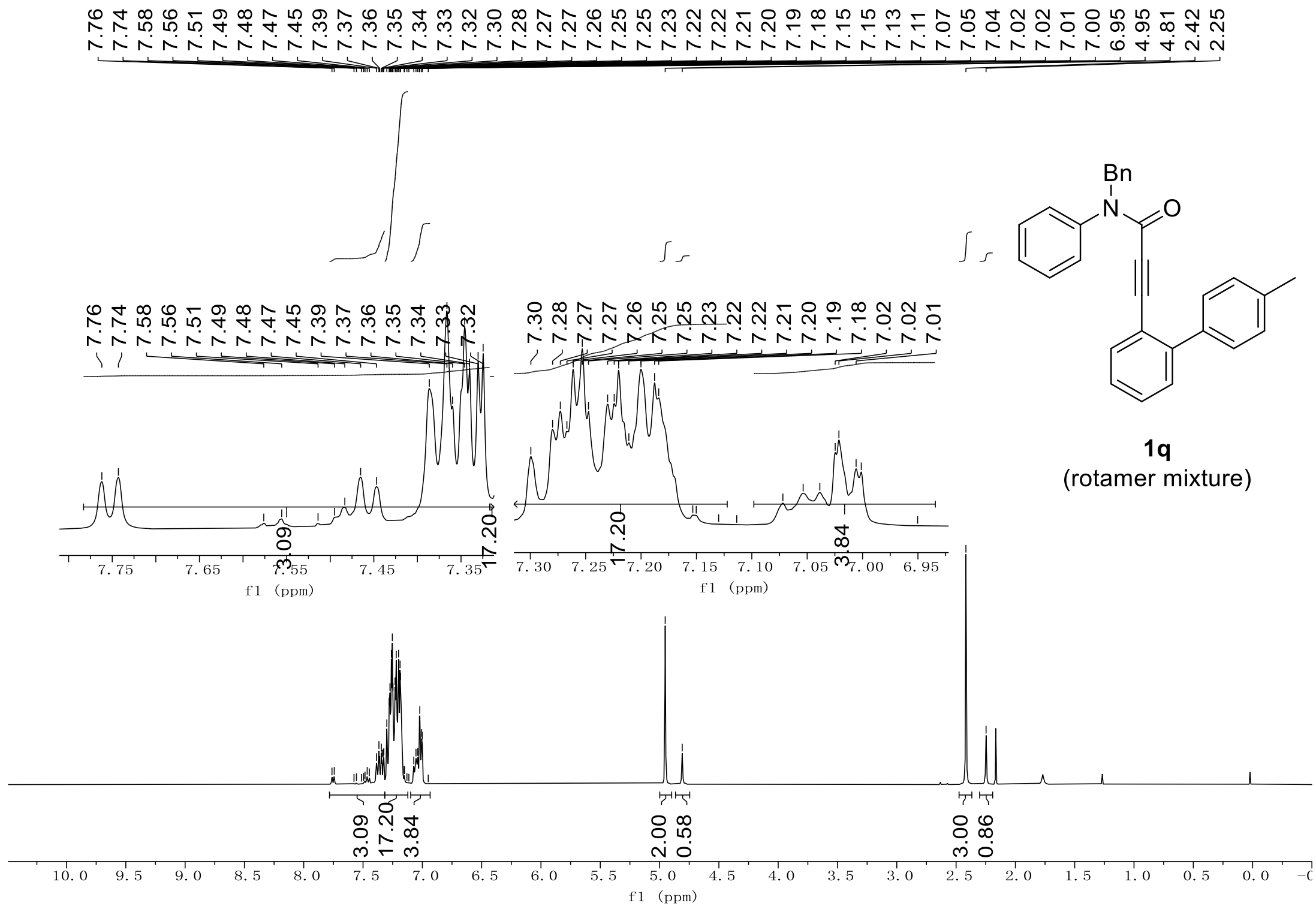

^{13}C NMR (100 MHz, $CDCl_3$) (Only signals corresponding to the major rotamer are reported)

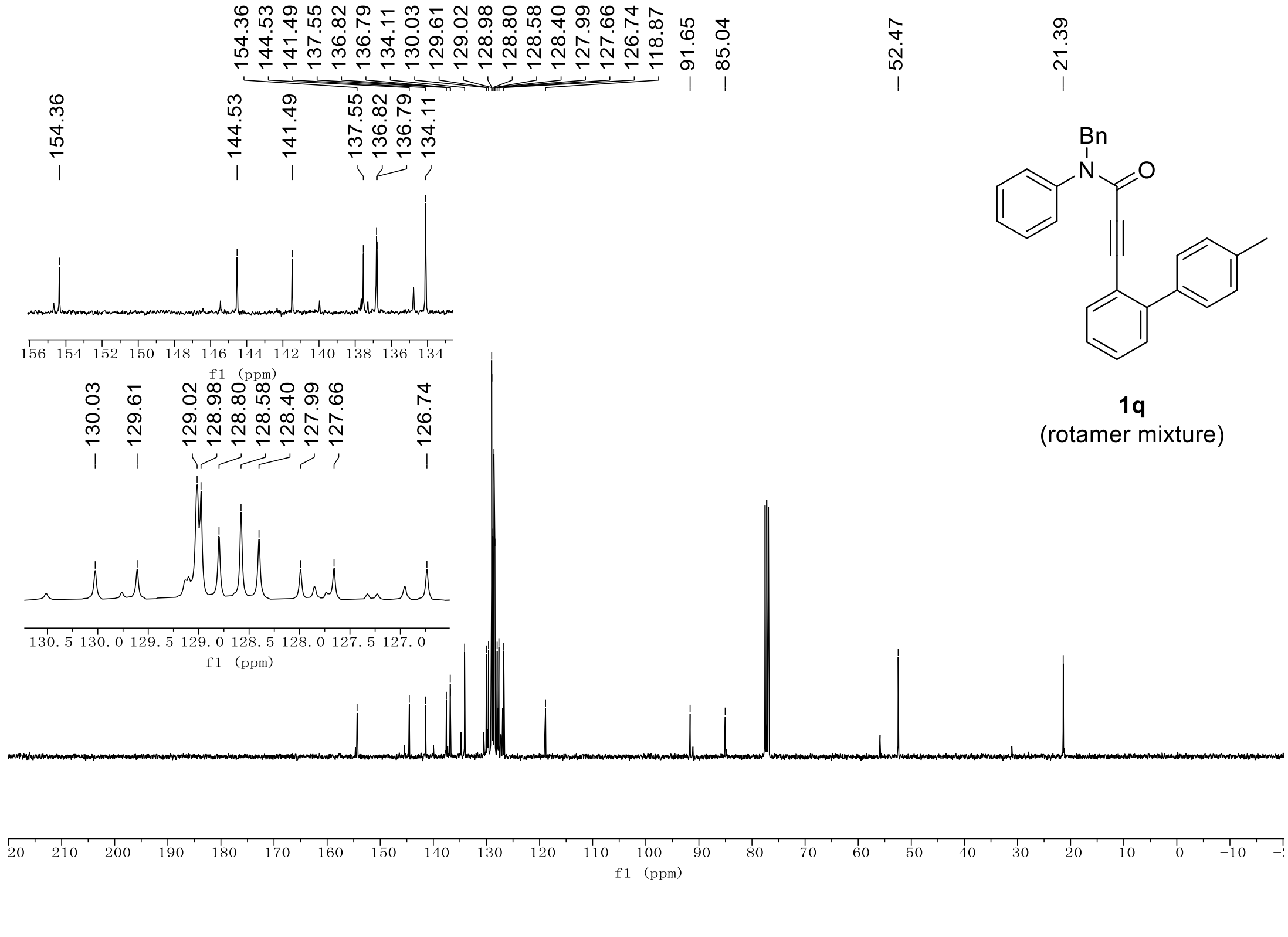

^{1}H NMR (400 MHz, $CDCl_3$)

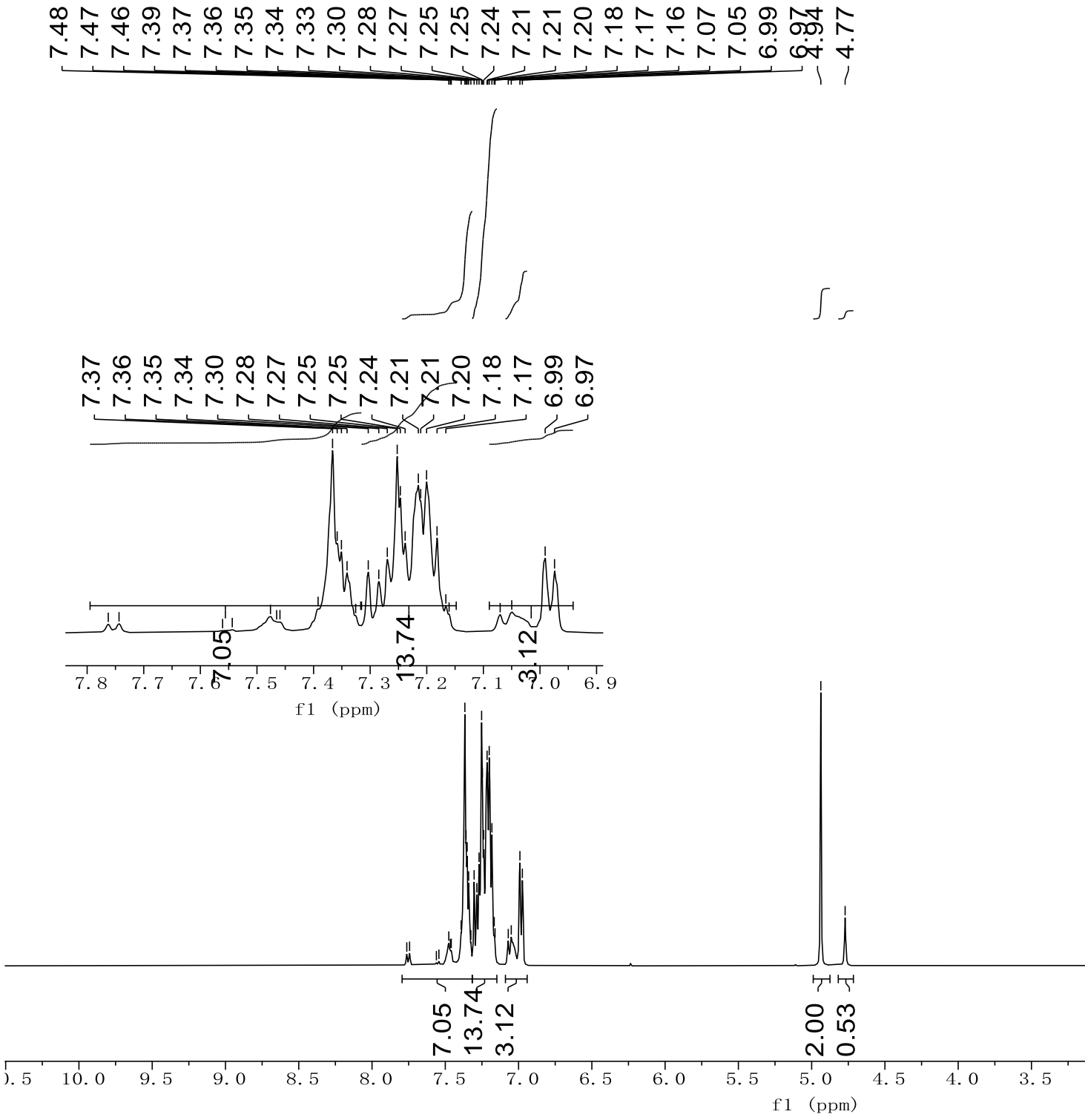

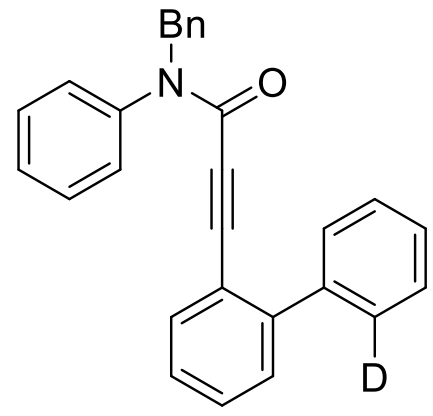

1a-*d*
(rotamer mixture)

^{13}C NMR (100 MHz, $CDCl_3$9 (Only signals corresponding to the major rotamer are reported)

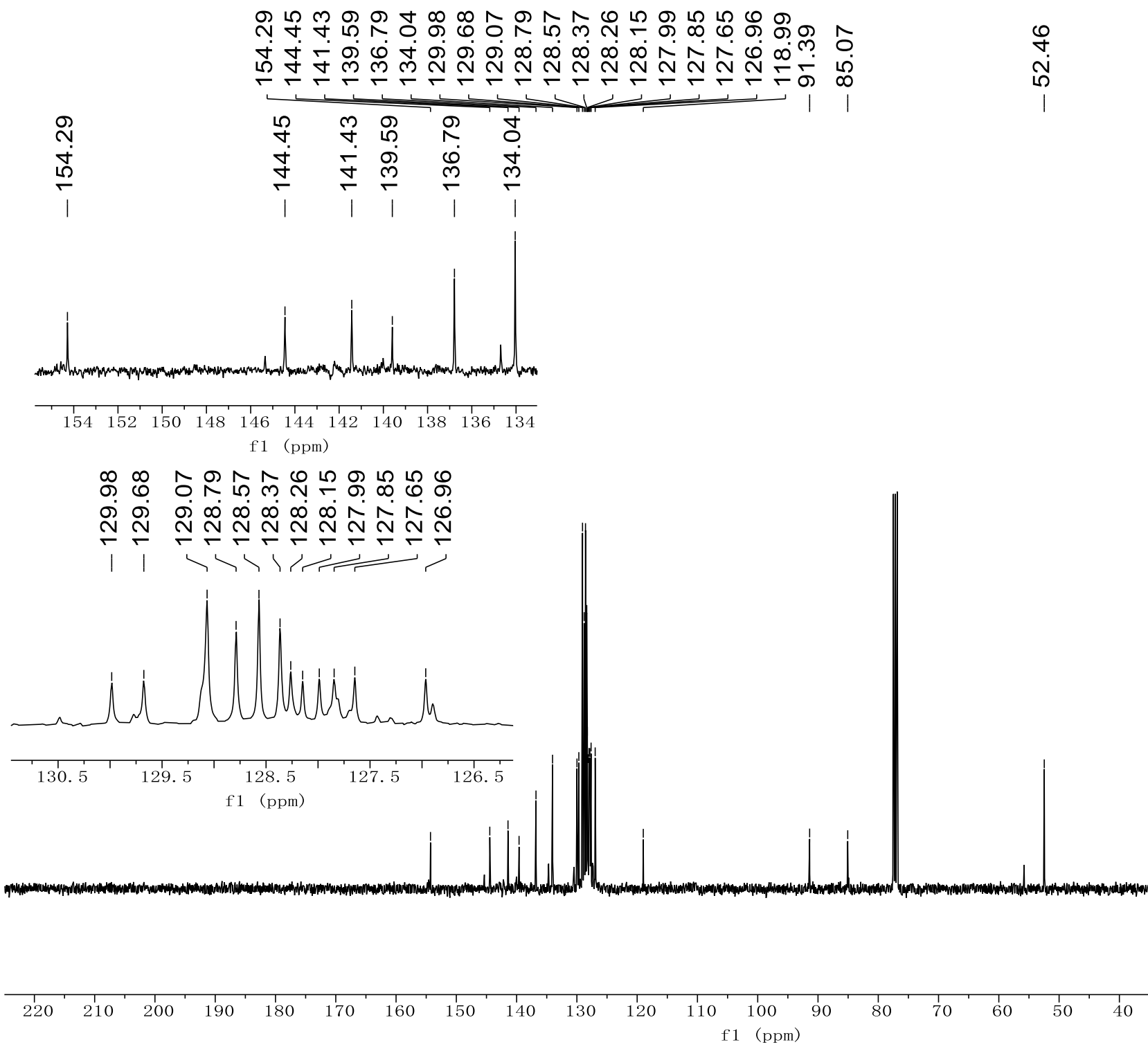

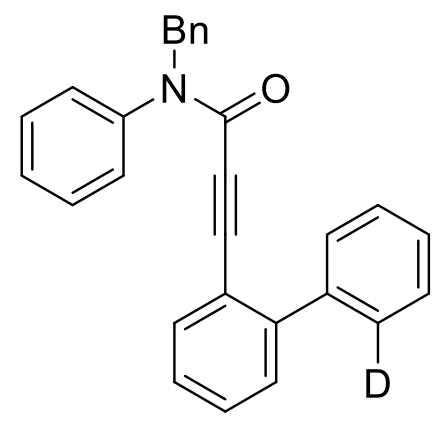

1a-*d*
(rotamer mixture)

^{1}H NMR (400 MHz, $CDCl_3$)

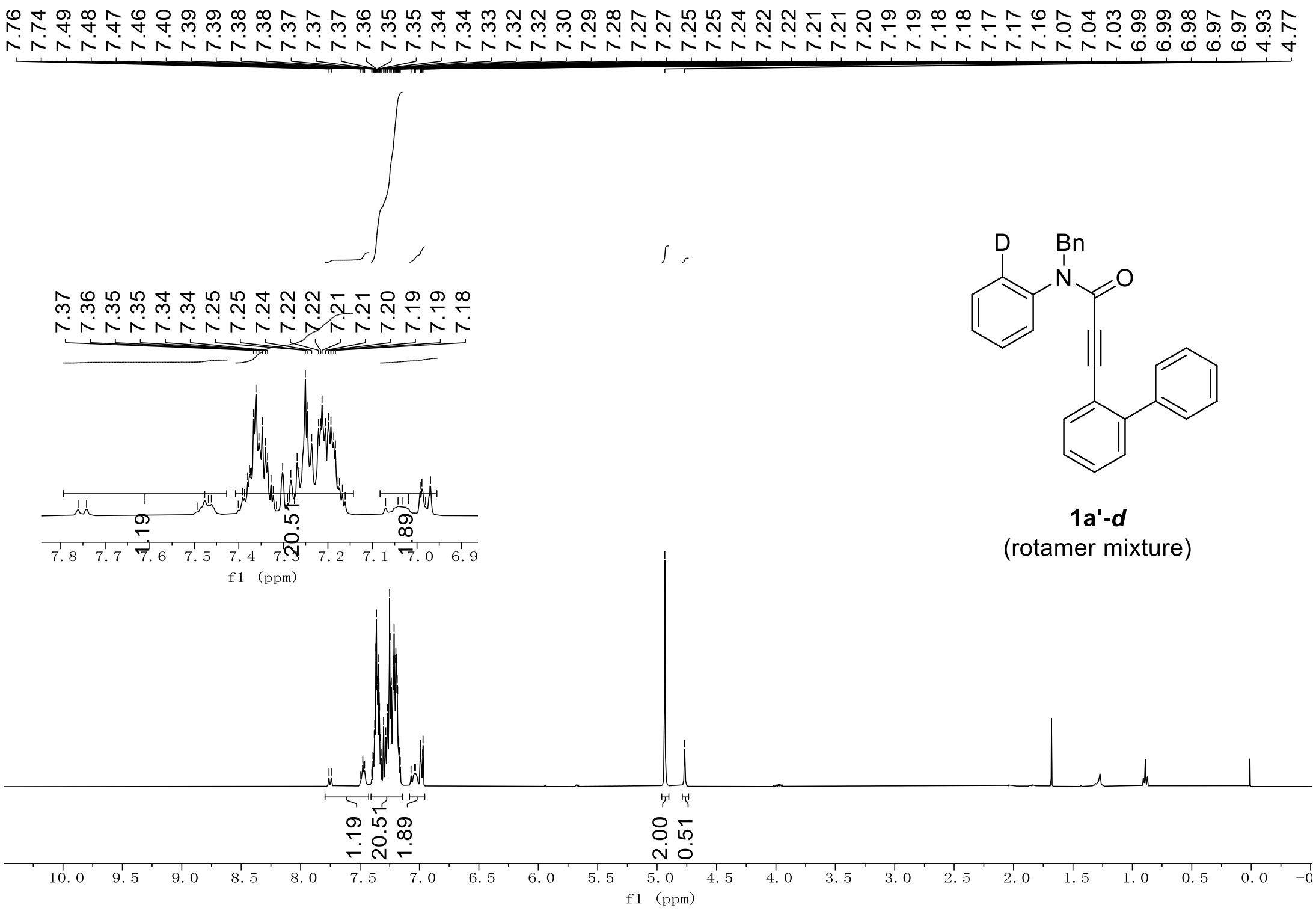

^{13}C NMR (100 MHz, $CDCl_3$) (Only signals corresponding to the major rotamer are reported)

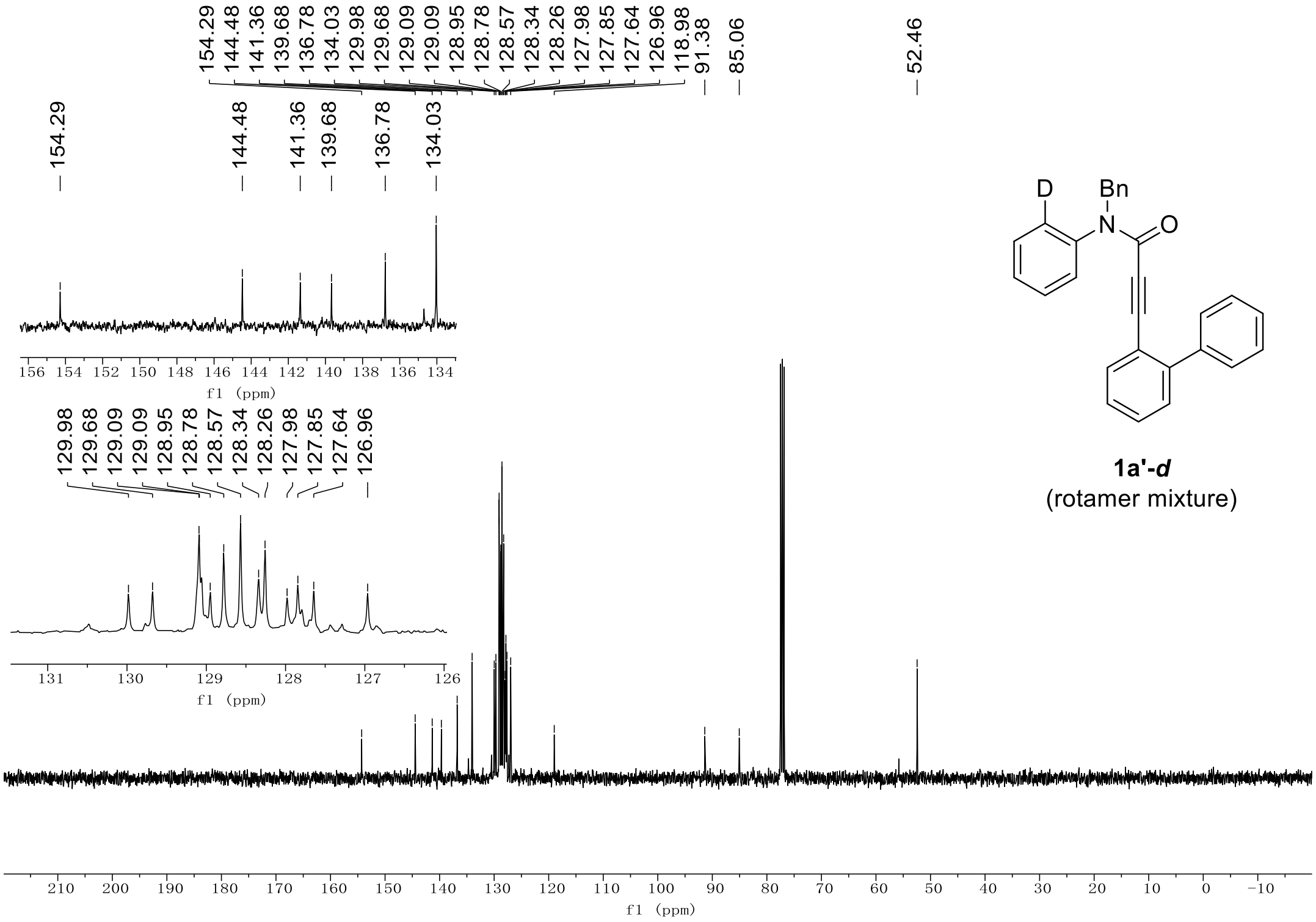

^1H NMR (400 MHz, $CDCl_3$)

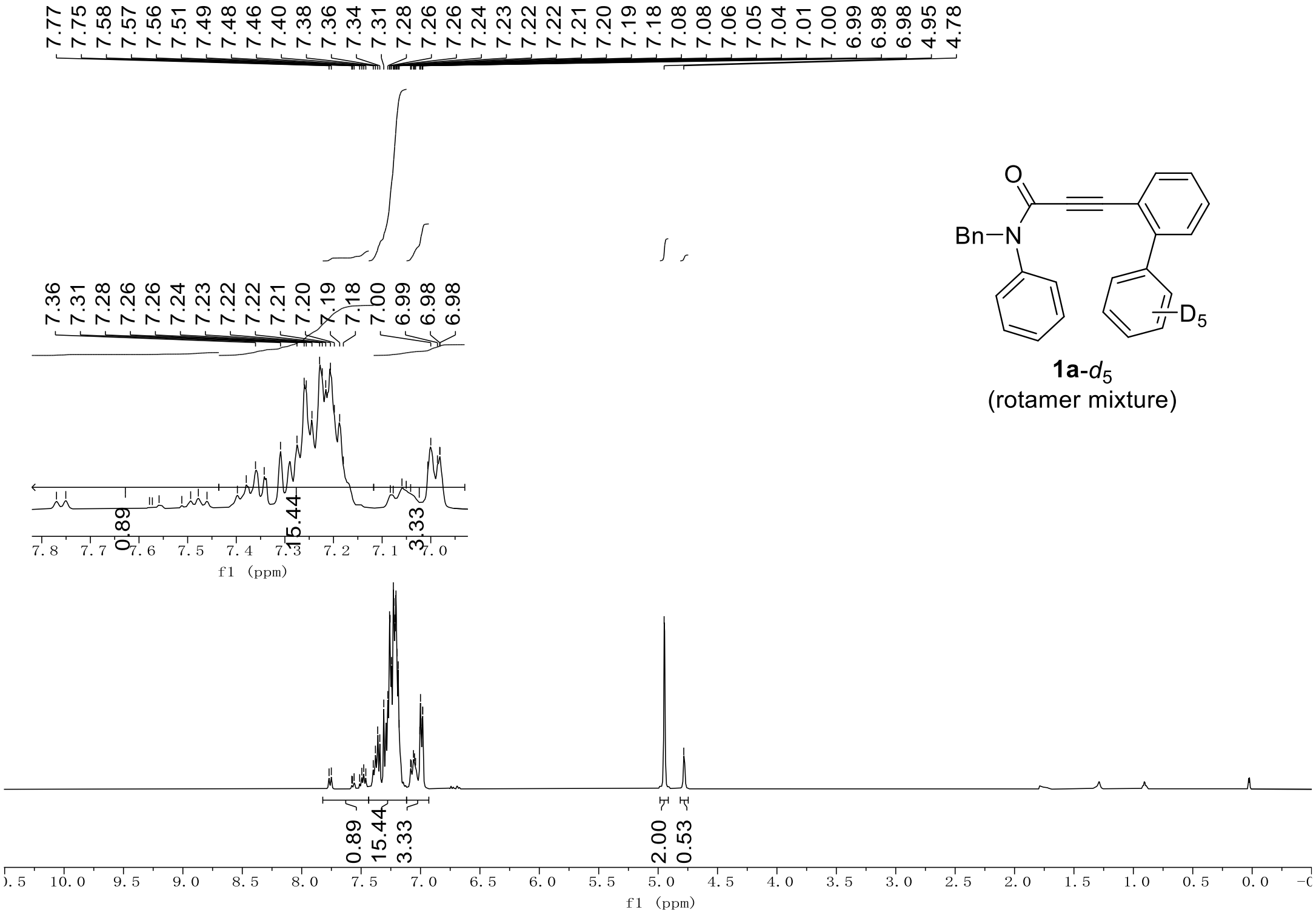

^{13}C NMR (100 MHz, $CDCl_3$) (Only signals corresponding to the major rotamer are reported)

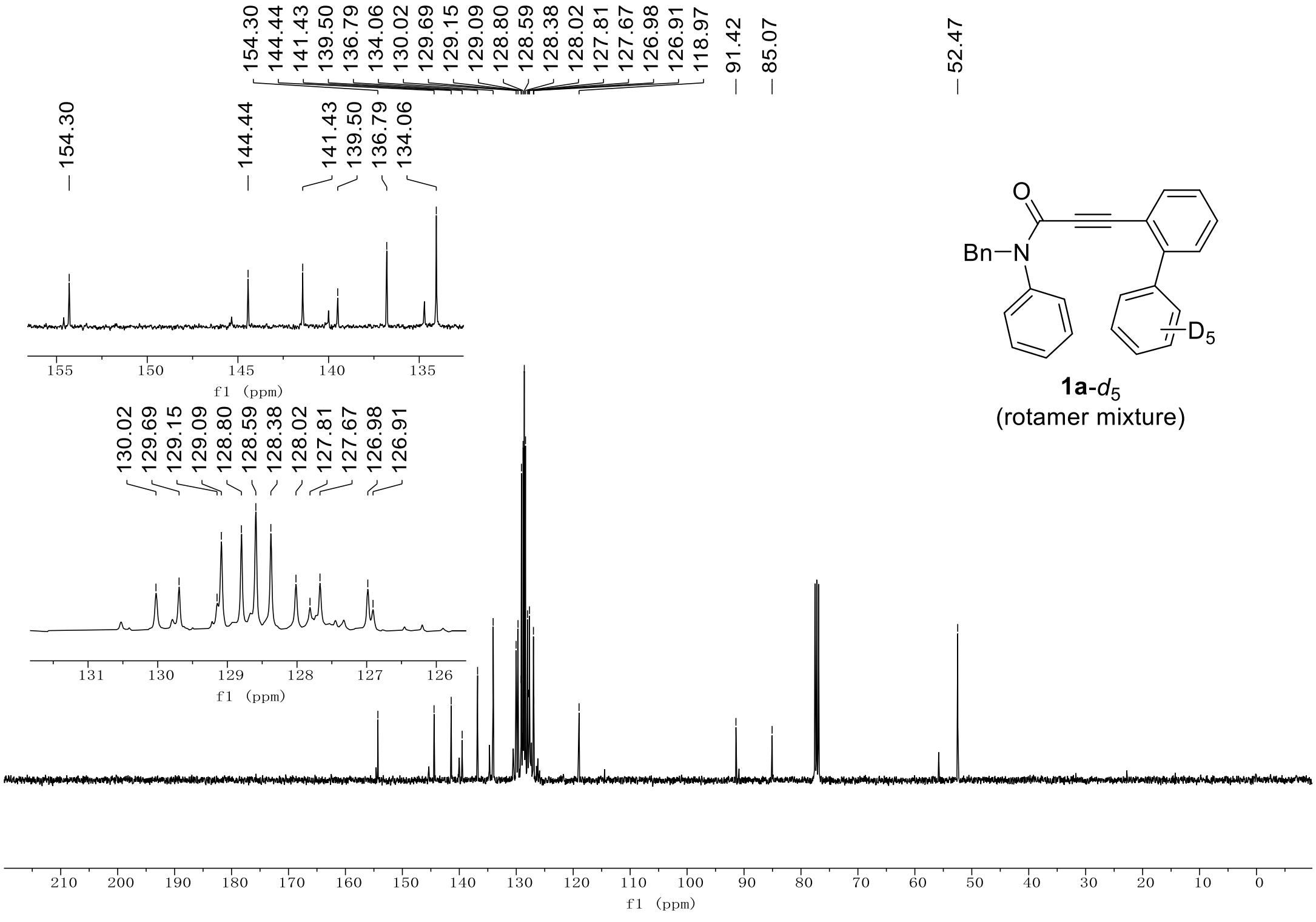

^{1}H NMR (400 MHz, $CDCl_3$)

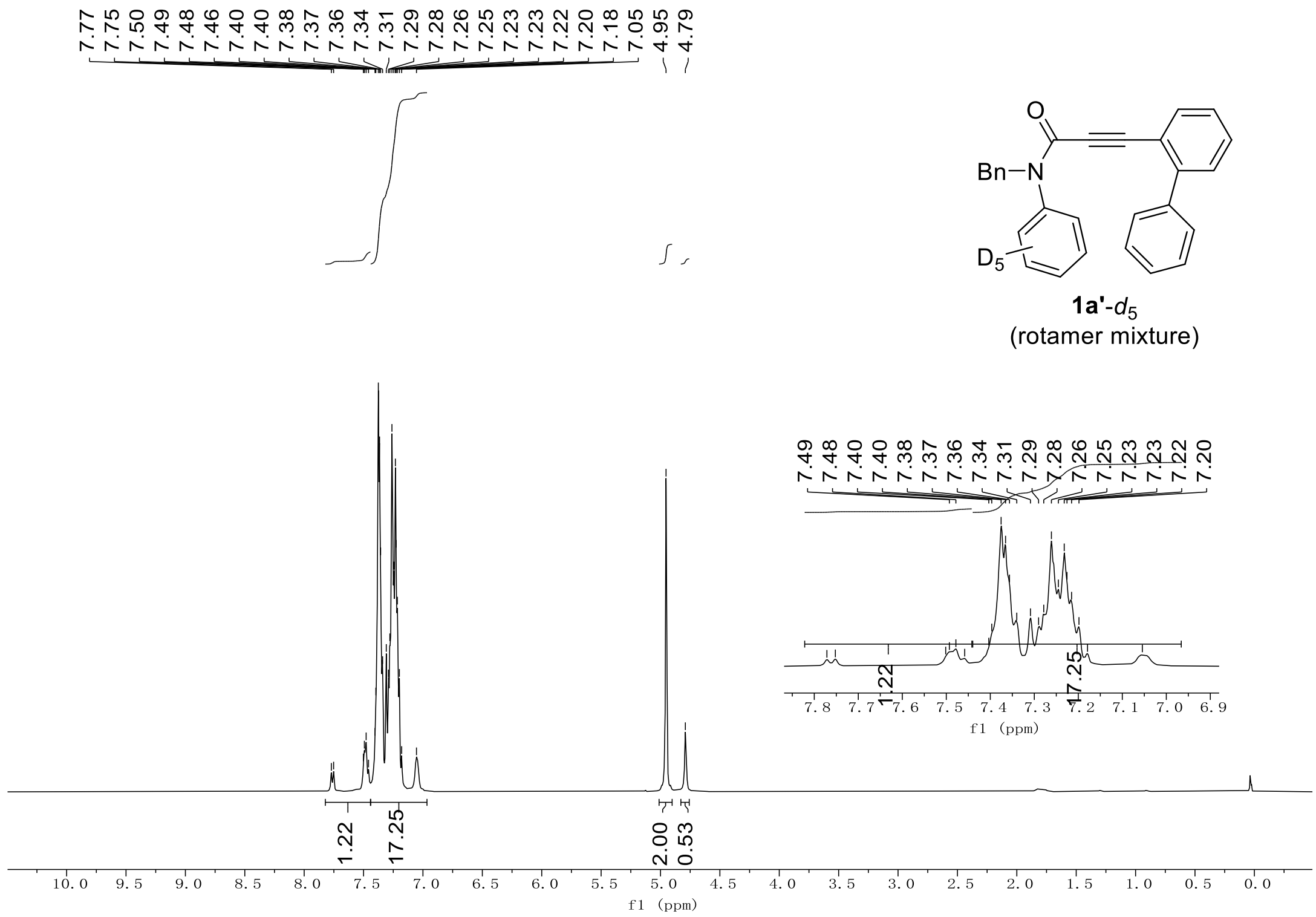

1a'-*d*$_5$
(rotamer mixture)

^{13}C NMR (100 MHz, $CDCl_3$) (Only signals corresponding to the major rotamer are reported)

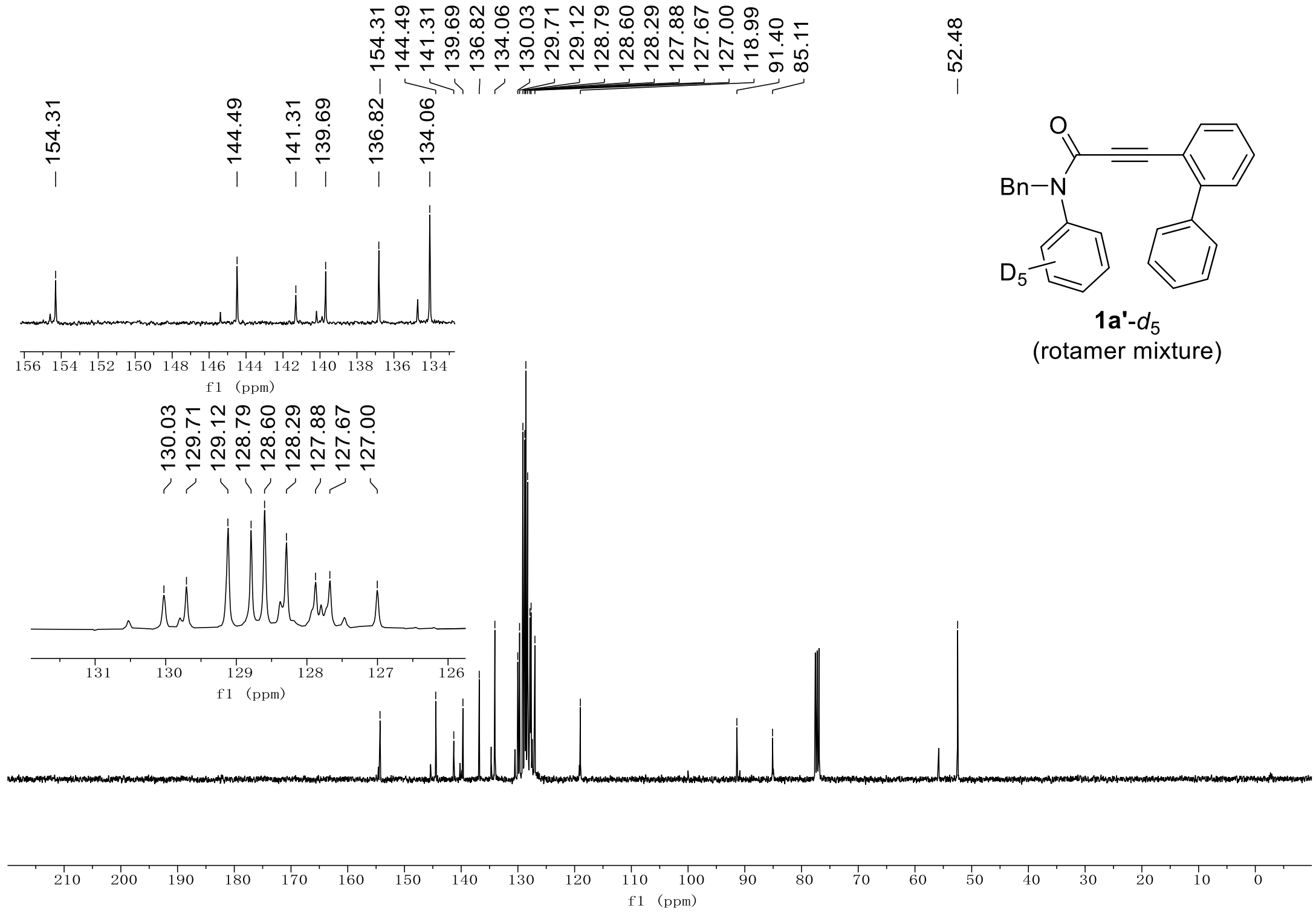

1a'-*d*$_5$
(rotamer mixture)

^{1}H NMR (400 MHz, $CDCl_3$)

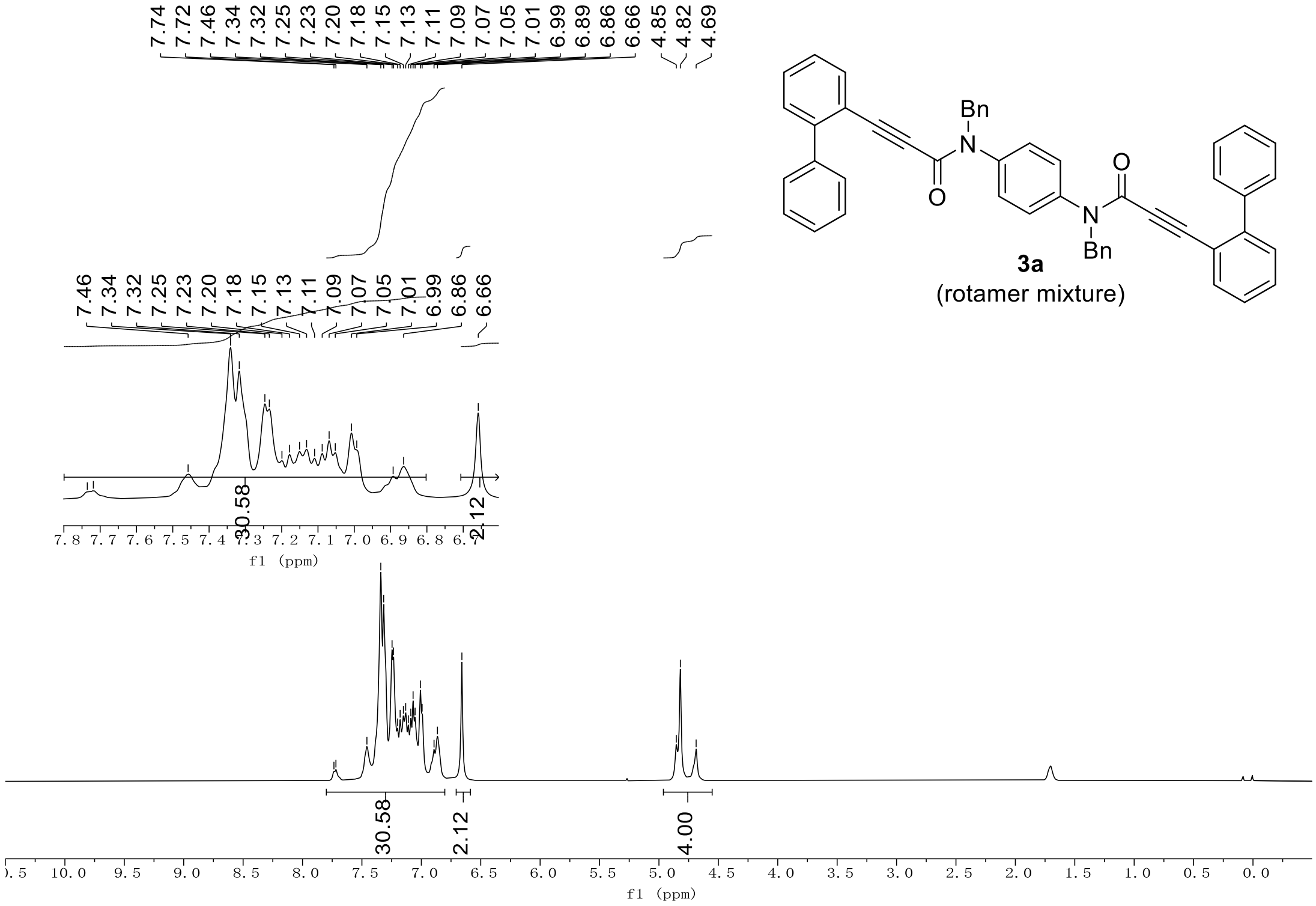

3a
(rotamer mixture)

^{13}C NMR (100 MHz, $CDCl_3$) (Only signals corresponding to the major rotamer are reported)

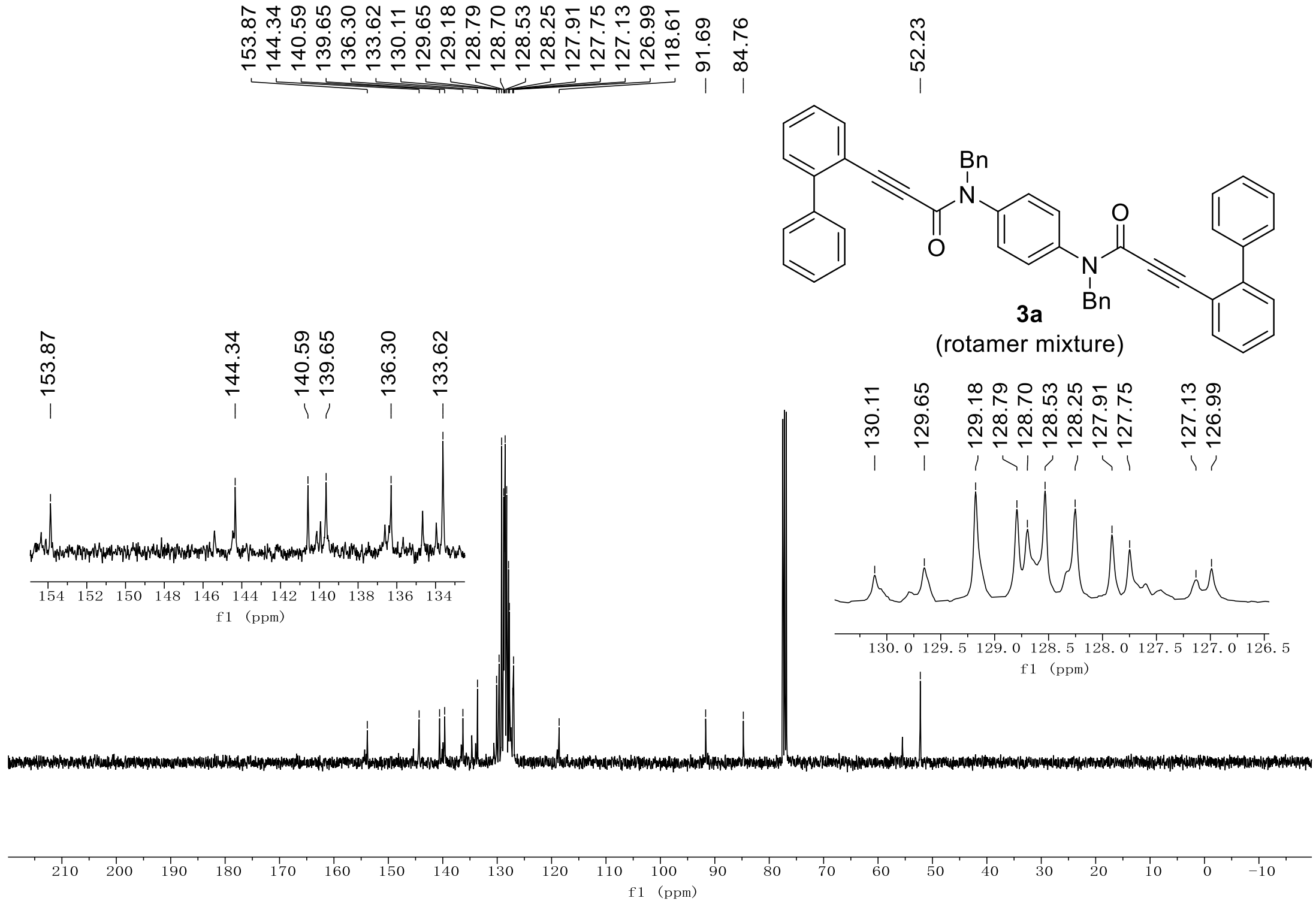

3a
(rotamer mixture)

^{1}H NMR (400 MHz, $CDCl_3$)

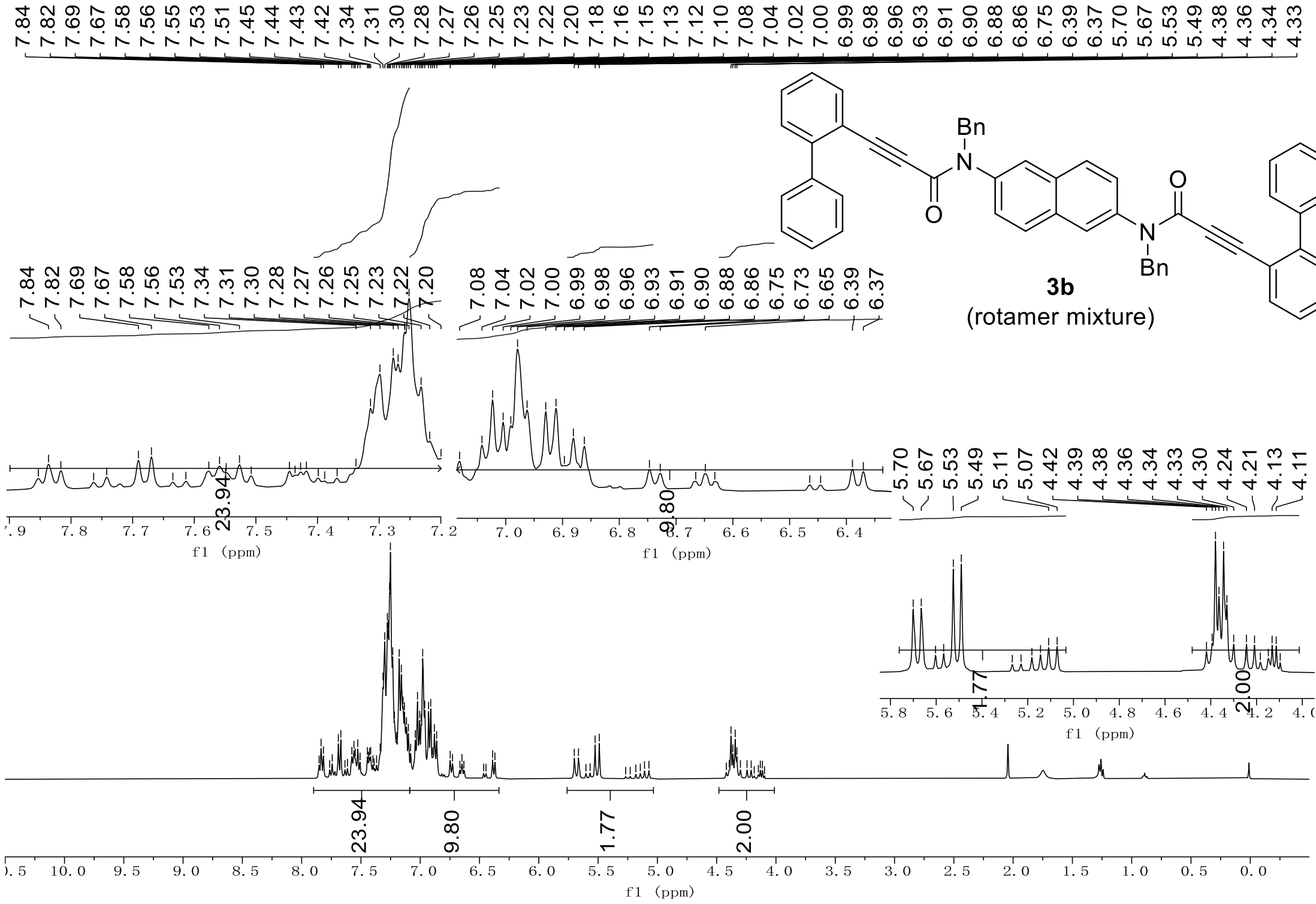

^{13}C NMR (100 MHz, $CDCl_3$) (Only signals corresponding to the major rotamer are reported)

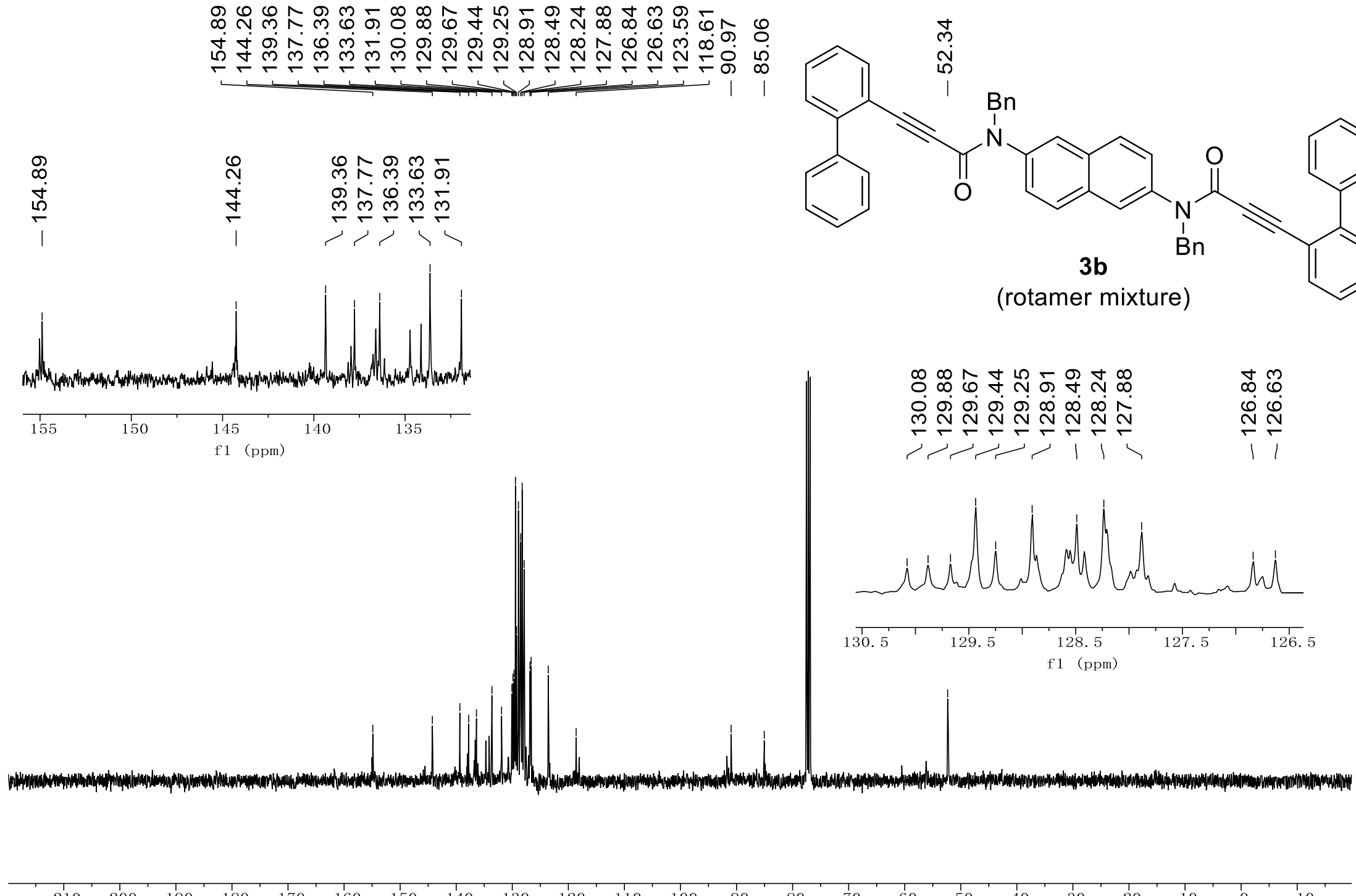

^{1}H NMR (400 MHz, $CDCl_3$)

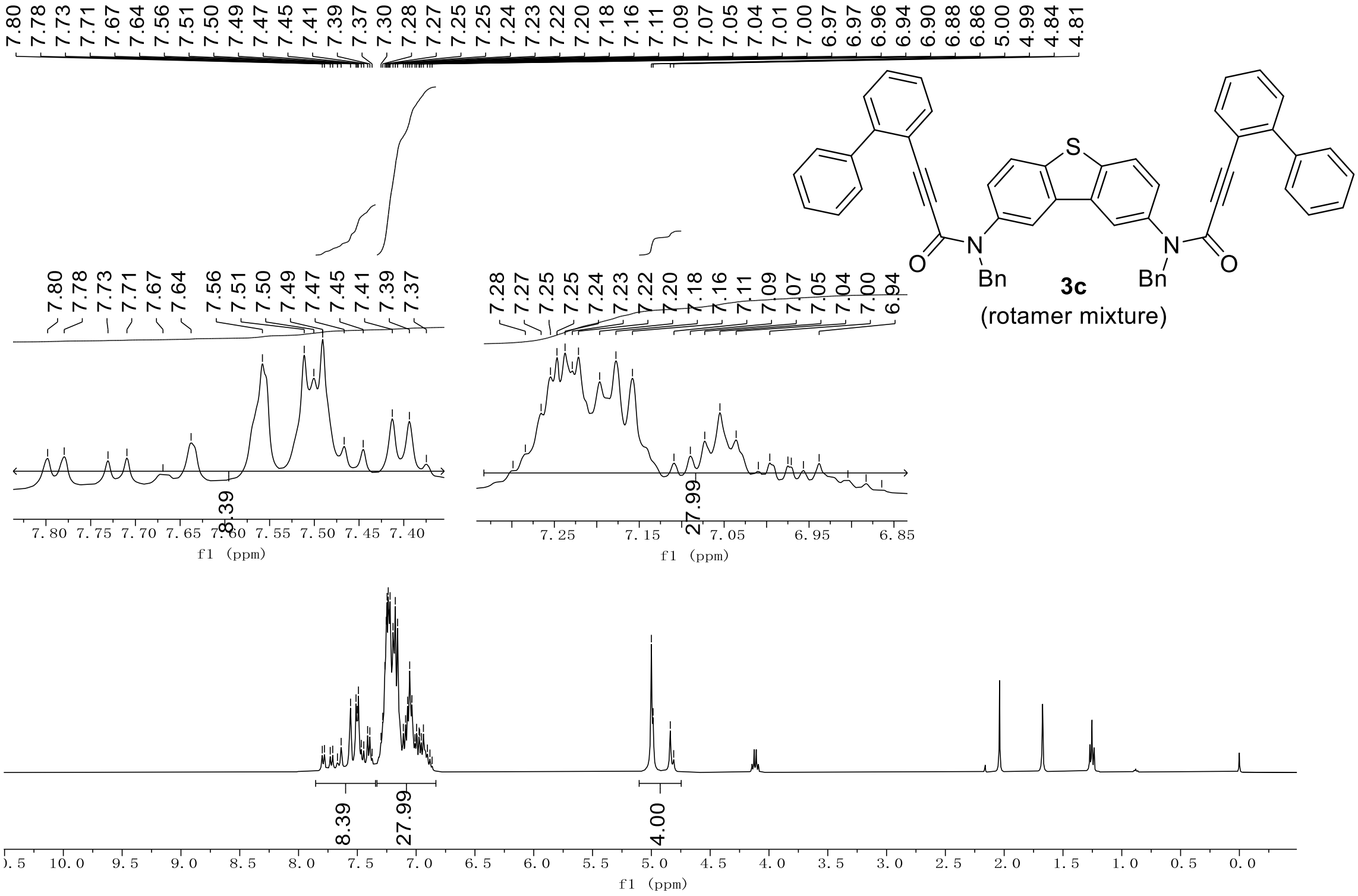

^{13}C NMR (100 MHz, $CDCl_3$) (Only signals corresponding to the major rotamer are reported)

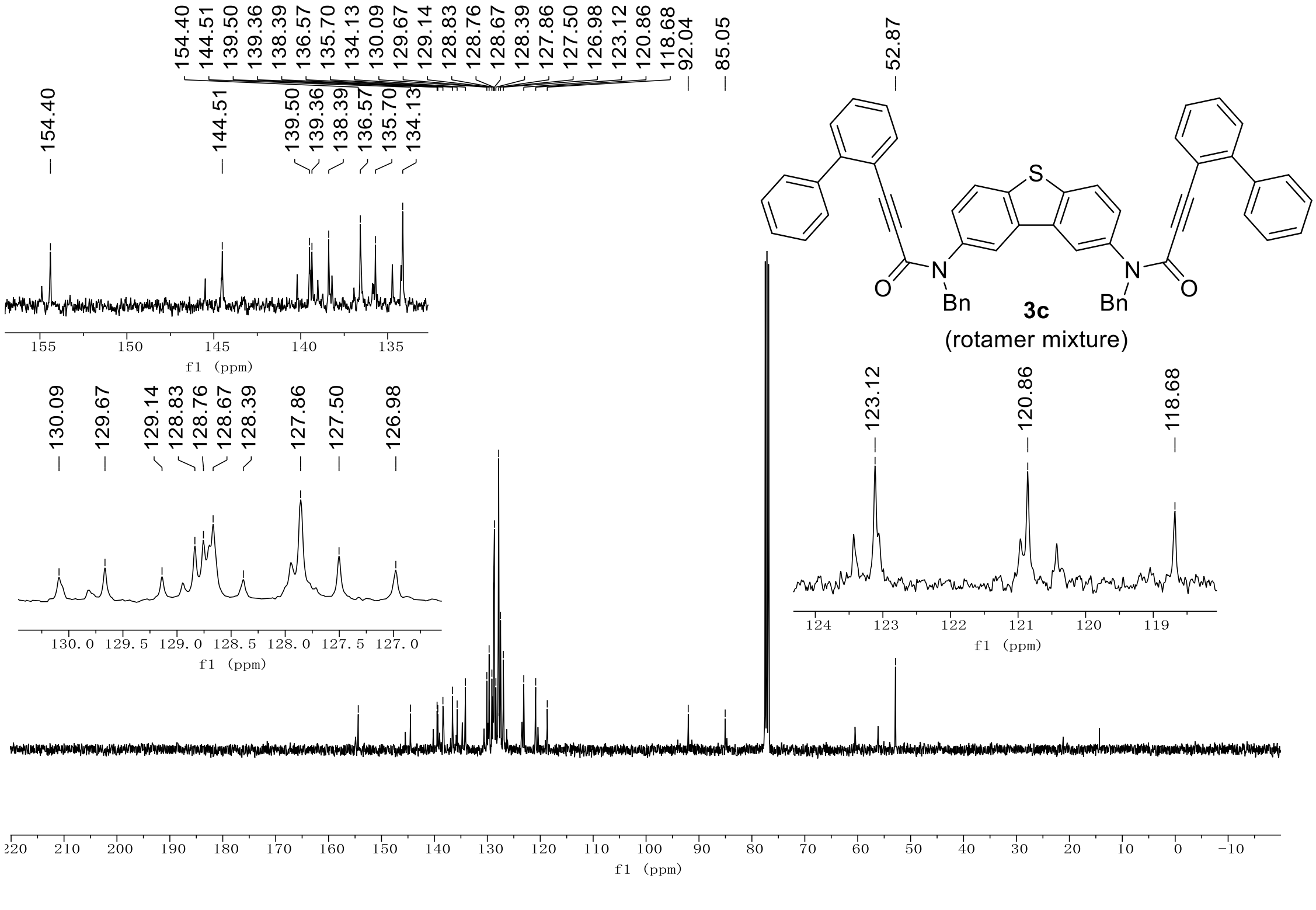

^{1}H NMR (400 MHz, $CDCl_3$)

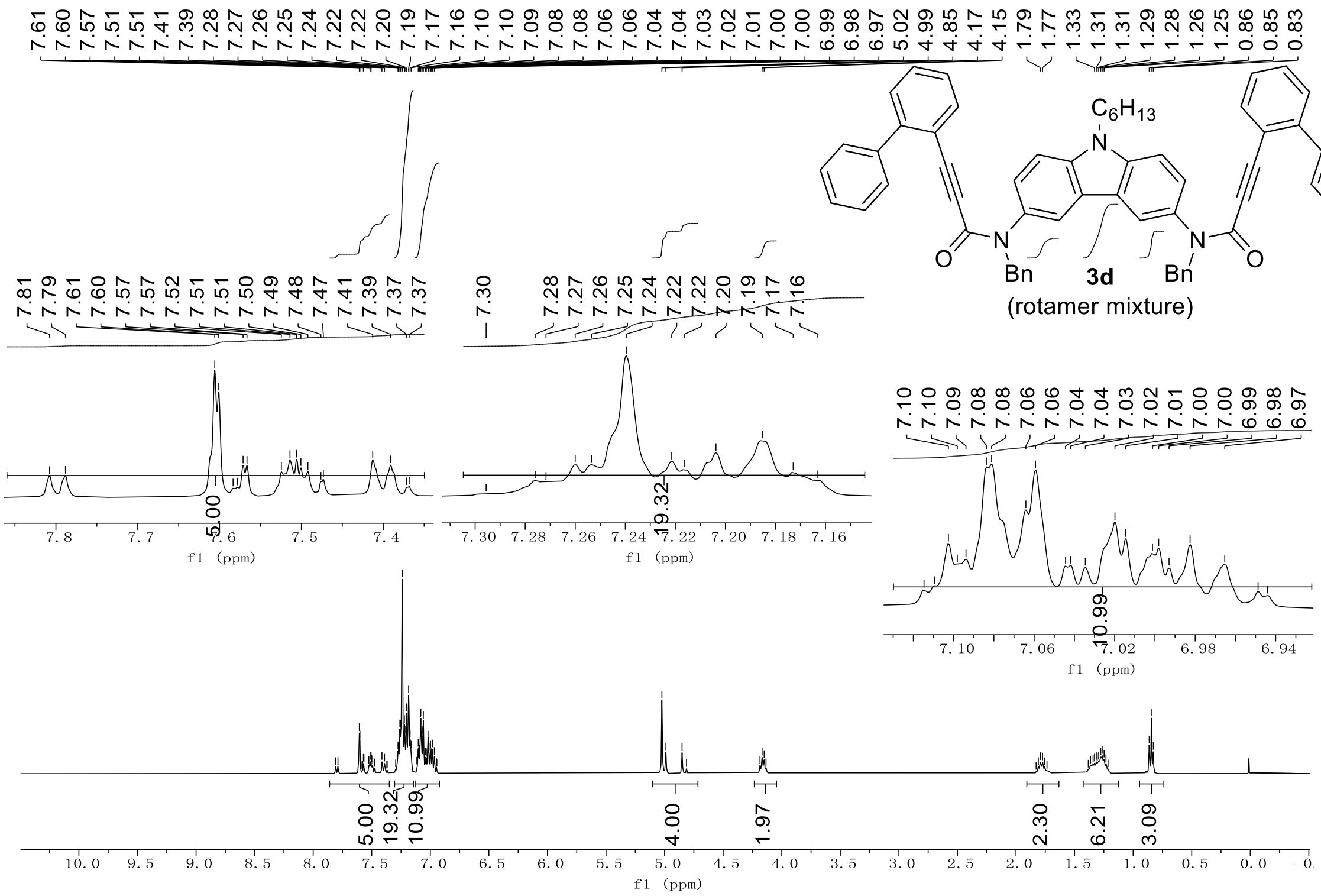

^{13}C NMR (100 MHz, $CDCl_3$) (Only signals corresponding to the major rotamer are reported)

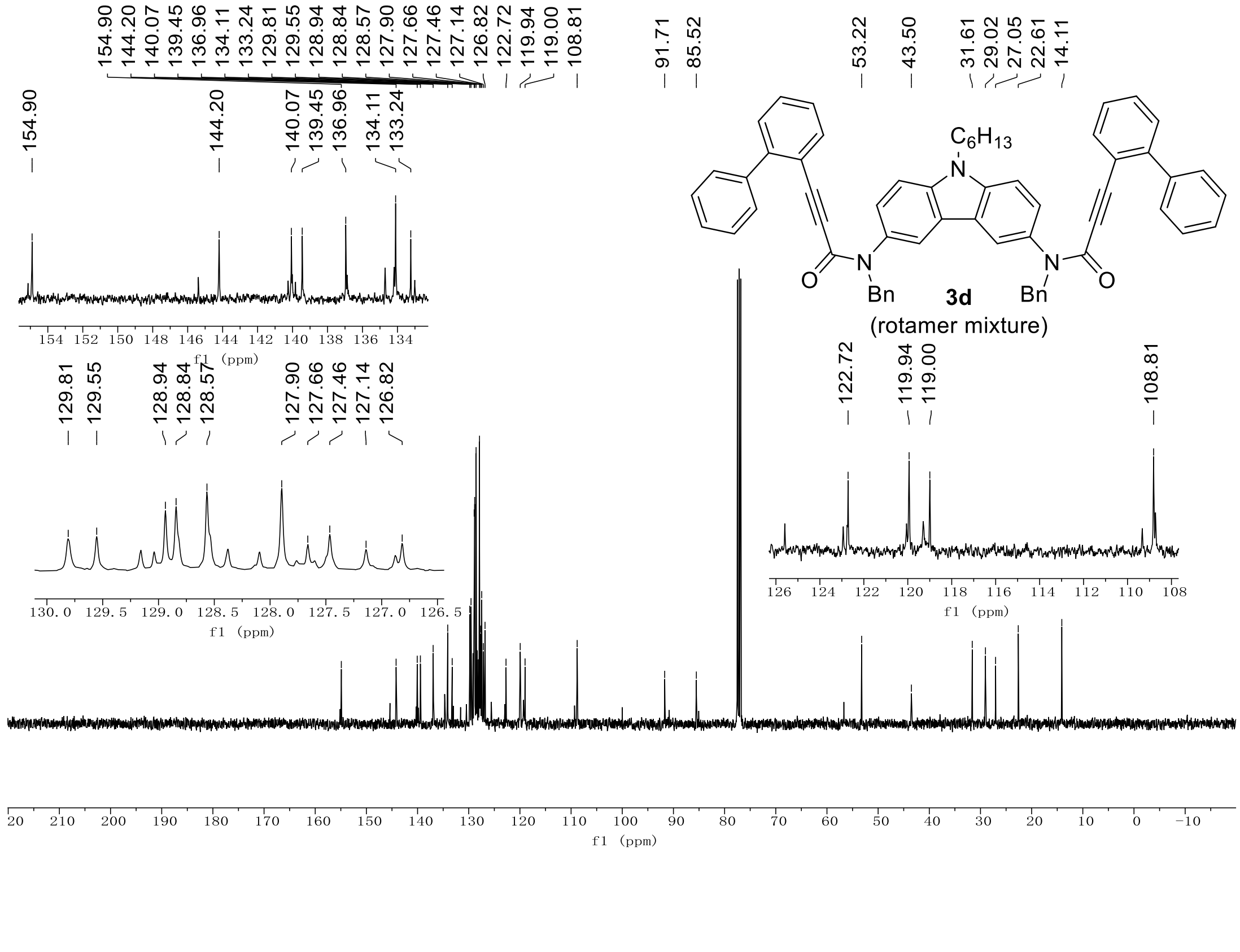

^1H NMR (400 MHz, $CDCl_3$)

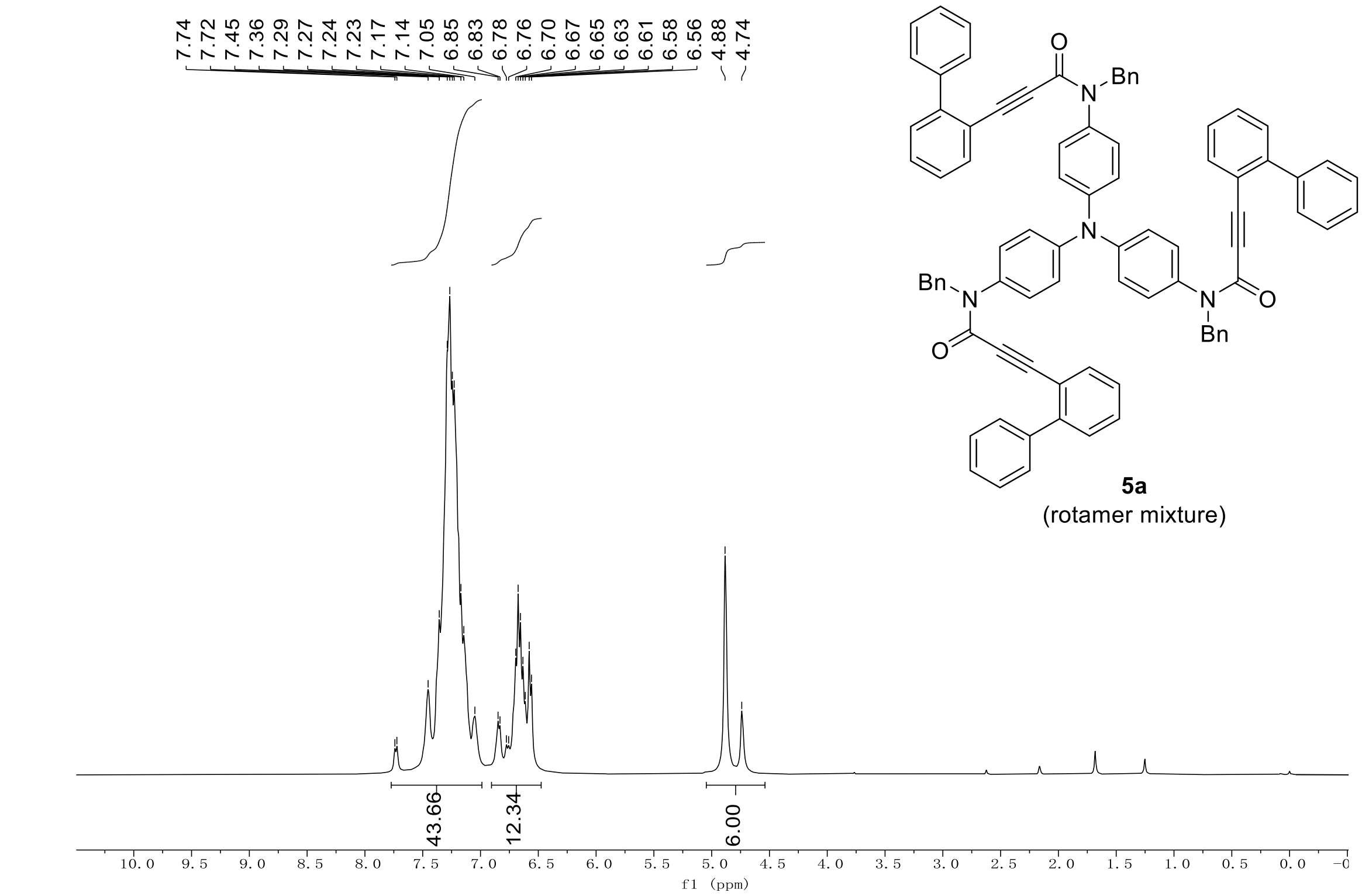

^{13}C NMR (100 MHz, $CDCl_3$) (Only signals corresponding to the major rotamer are reported)

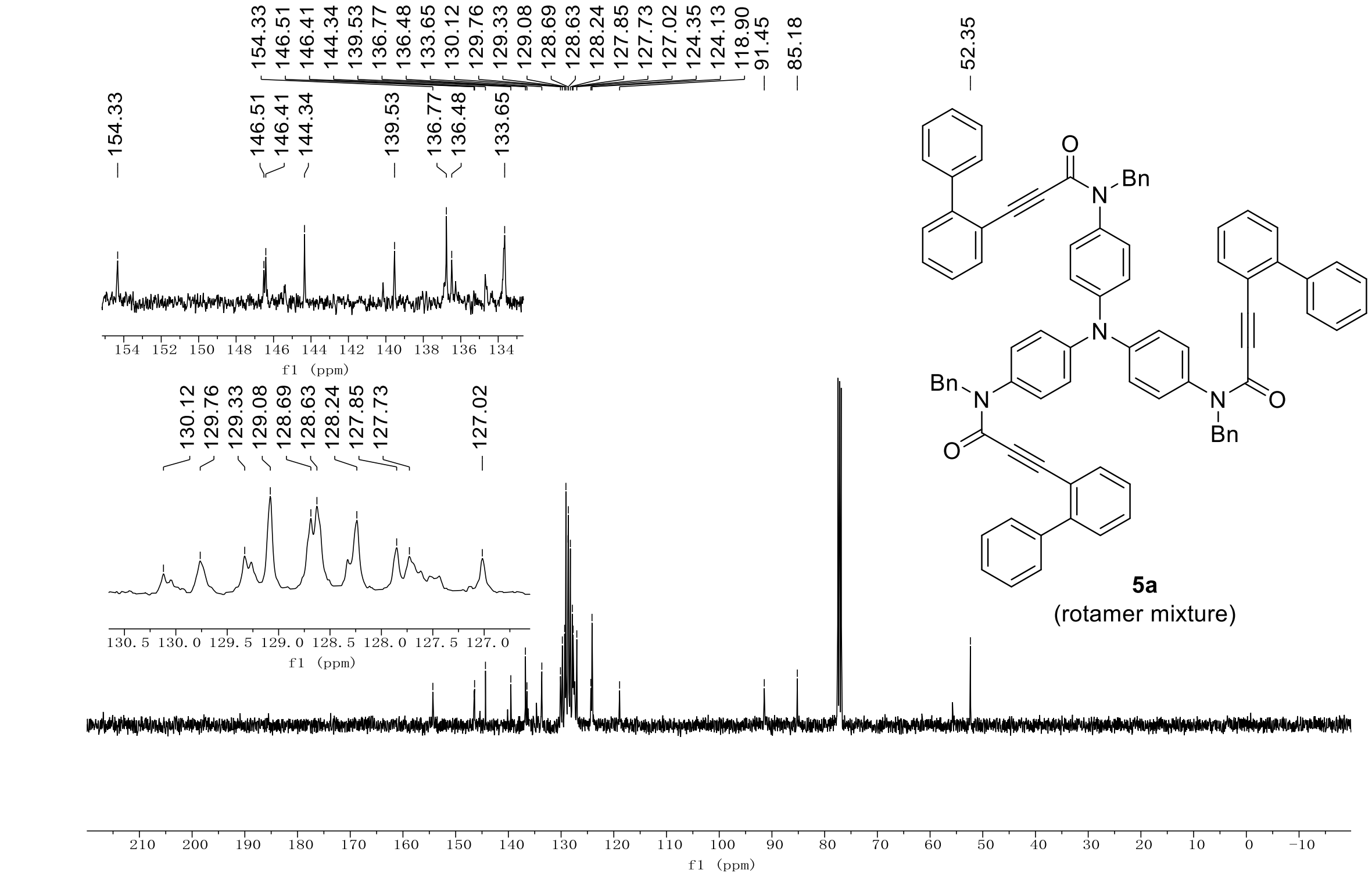

^{1}H NMR (400 MHz, $CDCl_3$)

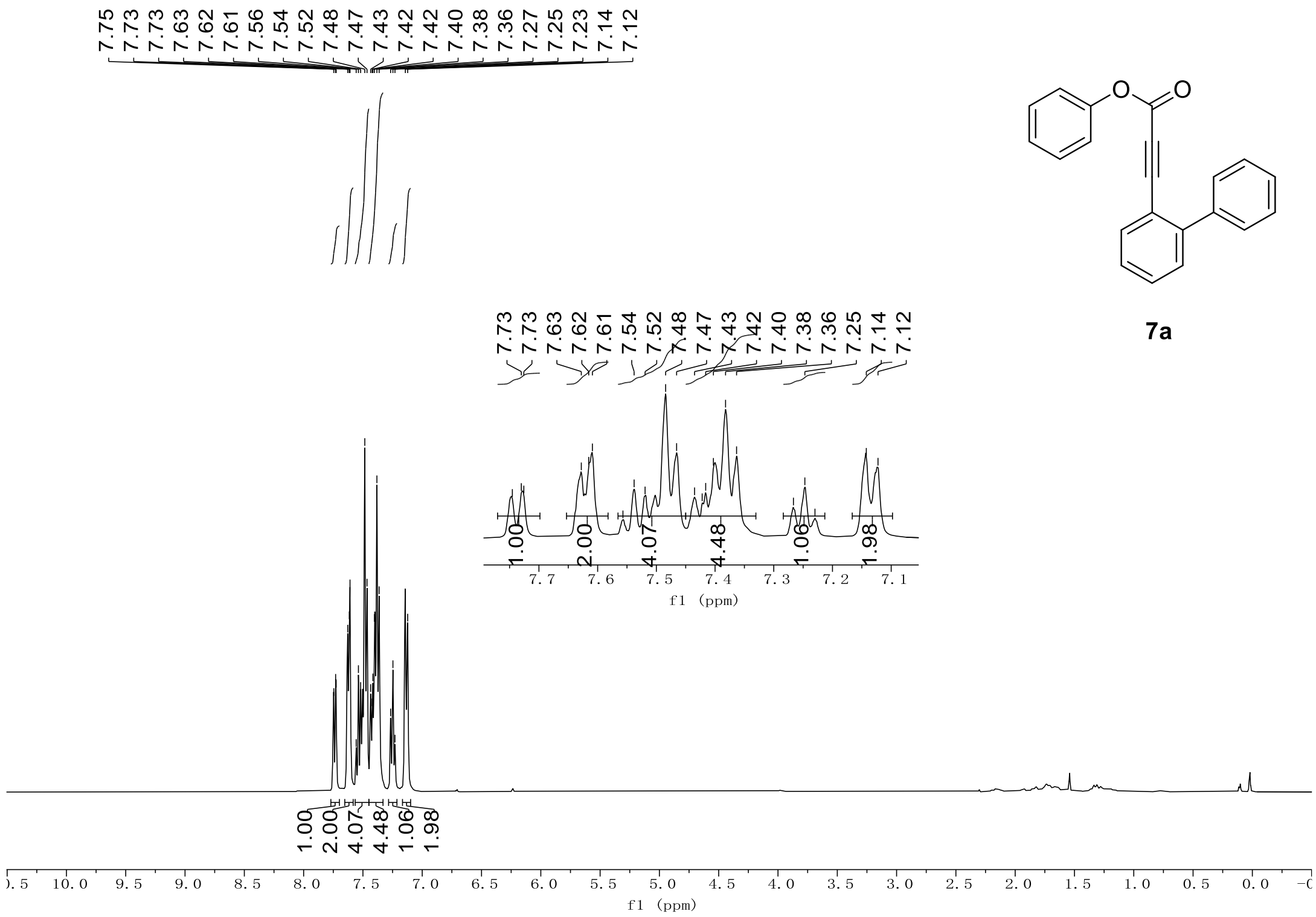

7a

^{13}C NMR (100 MHz, $CDCl_3$)

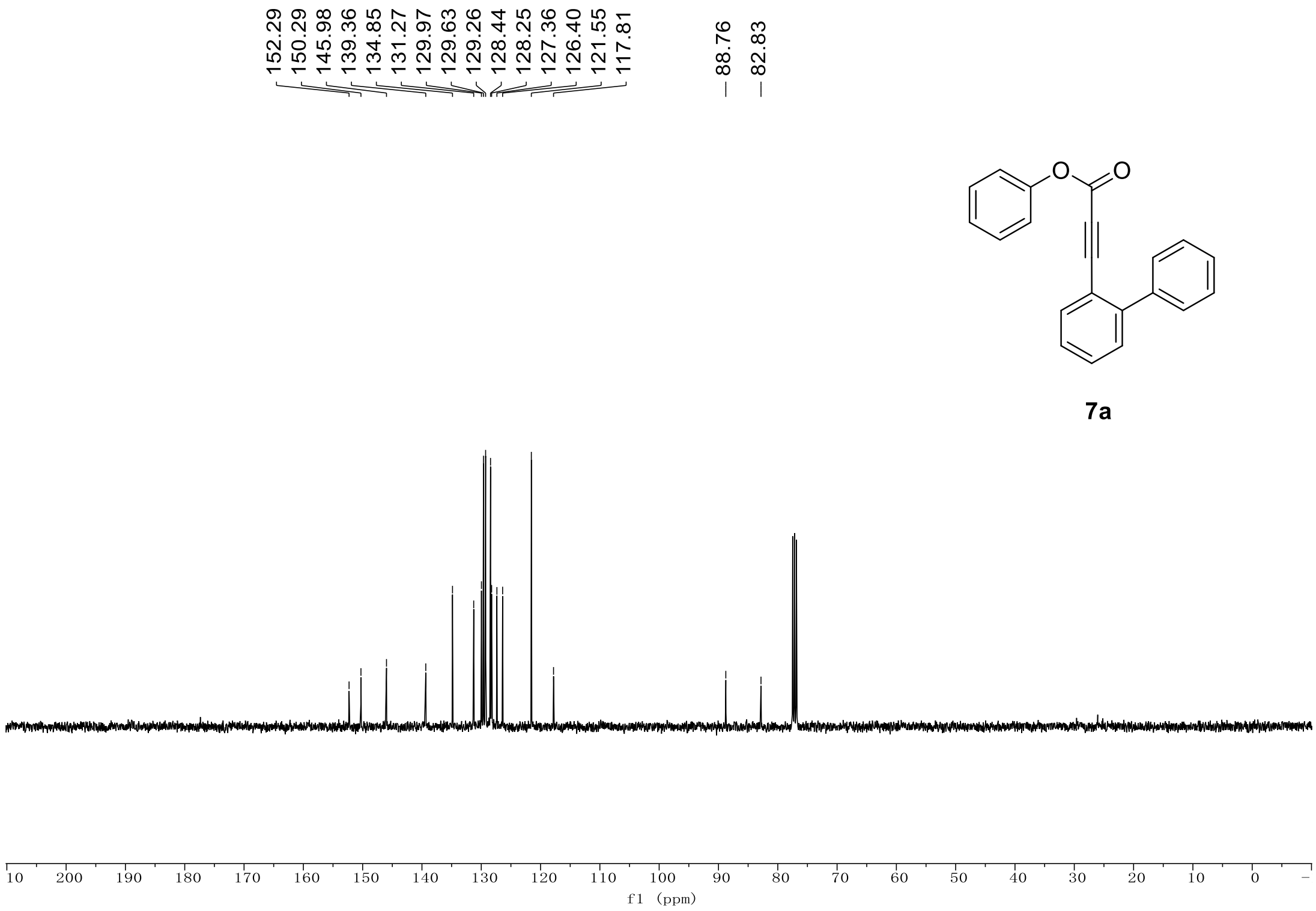

7a

^{1}H NMR (400 MHz, $CDCl_3$)

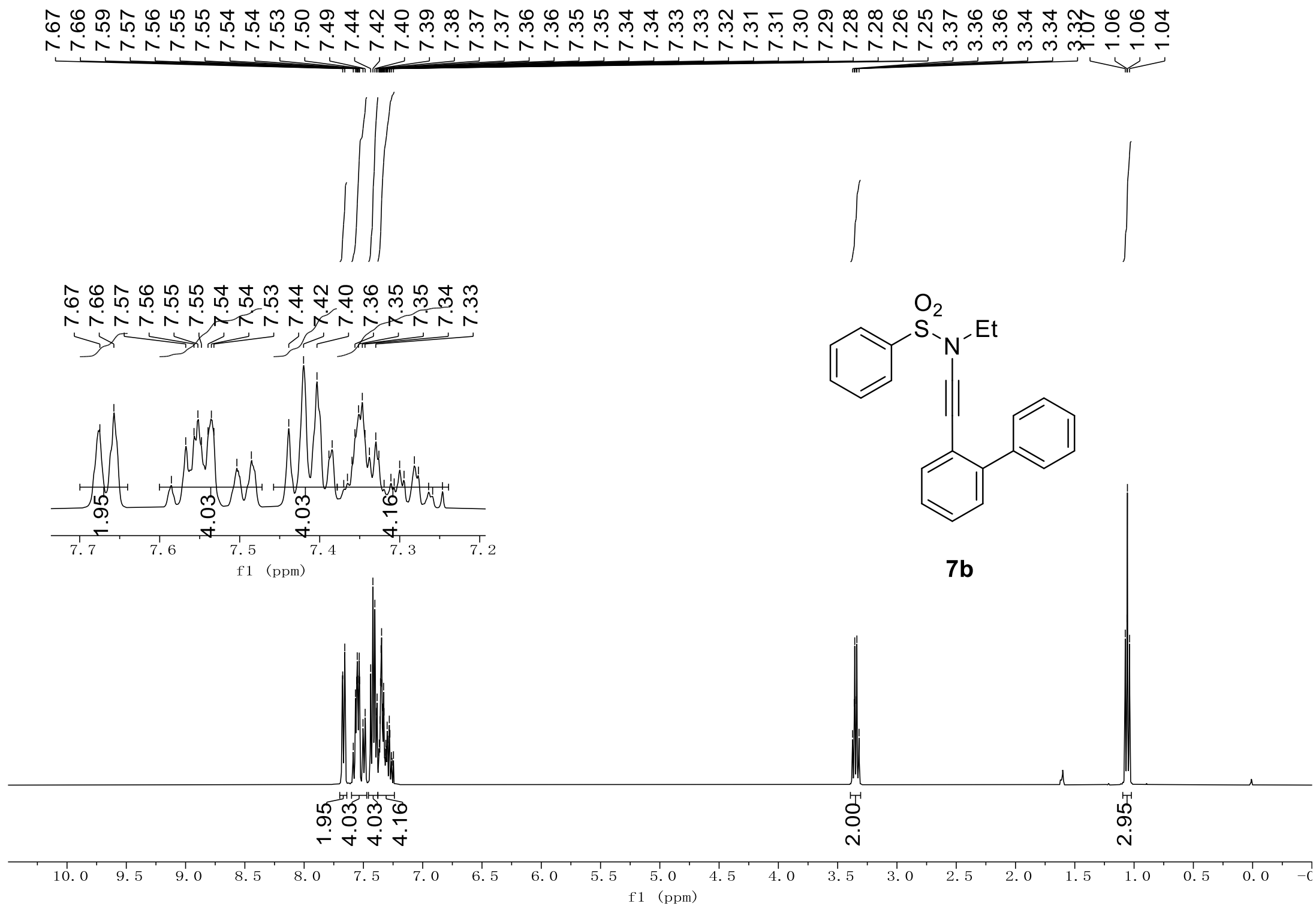

^{13}C NMR (100 MHz, $CDCl_3$)

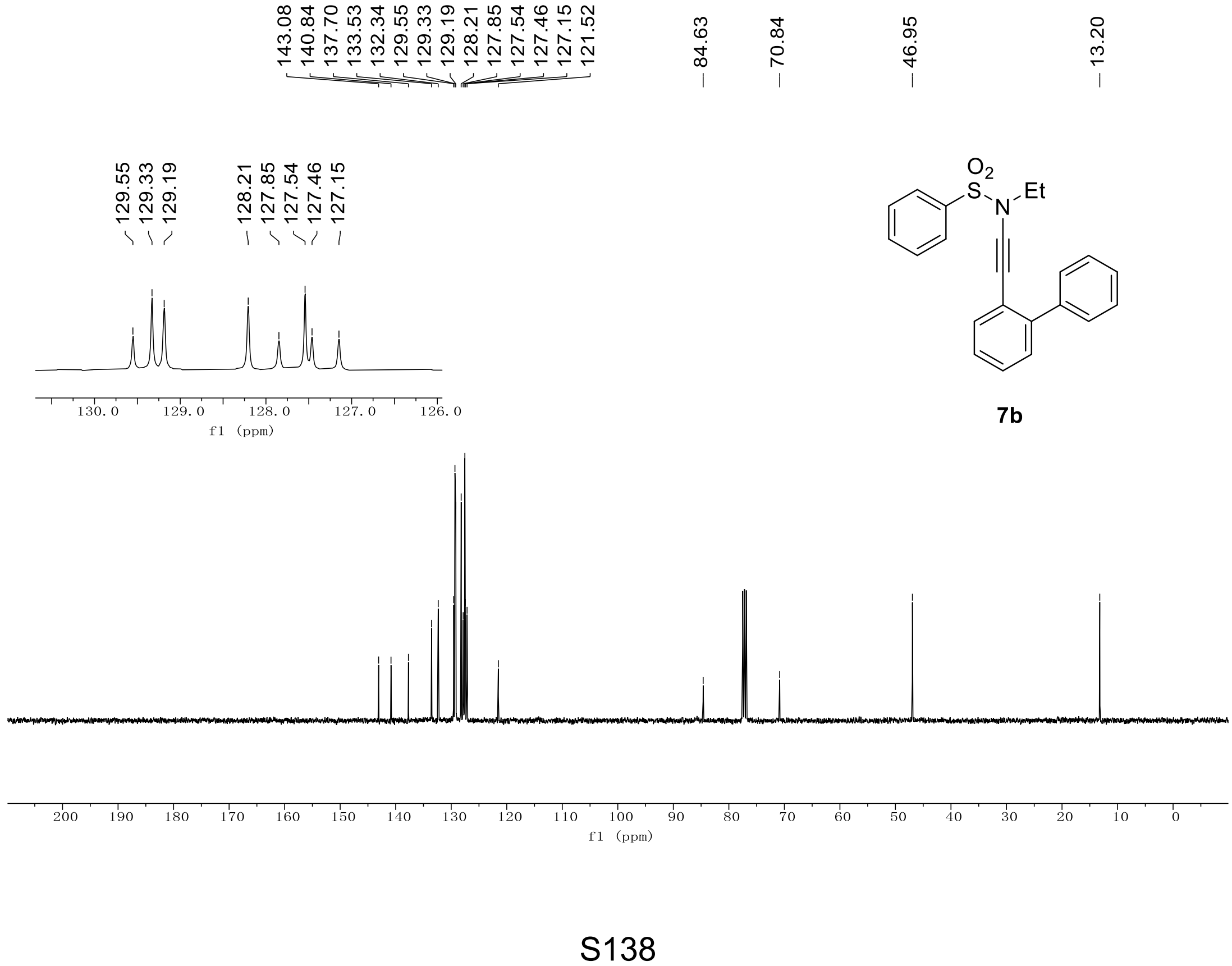

^1H NMR (400 MHz, $CDCl_3$)

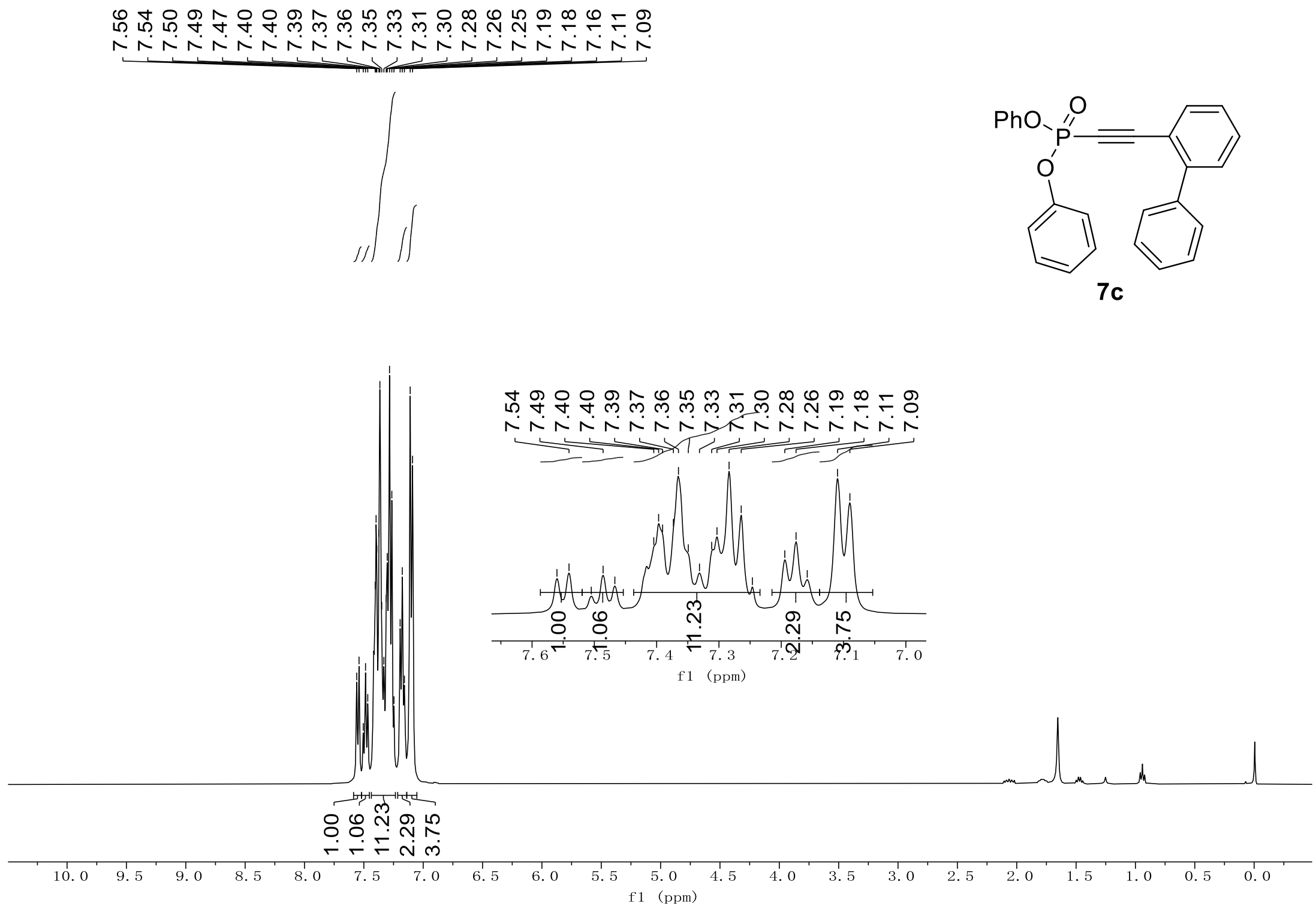

^{13}C NMR (100 MHz, $CDCl_3$)

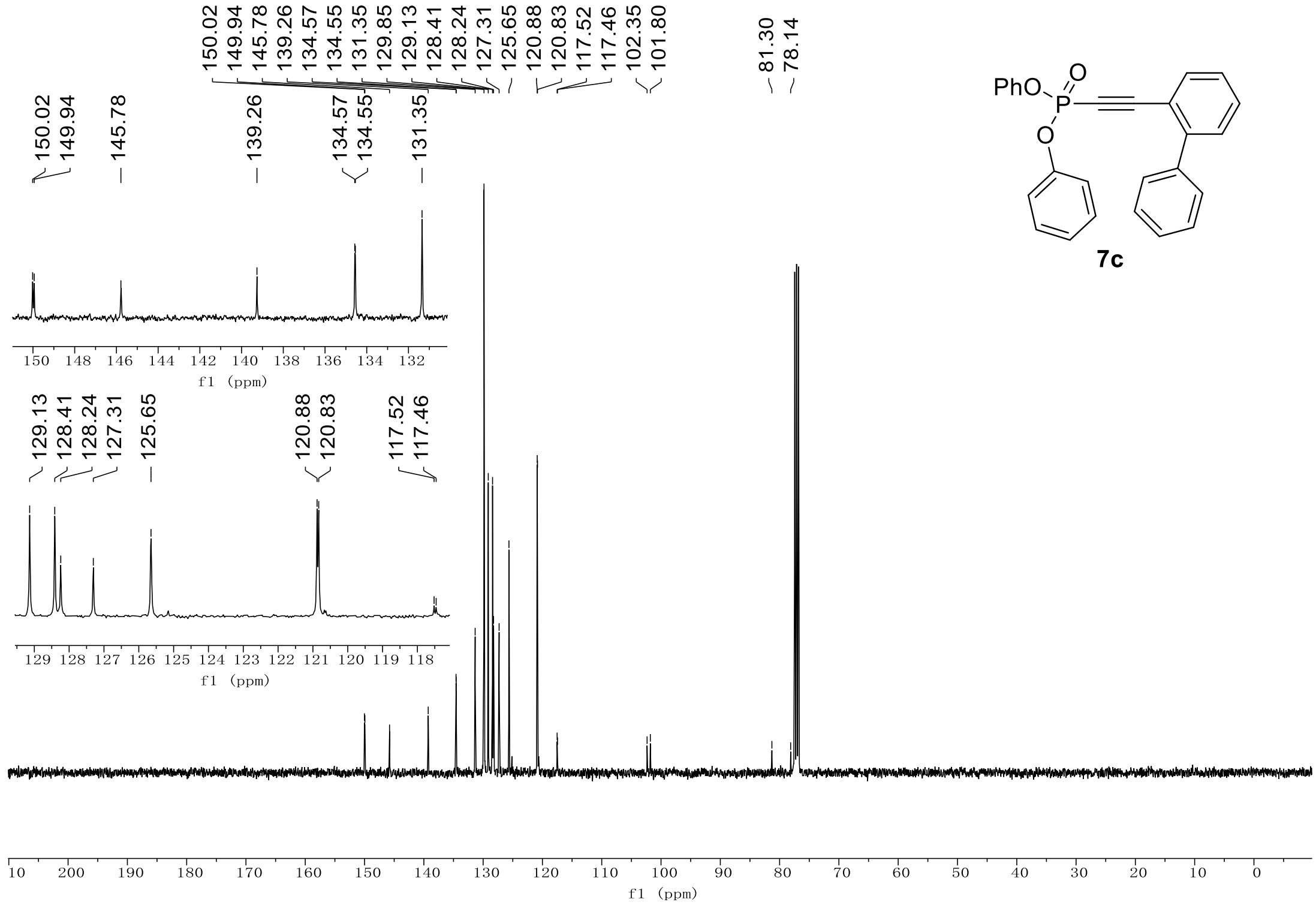

^{31}P NMR (162 MHz, $CDCl_3$)

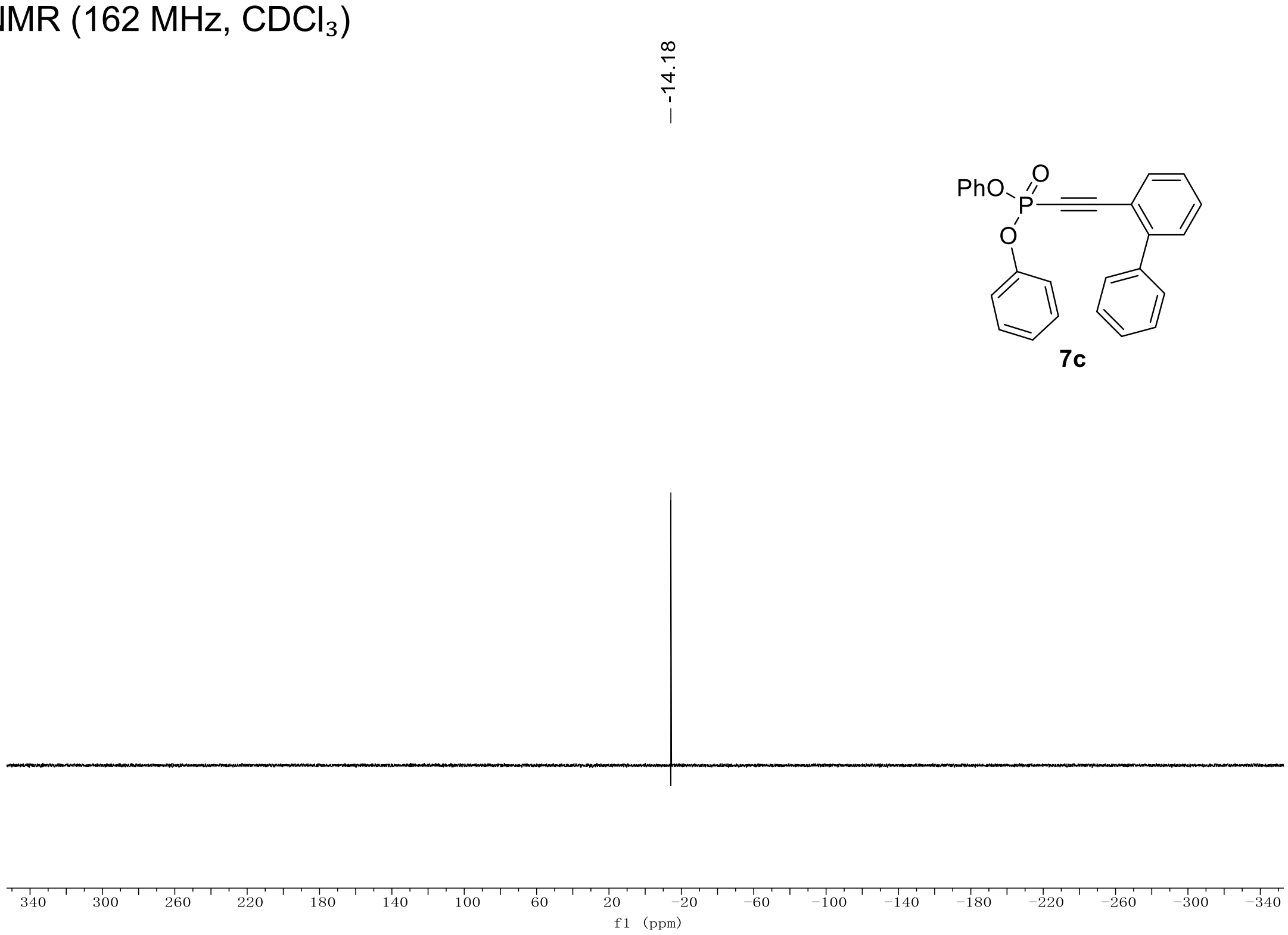

7c

^1H NMR (400 MHz, $CDCl_3$)

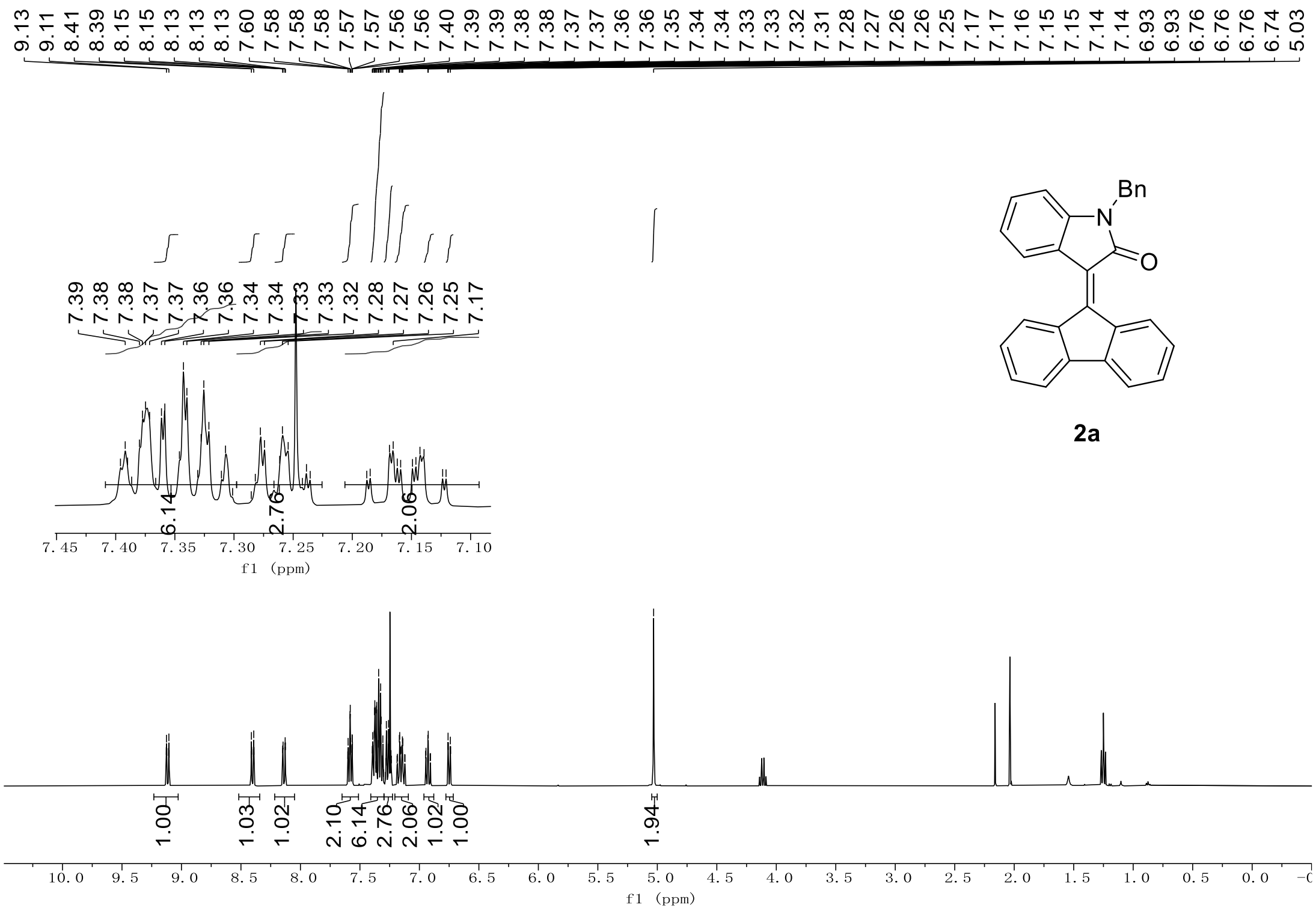

^{13}C NMR (100 MHz, $CDCl_3$)

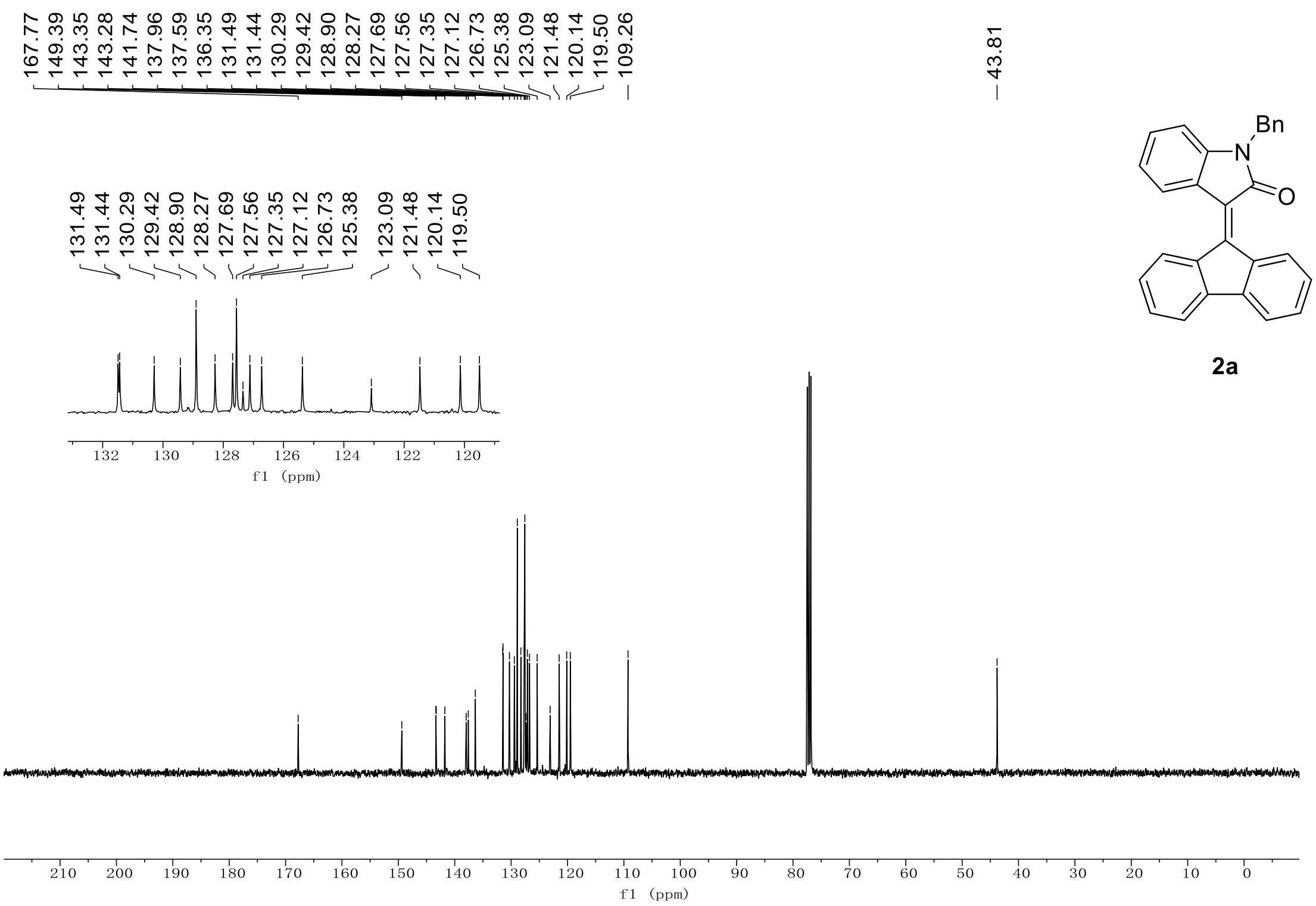

^{1}H NMR (400 MHz, $CDCl_3$)

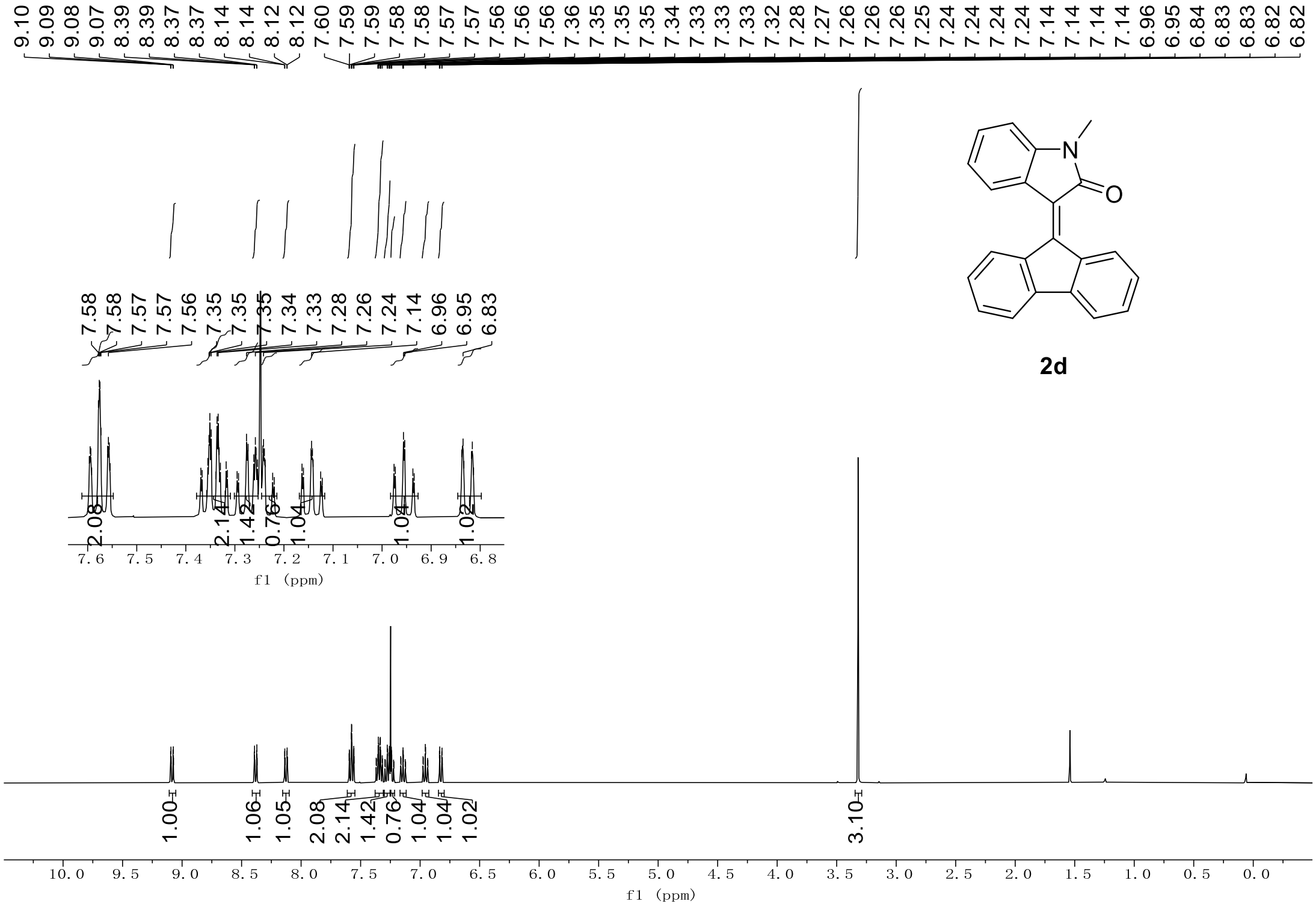

^{13}C NMR (100 MHz, $CDCl_3$)

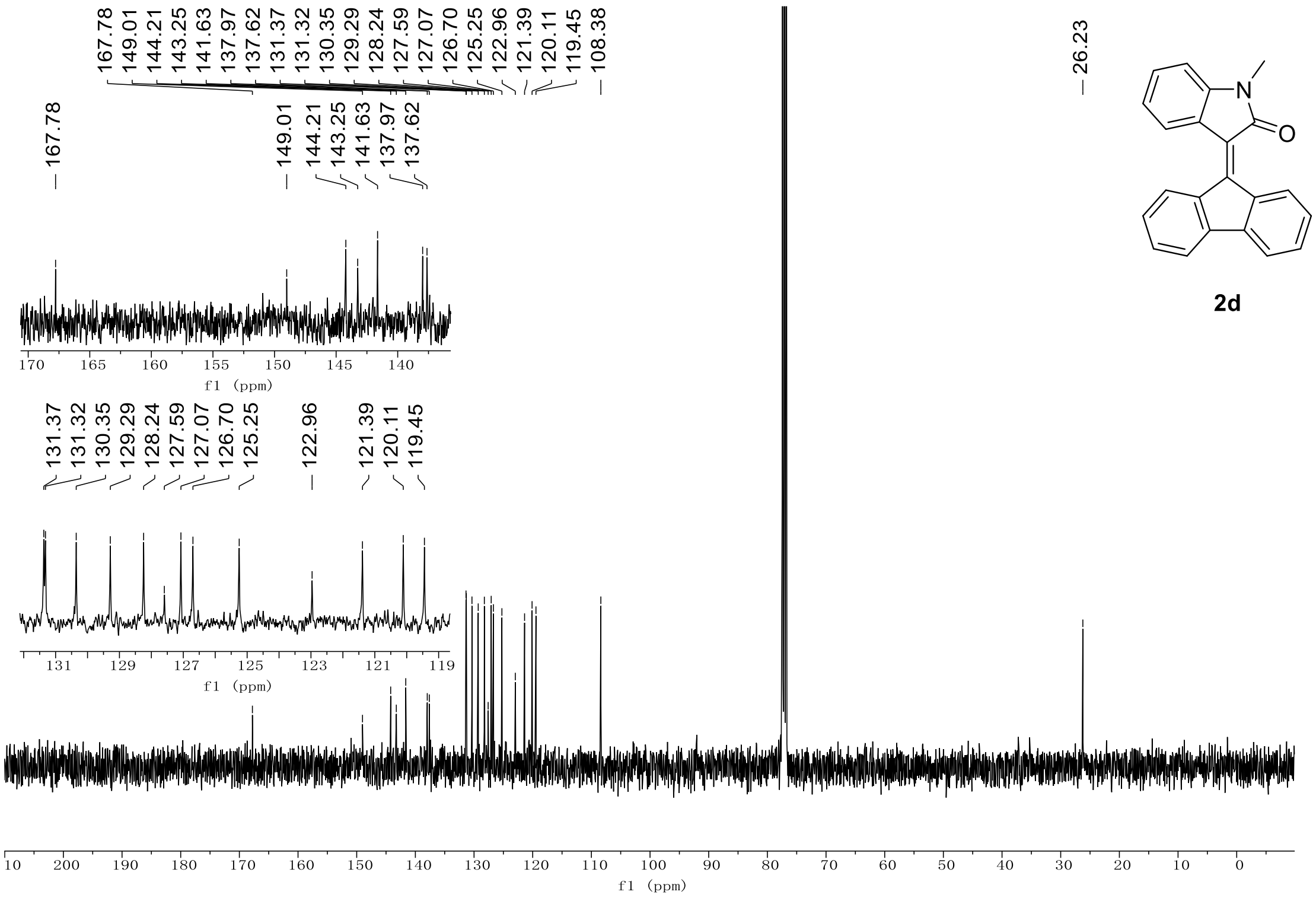

^1H NMR (400 MHz, $CDCl_3$)

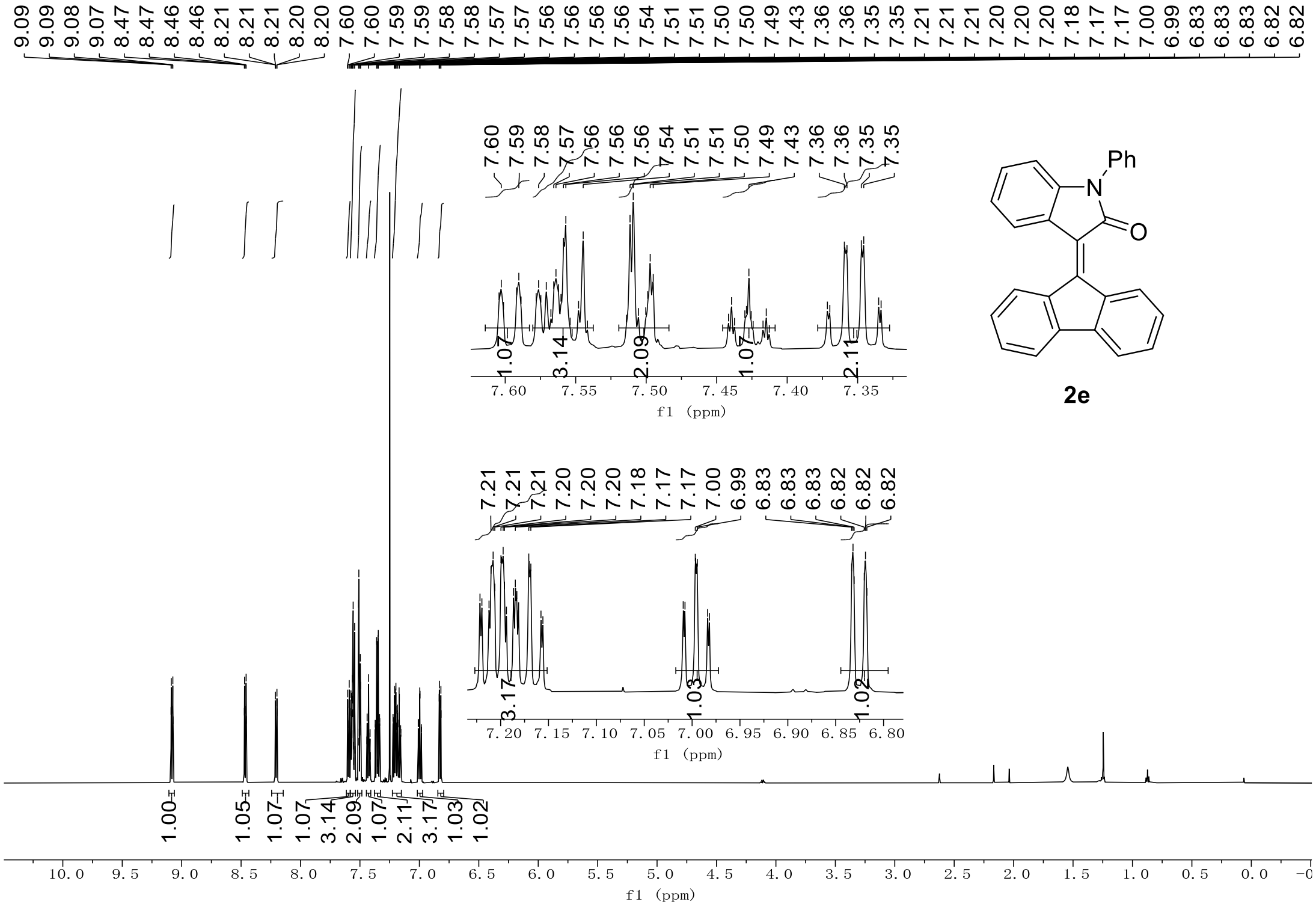

^{13}C NMR (100 MHz, $CDCl_3$)

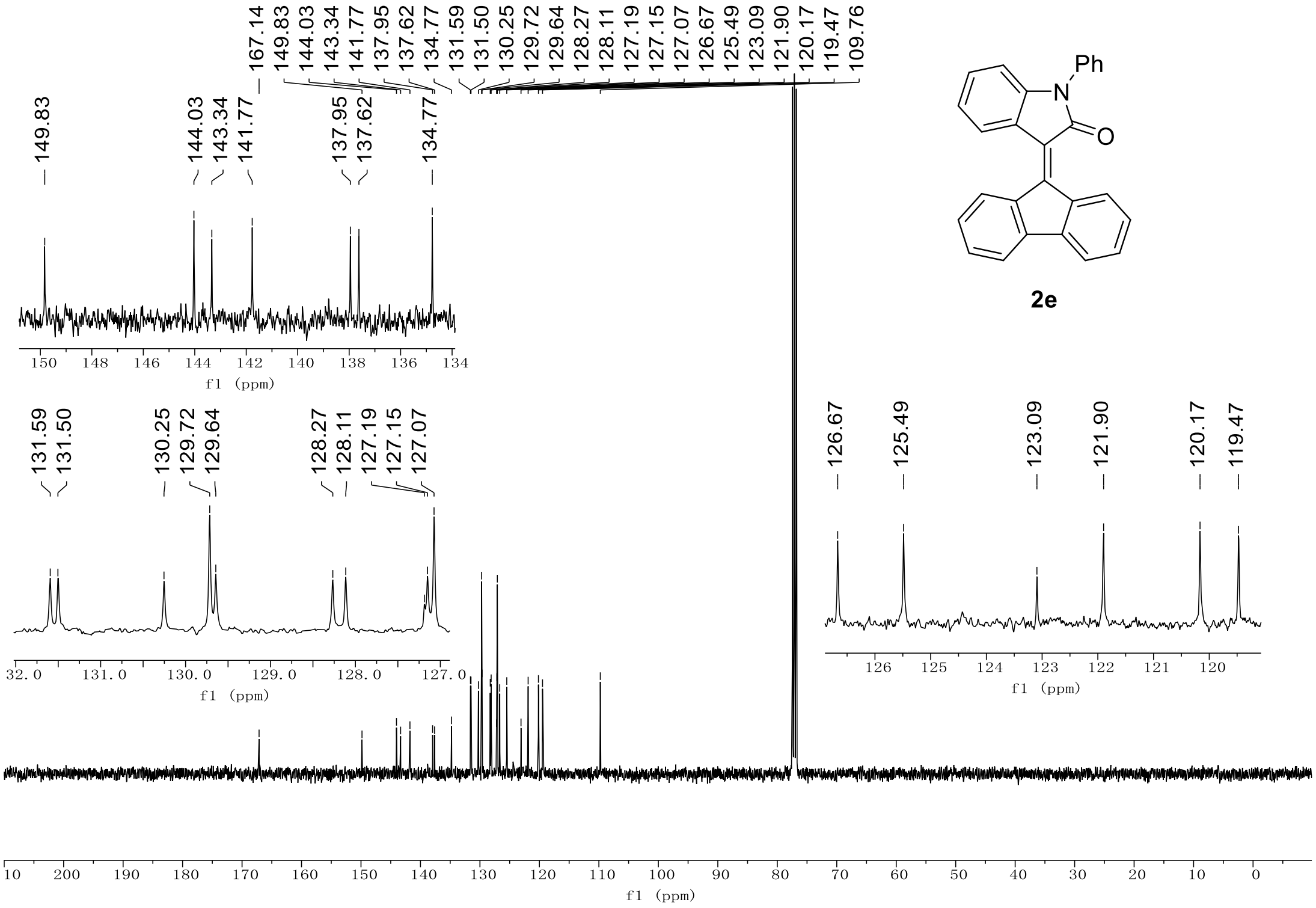

^{1}H NMR (400 MHz, $CDCl_3$)

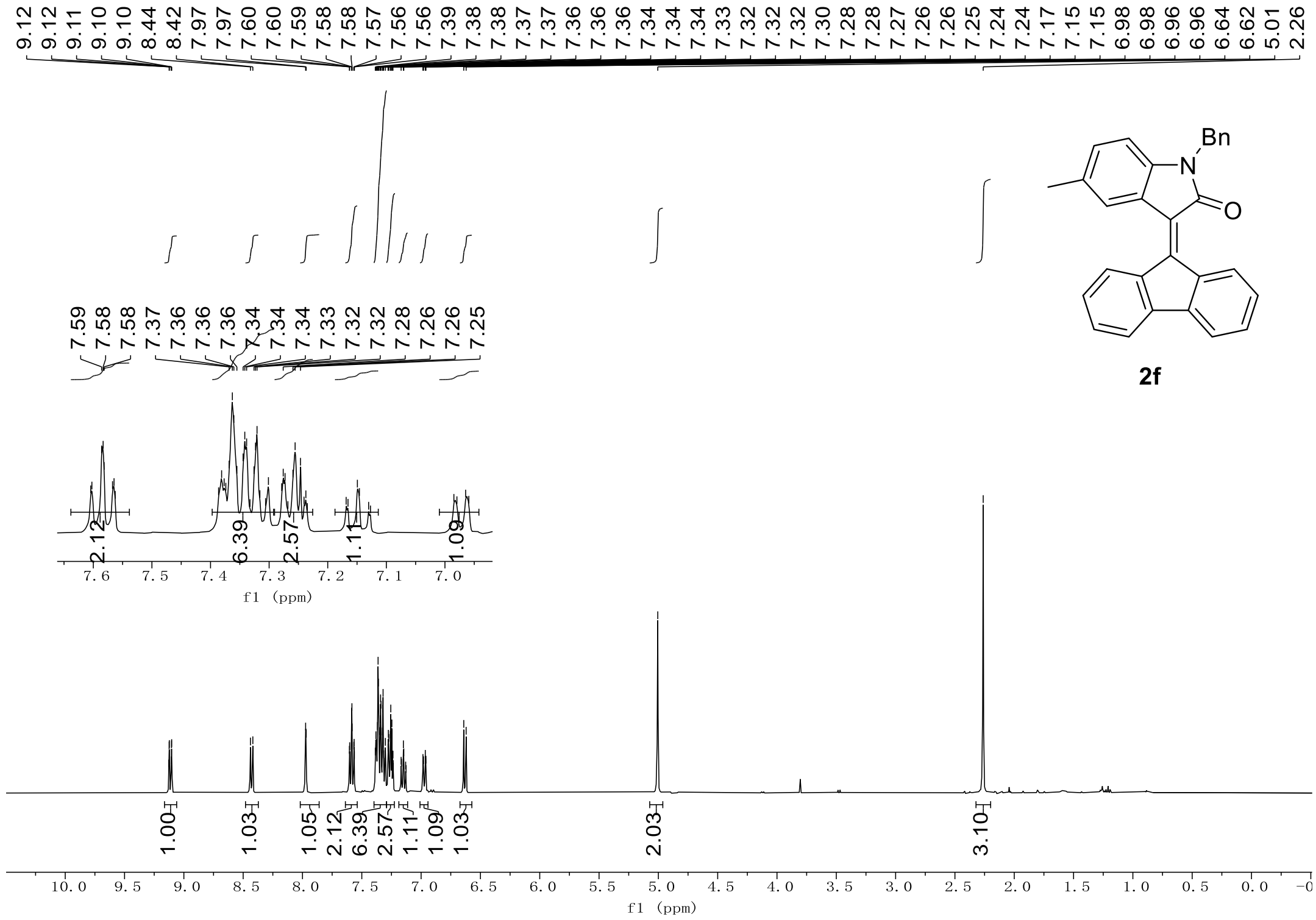

^{13}C NMR (100 MHz, $CDCl_3$)

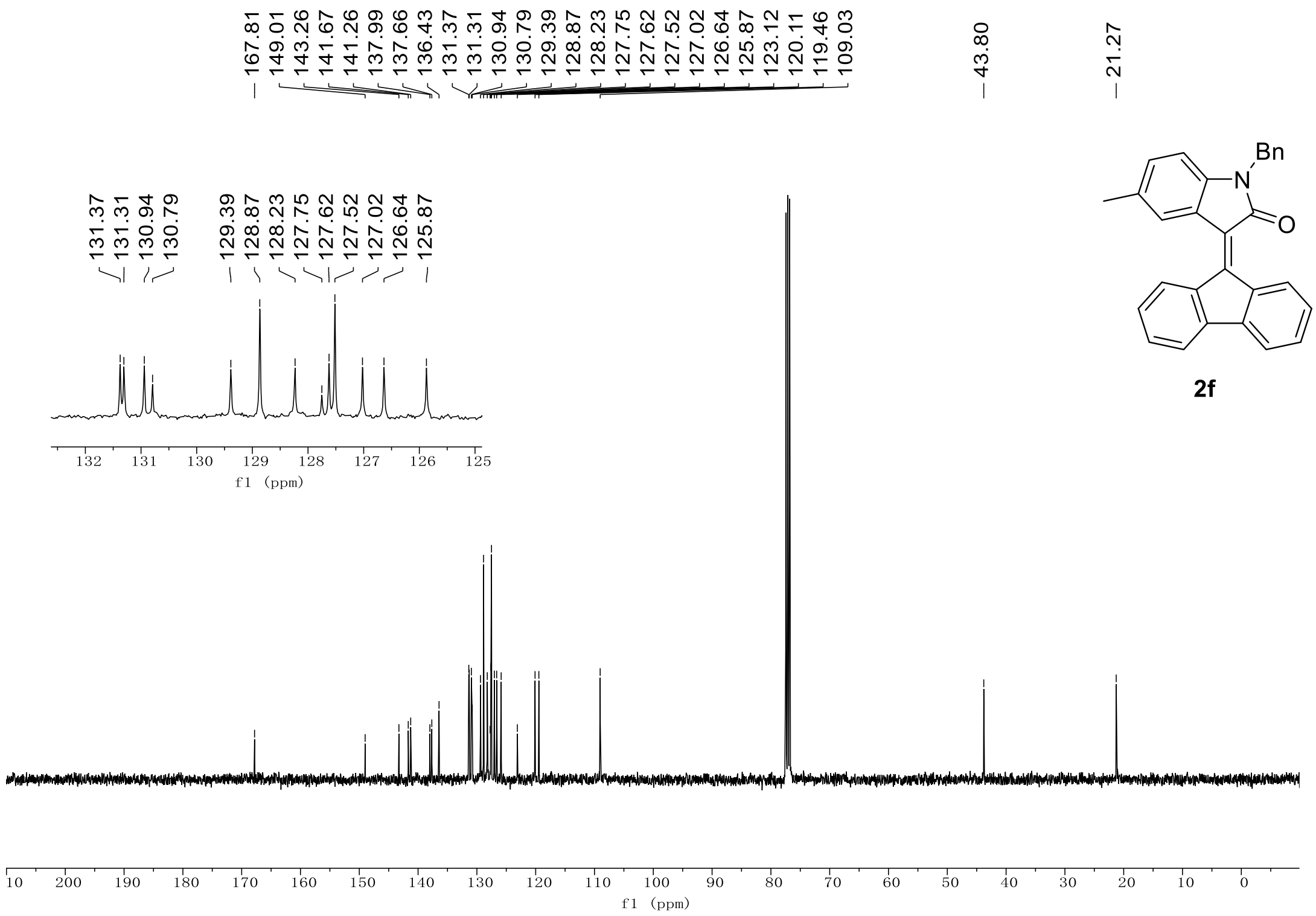

^{1}H NMR (400 MHz, $CDCl_3$)

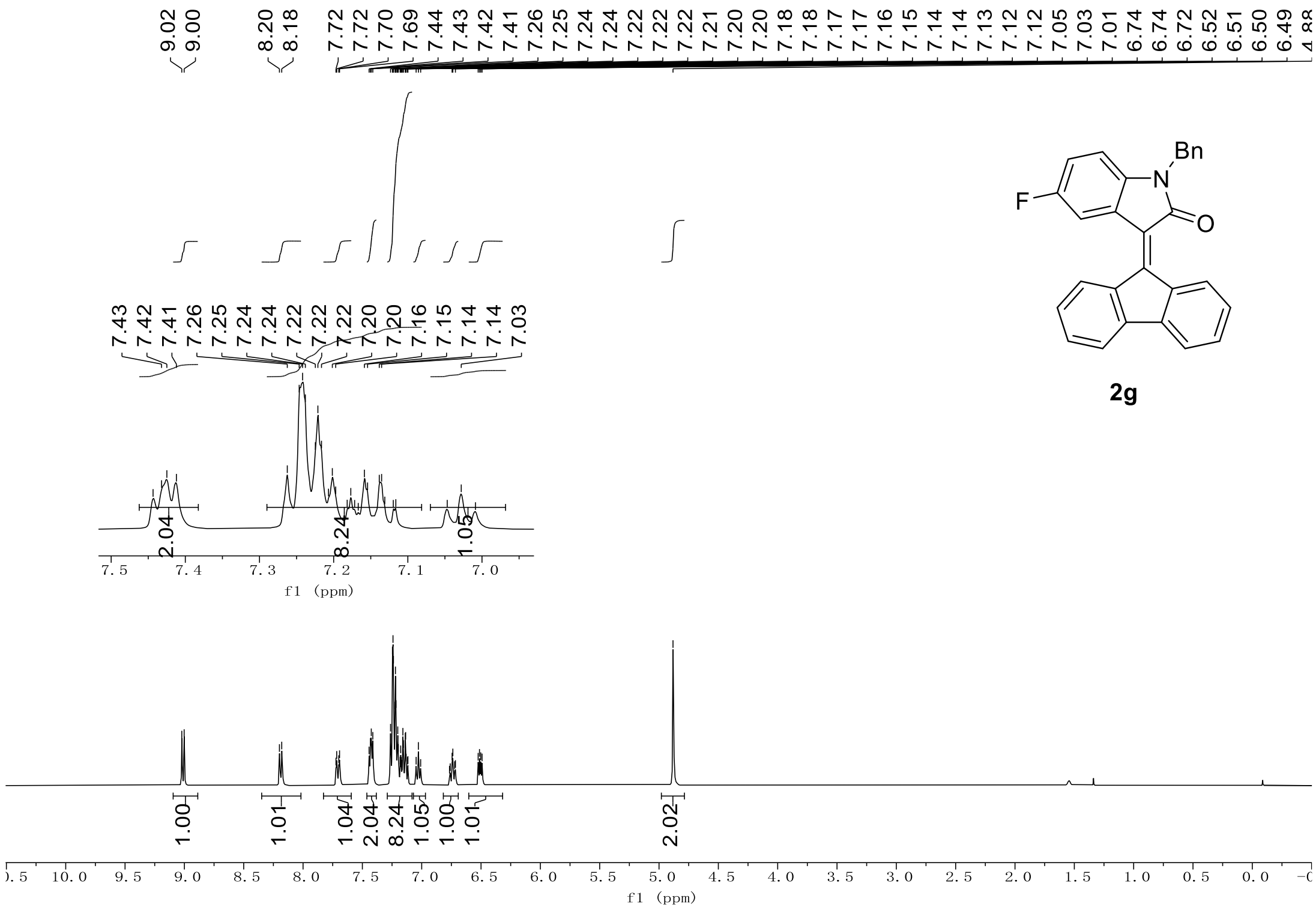

2g

^{13}C NMR (100 MHz, $CDCl_3$)

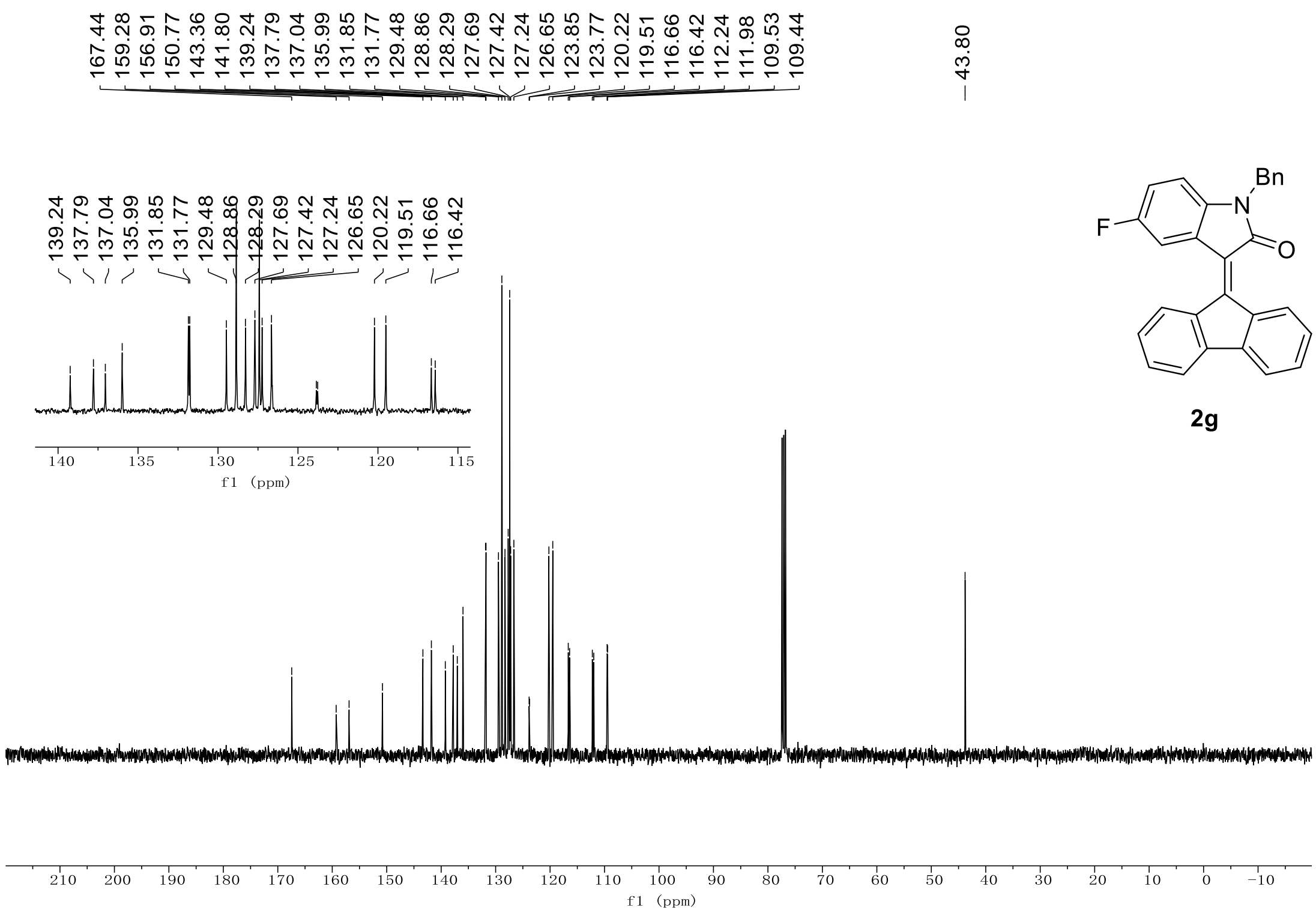

2g

^{19}F NMR (400 MHz, $CDCl_3$)

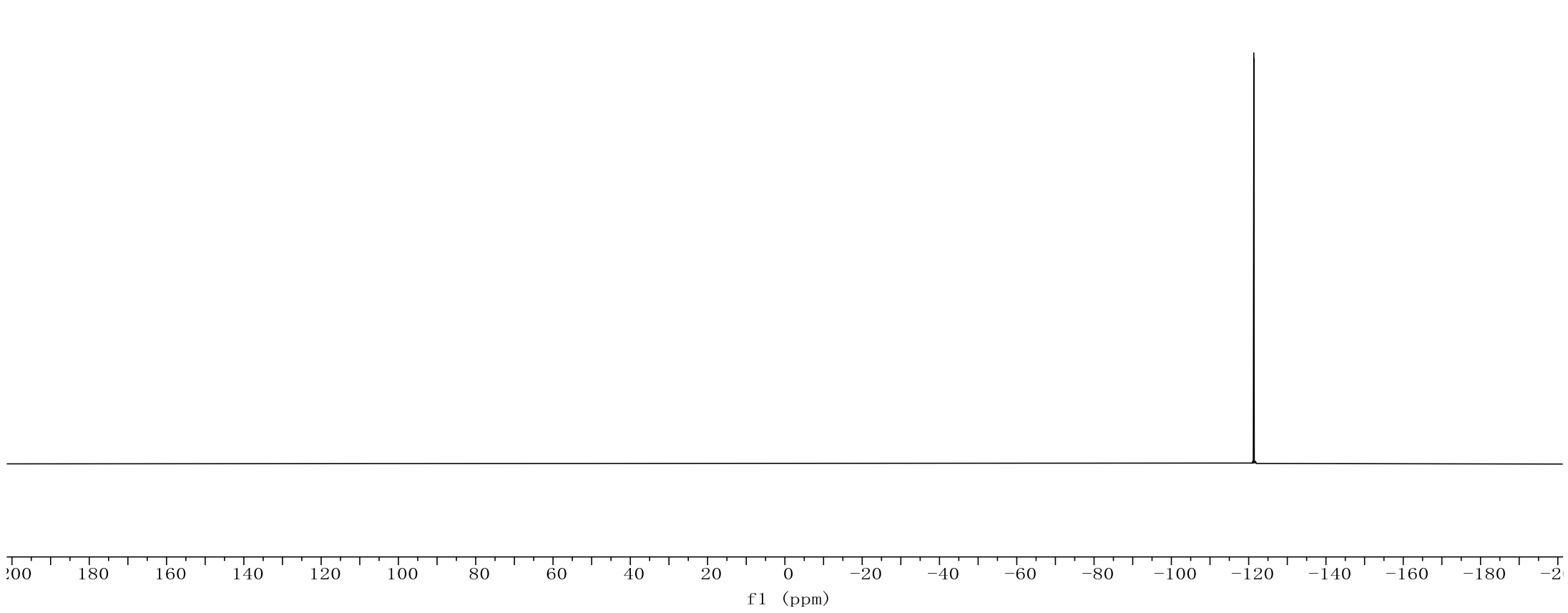

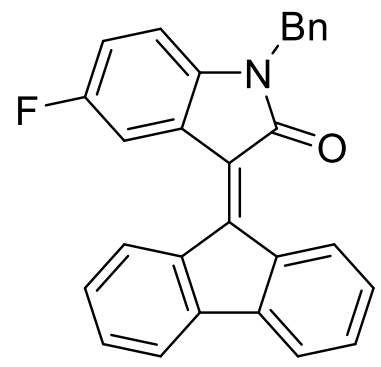

2g

^1H NMR (400 MHz, $CDCl_3$)

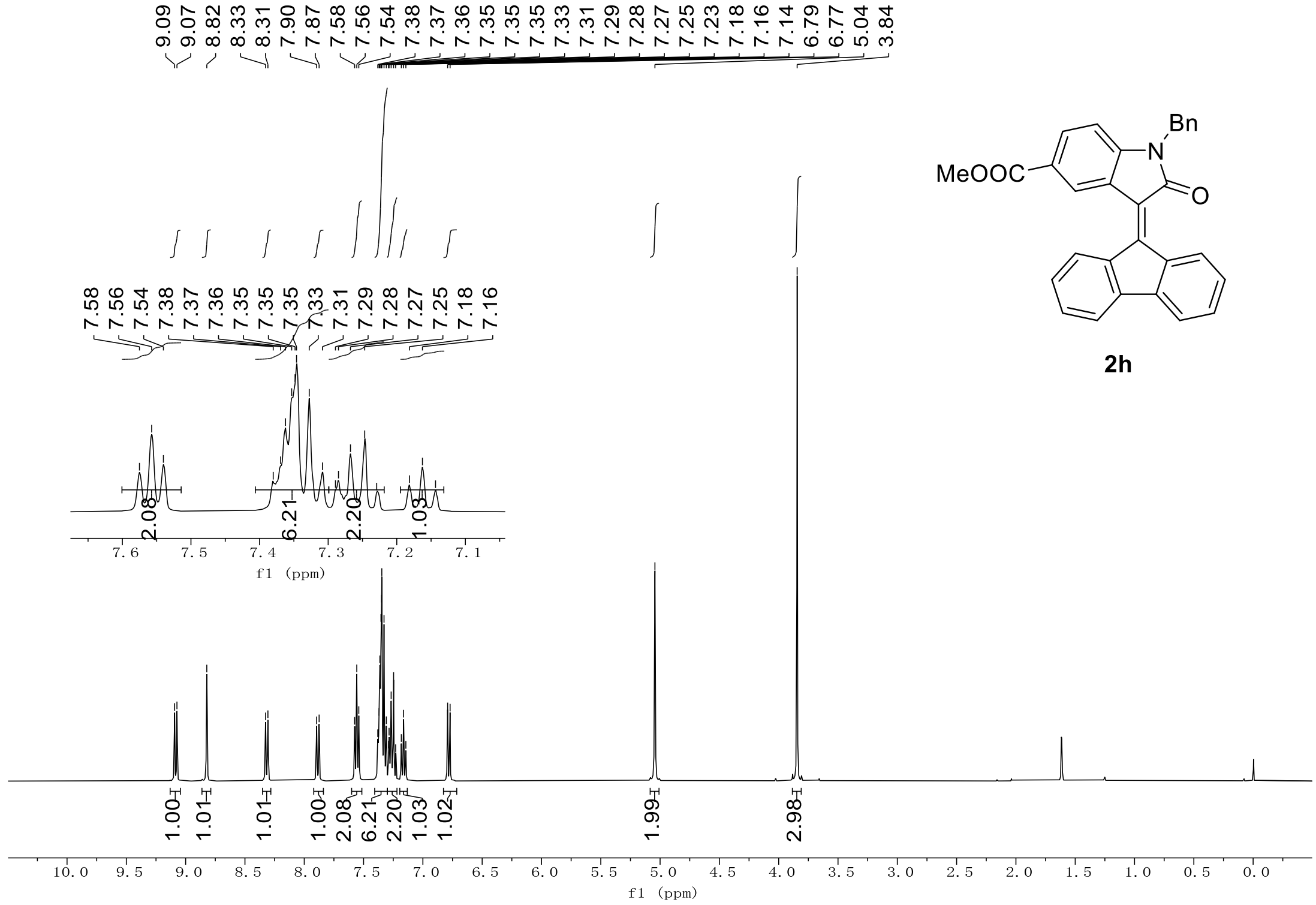

2h

^{13}C NMR (100 MHz, $CDCl_3$)

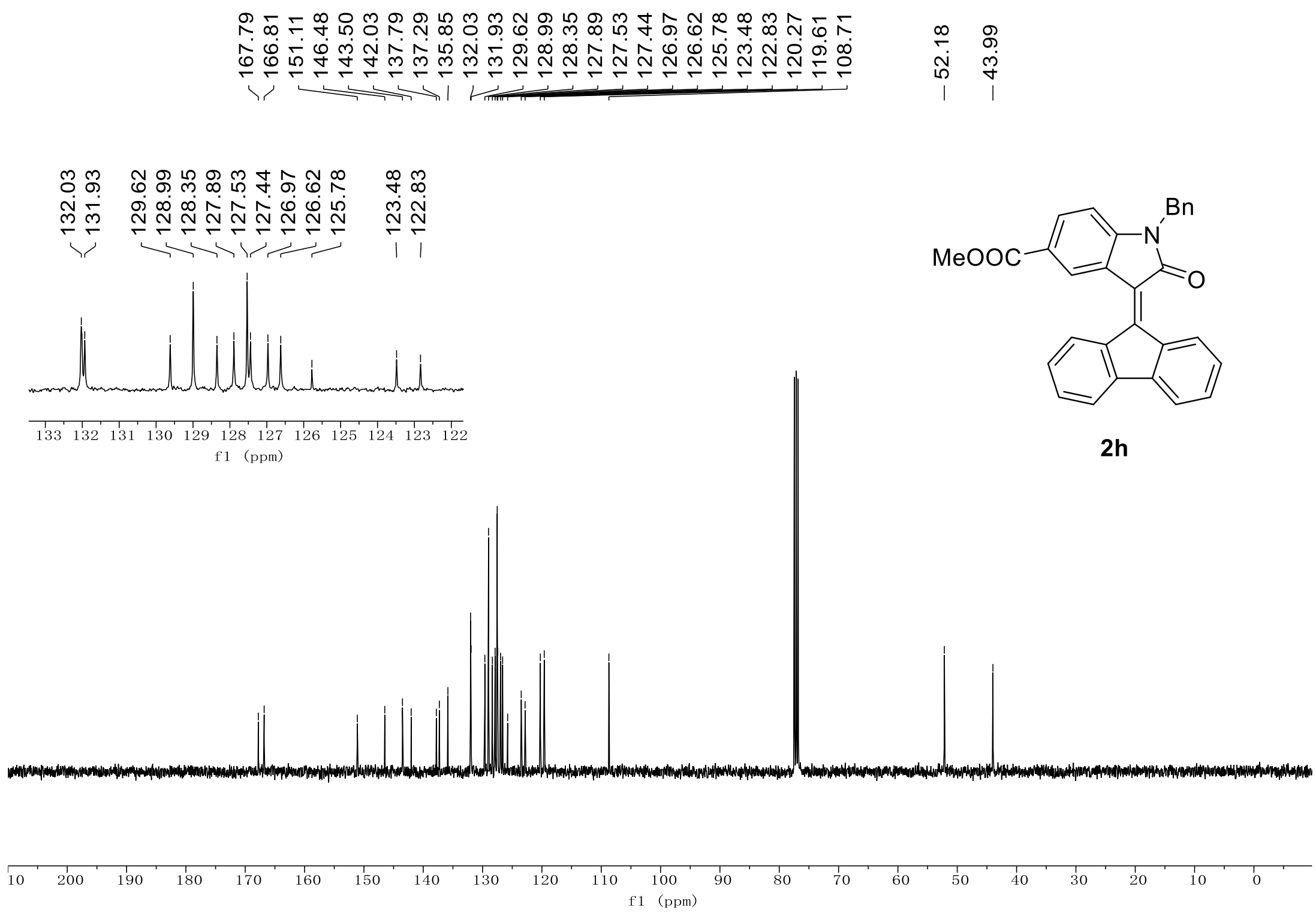

2h

^{1}H NMR (400 MHz, $CDCl_3$)

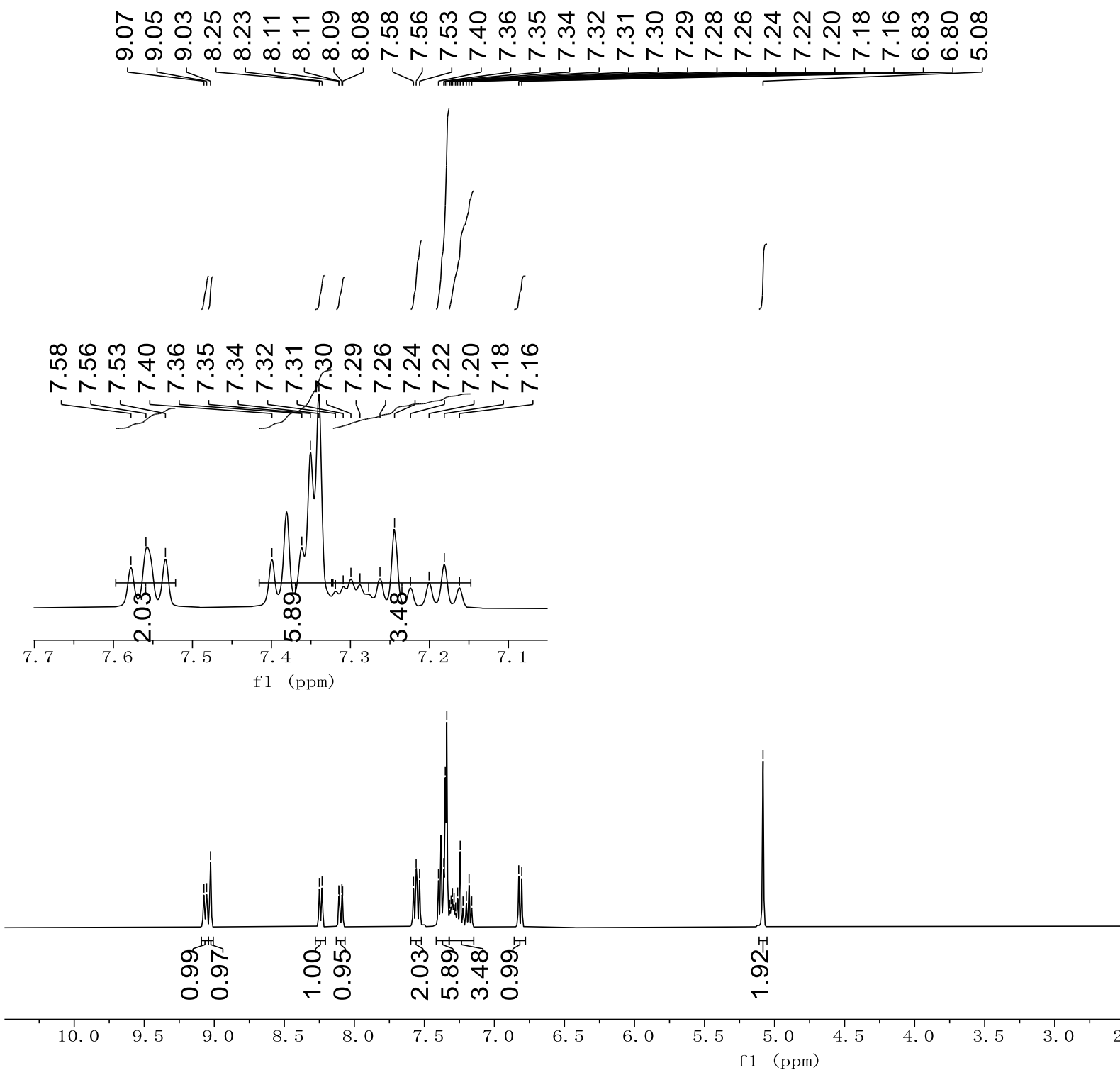

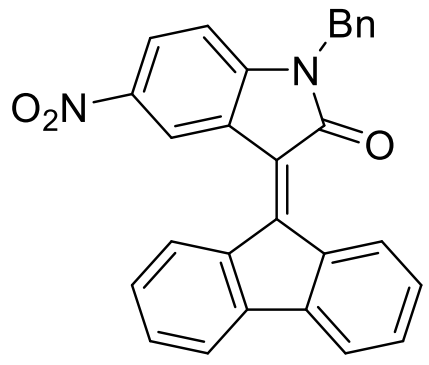

2i

^{13}C NMR (100 MHz, $CDCl_3$)

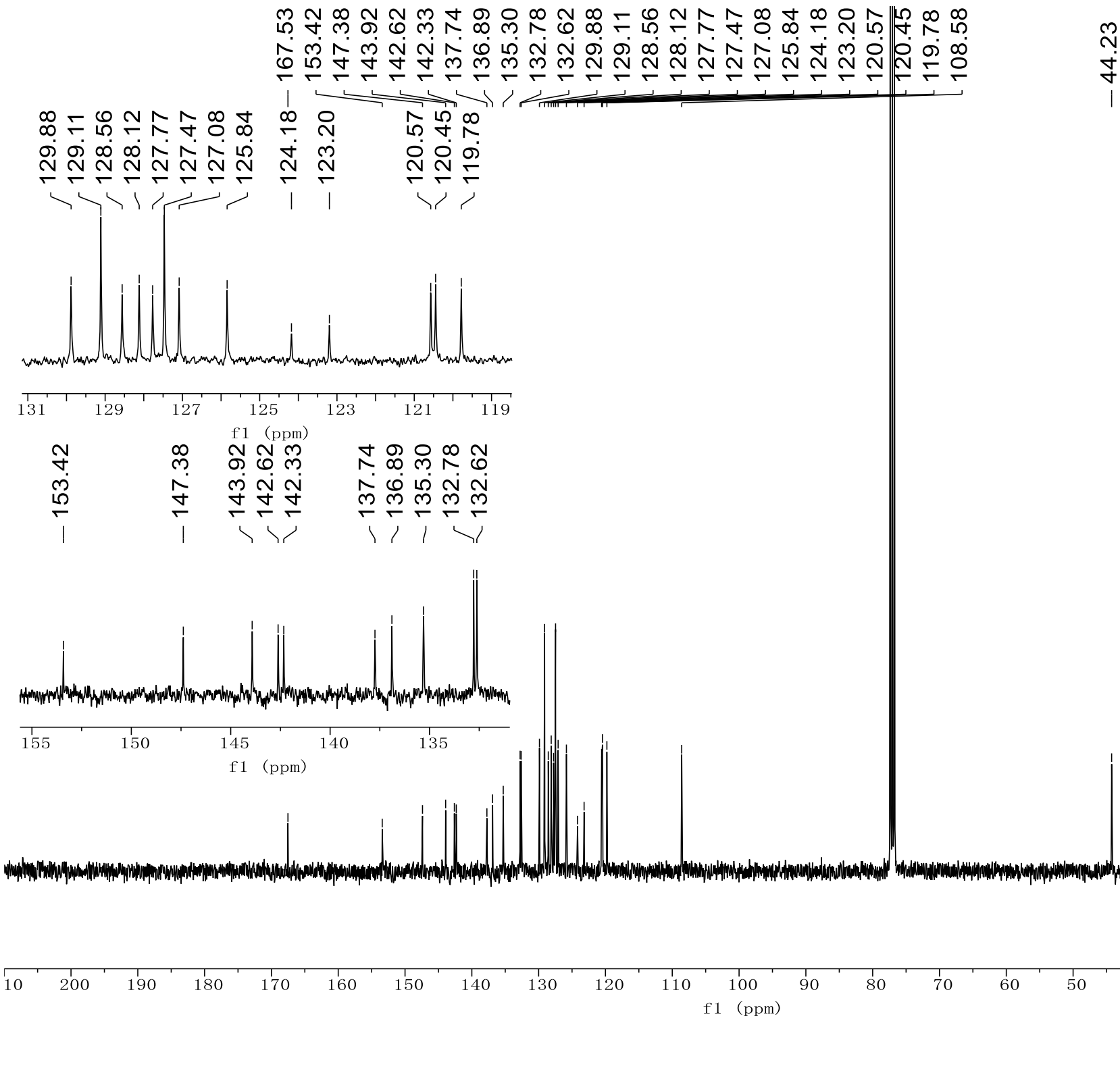

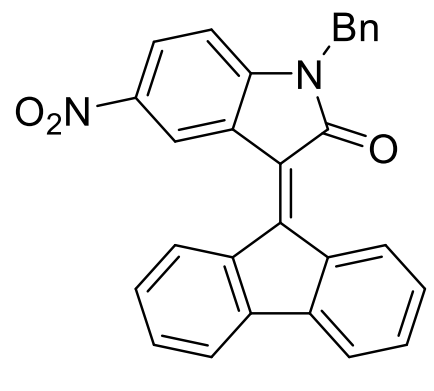

2i

^{1}H NMR (400 MHz, $CDCl_3$)

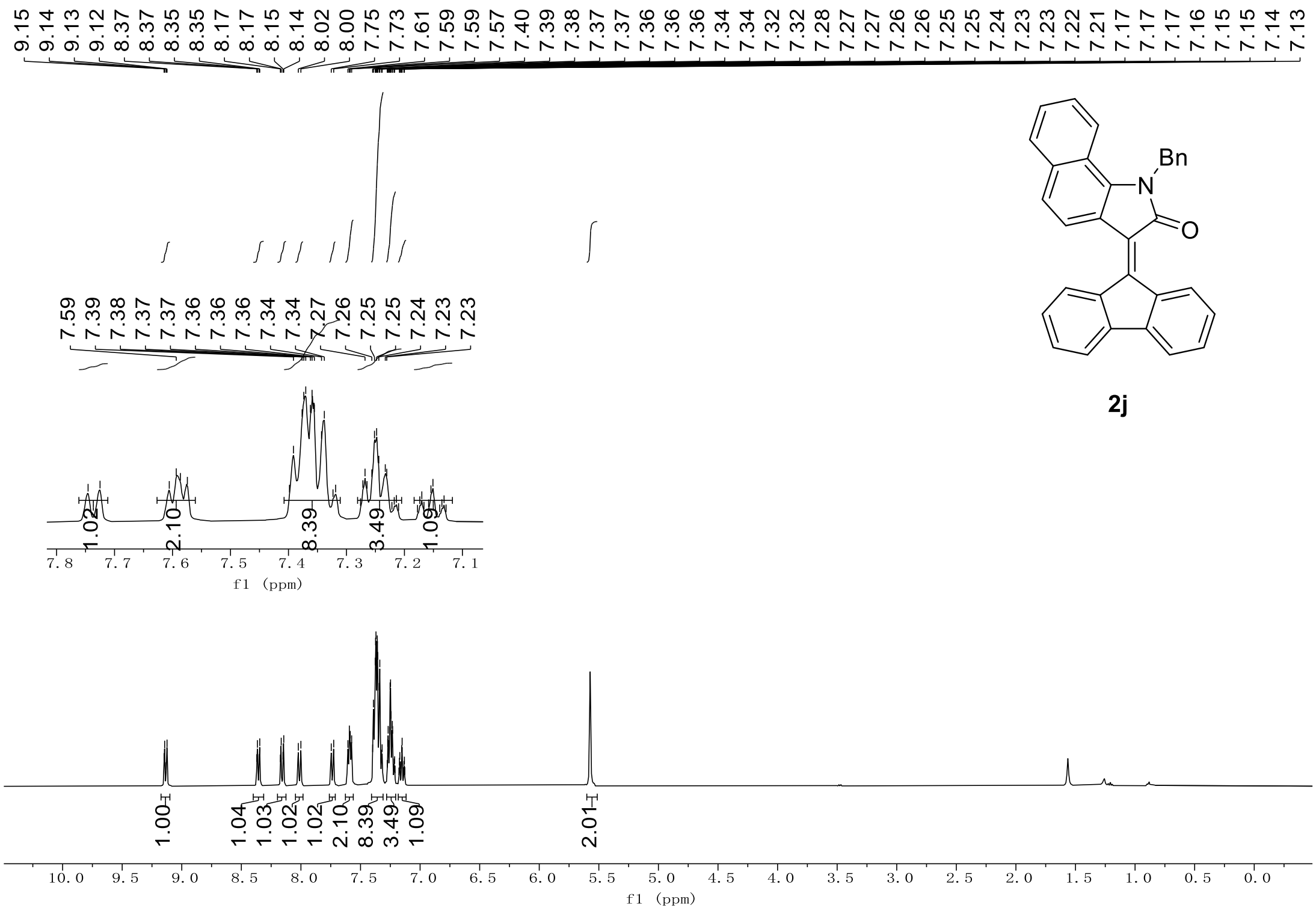

2j

^{13}C NMR (100 MHz, $CDCl_3$)

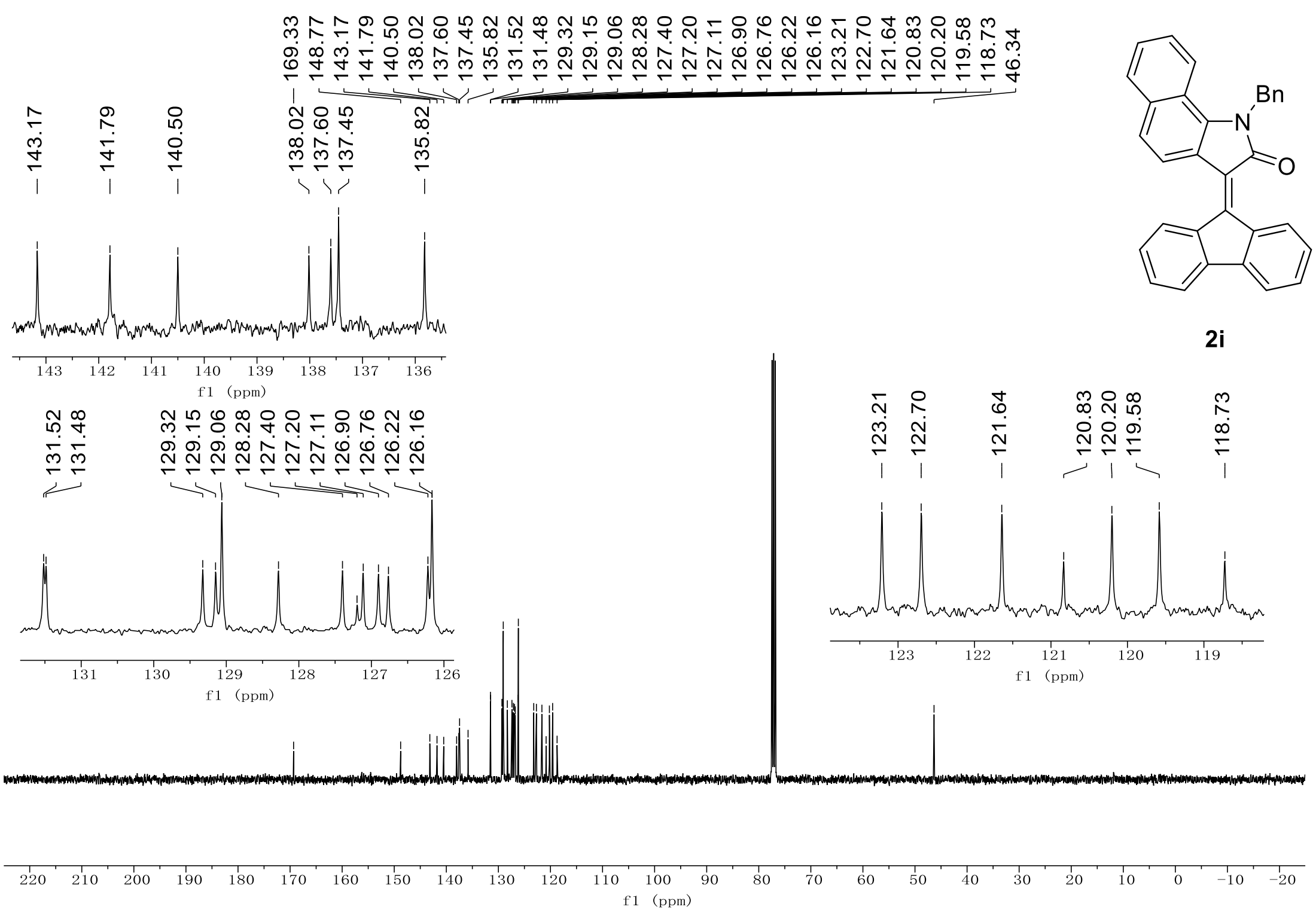

2i

^{1}H NMR (700 MHz, $CDCl_3$)

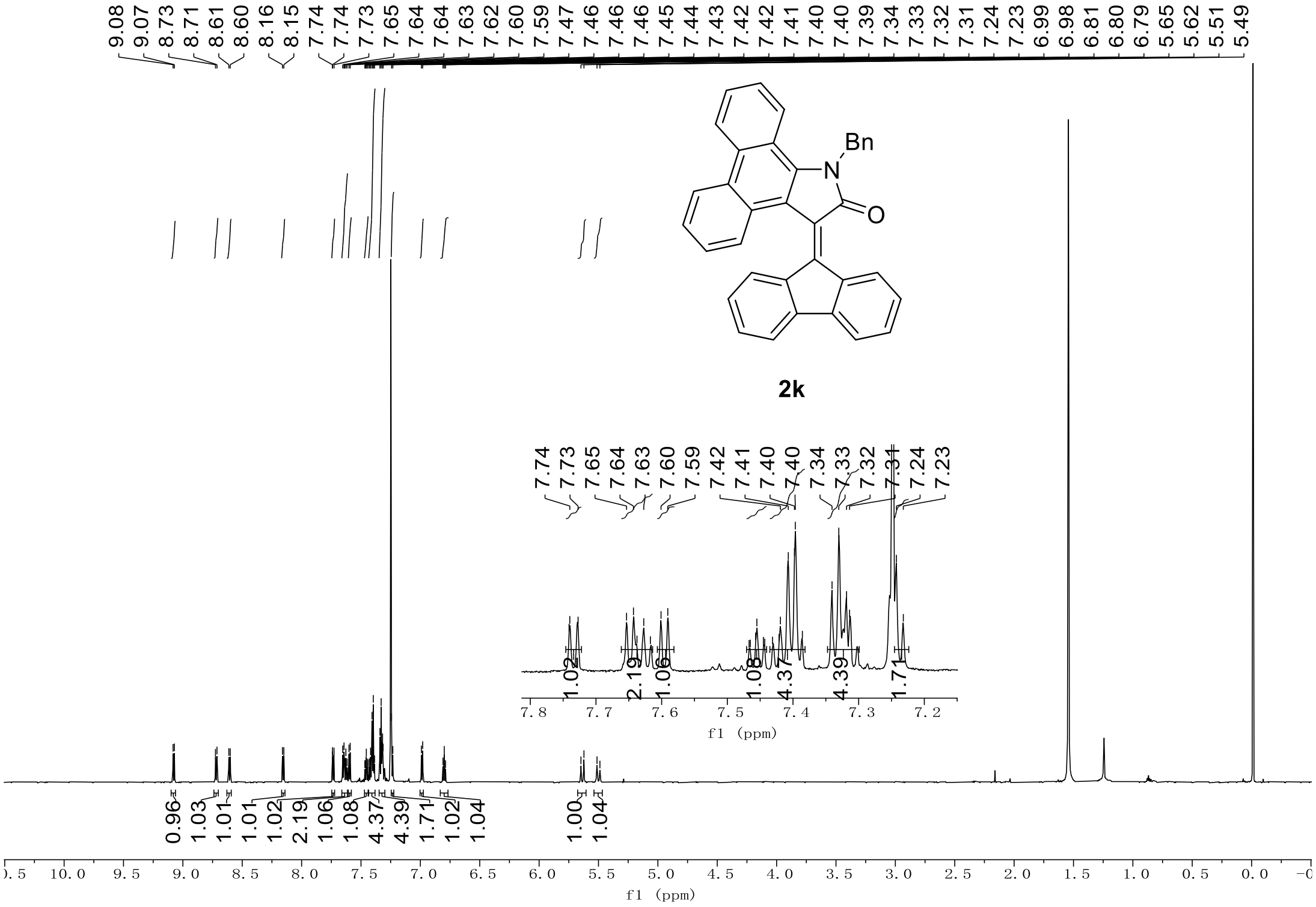

^{13}C NMR (175 MHz, $CDCl_3$)

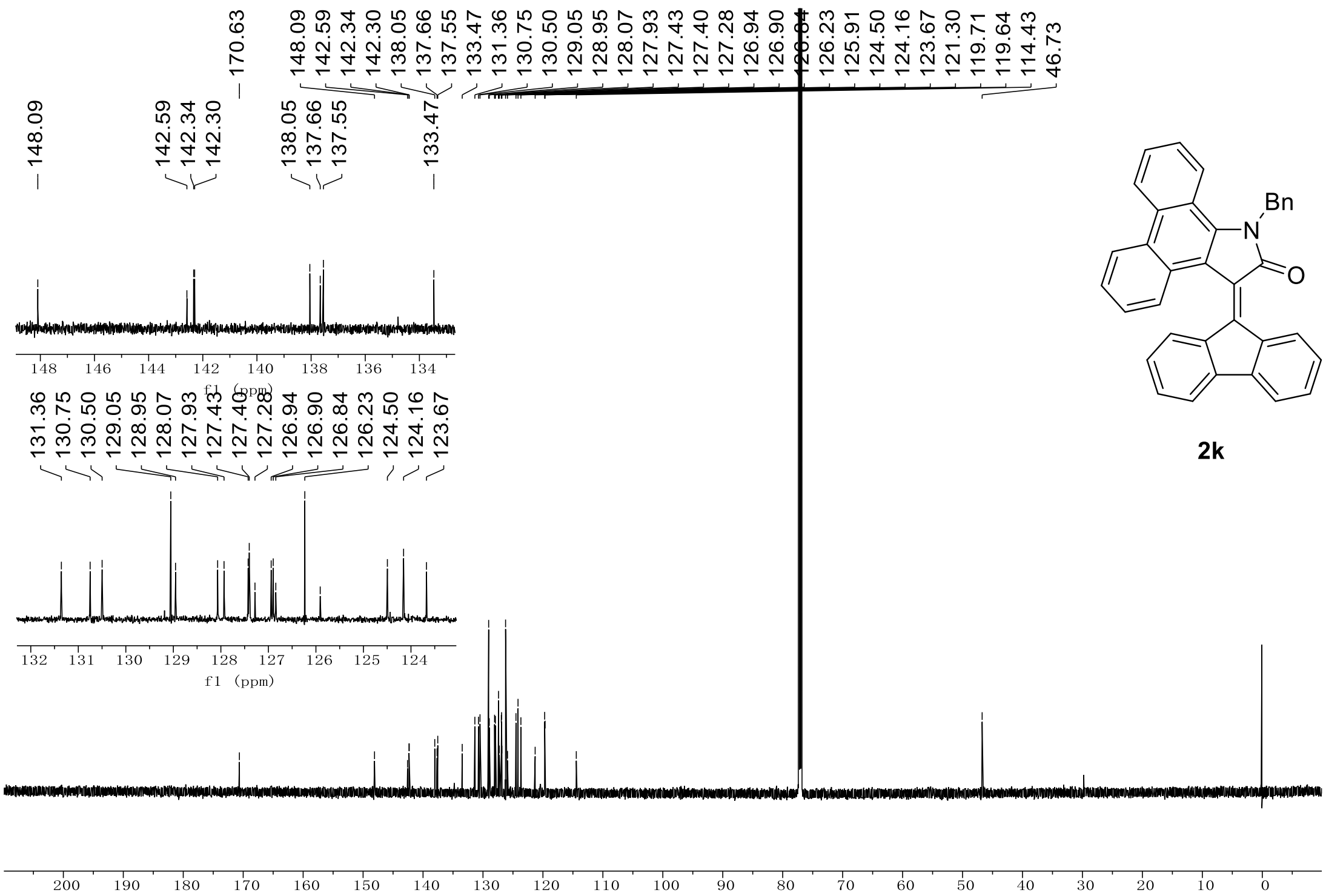

^{1}H NMR (400 MHz, $CDCl_3$)

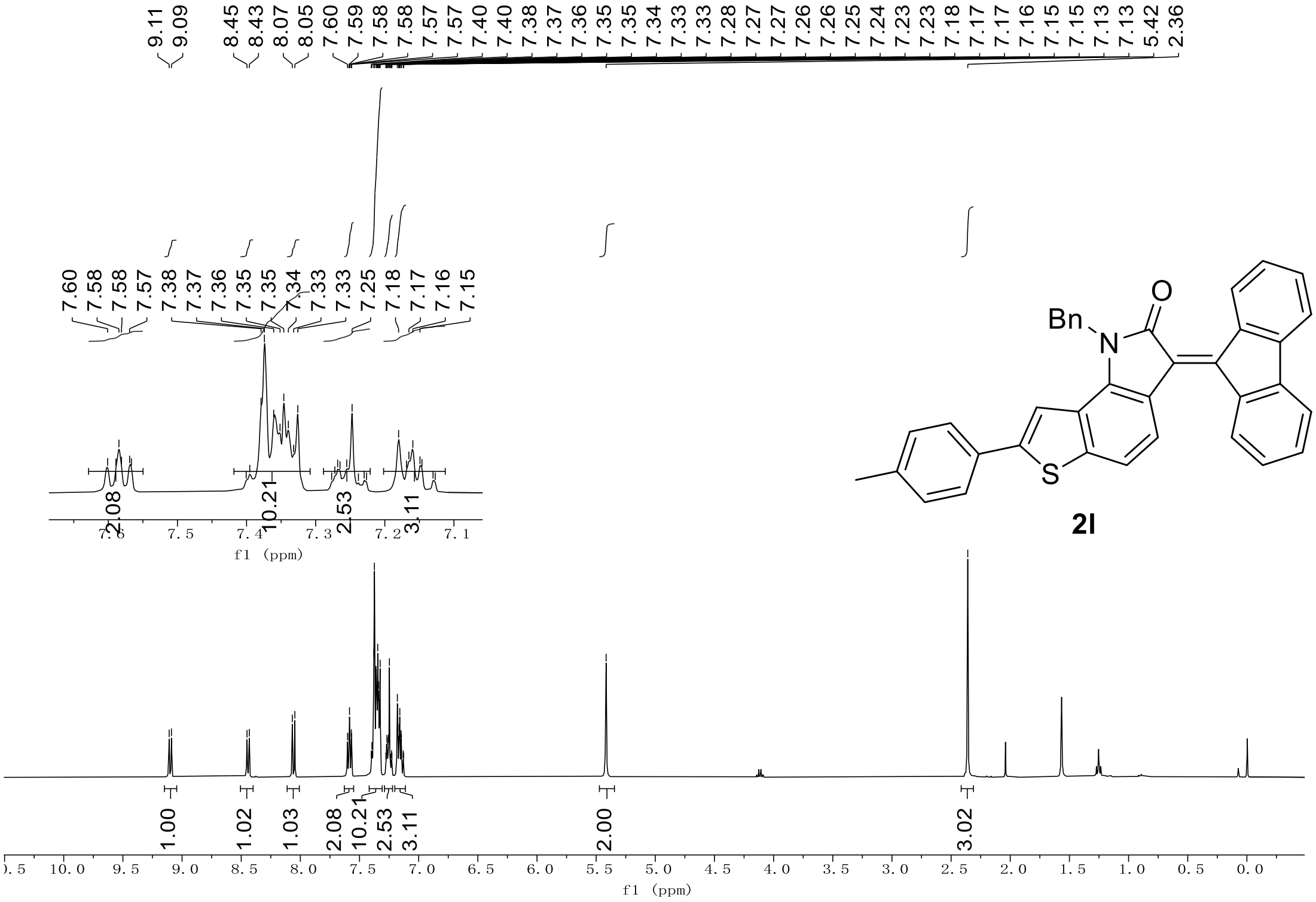

^{13}C NMR (100 MHz, $CDCl_3$)

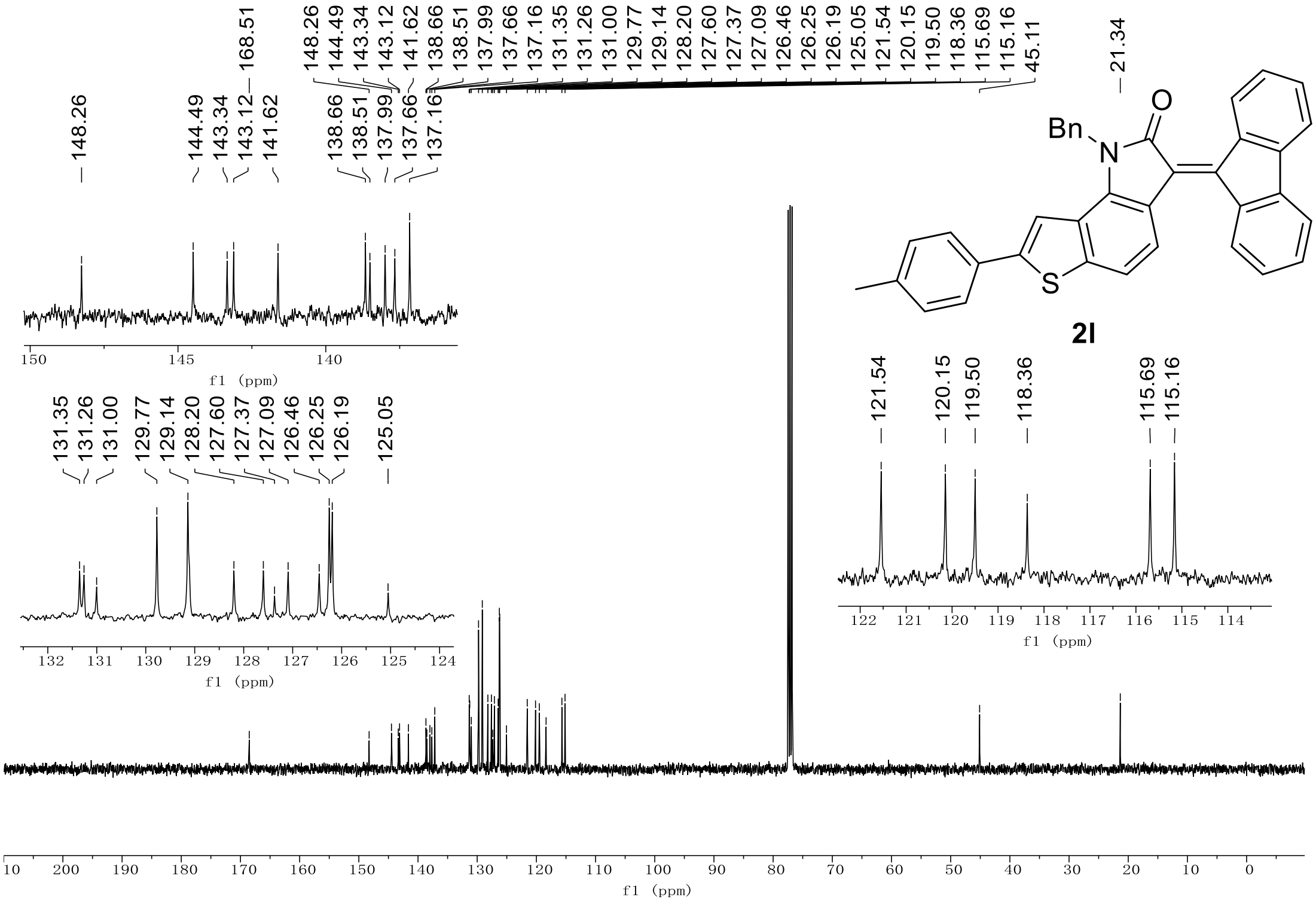

^{1}H NMR (400 MHz, $CDCl_3$)

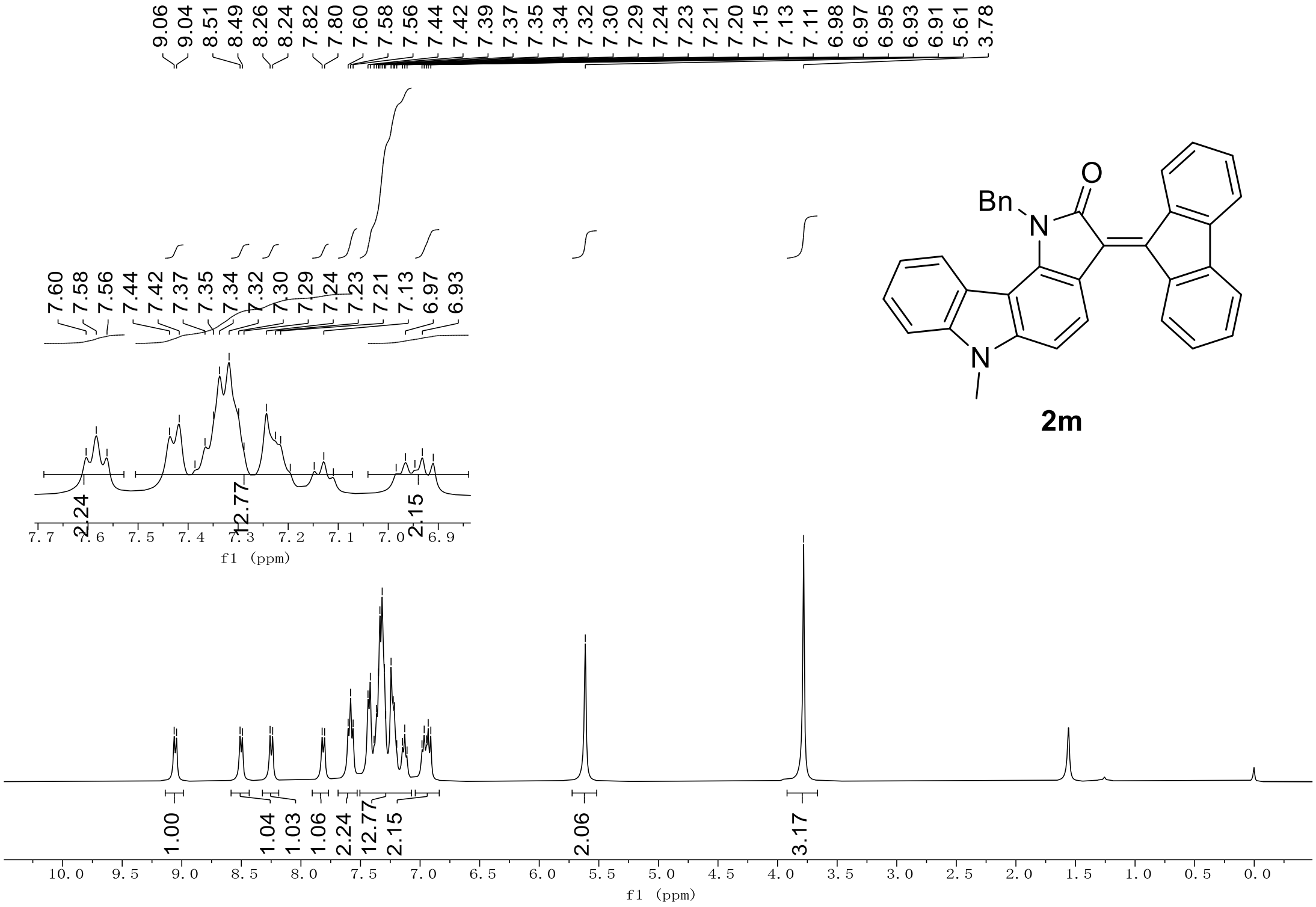

^{13}C NMR (100 MHz, $CDCl_3$)

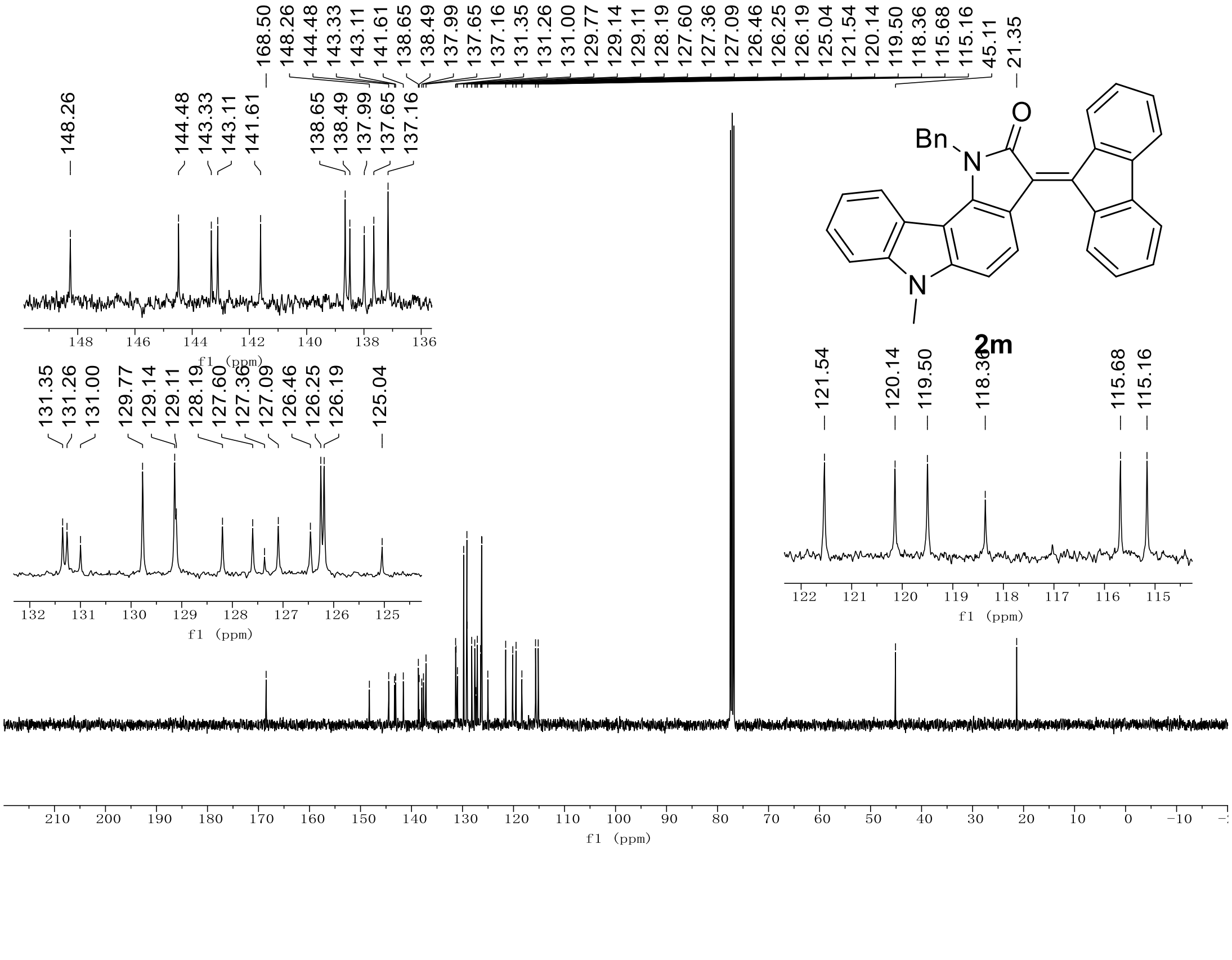

^{1}H NMR (400 MHz, $CDCl_3$)

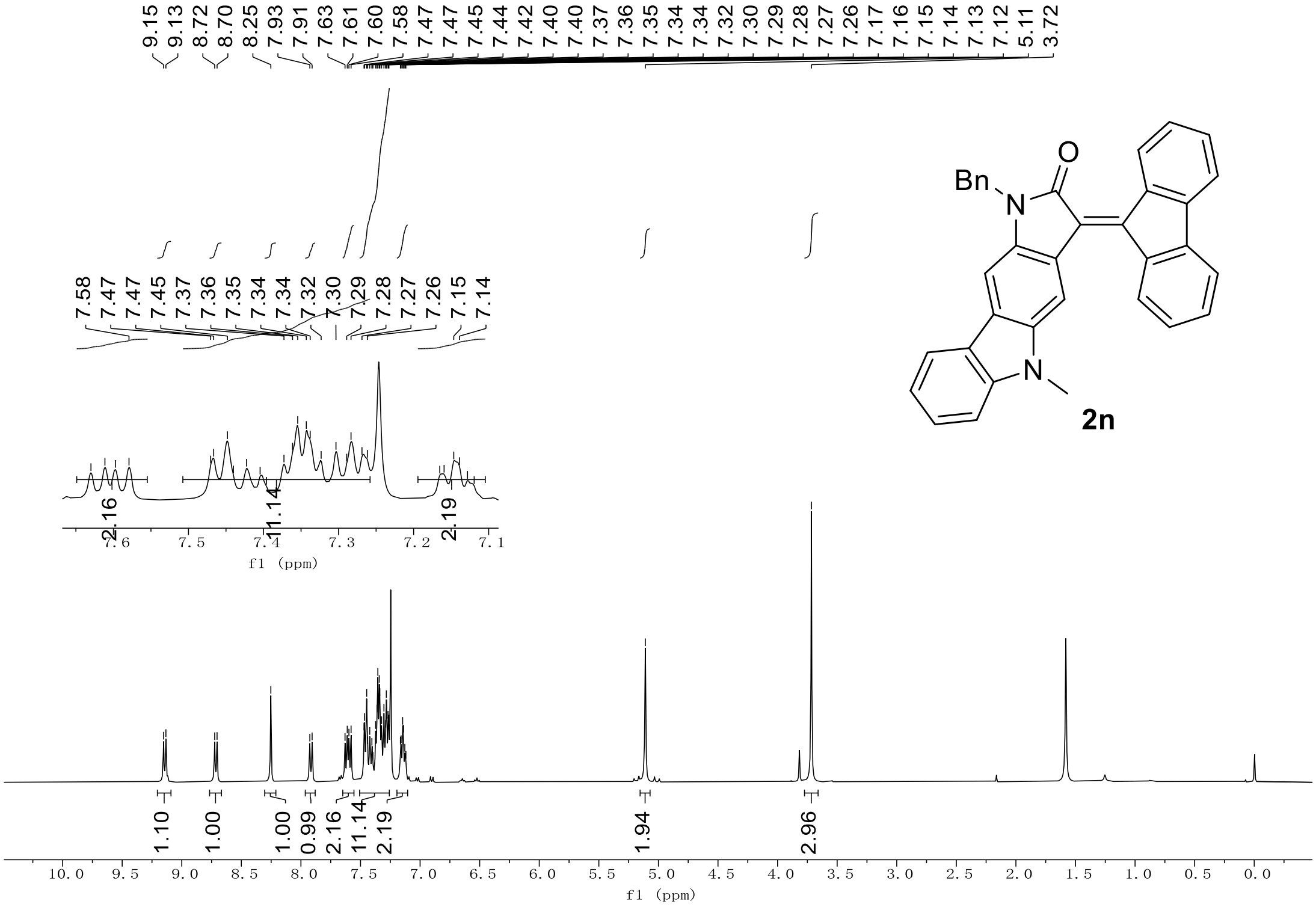

^{13}C NMR (100 MHz, $CDCl_3$)

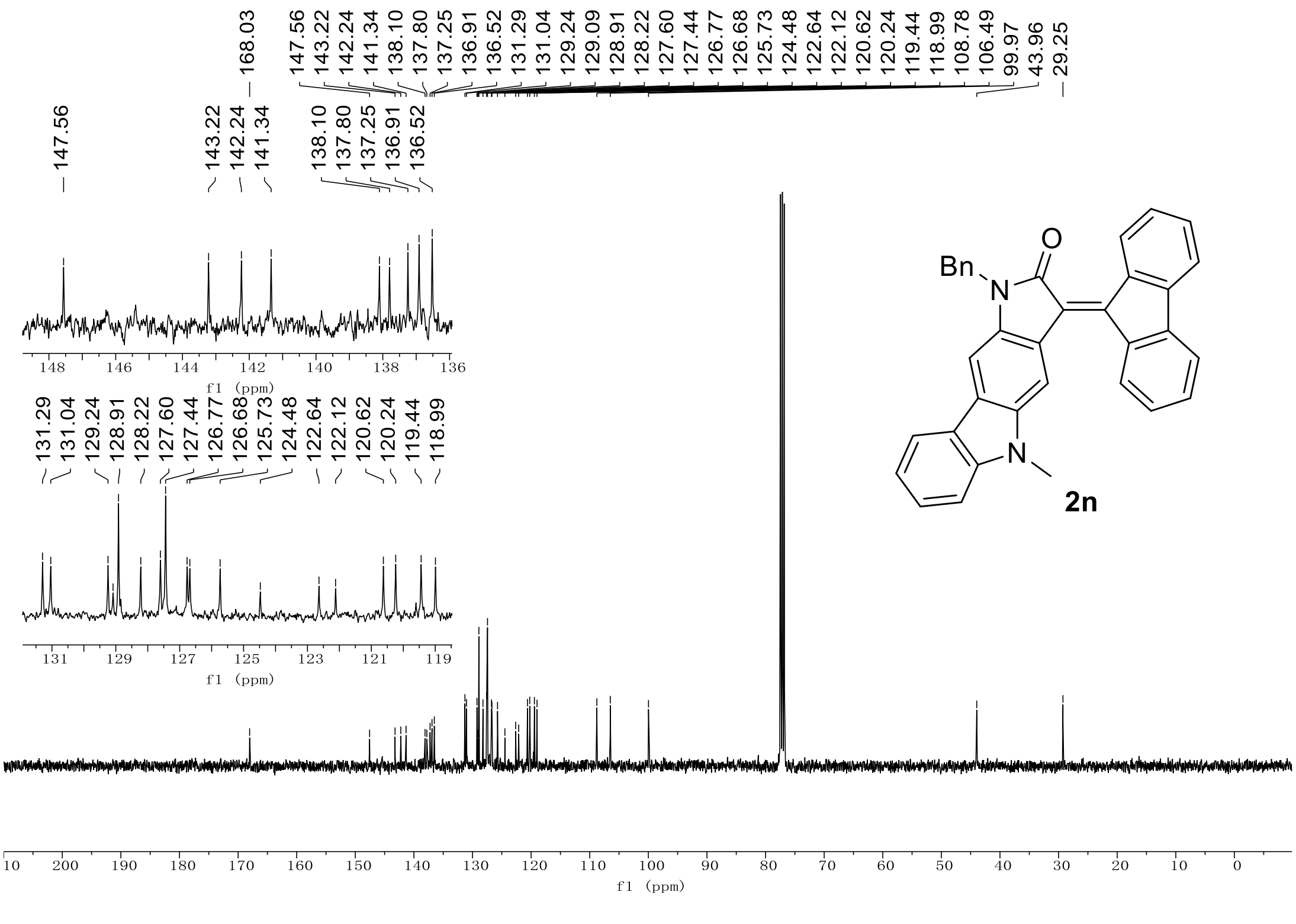

^1H NMR (400 MHz, $CDCl_3$)

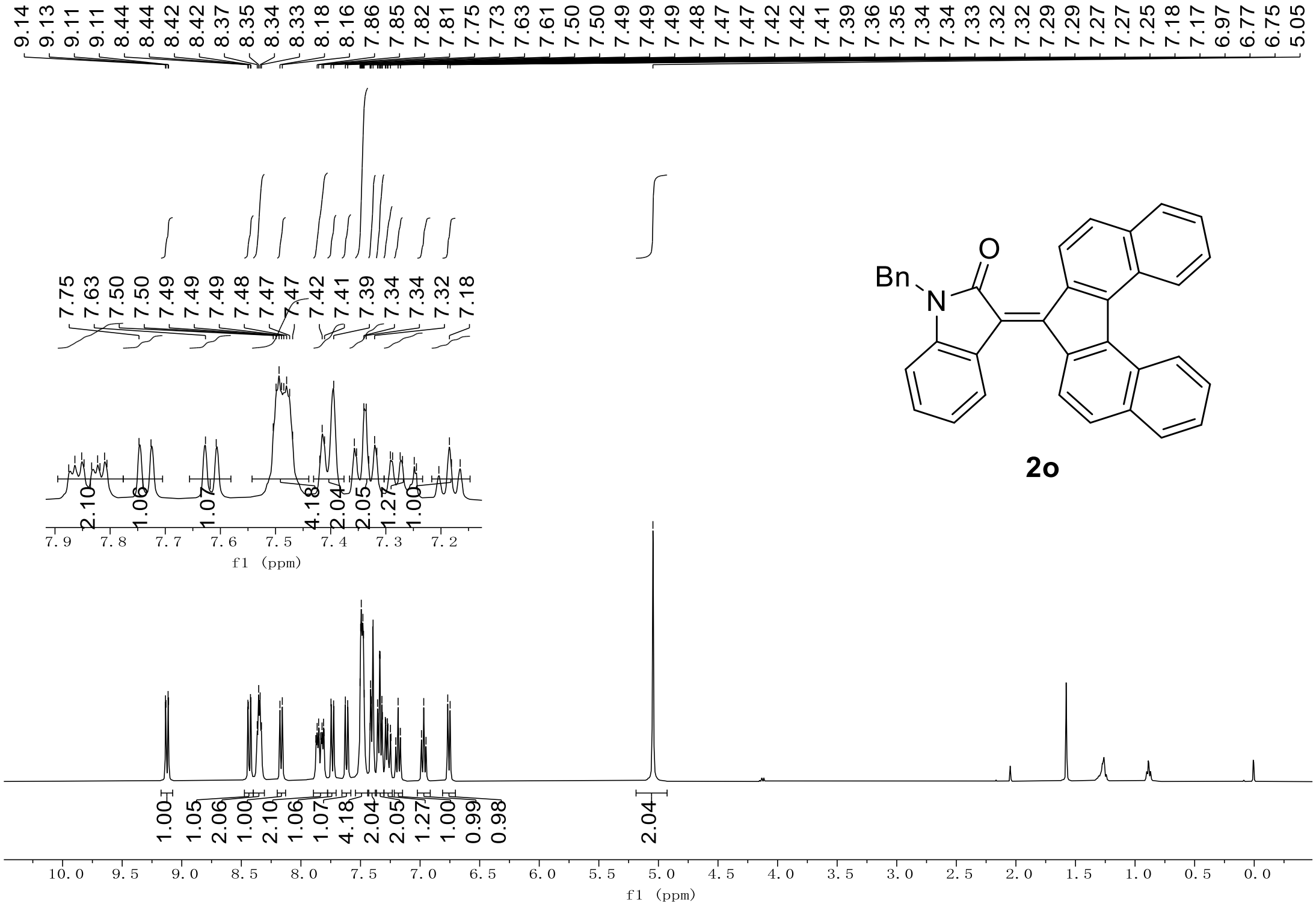

^{13}C NMR (100 MHz, $CDCl_3$)

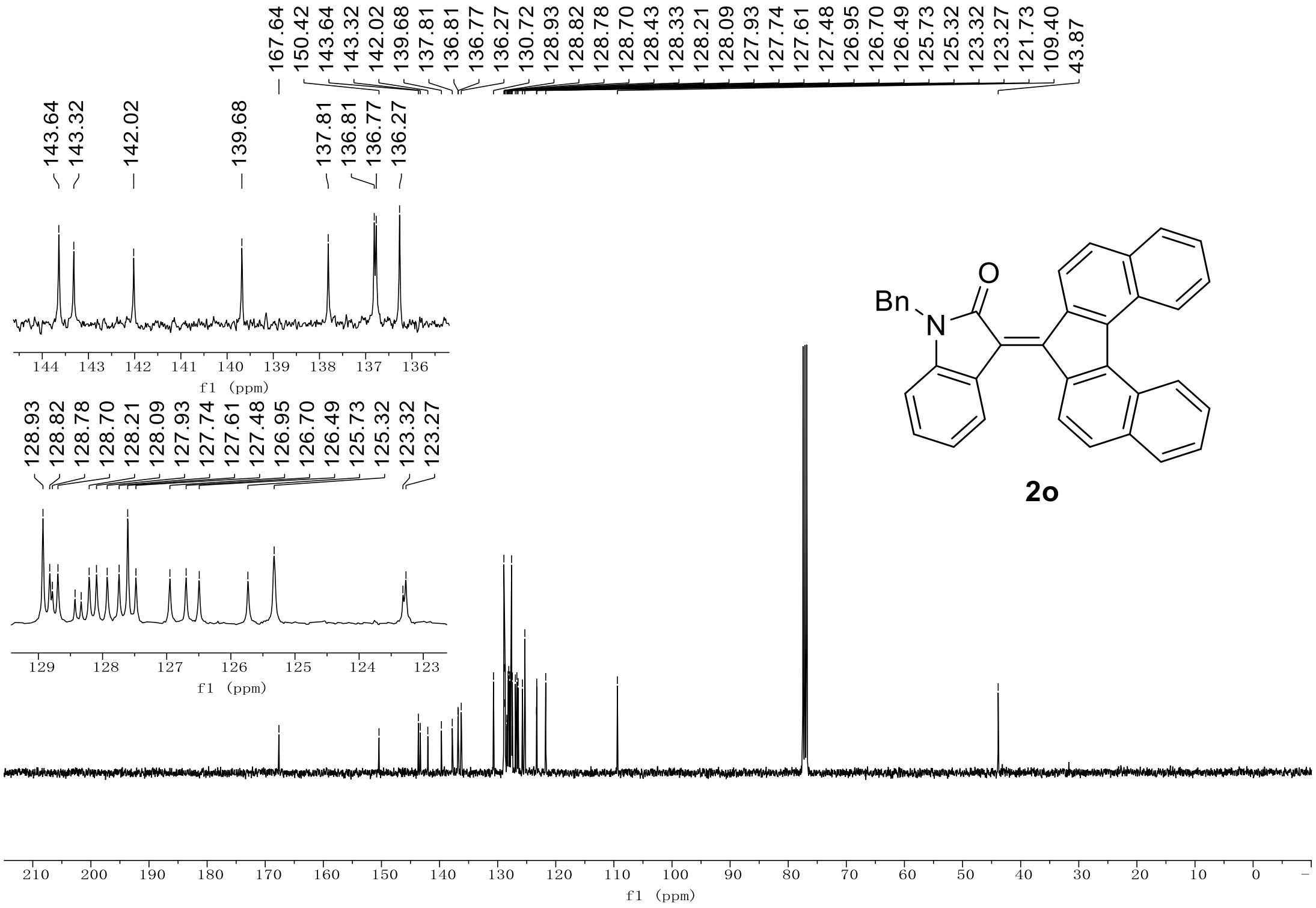

^{1}H NMR (400 MHz, CDCl$_3$)

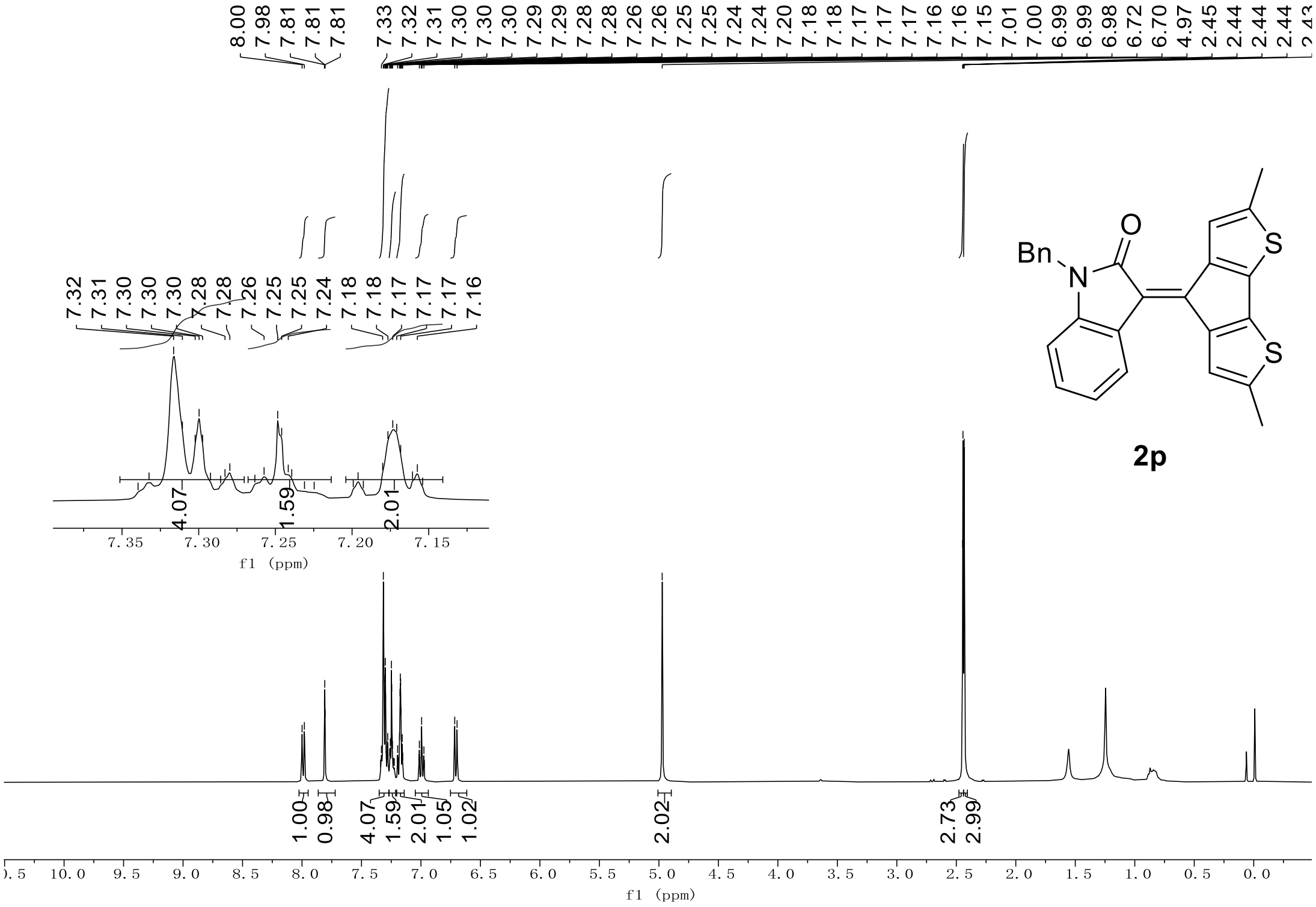

2p

^{13}C NMR (100 MHz, CDCl$_3$)

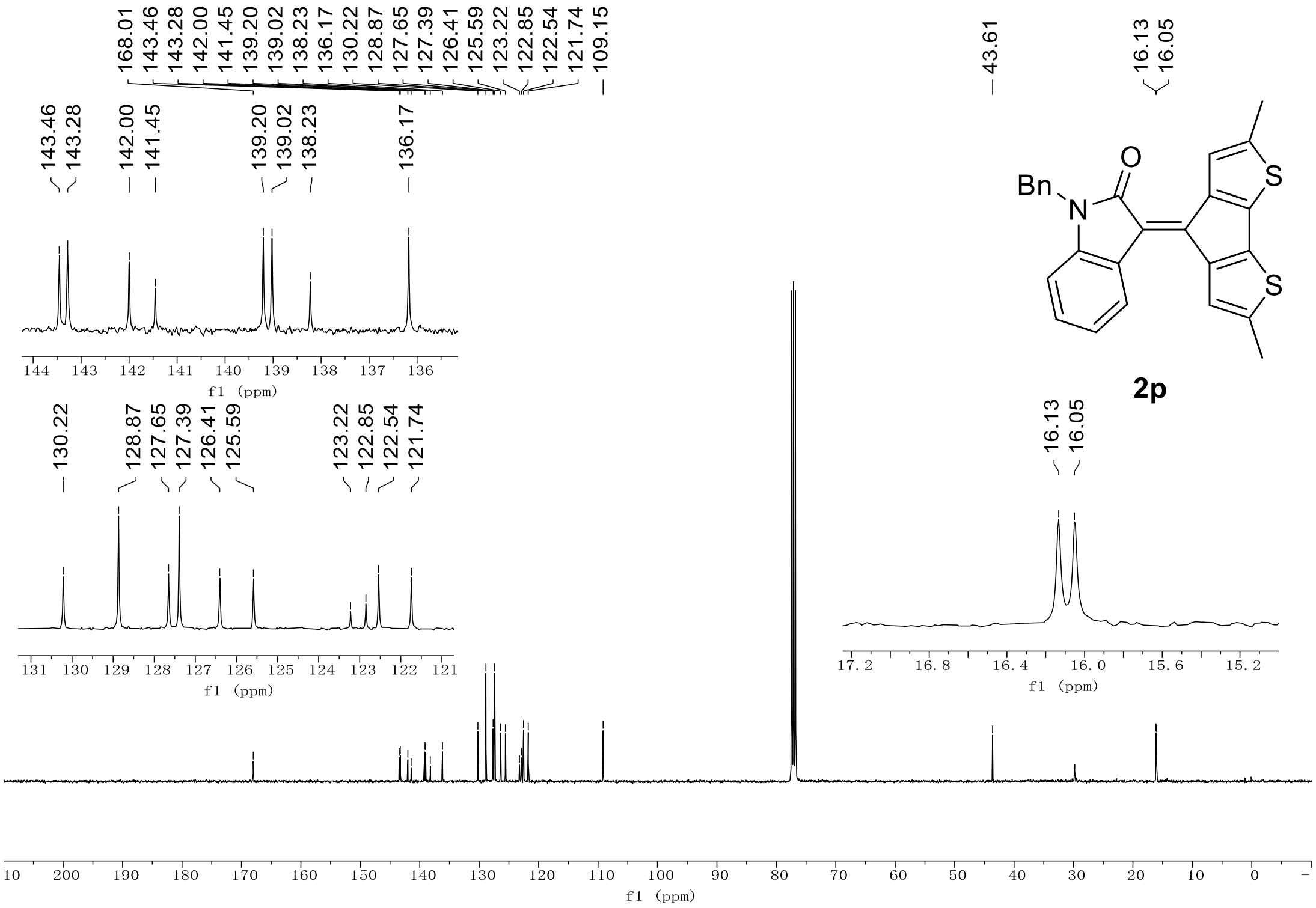

2p

^{1}H NMR (400 MHz, $CDCl_3$)

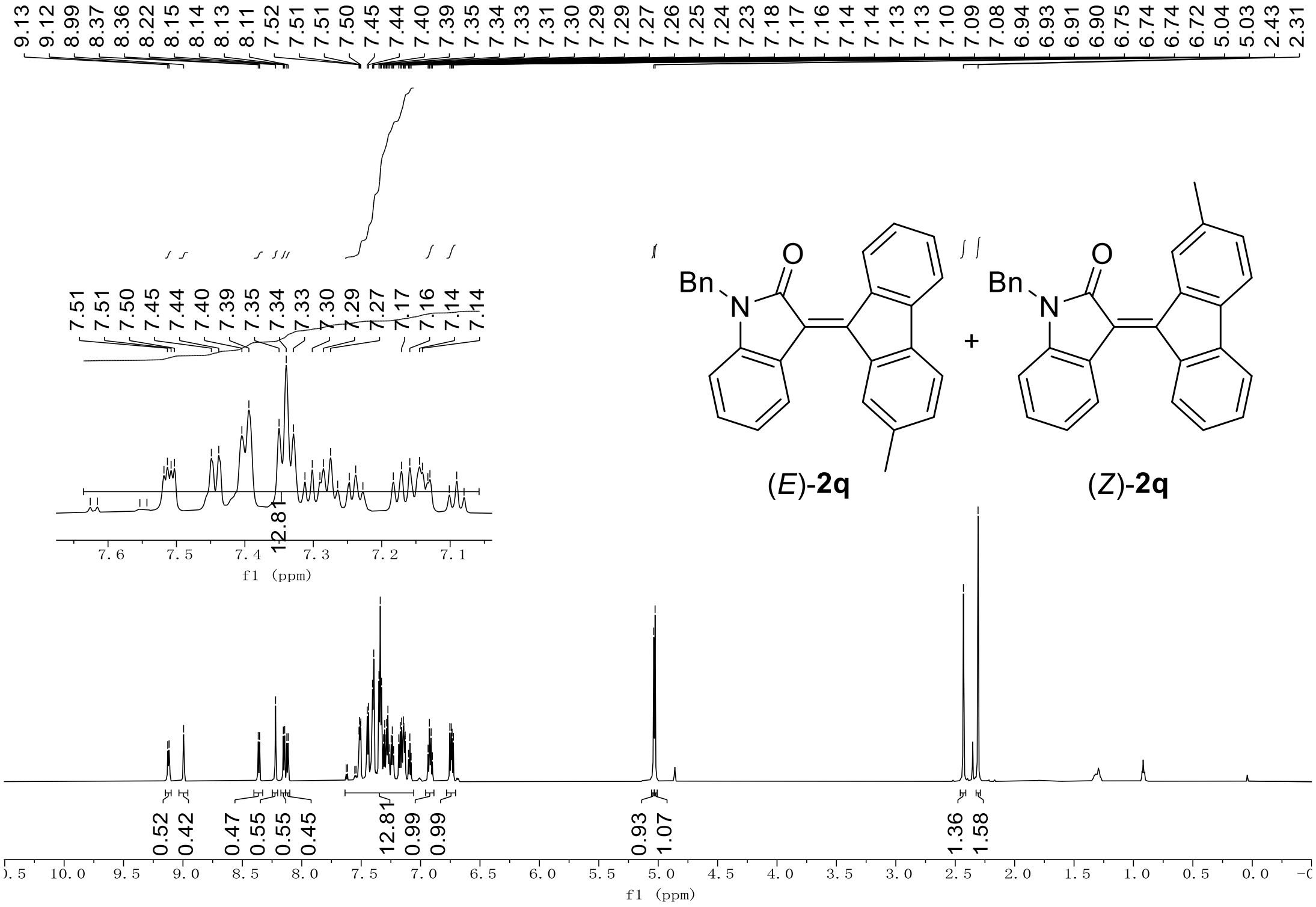

^{13}C NMR (100 MHz, $CDCl_3$)

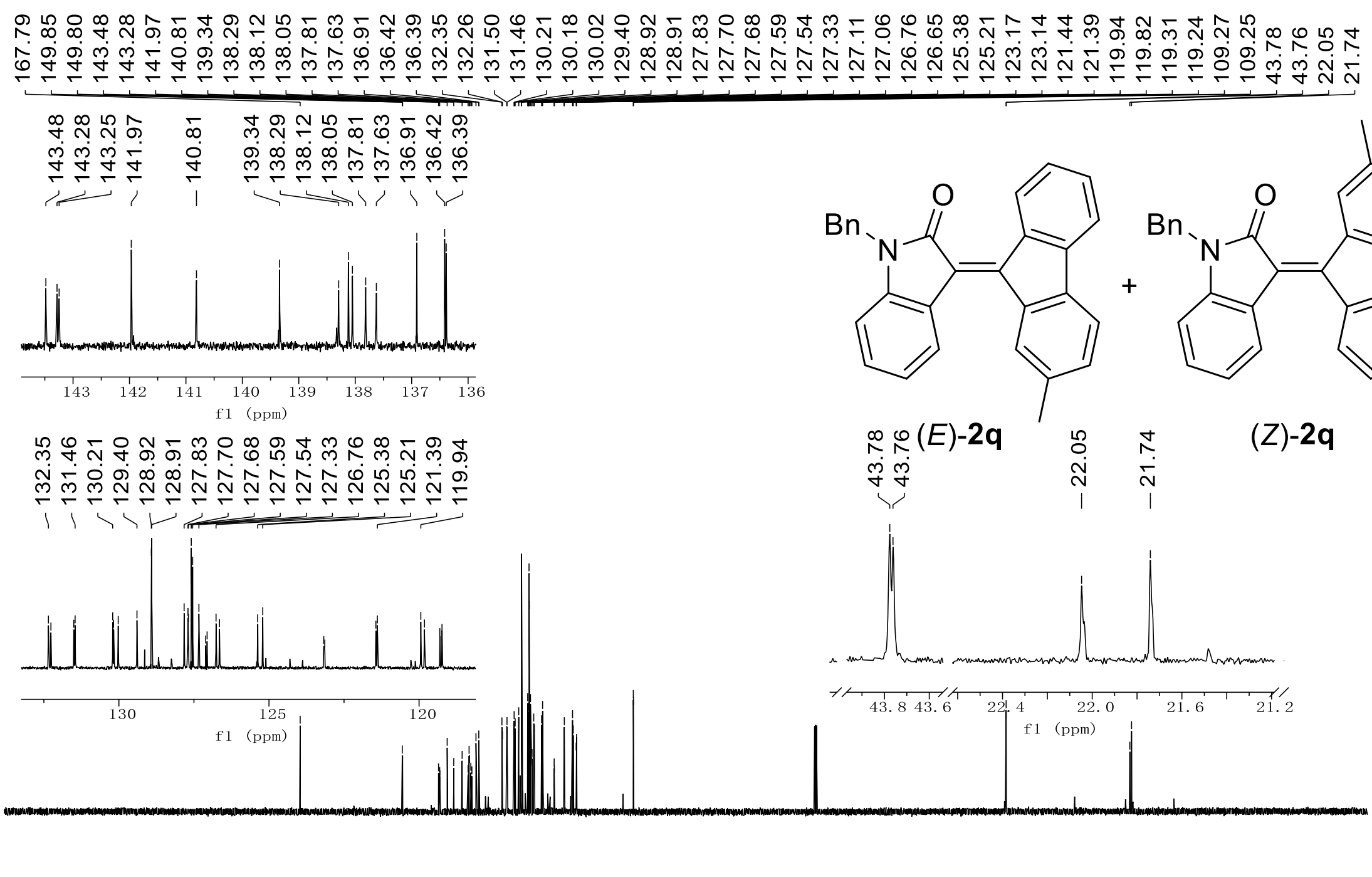

^1H–^1H DQF-COSY NMR (700 MHz, $CDCl_3$)

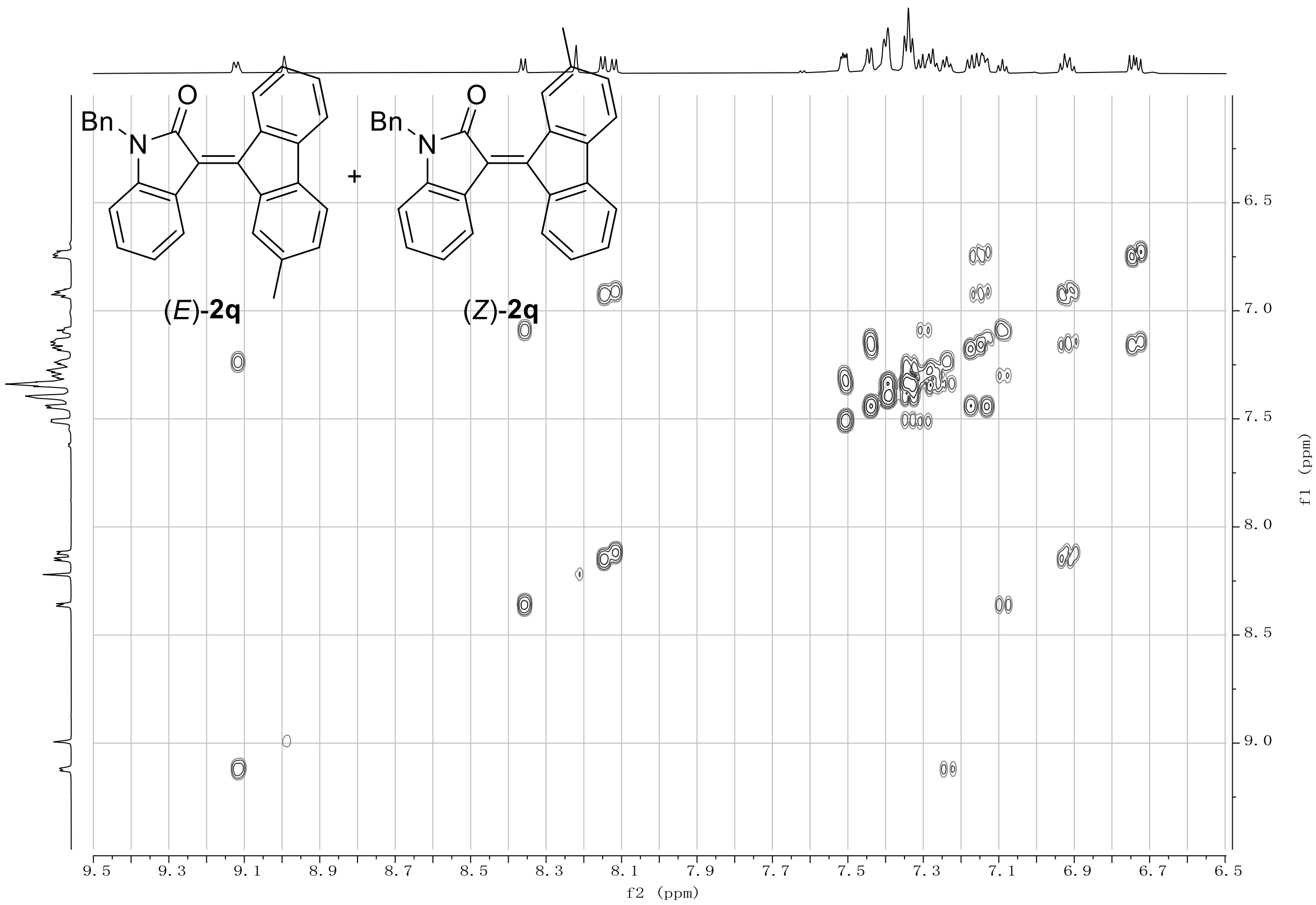

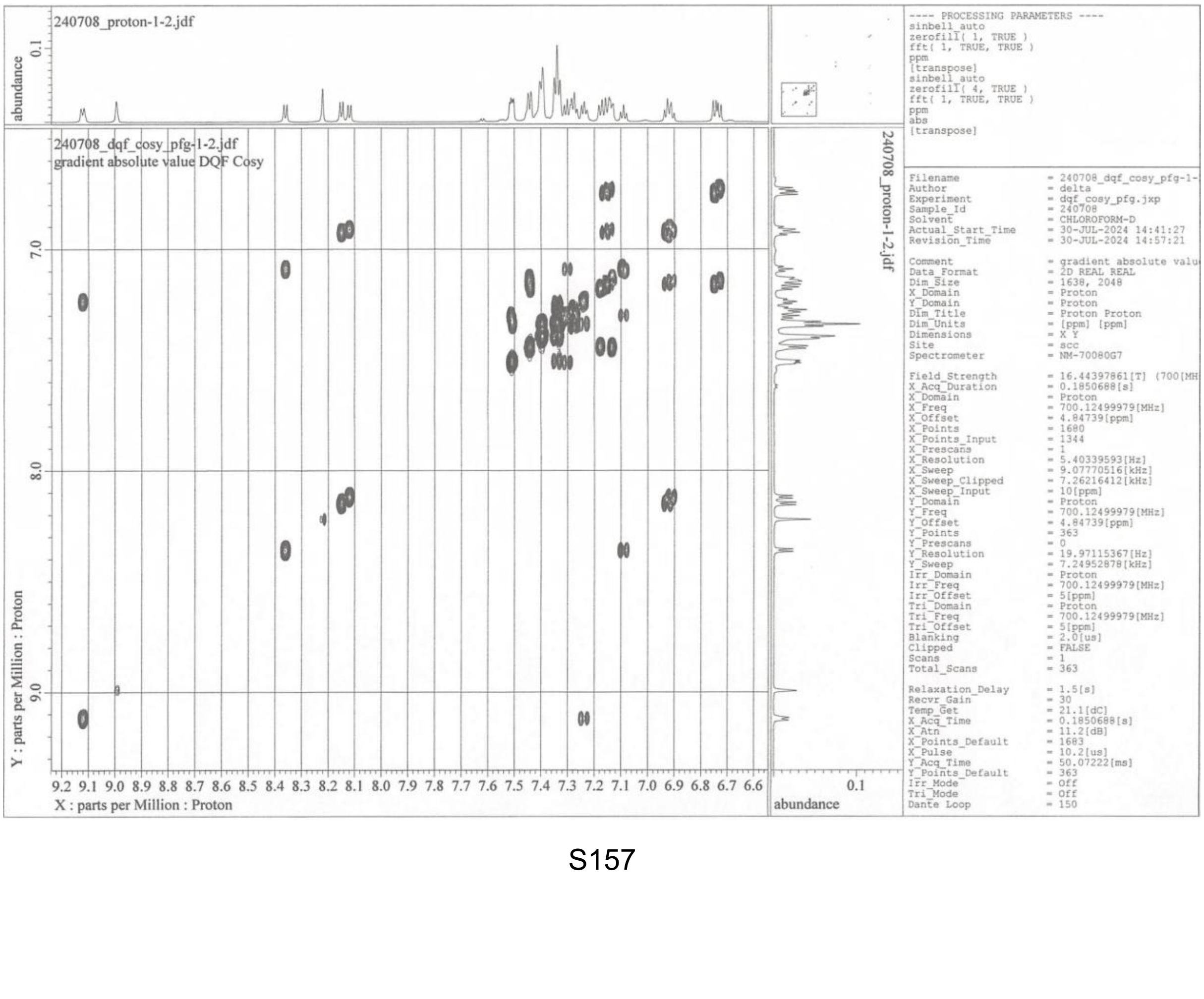

^{1}H–^{1}H TOCSY NMR (700 MHz, $CDCl_3$)

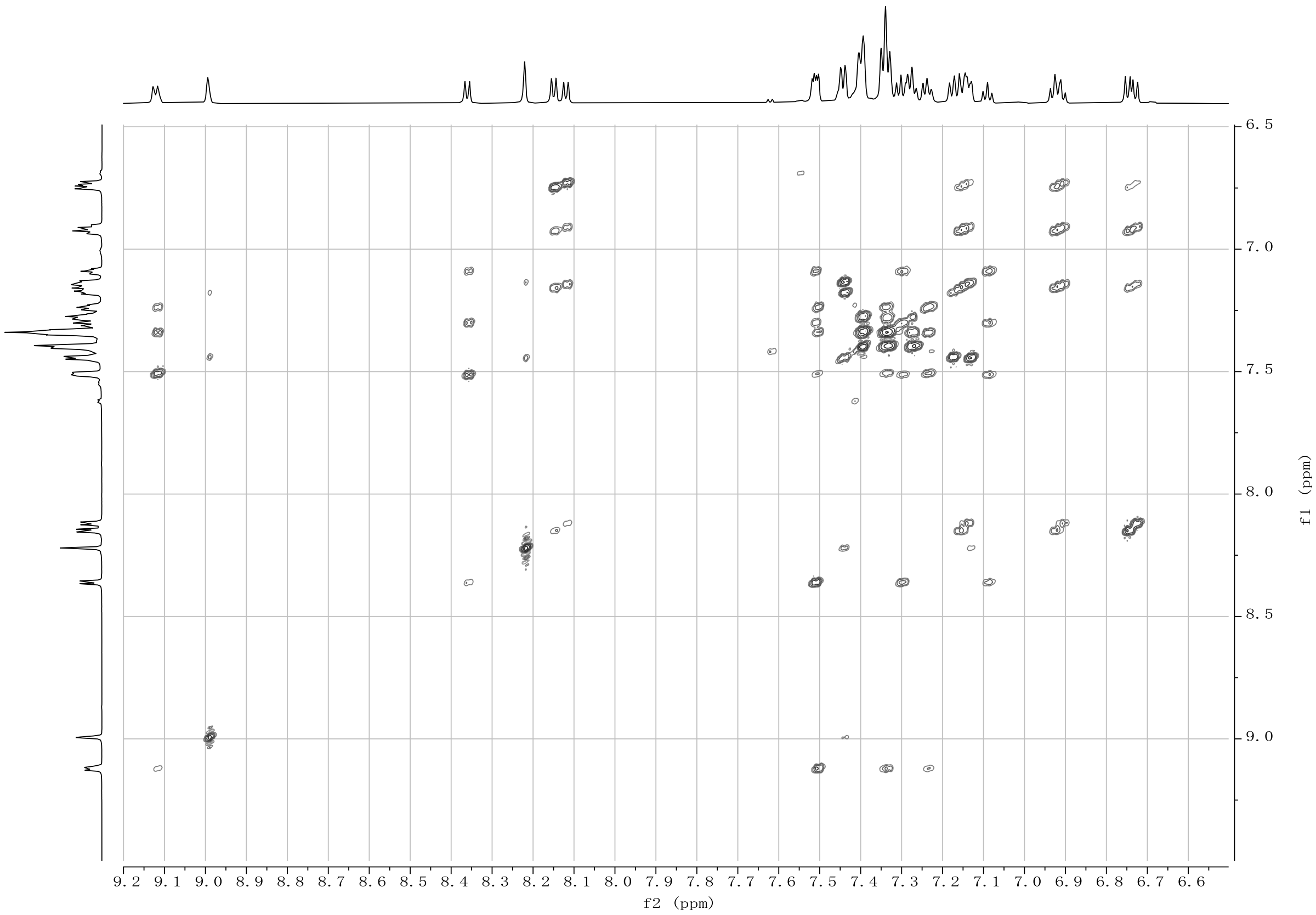

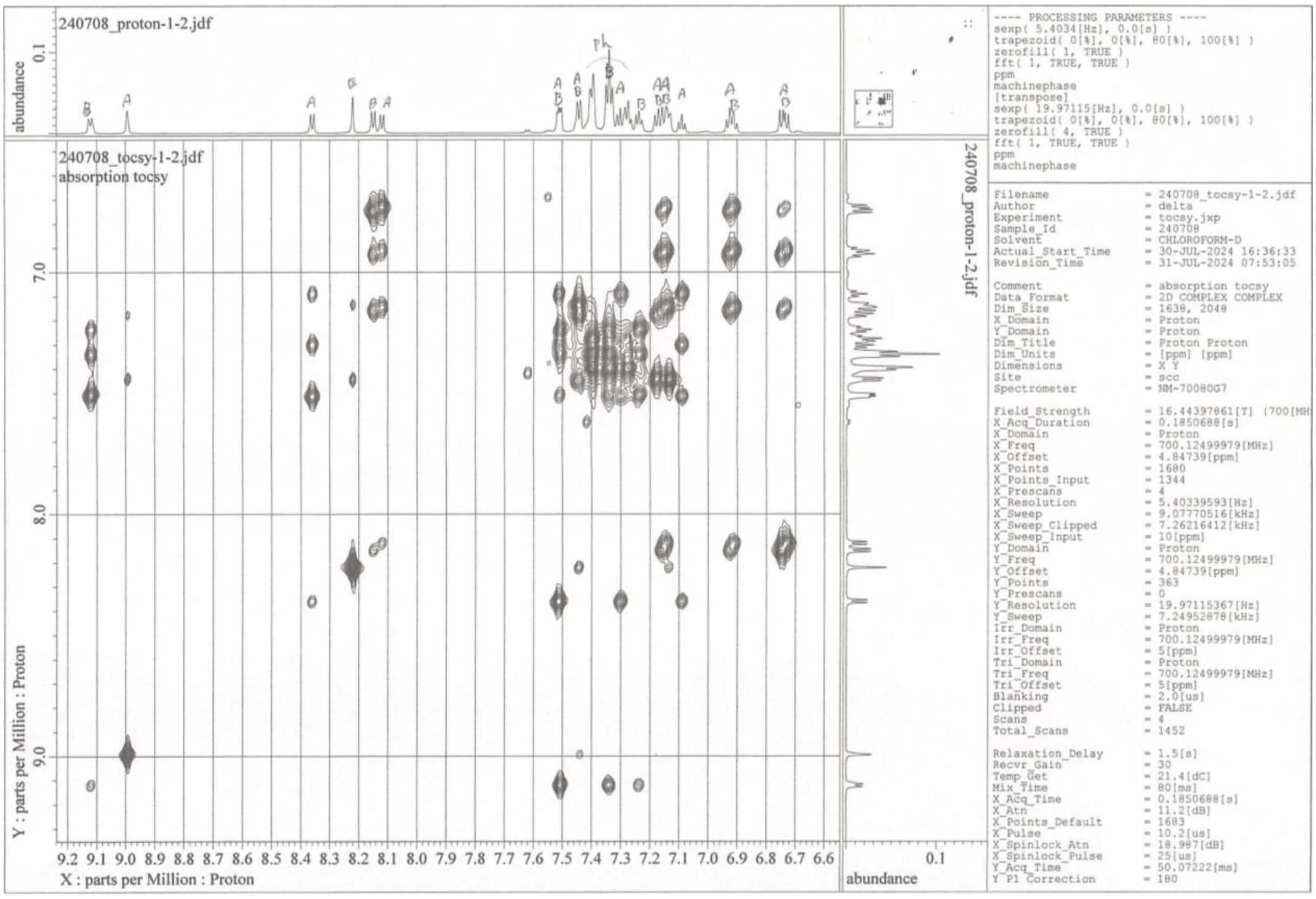

^{1}H 1D DPFGSE NOE difference NMR (700 MHz, $CDCl_3$)

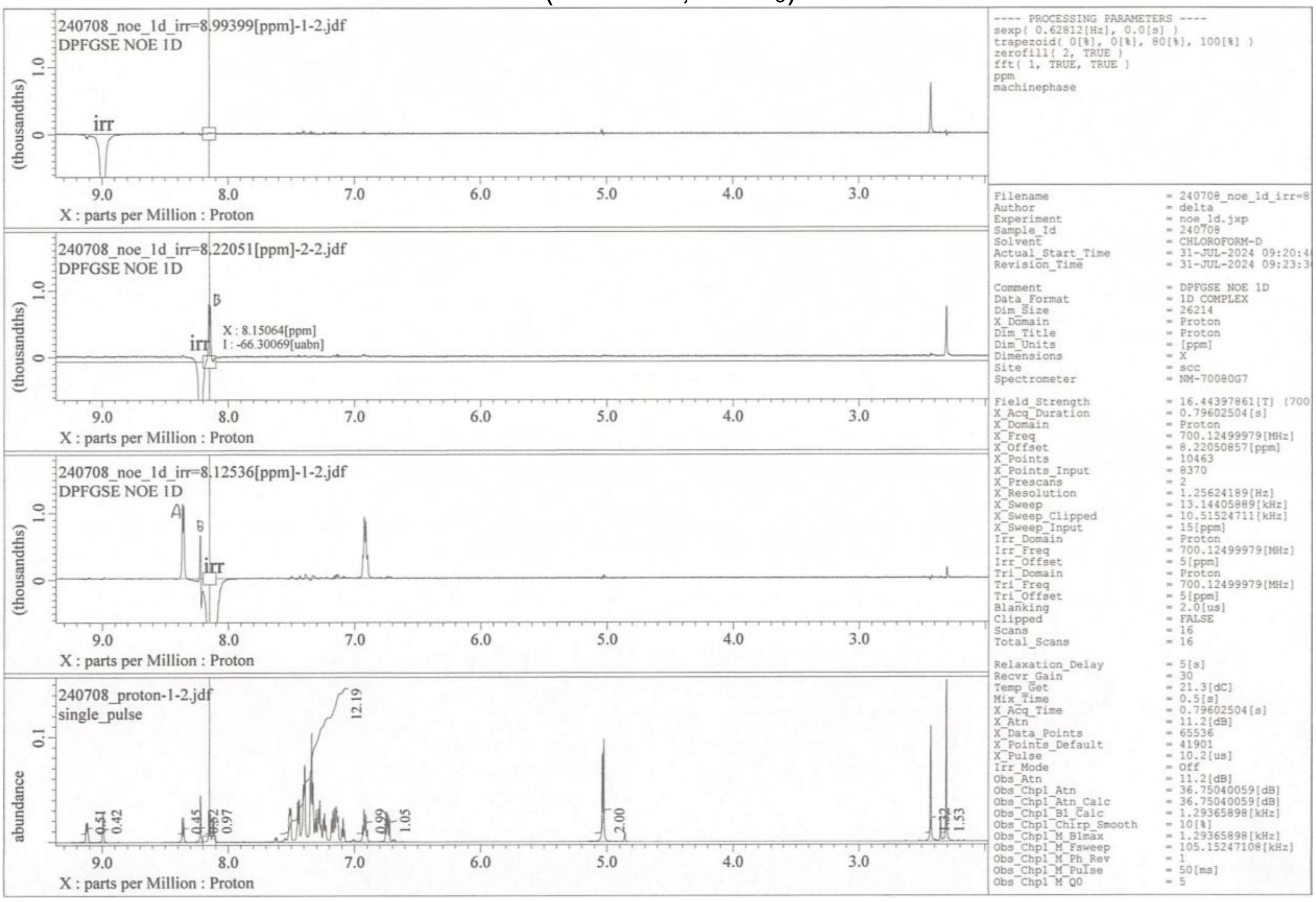

^{1}H 1D DPFGSE NOE difference NMR (700 MHz, $CDCl_3$)

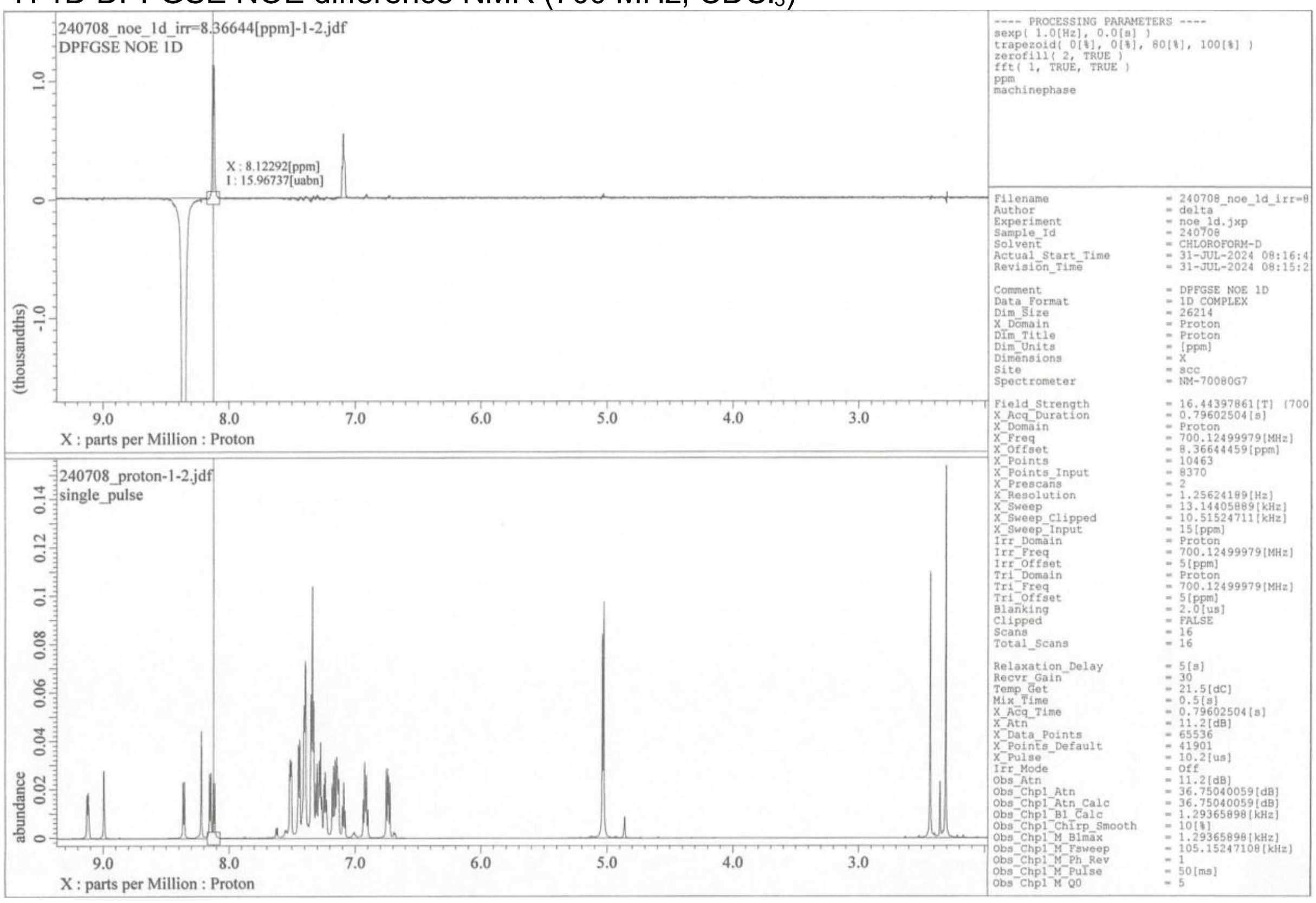

^{1}H 1D DPFGSE NOE difference NMR (700 MHz, $CDCl_3$)

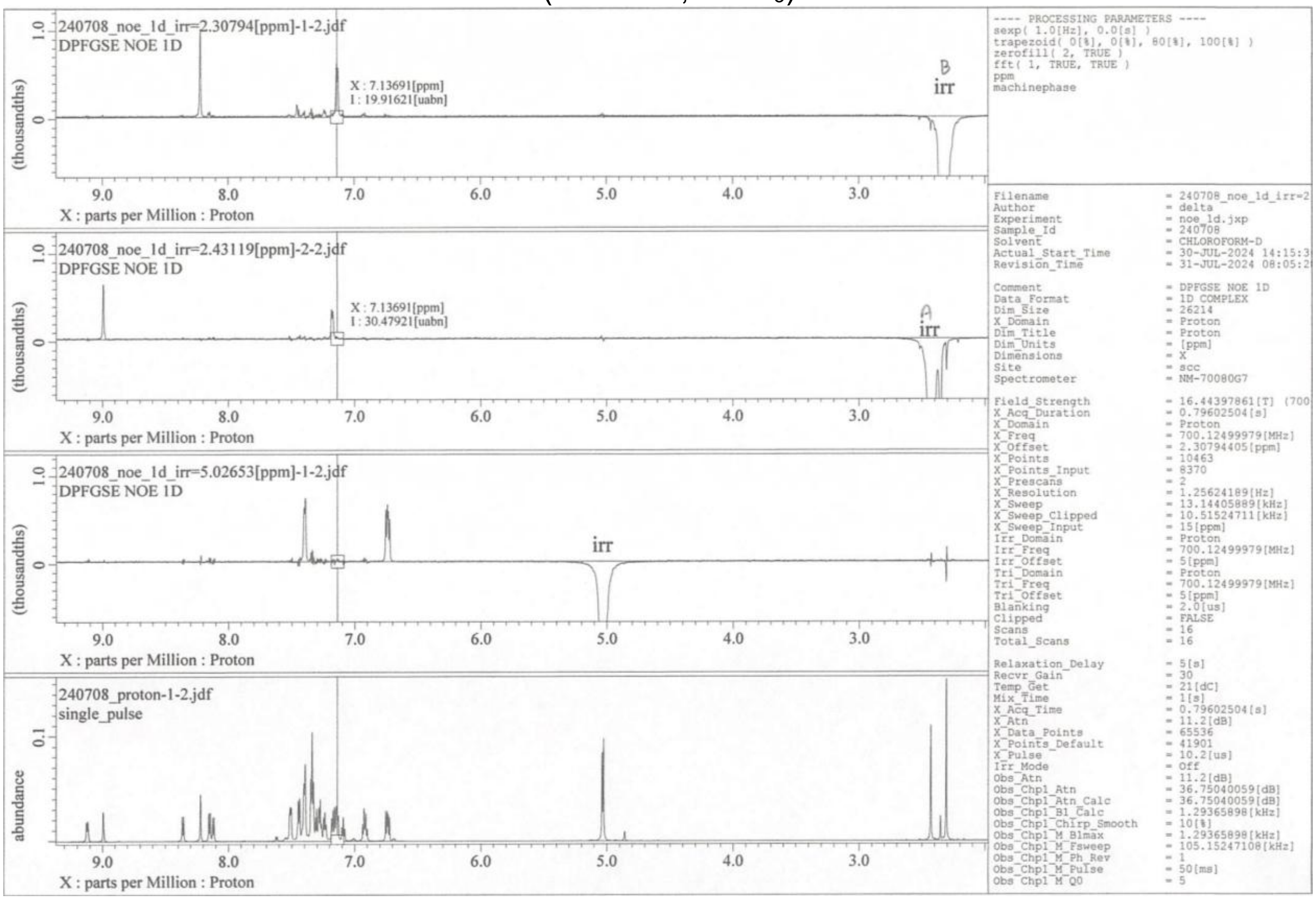

^{1}H NMR (400 MHz, $CDCl_3$)

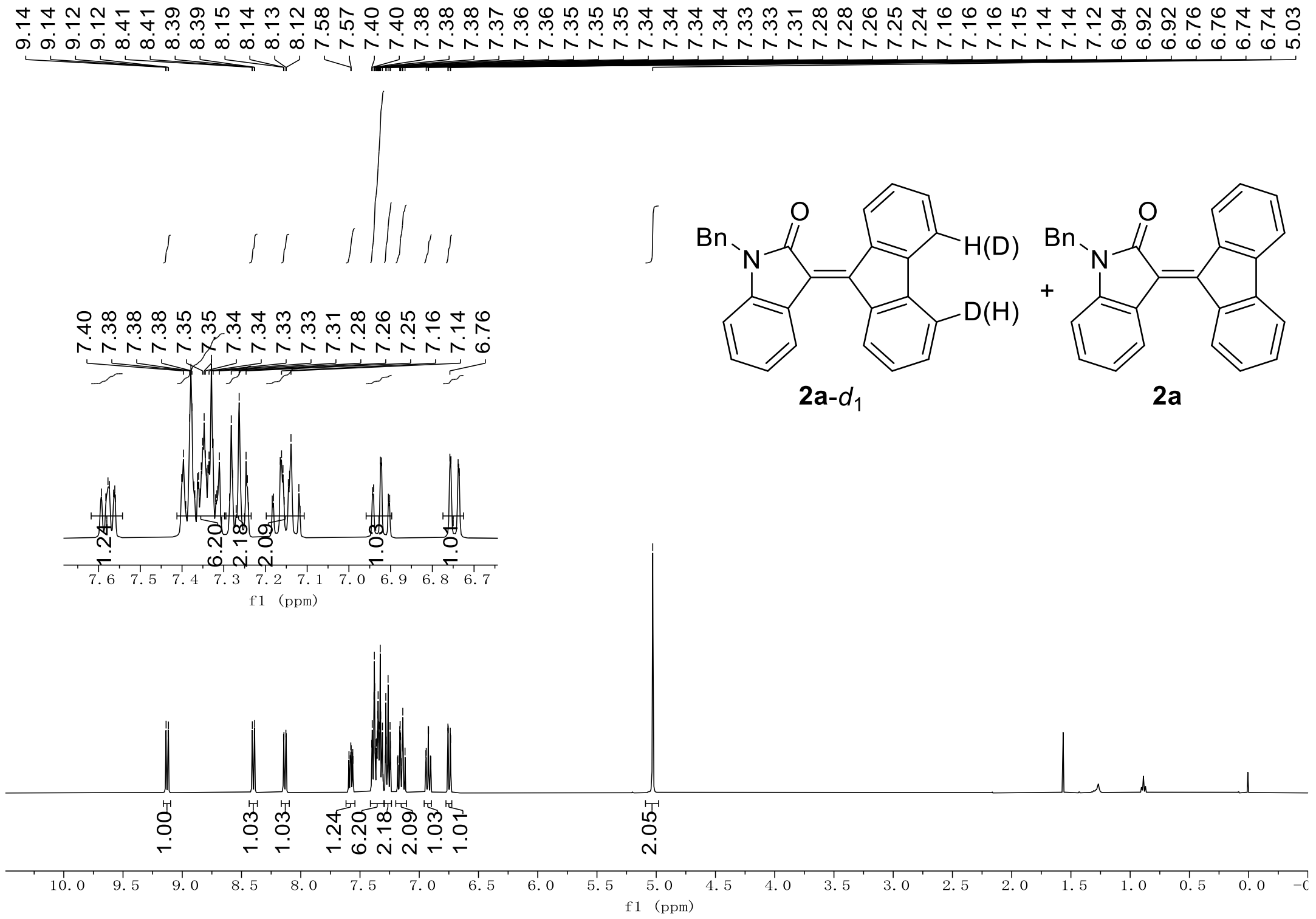

^{13}C NMR (100 MHz, $CDCl_3$)

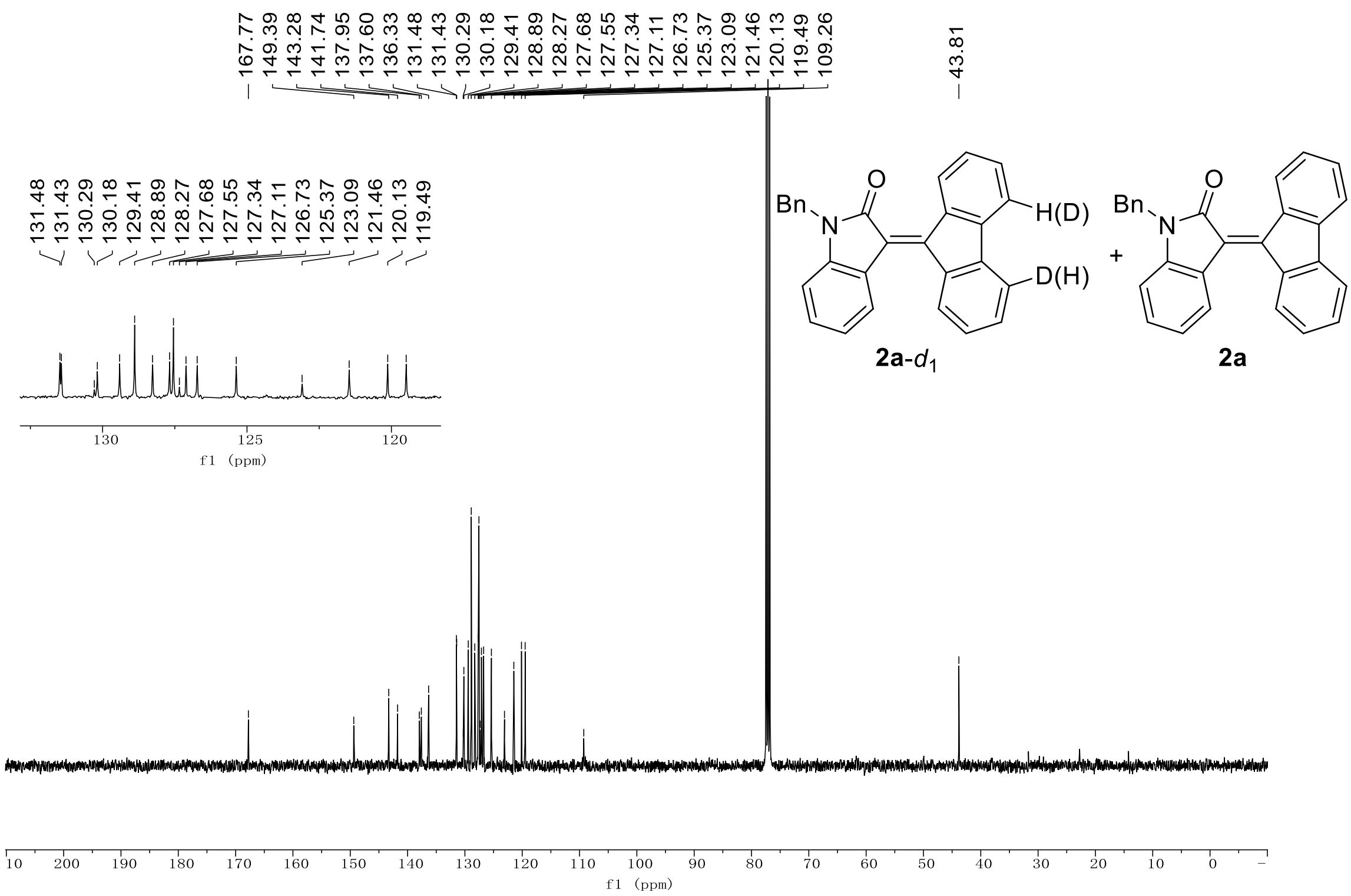

^1H NMR (400 MHz, $CDCl_3$)

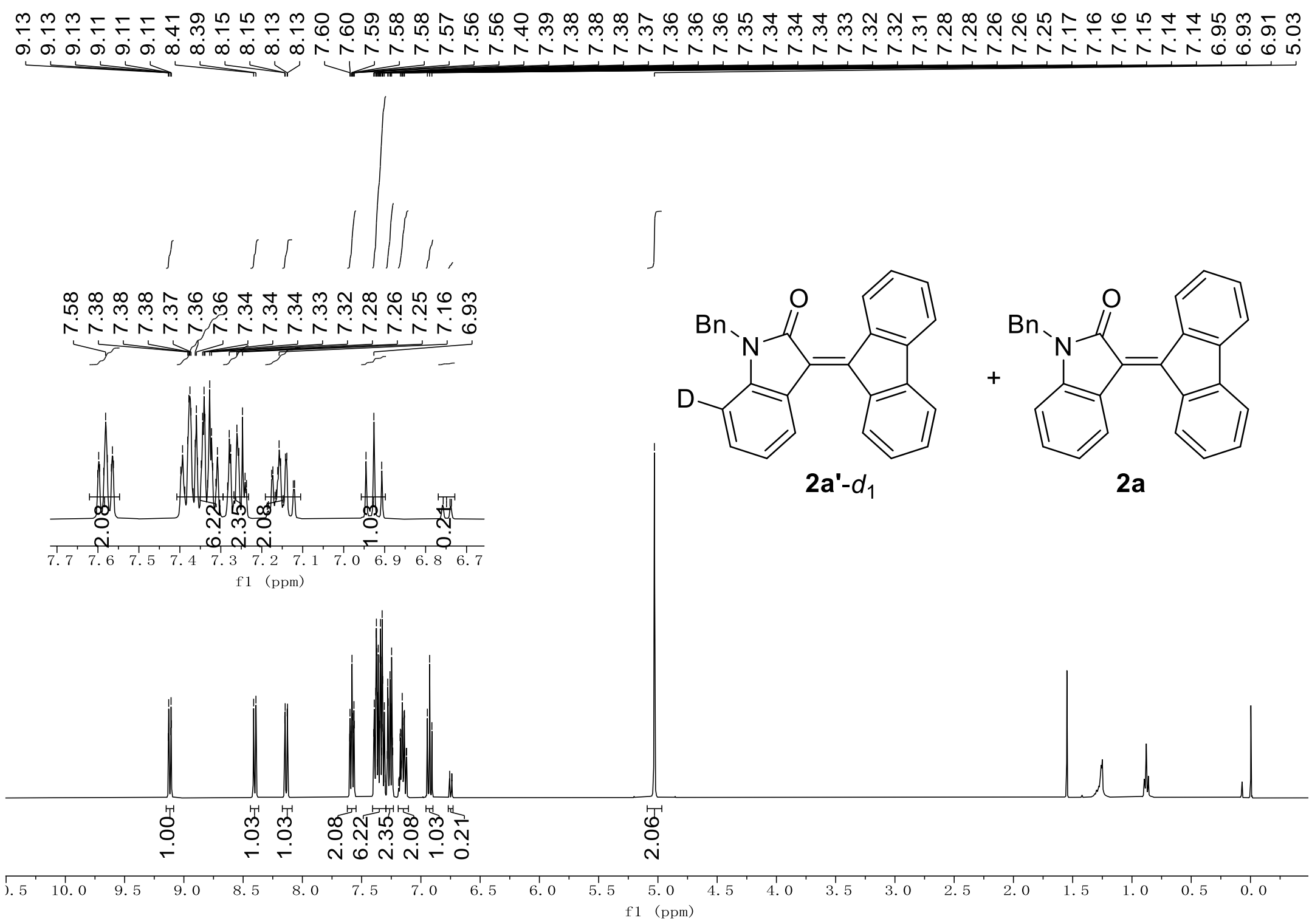

^{13}C NMR (100 MHz, $CDCl_3$)

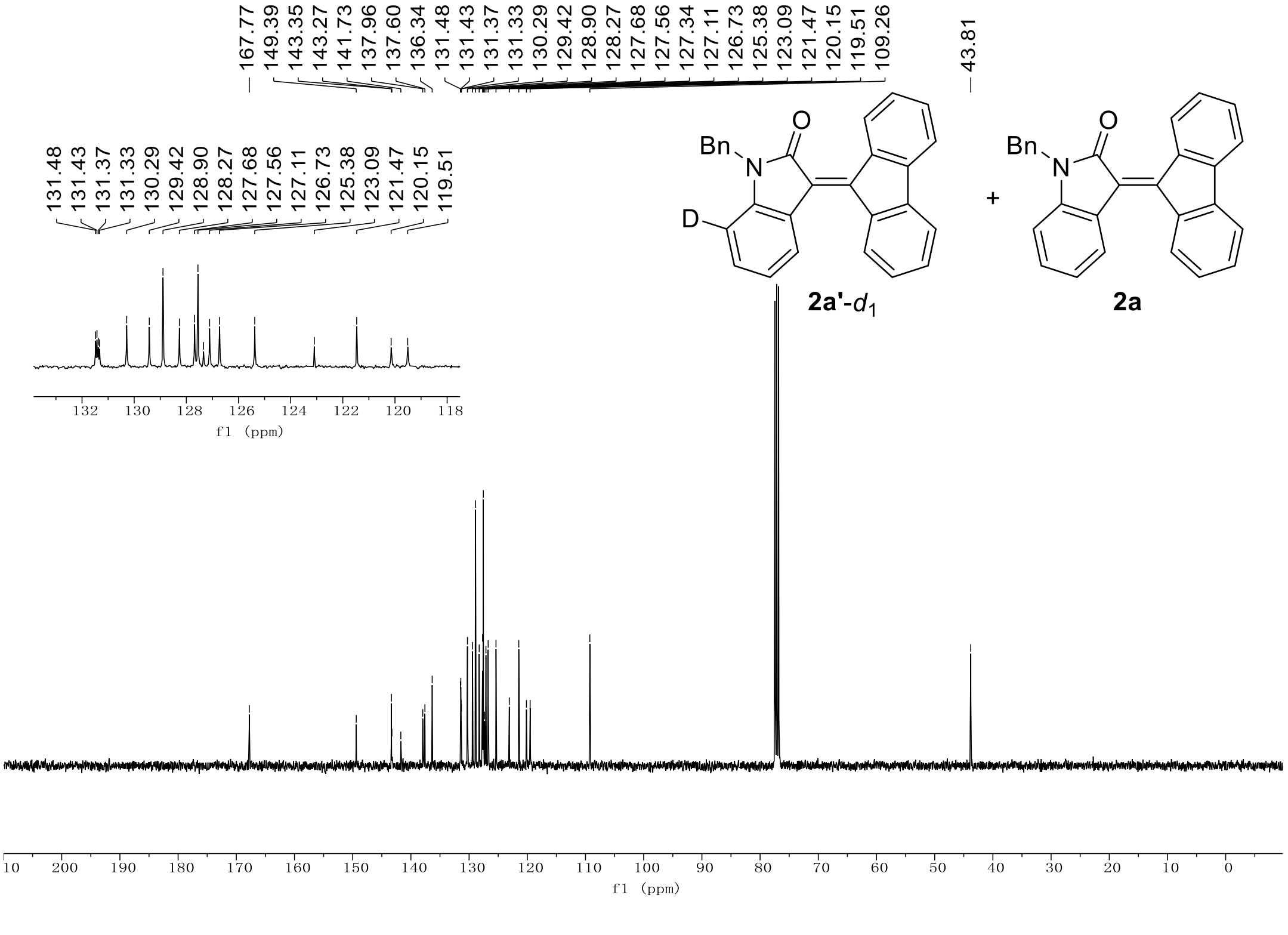

^{1}H NMR (400 MHz, $CDCl_3$)

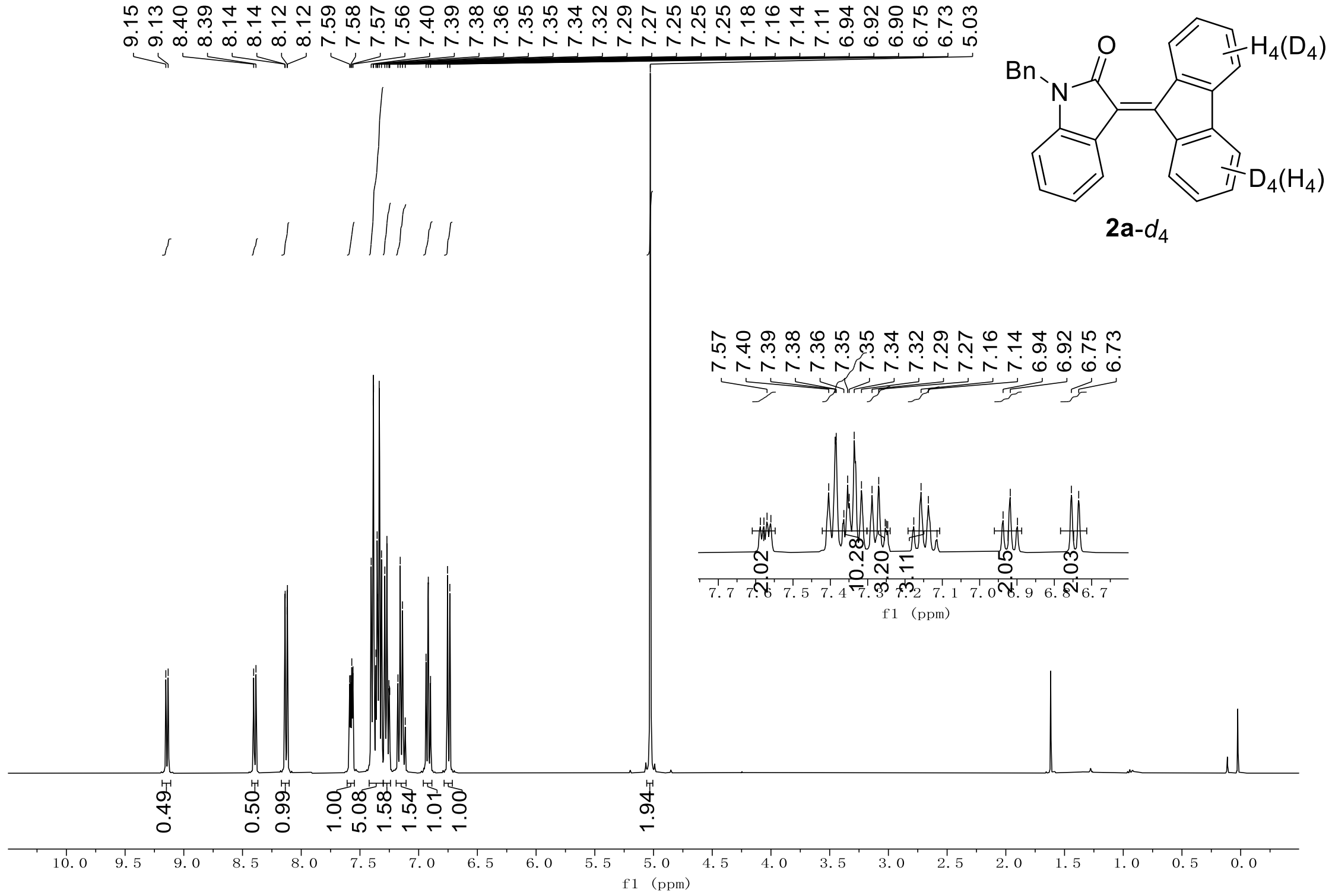

2a-d_4

^{13}C NMR (100 MHz, $CDCl_3$)

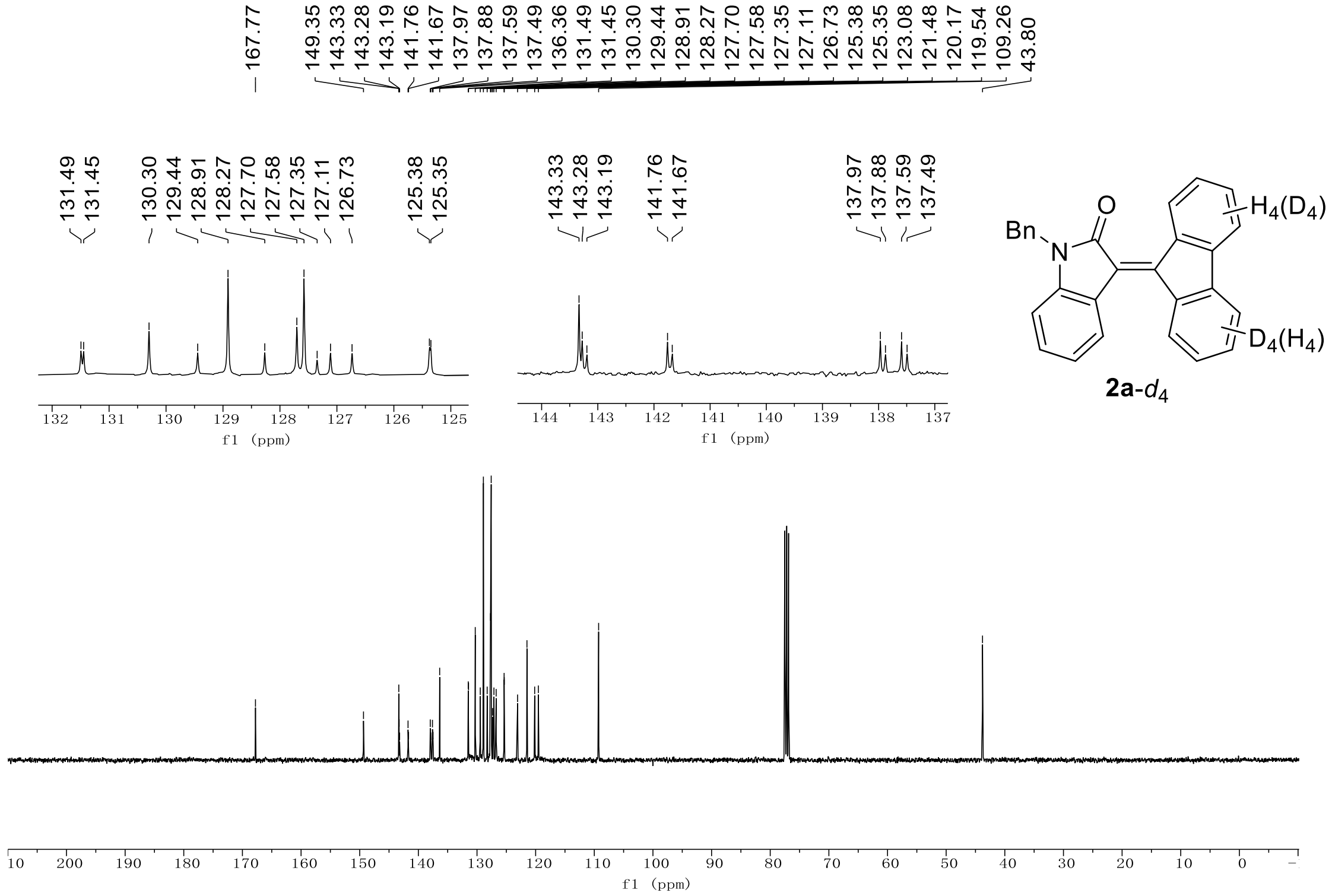

2a-d_4

^{1}H NMR (400 MHz, $CDCl_3$)

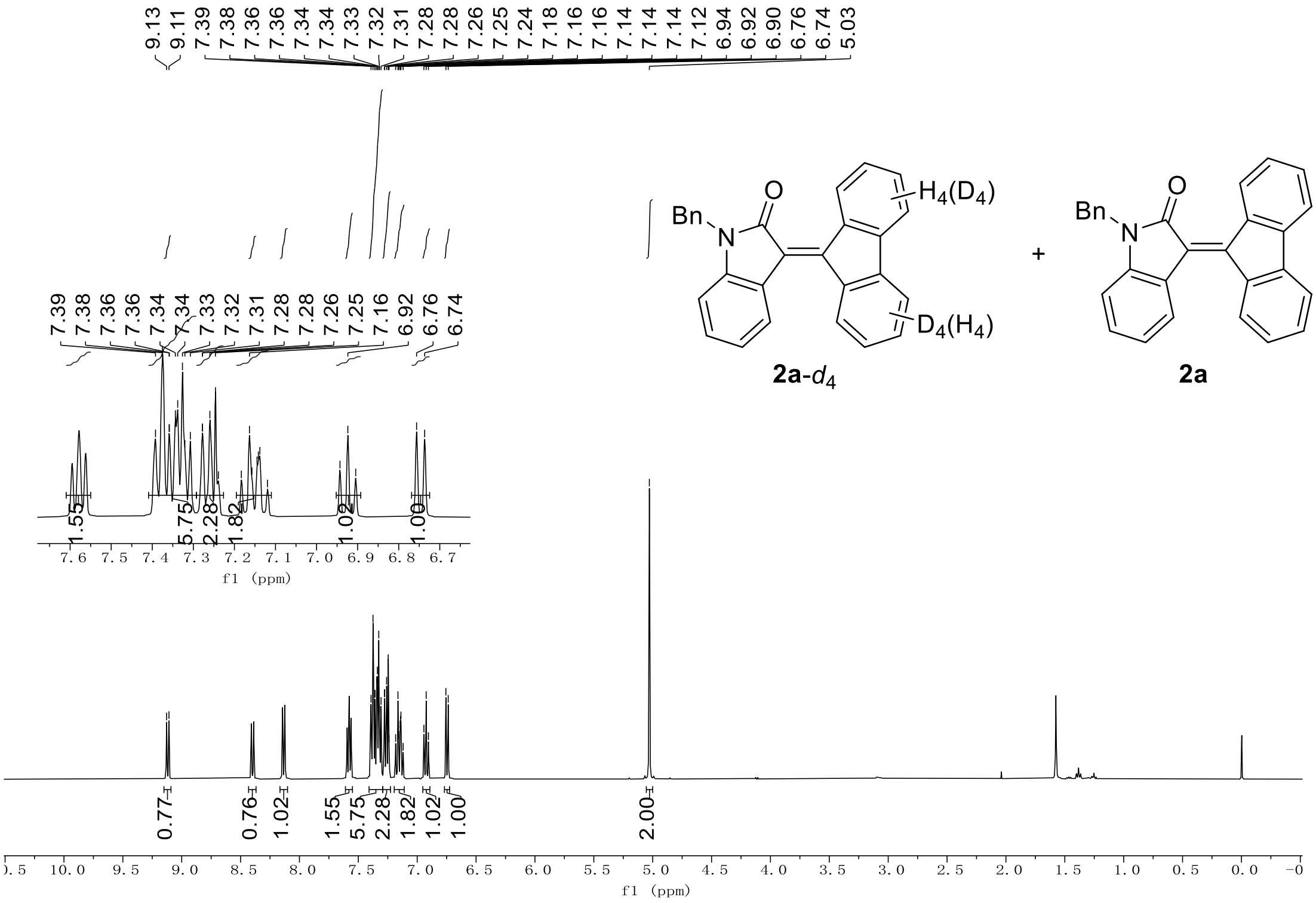

^1H NMR (400 MHz, $CDCl_3$)

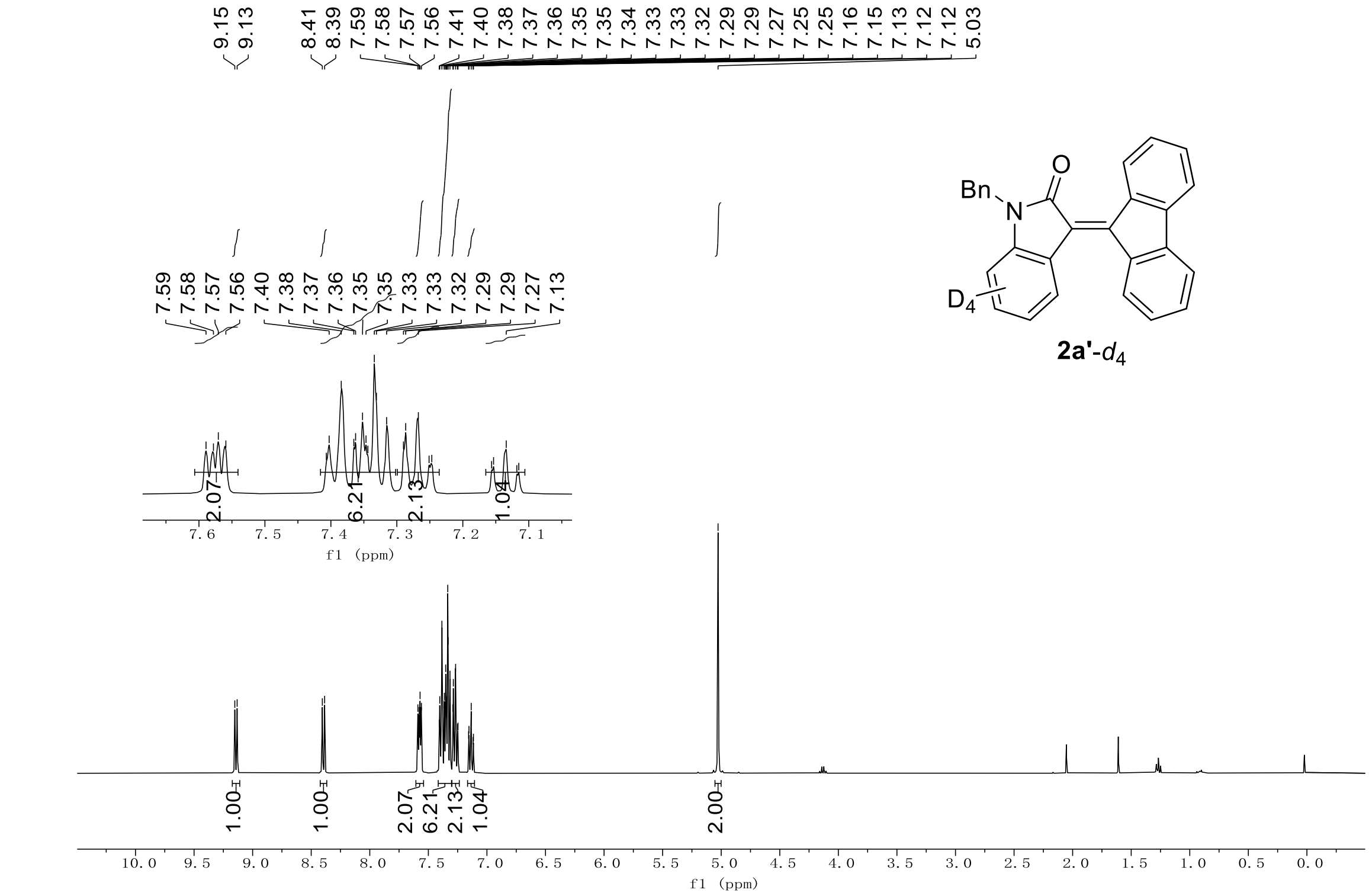

^{13}C NMR (100 MHz, $CDCl_3$)

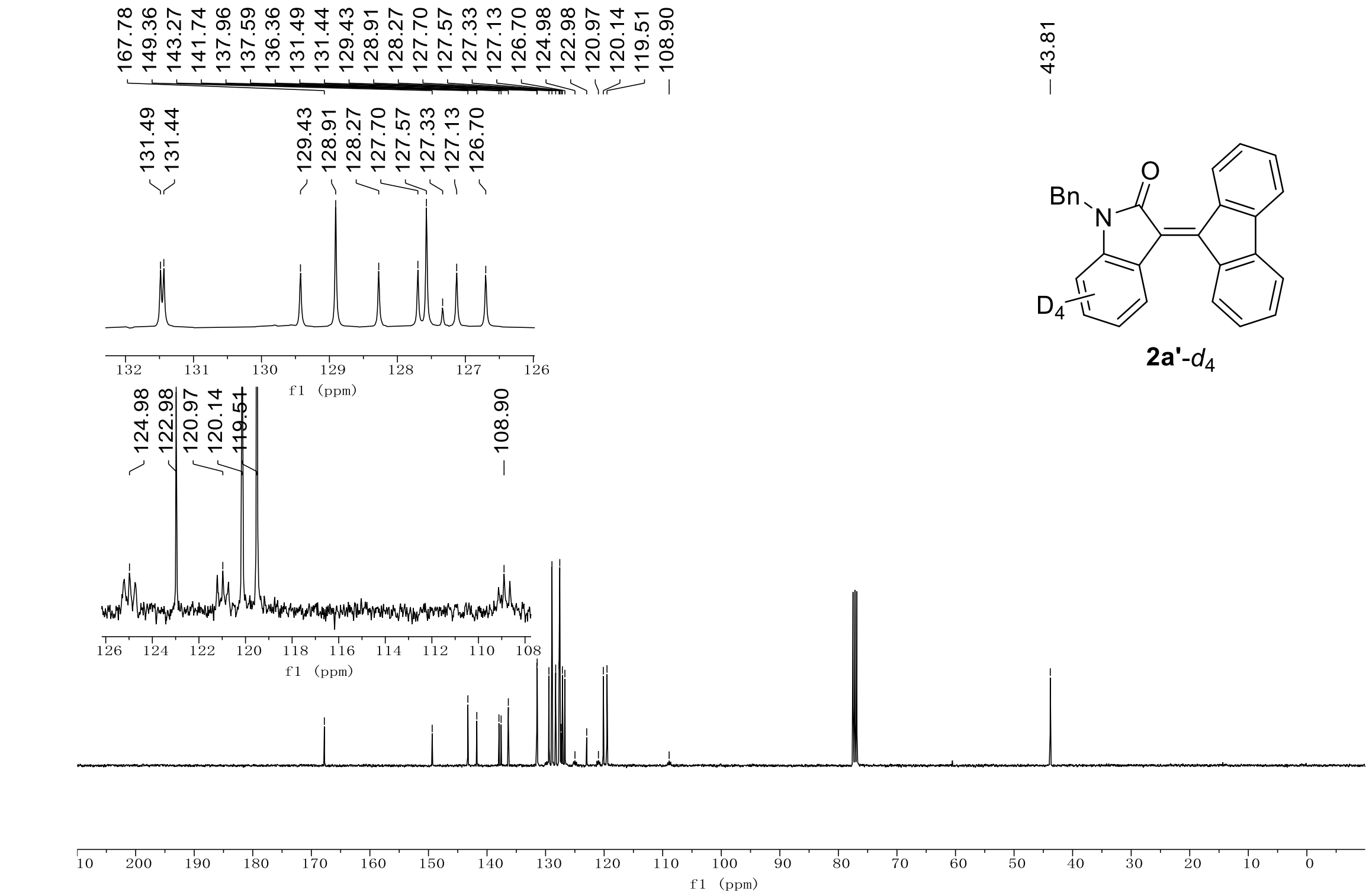

^{1}H NMR (400 MHz, $CDCl_3$)

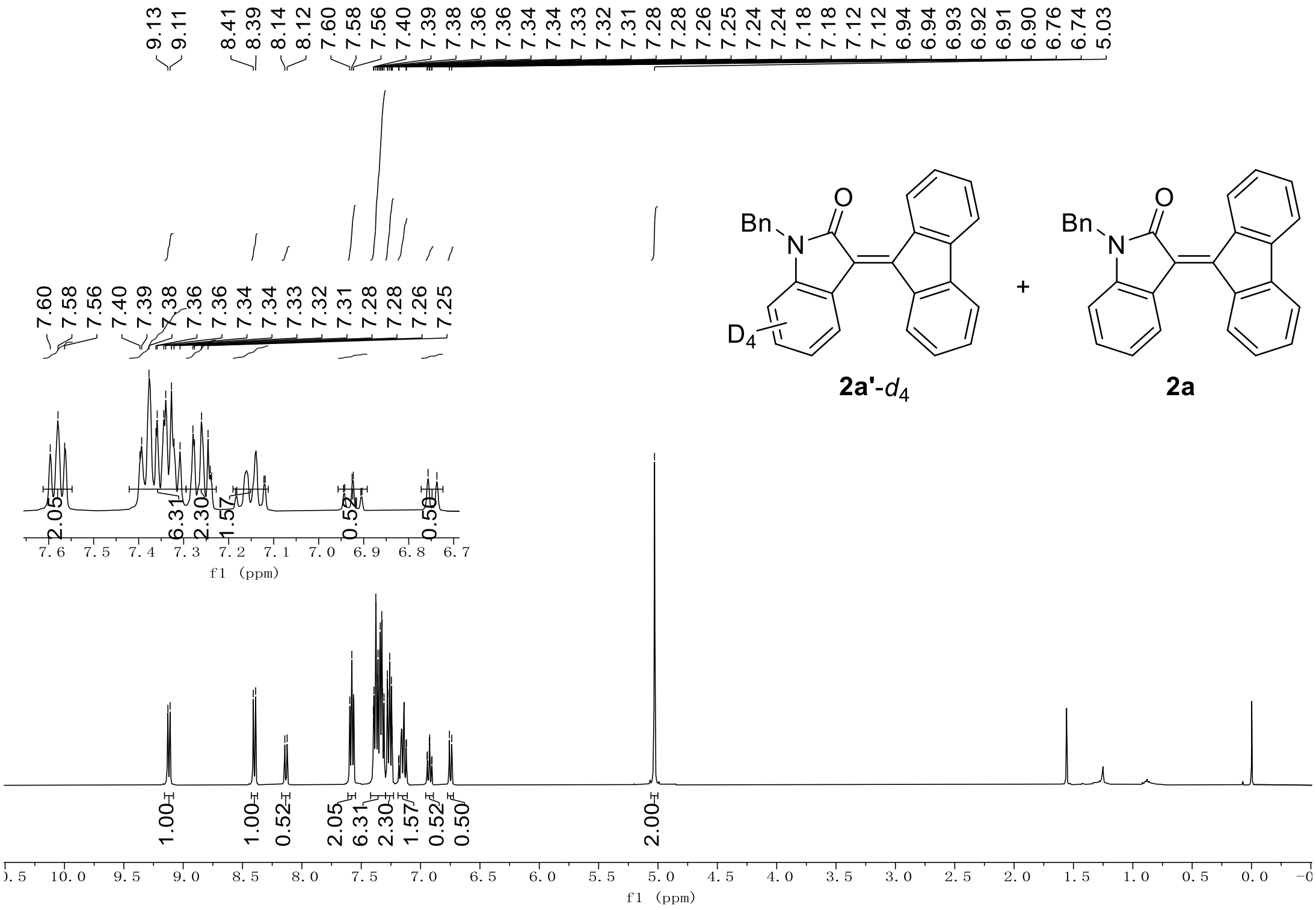

^{1}H NMR (700 MHz, $CDCl_3$)

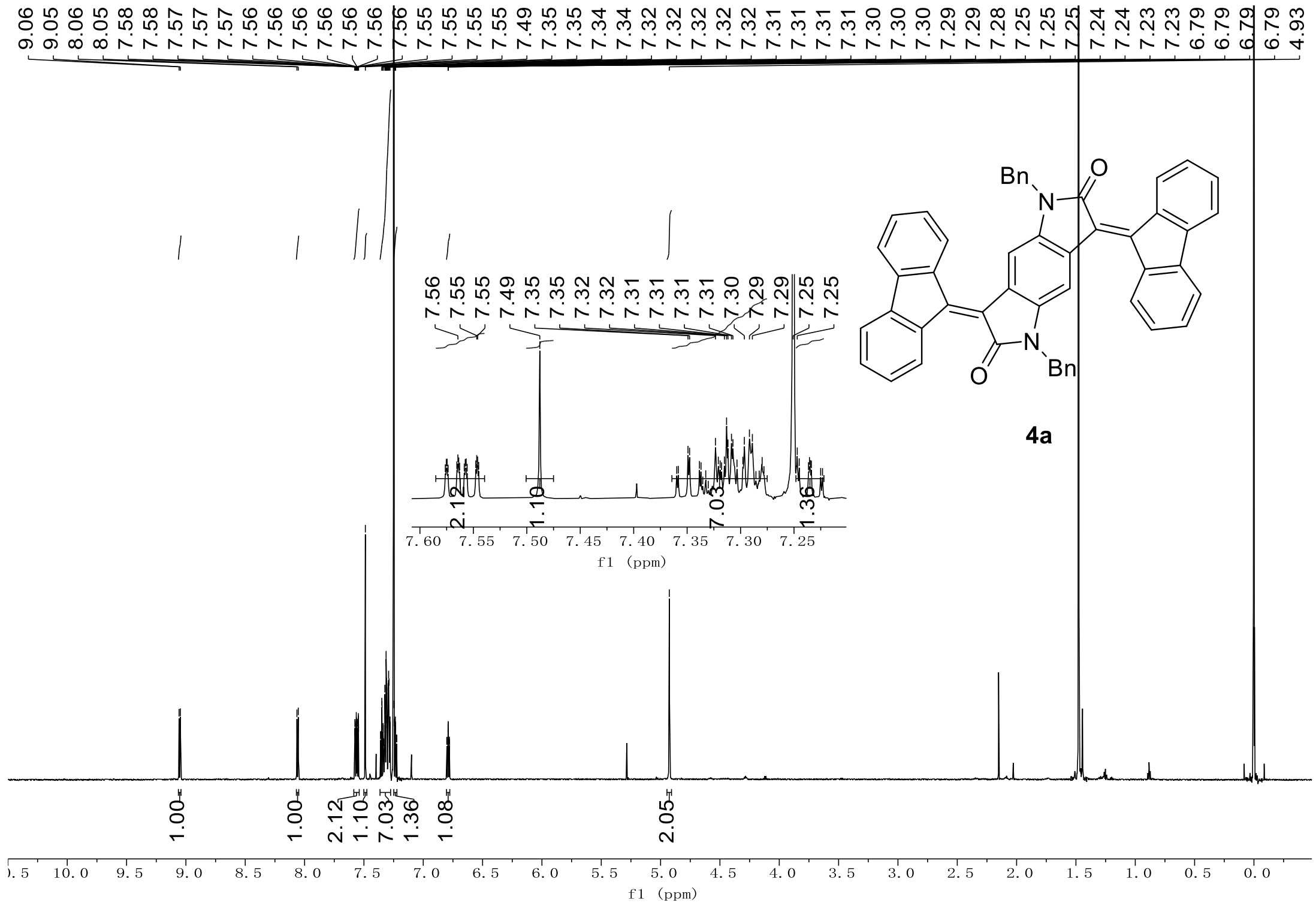

^{13}C NMR (175 MHz, $CDCl_3$)

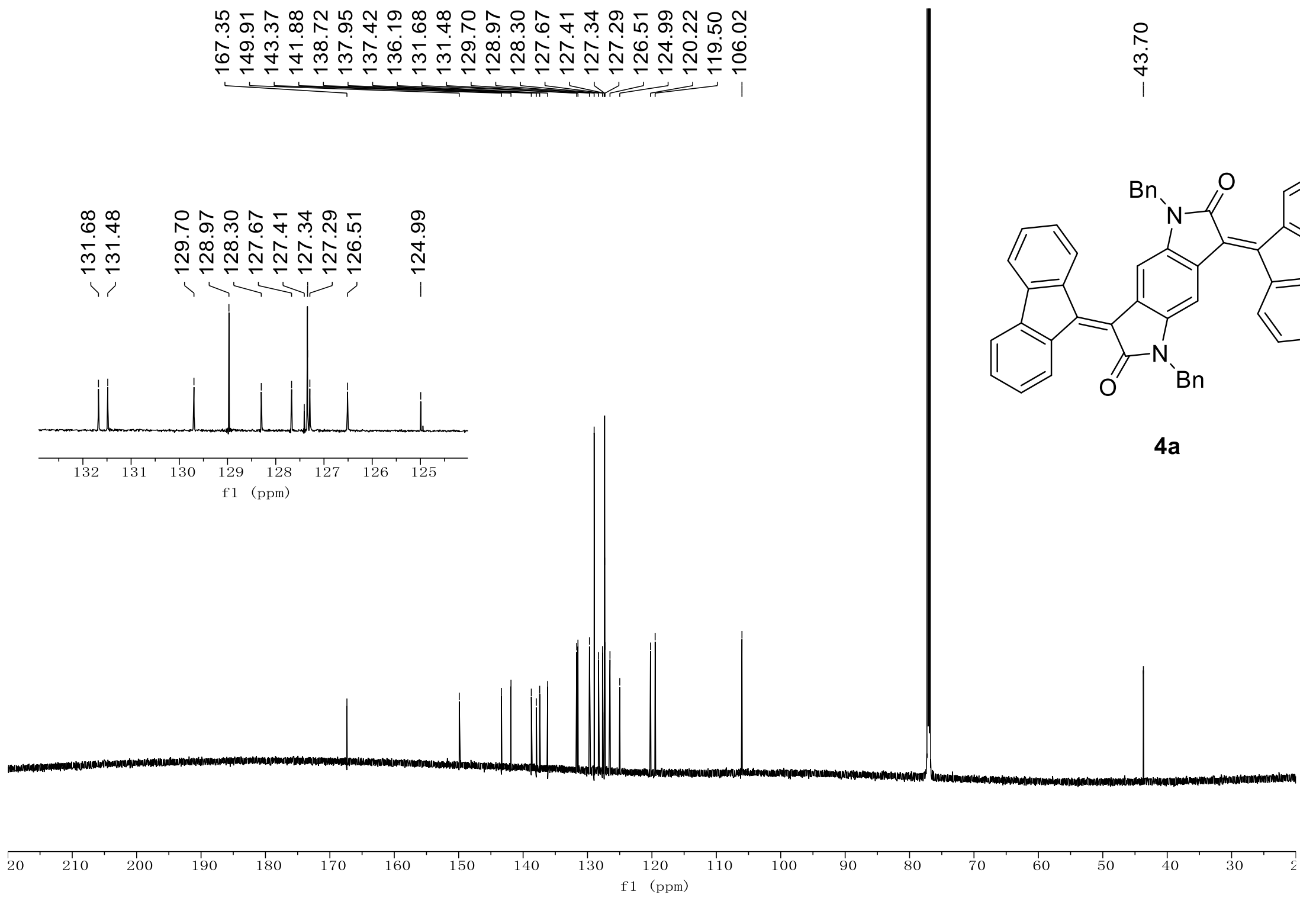

^{1}H NMR (400 MHz, $CDCl_3$)

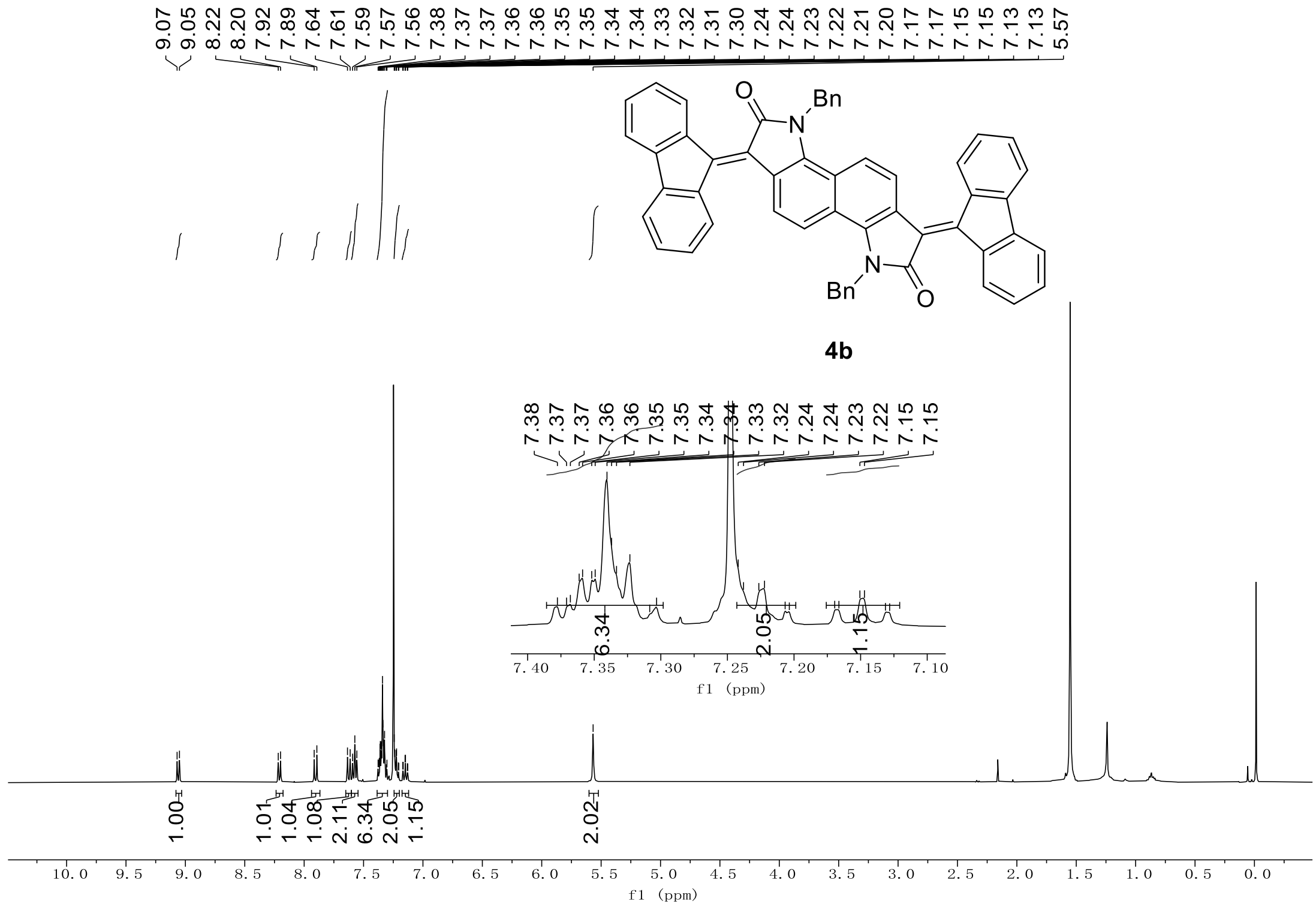

^{13}C NMR (100 MHz, $CDCl_3$)

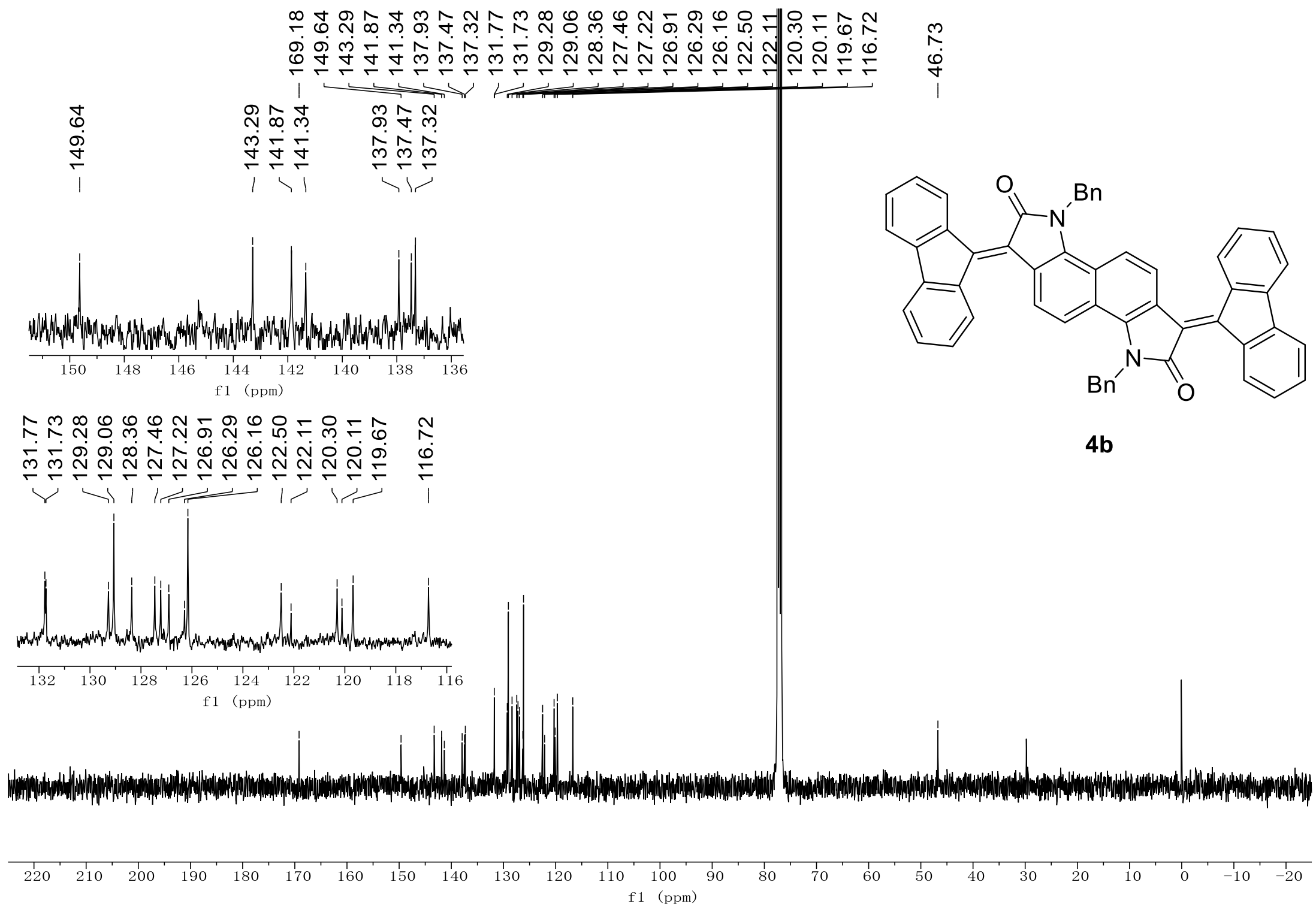

^{1}H NMR (400 MHz, $CDCl_3$)

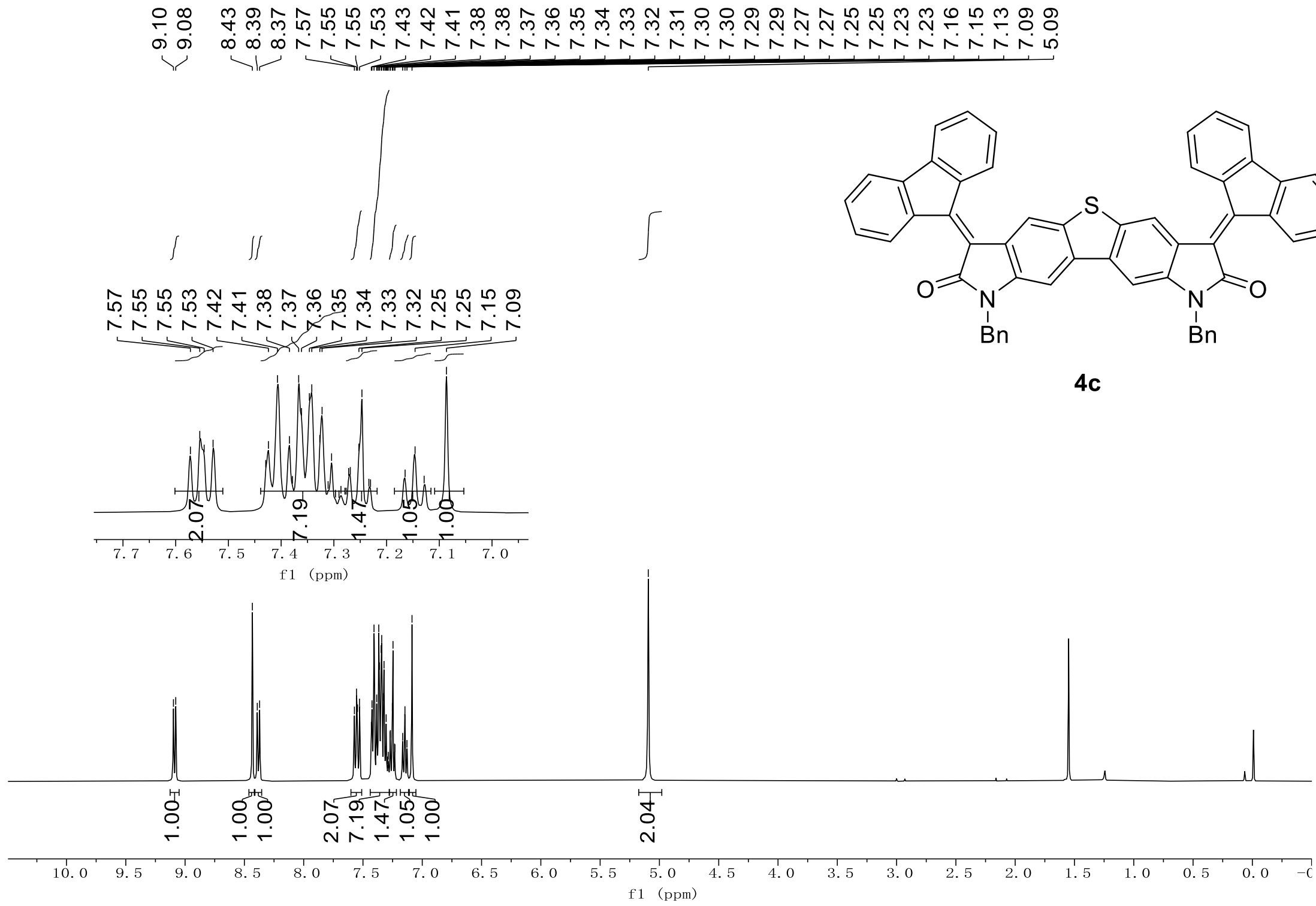

^{13}C NMR (100 MHz, $CDCl_3$)

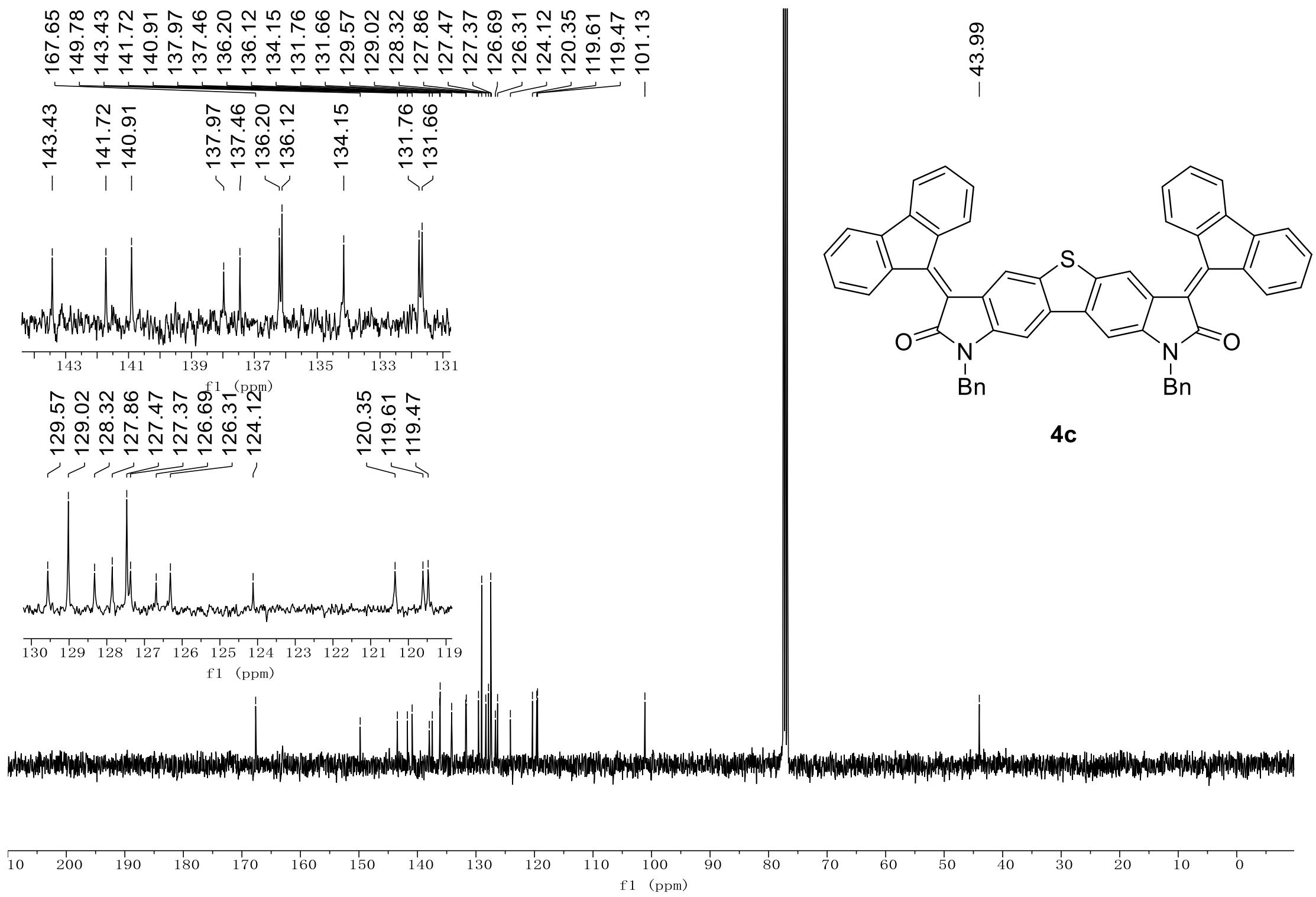

^{1}H NMR (400 MHz, $CDCl_3$)

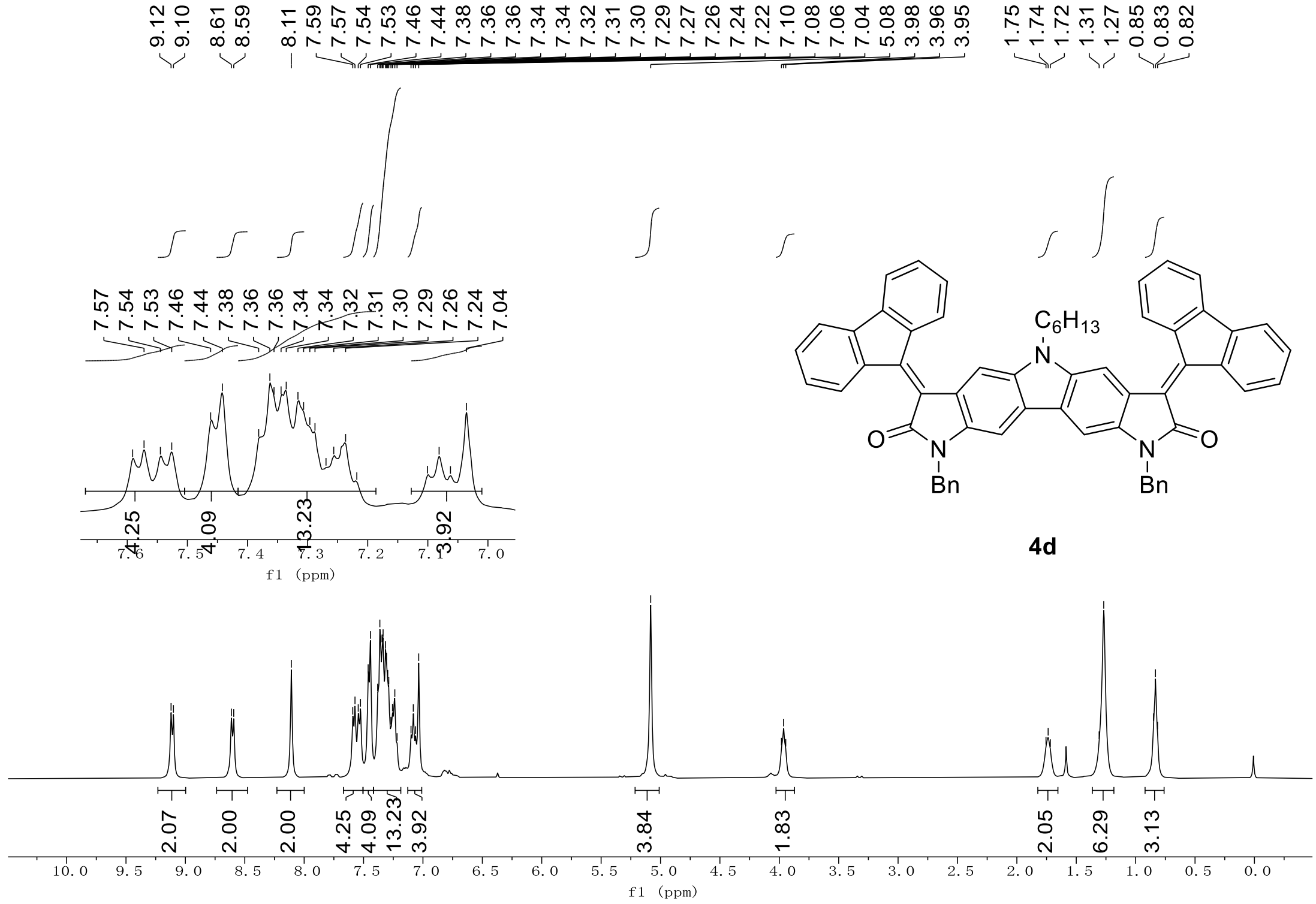

^{13}C NMR (100 MHz, $CDCl_3$)

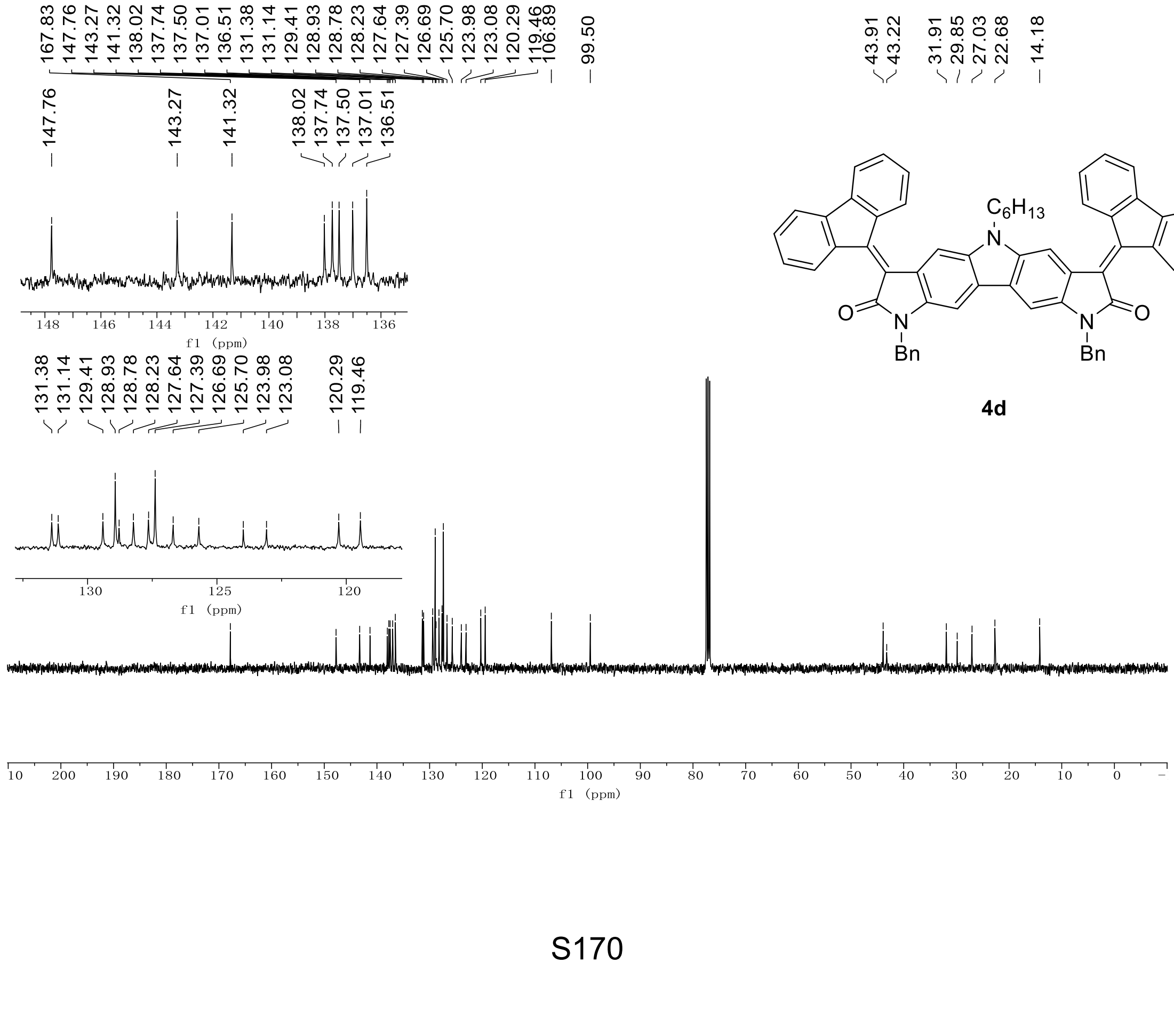

^{1}H NMR (400 MHz, CDCl$_3$)

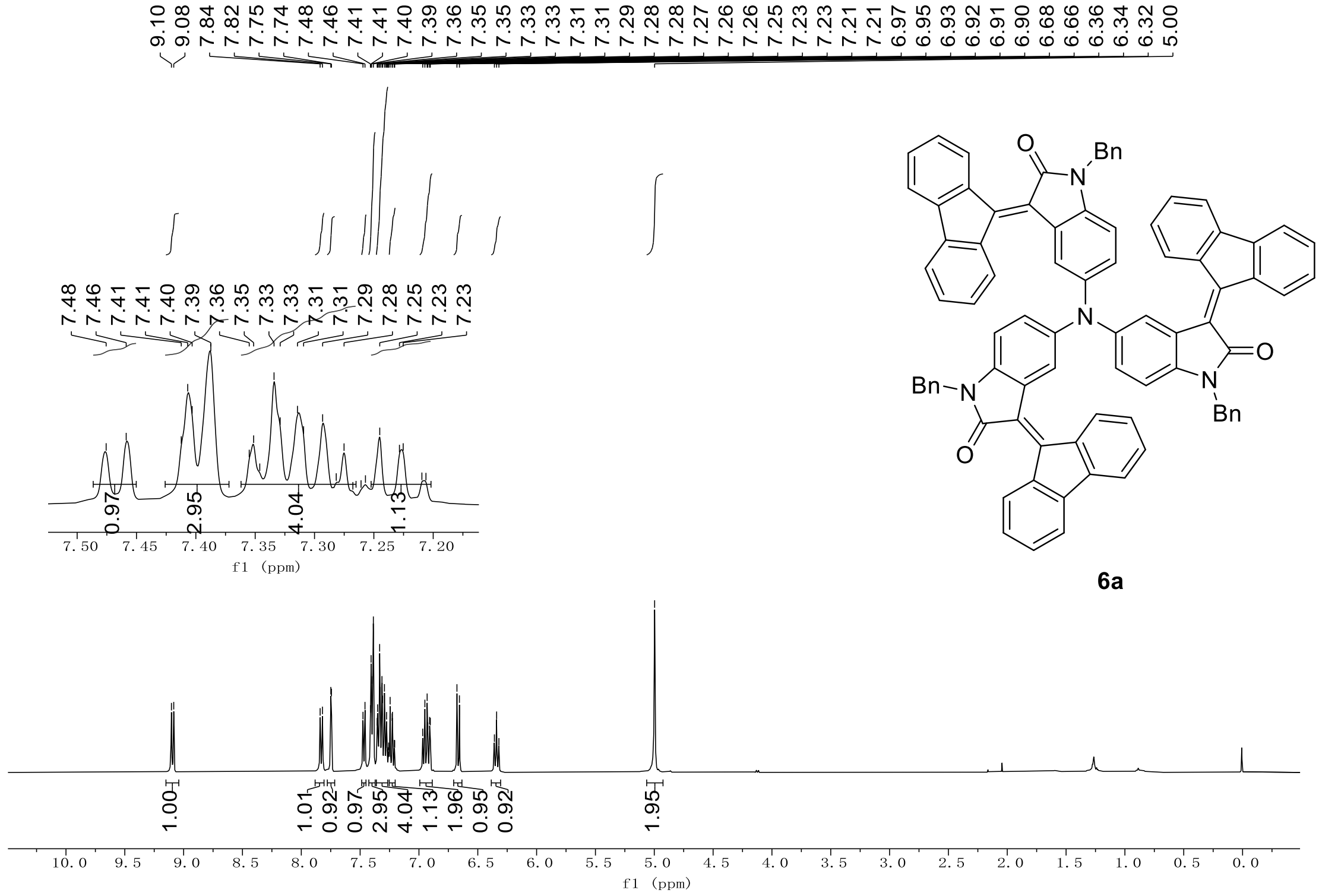

^{13}C NMR (100 MHz, CDCl$_3$)

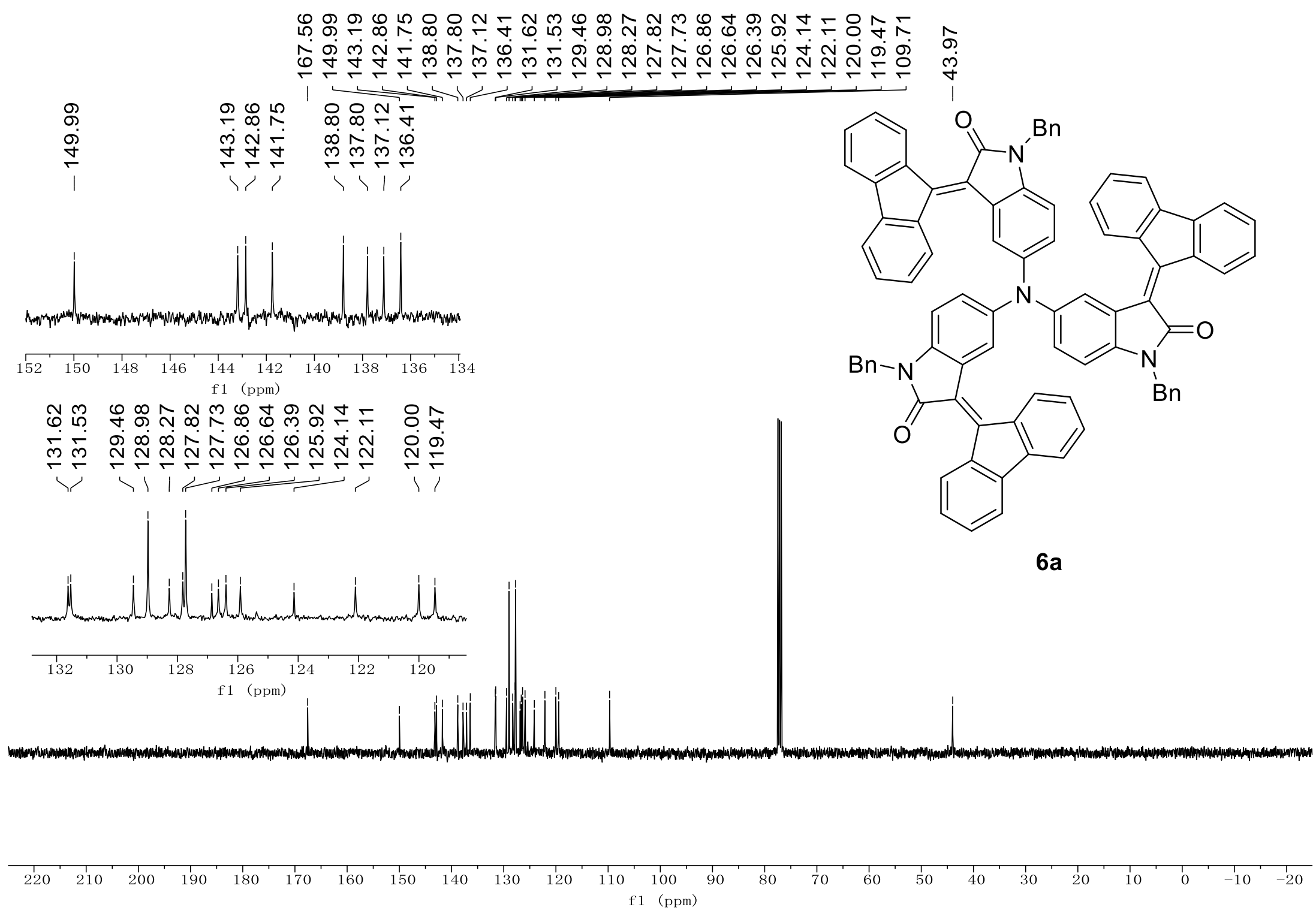

^{1}H NMR (400 MHz, $CDCl_3$)

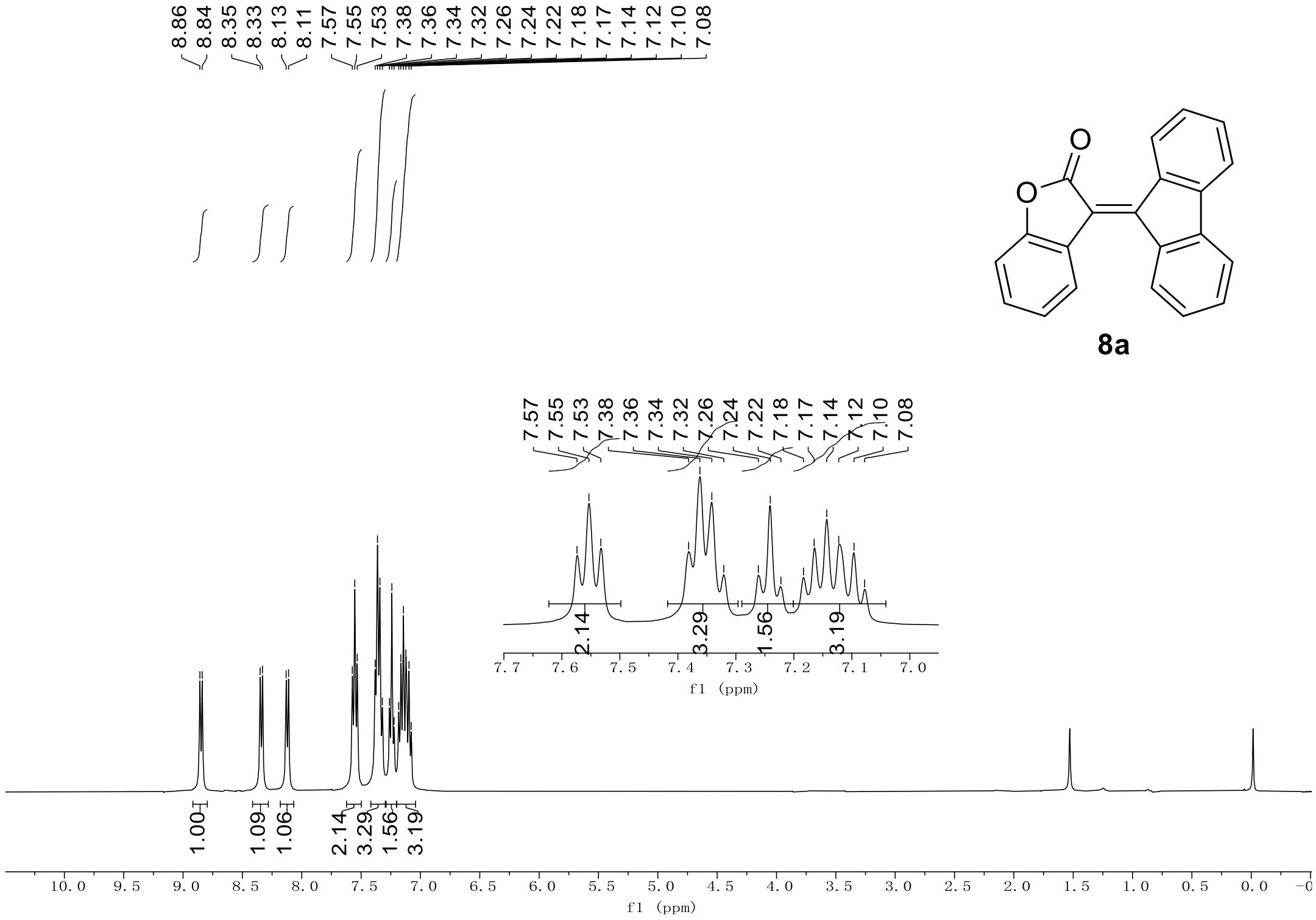

8a

^{13}C NMR (100 MHz, $CDCl_3$)

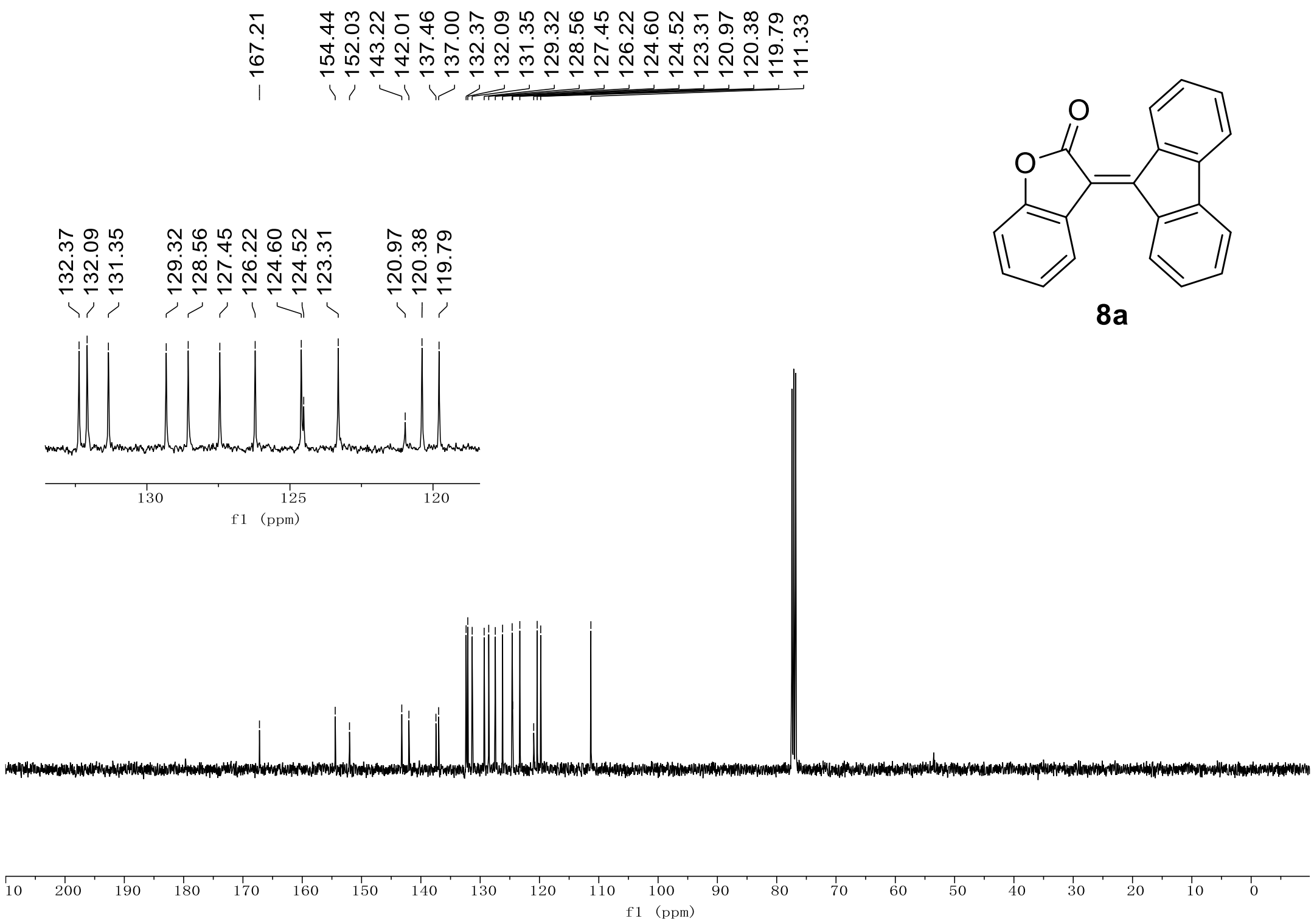

8a

^{1}H NMR (400 MHz, $CDCl_3$)

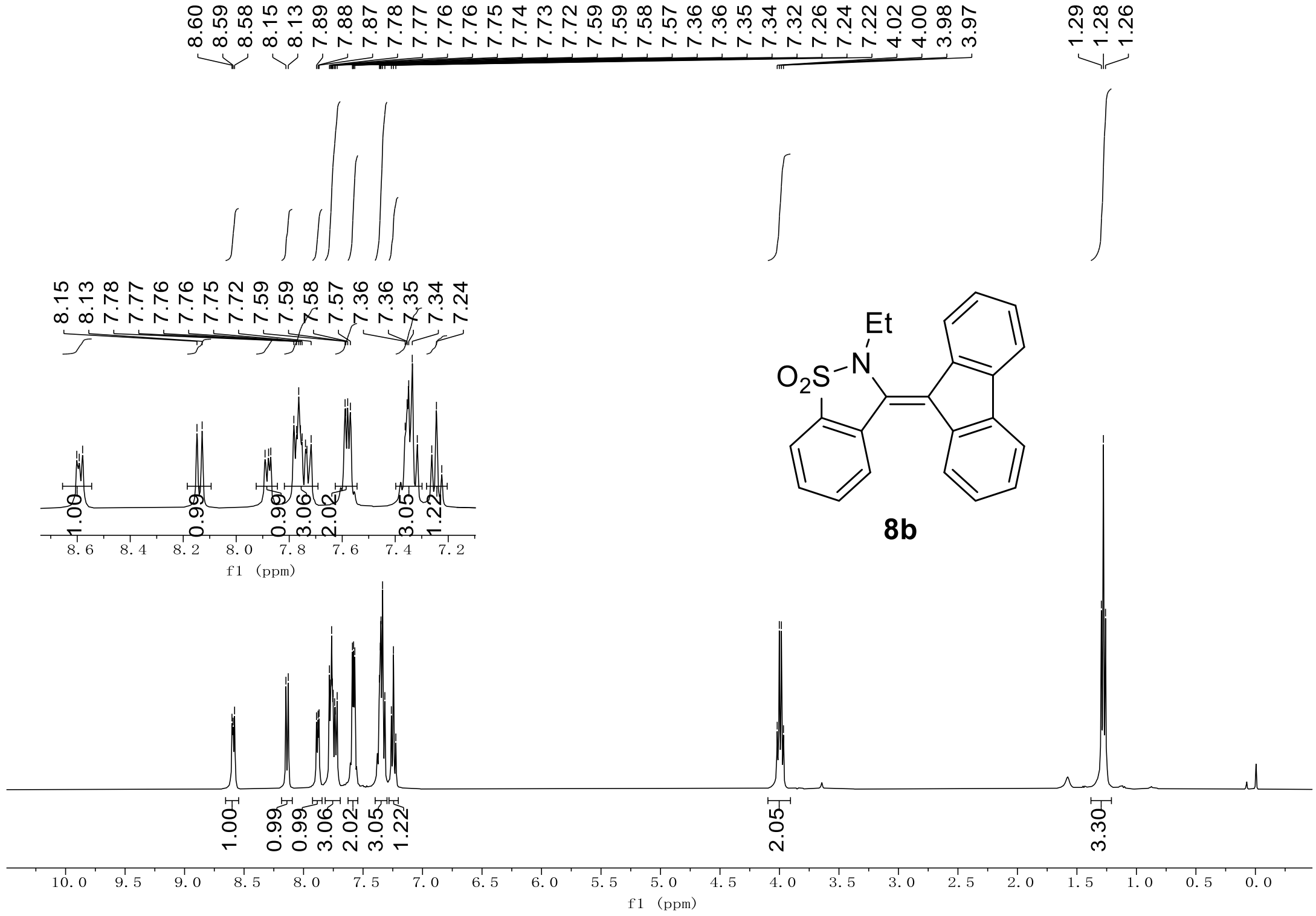

^{13}C NMR (100 MHz, $CDCl_3$)

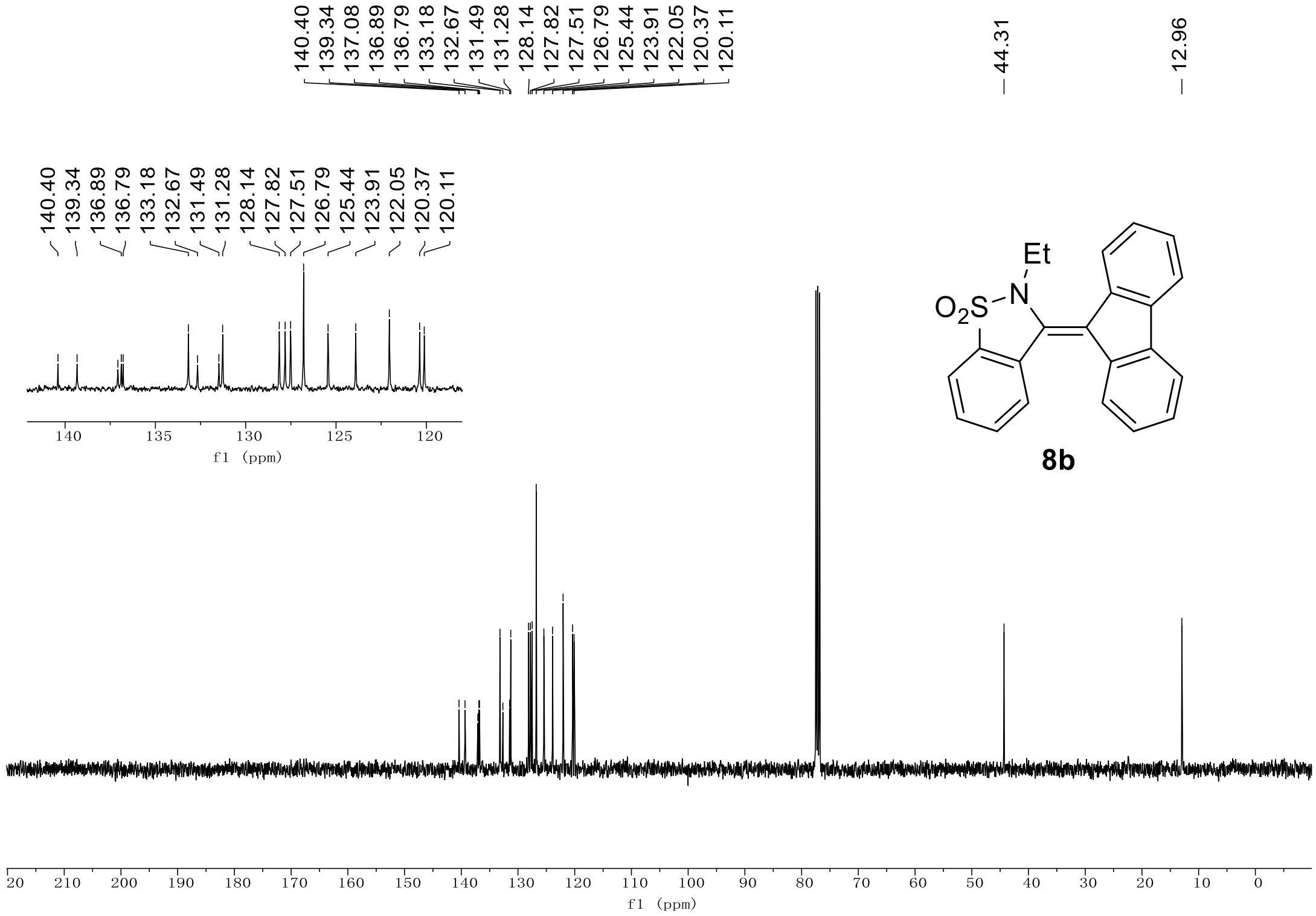

^{1}H NMR (400 MHz, $CDCl_3$)

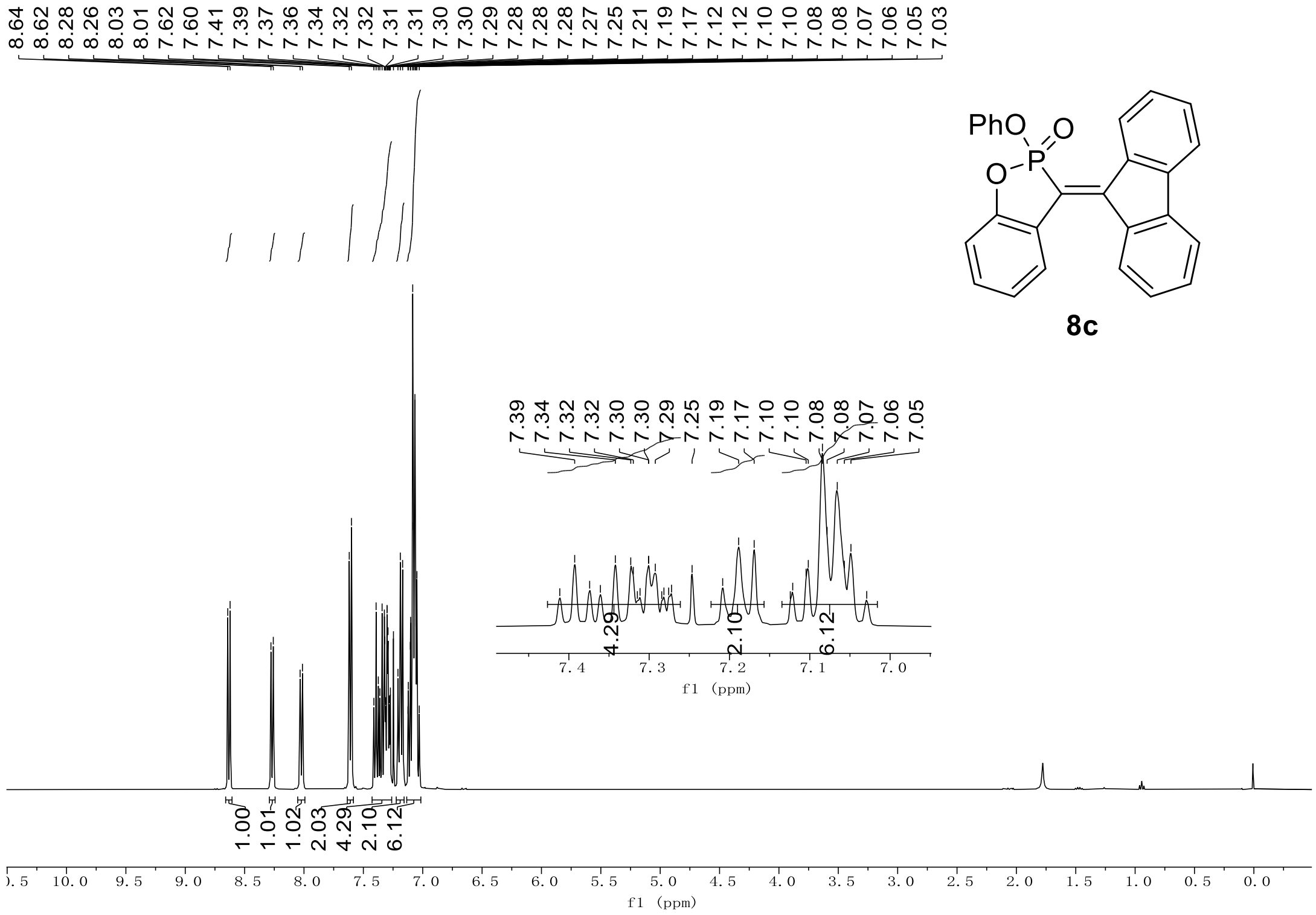

^{13}C NMR (100 MHz, $CDCl_3$)

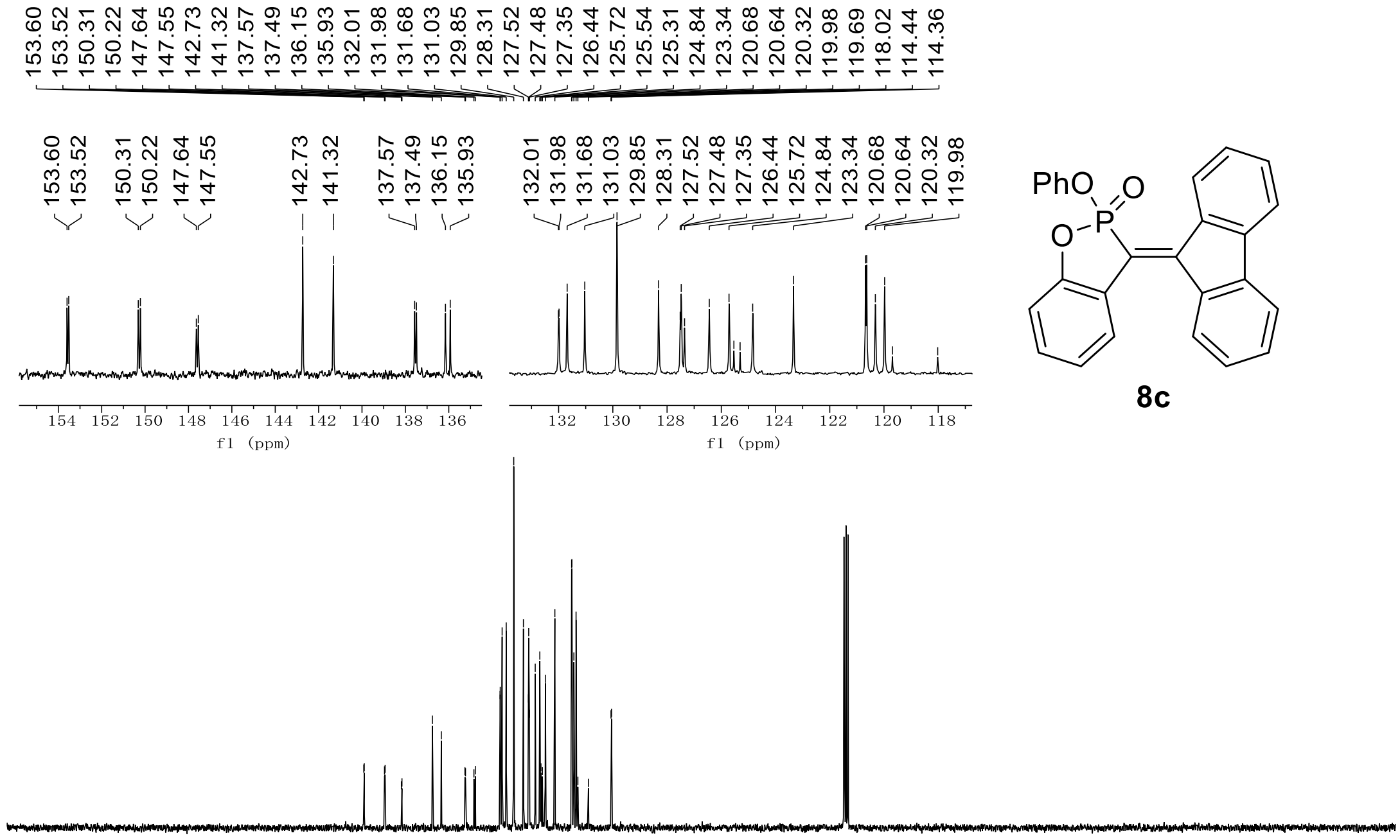

^{31}P NMR (162 MHz, $CDCl_3$)

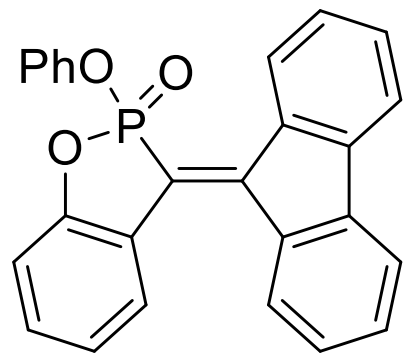

8c

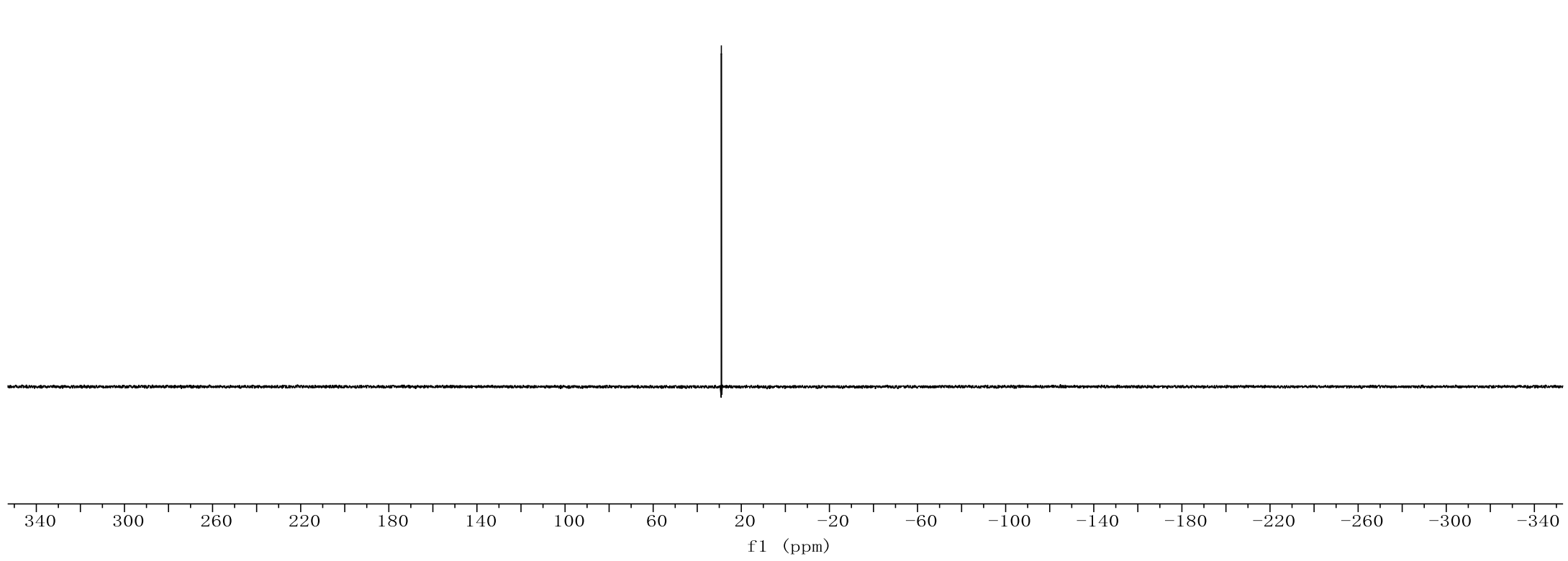

12. References

[1] D. Qian, J. Zhang, *Chem. Commun.* **2012***, 48,* 7082–7084.

[2] Y. Chen, D.-G. Chen, Y.-A. Chen, C.-H. Wu, K.-H. Chang, F.-Y. Meng, M.-C. Chen, J.-A. Lin, C.-Y. Huang, J. Su, H. Tian, P.-T. Chou, *Chem. Eur. J.* **2019**, *25*, 16755.

[3] C. Colletto, A. Panigrahi, J. Fernandez-Casado, I. Larrosa, *J. Am. Chem. Soc.* **2018**, *140*, 9638−9643.

[4] J. A. Birrell, J.-N. Desrosiers, E. N. Jacobsen, *J. Am. Chem. Soc*. **2011**, *133*, 13872–13875

[5] E. Sawicki, *J Am. Chem. Soc*. **1954**, *76*, 3, 664–667.

[6] H. Tsuno, J. Shen, H. Komatsu, N. Arichi, S. Inuki, H. Ohno, *Angew. Chem. Int. Ed.* **2023**, *62,* e202307532.

[7] X. Wang, F Zhuang, X. Zhou, D. Yang, J. Wang, J. Pei, *J. Mater. Chem. C.* **2014**, *2*, 8152–8161

[8] V. Akhmetov, M. Feofanov, D. I. Sharapa, K. Amsharov, *J. Am. Chem. Soc*. **2021**, *143*, 15420–15426.

[9] D. S. Schirra, M. Hirschmann, I. A. Radulov, M. Lehmann, C. M. Thiele, *Angew. Chem. Int. Ed.* **2021**, *60*, 21040.

[10] S. Sharma, R. S. Pathare, A. K. Maurya, K. Gopal, T. K. Roy, D. M. Sawant, R. T. Pardasani, *Org. Lett.* **2016**, *18*, 356–359

[11] A. Jančařík, J. Rybáček, K. Cocq, J. Vacek Chocholoušová, J. Vacek, R. Pohl, L. Bednárová, P. Fiedler, I. Císařová, I. G. Stará, I. Starý, *Angew. Chem. Int. Ed.* **2013**, *52*, 9970–9975.

[12] F. C. Krebs, M. Jørgensen, *J. Org. Chem*. **2002**, *67*, 7511–7518

[13] J. Korang, W. R. Grither, R. D. McCulla, *J. Am. Chem. Soc*. **2010**, *132*, 4466–4476

[14] İ. Kaya, S. Koyuncu, *J. Appl. Polym. Sci.* **2009**, *113*, 1975–1985.

[15] J.-T. Wu, T.-L. Hsiang, G.-S. Liou, *J. Mater. Chem. C* **2018**, *6*, 13345–13351.

[16] B.-C. Tzeng, A. Chao, T. Selvama, T.-Y. Chang, *CrystEngComm.* **2013**, *15*, 5337–5344

[17] J. Cai, L. Zhao, J. Wei, C. He, S. Longa, C. Duan, *Chem. Commun.* **2019**, *55*, 8524–8527

[18] Y. Zhong, Y. He, Y. Hu, X. Yan, R. Zhang, J. Yi, F. Zhao, B. Fan, *Org. Lett.* **2025**, *27*, 2180–2185.

[19] L. Zhu, Y. Yu, Z. Mao, X. Huang, *Org. Lett.* **2015**, *17*, 30–33.

[20] C. Schneider, E. Broda, V. Snieckus, *Org. Lett.* **2011**, *13*, 3588–3591.

[21] C.-E. Kim, T. Ryu, S. Kim, K. Lee, C.-H. Lee, P. H. Lee, *Adv. Synth. Catal.* **2013**, *355*, 2873–2883.
